# Supplementary material for: Serotonin Promotes Serum Albumin Interaction with the Monomeric Amyloid β Peptide
Source: Int J Mol Sci. 2021 May 31;22(11):5896. doi: 10.3390/ijms22115896 (PMC8199245; doi:10.3390/ijms22115896)
Supplement: Supplementary file 1 [file ijms-22-05896-s001.zip › ijms-1232894-supplementary.pdf]

Supplementary materials

Serotonin Promotes Serum Albumin Interaction with Monomeric Amyloid β Peptide

Ekaterina A. Litus[1], Alexey S. Kazakov[1], Evgenia I. Deryusheva[1], Ekaterina L. Nemashkalova [1], Marina P. Shevelyova[1], Aliya A. Nazipova[1], Maria E. Permyakova[1], Elena V. Raznikova[1], Vladimir N. Uversky[2,*], Sergei E. Permyakov[1,*]

[1] Institute for Biological Instrumentation, Pushchino Scientific Center for Biological Research of the Russian Academy of Sciences, Pushchino, Moscow Region 142290, Russia; *ealitus@gmail.com* (E.A.L.); *fenixfly@yandex.ru* (A.S.K.); *janed1986@ya.ru* (E.I.D.); *elnemashkalova@gmail.com* (E.L.N.); *marina.shevelyova@gmail.com* (M.P.S.); *alija-alex@rambler.ru* (A.A.N.); *mperm1977@gmail.com* (M.E.P.); *raznikova.elena@yandex.ru* (E.V.R.); *permyakov.s@gmail.com* (S.E.P.)

[2] Department of Molecular Medicine and USF Health Byrd Alzheimer's Research Institute, Morsani College of Medicine, University of South Florida, Tampa, FL 33612, USA; *vuversky@usf.edu*

* Correspondence: (V.N.U.); *permyakov.s@gmail.com* (S.E.P.); Tel.: +7-(495)-143-7741 (S.E.P.); Fax: +7-(4967)-33-05-22 (S.E.P.)

ALB_SRO.pdb and ALB_TRP.pdb files: model tertiary structures of HSA-SRO/TRP complexes predicted using AutoDock Vina software (http://vina.scripps.edu/index.html) [1], based on crystal structure of HSA (chain A of PDB entry 1UOR).

1. Trott, O. and A.J. Olson, *AutoDock Vina: improving the speed and accuracy of docking with a new scoring function, efficient optimization, and multithreading.* J Comput Chem, 2010. **31**(2): p. 455-61.

ALB_SRO.pdb

```
CRYST1   58.860   88.280   60.680  90.00 101.85  90.00 P 1 21 1      0
ATOM      1  N   LYS A   4      -4.155 -21.388 -11.190  1.00 29.11           N
ATOM      2  CA  LYS A   4      -3.964 -19.918 -11.113  1.00 27.16           C
ATOM      3  C   LYS A   4      -2.692 -19.737 -10.315  1.00 26.91           C
ATOM      4  O   LYS A   4      -2.108 -20.724  -9.881  1.00 25.06           O
ATOM      5  CB  LYS A   4      -5.146 -19.257 -10.398  1.00 25.62           C
ATOM      6  CG  LYS A   4      -6.503 -19.517 -11.051  1.00 24.88           C
ATOM      7  CD  LYS A   4      -7.331 -18.233 -11.175  1.00 26.34           C
ATOM      8  CE  LYS A   4      -8.649 -18.441 -11.958  1.00 27.70           C
ATOM      9  NZ  LYS A   4      -9.717 -19.227 -11.215  1.00 25.48           N1+
ATOM     10  N   SER A   5      -2.168 -18.518 -10.290  1.00 28.23           N
ATOM     11  CA  SER A   5      -0.939 -18.247  -9.551  1.00 30.21           C
ATOM     12  C   SER A   5      -1.207 -17.300  -8.380  1.00 30.60           C
ATOM     13  O   SER A   5      -2.299 -16.719  -8.247  1.00 28.63           O
ATOM     14  CB  SER A   5       0.155 -17.652 -10.476  1.00 30.07           C
ATOM     15  OG  SER A   5       0.110 -16.225 -10.543  1.00 25.84           O
ATOM     16  N   GLU A   6      -0.206 -17.159  -7.526  1.00 29.67           N
ATOM     17  CA  GLU A   6      -0.250 -16.146  -6.511  1.00 28.73           C
ATOM     18  C   GLU A   6       0.506 -14.913  -6.949  1.00 25.98           C
ATOM     19  O   GLU A   6      -0.094 -13.854  -7.065  1.00 25.99           O
ATOM     20  CB  GLU A   6       0.289 -16.706  -5.196  1.00 32.20           C
ATOM     21  CG  GLU A   6      -0.730 -17.561  -4.457  1.00 34.86           C
ATOM     22  CD  GLU A   6      -2.158 -17.229  -4.877  1.00 37.50           C
ATOM     23  OE1 GLU A   6      -2.669 -16.146  -4.502  1.00 36.49           O
```

ATOM	24	OE2	GLU	A	6	-2.746	-18.025	-5.641	1.00	41.69	O1-
ATOM	25	N	VAL	A	7	1.732	-15.105	-7.428	1.00	26.46	N
ATOM	26	CA	VAL	A	7	2.660	-13.992	-7.658	1.00	28.01	C
ATOM	27	C	VAL	A	7	2.019	-12.877	-8.472	1.00	29.09	C
ATOM	28	O	VAL	A	7	1.728	-11.784	-7.951	1.00	28.54	O
ATOM	29	CB	VAL	A	7	3.929	-14.439	-8.413	1.00	27.74	C
ATOM	30	CG1	VAL	A	7	4.947	-13.333	-8.405	1.00	29.26	C
ATOM	31	CG2	VAL	A	7	4.524	-15.668	-7.773	1.00	31.03	C
ATOM	32	N	ALA	A	8	1.681	-13.202	-9.716	1.00	29.77	N
ATOM	33	CA	ALA	A	8	1.215	-12.182	-10.646	1.00	29.95	C
ATOM	34	C	ALA	A	8	-0.133	-11.672	-10.189	1.00	29.29	C
ATOM	35	O	ALA	A	8	-0.491	-10.525	-10.473	1.00	30.27	O
ATOM	36	CB	ALA	A	8	1.117	-12.736	-12.073	1.00	29.88	C
ATOM	37	N	HIS	A	9	-0.866	-12.500	-9.452	1.00	28.46	N
ATOM	38	CA	HIS	A	9	-2.215	-12.121	-9.052	1.00	28.28	C
ATOM	39	C	HIS	A	9	-2.121	-10.972	-8.070	1.00	28.15	C
ATOM	40	O	HIS	A	9	-2.926	-10.045	-8.124	1.00	28.25	O
ATOM	41	CB	HIS	A	9	-2.961	-13.275	-8.387	1.00	26.15	C
ATOM	42	CG	HIS	A	9	-4.260	-12.866	-7.776	1.00	23.88	C
ATOM	43	CD2	HIS	A	9	-5.086	-11.821	-8.052	1.00	22.55	C
ATOM	44	ND1	HIS	A	9	-4.868	-13.577	-6.766	1.00	22.06	N
ATOM	45	CE1	HIS	A	9	-6.009	-12.994	-6.443	1.00	23.71	C
ATOM	46	NE2	HIS	A	9	-6.162	-11.927	-7.212	1.00	25.62	N
ATOM	47	N	ARG	A	10	-1.181	-11.070	-7.134	1.00	26.61	N
ATOM	48	CA	ARG	A	10	-0.934	-9.977	-6.211	1.00	26.64	C
ATOM	49	C	ARG	A	10	-0.481	-8.776	-7.042	1.00	26.73	C
ATOM	50	O	ARG	A	10	-1.080	-7.701	-6.952	1.00	26.46	O
ATOM	51	CB	ARG	A	10	0.123	-10.384	-5.179	1.00	25.45	C
ATOM	52	CG	ARG	A	10	-0.411	-11.233	-3.973	1.00	26.28	C
ATOM	53	CD	ARG	A	10	-1.808	-11.885	-4.176	1.00	22.90	C
ATOM	54	NE	ARG	A	10	-2.565	-11.999	-2.920	1.00	22.44	N
ATOM	55	CZ	ARG	A	10	-3.843	-12.387	-2.815	1.00	23.29	C
ATOM	56	NH1	ARG	A	10	-4.491	-12.934	-3.836	1.00	20.37	N1+
ATOM	57	NH2	ARG	A	10	-4.460	-12.295	-1.650	1.00	22.05	N
ATOM	58	N	PHE	A	11	0.332	-9.061	-8.061	1.00	27.91	N
ATOM	59	CA	PHE	A	11	0.735	-8.053	-9.045	1.00	25.84	C
ATOM	60	C	PHE	A	11	-0.452	-7.383	-9.727	1.00	24.74	C
ATOM	61	O	PHE	A	11	-0.444	-6.168	-9.938	1.00	25.29	O
ATOM	62	CB	PHE	A	11	1.652	-8.664	-10.105	1.00	24.40	C
ATOM	63	CG	PHE	A	11	2.851	-7.809	-10.430	1.00	27.14	C
ATOM	64	CD1	PHE	A	11	2.762	-6.785	-11.368	1.00	26.91	C
ATOM	65	CD2	PHE	A	11	4.042	-7.965	-9.728	1.00	26.00	C
ATOM	66	CE1	PHE	A	11	3.829	-5.934	-11.585	1.00	27.01	C
ATOM	67	CE2	PHE	A	11	5.115	-7.111	-9.945	1.00	25.82	C
ATOM	68	CZ	PHE	A	11	5.009	-6.095	-10.871	1.00	25.28	C
ATOM	69	N	LYS	A	12	-1.530	-8.136	-9.903	1.00	23.91	N
ATOM	70	CA	LYS	A	12	-2.692	-7.668	-10.651	1.00	22.95	C
ATOM	71	C	LYS	A	12	-3.685	-6.841	-9.819	1.00	22.94	C
ATOM	72	O	LYS	A	12	-4.825	-6.624	-10.255	1.00	22.98	O
ATOM	73	CB	LYS	A	12	-3.449	-8.860	-11.267	1.00	23.41	C
ATOM	74	CG	LYS	A	12	-2.859	-9.489	-12.523	1.00	18.48	C
ATOM	75	CD	LYS	A	12	-3.793	-10.579	-13.069	1.00	15.47	C
ATOM	76	CE	LYS	A	12	-3.024	-11.641	-13.836	1.00	13.47	C
ATOM	77	NZ	LYS	A	12	-2.824	-11.308	-15.273	1.00	12.20	N1+
ATOM	78	N	ASP	A	13	-3.313	-6.444	-8.606	1.00	22.30	N
ATOM	79	CA	ASP	A	13	-4.140	-5.462	-7.897	1.00	22.25	C
ATOM	80	C	ASP	A	13	-3.358	-4.266	-7.363	1.00	20.77	C
ATOM	81	O	ASP	A	13	-3.612	-3.114	-7.758	1.00	18.06	O
ATOM	82	CB	ASP	A	13	-4.929	-6.102	-6.748	1.00	27.23	C
ATOM	83	CG	ASP	A	13	-4.846	-7.609	-6.734	1.00	30.68	C
ATOM	84	OD1	ASP	A	13	-3.712	-8.130	-6.758	1.00	31.81	O
ATOM	85	OD2	ASP	A	13	-5.905	-8.262	-6.569	1.00	31.63	O1-
ATOM	86	N	LEU	A	14	-2.360	-4.555	-6.527	1.00	19.90	N
ATOM	87	CA	LEU	A	14	-1.567	-3.530	-5.840	1.00	19.12	C
ATOM	88	C	LEU	A	14	-0.542	-2.906	-6.802	1.00	21.59	C
ATOM	89	O	LEU	A	14	0.414	-2.214	-6.388	1.00	20.70	O
ATOM	90	CB	LEU	A	14	-0.867	-4.154	-4.625	1.00	16.73	C
ATOM	91	CG	LEU	A	14	0.256	-5.186	-4.824	1.00	15.13	C
ATOM	92	CD1	LEU	A	14	1.447	-4.781	-3.981	1.00	11.65	C
ATOM	93	CD2	LEU	A	14	-0.189	-6.568	-4.441	1.00	11.37	C
ATOM	94	N	GLY	A	15	-0.829	-3.065	-8.092	1.00	21.89	N

```
ATOM     95  CA  GLY A  15       0.087  -2.683  -9.140  1.00 23.39           C
ATOM     96  C   GLY A  15       1.491  -3.220  -8.965  1.00 24.62           C
ATOM     97  O   GLY A  15       1.700  -4.264  -8.353  1.00 23.01           O
ATOM     98  N   GLU A  16       2.457  -2.395  -9.367  1.00 26.22           N
ATOM     99  CA  GLU A  16       3.837  -2.809  -9.504  1.00 25.91           C
ATOM    100  C   GLU A  16       4.725  -2.038  -8.535  1.00 27.19           C
ATOM    101  O   GLU A  16       5.421  -2.680  -7.760  1.00 26.80           O
ATOM    102  CB  GLU A  16       4.282  -2.641 -10.966  1.00 27.05           C
ATOM    103  CG  GLU A  16       5.730  -2.204 -11.175  1.00 28.89           C
ATOM    104  CD  GLU A  16       6.302  -2.613 -12.537  1.00 29.95           C
ATOM    105  OE1 GLU A  16       5.637  -2.398 -13.596  1.00 28.27           O
ATOM    106  OE2 GLU A  16       7.444  -3.137 -12.531  1.00 28.17           O1-
ATOM    107  N   GLU A  17       4.596  -0.699  -8.451  1.00 30.38           N
ATOM    108  CA  GLU A  17       5.444   0.099  -7.520  1.00 30.91           C
ATOM    109  C   GLU A  17       5.306  -0.525  -6.158  1.00 32.10           C
ATOM    110  O   GLU A  17       6.187  -1.268  -5.730  1.00 34.11           O
ATOM    111  CB  GLU A  17       5.044   1.589  -7.404  1.00 28.23           C
ATOM    112  CG  GLU A  17       6.165   2.451  -6.706  1.00 30.17           C
ATOM    113  CD  GLU A  17       5.688   3.762  -5.967  1.00 32.12           C
ATOM    114  OE1 GLU A  17       5.082   4.666  -6.598  1.00 30.67           O
ATOM    115  OE2 GLU A  17       6.027   3.939  -4.760  1.00 31.49           O1-
ATOM    116  N   ASN A  18       4.054  -0.563  -5.720  1.00 33.09           N
ATOM    117  CA  ASN A  18       3.696  -1.121  -4.420  1.00 32.50           C
ATOM    118  C   ASN A  18       3.913  -2.668  -4.325  1.00 31.11           C
ATOM    119  O   ASN A  18       3.947  -3.231  -3.212  1.00 30.55           O
ATOM    120  CB  ASN A  18       2.249  -0.690  -4.057  1.00 31.08           C
ATOM    121  CG  ASN A  18       2.086   0.851  -3.988  1.00 28.14           C
ATOM    122  ND2 ASN A  18       0.918   1.344  -4.361  1.00 28.12           N
ATOM    123  OD1 ASN A  18       3.018   1.572  -3.648  1.00 24.14           O
ATOM    124  N   PHE A  19       4.287  -3.307  -5.437  1.00 27.36           N
ATOM    125  CA  PHE A  19       4.761  -4.691  -5.361  1.00 25.30           C
ATOM    126  C   PHE A  19       6.240  -4.636  -5.071  1.00 25.50           C
ATOM    127  O   PHE A  19       6.703  -5.188  -4.078  1.00 26.44           O
ATOM    128  CB  PHE A  19       4.513  -5.455  -6.667  1.00 24.34           C
ATOM    129  CG  PHE A  19       4.448  -6.959  -6.504  1.00 21.73           C
ATOM    130  CD1 PHE A  19       5.594  -7.720  -6.488  1.00 21.65           C
ATOM    131  CD2 PHE A  19       3.236  -7.616  -6.507  1.00 21.52           C
ATOM    132  CE1 PHE A  19       5.532  -9.106  -6.486  1.00 18.58           C
ATOM    133  CE2 PHE A  19       3.175  -8.997  -6.508  1.00 20.40           C
ATOM    134  CZ  PHE A  19       4.328  -9.738  -6.499  1.00 18.31           C
ATOM    135  N   LYS A  20       6.957  -3.831  -5.839  1.00 24.36           N
ATOM    136  CA  LYS A  20       8.340  -3.545  -5.501  1.00 26.07           C
ATOM    137  C   LYS A  20       8.335  -3.055  -4.053  1.00 26.27           C
ATOM    138  O   LYS A  20       9.090  -3.567  -3.221  1.00 25.42           O
ATOM    139  CB  LYS A  20       8.921  -2.458  -6.432  1.00 27.79           C
ATOM    140  CG  LYS A  20       9.948  -2.952  -7.490  1.00 26.76           C
ATOM    141  CD  LYS A  20       9.952  -2.041  -8.743  1.00 22.31           C
ATOM    142  CE  LYS A  20      10.898  -2.548  -9.824  1.00 20.10           C
ATOM    143  NZ  LYS A  20      12.337  -2.566  -9.387  1.00 19.71           N1+
ATOM    144  N   ALA A  21       7.287  -2.301  -3.711  1.00 26.57           N
ATOM    145  CA  ALA A  21       7.264  -1.504  -2.480  1.00 25.54           C
ATOM    146  C   ALA A  21       7.045  -2.381  -1.289  1.00 23.24           C
ATOM    147  O   ALA A  21       7.661  -2.178  -0.261  1.00 24.58           O
ATOM    148  CB  ALA A  21       6.188  -0.411  -2.539  1.00 24.10           C
ATOM    149  N   LEU A  22       6.251  -3.422  -1.444  1.00 22.80           N
ATOM    150  CA  LEU A  22       6.151  -4.371  -0.353  1.00 23.66           C
ATOM    151  C   LEU A  22       7.414  -5.226  -0.300  1.00 22.00           C
ATOM    152  O   LEU A  22       8.277  -4.987   0.543  1.00 22.37           O
ATOM    153  CB  LEU A  22       4.892  -5.242  -0.489  1.00 25.44           C
ATOM    154  CG  LEU A  22       3.648  -4.849   0.324  1.00 23.88           C
ATOM    155  CD1 LEU A  22       4.053  -4.132   1.586  1.00 21.55           C
ATOM    156  CD2 LEU A  22       2.740  -3.964  -0.500  1.00 23.25           C
ATOM    157  N   VAL A  23       7.649  -5.965  -1.382  1.00 19.37           N
ATOM    158  CA  VAL A  23       8.590  -7.075  -1.410  1.00 16.76           C
ATOM    159  C   VAL A  23      10.018  -6.698  -0.945  1.00 18.40           C
ATOM    160  O   VAL A  23      10.734  -7.519  -0.372  1.00 17.50           O
ATOM    161  CB  VAL A  23       8.579  -7.698  -2.823  1.00 15.62           C
ATOM    162  CG1 VAL A  23       9.429  -8.950  -2.878  1.00 13.90           C
ATOM    163  CG2 VAL A  23       7.157  -8.040  -3.211  1.00  9.89           C
ATOM    164  N   LEU A  24      10.336  -5.412  -0.999  1.00 20.80           N
ATOM    165  CA  LEU A  24      11.503  -4.879  -0.319  1.00 21.23           C
```

ATOM	166	C	LEU	A	24	11.278	-4.949	1.182	1.00	22.45	C
ATOM	167	O	LEU	A	24	11.885	-5.812	1.826	1.00	25.32	O
ATOM	168	CB	LEU	A	24	11.767	-3.434	-0.729	1.00	23.94	C
ATOM	169	CG	LEU	A	24	13.205	-2.910	-0.666	1.00	25.01	C
ATOM	170	CD1	LEU	A	24	13.154	-1.397	-0.414	1.00	24.47	C
ATOM	171	CD2	LEU	A	24	14.017	-3.618	0.417	1.00	24.82	C
ATOM	172	N	ILE	A	25	10.410	-4.100	1.756	1.00	21.80	N
ATOM	173	CA	ILE	A	25	10.241	-4.122	3.238	1.00	22.89	C
ATOM	174	C	ILE	A	25	10.125	-5.571	3.747	1.00	23.14	C
ATOM	175	O	ILE	A	25	10.935	-5.997	4.564	1.00	20.48	O
ATOM	176	CB	ILE	A	25	8.993	-3.319	3.785	1.00	19.69	C
ATOM	177	CG1	ILE	A	25	7.714	-3.751	3.081	1.00	18.78	C
ATOM	178	CG2	ILE	A	25	9.220	-1.841	3.691	1.00	14.48	C
ATOM	179	CD1	ILE	A	25	6.793	-2.619	2.745	1.00	19.12	C
ATOM	180	N	ALA	A	26	9.289	-6.364	3.071	1.00	23.61	N
ATOM	181	CA	ALA	A	26	9.182	-7.799	3.320	1.00	24.15	C
ATOM	182	C	ALA	A	26	10.563	-8.405	3.520	1.00	24.53	C
ATOM	183	O	ALA	A	26	10.954	-8.689	4.649	1.00	24.25	O
ATOM	184	CB	ALA	A	26	8.475	-8.487	2.149	1.00	23.75	C
ATOM	185	N	PHE	A	27	11.367	-8.391	2.458	1.00	25.34	N
ATOM	186	CA	PHE	A	27	12.700	-8.999	2.472	1.00	23.54	C
ATOM	187	C	PHE	A	27	13.657	-8.301	3.432	1.00	21.84	C
ATOM	188	O	PHE	A	27	14.755	-8.811	3.685	1.00	21.92	O
ATOM	189	CB	PHE	A	27	13.312	-8.985	1.067	1.00	22.66	C
ATOM	190	CG	PHE	A	27	12.781	-10.058	0.157	1.00	21.80	C
ATOM	191	CD1	PHE	A	27	12.742	-11.375	0.562	1.00	22.31	C
ATOM	192	CD2	PHE	A	27	12.383	-9.753	-1.130	1.00	19.87	C
ATOM	193	CE1	PHE	A	27	12.309	-12.365	-0.311	1.00	23.11	C
ATOM	194	CE2	PHE	A	27	11.955	-10.733	-1.999	1.00	17.81	C
ATOM	195	CZ	PHE	A	27	11.914	-12.034	-1.595	1.00	20.05	C
ATOM	196	N	ALA	A	28	13.258	-7.145	3.956	1.00	19.72	N
ATOM	197	CA	ALA	A	28	14.162	-6.345	4.775	1.00	20.10	C
ATOM	198	C	ALA	A	28	13.699	-5.958	6.201	1.00	21.53	C
ATOM	199	O	ALA	A	28	14.345	-5.141	6.856	1.00	21.18	O
ATOM	200	CB	ALA	A	28	14.589	-5.111	4.002	1.00	19.50	C
ATOM	201	N	GLN	A	29	12.557	-6.484	6.644	1.00	22.14	N
ATOM	202	CA	GLN	A	29	12.293	-6.695	8.068	1.00	19.68	C
ATOM	203	C	GLN	A	29	12.975	-8.025	8.333	1.00	22.08	C
ATOM	204	O	GLN	A	29	13.984	-8.084	9.029	1.00	21.67	O
ATOM	205	CB	GLN	A	29	10.785	-6.840	8.336	1.00	15.49	C
ATOM	206	CG	GLN	A	29	9.953	-5.582	8.068	1.00	16.39	C
ATOM	207	CD	GLN	A	29	8.445	-5.805	8.202	1.00	17.09	C
ATOM	208	NE2	GLN	A	29	7.699	-4.724	8.411	1.00	12.64	N
ATOM	209	OE1	GLN	A	29	7.959	-6.934	8.092	1.00	19.84	O
ATOM	210	N	TYR	A	30	12.622	-8.974	7.462	1.00	24.94	N
ATOM	211	CA	TYR	A	30	13.071	-10.369	7.483	1.00	23.92	C
ATOM	212	C	TYR	A	30	14.537	-10.604	7.852	1.00	24.37	C
ATOM	213	O	TYR	A	30	14.813	-11.198	8.911	1.00	26.54	O
ATOM	214	CB	TYR	A	30	12.785	-11.008	6.129	1.00	22.66	C
ATOM	215	CG	TYR	A	30	11.456	-11.702	6.058	1.00	24.59	C
ATOM	216	CD1	TYR	A	30	11.236	-12.867	6.775	1.00	26.93	C
ATOM	217	CD2	TYR	A	30	10.448	-11.251	5.218	1.00	25.02	C
ATOM	218	CE1	TYR	A	30	10.052	-13.584	6.651	1.00	27.16	C
ATOM	219	CE2	TYR	A	30	9.253	-11.953	5.082	1.00	26.54	C
ATOM	220	CZ	TYR	A	30	9.068	-13.128	5.798	1.00	27.87	C
ATOM	221	OH	TYR	A	30	7.931	-13.888	5.629	1.00	28.25	O
ATOM	222	N	LEU	A	31	15.469	-10.188	6.982	1.00	19.65	N
ATOM	223	CA	LEU	A	31	16.893	-10.427	7.227	1.00	16.82	C
ATOM	224	C	LEU	A	31	17.698	-9.262	7.838	1.00	16.70	C
ATOM	225	O	LEU	A	31	18.814	-9.467	8.332	1.00	16.93	O
ATOM	226	CB	LEU	A	31	17.569	-10.920	5.949	1.00	18.70	C
ATOM	227	CG	LEU	A	31	17.564	-12.423	5.628	1.00	18.34	C
ATOM	228	CD1	LEU	A	31	18.751	-12.798	4.728	1.00	16.70	C
ATOM	229	CD2	LEU	A	31	17.629	-13.188	6.913	1.00	17.85	C
ATOM	230	N	GLN	A	32	17.196	-8.040	7.703	1.00	12.99	N
ATOM	231	CA	GLN	A	32	17.563	-6.922	8.579	1.00	15.39	C
ATOM	232	C	GLN	A	32	19.015	-6.620	9.024	1.00	16.50	C
ATOM	233	O	GLN	A	32	19.233	-5.706	9.832	1.00	16.68	O
ATOM	234	CB	GLN	A	32	16.647	-6.914	9.830	1.00	17.32	C
ATOM	235	CG	GLN	A	32	16.825	-8.056	10.882	1.00	18.77	C
ATOM	236	CD	GLN	A	32	16.438	-7.610	12.302	1.00	19.47	C

ATOM	237	NE2	GLN	A	32	17.197	-8.054	13.301	1.00	11.37	N
ATOM	238	OE1	GLN	A	32	15.564	-6.759	12.468	1.00	21.08	O
ATOM	239	N	GLN	A	33	20.018	-7.295	8.474	1.00	17.10	N
ATOM	240	CA	GLN	A	33	21.399	-6.839	8.685	1.00	18.29	C
ATOM	241	C	GLN	A	33	22.190	-6.783	7.353	1.00	19.96	C
ATOM	242	O	GLN	A	33	22.912	-5.816	7.095	1.00	20.35	O
ATOM	243	CB	GLN	A	33	22.119	-7.705	9.752	1.00	16.98	C
ATOM	244	CG	GLN	A	33	21.184	-8.265	10.879	1.00	21.56	C
ATOM	245	CD	GLN	A	33	21.643	-7.989	12.350	1.00	23.80	C
ATOM	246	NE2	GLN	A	33	22.932	-7.685	12.533	1.00	26.50	N
ATOM	247	OE1	GLN	A	33	20.834	-8.040	13.295	1.00	19.44	O
ATOM	248	N	CYS	A	34	21.943	-7.745	6.458	1.00	20.58	N
ATOM	249	CA	CYS	A	34	22.517	-7.742	5.113	1.00	18.89	C
ATOM	250	C	CYS	A	34	22.254	-6.421	4.403	1.00	20.26	C
ATOM	251	O	CYS	A	34	21.094	-5.970	4.313	1.00	18.49	O
ATOM	252	CB	CYS	A	34	21.906	-8.857	4.277	1.00	17.31	C
ATOM	253	SG	CYS	A	34	22.155	-10.502	4.964	1.00	28.19	S
ATOM	254	N	PRO	A	35	23.309	-5.836	3.795	1.00	19.86	N
ATOM	255	CA	PRO	A	35	23.265	-4.593	3.019	1.00	16.08	C
ATOM	256	C	PRO	A	35	22.189	-4.451	1.981	1.00	13.85	C
ATOM	257	O	PRO	A	35	21.368	-5.324	1.779	1.00	11.96	O
ATOM	258	CB	PRO	A	35	24.648	-4.512	2.420	1.00	14.75	C
ATOM	259	CG	PRO	A	35	25.491	-4.991	3.508	1.00	21.60	C
ATOM	260	CD	PRO	A	35	24.692	-6.116	4.215	1.00	21.49	C
ATOM	261	N	PHE	A	36	22.110	-3.257	1.430	1.00	16.85	N
ATOM	262	CA	PHE	A	36	20.988	-2.926	0.602	1.00	17.73	C
ATOM	263	C	PHE	A	36	20.944	-3.782	-0.646	1.00	20.98	C
ATOM	264	O	PHE	A	36	20.009	-4.576	-0.789	1.00	24.39	O
ATOM	265	CB	PHE	A	36	20.979	-1.443	0.233	1.00	18.53	C
ATOM	266	CG	PHE	A	36	19.674	-0.996	-0.359	1.00	19.96	C
ATOM	267	CD1	PHE	A	36	18.482	-1.376	0.223	1.00	18.68	C
ATOM	268	CD2	PHE	A	36	19.632	-0.366	-1.579	1.00	20.99	C
ATOM	269	CE1	PHE	A	36	17.308	-1.164	-0.391	1.00	17.22	C
ATOM	270	CE2	PHE	A	36	18.443	-0.149	-2.195	1.00	19.46	C
ATOM	271	CZ	PHE	A	36	17.282	-0.559	-1.600	1.00	19.20	C
ATOM	272	N	GLU	A	37	21.990	-3.721	-1.482	1.00	21.10	N
ATOM	273	CA	GLU	A	37	21.895	-4.190	-2.874	1.00	20.88	C
ATOM	274	C	GLU	A	37	21.529	-5.668	-3.024	1.00	20.33	C
ATOM	275	O	GLU	A	37	20.863	-6.060	-3.979	1.00	18.29	O
ATOM	276	CB	GLU	A	37	23.192	-3.914	-3.617	1.00	22.94	C
ATOM	277	CG	GLU	A	37	23.564	-2.453	-3.675	1.00	24.70	C
ATOM	278	CD	GLU	A	37	24.725	-2.103	-2.759	1.00	27.37	C
ATOM	279	OE1	GLU	A	37	25.368	-3.042	-2.215	1.00	26.97	O
ATOM	280	OE2	GLU	A	37	24.966	-0.880	-2.567	1.00	25.74	O1-
ATOM	281	N	ASP	A	38	22.115	-6.488	-2.161	1.00	20.03	N
ATOM	282	CA	ASP	A	38	21.747	-7.891	-1.979	1.00	18.25	C
ATOM	283	C	ASP	A	38	20.224	-8.028	-1.946	1.00	20.19	C
ATOM	284	O	ASP	A	38	19.615	-8.679	-2.800	1.00	17.87	O
ATOM	285	CB	ASP	A	38	22.348	-8.378	-0.663	1.00	16.94	C
ATOM	286	CG	ASP	A	38	23.796	-7.902	-0.463	1.00	18.58	C
ATOM	287	OD1	ASP	A	38	24.047	-6.683	-0.383	1.00	14.38	O
ATOM	288	OD2	ASP	A	38	24.696	-8.758	-0.380	1.00	22.12	O1-
ATOM	289	N	HIS	A	39	19.612	-7.271	-1.038	1.00	23.52	N
ATOM	290	CA	HIS	A	39	18.160	-7.214	-0.882	1.00	22.04	C
ATOM	291	C	HIS	A	39	17.507	-6.768	-2.173	1.00	20.54	C
ATOM	292	O	HIS	A	39	16.482	-7.318	-2.558	1.00	21.75	O
ATOM	293	CB	HIS	A	39	17.765	-6.228	0.235	1.00	22.52	C
ATOM	294	CG	HIS	A	39	17.962	-6.765	1.616	1.00	21.90	C
ATOM	295	CD2	HIS	A	39	17.572	-7.930	2.183	1.00	22.42	C
ATOM	296	ND1	HIS	A	39	18.606	-6.054	2.609	1.00	23.78	N
ATOM	297	CE1	HIS	A	39	18.596	-6.759	3.729	1.00	21.46	C
ATOM	298	NE2	HIS	A	39	17.980	-7.900	3.492	1.00	22.35	N
ATOM	299	N	VAL	A	40	18.052	-5.725	-2.797	1.00	17.74	N
ATOM	300	CA	VAL	A	40	17.394	-5.125	-3.959	1.00	17.68	C
ATOM	301	C	VAL	A	40	17.509	-5.956	-5.264	1.00	21.04	C
ATOM	302	O	VAL	A	40	16.509	-6.136	-5.967	1.00	22.22	O
ATOM	303	CB	VAL	A	40	17.863	-3.667	-4.225	1.00	15.09	C
ATOM	304	CG1	VAL	A	40	16.681	-2.748	-4.220	1.00	15.66	C
ATOM	305	CG2	VAL	A	40	18.816	-3.204	-3.185	1.00	12.61	C
ATOM	306	N	LYS	A	41	18.664	-6.575	-5.518	1.00	21.82	N
ATOM	307	CA	LYS	A	41	18.789	-7.519	-6.630	1.00	21.46	C

```
ATOM    308  C   LYS A  41      17.644  -8.486  -6.523  1.00 21.01           C
ATOM    309  O   LYS A  41      16.769  -8.480  -7.376  1.00 23.65           O
ATOM    310  CB  LYS A  41      20.108  -8.290  -6.546  1.00 25.68           C
ATOM    311  CG  LYS A  41      20.620  -8.833  -7.888  1.00 27.49           C
ATOM    312  CD  LYS A  41      22.165  -8.736  -8.020  1.00 23.95           C
ATOM    313  CE  LYS A  41      22.573  -8.660  -9.504  1.00 21.47           C
ATOM    314  NZ  LYS A  41      23.966  -8.207  -9.722  1.00 16.15           N1+
ATOM    315  N   LEU A  42      17.495  -9.053  -5.324  1.00 21.14           N
ATOM    316  CA  LEU A  42      16.451 -10.045  -5.020  1.00 20.21           C
ATOM    317  C   LEU A  42      15.021  -9.526  -5.193  1.00 18.80           C
ATOM    318  O   LEU A  42      14.119 -10.252  -5.621  1.00 14.06           O
ATOM    319  CB  LEU A  42      16.653 -10.582  -3.604  1.00 20.68           C
ATOM    320  CG  LEU A  42      18.057 -11.154  -3.410  1.00 23.93           C
ATOM    321  CD1 LEU A  42      18.337 -11.363  -1.954  1.00 26.48           C
ATOM    322  CD2 LEU A  42      18.195 -12.468  -4.155  1.00 27.94           C
ATOM    323  N   VAL A  43      14.828  -8.248  -4.920  1.00 20.91           N
ATOM    324  CA  VAL A  43      13.557  -7.646  -5.243  1.00 25.09           C
ATOM    325  C   VAL A  43      13.455  -7.618  -6.756  1.00 24.80           C
ATOM    326  O   VAL A  43      12.655  -8.371  -7.319  1.00 24.61           O
ATOM    327  CB  VAL A  43      13.424  -6.220  -4.671  1.00 29.14           C
ATOM    328  CG1 VAL A  43      12.078  -5.628  -5.086  1.00 29.20           C
ATOM    329  CG2 VAL A  43      13.555  -6.252  -3.122  1.00 29.89           C
ATOM    330  N   ASN A  44      14.422  -6.952  -7.393  1.00 24.28           N
ATOM    331  CA  ASN A  44      14.472  -6.794  -8.849  1.00 24.67           C
ATOM    332  C   ASN A  44      13.983  -8.073  -9.527  1.00 25.13           C
ATOM    333  O   ASN A  44      13.160  -8.021 -10.443  1.00 27.80           O
ATOM    334  CB  ASN A  44      15.908  -6.461  -9.325  1.00 25.32           C
ATOM    335  CG  ASN A  44      16.357  -5.040  -8.947  1.00 29.48           C
ATOM    336  ND2 ASN A  44      15.803  -4.049  -9.631  1.00 31.83           N
ATOM    337  OD1 ASN A  44      17.192  -4.838  -8.052  1.00 30.17           O
ATOM    338  N   GLU A  45      14.326  -9.217  -8.942  1.00 24.11           N
ATOM    339  CA  GLU A  45      14.019 -10.503  -9.553  1.00 25.12           C
ATOM    340  C   GLU A  45      12.569 -10.992  -9.356  1.00 26.68           C
ATOM    341  O   GLU A  45      11.931 -11.464 -10.315  1.00 26.81           O
ATOM    342  CB  GLU A  45      15.015 -11.547  -9.043  1.00 22.72           C
ATOM    343  CG  GLU A  45      16.351 -11.537  -9.773  1.00 16.42           C
ATOM    344  CD  GLU A  45      17.486 -11.808  -8.848  1.00 12.41           C
ATOM    345  OE1 GLU A  45      17.454 -12.854  -8.182  1.00  8.42           O
ATOM    346  OE2 GLU A  45      18.353 -10.928  -8.705  1.00 12.61           O1-
ATOM    347  N   VAL A  46      12.105 -10.980  -8.100  1.00 28.78           N
ATOM    348  CA  VAL A  46      10.677 -11.141  -7.758  1.00 27.45           C
ATOM    349  C   VAL A  46       9.849 -10.270  -8.670  1.00 25.30           C
ATOM    350  O   VAL A  46       8.937 -10.754  -9.344  1.00 24.88           O
ATOM    351  CB  VAL A  46      10.352 -10.637  -6.332  1.00 28.88           C
ATOM    352  CG1 VAL A  46       8.928 -11.039  -5.958  1.00 28.73           C
ATOM    353  CG2 VAL A  46      11.381 -11.152  -5.327  1.00 30.37           C
ATOM    354  N   THR A  47      10.115  -8.966  -8.588  1.00 22.75           N
ATOM    355  CA  THR A  47       9.506  -7.984  -9.460  1.00 21.85           C
ATOM    356  C   THR A  47       9.418  -8.500 -10.909  1.00 24.34           C
ATOM    357  O   THR A  47       8.328  -8.429 -11.517  1.00 23.89           O
ATOM    358  CB  THR A  47      10.293  -6.653  -9.427  1.00 20.39           C
ATOM    359  CG2 THR A  47       9.430  -5.527  -9.894  1.00 22.88           C
ATOM    360  OG1 THR A  47      10.696  -6.351  -8.090  1.00 18.42           O
ATOM    361  N   GLU A  48      10.502  -9.131 -11.402  1.00 24.26           N
ATOM    362  CA  GLU A  48      10.540  -9.677 -12.778  1.00 25.92           C
ATOM    363  C   GLU A  48       9.432 -10.732 -13.024  1.00 26.47           C
ATOM    364  O   GLU A  48       8.254 -10.386 -13.267  1.00 21.91           O
ATOM    365  CB  GLU A  48      11.920 -10.285 -13.101  1.00 25.59           C
ATOM    366  CG  GLU A  48      12.994  -9.275 -13.584  1.00 34.29           C
ATOM    367  CD  GLU A  48      13.681  -9.665 -14.925  1.00 37.71           C
ATOM    368  OE1 GLU A  48      14.662 -10.459 -14.916  1.00 37.81           O
ATOM    369  OE2 GLU A  48      13.290  -9.109 -15.986  1.00 39.96           O1-
ATOM    370  N   PHE A  49       9.865 -11.982 -13.195  1.00 26.44           N
ATOM    371  CA  PHE A  49       9.041 -13.162 -12.909  1.00 26.68           C
ATOM    372  C   PHE A  49       7.528 -12.915 -12.813  1.00 27.60           C
ATOM    373  O   PHE A  49       6.732 -13.630 -13.444  1.00 27.93           O
ATOM    374  CB  PHE A  49       9.534 -13.796 -11.611  1.00 23.99           C
ATOM    375  CG  PHE A  49       9.256 -15.241 -11.510  1.00 22.41           C
ATOM    376  CD1 PHE A  49       8.042 -15.760 -11.905  1.00 25.30           C
ATOM    377  CD2 PHE A  49      10.177 -16.079 -10.948  1.00 23.15           C
ATOM    378  CE1 PHE A  49       7.754 -17.099 -11.725  1.00 26.44           C
```

```
ATOM    379  CE2 PHE A  49       9.893 -17.410 -10.766  1.00 26.07           C
ATOM    380  CZ  PHE A  49       8.682 -17.917 -11.151  1.00 23.64           C
ATOM    381  N   ALA A  50       7.147 -12.051 -11.869  1.00 28.68           N
ATOM    382  CA  ALA A  50       5.757 -11.693 -11.611  1.00 26.29           C
ATOM    383  C   ALA A  50       5.093 -11.370 -12.939  1.00 26.64           C
ATOM    384  O   ALA A  50       4.068 -11.963 -13.302  1.00 25.34           O
ATOM    385  CB  ALA A  50       5.717 -10.487 -10.704  1.00 22.98           C
ATOM    386  N   LYS A  51       5.773 -10.515 -13.703  1.00 26.55           N
ATOM    387  CA  LYS A  51       5.334 -10.065 -15.024  1.00 24.67           C
ATOM    388  C   LYS A  51       5.138 -11.225 -15.983  1.00 23.19           C
ATOM    389  O   LYS A  51       4.298 -11.140 -16.871  1.00 22.00           O
ATOM    390  CB  LYS A  51       6.366  -9.099 -15.636  1.00 25.82           C
ATOM    391  CG  LYS A  51       6.392  -7.692 -15.057  1.00 20.83           C
ATOM    392  CD  LYS A  51       7.753  -7.062 -15.237  1.00 15.07           C
ATOM    393  CE  LYS A  51       7.799  -5.778 -14.475  1.00 17.03           C
ATOM    394  NZ  LYS A  51       7.346  -5.991 -13.051  1.00 19.85           N1+
ATOM    395  N   THR A  52       5.942 -12.276 -15.844  1.00 20.21           N
ATOM    396  CA  THR A  52       5.860 -13.406 -16.750  1.00 20.84           C
ATOM    397  C   THR A  52       4.763 -14.438 -16.407  1.00 23.73           C
ATOM    398  O   THR A  52       4.255 -15.121 -17.310  1.00 25.44           O
ATOM    399  CB  THR A  52       7.233 -14.084 -16.902  1.00 18.84           C
ATOM    400  CG2 THR A  52       7.234 -15.510 -16.319  1.00 15.42           C
ATOM    401  OG1 THR A  52       7.585 -14.107 -18.292  1.00 18.38           O
ATOM    402  N   CYS A  53       4.305 -14.468 -15.154  1.00 24.15           N
ATOM    403  CA  CYS A  53       3.020 -15.113 -14.852  1.00 23.39           C
ATOM    404  C   CYS A  53       1.841 -14.134 -15.054  1.00 21.30           C
ATOM    405  O   CYS A  53       0.704 -14.552 -15.268  1.00 21.30           O
ATOM    406  CB  CYS A  53       3.000 -15.710 -13.428  1.00 24.03           C
ATOM    407  SG  CYS A  53       4.336 -16.896 -13.023  1.00 22.23           S
ATOM    408  N   VAL A  54       2.135 -12.843 -15.077  1.00 19.83           N
ATOM    409  CA  VAL A  54       1.222 -11.856 -15.647  1.00 23.85           C
ATOM    410  C   VAL A  54       0.895 -12.233 -17.099  1.00 24.27           C
ATOM    411  O   VAL A  54      -0.282 -12.282 -17.504  1.00 24.50           O
ATOM    412  CB  VAL A  54       1.865 -10.426 -15.619  1.00 26.97           C
ATOM    413  CG1 VAL A  54       1.309  -9.538 -16.746  1.00 26.12           C
ATOM    414  CG2 VAL A  54       1.638  -9.771 -14.250  1.00 30.36           C
ATOM    415  N   ALA A  55       1.952 -12.457 -17.881  1.00 22.66           N
ATOM    416  CA  ALA A  55       1.845 -12.830 -19.291  1.00 20.65           C
ATOM    417  C   ALA A  55       0.971 -14.066 -19.412  1.00 21.32           C
ATOM    418  O   ALA A  55       0.002 -14.111 -20.189  1.00 20.09           O
ATOM    419  CB  ALA A  55       3.235 -13.120 -19.850  1.00 18.03           C
ATOM    420  N   ASP A  56       1.236 -14.994 -18.498  1.00 22.03           N
ATOM    421  CA  ASP A  56       0.765 -16.364 -18.591  1.00 20.78           C
ATOM    422  C   ASP A  56       0.823 -16.974 -17.200  1.00 17.21           C
ATOM    423  O   ASP A  56       1.892 -17.299 -16.698  1.00 13.60           O
ATOM    424  CB  ASP A  56       1.667 -17.168 -19.555  1.00 21.11           C
ATOM    425  CG  ASP A  56       0.882 -17.885 -20.641  1.00 20.85           C
ATOM    426  OD1 ASP A  56      -0.136 -17.326 -21.131  1.00 18.50           O
ATOM    427  OD2 ASP A  56       1.283 -19.021 -20.979  1.00 19.93           O1-
ATOM    428  N   GLU A  57      -0.326 -17.055 -16.554  1.00 17.39           N
ATOM    429  CA  GLU A  57      -0.486 -17.952 -15.418  1.00 19.41           C
ATOM    430  C   GLU A  57       0.281 -19.246 -15.683  1.00 19.69           C
ATOM    431  O   GLU A  57       1.357 -19.437 -15.115  1.00 18.89           O
ATOM    432  CB  GLU A  57      -1.968 -18.291 -15.198  1.00 21.01           C
ATOM    433  CG  GLU A  57      -2.926 -17.181 -15.550  1.00 18.07           C
ATOM    434  CD  GLU A  57      -3.521 -16.521 -14.336  1.00 21.55           C
ATOM    435  OE1 GLU A  57      -2.782 -15.874 -13.551  1.00 18.91           O
ATOM    436  OE2 GLU A  57      -4.756 -16.620 -14.200  1.00 23.50           O1-
ATOM    437  N   SER A  58      -0.138 -19.944 -16.752  1.00 20.54           N
ATOM    438  CA  SER A  58       0.233 -21.343 -17.074  1.00 18.77           C
ATOM    439  C   SER A  58       1.734 -21.685 -17.200  1.00 16.17           C
ATOM    440  O   SER A  58       2.107 -22.848 -17.305  1.00 12.41           O
ATOM    441  CB  SER A  58      -0.468 -21.771 -18.367  1.00 19.91           C
ATOM    442  OG  SER A  58      -1.586 -20.943 -18.670  1.00 22.34           O
ATOM    443  N   ALA A  59       2.580 -20.666 -17.191  1.00 16.30           N
ATOM    444  CA  ALA A  59       4.000 -20.840 -17.363  1.00 19.02           C
ATOM    445  C   ALA A  59       4.585 -21.590 -16.177  1.00 21.95           C
ATOM    446  O   ALA A  59       4.252 -21.262 -15.033  1.00 23.97           O
ATOM    447  CB  ALA A  59       4.640 -19.489 -17.474  1.00 22.09           C
ATOM    448  N   GLU A  60       5.602 -22.419 -16.441  1.00 22.59           N
ATOM    449  CA  GLU A  60       6.267 -23.242 -15.417  1.00 22.93           C
```

ATOM	450	C	GLU	A	60	6.511	-22.498	-14.113	1.00	24.76	C
ATOM	451	O	GLU	A	60	6.901	-21.338	-14.122	1.00	28.75	O
ATOM	452	CB	GLU	A	60	7.611	-23.778	-15.934	1.00	19.81	C
ATOM	453	CG	GLU	A	60	8.417	-24.596	-14.918	1.00	16.82	C
ATOM	454	CD	GLU	A	60	9.732	-25.160	-15.474	1.00	17.21	C
ATOM	455	OE1	GLU	A	60	10.733	-24.414	-15.489	1.00	14.77	O
ATOM	456	OE2	GLU	A	60	9.794	-26.376	-15.792	1.00	16.15	O1-
ATOM	457	N	ASN	A	61	6.248	-23.175	-13.002	1.00	25.15	N
ATOM	458	CA	ASN	A	61	6.546	-22.712	-11.638	1.00	26.26	C
ATOM	459	C	ASN	A	61	5.873	-21.448	-11.121	1.00	26.29	C
ATOM	460	O	ASN	A	61	6.007	-21.124	-9.945	1.00	26.16	O
ATOM	461	CB	ASN	A	61	8.054	-22.619	-11.376	1.00	25.05	C
ATOM	462	CG	ASN	A	61	8.730	-21.612	-12.252	1.00	25.32	C
ATOM	463	ND2	ASN	A	61	8.313	-20.363	-12.155	1.00	21.35	N
ATOM	464	OD1	ASN	A	61	9.503	-21.985	-13.129	1.00	26.74	O
ATOM	465	N	CYS	A	62	5.005	-20.840	-11.915	1.00	27.22	N
ATOM	466	CA	CYS	A	62	4.054	-19.901	-11.338	1.00	27.20	C
ATOM	467	C	CYS	A	62	3.419	-20.547	-10.106	1.00	27.53	C
ATOM	468	O	CYS	A	62	3.560	-20.010	-9.012	1.00	27.96	O
ATOM	469	CB	CYS	A	62	3.001	-19.538	-12.379	1.00	27.41	C
ATOM	470	SG	CYS	A	62	3.731	-18.673	-13.817	1.00	27.27	S
ATOM	471	N	ASP	A	63	3.166	-21.853	-10.247	1.00	29.32	N
ATOM	472	CA	ASP	A	63	2.519	-22.729	-9.259	1.00	29.88	C
ATOM	473	C	ASP	A	63	3.127	-22.814	-7.853	1.00	31.82	C
ATOM	474	O	ASP	A	63	2.702	-23.660	-7.046	1.00	30.96	O
ATOM	475	CB	ASP	A	63	2.437	-24.142	-9.824	1.00	29.57	C
ATOM	476	CG	ASP	A	63	1.214	-24.355	-10.687	1.00	31.17	C
ATOM	477	OD1	ASP	A	63	1.082	-23.702	-11.758	1.00	32.77	O
ATOM	478	OD2	ASP	A	63	0.381	-25.200	-10.295	1.00	32.47	O1-
ATOM	479	N	LYS	A	64	4.183	-22.033	-7.600	1.00	33.23	N
ATOM	480	CA	LYS	A	64	4.695	-21.785	-6.244	1.00	30.18	C
ATOM	481	C	LYS	A	64	3.561	-21.200	-5.372	1.00	29.80	C
ATOM	482	O	LYS	A	64	2.803	-20.316	-5.817	1.00	28.83	O
ATOM	483	CB	LYS	A	64	5.864	-20.782	-6.308	1.00	27.37	C
ATOM	484	CG	LYS	A	64	7.257	-21.397	-6.361	1.00	25.95	C
ATOM	485	CD	LYS	A	64	7.352	-22.527	-7.386	1.00	27.90	C
ATOM	486	CE	LYS	A	64	8.191	-23.714	-6.889	1.00	28.98	C
ATOM	487	NZ	LYS	A	64	9.640	-23.392	-6.604	1.00	27.67	N1+
ATOM	488	N	SER	A	65	3.474	-21.647	-4.120	1.00	28.75	N
ATOM	489	CA	SER	A	65	2.650	-20.934	-3.136	1.00	27.25	C
ATOM	490	C	SER	A	65	3.419	-19.725	-2.546	1.00	25.40	C
ATOM	491	O	SER	A	65	4.196	-19.869	-1.583	1.00	21.94	O
ATOM	492	CB	SER	A	65	2.188	-21.897	-2.019	1.00	28.94	C
ATOM	493	OG	SER	A	65	0.824	-22.307	-2.198	1.00	26.21	O
ATOM	494	N	LEU	A	66	3.329	-18.597	-3.256	1.00	21.69	N
ATOM	495	CA	LEU	A	66	3.971	-17.330	-2.891	1.00	18.29	C
ATOM	496	C	LEU	A	66	5.229	-17.507	-2.069	1.00	20.23	C
ATOM	497	O	LEU	A	66	6.283	-17.683	-2.652	1.00	23.75	O
ATOM	498	CB	LEU	A	66	2.982	-16.401	-2.180	1.00	12.64	C
ATOM	499	CG	LEU	A	66	3.160	-14.880	-2.253	1.00	5.07	C
ATOM	500	CD1	LEU	A	66	4.592	-14.537	-2.380	1.00	2.40	C
ATOM	501	CD2	LEU	A	66	2.384	-14.296	-3.399	1.00	2.17	C
ATOM	502	N	HIS	A	67	5.083	-17.646	-0.749	1.00	23.72	N
ATOM	503	CA	HIS	A	67	6.208	-17.826	0.192	1.00	25.77	C
ATOM	504	C	HIS	A	67	7.266	-18.712	-0.428	1.00	27.03	C
ATOM	505	O	HIS	A	67	8.407	-18.292	-0.612	1.00	28.17	O
ATOM	506	CB	HIS	A	67	5.757	-18.505	1.505	1.00	27.30	C
ATOM	507	CG	HIS	A	67	5.053	-17.596	2.474	1.00	28.03	C
ATOM	508	CD2	HIS	A	67	5.372	-16.363	2.940	1.00	25.15	C
ATOM	509	ND1	HIS	A	67	3.955	-18.009	3.206	1.00	25.30	N
ATOM	510	CE1	HIS	A	67	3.645	-17.076	4.091	1.00	24.34	C
ATOM	511	NE2	HIS	A	67	4.488	-16.070	3.947	1.00	23.45	N
ATOM	512	N	THR	A	68	6.833	-19.877	-0.895	1.00	28.20	N
ATOM	513	CA	THR	A	68	7.749	-20.853	-1.467	1.00	31.25	C
ATOM	514	C	THR	A	68	8.754	-20.271	-2.461	1.00	32.00	C
ATOM	515	O	THR	A	68	9.905	-20.750	-2.549	1.00	31.36	O
ATOM	516	CB	THR	A	68	6.986	-21.972	-2.142	1.00	32.39	C
ATOM	517	CG2	THR	A	68	7.624	-23.327	-1.793	1.00	31.68	C
ATOM	518	OG1	THR	A	68	5.617	-21.933	-1.697	1.00	35.75	O
ATOM	519	N	LEU	A	69	8.353	-19.227	-3.185	1.00	31.87	N
ATOM	520	CA	LEU	A	69	9.340	-18.500	-3.974	1.00	33.65	C

ATOM	521	C	LEU	A	69	10.314	-17.880	-2.982	1.00	32.74	C
ATOM	522	O	LEU	A	69	11.288	-18.548	-2.631	1.00	32.06	O
ATOM	523	CB	LEU	A	69	8.705	-17.444	-4.905	1.00	34.67	C
ATOM	524	CG	LEU	A	69	9.426	-17.171	-6.259	1.00	34.79	C
ATOM	525	CD1	LEU	A	69	10.395	-15.967	-6.148	1.00	32.65	C
ATOM	526	CD2	LEU	A	69	10.174	-18.427	-6.738	1.00	31.64	C
ATOM	527	N	PHE	A	70	9.943	-16.747	-2.374	1.00	30.96	N
ATOM	528	CA	PHE	A	70	10.788	-16.080	-1.365	1.00	30.54	C
ATOM	529	C	PHE	A	70	11.691	-17.099	-0.646	1.00	29.74	C
ATOM	530	O	PHE	A	70	12.906	-17.097	-0.807	1.00	26.41	O
ATOM	531	CB	PHE	A	70	9.932	-15.387	-0.288	1.00	30.25	C
ATOM	532	CG	PHE	A	70	9.227	-14.125	-0.737	1.00	28.46	C
ATOM	533	CD1	PHE	A	70	8.468	-14.109	-1.899	1.00	26.39	C
ATOM	534	CD2	PHE	A	70	9.075	-13.072	0.168	1.00	24.40	C
ATOM	535	CE1	PHE	A	70	7.554	-13.084	-2.130	1.00	27.08	C
ATOM	536	CE2	PHE	A	70	8.165	-12.058	-0.054	1.00	21.72	C
ATOM	537	CZ	PHE	A	70	7.399	-12.062	-1.196	1.00	25.72	C
ATOM	538	N	GLY	A	71	11.043	-18.122	-0.104	1.00	31.24	N
ATOM	539	CA	GLY	A	71	11.712	-19.075	0.764	1.00	31.89	C
ATOM	540	C	GLY	A	71	12.873	-19.697	0.044	1.00	31.60	C
ATOM	541	O	GLY	A	71	14.004	-19.640	0.536	1.00	31.27	O
ATOM	542	N	ASP	A	72	12.614	-19.987	-1.227	1.00	33.37	N
ATOM	543	CA	ASP	A	72	13.555	-20.627	-2.154	1.00	33.34	C
ATOM	544	C	ASP	A	72	14.353	-19.663	-3.083	1.00	32.34	C
ATOM	545	O	ASP	A	72	15.121	-20.113	-3.946	1.00	30.19	O
ATOM	546	CB	ASP	A	72	12.767	-21.662	-2.979	1.00	33.35	C
ATOM	547	CG	ASP	A	72	12.165	-22.762	-2.099	1.00	31.88	C
ATOM	548	OD1	ASP	A	72	12.805	-23.100	-1.077	1.00	30.99	O
ATOM	549	OD2	ASP	A	72	11.059	-23.269	-2.406	1.00	29.43	O1-
ATOM	550	N	LYS	A	73	14.183	-18.352	-2.870	1.00	31.11	N
ATOM	551	CA	LYS	A	73	14.958	-17.288	-3.538	1.00	27.64	C
ATOM	552	C	LYS	A	73	15.199	-16.145	-2.533	1.00	26.15	C
ATOM	553	O	LYS	A	73	14.585	-15.081	-2.619	1.00	23.20	O
ATOM	554	CB	LYS	A	73	14.187	-16.738	-4.757	1.00	28.01	C
ATOM	555	CG	LYS	A	73	14.083	-17.691	-5.962	1.00	25.72	C
ATOM	556	CD	LYS	A	73	14.918	-17.203	-7.143	1.00	24.18	C
ATOM	557	CE	LYS	A	73	14.754	-15.712	-7.383	1.00	23.68	C
ATOM	558	NZ	LYS	A	73	16.079	-15.048	-7.529	1.00	22.67	N1+
ATOM	559	N	LEU	A	74	16.031	-16.423	-1.533	1.00	27.33	N
ATOM	560	CA	LEU	A	74	16.166	-15.602	-0.312	1.00	26.12	C
ATOM	561	C	LEU	A	74	17.355	-16.178	0.438	1.00	23.46	C
ATOM	562	O	LEU	A	74	18.398	-15.545	0.518	1.00	21.59	O
ATOM	563	CB	LEU	A	74	14.922	-15.728	0.593	1.00	26.77	C
ATOM	564	CG	LEU	A	74	14.153	-14.538	1.199	1.00	25.70	C
ATOM	565	CD1	LEU	A	74	12.799	-15.077	1.659	1.00	26.20	C
ATOM	566	CD2	LEU	A	74	14.879	-13.871	2.381	1.00	22.43	C
ATOM	567	N	CYS	A	75	17.225	-17.448	0.826	1.00	24.52	N
ATOM	568	CA	CYS	A	75	18.313	-18.240	1.395	1.00	26.88	C
ATOM	569	C	CYS	A	75	19.554	-18.212	0.475	1.00	28.33	C
ATOM	570	O	CYS	A	75	20.714	-18.148	0.939	1.00	26.66	O
ATOM	571	CB	CYS	A	75	17.830	-19.698	1.633	1.00	26.77	C
ATOM	572	SG	CYS	A	75	17.685	-20.118	3.425	1.00	27.16	S
ATOM	573	N	THR	A	76	19.283	-18.088	-0.822	1.00	30.35	N
ATOM	574	CA	THR	A	76	20.287	-18.225	-1.866	1.00	30.59	C
ATOM	575	C	THR	A	76	21.471	-17.261	-1.658	1.00	30.26	C
ATOM	576	O	THR	A	76	22.293	-17.437	-0.739	1.00	29.82	O
ATOM	577	CB	THR	A	76	19.623	-18.024	-3.284	1.00	32.29	C
ATOM	578	CG2	THR	A	76	20.023	-19.164	-4.228	1.00	32.43	C
ATOM	579	OG1	THR	A	76	18.179	-17.994	-3.171	1.00	31.05	O
ATOM	580	N	VAL	A	77	21.493	-16.179	-2.427	1.00	31.21	N
ATOM	581	CA	VAL	A	77	22.661	-15.299	-2.455	1.00	30.79	C
ATOM	582	C	VAL	A	77	22.468	-14.103	-1.514	1.00	32.91	C
ATOM	583	O	VAL	A	77	23.392	-13.312	-1.272	1.00	33.22	O
ATOM	584	CB	VAL	A	77	22.944	-14.813	-3.893	1.00	28.48	C
ATOM	585	CG1	VAL	A	77	22.227	-13.486	-4.176	1.00	25.60	C
ATOM	586	CG2	VAL	A	77	24.439	-14.718	-4.118	1.00	24.59	C
ATOM	587	N	ALA	A	78	21.257	-13.954	-0.987	1.00	33.04	N
ATOM	588	CA	ALA	A	78	21.110	-13.066	0.134	1.00	33.55	C
ATOM	589	C	ALA	A	78	22.005	-13.706	1.199	1.00	35.33	C
ATOM	590	O	ALA	A	78	23.151	-13.260	1.362	1.00	36.95	O
ATOM	591	CB	ALA	A	78	19.660	-13.008	0.576	1.00	32.27	C

ATOM	592	N	THR	A	79	21.683	-14.963	1.521	1.00	34.35	N
ATOM	593	CA	THR	A	79	22.100	-15.570	2.779	1.00	33.26	C
ATOM	594	C	THR	A	79	23.555	-16.077	2.835	1.00	32.95	C
ATOM	595	O	THR	A	79	24.250	-15.840	3.828	1.00	29.56	O
ATOM	596	CB	THR	A	79	21.140	-16.723	3.185	1.00	34.83	C
ATOM	597	CG2	THR	A	79	21.198	-16.977	4.704	1.00	35.95	C
ATOM	598	OG1	THR	A	79	19.791	-16.383	2.832	1.00	32.22	O
ATOM	599	N	LEU	A	80	24.027	-16.777	1.798	1.00	32.03	N
ATOM	600	CA	LEU	A	80	25.417	-17.297	1.808	1.00	28.26	C
ATOM	601	C	LEU	A	80	26.496	-16.225	1.630	1.00	27.39	C
ATOM	602	O	LEU	A	80	27.663	-16.471	1.914	1.00	27.30	O
ATOM	603	CB	LEU	A	80	25.609	-18.388	0.750	1.00	27.14	C
ATOM	604	CG	LEU	A	80	26.202	-18.027	-0.613	1.00	23.17	C
ATOM	605	CD1	LEU	A	80	27.191	-19.114	-1.025	1.00	23.29	C
ATOM	606	CD2	LEU	A	80	25.084	-17.888	-1.649	1.00	22.37	C
ATOM	607	N	ARG	A	81	26.117	-15.063	1.102	1.00	28.16	N
ATOM	608	CA	ARG	A	81	26.911	-13.843	1.282	1.00	28.08	C
ATOM	609	C	ARG	A	81	26.719	-13.293	2.706	1.00	29.33	C
ATOM	610	O	ARG	A	81	27.463	-12.415	3.169	1.00	28.86	O
ATOM	611	CB	ARG	A	81	26.477	-12.780	0.281	1.00	25.69	C
ATOM	612	CG	ARG	A	81	27.446	-11.627	0.186	1.00	24.75	C
ATOM	613	CD	ARG	A	81	27.337	-10.950	-1.152	1.00	25.26	C
ATOM	614	NE	ARG	A	81	27.236	-11.935	-2.211	1.00	28.70	N
ATOM	615	CZ	ARG	A	81	28.043	-12.988	-2.337	1.00	32.72	C
ATOM	616	NH1	ARG	A	81	29.145	-13.080	-1.599	1.00	31.48	N1+
ATOM	617	NH2	ARG	A	81	27.766	-13.933	-3.239	1.00	35.72	N
ATOM	618	N	GLU	A	82	25.660	-13.760	3.361	1.00	29.80	N
ATOM	619	CA	GLU	A	82	25.385	-13.405	4.745	1.00	30.13	C
ATOM	620	C	GLU	A	82	25.702	-14.591	5.709	1.00	28.95	C
ATOM	621	O	GLU	A	82	24.836	-15.378	6.068	1.00	25.40	O
ATOM	622	CB	GLU	A	82	23.930	-12.881	4.802	1.00	29.79	C
ATOM	623	CG	GLU	A	82	22.913	-13.604	5.673	1.00	28.95	C
ATOM	624	CD	GLU	A	82	22.631	-12.867	6.965	1.00	28.32	C
ATOM	625	OE1	GLU	A	82	23.152	-11.733	7.119	1.00	27.70	O
ATOM	626	OE2	GLU	A	82	21.883	-13.416	7.814	1.00	23.91	O1-
ATOM	627	N	THR	A	83	26.989	-14.795	5.996	1.00	29.92	N
ATOM	628	CA	THR	A	83	27.418	-16.000	6.718	1.00	32.38	C
ATOM	629	C	THR	A	83	28.141	-15.685	8.052	1.00	35.54	C
ATOM	630	O	THR	A	83	29.381	-15.602	8.095	1.00	37.17	O
ATOM	631	CB	THR	A	83	28.363	-16.937	5.826	1.00	29.73	C
ATOM	632	CG2	THR	A	83	27.562	-17.986	5.086	1.00	28.72	C
ATOM	633	OG1	THR	A	83	29.107	-16.168	4.865	1.00	28.72	O
ATOM	634	N	TYR	A	84	27.418	-15.611	9.171	1.00	36.72	N
ATOM	635	CA	TYR	A	84	28.134	-15.232	10.392	1.00	36.41	C
ATOM	636	C	TYR	A	84	27.787	-15.855	11.752	1.00	37.17	C
ATOM	637	O	TYR	A	84	28.696	-16.145	12.553	1.00	37.81	O
ATOM	638	CB	TYR	A	84	28.155	-13.719	10.504	1.00	32.67	C
ATOM	639	CG	TYR	A	84	29.547	-13.173	10.612	1.00	27.81	C
ATOM	640	CD1	TYR	A	84	30.321	-13.442	11.728	1.00	26.38	C
ATOM	641	CD2	TYR	A	84	30.000	-12.224	9.714	1.00	27.01	C
ATOM	642	CE1	TYR	A	84	31.467	-12.758	11.964	1.00	24.78	C
ATOM	643	CE2	TYR	A	84	31.147	-11.539	9.938	1.00	24.86	C
ATOM	644	CZ	TYR	A	84	31.865	-11.794	11.072	1.00	25.15	C
ATOM	645	OH	TYR	A	84	32.909	-10.976	11.395	1.00	27.73	O
ATOM	646	N	GLY	A	85	26.498	-15.954	12.073	1.00	37.02	N
ATOM	647	CA	GLY	A	85	26.123	-16.752	13.222	1.00	36.20	C
ATOM	648	C	GLY	A	85	25.033	-16.151	14.070	1.00	35.50	C
ATOM	649	O	GLY	A	85	25.291	-15.646	15.165	1.00	36.73	O
ATOM	650	N	GLU	A	86	23.816	-16.174	13.539	1.00	34.43	N
ATOM	651	CA	GLU	A	86	22.602	-16.106	14.366	1.00	31.00	C
ATOM	652	C	GLU	A	86	21.465	-16.802	13.613	1.00	28.33	C
ATOM	653	O	GLU	A	86	20.837	-17.735	14.124	1.00	28.62	O
ATOM	654	CB	GLU	A	86	22.220	-14.655	14.676	1.00	29.50	C
ATOM	655	CG	GLU	A	86	21.084	-14.522	15.646	1.00	25.84	C
ATOM	656	CD	GLU	A	86	21.097	-15.620	16.687	1.00	26.43	C
ATOM	657	OE1	GLU	A	86	21.992	-15.623	17.568	1.00	22.42	O
ATOM	658	OE2	GLU	A	86	20.223	-16.512	16.584	1.00	27.71	O1-
ATOM	659	N	MET	A	87	21.326	-16.474	12.340	1.00	24.32	N
ATOM	660	CA	MET	A	87	20.476	-17.275	11.494	1.00	24.03	C
ATOM	661	C	MET	A	87	21.175	-18.576	11.102	1.00	23.82	C
ATOM	662	O	MET	A	87	21.884	-18.629	10.092	1.00	22.28	O

ATOM	663	CB	MET	A	87	20.062	-16.483	10.266	1.00 22.69	C
ATOM	664	CG	MET	A	87	19.281	-15.254	10.625	1.00 21.49	C
ATOM	665	SD	MET	A	87	17.968	-15.060	9.479	1.00 25.30	S
ATOM	666	CE	MET	A	87	17.164	-13.558	10.089	1.00 25.45	C
ATOM	667	N	ALA	A	88	21.100	-19.558	12.007	1.00 24.16	N
ATOM	668	CA	ALA	A	88	21.551	-20.939	11.769	1.00 23.40	C
ATOM	669	C	ALA	A	88	20.674	-21.671	10.750	1.00 22.19	C
ATOM	670	O	ALA	A	88	20.034	-22.659	11.074	1.00 19.67	O
ATOM	671	CB	ALA	A	88	21.562	-21.707	13.092	1.00 23.53	C
ATOM	672	N	ASP	A	89	20.914	-21.344	9.489	1.00 24.31	N
ATOM	673	CA	ASP	A	89	19.989	-21.540	8.373	1.00 26.76	C
ATOM	674	C	ASP	A	89	18.712	-22.355	8.559	1.00 27.24	C
ATOM	675	O	ASP	A	89	18.747	-23.479	9.048	1.00 28.36	O
ATOM	676	CB	ASP	A	89	20.745	-22.081	7.155	1.00 29.44	C
ATOM	677	CG	ASP	A	89	20.619	-21.162	5.954	1.00 33.40	C
ATOM	678	OD1	ASP	A	89	21.358	-20.136	5.934	1.00 34.86	O
ATOM	679	OD2	ASP	A	89	19.716	-21.401	5.103	1.00 29.72	O1-
ATOM	680	N	CYS	A	90	17.732	-21.971	7.752	1.00 27.38	N
ATOM	681	CA	CYS	A	90	16.343	-22.307	8.008	1.00 26.95	C
ATOM	682	C	CYS	A	90	15.605	-22.522	6.664	1.00 26.81	C
ATOM	683	O	CYS	A	90	14.461	-22.077	6.485	1.00 28.42	O
ATOM	684	CB	CYS	A	90	15.719	-21.154	8.799	1.00 27.53	C
ATOM	685	SG	CYS	A	90	14.094	-21.429	9.556	1.00 25.39	S
ATOM	686	N	CYS	A	91	16.248	-23.192	5.708	1.00 24.62	N
ATOM	687	CA	CYS	A	91	15.559	-23.491	4.457	1.00 22.81	C
ATOM	688	C	CYS	A	91	15.315	-24.990	4.337	1.00 21.96	C
ATOM	689	O	CYS	A	91	14.563	-25.413	3.449	1.00 19.33	O
ATOM	690	CB	CYS	A	91	16.330	-22.934	3.255	1.00 24.46	C
ATOM	691	SG	CYS	A	91	17.922	-22.131	3.674	1.00 26.72	S
ATOM	692	N	ALA	A	92	15.547	-25.641	5.480	1.00 20.50	N
ATOM	693	CA	ALA	A	92	15.453	-27.100	5.702	1.00 21.22	C
ATOM	694	C	ALA	A	92	14.711	-27.984	4.682	1.00 24.74	C
ATOM	695	O	ALA	A	92	15.053	-27.983	3.494	1.00 24.03	O
ATOM	696	CB	ALA	A	92	14.900	-27.358	7.109	1.00 19.84	C
ATOM	697	N	LYS	A	93	13.819	-28.862	5.165	1.00 29.36	N
ATOM	698	CA	LYS	A	93	13.030	-29.749	4.272	1.00 32.05	C
ATOM	699	C	LYS	A	93	11.829	-28.986	3.713	1.00 32.90	C
ATOM	700	O	LYS	A	93	10.689	-29.355	4.021	1.00 32.86	O
ATOM	701	CB	LYS	A	93	12.505	-30.991	5.016	1.00 31.93	C
ATOM	702	CG	LYS	A	93	13.547	-31.775	5.787	1.00 33.86	C
ATOM	703	CD	LYS	A	93	13.897	-33.068	5.060	1.00 41.47	C
ATOM	704	CE	LYS	A	93	13.058	-34.327	5.515	1.00 43.89	C
ATOM	705	NZ	LYS	A	93	13.324	-35.551	4.649	1.00 41.62	N1+
ATOM	706	N	GLN	A	94	12.106	-27.847	3.058	1.00 34.28	N
ATOM	707	CA	GLN	A	94	11.143	-27.119	2.211	1.00 33.24	C
ATOM	708	C	GLN	A	94	9.733	-27.305	2.726	1.00 34.19	C
ATOM	709	O	GLN	A	94	9.042	-28.292	2.385	1.00 33.84	O
ATOM	710	CB	GLN	A	94	11.233	-27.588	0.762	1.00 31.80	C
ATOM	711	CG	GLN	A	94	10.790	-26.549	-0.236	1.00 34.94	C
ATOM	712	CD	GLN	A	94	11.426	-26.732	-1.637	1.00 38.30	C
ATOM	713	NE2	GLN	A	94	12.641	-26.177	-1.815	1.00 40.46	N
ATOM	714	OE1	GLN	A	94	10.825	-27.321	-2.563	1.00 32.88	O
ATOM	715	N	GLU	A	95	9.332	-26.345	3.554	1.00 32.90	N
ATOM	716	CA	GLU	A	95	8.335	-26.553	4.596	1.00 29.83	C
ATOM	717	C	GLU	A	95	7.742	-25.193	4.953	1.00 27.35	C
ATOM	718	O	GLU	A	95	8.463	-24.205	5.067	1.00 27.18	O
ATOM	719	CB	GLU	A	95	8.999	-27.179	5.835	1.00 29.20	C
ATOM	720	CG	GLU	A	95	10.428	-26.672	6.091	1.00 31.06	C
ATOM	721	CD	GLU	A	95	11.023	-27.126	7.434	1.00 31.73	C
ATOM	722	OE1	GLU	A	95	10.421	-28.009	8.097	1.00 33.07	O
ATOM	723	OE2	GLU	A	95	12.075	-26.563	7.821	1.00 28.41	O1-
ATOM	724	N	PRO	A	96	6.413	-25.117	5.082	1.00 26.21	N
ATOM	725	CA	PRO	A	96	5.812	-24.684	6.341	1.00 24.52	C
ATOM	726	C	PRO	A	96	6.808	-24.184	7.407	1.00 25.14	C
ATOM	727	O	PRO	A	96	7.050	-22.970	7.514	1.00 25.18	O
ATOM	728	CB	PRO	A	96	5.052	-25.927	6.769	1.00 25.77	C
ATOM	729	CG	PRO	A	96	4.695	-26.630	5.390	1.00 25.29	C
ATOM	730	CD	PRO	A	96	5.461	-25.931	4.308	1.00 22.87	C
ATOM	731	N	GLU	A	97	7.471	-25.114	8.099	1.00 25.87	N
ATOM	732	CA	GLU	A	97	8.490	-24.774	9.105	1.00 24.65	C
ATOM	733	C	GLU	A	97	9.746	-24.064	8.538	1.00 24.75	C

ATOM	734	O	GLU	A	97	10.841	-24.176	9.090	1.00	25.54	O
ATOM	735	CB	GLU	A	97	8.900	-26.035	9.903	1.00	22.10	C
ATOM	736	CG	GLU	A	97	9.205	-25.768	11.382	1.00	20.67	C
ATOM	737	CD	GLU	A	97	9.771	-26.971	12.146	1.00	21.89	C
ATOM	738	OE1	GLU	A	97	9.336	-28.118	11.890	1.00	20.02	O
ATOM	739	OE2	GLU	A	97	10.584	-26.752	13.080	1.00	21.31	O1-
ATOM	740	N	ARG	A	98	9.603	-23.344	7.433	1.00	25.09	N
ATOM	741	CA	ARG	A	98	10.673	-22.458	7.010	1.00	25.81	C
ATOM	742	C	ARG	A	98	10.317	-21.131	7.623	1.00	25.39	C
ATOM	743	O	ARG	A	98	10.978	-20.710	8.581	1.00	24.60	O
ATOM	744	CB	ARG	A	98	10.787	-22.358	5.466	1.00	28.52	C
ATOM	745	CG	ARG	A	98	10.015	-21.212	4.761	1.00	29.53	C
ATOM	746	CD	ARG	A	98	9.578	-21.625	3.364	1.00	32.14	C
ATOM	747	NE	ARG	A	98	8.122	-21.618	3.201	1.00	35.46	N
ATOM	748	CZ	ARG	A	98	7.473	-22.269	2.232	1.00	36.58	C
ATOM	749	NH1	ARG	A	98	8.153	-23.041	1.390	1.00	34.88	N1+
ATOM	750	NH2	ARG	A	98	6.140	-22.181	2.122	1.00	35.51	N
ATOM	751	N	ASN	A	99	9.118	-20.641	7.289	1.00	24.15	N
ATOM	752	CA	ASN	A	99	8.828	-19.225	7.492	1.00	23.31	C
ATOM	753	C	ASN	A	99	9.235	-18.901	8.910	1.00	22.87	C
ATOM	754	O	ASN	A	99	10.205	-18.165	9.103	1.00	20.69	O
ATOM	755	CB	ASN	A	99	7.347	-18.902	7.287	1.00	22.49	C
ATOM	756	CG	ASN	A	99	7.134	-17.493	6.738	1.00	24.38	C
ATOM	757	ND2	ASN	A	99	7.943	-16.540	7.206	1.00	21.20	N
ATOM	758	OD1	ASN	A	99	6.321	-17.287	5.821	1.00	28.44	O
ATOM	759	N	GLU	A	100	8.818	-19.821	9.785	1.00	21.65	N
ATOM	760	CA	GLU	A	100	8.975	-19.687	11.215	1.00	20.63	C
ATOM	761	C	GLU	A	100	10.448	-19.660	11.578	1.00	22.84	C
ATOM	762	O	GLU	A	100	11.049	-18.584	11.656	1.00	23.88	O
ATOM	763	CB	GLU	A	100	8.255	-20.822	11.950	1.00	16.75	C
ATOM	764	CG	GLU	A	100	6.754	-20.854	11.705	1.00	18.79	C
ATOM	765	CD	GLU	A	100	6.355	-21.788	10.556	1.00	24.05	C
ATOM	766	OE1	GLU	A	100	6.735	-22.979	10.603	1.00	23.63	O
ATOM	767	OE2	GLU	A	100	5.629	-21.357	9.622	1.00	26.06	O1-
ATOM	768	N	CYS	A	101	11.073	-20.826	11.570	1.00	26.26	N
ATOM	769	CA	CYS	A	101	12.183	-21.078	12.477	1.00	29.17	C
ATOM	770	C	CYS	A	101	13.170	-19.962	12.458	1.00	28.71	C
ATOM	771	O	CYS	A	101	13.708	-19.631	13.507	1.00	29.61	O
ATOM	772	CB	CYS	A	101	12.927	-22.363	12.140	1.00	31.73	C
ATOM	773	SG	CYS	A	101	14.563	-21.967	11.457	1.00	33.00	S
ATOM	774	N	PHE	A	102	13.459	-19.419	11.274	1.00	29.99	N
ATOM	775	CA	PHE	A	102	14.339	-18.251	11.208	1.00	30.99	C
ATOM	776	C	PHE	A	102	13.937	-17.084	10.309	1.00	30.01	C
ATOM	777	O	PHE	A	102	14.198	-15.929	10.646	1.00	31.09	O
ATOM	778	CB	PHE	A	102	15.794	-18.669	10.927	1.00	29.15	C
ATOM	779	CG	PHE	A	102	16.643	-18.701	12.165	1.00	31.69	C
ATOM	780	CD1	PHE	A	102	16.206	-18.063	13.325	1.00	31.15	C
ATOM	781	CD2	PHE	A	102	17.771	-19.508	12.236	1.00	33.88	C
ATOM	782	CE1	PHE	A	102	16.858	-18.244	14.537	1.00	33.52	C
ATOM	783	CE2	PHE	A	102	18.441	-19.698	13.451	1.00	34.28	C
ATOM	784	CZ	PHE	A	102	17.979	-19.066	14.605	1.00	35.49	C
ATOM	785	N	LEU	A	103	13.308	-17.360	9.174	1.00	29.36	N
ATOM	786	CA	LEU	A	103	13.003	-16.279	8.237	1.00	26.14	C
ATOM	787	C	LEU	A	103	11.972	-15.389	8.900	1.00	25.93	C
ATOM	788	O	LEU	A	103	12.147	-14.171	8.921	1.00	25.78	O
ATOM	789	CB	LEU	A	103	12.461	-16.815	6.906	1.00	19.74	C
ATOM	790	CG	LEU	A	103	13.228	-17.942	6.214	1.00	12.38	C
ATOM	791	CD1	LEU	A	103	12.638	-18.120	4.854	1.00	9.21	C
ATOM	792	CD2	LEU	A	103	14.710	-17.659	6.156	1.00	6.56	C
ATOM	793	N	GLN	A	104	11.085	-16.014	9.683	1.00	25.30	N
ATOM	794	CA	GLN	A	104	10.052	-15.283	10.414	1.00	25.11	C
ATOM	795	C	GLN	A	104	10.454	-14.979	11.849	1.00	25.04	C
ATOM	796	O	GLN	A	104	9.587	-14.855	12.719	1.00	24.08	O
ATOM	797	CB	GLN	A	104	8.742	-16.057	10.419	1.00	24.18	C
ATOM	798	CG	GLN	A	104	7.502	-15.185	10.263	1.00	26.71	C
ATOM	799	CD	GLN	A	104	6.727	-14.995	11.555	1.00	27.66	C
ATOM	800	NE2	GLN	A	104	5.873	-13.976	11.579	1.00	27.08	N
ATOM	801	OE1	GLN	A	104	6.884	-15.758	12.520	1.00	26.58	O
ATOM	802	N	HIS	A	105	11.771	-14.903	12.102	1.00	27.00	N
ATOM	803	CA	HIS	A	105	12.310	-14.502	13.416	1.00	24.47	C
ATOM	804	C	HIS	A	105	13.366	-13.402	13.432	1.00	21.96	C

ATOM	805	O	HIS	A	105	14.561	-13.626	13.636	1.00	18.43	O
ATOM	806	CB	HIS	A	105	12.745	-15.726	14.239	1.00	22.38	C
ATOM	807	CG	HIS	A	105	11.579	-16.471	14.785	1.00	21.89	C
ATOM	808	CD2	HIS	A	105	10.375	-16.007	15.196	1.00	21.60	C
ATOM	809	ND1	HIS	A	105	11.379	-17.806	14.479	1.00	20.64	N
ATOM	810	CE1	HIS	A	105	10.095	-18.100	14.625	1.00	22.23	C
ATOM	811	NE2	HIS	A	105	9.467	-17.022	15.048	1.00	23.35	N
ATOM	812	N	LYS	A	106	12.917	-12.304	12.860	1.00	19.95	N
ATOM	813	CA	LYS	A	106	12.919	-11.020	13.530	1.00	19.97	C
ATOM	814	C	LYS	A	106	13.352	-11.055	14.998	1.00	20.41	C
ATOM	815	O	LYS	A	106	12.569	-11.484	15.844	1.00	19.74	O
ATOM	816	CB	LYS	A	106	11.509	-10.434	13.441	1.00	18.97	C
ATOM	817	CG	LYS	A	106	10.855	-10.624	12.077	1.00	14.34	C
ATOM	818	CD	LYS	A	106	9.593	-9.822	11.956	1.00	12.34	C
ATOM	819	CE	LYS	A	106	8.917	-10.071	10.618	1.00	15.93	C
ATOM	820	NZ	LYS	A	106	7.629	-9.296	10.461	1.00	19.28	N1+
ATOM	821	N	ASP	A	107	14.647	-10.804	15.235	1.00	20.45	N
ATOM	822	CA	ASP	A	107	15.129	-10.023	16.386	1.00	19.31	C
ATOM	823	C	ASP	A	107	14.254	-8.775	16.470	1.00	20.32	C
ATOM	824	O	ASP	A	107	13.725	-8.318	15.464	1.00	18.89	O
ATOM	825	CB	ASP	A	107	16.582	-9.589	16.168	1.00	18.38	C
ATOM	826	CG	ASP	A	107	17.324	-9.293	17.468	1.00	20.19	C
ATOM	827	OD1	ASP	A	107	16.972	-8.328	18.186	1.00	20.89	O
ATOM	828	OD2	ASP	A	107	18.343	-9.964	17.709	1.00	20.24	O1-
ATOM	829	N	ASP	A	108	14.062	-8.248	17.673	1.00	22.88	N
ATOM	830	CA	ASP	A	108	12.971	-7.310	17.867	1.00	24.38	C
ATOM	831	C	ASP	A	108	13.419	-5.855	17.972	1.00	28.19	C
ATOM	832	O	ASP	A	108	13.842	-5.278	16.979	1.00	34.68	O
ATOM	833	CB	ASP	A	108	12.119	-7.724	19.066	1.00	21.46	C
ATOM	834	CG	ASP	A	108	10.665	-7.323	18.893	1.00	21.67	C
ATOM	835	OD1	ASP	A	108	10.407	-6.411	18.098	1.00	20.61	O
ATOM	836	OD2	ASP	A	108	9.792	-7.901	19.583	1.00	24.35	O1-
ATOM	837	N	ASN	A	109	13.182	-5.218	19.110	1.00	28.47	N
ATOM	838	CA	ASN	A	109	13.326	-3.763	19.264	1.00	28.03	C
ATOM	839	C	ASN	A	109	14.728	-3.298	18.833	1.00	28.47	C
ATOM	840	O	ASN	A	109	15.003	-3.159	17.631	1.00	29.63	O
ATOM	841	CB	ASN	A	109	13.100	-3.463	20.742	1.00	30.55	C
ATOM	842	CG	ASN	A	109	12.170	-2.307	20.987	1.00	30.28	C
ATOM	843	ND2	ASN	A	109	11.029	-2.303	20.315	1.00	34.20	N
ATOM	844	OD1	ASN	A	109	12.411	-1.514	21.893	1.00	28.96	O
ATOM	845	N	PRO	A	110	15.632	-3.006	19.797	1.00	27.35	N
ATOM	846	CA	PRO	A	110	16.964	-3.597	19.834	1.00	27.24	C
ATOM	847	C	PRO	A	110	17.241	-4.929	19.113	1.00	29.36	C
ATOM	848	O	PRO	A	110	17.484	-6.001	19.740	1.00	29.54	O
ATOM	849	CB	PRO	A	110	17.284	-3.559	21.317	1.00	25.13	C
ATOM	850	CG	PRO	A	110	16.640	-2.142	21.702	1.00	25.75	C
ATOM	851	CD	PRO	A	110	15.683	-1.751	20.558	1.00	24.19	C
ATOM	852	N	ASN	A	111	17.144	-4.835	17.781	1.00	27.97	N
ATOM	853	CA	ASN	A	111	18.078	-5.469	16.846	1.00	24.32	C
ATOM	854	C	ASN	A	111	19.394	-4.725	16.826	1.00	22.19	C
ATOM	855	O	ASN	A	111	19.509	-3.710	17.495	1.00	19.34	O
ATOM	856	CB	ASN	A	111	17.515	-5.455	15.440	1.00	21.73	C
ATOM	857	CG	ASN	A	111	16.979	-4.106	15.050	1.00	19.05	C
ATOM	858	ND2	ASN	A	111	17.263	-3.075	15.851	1.00	14.84	N
ATOM	859	OD1	ASN	A	111	16.299	-3.992	14.035	1.00	22.36	O
ATOM	860	N	LEU	A	112	20.147	-4.978	15.759	1.00	24.65	N
ATOM	861	CA	LEU	A	112	21.611	-4.907	15.800	1.00	26.73	C
ATOM	862	C	LEU	A	112	22.172	-3.568	15.277	1.00	26.67	C
ATOM	863	O	LEU	A	112	21.839	-2.531	15.854	1.00	23.61	O
ATOM	864	CB	LEU	A	112	22.185	-6.148	15.089	1.00	27.52	C
ATOM	865	CG	LEU	A	112	23.017	-7.089	15.972	1.00	31.09	C
ATOM	866	CD1	LEU	A	112	22.946	-8.557	15.481	1.00	26.90	C
ATOM	867	CD2	LEU	A	112	24.458	-6.546	16.003	1.00	29.65	C
ATOM	868	N	PRO	A	113	23.112	-3.565	14.288	1.00	29.08	N
ATOM	869	CA	PRO	A	113	23.734	-2.245	14.101	1.00	31.20	C
ATOM	870	C	PRO	A	113	22.679	-1.238	13.619	1.00	33.56	C
ATOM	871	O	PRO	A	113	21.661	-1.636	12.981	1.00	31.23	O
ATOM	872	CB	PRO	A	113	24.818	-2.486	13.030	1.00	29.19	C
ATOM	873	CG	PRO	A	113	24.576	-3.877	12.496	1.00	28.17	C
ATOM	874	CD	PRO	A	113	23.241	-4.343	13.047	1.00	29.64	C
ATOM	875	N	ARG	A	114	22.928	0.050	13.928	1.00	33.99	N

ATOM	876	CA	ARG	A	114	21.881	1.077	13.890	1.00	32.55	C
ATOM	877	C	ARG	A	114	22.292	2.540	13.957	1.00	32.21	C
ATOM	878	O	ARG	A	114	21.575	3.391	13.430	1.00	31.11	O
ATOM	879	CB	ARG	A	114	20.905	0.809	15.017	1.00	33.18	C
ATOM	880	CG	ARG	A	114	19.953	-0.281	14.661	1.00	36.64	C
ATOM	881	CD	ARG	A	114	19.020	-0.467	15.750	1.00	39.87	C
ATOM	882	NE	ARG	A	114	18.517	0.826	16.159	1.00	43.15	N
ATOM	883	CZ	ARG	A	114	17.756	0.977	17.222	1.00	47.64	C
ATOM	884	NH1	ARG	A	114	17.324	-0.119	17.852	1.00	51.30	N1+
ATOM	885	NH2	ARG	A	114	17.373	2.192	17.608	1.00	50.22	N
ATOM	886	N	LEU	A	115	23.284	2.864	14.788	1.00	33.02	N
ATOM	887	CA	LEU	A	115	23.491	4.243	15.202	1.00	33.18	C
ATOM	888	C	LEU	A	115	23.305	5.255	14.059	1.00	34.71	C
ATOM	889	O	LEU	A	115	22.386	6.065	14.145	1.00	35.24	O
ATOM	890	CB	LEU	A	115	24.860	4.423	15.860	1.00	32.34	C
ATOM	891	CG	LEU	A	115	25.037	5.442	17.017	1.00	31.68	C
ATOM	892	CD1	LEU	A	115	26.534	5.671	17.168	1.00	29.02	C
ATOM	893	CD2	LEU	A	115	24.324	6.786	16.764	1.00	26.36	C
ATOM	894	N	VAL	A	116	24.007	5.019	12.919	1.00	35.67	N
ATOM	895	CA	VAL	A	116	24.061	5.935	11.756	1.00	36.09	C
ATOM	896	C	VAL	A	116	22.692	6.478	11.298	1.00	37.34	C
ATOM	897	O	VAL	A	116	21.657	6.293	11.959	1.00	39.54	O
ATOM	898	CB	VAL	A	116	24.705	5.248	10.482	1.00	34.24	C
ATOM	899	CG1	VAL	A	116	26.241	4.937	10.734	1.00	33.93	C
ATOM	900	CG2	VAL	A	116	23.974	3.954	10.123	1.00	30.72	C
ATOM	901	N	ARG	A	117	22.732	7.203	10.185	1.00	37.72	N
ATOM	902	CA	ARG	A	117	21.486	7.644	9.511	1.00	40.29	C
ATOM	903	C	ARG	A	117	21.917	8.611	8.413	1.00	39.62	C
ATOM	904	O	ARG	A	117	23.104	8.596	8.037	1.00	41.35	O
ATOM	905	CB	ARG	A	117	20.547	8.386	10.490	1.00	42.16	C
ATOM	906	CG	ARG	A	117	19.116	7.763	10.656	1.00	46.39	C
ATOM	907	CD	ARG	A	117	18.704	7.899	12.134	1.00	51.79	C
ATOM	908	NE	ARG	A	117	19.092	9.229	12.637	1.00	56.27	N
ATOM	909	CZ	ARG	A	117	18.282	10.024	13.353	1.00	57.56	C
ATOM	910	NH1	ARG	A	117	18.086	11.271	12.915	1.00	57.16	N1+
ATOM	911	NH2	ARG	A	117	18.065	9.782	14.657	1.00	55.96	N
ATOM	912	N	PRO	A	118	20.982	9.415	7.863	1.00	37.83	N
ATOM	913	CA	PRO	A	118	21.179	10.738	7.262	1.00	38.11	C
ATOM	914	C	PRO	A	118	21.640	11.818	8.236	1.00	36.72	C
ATOM	915	O	PRO	A	118	21.590	11.611	9.463	1.00	38.18	O
ATOM	916	CB	PRO	A	118	19.774	11.075	6.712	1.00	38.53	C
ATOM	917	CG	PRO	A	118	18.892	10.088	7.386	1.00	38.63	C
ATOM	918	CD	PRO	A	118	19.697	8.870	7.443	1.00	37.42	C
ATOM	919	N	GLU	A	119	21.806	13.037	7.693	1.00	34.77	N
ATOM	920	CA	GLU	A	119	22.310	14.182	8.455	1.00	33.84	C
ATOM	921	C	GLU	A	119	22.333	15.588	7.791	1.00	31.31	C
ATOM	922	O	GLU	A	119	21.800	15.762	6.680	1.00	27.96	O
ATOM	923	CB	GLU	A	119	23.684	13.859	9.039	1.00	34.16	C
ATOM	924	CG	GLU	A	119	23.650	13.731	10.573	1.00	29.57	C
ATOM	925	CD	GLU	A	119	23.678	15.084	11.263	1.00	27.59	C
ATOM	926	OE1	GLU	A	119	24.804	15.594	11.417	1.00	25.30	O
ATOM	927	OE2	GLU	A	119	22.602	15.581	11.689	1.00	21.70	O1-
ATOM	928	N	VAL	A	120	23.119	16.516	8.368	1.00	29.09	N
ATOM	929	CA	VAL	A	120	22.754	17.938	8.468	1.00	27.70	C
ATOM	930	C	VAL	A	120	21.995	18.387	7.264	1.00	27.84	C
ATOM	931	O	VAL	A	120	20.779	18.514	7.318	1.00	29.90	O
ATOM	932	CB	VAL	A	120	23.973	18.925	8.622	1.00	27.28	C
ATOM	933	CG1	VAL	A	120	23.965	19.580	10.005	1.00	25.95	C
ATOM	934	CG2	VAL	A	120	25.309	18.221	8.353	1.00	27.55	C
ATOM	935	N	ASP	A	121	22.688	18.398	6.131	1.00	28.02	N
ATOM	936	CA	ASP	A	121	22.234	19.108	4.946	1.00	26.82	C
ATOM	937	C	ASP	A	121	21.939	18.197	3.749	1.00	25.21	C
ATOM	938	O	ASP	A	121	21.035	18.483	2.953	1.00	23.24	O
ATOM	939	CB	ASP	A	121	23.247	20.212	4.560	1.00	25.99	C
ATOM	940	CG	ASP	A	121	24.676	19.899	5.019	1.00	26.35	C
ATOM	941	OD1	ASP	A	121	25.271	18.950	4.459	1.00	27.73	O
ATOM	942	OD2	ASP	A	121	25.210	20.583	5.923	1.00	21.53	O1-
ATOM	943	N	VAL	A	122	22.591	17.041	3.682	1.00	23.49	N
ATOM	944	CA	VAL	A	122	22.769	16.383	2.386	1.00	25.51	C
ATOM	945	C	VAL	A	122	21.644	15.468	1.927	1.00	26.41	C
ATOM	946	O	VAL	A	122	21.527	15.180	0.732	1.00	25.53	O

ATOM	947	CB	VAL	A	122	24.124	15.616	2.299	1.00	25.87	C
ATOM	948	CG1	VAL	A	122	24.962	15.857	3.561	1.00	27.67	C
ATOM	949	CG2	VAL	A	122	23.896	14.114	2.031	1.00	25.34	C
ATOM	950	N	MET	A	123	20.814	15.019	2.867	1.00	29.60	N
ATOM	951	CA	MET	A	123	19.863	13.932	2.601	1.00	30.15	C
ATOM	952	C	MET	A	123	18.835	14.384	1.578	1.00	32.67	C
ATOM	953	O	MET	A	123	18.100	13.549	1.034	1.00	33.77	O
ATOM	954	CB	MET	A	123	19.148	13.469	3.889	1.00	27.54	C
ATOM	955	CG	MET	A	123	17.910	14.282	4.295	1.00	25.66	C
ATOM	956	SD	MET	A	123	18.202	15.511	5.615	1.00	22.74	S
ATOM	957	CE	MET	A	123	16.615	16.318	5.641	1.00	22.28	C
ATOM	958	N	CYS	A	124	18.756	15.707	1.361	1.00	32.18	N
ATOM	959	CA	CYS	A	124	17.895	16.302	0.328	1.00	29.82	C
ATOM	960	C	CYS	A	124	18.423	15.917	-1.060	1.00	27.70	C
ATOM	961	O	CYS	A	124	19.457	16.417	-1.503	1.00	27.67	O
ATOM	962	CB	CYS	A	124	17.879	17.819	0.455	1.00	27.21	C
ATOM	963	SG	CYS	A	124	17.768	18.608	-1.174	1.00	27.90	S
ATOM	964	N	ALA	A	126	16.357	14.257	-3.975	1.00	22.43	N
ATOM	965	CA	ALA	A	126	15.531	13.862	-5.118	1.00	26.10	C
ATOM	966	C	ALA	A	126	16.012	14.663	-6.335	1.00	27.83	C
ATOM	967	O	ALA	A	126	15.423	15.699	-6.716	1.00	28.31	O
ATOM	968	CB	ALA	A	126	14.054	14.116	-4.848	1.00	27.88	C
ATOM	969	N	PHE	A	127	17.206	14.284	-6.793	1.00	27.19	N
ATOM	970	CA	PHE	A	127	17.877	14.923	-7.916	1.00	25.57	C
ATOM	971	C	PHE	A	127	18.309	13.827	-8.896	1.00	25.18	C
ATOM	972	O	PHE	A	127	17.915	13.833	-10.062	1.00	24.40	O
ATOM	973	CB	PHE	A	127	19.107	15.705	-7.422	1.00	25.50	C
ATOM	974	CG	PHE	A	127	18.819	16.683	-6.298	1.00	24.31	C
ATOM	975	CD1	PHE	A	127	17.616	17.361	-6.224	1.00	23.10	C
ATOM	976	CD2	PHE	A	127	19.761	16.911	-5.307	1.00	24.98	C
ATOM	977	CE1	PHE	A	127	17.356	18.229	-5.169	1.00	24.24	C
ATOM	978	CE2	PHE	A	127	19.507	17.792	-4.260	1.00	23.55	C
ATOM	979	CZ	PHE	A	127	18.304	18.442	-4.188	1.00	22.96	C
ATOM	980	N	HIS	A	128	19.093	12.872	-8.395	1.00	23.97	N
ATOM	981	CA	HIS	A	128	19.603	11.748	-9.190	1.00	25.06	C
ATOM	982	C	HIS	A	128	19.666	10.507	-8.299	1.00	25.79	C
ATOM	983	O	HIS	A	128	20.736	9.885	-8.155	1.00	24.46	O
ATOM	984	CB	HIS	A	128	21.006	12.060	-9.762	1.00	26.48	C
ATOM	985	CG	HIS	A	128	21.630	10.926	-10.528	1.00	26.14	C
ATOM	986	CD2	HIS	A	128	21.599	10.603	-11.842	1.00	26.98	C
ATOM	987	ND1	HIS	A	128	22.404	9.955	-9.919	1.00	25.60	N
ATOM	988	CE1	HIS	A	128	22.814	9.086	-10.824	1.00	25.56	C
ATOM	989	NE2	HIS	A	128	22.341	9.456	-11.999	1.00	26.11	N
ATOM	990	N	ASP	A	129	18.631	10.376	-7.473	1.00	25.20	N
ATOM	991	CA	ASP	A	129	18.171	9.095	-6.944	1.00	23.48	C
ATOM	992	C	ASP	A	129	17.157	9.345	-5.843	1.00	24.69	C
ATOM	993	O	ASP	A	129	17.123	10.429	-5.245	1.00	22.84	O
ATOM	994	CB	ASP	A	129	19.312	8.207	-6.401	1.00	22.35	C
ATOM	995	CG	ASP	A	129	20.159	8.879	-5.310	1.00	19.90	C
ATOM	996	OD1	ASP	A	129	19.796	9.960	-4.801	1.00	16.62	O
ATOM	997	OD2	ASP	A	129	21.216	8.304	-4.963	1.00	18.41	O1-
ATOM	998	N	GLU	A	131	16.294	6.107	-4.845	1.00	26.76	N
ATOM	999	CA	GLU	A	131	16.936	4.947	-4.238	1.00	26.85	C
ATOM	1000	C	GLU	A	131	17.501	5.427	-2.883	1.00	26.10	C
ATOM	1001	O	GLU	A	131	17.117	4.893	-1.861	1.00	25.45	O
ATOM	1002	CB	GLU	A	131	18.035	4.384	-5.180	1.00	25.89	C
ATOM	1003	CG	GLU	A	131	17.551	3.445	-6.329	1.00	21.04	C
ATOM	1004	CD	GLU	A	131	16.340	3.971	-7.147	1.00	22.14	C
ATOM	1005	OE1	GLU	A	131	16.533	4.847	-8.019	1.00	19.96	O
ATOM	1006	OE2	GLU	A	131	15.222	3.409	-7.018	1.00	19.19	O1-
ATOM	1007	N	GLU	A	132	18.176	6.583	-2.862	1.00	27.14	N
ATOM	1008	CA	GLU	A	132	18.411	7.302	-1.593	1.00	27.48	C
ATOM	1009	C	GLU	A	132	17.082	7.842	-1.037	1.00	28.11	C
ATOM	1010	O	GLU	A	132	16.106	7.962	-1.754	1.00	24.38	O
ATOM	1011	CB	GLU	A	132	19.392	8.487	-1.758	1.00	26.09	C
ATOM	1012	CG	GLU	A	132	20.903	8.155	-1.682	1.00	25.28	C
ATOM	1013	CD	GLU	A	132	21.435	7.896	-0.264	1.00	21.57	C
ATOM	1014	OE1	GLU	A	132	21.255	6.778	0.262	1.00	19.27	O
ATOM	1015	OE2	GLU	A	132	22.139	8.767	0.272	1.00	15.77	O1-
ATOM	1016	N	THR	A	133	17.137	8.383	0.170	1.00	30.85	N
ATOM	1017	CA	THR	A	133	15.943	8.621	0.974	1.00	33.25	C

ATOM	1018	C	THR	A	133	14.702	7.774	0.628	1.00	34.75	C
ATOM	1019	O	THR	A	133	14.606	6.619	1.090	1.00	36.23	O
ATOM	1020	CB	THR	A	133	15.615	10.107	0.979	1.00	34.23	C
ATOM	1021	CG2	THR	A	133	16.767	10.868	1.680	1.00	36.08	C
ATOM	1022	OG1	THR	A	133	15.497	10.586	-0.370	1.00	35.21	O
ATOM	1023	N	PHE	A	134	13.798	8.301	-0.209	1.00	34.14	N
ATOM	1024	CA	PHE	A	134	12.474	7.689	-0.444	1.00	32.44	C
ATOM	1025	C	PHE	A	134	12.485	6.162	-0.360	1.00	32.71	C
ATOM	1026	O	PHE	A	134	11.952	5.604	0.584	1.00	35.71	O
ATOM	1027	CB	PHE	A	134	11.886	8.175	-1.787	1.00	30.94	C
ATOM	1028	CG	PHE	A	134	11.252	7.086	-2.630	1.00	29.00	C
ATOM	1029	CD1	PHE	A	134	10.070	6.474	-2.245	1.00	30.88	C
ATOM	1030	CD2	PHE	A	134	11.853	6.672	-3.805	1.00	28.45	C
ATOM	1031	CE1	PHE	A	134	9.508	5.473	-3.015	1.00	29.95	C
ATOM	1032	CE2	PHE	A	134	11.302	5.674	-4.585	1.00	28.90	C
ATOM	1033	CZ	PHE	A	134	10.132	5.074	-4.191	1.00	31.63	C
ATOM	1034	N	LEU	A	135	13.227	5.508	-1.242	1.00	32.49	N
ATOM	1035	CA	LEU	A	135	13.400	4.061	-1.193	1.00	32.55	C
ATOM	1036	C	LEU	A	135	14.020	3.666	0.150	1.00	32.40	C
ATOM	1037	O	LEU	A	135	13.384	2.984	0.946	1.00	33.86	O
ATOM	1038	CB	LEU	A	135	14.295	3.623	-2.362	1.00	34.15	C
ATOM	1039	CG	LEU	A	135	14.898	2.211	-2.532	1.00	37.12	C
ATOM	1040	CD1	LEU	A	135	14.673	1.676	-3.994	1.00	35.21	C
ATOM	1041	CD2	LEU	A	135	16.396	2.278	-2.232	1.00	33.42	C
ATOM	1042	N	LYS	A	136	15.169	4.258	0.477	1.00	32.96	N
ATOM	1043	CA	LYS	A	136	15.967	3.890	1.662	1.00	31.27	C
ATOM	1044	C	LYS	A	136	15.382	4.437	2.974	1.00	30.10	C
ATOM	1045	O	LYS	A	136	16.095	4.623	3.975	1.00	27.30	O
ATOM	1046	CB	LYS	A	136	17.429	4.370	1.501	1.00	30.80	C
ATOM	1047	CG	LYS	A	136	18.317	3.488	0.613	1.00	25.87	C
ATOM	1048	CD	LYS	A	136	19.724	4.099	0.430	1.00	25.64	C
ATOM	1049	CE	LYS	A	136	20.846	3.309	1.182	1.00	21.64	C
ATOM	1050	NZ	LYS	A	136	22.219	3.921	1.063	1.00	11.74	N1+
ATOM	1051	N	LYS	A	137	14.073	4.642	2.986	1.00	28.98	N
ATOM	1052	CA	LYS	A	137	13.340	4.610	4.251	1.00	27.29	C
ATOM	1053	C	LYS	A	137	12.719	3.231	4.562	1.00	25.01	C
ATOM	1054	O	LYS	A	137	11.769	3.148	5.343	1.00	19.88	O
ATOM	1055	CB	LYS	A	137	12.267	5.703	4.278	1.00	27.89	C
ATOM	1056	CG	LYS	A	137	11.043	5.458	3.424	1.00	25.43	C
ATOM	1057	CD	LYS	A	137	10.013	6.549	3.657	1.00	24.61	C
ATOM	1058	CE	LYS	A	137	9.532	6.581	5.121	1.00	25.80	C
ATOM	1059	NZ	LYS	A	137	10.478	7.205	6.080	1.00	22.10	N1+
ATOM	1060	N	TYR	A	138	13.261	2.170	3.944	1.00	23.60	N
ATOM	1061	CA	TYR	A	138	12.987	0.797	4.367	1.00	23.57	C
ATOM	1062	C	TYR	A	138	14.201	-0.014	4.898	1.00	25.14	C
ATOM	1063	O	TYR	A	138	14.157	-0.518	6.019	1.00	26.51	O
ATOM	1064	CB	TYR	A	138	12.287	0.013	3.262	1.00	23.90	C
ATOM	1065	CG	TYR	A	138	11.095	0.708	2.626	1.00	23.12	C
ATOM	1066	CD1	TYR	A	138	10.272	1.532	3.375	1.00	21.31	C
ATOM	1067	CD2	TYR	A	138	10.950	0.725	1.238	1.00	20.04	C
ATOM	1068	CE1	TYR	A	138	9.396	2.388	2.764	1.00	21.87	C
ATOM	1069	CE2	TYR	A	138	10.096	1.584	0.627	1.00	18.89	C
ATOM	1070	CZ	TYR	A	138	9.347	2.451	1.385	1.00	19.37	C
ATOM	1071	OH	TYR	A	138	8.863	3.599	0.796	1.00	16.36	O
ATOM	1072	N	LEU	A	139	15.348	-0.004	4.229	1.00	26.31	N
ATOM	1073	CA	LEU	A	139	16.544	-0.492	4.932	1.00	28.30	C
ATOM	1074	C	LEU	A	139	16.903	0.423	6.137	1.00	30.40	C
ATOM	1075	O	LEU	A	139	18.050	0.459	6.603	1.00	33.77	O
ATOM	1076	CB	LEU	A	139	17.764	-0.643	3.987	1.00	23.98	C
ATOM	1077	CG	LEU	A	139	18.987	-1.356	4.621	1.00	21.06	C
ATOM	1078	CD1	LEU	A	139	19.161	-2.769	4.075	1.00	15.84	C
ATOM	1079	CD2	LEU	A	139	20.249	-0.513	4.429	1.00	18.03	C
ATOM	1080	N	TYR	A	140	15.914	1.125	6.676	1.00	28.28	N
ATOM	1081	CA	TYR	A	140	16.160	2.032	7.782	1.00	27.48	C
ATOM	1082	C	TYR	A	140	14.903	2.160	8.640	1.00	26.31	C
ATOM	1083	O	TYR	A	140	14.644	1.316	9.484	1.00	25.70	O
ATOM	1084	CB	TYR	A	140	16.555	3.370	7.196	1.00	29.37	C
ATOM	1085	CG	TYR	A	140	18.029	3.568	7.070	1.00	32.07	C
ATOM	1086	CD1	TYR	A	140	18.907	2.754	7.774	1.00	32.26	C
ATOM	1087	CD2	TYR	A	140	18.511	4.785	6.615	1.00	35.07	C
ATOM	1088	CE1	TYR	A	140	20.189	3.177	8.085	1.00	31.31	C

ATOM	1089	CE2	TYR	A	140	19.788	5.220	6.938	1.00	34.45	C
ATOM	1090	CZ	TYR	A	140	20.604	4.429	7.691	1.00	30.17	C
ATOM	1091	OH	TYR	A	140	21.706	5.009	8.252	1.00	30.52	O
ATOM	1092	N	GLU	A	141	13.993	3.007	8.184	1.00	27.43	N
ATOM	1093	CA	GLU	A	141	12.635	3.080	8.702	1.00	26.66	C
ATOM	1094	C	GLU	A	141	11.830	1.793	8.474	1.00	25.94	C
ATOM	1095	O	GLU	A	141	10.589	1.841	8.394	1.00	24.42	O
ATOM	1096	CB	GLU	A	141	11.920	4.269	8.058	1.00	27.66	C
ATOM	1097	CG	GLU	A	141	12.753	5.571	8.070	1.00	28.85	C
ATOM	1098	CD	GLU	A	141	12.126	6.665	8.930	1.00	30.04	C
ATOM	1099	OE1	GLU	A	141	12.059	6.463	10.169	1.00	28.05	O
ATOM	1100	OE2	GLU	A	141	11.684	7.706	8.368	1.00	27.92	O1-
ATOM	1101	N	ILE	A	142	12.543	0.668	8.301	1.00	25.02	N
ATOM	1102	CA	ILE	A	142	11.959	-0.670	8.411	1.00	23.92	C
ATOM	1103	C	ILE	A	142	12.891	-1.879	8.483	1.00	22.66	C
ATOM	1104	O	ILE	A	142	12.422	-3.019	8.460	1.00	21.14	O
ATOM	1105	CB	ILE	A	142	10.920	-0.960	7.307	1.00	25.56	C
ATOM	1106	CG1	ILE	A	142	9.839	-1.855	7.893	1.00	31.26	C
ATOM	1107	CG2	ILE	A	142	11.533	-1.747	6.136	1.00	26.16	C
ATOM	1108	CD1	ILE	A	142	9.808	-1.835	9.391	1.00	32.34	C
ATOM	1109	N	ALA	A	143	14.192	-1.655	8.574	1.00	21.53	N
ATOM	1110	CA	ALA	A	143	15.080	-2.739	8.995	1.00	21.28	C
ATOM	1111	C	ALA	A	143	15.259	-2.555	10.491	1.00	21.40	C
ATOM	1112	O	ALA	A	143	15.077	-3.478	11.295	1.00	20.55	O
ATOM	1113	CB	ALA	A	143	16.437	-2.637	8.279	1.00	20.32	C
ATOM	1114	N	ARG	A	144	15.496	-1.300	10.850	1.00	21.00	N
ATOM	1115	CA	ARG	A	144	15.726	-0.906	12.216	1.00	21.33	C
ATOM	1116	C	ARG	A	144	14.498	-1.163	13.053	1.00	21.04	C
ATOM	1117	O	ARG	A	144	13.397	-0.738	12.694	1.00	20.32	O
ATOM	1118	CB	ARG	A	144	16.052	0.577	12.271	1.00	21.01	C
ATOM	1119	CG	ARG	A	144	17.510	0.863	12.247	1.00	17.71	C
ATOM	1120	CD	ARG	A	144	18.023	1.041	10.857	1.00	17.22	C
ATOM	1121	NE	ARG	A	144	18.645	2.342	10.630	1.00	18.81	N
ATOM	1122	CZ	ARG	A	144	19.496	2.950	11.447	1.00	17.78	C
ATOM	1123	NH1	ARG	A	144	19.398	2.791	12.749	1.00	22.32	N1+
ATOM	1124	NH2	ARG	A	144	20.194	3.978	11.001	1.00	19.08	N
ATOM	1125	N	ARG	A	145	14.686	-1.933	14.117	1.00	22.67	N
ATOM	1126	CA	ARG	A	145	13.838	-1.888	15.297	1.00	22.80	C
ATOM	1127	C	ARG	A	145	12.341	-1.995	14.978	1.00	23.74	C
ATOM	1128	O	ARG	A	145	11.492	-1.697	15.827	1.00	24.29	O
ATOM	1129	CB	ARG	A	145	14.150	-0.595	16.060	1.00	23.74	C
ATOM	1130	CG	ARG	A	145	13.284	0.605	15.663	1.00	24.56	C
ATOM	1131	CD	ARG	A	145	14.041	1.882	15.206	1.00	23.54	C
ATOM	1132	NE	ARG	A	145	13.036	2.868	14.811	1.00	23.80	N
ATOM	1133	CZ	ARG	A	145	13.214	3.859	13.954	1.00	24.48	C
ATOM	1134	NH1	ARG	A	145	14.272	4.656	14.069	1.00	24.11	N1+
ATOM	1135	NH2	ARG	A	145	12.154	4.288	13.278	1.00	24.06	N
ATOM	1136	N	HIS	A	146	12.021	-2.380	13.739	1.00	23.24	N
ATOM	1137	CA	HIS	A	146	10.633	-2.505	13.276	1.00	21.90	C
ATOM	1138	C	HIS	A	146	10.413	-3.719	12.380	1.00	19.01	C
ATOM	1139	O	HIS	A	146	9.649	-3.649	11.433	1.00	15.22	O
ATOM	1140	CB	HIS	A	146	10.196	-1.244	12.519	1.00	21.14	C
ATOM	1141	CG	HIS	A	146	10.226	-0.013	13.357	1.00	23.07	C
ATOM	1142	CD2	HIS	A	146	10.883	1.158	13.170	1.00	24.02	C
ATOM	1143	ND1	HIS	A	146	9.778	0.000	14.658	1.00	21.23	N
ATOM	1144	CE1	HIS	A	146	10.178	1.100	15.251	1.00	24.29	C
ATOM	1145	NE2	HIS	A	146	10.844	1.831	14.373	1.00	24.47	N
ATOM	1146	N	PRO	A	147	10.896	-4.885	12.783	1.00	18.32	N
ATOM	1147	CA	PRO	A	147	10.624	-5.945	11.843	1.00	19.37	C
ATOM	1148	C	PRO	A	147	9.128	-6.280	11.753	1.00	24.48	C
ATOM	1149	O	PRO	A	147	8.718	-6.993	10.833	1.00	27.95	O
ATOM	1150	CB	PRO	A	147	11.461	-7.097	12.365	1.00	17.35	C
ATOM	1151	CG	PRO	A	147	12.544	-6.430	13.108	1.00	17.68	C
ATOM	1152	CD	PRO	A	147	11.866	-5.332	13.806	1.00	18.03	C
ATOM	1153	N	TYR	A	148	8.294	-5.713	12.629	1.00	24.82	N
ATOM	1154	CA	TYR	A	148	6.868	-6.045	12.564	1.00	25.01	C
ATOM	1155	C	TYR	A	148	6.009	-4.990	11.846	1.00	26.88	C
ATOM	1156	O	TYR	A	148	5.048	-5.353	11.167	1.00	29.08	O
ATOM	1157	CB	TYR	A	148	6.326	-6.357	13.960	1.00	21.84	C
ATOM	1158	CG	TYR	A	148	6.986	-7.573	14.586	1.00	20.84	C
ATOM	1159	CD1	TYR	A	148	8.272	-7.502	15.093	1.00	21.27	C

ATOM	1160	CD2	TYR	A	148	6.368	-8.812	14.576	1.00	21.92	C
ATOM	1161	CE1	TYR	A	148	8.926	-8.629	15.558	1.00	18.20	C
ATOM	1162	CE2	TYR	A	148	7.019	-9.947	15.036	1.00	19.43	C
ATOM	1163	CZ	TYR	A	148	8.298	-9.843	15.521	1.00	17.54	C
ATOM	1164	OH	TYR	A	148	8.966	-10.954	15.973	1.00	19.26	O
ATOM	1165	N	PHE	A	149	6.519	-3.755	11.776	1.00	26.54	N
ATOM	1166	CA	PHE	A	149	5.862	-2.630	11.080	1.00	23.95	C
ATOM	1167	C	PHE	A	149	4.992	-3.027	9.893	1.00	23.50	C
ATOM	1168	O	PHE	A	149	5.444	-3.643	8.915	1.00	20.73	O
ATOM	1169	CB	PHE	A	149	6.892	-1.578	10.614	1.00	19.95	C
ATOM	1170	CG	PHE	A	149	6.368	-0.157	10.584	1.00	18.95	C
ATOM	1171	CD1	PHE	A	149	5.244	0.189	9.840	1.00	21.19	C
ATOM	1172	CD2	PHE	A	149	7.044	0.853	11.240	1.00	17.97	C
ATOM	1173	CE1	PHE	A	149	4.814	1.514	9.748	1.00	17.03	C
ATOM	1174	CE2	PHE	A	149	6.613	2.177	11.152	1.00	17.64	C
ATOM	1175	CZ	PHE	A	149	5.501	2.501	10.402	1.00	13.77	C
ATOM	1176	N	ALA	A	151	3.646	-2.885	6.915	1.00	16.85	N
ATOM	1177	CA	ALA	A	151	3.539	-2.791	5.463	1.00	14.78	C
ATOM	1178	C	ALA	A	151	2.681	-1.614	4.972	1.00	15.60	C
ATOM	1179	O	ALA	A	151	3.223	-0.518	4.831	1.00	17.98	O
ATOM	1180	CB	ALA	A	151	3.038	-4.104	4.884	1.00	13.97	C
ATOM	1181	N	PRO	A	152	1.346	-1.776	4.762	1.00	13.15	N
ATOM	1182	CA	PRO	A	152	0.722	-0.739	3.932	1.00	9.23	C
ATOM	1183	C	PRO	A	152	0.901	0.647	4.494	1.00	9.42	C
ATOM	1184	O	PRO	A	152	1.203	1.559	3.742	1.00	12.31	O
ATOM	1185	CB	PRO	A	152	-0.746	-1.137	3.863	1.00	3.96	C
ATOM	1186	CG	PRO	A	152	-0.950	-1.981	4.978	1.00	9.07	C
ATOM	1187	CD	PRO	A	152	0.329	-2.722	5.244	1.00	12.15	C
ATOM	1188	N	GLU	A	153	1.031	0.730	5.816	1.00	11.50	N
ATOM	1189	CA	GLU	A	153	1.234	2.006	6.514	1.00	9.86	C
ATOM	1190	C	GLU	A	153	2.605	2.638	6.305	1.00	10.68	C
ATOM	1191	O	GLU	A	153	2.752	3.857	6.337	1.00	9.78	O
ATOM	1192	CB	GLU	A	153	0.971	1.828	7.995	1.00	5.51	C
ATOM	1193	CG	GLU	A	153	-0.476	2.003	8.348	1.00	7.11	C
ATOM	1194	CD	GLU	A	153	-0.969	3.409	8.114	1.00	7.65	C
ATOM	1195	OE1	GLU	A	153	-0.136	4.313	8.022	1.00	5.80	O
ATOM	1196	OE2	GLU	A	153	-2.194	3.611	8.020	1.00	11.12	O1-
ATOM	1197	N	LEU	A	154	3.597	1.806	6.027	1.00	13.56	N
ATOM	1198	CA	LEU	A	154	4.861	2.287	5.484	1.00	17.58	C
ATOM	1199	C	LEU	A	154	4.729	2.816	4.045	1.00	19.77	C
ATOM	1200	O	LEU	A	154	5.245	3.881	3.731	1.00	19.71	O
ATOM	1201	CB	LEU	A	154	5.914	1.189	5.541	1.00	14.47	C
ATOM	1202	CG	LEU	A	154	7.016	1.644	6.467	1.00	16.08	C
ATOM	1203	CD1	LEU	A	154	8.162	0.670	6.454	1.00	16.69	C
ATOM	1204	CD2	LEU	A	154	7.461	3.019	6.022	1.00	19.40	C
ATOM	1205	N	LEU	A	155	3.982	2.112	3.201	1.00	20.63	N
ATOM	1206	CA	LEU	A	155	3.669	2.622	1.872	1.00	22.58	C
ATOM	1207	C	LEU	A	155	2.916	3.949	2.012	1.00	25.56	C
ATOM	1208	O	LEU	A	155	3.099	4.850	1.190	1.00	27.00	O
ATOM	1209	CB	LEU	A	155	2.801	1.636	1.075	1.00	19.65	C
ATOM	1210	CG	LEU	A	155	3.080	0.133	1.019	1.00	17.19	C
ATOM	1211	CD1	LEU	A	155	3.069	-0.273	-0.422	1.00	18.14	C
ATOM	1212	CD2	LEU	A	155	4.396	-0.259	1.647	1.00	17.17	C
ATOM	1213	N	PHE	A	156	2.120	4.101	3.079	1.00	26.64	N
ATOM	1214	CA	PHE	A	156	1.490	5.403	3.375	1.00	26.26	C
ATOM	1215	C	PHE	A	156	2.460	6.391	4.033	1.00	24.10	C
ATOM	1216	O	PHE	A	156	2.373	7.601	3.814	1.00	23.49	O
ATOM	1217	CB	PHE	A	156	0.240	5.268	4.260	1.00	23.92	C
ATOM	1218	CG	PHE	A	156	-0.286	6.590	4.749	1.00	21.57	C
ATOM	1219	CD1	PHE	A	156	-0.843	7.500	3.865	1.00	20.83	C
ATOM	1220	CD2	PHE	A	156	-0.113	6.977	6.066	1.00	20.34	C
ATOM	1221	CE1	PHE	A	156	-1.209	8.766	4.286	1.00	16.36	C
ATOM	1222	CE2	PHE	A	156	-0.481	8.251	6.492	1.00	17.60	C
ATOM	1223	CZ	PHE	A	156	-1.029	9.140	5.594	1.00	16.53	C
ATOM	1224	N	PHE	A	157	3.415	5.874	4.791	1.00	21.35	N
ATOM	1225	CA	PHE	A	157	4.441	6.724	5.339	1.00	21.42	C
ATOM	1226	C	PHE	A	157	5.657	6.859	4.427	1.00	22.33	C
ATOM	1227	O	PHE	A	157	6.712	7.303	4.879	1.00	23.73	O
ATOM	1228	CB	PHE	A	157	4.863	6.195	6.700	1.00	21.49	C
ATOM	1229	CG	PHE	A	157	4.238	6.920	7.846	1.00	17.83	C
ATOM	1230	CD1	PHE	A	157	3.628	8.139	7.652	1.00	17.24	C

ATOM	1231	CD2	PHE	A	157	4.376	6.444	9.120	1.00	19.41	C
ATOM	1232	CE1	PHE	A	157	3.197	8.872	8.688	1.00	14.49	C
ATOM	1233	CE2	PHE	A	157	3.948	7.170	10.159	1.00	18.81	C
ATOM	1234	CZ	PHE	A	157	3.362	8.398	9.940	1.00	20.97	C
ATOM	1235	N	ALA	A	158	5.524	6.405	3.175	1.00	23.41	N
ATOM	1236	CA	ALA	A	158	6.590	6.492	2.160	1.00	21.51	C
ATOM	1237	C	ALA	A	158	6.383	7.773	1.393	1.00	22.27	C
ATOM	1238	O	ALA	A	158	7.251	8.647	1.377	1.00	22.61	O
ATOM	1239	CB	ALA	A	158	6.526	5.321	1.199	1.00	16.28	C
ATOM	1240	N	LYS	A	159	5.185	7.930	0.840	1.00	22.44	N
ATOM	1241	CA	LYS	A	159	4.829	9.184	0.200	1.00	21.86	C
ATOM	1242	C	LYS	A	159	5.146	10.345	1.144	1.00	23.62	C
ATOM	1243	O	LYS	A	159	5.899	11.218	0.761	1.00	25.81	O
ATOM	1244	CB	LYS	A	159	3.358	9.187	-0.202	1.00	19.48	C
ATOM	1245	CG	LYS	A	159	3.096	8.664	-1.584	1.00	18.68	C
ATOM	1246	CD	LYS	A	159	4.087	7.579	-1.993	1.00	19.63	C
ATOM	1247	CE	LYS	A	159	3.781	7.040	-3.387	1.00	20.86	C
ATOM	1248	NZ	LYS	A	159	4.795	6.042	-3.824	1.00	23.26	N1+
ATOM	1249	N	ARG	A	160	4.850	10.195	2.436	1.00	23.94	N
ATOM	1250	CA	ARG	A	160	5.123	11.255	3.413	1.00	25.27	C
ATOM	1251	C	ARG	A	160	6.546	11.157	3.957	1.00	26.63	C
ATOM	1252	O	ARG	A	160	6.750	11.043	5.183	1.00	23.87	O
ATOM	1253	CB	ARG	A	160	4.135	11.184	4.583	1.00	28.93	C
ATOM	1254	CG	ARG	A	160	2.655	11.179	4.194	1.00	27.51	C
ATOM	1255	CD	ARG	A	160	2.030	12.549	4.347	1.00	25.69	C
ATOM	1256	NE	ARG	A	160	0.589	12.455	4.562	1.00	24.09	N
ATOM	1257	CZ	ARG	A	160	0.025	12.102	5.714	1.00	22.36	C
ATOM	1258	NH1	ARG	A	160	0.772	11.875	6.797	1.00	20.48	N1+
ATOM	1259	NH2	ARG	A	160	-1.292	11.998	5.786	1.00	19.93	N
ATOM	1260	N	TYR	A	161	7.468	10.909	3.020	1.00	26.96	N
ATOM	1261	CA	TYR	A	161	8.917	11.151	3.154	1.00	25.36	C
ATOM	1262	C	TYR	A	161	9.285	11.672	1.781	1.00	25.00	C
ATOM	1263	O	TYR	A	161	9.938	12.717	1.649	1.00	26.93	O
ATOM	1264	CB	TYR	A	161	9.666	9.839	3.404	1.00	22.15	C
ATOM	1265	CG	TYR	A	161	11.146	9.948	3.736	1.00	19.16	C
ATOM	1266	CD1	TYR	A	161	11.649	10.985	4.526	1.00	21.34	C
ATOM	1267	CD2	TYR	A	161	12.035	8.967	3.311	1.00	17.23	C
ATOM	1268	CE1	TYR	A	161	13.021	11.033	4.875	1.00	18.50	C
ATOM	1269	CE2	TYR	A	161	13.376	9.007	3.641	1.00	15.97	C
ATOM	1270	CZ	TYR	A	161	13.870	10.035	4.411	1.00	16.75	C
ATOM	1271	OH	TYR	A	161	15.220	10.080	4.642	1.00	14.91	O
ATOM	1272	N	LYS	A	162	8.684	11.030	0.776	1.00	24.44	N
ATOM	1273	CA	LYS	A	162	8.762	11.445	-0.629	1.00	20.54	C
ATOM	1274	C	LYS	A	162	8.217	12.847	-0.779	1.00	18.06	C
ATOM	1275	O	LYS	A	162	8.972	13.809	-0.711	1.00	18.82	O
ATOM	1276	CB	LYS	A	162	7.963	10.477	-1.508	1.00	19.05	C
ATOM	1277	CG	LYS	A	162	8.158	10.649	-3.005	1.00	18.72	C
ATOM	1278	CD	LYS	A	162	7.086	11.530	-3.600	1.00	15.41	C
ATOM	1279	CE	LYS	A	162	6.906	11.243	-5.053	1.00	13.03	C
ATOM	1280	NZ	LYS	A	162	6.676	9.809	-5.269	1.00	14.01	N1+
ATOM	1281	N	ALA	A	163	6.893	12.947	-0.846	1.00	17.36	N
ATOM	1282	CA	ALA	A	163	6.153	14.205	-0.954	1.00	17.24	C
ATOM	1283	C	ALA	A	163	6.795	15.380	-0.258	1.00	17.12	C
ATOM	1284	O	ALA	A	163	6.690	16.493	-0.748	1.00	19.51	O
ATOM	1285	CB	ALA	A	163	4.736	14.030	-0.439	1.00	14.73	C
ATOM	1286	N	ALA	A	164	7.419	15.144	0.894	1.00	16.39	N
ATOM	1287	CA	ALA	A	164	8.122	16.201	1.603	1.00	17.36	C
ATOM	1288	C	ALA	A	164	9.253	16.693	0.709	1.00	19.77	C
ATOM	1289	O	ALA	A	164	9.142	17.759	0.114	1.00	20.30	O
ATOM	1290	CB	ALA	A	164	8.670	15.686	2.930	1.00	15.34	C
ATOM	1291	N	PHE	A	165	10.238	15.827	0.466	1.00	21.94	N
ATOM	1292	CA	PHE	A	165	11.379	16.135	-0.393	1.00	20.00	C
ATOM	1293	C	PHE	A	165	10.864	16.794	-1.664	1.00	19.97	C
ATOM	1294	O	PHE	A	165	11.217	17.951	-1.933	1.00	19.72	O
ATOM	1295	CB	PHE	A	165	12.108	14.842	-0.765	1.00	24.10	C
ATOM	1296	CG	PHE	A	165	13.261	14.491	0.139	1.00	26.13	C
ATOM	1297	CD1	PHE	A	165	13.084	14.331	1.495	1.00	26.42	C
ATOM	1298	CD2	PHE	A	165	14.505	14.200	-0.396	1.00	27.17	C
ATOM	1299	CE1	PHE	A	165	14.128	13.891	2.287	1.00	24.89	C
ATOM	1300	CE2	PHE	A	165	15.536	13.758	0.397	1.00	22.89	C
ATOM	1301	CZ	PHE	A	165	15.350	13.609	1.730	1.00	22.00	C

ATOM	1302	N	THR	A	166	9.807	16.176	-2.209	1.00	18.51	N
ATOM	1303	CA	THR	A	166	9.151	16.502	-3.488	1.00	17.70	C
ATOM	1304	C	THR	A	166	8.432	17.857	-3.457	1.00	20.02	C
ATOM	1305	O	THR	A	166	7.358	18.038	-4.048	1.00	21.09	O
ATOM	1306	CB	THR	A	166	8.105	15.409	-3.838	1.00	18.48	C
ATOM	1307	CG2	THR	A	166	7.511	15.601	-5.242	1.00	15.89	C
ATOM	1308	OG1	THR	A	166	8.715	14.114	-3.735	1.00	18.39	O
ATOM	1309	N	GLU	A	167	8.978	18.778	-2.677	1.00	20.35	N
ATOM	1310	CA	GLU	A	167	8.511	20.145	-2.670	1.00	20.28	C
ATOM	1311	C	GLU	A	167	9.603	21.010	-2.066	1.00	18.79	C
ATOM	1312	O	GLU	A	167	10.110	21.924	-2.699	1.00	19.11	O
ATOM	1313	CB	GLU	A	167	7.243	20.228	-1.846	1.00	20.88	C
ATOM	1314	CG	GLU	A	167	6.759	21.612	-1.587	1.00	26.70	C
ATOM	1315	CD	GLU	A	167	5.856	21.656	-0.377	1.00	29.94	C
ATOM	1316	OE1	GLU	A	167	4.740	21.074	-0.432	1.00	32.74	O
ATOM	1317	OE2	GLU	A	167	6.300	22.200	0.658	1.00	32.31	O1-
ATOM	1318	N	CYS	A	168	10.066	20.636	-0.895	1.00	19.65	N
ATOM	1319	CA	CYS	A	168	10.978	21.490	-0.205	1.00	22.23	C
ATOM	1320	C	CYS	A	168	12.291	20.781	-0.033	1.00	25.31	C
ATOM	1321	O	CYS	A	168	12.626	20.370	1.077	1.00	29.59	O
ATOM	1322	CB	CYS	A	168	10.395	21.919	1.147	1.00	22.76	C
ATOM	1323	SG	CYS	A	168	11.603	22.432	2.429	1.00	26.02	S
ATOM	1324	N	CYS	A	169	13.009	20.566	-1.136	1.00	24.80	N
ATOM	1325	CA	CYS	A	169	14.459	20.798	-1.101	1.00	27.27	C
ATOM	1326	C	CYS	A	169	14.569	22.293	-1.412	1.00	26.49	C
ATOM	1327	O	CYS	A	169	15.379	23.015	-0.802	1.00	26.20	O
ATOM	1328	CB	CYS	A	169	15.190	19.988	-2.194	1.00	30.98	C
ATOM	1329	SG	CYS	A	169	15.841	18.333	-1.718	1.00	33.25	S
ATOM	1330	N	GLN	A	170	13.492	22.748	-2.059	1.00	26.22	N
ATOM	1331	CA	GLN	A	170	13.353	23.983	-2.844	1.00	25.86	C
ATOM	1332	C	GLN	A	170	13.998	25.203	-2.207	1.00	26.18	C
ATOM	1333	O	GLN	A	170	15.202	25.251	-2.033	1.00	30.02	O
ATOM	1334	CB	GLN	A	170	11.842	24.269	-3.085	1.00	25.67	C
ATOM	1335	CG	GLN	A	170	11.397	24.518	-4.531	1.00	21.68	C
ATOM	1336	CD	GLN	A	170	11.373	23.257	-5.393	1.00	20.70	C
ATOM	1337	NE2	GLN	A	170	12.540	22.814	-5.816	1.00	20.61	N
ATOM	1338	OE1	GLN	A	170	10.321	22.737	-5.727	1.00	16.98	O
ATOM	1339	N	ALA	A	171	13.192	26.161	-1.785	1.00	25.17	N
ATOM	1340	CA	ALA	A	171	13.718	27.493	-1.587	1.00	22.37	C
ATOM	1341	C	ALA	A	171	14.440	27.547	-0.279	1.00	23.13	C
ATOM	1342	O	ALA	A	171	15.176	28.489	-0.017	1.00	25.63	O
ATOM	1343	CB	ALA	A	171	12.619	28.501	-1.608	1.00	24.39	C
ATOM	1344	N	ALA	A	172	14.170	26.578	0.583	1.00	22.80	N
ATOM	1345	CA	ALA	A	172	14.686	26.649	1.935	1.00	23.27	C
ATOM	1346	C	ALA	A	172	15.967	25.845	2.129	1.00	23.80	C
ATOM	1347	O	ALA	A	172	16.315	25.519	3.270	1.00	21.53	O
ATOM	1348	CB	ALA	A	172	13.634	26.207	2.915	1.00	21.51	C
ATOM	1349	N	ASP	A	173	16.684	25.569	1.031	1.00	25.42	N
ATOM	1350	CA	ASP	A	173	17.901	24.751	1.088	1.00	28.45	C
ATOM	1351	C	ASP	A	173	17.612	23.425	1.779	1.00	30.18	C
ATOM	1352	O	ASP	A	173	18.403	22.987	2.608	1.00	31.67	O
ATOM	1353	CB	ASP	A	173	18.973	25.462	1.906	1.00	29.65	C
ATOM	1354	CG	ASP	A	173	19.953	26.208	1.057	1.00	30.99	C
ATOM	1355	OD1	ASP	A	173	19.492	27.029	0.227	1.00	32.55	O
ATOM	1356	OD2	ASP	A	173	21.176	25.999	1.265	1.00	32.46	O1-
ATOM	1357	N	LYS	A	174	16.377	22.957	1.652	1.00	30.45	N
ATOM	1358	CA	LYS	A	174	15.795	21.965	2.549	1.00	29.08	C
ATOM	1359	C	LYS	A	174	15.766	22.368	4.036	1.00	28.75	C
ATOM	1360	O	LYS	A	174	15.050	21.761	4.840	1.00	29.82	O
ATOM	1361	CB	LYS	A	174	16.501	20.618	2.369	1.00	29.28	C
ATOM	1362	CG	LYS	A	174	17.744	20.415	3.213	1.00	28.90	C
ATOM	1363	CD	LYS	A	174	17.508	19.425	4.326	1.00	28.34	C
ATOM	1364	CE	LYS	A	174	18.696	19.385	5.263	1.00	28.62	C
ATOM	1365	NZ	LYS	A	174	19.405	18.085	5.065	1.00	31.78	N1+
ATOM	1366	N	ALA	A	175	16.507	23.402	4.401	1.00	26.56	N
ATOM	1367	CA	ALA	A	175	16.781	23.678	5.795	1.00	27.02	C
ATOM	1368	C	ALA	A	175	15.532	23.529	6.661	1.00	27.16	C
ATOM	1369	O	ALA	A	175	15.266	22.457	7.181	1.00	27.67	O
ATOM	1370	CB	ALA	A	175	17.359	25.092	5.929	1.00	29.25	C
ATOM	1371	N	ALA	A	176	14.672	24.542	6.602	1.00	26.48	N
ATOM	1372	CA	ALA	A	176	13.614	24.748	7.567	1.00	20.96	C

ATOM	1373	C	ALA	A	176	12.325	24.254	6.976	1.00	21.35	C
ATOM	1374	O	ALA	A	176	11.676	23.432	7.557	1.00	22.19	O
ATOM	1375	CB	ALA	A	176	13.506	26.217	7.899	1.00	20.23	C
ATOM	1376	N	CYS	A	177	11.973	24.711	5.784	1.00	22.81	N
ATOM	1377	CA	CYS	A	177	10.706	24.305	5.168	1.00	25.45	C
ATOM	1378	C	CYS	A	177	10.548	22.808	4.974	1.00	25.72	C
ATOM	1379	O	CYS	A	177	9.532	22.387	4.413	1.00	25.67	O
ATOM	1380	CB	CYS	A	177	10.498	24.980	3.800	1.00	28.47	C
ATOM	1381	SG	CYS	A	177	11.590	24.464	2.414	1.00	27.41	S
ATOM	1382	N	LEU	A	178	11.645	22.068	5.169	1.00	25.62	N
ATOM	1383	CA	LEU	A	178	11.655	20.608	5.049	1.00	25.90	C
ATOM	1384	C	LEU	A	178	11.677	19.970	6.433	1.00	27.85	C
ATOM	1385	O	LEU	A	178	10.620	19.599	6.972	1.00	31.37	O
ATOM	1386	CB	LEU	A	178	12.880	20.117	4.255	1.00	23.18	C
ATOM	1387	CG	LEU	A	178	12.918	18.765	3.515	1.00	22.71	C
ATOM	1388	CD1	LEU	A	178	14.366	18.315	3.373	1.00	15.42	C
ATOM	1389	CD2	LEU	A	178	12.073	17.684	4.217	1.00	19.52	C
ATOM	1390	N	LEU	A	179	12.858	19.897	7.046	1.00	24.44	N
ATOM	1391	CA	LEU	A	179	13.079	18.960	8.143	1.00	23.00	C
ATOM	1392	C	LEU	A	179	12.215	19.038	9.449	1.00	23.84	C
ATOM	1393	O	LEU	A	179	12.326	18.165	10.315	1.00	22.67	O
ATOM	1394	CB	LEU	A	179	14.588	18.940	8.460	1.00	21.35	C
ATOM	1395	CG	LEU	A	179	15.248	19.579	9.697	1.00	23.42	C
ATOM	1396	CD1	LEU	A	179	16.763	19.387	9.589	1.00	22.06	C
ATOM	1397	CD2	LEU	A	179	14.898	21.062	9.845	1.00	18.81	C
ATOM	1398	N	PRO	A	180	11.301	20.026	9.578	1.00	24.90	N
ATOM	1399	CA	PRO	A	180	10.275	19.957	10.637	1.00	25.32	C
ATOM	1400	C	PRO	A	180	9.020	19.164	10.271	1.00	25.47	C
ATOM	1401	O	PRO	A	180	8.078	19.034	11.074	1.00	24.17	O
ATOM	1402	CB	PRO	A	180	9.956	21.425	10.914	1.00	25.04	C
ATOM	1403	CG	PRO	A	180	10.217	22.072	9.597	1.00	22.93	C
ATOM	1404	CD	PRO	A	180	11.523	21.427	9.210	1.00	24.99	C
ATOM	1405	N	LYS	A	181	9.018	18.641	9.054	1.00	24.23	N
ATOM	1406	CA	LYS	A	181	8.168	17.507	8.701	1.00	23.92	C
ATOM	1407	C	LYS	A	181	9.027	16.222	8.798	1.00	22.82	C
ATOM	1408	O	LYS	A	181	8.584	15.135	8.454	1.00	20.58	O
ATOM	1409	CB	LYS	A	181	7.615	17.711	7.286	1.00	25.23	C
ATOM	1410	CG	LYS	A	181	7.840	19.139	6.771	1.00	23.39	C
ATOM	1411	CD	LYS	A	181	7.528	19.289	5.312	1.00	21.94	C
ATOM	1412	CE	LYS	A	181	6.048	19.541	5.120	1.00	22.34	C
ATOM	1413	NZ	LYS	A	181	5.471	20.266	6.296	1.00	24.22	N1+
ATOM	1414	N	LEU	A	182	10.231	16.372	9.354	1.00	21.40	N
ATOM	1415	CA	LEU	A	182	11.159	15.277	9.612	1.00	18.92	C
ATOM	1416	C	LEU	A	182	11.513	15.303	11.107	1.00	18.56	C
ATOM	1417	O	LEU	A	182	12.482	14.685	11.539	1.00	16.25	O
ATOM	1418	CB	LEU	A	182	12.431	15.475	8.777	1.00	19.22	C
ATOM	1419	CG	LEU	A	182	12.869	14.556	7.621	1.00	20.67	C
ATOM	1420	CD1	LEU	A	182	12.740	13.115	8.064	1.00	22.41	C
ATOM	1421	CD2	LEU	A	182	12.070	14.796	6.342	1.00	16.92	C
ATOM	1422	N	ASP	A	183	10.857	16.186	11.849	1.00	19.20	N
ATOM	1423	CA	ASP	A	183	10.839	16.085	13.305	1.00	21.07	C
ATOM	1424	C	ASP	A	183	9.608	15.238	13.640	1.00	21.27	C
ATOM	1425	O	ASP	A	183	9.693	14.015	13.759	1.00	20.65	O
ATOM	1426	CB	ASP	A	183	10.731	17.485	13.960	1.00	22.49	C
ATOM	1427	CG	ASP	A	183	12.064	18.279	13.924	1.00	24.78	C
ATOM	1428	OD1	ASP	A	183	12.922	18.040	14.805	1.00	22.96	O
ATOM	1429	OD2	ASP	A	183	12.261	19.129	13.018	1.00	24.01	O1-
ATOM	1430	N	GLU	A	184	8.443	15.856	13.510	1.00	20.83	N
ATOM	1431	CA	GLU	A	184	7.190	15.210	13.853	1.00	20.58	C
ATOM	1432	C	GLU	A	184	7.081	13.767	13.329	1.00	19.58	C
ATOM	1433	O	GLU	A	184	7.119	12.822	14.118	1.00	18.60	O
ATOM	1434	CB	GLU	A	184	6.021	16.067	13.356	1.00	21.76	C
ATOM	1435	CG	GLU	A	184	4.698	15.723	13.997	1.00	25.58	C
ATOM	1436	CD	GLU	A	184	3.531	15.959	13.071	1.00	27.71	C
ATOM	1437	OE1	GLU	A	184	3.257	17.145	12.760	1.00	26.41	O
ATOM	1438	OE2	GLU	A	184	2.892	14.954	12.659	1.00	29.24	O1-
ATOM	1439	N	LEU	A	185	7.114	13.580	12.015	1.00	19.28	N
ATOM	1440	CA	LEU	A	185	6.872	12.254	11.438	1.00	21.28	C
ATOM	1441	C	LEU	A	185	8.027	11.229	11.664	1.00	21.48	C
ATOM	1442	O	LEU	A	185	8.321	10.422	10.797	1.00	20.44	O
ATOM	1443	CB	LEU	A	185	6.549	12.387	9.930	1.00	21.87	C

ATOM	1444	CG	LEU	A	185	5.217	13.009	9.440	1.00	24.75	C
ATOM	1445	CD1	LEU	A	185	5.400	14.429	8.858	1.00	21.49	C
ATOM	1446	CD2	LEU	A	185	4.605	12.100	8.376	1.00	22.23	C
ATOM	1447	N	ARG	A	186	8.728	11.323	12.789	1.00	21.60	N
ATOM	1448	CA	ARG	A	186	9.692	10.304	13.191	1.00	20.65	C
ATOM	1449	C	ARG	A	186	9.175	9.608	14.448	1.00	23.63	C
ATOM	1450	O	ARG	A	186	9.353	8.393	14.626	1.00	24.05	O
ATOM	1451	CB	ARG	A	186	11.036	10.941	13.514	1.00	21.81	C
ATOM	1452	CG	ARG	A	186	11.149	11.524	14.926	1.00	20.40	C
ATOM	1453	CD	ARG	A	186	12.527	11.273	15.525	1.00	22.73	C
ATOM	1454	NE	ARG	A	186	12.739	9.855	15.758	1.00	24.25	N
ATOM	1455	CZ	ARG	A	186	13.468	9.358	16.750	1.00	26.77	C
ATOM	1456	NH1	ARG	A	186	14.043	10.164	17.638	1.00	26.94	N1+
ATOM	1457	NH2	ARG	A	186	13.561	8.042	16.896	1.00	28.22	N
ATOM	1458	N	ASP	A	187	8.589	10.399	15.349	1.00	23.17	N
ATOM	1459	CA	ASP	A	187	7.844	9.858	16.463	1.00	20.77	C
ATOM	1460	C	ASP	A	187	6.644	9.094	15.937	1.00	18.87	C
ATOM	1461	O	ASP	A	187	6.412	7.993	16.382	1.00	18.84	O
ATOM	1462	CB	ASP	A	187	7.385	10.972	17.388	1.00	23.77	C
ATOM	1463	CG	ASP	A	187	8.523	11.556	18.208	1.00	26.43	C
ATOM	1464	OD1	ASP	A	187	8.918	10.937	19.241	1.00	27.86	O
ATOM	1465	OD2	ASP	A	187	8.981	12.669	17.847	1.00	26.15	O1-
ATOM	1466	N	GLU	A	188	6.000	9.594	14.880	1.00	18.08	N
ATOM	1467	CA	GLU	A	188	4.861	8.894	14.247	1.00	18.47	C
ATOM	1468	C	GLU	A	188	5.280	7.641	13.475	1.00	18.83	C
ATOM	1469	O	GLU	A	188	4.440	6.970	12.890	1.00	18.98	O
ATOM	1470	CB	GLU	A	188	4.074	9.840	13.299	1.00	16.29	C
ATOM	1471	CG	GLU	A	188	2.648	9.362	12.934	1.00	12.67	C
ATOM	1472	CD	GLU	A	188	1.770	10.425	12.231	1.00	16.08	C
ATOM	1473	OE1	GLU	A	188	2.195	11.591	12.086	1.00	16.21	O
ATOM	1474	OE2	GLU	A	188	0.607	10.109	11.882	1.00	14.17	O1-
ATOM	1475	N	GLY	A	189	6.587	7.418	13.355	1.00	20.49	N
ATOM	1476	CA	GLY	A	189	7.098	6.195	12.753	1.00	19.35	C
ATOM	1477	C	GLY	A	189	7.221	5.173	13.857	1.00	18.26	C
ATOM	1478	O	GLY	A	189	6.532	4.161	13.867	1.00	18.56	O
ATOM	1479	N	LYS	A	190	7.964	5.536	14.886	1.00	20.08	N
ATOM	1480	CA	LYS	A	190	7.903	4.816	16.152	1.00	22.77	C
ATOM	1481	C	LYS	A	190	6.463	4.574	16.647	1.00	22.46	C
ATOM	1482	O	LYS	A	190	6.120	3.434	16.940	1.00	24.59	O
ATOM	1483	CB	LYS	A	190	8.710	5.555	17.232	1.00	24.21	C
ATOM	1484	CG	LYS	A	190	10.186	5.699	16.889	1.00	27.27	C
ATOM	1485	CD	LYS	A	190	11.034	5.974	18.115	1.00	31.27	C
ATOM	1486	CE	LYS	A	190	10.959	7.431	18.550	1.00	32.12	C
ATOM	1487	NZ	LYS	A	190	12.010	7.723	19.570	1.00	31.95	N1+
ATOM	1488	N	ALA	A	191	5.594	5.589	16.605	1.00	20.34	N
ATOM	1489	CA	ALA	A	191	4.280	5.495	17.239	1.00	17.99	C
ATOM	1490	C	ALA	A	191	3.575	4.269	16.726	1.00	17.52	C
ATOM	1491	O	ALA	A	191	3.591	3.250	17.391	1.00	21.21	O
ATOM	1492	CB	ALA	A	191	3.442	6.712	16.971	1.00	17.43	C
ATOM	1493	N	SER	A	192	3.200	4.269	15.458	1.00	16.48	N
ATOM	1494	CA	SER	A	192	2.445	3.151	14.932	1.00	15.33	C
ATOM	1495	C	SER	A	192	3.246	1.855	14.918	1.00	17.13	C
ATOM	1496	O	SER	A	192	2.673	0.771	15.078	1.00	18.89	O
ATOM	1497	CB	SER	A	192	1.943	3.446	13.538	1.00	14.90	C
ATOM	1498	OG	SER	A	192	1.128	2.380	13.116	1.00	19.59	O
ATOM	1499	N	SER	A	193	4.567	1.946	14.777	1.00	13.75	N
ATOM	1500	CA	SER	A	193	5.377	0.738	14.665	1.00	11.93	C
ATOM	1501	C	SER	A	193	5.408	0.064	15.996	1.00	10.26	C
ATOM	1502	O	SER	A	193	6.136	-0.909	16.173	1.00	8.48	O
ATOM	1503	CB	SER	A	193	6.802	1.089	14.258	1.00	15.39	C
ATOM	1504	OG	SER	A	193	7.526	1.680	15.322	1.00	15.80	O
ATOM	1505	N	ALA	A	194	4.916	0.833	16.965	1.00	13.13	N
ATOM	1506	CA	ALA	A	194	4.875	0.494	18.384	1.00	14.96	C
ATOM	1507	C	ALA	A	194	3.436	0.431	18.859	1.00	12.28	C
ATOM	1508	O	ALA	A	194	3.117	-0.360	19.733	1.00	16.44	O
ATOM	1509	CB	ALA	A	194	5.647	1.543	19.205	1.00	14.37	C
ATOM	1510	N	LYS	A	195	2.560	1.162	18.186	1.00	8.87	N
ATOM	1511	CA	LYS	A	195	1.129	1.081	18.415	1.00	10.05	C
ATOM	1512	C	LYS	A	195	0.656	-0.181	17.755	1.00	9.99	C
ATOM	1513	O	LYS	A	195	-0.535	-0.421	17.682	1.00	12.22	O
ATOM	1514	CB	LYS	A	195	0.390	2.267	17.767	1.00	12.99	C

```
ATOM   1515  CG  LYS A 195       0.478   3.588  18.531  1.00 15.37           C
ATOM   1516  CD  LYS A 195       0.179   4.787  17.664  1.00 16.04           C
ATOM   1517  CE  LYS A 195      -1.184   5.396  17.995  1.00 19.24           C
ATOM   1518  NZ  LYS A 195      -1.397   6.691  17.246  1.00 20.94           N1+
ATOM   1519  N   GLN A 196       1.586  -0.882  17.112  1.00 13.42           N
ATOM   1520  CA  GLN A 196       1.302  -2.120  16.370  1.00 13.91           C
ATOM   1521  C   GLN A 196       1.790  -3.377  17.084  1.00 13.37           C
ATOM   1522  O   GLN A 196       0.998  -4.282  17.270  1.00 15.65           O
ATOM   1523  CB  GLN A 196       1.923  -2.071  14.971  1.00 13.77           C
ATOM   1524  CG  GLN A 196       1.763  -3.343  14.183  1.00 15.75           C
ATOM   1525  CD  GLN A 196       0.630  -3.266  13.192  1.00 20.39           C
ATOM   1526  NE2 GLN A 196      -0.021  -2.104  13.132  1.00 21.97           N
ATOM   1527  OE1 GLN A 196       0.344  -4.228  12.470  1.00 19.31           O
ATOM   1528  N   ARG A 197       3.063  -3.437  17.495  1.00 10.81           N
ATOM   1529  CA  ARG A 197       3.666  -4.683  17.989  1.00 10.56           C
ATOM   1530  C   ARG A 197       2.685  -5.310  18.950  1.00 12.83           C
ATOM   1531  O   ARG A 197       2.476  -6.508  18.910  1.00 14.23           O
ATOM   1532  CB  ARG A 197       5.014  -4.404  18.679  1.00 11.17           C
ATOM   1533  CG  ARG A 197       5.808  -5.620  19.258  1.00 11.07           C
ATOM   1534  CD  ARG A 197       5.853  -6.866  18.353  1.00  8.47           C
ATOM   1535  NE  ARG A 197       6.520  -8.013  18.986  1.00  6.63           N
ATOM   1536  CZ  ARG A 197       5.931  -9.185  19.260  1.00  8.70           C
ATOM   1537  NH1 ARG A 197       4.618  -9.319  19.201  1.00  7.16           N1+
ATOM   1538  NH2 ARG A 197       6.645 -10.211  19.706  1.00  7.19           N
ATOM   1539  N   LEU A 198       1.906  -4.441  19.595  1.00 14.31           N
ATOM   1540  CA  LEU A 198       0.664  -4.783  20.286  1.00 15.62           C
ATOM   1541  C   LEU A 198      -0.305  -5.651  19.460  1.00 18.97           C
ATOM   1542  O   LEU A 198      -0.561  -6.800  19.835  1.00 22.05           O
ATOM   1543  CB  LEU A 198      -0.047  -3.501  20.714  1.00 12.42           C
ATOM   1544  CG  LEU A 198      -0.455  -3.324  22.174  1.00 13.86           C
ATOM   1545  CD1 LEU A 198      -1.936  -2.970  22.232  1.00 13.35           C
ATOM   1546  CD2 LEU A 198      -0.162  -4.573  22.987  1.00 10.76           C
ATOM   1547  N   LYS A 199      -0.925  -5.076  18.423  1.00 18.33           N
ATOM   1548  CA  LYS A 199      -1.898  -5.785  17.578  1.00 18.04           C
ATOM   1549  C   LYS A 199      -1.443  -7.160  17.098  1.00 19.50           C
ATOM   1550  O   LYS A 199      -2.262  -7.986  16.724  1.00 20.23           O
ATOM   1551  CB  LYS A 199      -2.249  -4.949  16.355  1.00 18.92           C
ATOM   1552  CG  LYS A 199      -3.608  -4.261  16.389  1.00 18.14           C
ATOM   1553  CD  LYS A 199      -3.598  -2.944  15.594  1.00 13.47           C
ATOM   1554  CE  LYS A 199      -2.700  -1.907  16.227  1.00  8.90           C
ATOM   1555  NZ  LYS A 199      -2.448  -2.218  17.659  1.00  9.83           N1+
ATOM   1556  N   CYS A 200      -0.140  -7.375  17.001  1.00 21.92           N
ATOM   1557  CA  CYS A 200       0.372  -8.717  16.755  1.00 23.15           C
ATOM   1558  C   CYS A 200       0.459  -9.453  18.071  1.00 25.39           C
ATOM   1559  O   CYS A 200       0.080 -10.614  18.142  1.00 29.62           O
ATOM   1560  CB  CYS A 200       1.762  -8.682  16.118  1.00 22.40           C
ATOM   1561  SG  CYS A 200       1.793  -7.924  14.469  1.00 21.69           S
ATOM   1562  N   ALA A 201       0.949  -8.778  19.114  1.00 26.47           N
ATOM   1563  CA  ALA A 201       1.139  -9.381  20.440  1.00 23.93           C
ATOM   1564  C   ALA A 201      -0.165  -9.943  20.993  1.00 21.85           C
ATOM   1565  O   ALA A 201      -0.208 -11.107  21.360  1.00 20.07           O
ATOM   1566  CB  ALA A 201       1.740  -8.371  21.408  1.00 24.67           C
ATOM   1567  N   SER A 202      -1.245  -9.173  20.919  1.00 20.21           N
ATOM   1568  CA  SER A 202      -2.584  -9.760  20.944  1.00 22.62           C
ATOM   1569  C   SER A 202      -2.608 -11.018  20.056  1.00 23.67           C
ATOM   1570  O   SER A 202      -2.798 -12.128  20.544  1.00 24.94           O
ATOM   1571  CB  SER A 202      -3.644  -8.779  20.400  1.00 23.40           C
ATOM   1572  OG  SER A 202      -3.857  -7.642  21.221  1.00 23.09           O
ATOM   1573  N   LEU A 203      -2.326 -10.847  18.765  1.00 24.01           N
ATOM   1574  CA  LEU A 203      -2.674 -11.850  17.762  1.00 22.30           C
ATOM   1575  C   LEU A 203      -2.159 -13.281  17.940  1.00 20.69           C
ATOM   1576  O   LEU A 203      -2.961 -14.201  17.903  1.00 20.01           O
ATOM   1577  CB  LEU A 203      -2.320 -11.341  16.368  1.00 21.07           C
ATOM   1578  CG  LEU A 203      -3.523 -11.255  15.429  1.00 20.68           C
ATOM   1579  CD1 LEU A 203      -4.289 -12.558  15.445  1.00 22.29           C
ATOM   1580  CD2 LEU A 203      -4.410 -10.140  15.868  1.00 20.27           C
ATOM   1581  N   GLN A 204      -0.843 -13.487  17.919  1.00 20.66           N
ATOM   1582  CA  GLN A 204      -0.277 -14.834  18.099  1.00 23.10           C
ATOM   1583  C   GLN A 204       1.062 -14.932  18.874  1.00 24.84           C
ATOM   1584  O   GLN A 204       1.816 -15.917  18.736  1.00 25.14           O
ATOM   1585  CB  GLN A 204      -0.135 -15.567  16.762  1.00 22.96           C
```

```
ATOM   1586  CG  GLN A 204      -0.633 -14.814  15.556  1.00 24.32           C
ATOM   1587  CD  GLN A 204       0.297 -14.952  14.367  1.00 23.07           C
ATOM   1588  NE2 GLN A 204       0.408 -13.888  13.570  1.00 22.46           N
ATOM   1589  OE1 GLN A 204       0.920 -15.990  14.175  1.00 21.96           O
ATOM   1590  N   LYS A 205       1.345 -13.922  19.695  1.00 21.97           N
ATOM   1591  CA  LYS A 205       2.050 -14.152  20.952  1.00 18.40           C
ATOM   1592  C   LYS A 205       0.998 -14.276  22.063  1.00 14.76           C
ATOM   1593  O   LYS A 205       1.311 -14.599  23.192  1.00 13.45           O
ATOM   1594  CB  LYS A 205       3.020 -13.007  21.232  1.00 19.14           C
ATOM   1595  CG  LYS A 205       2.841 -12.327  22.558  1.00 18.87           C
ATOM   1596  CD  LYS A 205       3.969 -12.710  23.485  1.00 21.97           C
ATOM   1597  CE  LYS A 205       5.126 -11.720  23.351  1.00 23.57           C
ATOM   1598  NZ  LYS A 205       6.410 -12.330  23.789  1.00 23.75           N1+
ATOM   1599  N   PHE A 206      -0.253 -14.040  21.707  1.00 12.24           N
ATOM   1600  CA  PHE A 206      -1.395 -14.291  22.578  1.00 13.28           C
ATOM   1601  C   PHE A 206      -2.440 -14.872  21.656  1.00 13.87           C
ATOM   1602  O   PHE A 206      -2.389 -14.638  20.459  1.00 15.15           O
ATOM   1603  CB  PHE A 206      -1.945 -12.972  23.110  1.00 14.11           C
ATOM   1604  CG  PHE A 206      -1.995 -12.870  24.594  1.00  8.89           C
ATOM   1605  CD1 PHE A 206      -0.961 -12.280  25.264  1.00  7.13           C
ATOM   1606  CD2 PHE A 206      -3.173 -13.071  25.251  1.00  6.33           C
ATOM   1607  CE1 PHE A 206      -1.102 -11.880  26.508  1.00  7.41           C
ATOM   1608  CE2 PHE A 206      -3.317 -12.670  26.507  1.00  8.37           C
ATOM   1609  CZ  PHE A 206      -2.278 -12.066  27.139  1.00  9.60           C
ATOM   1610  N   GLY A 207      -3.502 -15.416  22.230  1.00 14.43           N
ATOM   1611  CA  GLY A 207      -4.553 -16.003  21.425  1.00 14.40           C
ATOM   1612  C   GLY A 207      -5.095 -15.066  20.366  1.00 15.46           C
ATOM   1613  O   GLY A 207      -5.411 -13.904  20.628  1.00 16.20           O
ATOM   1614  N   GLU A 208      -5.330 -15.625  19.192  1.00 15.63           N
ATOM   1615  CA  GLU A 208      -5.998 -14.904  18.125  1.00 18.59           C
ATOM   1616  C   GLU A 208      -7.446 -14.492  18.494  1.00 19.71           C
ATOM   1617  O   GLU A 208      -7.997 -13.540  17.917  1.00 21.56           O
ATOM   1618  CB  GLU A 208      -5.971 -15.761  16.845  1.00 18.16           C
ATOM   1619  CG  GLU A 208      -7.133 -16.747  16.691  1.00 18.76           C
ATOM   1620  CD  GLU A 208      -8.245 -16.193  15.814  1.00 19.03           C
ATOM   1621  OE1 GLU A 208      -8.448 -14.963  15.793  1.00 18.38           O
ATOM   1622  OE2 GLU A 208      -8.860 -16.979  15.069  1.00 22.62           O1-
ATOM   1623  N   ARG A 209      -8.083 -15.253  19.389  1.00 18.66           N
ATOM   1624  CA  ARG A 209      -9.477 -15.021  19.769  1.00 17.70           C
ATOM   1625  C   ARG A 209      -9.626 -13.656  20.423  1.00 18.80           C
ATOM   1626  O   ARG A 209     -10.580 -12.930  20.170  1.00 17.41           O
ATOM   1627  CB  ARG A 209      -9.939 -16.095  20.751  1.00 17.78           C
ATOM   1628  CG  ARG A 209     -11.325 -16.650  20.479  1.00 22.53           C
ATOM   1629  CD  ARG A 209     -12.149 -15.748  19.536  1.00 23.84           C
ATOM   1630  NE  ARG A 209     -12.523 -14.506  20.204  1.00 25.57           N
ATOM   1631  CZ  ARG A 209     -13.644 -13.825  19.989  1.00 25.17           C
ATOM   1632  NH1 ARG A 209     -14.470 -14.180  19.011  1.00 27.58           N1+
ATOM   1633  NH2 ARG A 209     -13.900 -12.739  20.714  1.00 24.46           N
ATOM   1634  N   ALA A 210      -8.623 -13.292  21.210  1.00 22.42           N
ATOM   1635  CA  ALA A 210      -8.644 -12.078  21.995  1.00 25.29           C
ATOM   1636  C   ALA A 210      -8.974 -10.918  21.071  1.00 27.14           C
ATOM   1637  O   ALA A 210     -10.042 -10.313  21.208  1.00 29.89           O
ATOM   1638  CB  ALA A 210      -7.289 -11.867  22.666  1.00 24.38           C
ATOM   1639  N   PHE A 211      -8.173 -10.760  20.012  1.00 26.77           N
ATOM   1640  CA  PHE A 211      -8.289  -9.629  19.081  1.00 23.38           C
ATOM   1641  C   PHE A 211      -9.721  -9.257  18.741  1.00 21.80           C
ATOM   1642  O   PHE A 211     -10.079  -8.090  18.838  1.00 18.92           O
ATOM   1643  CB  PHE A 211      -7.543  -9.926  17.788  1.00 25.16           C
ATOM   1644  CG  PHE A 211      -7.077  -8.695  17.057  1.00 26.16           C
ATOM   1645  CD1 PHE A 211      -6.020  -7.951  17.542  1.00 25.15           C
ATOM   1646  CD2 PHE A 211      -7.562  -8.404  15.786  1.00 25.67           C
ATOM   1647  CE1 PHE A 211      -5.440  -6.965  16.771  1.00 23.64           C
ATOM   1648  CE2 PHE A 211      -6.994  -7.414  15.017  1.00 23.27           C
ATOM   1649  CZ  PHE A 211      -5.925  -6.698  15.511  1.00 25.63           C
ATOM   1650  N   LYS A 212     -10.536 -10.244  18.363  1.00 20.64           N
ATOM   1651  CA  LYS A 212     -11.955 -10.003  18.096  1.00 20.36           C
ATOM   1652  C   LYS A 212     -12.497  -9.141  19.218  1.00 22.63           C
ATOM   1653  O   LYS A 212     -12.772  -7.964  19.004  1.00 24.04           O
ATOM   1654  CB  LYS A 212     -12.748 -11.308  18.044  1.00 19.99           C
ATOM   1655  CG  LYS A 212     -13.261 -11.723  16.651  1.00 22.19           C
ATOM   1656  CD  LYS A 212     -12.319 -12.755  15.969  1.00 17.63           C
```

ATOM	1657	CE	LYS	A	212	-13.061	-13.725	15.077	1.00	14.45	C
ATOM	1658	NZ	LYS	A	212	-14.268	-13.106	14.457	1.00	14.02	N1+
ATOM	1659	N	ALA	A	213	-12.384	-9.642	20.446	1.00	22.31	N
ATOM	1660	CA	ALA	A	213	-12.827	-8.900	21.614	1.00	21.34	C
ATOM	1661	C	ALA	A	213	-12.149	-7.537	21.705	1.00	21.10	C
ATOM	1662	O	ALA	A	213	-12.763	-6.552	22.098	1.00	23.45	O
ATOM	1663	CB	ALA	A	213	-12.569	-9.696	22.871	1.00	20.73	C
ATOM	1664	N	TRP	A	214	-10.922	-7.437	21.240	1.00	19.46	N
ATOM	1665	CA	TRP	A	214	-10.271	-6.142	21.224	1.00	19.15	C
ATOM	1666	C	TRP	A	214	-11.048	-5.224	20.314	1.00	18.85	C
ATOM	1667	O	TRP	A	214	-11.516	-4.175	20.734	1.00	20.49	O
ATOM	1668	CB	TRP	A	214	-8.870	-6.296	20.686	1.00	22.36	C
ATOM	1669	CG	TRP	A	214	-7.861	-5.482	21.350	1.00	24.56	C
ATOM	1670	CD1	TRP	A	214	-6.926	-5.942	22.246	1.00	26.33	C
ATOM	1671	CD2	TRP	A	214	-7.377	-4.211	20.888	1.00	25.12	C
ATOM	1672	CE2	TRP	A	214	-6.120	-4.005	21.484	1.00	28.85	C
ATOM	1673	CE3	TRP	A	214	-7.863	-3.267	19.975	1.00	21.79	C
ATOM	1674	NE1	TRP	A	214	-5.867	-5.073	22.305	1.00	31.06	N
ATOM	1675	CZ2	TRP	A	214	-5.341	-2.887	21.189	1.00	28.30	C
ATOM	1676	CZ3	TRP	A	214	-7.100	-2.178	19.689	1.00	18.16	C
ATOM	1677	CH2	TRP	A	214	-5.852	-1.991	20.287	1.00	25.36	C
ATOM	1678	N	ALA	A	215	-11.279	-5.677	19.092	1.00	18.46	N
ATOM	1679	CA	ALA	A	215	-11.815	-4.819	18.056	1.00	18.34	C
ATOM	1680	C	ALA	A	215	-13.346	-4.685	18.072	1.00	19.69	C
ATOM	1681	O	ALA	A	215	-13.853	-3.636	17.695	1.00	19.89	O
ATOM	1682	CB	ALA	A	215	-11.337	-5.295	16.705	1.00	17.27	C
ATOM	1683	N	VAL	A	216	-14.074	-5.711	18.532	1.00	19.07	N
ATOM	1684	CA	VAL	A	216	-15.533	-5.613	18.768	1.00	18.57	C
ATOM	1685	C	VAL	A	216	-15.801	-4.635	19.911	1.00	16.42	C
ATOM	1686	O	VAL	A	216	-16.854	-4.013	19.985	1.00	16.02	O
ATOM	1687	CB	VAL	A	216	-16.199	-6.979	19.159	1.00	18.60	C
ATOM	1688	CG1	VAL	A	216	-17.658	-6.980	18.765	1.00	18.91	C
ATOM	1689	CG2	VAL	A	216	-15.530	-8.129	18.488	1.00	18.58	C
ATOM	1690	N	ALA	A	217	-14.860	-4.555	20.837	1.00	15.89	N
ATOM	1691	CA	ALA	A	217	-14.811	-3.444	21.767	1.00	17.11	C
ATOM	1692	C	ALA	A	217	-14.553	-2.125	21.050	1.00	17.79	C
ATOM	1693	O	ALA	A	217	-15.155	-1.114	21.413	1.00	20.39	O
ATOM	1694	CB	ALA	A	217	-13.734	-3.679	22.827	1.00	17.06	C
ATOM	1695	N	ARG	A	218	-13.664	-2.114	20.050	1.00	16.98	N
ATOM	1696	CA	ARG	A	218	-13.190	-0.845	19.494	1.00	14.92	C
ATOM	1697	C	ARG	A	218	-13.902	-0.275	18.310	1.00	13.69	C
ATOM	1698	O	ARG	A	218	-14.093	0.920	18.241	1.00	17.63	O
ATOM	1699	CB	ARG	A	218	-11.717	-0.886	19.162	1.00	16.75	C
ATOM	1700	CG	ARG	A	218	-11.031	0.429	19.482	1.00	20.34	C
ATOM	1701	CD	ARG	A	218	-9.529	0.272	19.350	1.00	28.41	C
ATOM	1702	NE	ARG	A	218	-8.742	0.955	20.381	1.00	31.43	N
ATOM	1703	CZ	ARG	A	218	-7.410	0.999	20.357	1.00	31.18	C
ATOM	1704	NH1	ARG	A	218	-6.781	0.627	19.249	1.00	33.54	N1+
ATOM	1705	NH2	ARG	A	218	-6.709	1.463	21.392	1.00	28.29	N
ATOM	1706	N	LEU	A	219	-14.188	-1.074	17.304	1.00	13.67	N
ATOM	1707	CA	LEU	A	219	-14.863	-0.525	16.134	1.00	13.57	C
ATOM	1708	C	LEU	A	219	-16.279	-0.119	16.523	1.00	13.16	C
ATOM	1709	O	LEU	A	219	-16.875	0.721	15.861	1.00	11.91	O
ATOM	1710	CB	LEU	A	219	-14.861	-1.538	14.977	1.00	12.34	C
ATOM	1711	CG	LEU	A	219	-13.507	-2.219	14.718	1.00	11.08	C
ATOM	1712	CD1	LEU	A	219	-13.701	-3.486	13.935	1.00	12.13	C
ATOM	1713	CD2	LEU	A	219	-12.585	-1.277	13.980	1.00	10.31	C
ATOM	1714	N	SER	A	220	-16.711	-0.572	17.704	1.00	13.60	N
ATOM	1715	CA	SER	A	220	-18.039	-0.287	18.266	1.00	13.96	C
ATOM	1716	C	SER	A	220	-18.006	0.971	19.116	1.00	13.33	C
ATOM	1717	O	SER	A	220	-19.027	1.613	19.324	1.00	12.54	O
ATOM	1718	CB	SER	A	220	-18.527	-1.462	19.127	1.00	15.85	C
ATOM	1719	OG	SER	A	220	-19.168	-2.479	18.357	1.00	16.62	O
ATOM	1720	N	GLN	A	221	-16.798	1.360	19.507	1.00	15.03	N
ATOM	1721	CA	GLN	A	221	-16.498	2.618	20.203	1.00	14.21	C
ATOM	1722	C	GLN	A	221	-16.470	3.821	19.238	1.00	15.97	C
ATOM	1723	O	GLN	A	221	-16.314	4.959	19.669	1.00	17.34	O
ATOM	1724	CB	GLN	A	221	-15.127	2.473	20.843	1.00	13.10	C
ATOM	1725	CG	GLN	A	221	-14.878	3.266	22.062	1.00	20.06	C
ATOM	1726	CD	GLN	A	221	-14.119	2.455	23.085	1.00	24.79	C
ATOM	1727	NE2	GLN	A	221	-14.716	2.285	24.265	1.00	27.31	N

ATOM	1728	OE1	GLN	A	221	-13.066	1.884	22.784	1.00	24.46	O
ATOM	1729	N	ARG	A	222	-16.391	3.530	17.937	1.00	16.06	N
ATOM	1730	CA	ARG	A	222	-16.293	4.545	16.884	1.00	14.67	C
ATOM	1731	C	ARG	A	222	-17.453	4.450	15.874	1.00	14.79	C
ATOM	1732	O	ARG	A	222	-17.942	5.455	15.360	1.00	16.58	O
ATOM	1733	CB	ARG	A	222	-14.951	4.423	16.141	1.00	13.32	C
ATOM	1734	CG	ARG	A	222	-13.933	3.526	16.803	1.00	10.78	C
ATOM	1735	CD	ARG	A	222	-12.562	4.147	16.820	1.00	14.99	C
ATOM	1736	NE	ARG	A	222	-11.738	3.584	17.887	1.00	16.71	N
ATOM	1737	CZ	ARG	A	222	-10.937	4.294	18.683	1.00	17.32	C
ATOM	1738	NH1	ARG	A	222	-10.806	5.607	18.507	1.00	16.06	N1+
ATOM	1739	NH2	ARG	A	222	-10.252	3.687	19.654	1.00	12.90	N
ATOM	1740	N	PHE	A	223	-17.957	3.250	15.647	1.00	14.56	N
ATOM	1741	CA	PHE	A	223	-19.111	3.084	14.792	1.00	12.35	C
ATOM	1742	C	PHE	A	223	-20.298	2.602	15.626	1.00	13.17	C
ATOM	1743	O	PHE	A	223	-20.916	1.601	15.272	1.00	12.50	O
ATOM	1744	CB	PHE	A	223	-18.820	2.042	13.706	1.00	15.57	C
ATOM	1745	CG	PHE	A	223	-17.648	2.373	12.788	1.00	18.20	C
ATOM	1746	CD1	PHE	A	223	-16.762	3.398	13.060	1.00	18.12	C
ATOM	1747	CD2	PHE	A	223	-17.389	1.571	11.689	1.00	19.72	C
ATOM	1748	CE1	PHE	A	223	-15.642	3.594	12.261	1.00	20.71	C
ATOM	1749	CE2	PHE	A	223	-16.274	1.765	10.896	1.00	18.61	C
ATOM	1750	CZ	PHE	A	223	-15.401	2.767	11.182	1.00	18.31	C
ATOM	1751	N	PRO	A	224	-20.667	3.323	16.717	1.00	14.94	N
ATOM	1752	CA	PRO	A	224	-21.635	2.860	17.714	1.00	14.17	C
ATOM	1753	C	PRO	A	224	-23.096	2.979	17.275	1.00	15.44	C
ATOM	1754	O	PRO	A	224	-23.942	3.488	18.004	1.00	16.00	O
ATOM	1755	CB	PRO	A	224	-21.344	3.757	18.900	1.00	13.07	C
ATOM	1756	CG	PRO	A	224	-21.074	5.010	18.284	1.00	14.26	C
ATOM	1757	CD	PRO	A	224	-20.226	4.679	17.073	1.00	17.21	C
ATOM	1758	N	LYS	A	225	-23.347	2.630	16.026	1.00	15.99	N
ATOM	1759	CA	LYS	A	225	-24.668	2.300	15.555	1.00	15.34	C
ATOM	1760	C	LYS	A	225	-24.515	1.109	14.603	1.00	20.08	C
ATOM	1761	O	LYS	A	225	-25.481	0.396	14.332	1.00	23.63	O
ATOM	1762	CB	LYS	A	225	-25.285	3.490	14.839	1.00	10.48	C
ATOM	1763	CG	LYS	A	225	-24.358	4.174	13.887	1.00	10.33	C
ATOM	1764	CD	LYS	A	225	-24.354	5.652	14.130	1.00	9.50	C
ATOM	1765	CE	LYS	A	225	-25.685	6.266	13.772	1.00	11.24	C
ATOM	1766	NZ	LYS	A	225	-25.779	7.709	14.196	1.00	13.92	N1+
ATOM	1767	N	ALA	A	226	-23.282	0.839	14.171	1.00	21.60	N
ATOM	1768	CA	ALA	A	226	-22.963	-0.387	13.446	1.00	22.74	C
ATOM	1769	C	ALA	A	226	-23.686	-1.533	14.133	1.00	23.96	C
ATOM	1770	O	ALA	A	226	-23.520	-1.710	15.331	1.00	28.06	O
ATOM	1771	CB	ALA	A	226	-21.474	-0.630	13.497	1.00	21.05	C
ATOM	1772	N	GLU	A	227	-24.595	-2.212	13.446	1.00	23.06	N
ATOM	1773	CA	GLU	A	227	-25.267	-3.340	14.079	1.00	20.89	C
ATOM	1774	C	GLU	A	227	-24.243	-4.476	14.307	1.00	18.38	C
ATOM	1775	O	GLU	A	227	-23.142	-4.455	13.748	1.00	11.59	O
ATOM	1776	CB	GLU	A	227	-26.507	-3.810	13.258	1.00	22.63	C
ATOM	1777	CG	GLU	A	227	-27.720	-2.794	13.205	1.00	26.40	C
ATOM	1778	CD	GLU	A	227	-28.944	-3.104	14.146	1.00	29.49	C
ATOM	1779	OE1	GLU	A	227	-28.945	-4.106	14.913	1.00	29.48	O
ATOM	1780	OE2	GLU	A	227	-29.925	-2.315	14.120	1.00	26.69	O1-
ATOM	1781	N	PHE	A	228	-24.524	-5.295	15.321	1.00	19.59	N
ATOM	1782	CA	PHE	A	228	-23.714	-6.468	15.669	1.00	21.16	C
ATOM	1783	C	PHE	A	228	-23.418	-7.380	14.467	1.00	22.82	C
ATOM	1784	O	PHE	A	228	-22.343	-8.002	14.393	1.00	22.75	O
ATOM	1785	CB	PHE	A	228	-24.392	-7.285	16.798	1.00	19.31	C
ATOM	1786	CG	PHE	A	228	-23.470	-8.270	17.481	1.00	15.55	C
ATOM	1787	CD1	PHE	A	228	-22.401	-7.826	18.231	1.00	11.82	C
ATOM	1788	CD2	PHE	A	228	-23.598	-9.636	17.254	1.00	13.74	C
ATOM	1789	CE1	PHE	A	228	-21.482	-8.709	18.727	1.00	11.35	C
ATOM	1790	CE2	PHE	A	228	-22.675	-10.526	17.751	1.00	10.57	C
ATOM	1791	CZ	PHE	A	228	-21.620	-10.067	18.485	1.00	10.53	C
ATOM	1792	N	ALA	A	229	-24.362	-7.472	13.532	1.00	22.31	N
ATOM	1793	CA	ALA	A	229	-24.033	-7.999	12.215	1.00	21.94	C
ATOM	1794	C	ALA	A	229	-22.699	-7.383	11.737	1.00	22.28	C
ATOM	1795	O	ALA	A	229	-21.645	-7.988	11.955	1.00	22.58	O
ATOM	1796	CB	ALA	A	229	-25.157	-7.698	11.238	1.00	18.80	C
ATOM	1797	N	GLU	A	230	-22.718	-6.071	11.494	1.00	23.17	N
ATOM	1798	CA	GLU	A	230	-21.634	-5.373	10.779	1.00	22.57	C

```
ATOM   1799  C   GLU A 230     -20.289  -5.093  11.487  1.00 21.60           C
ATOM   1800  O   GLU A 230     -19.291  -4.855  10.813  1.00 20.95           O
ATOM   1801  CB  GLU A 230     -22.147  -4.061  10.165  1.00 19.48           C
ATOM   1802  CG  GLU A 230     -21.896  -3.975   8.671  1.00 17.15           C
ATOM   1803  CD  GLU A 230     -22.865  -4.813   7.850  1.00 17.55           C
ATOM   1804  OE1 GLU A 230     -23.995  -4.329   7.598  1.00 17.87           O
ATOM   1805  OE2 GLU A 230     -22.488  -5.926   7.409  1.00 15.08           O1-
ATOM   1806  N   VAL A 231     -20.231  -5.073  12.814  1.00 20.69           N
ATOM   1807  CA  VAL A 231     -18.917  -5.007  13.435  1.00 16.38           C
ATOM   1808  C   VAL A 231     -18.287  -6.364  13.136  1.00 20.32           C
ATOM   1809  O   VAL A 231     -17.101  -6.442  12.828  1.00 21.55           O
ATOM   1810  CB  VAL A 231     -18.978  -4.758  14.964  1.00 12.08           C
ATOM   1811  CG1 VAL A 231     -17.812  -5.401  15.645  1.00 11.97           C
ATOM   1812  CG2 VAL A 231     -18.937  -3.285  15.262  1.00 11.69           C
ATOM   1813  N   SER A 232     -19.108  -7.408  13.049  1.00 22.30           N
ATOM   1814  CA  SER A 232     -18.566  -8.749  12.844  1.00 25.21           C
ATOM   1815  C   SER A 232     -18.383  -9.125  11.373  1.00 26.83           C
ATOM   1816  O   SER A 232     -18.337 -10.306  11.009  1.00 28.08           O
ATOM   1817  CB  SER A 232     -19.408  -9.788  13.586  1.00 25.39           C
ATOM   1818  OG  SER A 232     -18.793 -10.070  14.841  1.00 28.11           O
ATOM   1819  N   LYS A 233     -18.260  -8.096  10.536  1.00 26.97           N
ATOM   1820  CA  LYS A 233     -17.661  -8.228   9.225  1.00 23.19           C
ATOM   1821  C   LYS A 233     -16.769  -7.018   9.066  1.00 20.48           C
ATOM   1822  O   LYS A 233     -16.735  -6.421   8.012  1.00 21.98           O
ATOM   1823  CB  LYS A 233     -18.733  -8.254   8.133  1.00 25.56           C
ATOM   1824  CG  LYS A 233     -18.198  -8.687   6.754  1.00 29.24           C
ATOM   1825  CD  LYS A 233     -19.299  -9.170   5.786  1.00 28.84           C
ATOM   1826  CE  LYS A 233     -18.745 -10.207   4.785  1.00 29.50           C
ATOM   1827  NZ  LYS A 233     -19.637 -10.474   3.618  1.00 28.23           N1+
ATOM   1828  N   LEU A 234     -16.191  -6.564  10.174  1.00 19.32           N
ATOM   1829  CA  LEU A 234     -15.119  -5.570  10.155  1.00 19.26           C
ATOM   1830  C   LEU A 234     -13.971  -6.022  11.075  1.00 20.45           C
ATOM   1831  O   LEU A 234     -12.798  -5.927  10.710  1.00 20.91           O
ATOM   1832  CB  LEU A 234     -15.623  -4.191  10.598  1.00 18.55           C
ATOM   1833  CG  LEU A 234     -16.945  -3.619  10.054  1.00 20.65           C
ATOM   1834  CD1 LEU A 234     -17.229  -2.274  10.718  1.00 20.73           C
ATOM   1835  CD2 LEU A 234     -16.900  -3.452   8.536  1.00 18.64           C
ATOM   1836  N   VAL A 235     -14.308  -6.626  12.214  1.00 19.59           N
ATOM   1837  CA  VAL A 235     -13.301  -7.227  13.089  1.00 16.45           C
ATOM   1838  C   VAL A 235     -12.927  -8.677  12.704  1.00 16.23           C
ATOM   1839  O   VAL A 235     -11.848  -9.153  13.054  1.00 17.61           O
ATOM   1840  CB  VAL A 235     -13.756  -7.164  14.556  1.00 14.01           C
ATOM   1841  CG1 VAL A 235     -15.208  -7.513  14.660  1.00 14.30           C
ATOM   1842  CG2 VAL A 235     -12.943  -8.102  15.421  1.00 14.29           C
ATOM   1843  N   THR A 236     -13.808  -9.361  11.976  1.00 13.39           N
ATOM   1844  CA  THR A 236     -13.512 -10.659  11.367  1.00 10.14           C
ATOM   1845  C   THR A 236     -12.673 -10.444  10.102  1.00 12.00           C
ATOM   1846  O   THR A 236     -11.962 -11.342   9.665  1.00 10.48           O
ATOM   1847  CB  THR A 236     -14.803 -11.359  10.949  1.00  9.09           C
ATOM   1848  CG2 THR A 236     -14.960 -12.671  11.657  1.00 10.99           C
ATOM   1849  OG1 THR A 236     -15.914 -10.525  11.281  1.00  5.10           O
ATOM   1850  N   ASP A 237     -12.708  -9.207   9.592  1.00 13.66           N
ATOM   1851  CA  ASP A 237     -12.113  -8.807   8.320  1.00 12.69           C
ATOM   1852  C   ASP A 237     -10.804  -8.086   8.567  1.00 16.23           C
ATOM   1853  O   ASP A 237     -10.160  -7.609   7.622  1.00 17.55           O
ATOM   1854  CB  ASP A 237     -13.052  -7.857   7.581  1.00  9.81           C
ATOM   1855  CG  ASP A 237     -14.195  -8.580   6.879  1.00 15.79           C
ATOM   1856  OD1 ASP A 237     -14.151  -9.824   6.788  1.00 20.11           O
ATOM   1857  OD2 ASP A 237     -15.114  -7.914   6.350  1.00 14.61           O1-
ATOM   1858  N   LEU A 238     -10.504  -7.875   9.849  1.00 17.84           N
ATOM   1859  CA  LEU A 238      -9.376  -7.046  10.284  1.00 17.70           C
ATOM   1860  C   LEU A 238      -8.366  -7.946  10.952  1.00 17.11           C
ATOM   1861  O   LEU A 238      -7.171  -7.656  10.954  1.00 18.24           O
ATOM   1862  CB  LEU A 238      -9.830  -6.016  11.310  1.00 18.48           C
ATOM   1863  CG  LEU A 238      -9.252  -4.614  11.261  1.00 15.02           C
ATOM   1864  CD1 LEU A 238     -10.258  -3.748  10.568  1.00 13.60           C
ATOM   1865  CD2 LEU A 238      -8.989  -4.102  12.650  1.00 11.12           C
ATOM   1866  N   THR A 239      -8.868  -8.928  11.684  1.00 15.12           N
ATOM   1867  CA  THR A 239      -8.031 -10.025  12.104  1.00 15.74           C
ATOM   1868  C   THR A 239      -7.229 -10.453  10.851  1.00 16.69           C
ATOM   1869  O   THR A 239      -6.120  -9.942  10.661  1.00 18.36           O
```

ATOM	1870	CB	THR	A	239	-8.899	-11.180	12.745	1.00	15.86	C
ATOM	1871	CG2	THR	A	239	-8.061	-12.423	13.008	1.00	12.44	C
ATOM	1872	OG1	THR	A	239	-9.476	-10.724	13.988	1.00	7.33	O
ATOM	1873	N	LYS	A	240	-7.899	-11.064	9.873	1.00	15.75	N
ATOM	1874	CA	LYS	A	240	-7.262	-11.521	8.617	1.00	14.50	C
ATOM	1875	C	LYS	A	240	-6.100	-10.649	8.114	1.00	12.60	C
ATOM	1876	O	LYS	A	240	-5.042	-11.164	7.764	1.00	12.12	O
ATOM	1877	CB	LYS	A	240	-8.314	-11.608	7.513	1.00	13.34	C
ATOM	1878	CG	LYS	A	240	-8.390	-12.935	6.845	1.00	16.59	C
ATOM	1879	CD	LYS	A	240	-9.351	-12.874	5.664	1.00	23.14	C
ATOM	1880	CE	LYS	A	240	-8.943	-13.846	4.544	1.00	26.65	C
ATOM	1881	NZ	LYS	A	240	-7.629	-13.487	3.874	1.00	31.21	N1+
ATOM	1882	N	VAL	A	241	-6.361	-9.349	7.966	1.00	12.72	N
ATOM	1883	CA	VAL	A	241	-5.359	-8.348	7.614	1.00	9.66	C
ATOM	1884	C	VAL	A	241	-4.266	-8.481	8.636	1.00	11.94	C
ATOM	1885	O	VAL	A	241	-3.289	-9.155	8.386	1.00	14.55	O
ATOM	1886	CB	VAL	A	241	-5.909	-6.900	7.705	1.00	8.96	C
ATOM	1887	CG1	VAL	A	241	-4.808	-5.903	7.469	1.00	6.95	C
ATOM	1888	CG2	VAL	A	241	-7.030	-6.681	6.719	1.00	7.52	C
ATOM	1889	N	HIS	A	242	-4.541	-8.050	9.858	1.00	11.85	N
ATOM	1890	CA	HIS	A	242	-3.490	-7.939	10.863	1.00	17.50	C
ATOM	1891	C	HIS	A	242	-2.715	-9.236	11.110	1.00	19.56	C
ATOM	1892	O	HIS	A	242	-1.490	-9.212	11.321	1.00	19.10	O
ATOM	1893	CB	HIS	A	242	-4.071	-7.433	12.179	1.00	19.40	C
ATOM	1894	CG	HIS	A	242	-4.033	-5.949	12.311	1.00	17.51	C
ATOM	1895	CD2	HIS	A	242	-3.023	-5.113	12.638	1.00	17.18	C
ATOM	1896	ND1	HIS	A	242	-5.063	-5.146	11.870	1.00	14.59	N
ATOM	1897	CE1	HIS	A	242	-4.680	-3.885	11.896	1.00	14.07	C
ATOM	1898	NE2	HIS	A	242	-3.445	-3.842	12.360	1.00	20.83	N
ATOM	1899	N	THR	A	243	-3.419	-10.364	11.043	1.00	20.29	N
ATOM	1900	CA	THR	A	243	-2.791	-11.653	11.247	1.00	21.66	C
ATOM	1901	C	THR	A	243	-1.638	-11.802	10.259	1.00	22.36	C
ATOM	1902	O	THR	A	243	-0.494	-11.979	10.667	1.00	20.98	O
ATOM	1903	CB	THR	A	243	-3.803	-12.808	11.058	1.00	22.04	C
ATOM	1904	CG2	THR	A	243	-4.560	-13.061	12.338	1.00	19.78	C
ATOM	1905	OG1	THR	A	243	-4.743	-12.465	10.030	1.00	22.04	O
ATOM	1906	N	GLU	A	244	-1.923	-11.503	8.994	1.00	24.92	N
ATOM	1907	CA	GLU	A	244	-0.963	-11.634	7.884	1.00	24.81	C
ATOM	1908	C	GLU	A	244	0.269	-10.751	8.073	1.00	24.55	C
ATOM	1909	O	GLU	A	244	1.365	-11.289	8.316	1.00	25.76	O
ATOM	1910	CB	GLU	A	244	-1.654	-11.320	6.540	1.00	21.04	C
ATOM	1911	CG	GLU	A	244	-2.417	-12.526	5.981	1.00	20.42	C
ATOM	1912	CD	GLU	A	244	-3.749	-12.186	5.369	1.00	16.71	C
ATOM	1913	OE1	GLU	A	244	-3.861	-11.097	4.794	1.00	19.88	O
ATOM	1914	OE2	GLU	A	244	-4.670	-13.029	5.410	1.00	15.80	O1-
ATOM	1915	N	CYS	A	245	0.061	-9.426	8.150	1.00	23.02	N
ATOM	1916	CA	CYS	A	245	1.148	-8.466	8.429	1.00	21.48	C
ATOM	1917	C	CYS	A	245	1.936	-8.829	9.702	1.00	21.49	C
ATOM	1918	O	CYS	A	245	2.965	-8.215	10.000	1.00	20.53	O
ATOM	1919	CB	CYS	A	245	0.604	-7.039	8.577	1.00	20.81	C
ATOM	1920	SG	CYS	A	245	0.340	-6.068	7.058	1.00	19.72	S
ATOM	1921	N	CYS	A	246	1.434	-9.803	10.458	1.00	20.66	N
ATOM	1922	CA	CYS	A	246	2.133	-10.288	11.635	1.00	22.93	C
ATOM	1923	C	CYS	A	246	2.810	-11.634	11.342	1.00	23.54	C
ATOM	1924	O	CYS	A	246	4.001	-11.829	11.613	1.00	23.49	O
ATOM	1925	CB	CYS	A	246	1.150	-10.431	12.828	1.00	23.38	C
ATOM	1926	SG	CYS	A	246	0.340	-8.909	13.476	1.00	21.26	S
ATOM	1927	N	HIS	A	247	2.054	-12.531	10.717	1.00	26.98	N
ATOM	1928	CA	HIS	A	247	2.461	-13.924	10.463	1.00	28.41	C
ATOM	1929	C	HIS	A	247	3.551	-14.018	9.371	1.00	27.16	C
ATOM	1930	O	HIS	A	247	3.999	-15.107	8.996	1.00	24.90	O
ATOM	1931	CB	HIS	A	247	1.221	-14.734	10.034	1.00	30.64	C
ATOM	1932	CG	HIS	A	247	0.887	-15.871	10.953	1.00	33.09	C
ATOM	1933	CD2	HIS	A	247	1.675	-16.666	11.718	1.00	31.19	C
ATOM	1934	ND1	HIS	A	247	-0.397	-16.372	11.077	1.00	32.64	N
ATOM	1935	CE1	HIS	A	247	-0.383	-17.423	11.881	1.00	32.18	C
ATOM	1936	NE2	HIS	A	247	0.859	-17.616	12.285	1.00	34.75	N
ATOM	1937	N	GLY	A	248	3.882	-12.875	8.788	1.00	26.19	N
ATOM	1938	CA	GLY	A	248	5.010	-12.822	7.893	1.00	24.62	C
ATOM	1939	C	GLY	A	248	4.560	-12.235	6.584	1.00	24.54	C
ATOM	1940	O	GLY	A	248	5.066	-11.200	6.176	1.00	25.55	O

```
ATOM   1941  N   ASP A 249       3.553 -12.847   5.966  1.00 23.88           N
ATOM   1942  CA  ASP A 249       3.154 -12.470   4.619  1.00 20.47           C
ATOM   1943  C   ASP A 249       2.317 -11.215   4.621  1.00 20.76           C
ATOM   1944  O   ASP A 249       1.132 -11.257   4.957  1.00 21.26           O
ATOM   1945  CB  ASP A 249       2.408 -13.612   3.903  1.00 20.40           C
ATOM   1946  CG  ASP A 249       1.351 -14.282   4.764  1.00 18.24           C
ATOM   1947  OD1 ASP A 249       1.703 -15.199   5.537  1.00 19.57           O
ATOM   1948  OD2 ASP A 249       0.152 -14.040   4.523  1.00 17.76           O1-
ATOM   1949  N   LEU A 250       3.003 -10.087   4.423  1.00 20.08           N
ATOM   1950  CA  LEU A 250       2.373  -8.789   4.180  1.00 20.75           C
ATOM   1951  C   LEU A 250       1.422  -8.841   2.956  1.00 23.97           C
ATOM   1952  O   LEU A 250       0.246  -9.180   3.084  1.00 24.23           O
ATOM   1953  CB  LEU A 250       3.427  -7.715   3.892  1.00 18.42           C
ATOM   1954  CG  LEU A 250       4.765  -7.394   4.537  1.00 17.52           C
ATOM   1955  CD1 LEU A 250       5.333  -8.472   5.381  1.00 20.69           C
ATOM   1956  CD2 LEU A 250       5.689  -7.106   3.393  1.00 20.41           C
ATOM   1957  N   LEU A 251       1.964  -8.473   1.788  1.00 24.70           N
ATOM   1958  CA  LEU A 251       1.273  -8.329   0.499  1.00 21.99           C
ATOM   1959  C   LEU A 251      -0.227  -8.608   0.403  1.00 22.01           C
ATOM   1960  O   LEU A 251      -0.983  -7.834  -0.179  1.00 19.96           O
ATOM   1961  CB  LEU A 251       2.016  -9.161  -0.540  1.00 22.73           C
ATOM   1962  CG  LEU A 251       3.503  -8.825  -0.674  1.00 21.62           C
ATOM   1963  CD1 LEU A 251       4.302  -9.609   0.354  1.00 22.59           C
ATOM   1964  CD2 LEU A 251       3.972  -9.145  -2.085  1.00 20.79           C
ATOM   1965  N   GLU A 252      -0.633  -9.779   0.856  1.00 23.52           N
ATOM   1966  CA  GLU A 252      -2.053 -10.078   0.994  1.00 24.95           C
ATOM   1967  C   GLU A 252      -2.699  -8.956   1.840  1.00 25.20           C
ATOM   1968  O   GLU A 252      -3.583  -8.247   1.364  1.00 26.85           O
ATOM   1969  CB  GLU A 252      -2.225 -11.463   1.648  1.00 23.50           C
ATOM   1970  CG  GLU A 252      -1.702 -12.659   0.793  1.00 23.59           C
ATOM   1971  CD  GLU A 252      -0.211 -12.968   0.966  1.00 22.46           C
ATOM   1972  OE1 GLU A 252       0.548 -12.064   1.372  1.00 21.42           O
ATOM   1973  OE2 GLU A 252       0.212 -14.112   0.667  1.00 20.54           O1-
ATOM   1974  N   CYS A 253      -2.113  -8.683   3.002  1.00 23.06           N
ATOM   1975  CA  CYS A 253      -2.418  -7.509   3.825  1.00 21.89           C
ATOM   1976  C   CYS A 253      -2.879  -6.277   3.017  1.00 22.51           C
ATOM   1977  O   CYS A 253      -4.077  -5.982   2.928  1.00 18.60           O
ATOM   1978  CB  CYS A 253      -1.171  -7.153   4.662  1.00 20.32           C
ATOM   1979  SG  CYS A 253      -1.466  -6.653   6.385  1.00 15.33           S
ATOM   1980  N   ALA A 254      -1.921  -5.638   2.345  1.00 22.49           N
ATOM   1981  CA  ALA A 254      -2.105  -4.310   1.755  1.00 22.36           C
ATOM   1982  C   ALA A 254      -3.233  -4.310   0.727  1.00 22.41           C
ATOM   1983  O   ALA A 254      -3.812  -3.264   0.430  1.00 20.73           O
ATOM   1984  CB  ALA A 254      -0.785  -3.836   1.104  1.00 20.82           C
ATOM   1985  N   ASP A 255      -3.433  -5.475   0.108  1.00 23.61           N
ATOM   1986  CA  ASP A 255      -4.448  -5.684  -0.918  1.00 23.62           C
ATOM   1987  C   ASP A 255      -5.725  -5.744  -0.112  1.00 24.73           C
ATOM   1988  O   ASP A 255      -6.638  -4.941  -0.353  1.00 25.80           O
ATOM   1989  CB  ASP A 255      -4.205  -7.026  -1.648  1.00 24.09           C
ATOM   1990  CG  ASP A 255      -5.128  -7.234  -2.858  1.00 26.69           C
ATOM   1991  OD1 ASP A 255      -5.894  -6.309  -3.200  1.00 28.50           O
ATOM   1992  OD2 ASP A 255      -5.063  -8.310  -3.507  1.00 25.31           O1-
ATOM   1993  N   ASP A 256      -5.645  -6.478   1.009  1.00 24.05           N
ATOM   1994  CA  ASP A 256      -6.778  -6.758   1.893  1.00 21.53           C
ATOM   1995  C   ASP A 256      -7.408  -5.507   2.499  1.00 22.11           C
ATOM   1996  O   ASP A 256      -8.603  -5.319   2.331  1.00 21.08           O
ATOM   1997  CB  ASP A 256      -6.375  -7.720   3.009  1.00 19.32           C
ATOM   1998  CG  ASP A 256      -6.474  -9.182   2.594  1.00 21.17           C
ATOM   1999  OD1 ASP A 256      -7.390  -9.548   1.814  1.00 17.48           O
ATOM   2000  OD2 ASP A 256      -5.650  -9.984   3.094  1.00 19.56           O1-
ATOM   2001  N   ARG A 257      -6.624  -4.584   3.069  1.00 22.19           N
ATOM   2002  CA  ARG A 257      -7.214  -3.327   3.566  1.00 22.80           C
ATOM   2003  C   ARG A 257      -7.861  -2.446   2.497  1.00 23.33           C
ATOM   2004  O   ARG A 257      -8.179  -1.277   2.756  1.00 24.22           O
ATOM   2005  CB  ARG A 257      -6.212  -2.474   4.344  1.00 23.48           C
ATOM   2006  CG  ARG A 257      -6.912  -1.398   5.220  1.00 26.53           C
ATOM   2007  CD  ARG A 257      -6.367  -1.389   6.660  1.00 32.02           C
ATOM   2008  NE  ARG A 257      -7.343  -1.126   7.730  1.00 29.83           N
ATOM   2009  CZ  ARG A 257      -6.970  -0.900   8.990  1.00 29.33           C
ATOM   2010  NH1 ARG A 257      -5.677  -0.815   9.258  1.00 31.19           N1+
ATOM   2011  NH2 ARG A 257      -7.857  -0.700   9.970  1.00 27.88           N
```

ATOM	2012	N	ALA	A	258	-8.016	-2.989	1.288	1.00	23.60	N
ATOM	2013	CA	ALA	A	258	-8.843	-2.381	0.247	1.00	19.06	C
ATOM	2014	C	ALA	A	258	-9.853	-3.397	-0.273	1.00	17.21	C
ATOM	2015	O	ALA	A	258	-11.020	-3.095	-0.345	1.00	16.88	O
ATOM	2016	CB	ALA	A	258	-7.989	-1.870	-0.849	1.00	17.23	C
ATOM	2017	N	ASP	A	259	-9.464	-4.662	-0.292	1.00	18.75	N
ATOM	2018	CA	ASP	A	259	-10.430	-5.768	-0.205	1.00	21.08	C
ATOM	2019	C	ASP	A	259	-11.253	-5.577	1.090	1.00	19.96	C
ATOM	2020	O	ASP	A	259	-12.159	-6.365	1.380	1.00	20.42	O
ATOM	2021	CB	ASP	A	259	-9.690	-7.150	-0.180	1.00	24.22	C
ATOM	2022	CG	ASP	A	259	-10.169	-8.150	-1.288	1.00	21.26	C
ATOM	2023	OD1	ASP	A	259	-11.075	-7.821	-2.076	1.00	21.43	O
ATOM	2024	OD2	ASP	A	259	-9.634	-9.279	-1.364	1.00	17.32	O1-
ATOM	2025	N	LEU	A	260	-10.848	-4.617	1.924	1.00	18.38	N
ATOM	2026	CA	LEU	A	260	-11.732	-4.087	2.948	1.00	19.05	C
ATOM	2027	C	LEU	A	260	-12.198	-2.692	2.561	1.00	20.78	C
ATOM	2028	O	LEU	A	260	-13.257	-2.581	1.950	1.00	22.27	O
ATOM	2029	CB	LEU	A	260	-11.087	-4.076	4.334	1.00	18.98	C
ATOM	2030	CG	LEU	A	260	-11.986	-4.337	5.564	1.00	15.64	C
ATOM	2031	CD1	LEU	A	260	-11.913	-3.163	6.525	1.00	14.71	C
ATOM	2032	CD2	LEU	A	260	-13.418	-4.561	5.153	1.00	12.68	C
ATOM	2033	N	ALA	A	261	-11.398	-1.651	2.801	1.00	19.14	N
ATOM	2034	CA	ALA	A	261	-11.884	-0.274	2.617	1.00	20.29	C
ATOM	2035	C	ALA	A	261	-12.836	-0.069	1.445	1.00	20.76	C
ATOM	2036	O	ALA	A	261	-13.748	0.727	1.551	1.00	23.76	O
ATOM	2037	CB	ALA	A	261	-10.738	0.703	2.506	1.00	20.41	C
ATOM	2038	N	LYS	A	262	-12.715	-0.870	0.390	1.00	21.67	N
ATOM	2039	CA	LYS	A	262	-13.724	-0.914	-0.678	1.00	22.13	C
ATOM	2040	C	LYS	A	262	-14.858	-1.897	-0.338	1.00	22.91	C
ATOM	2041	O	LYS	A	262	-15.231	-2.735	-1.178	1.00	24.14	O
ATOM	2042	CB	LYS	A	262	-13.079	-1.319	-2.018	1.00	21.14	C
ATOM	2043	CG	LYS	A	262	-12.745	-0.182	-2.958	1.00	20.45	C
ATOM	2044	CD	LYS	A	262	-13.871	0.032	-3.965	1.00	22.63	C
ATOM	2045	CE	LYS	A	262	-13.416	0.915	-5.138	1.00	22.50	C
ATOM	2046	NZ	LYS	A	262	-13.721	0.318	-6.475	1.00	18.96	N1+
ATOM	2047	N	TYR	A	263	-15.279	-1.881	0.933	1.00	20.93	N
ATOM	2048	CA	TYR	A	263	-16.547	-2.447	1.416	1.00	15.95	C
ATOM	2049	C	TYR	A	263	-17.010	-1.449	2.439	1.00	14.10	C
ATOM	2050	O	TYR	A	263	-17.949	-0.720	2.178	1.00	16.17	O
ATOM	2051	CB	TYR	A	263	-16.349	-3.803	2.096	1.00	16.93	C
ATOM	2052	CG	TYR	A	263	-17.327	-4.107	3.212	1.00	14.41	C
ATOM	2053	CD1	TYR	A	263	-18.595	-4.556	2.931	1.00	17.78	C
ATOM	2054	CD2	TYR	A	263	-17.055	-3.743	4.505	1.00	15.15	C
ATOM	2055	CE1	TYR	A	263	-19.577	-4.581	3.889	1.00	17.72	C
ATOM	2056	CE2	TYR	A	263	-18.025	-3.763	5.472	1.00	16.35	C
ATOM	2057	CZ	TYR	A	263	-19.294	-4.157	5.153	1.00	17.14	C
ATOM	2058	OH	TYR	A	263	-20.326	-3.914	6.025	1.00	18.82	O
ATOM	2059	N	ILE	A	264	-16.205	-1.275	3.488	1.00	13.60	N
ATOM	2060	CA	ILE	A	264	-16.396	-0.224	4.502	1.00	13.72	C
ATOM	2061	C	ILE	A	264	-16.916	0.992	3.801	1.00	14.67	C
ATOM	2062	O	ILE	A	264	-18.082	1.333	3.943	1.00	16.24	O
ATOM	2063	CB	ILE	A	264	-15.058	0.192	5.230	1.00	11.33	C
ATOM	2064	CG1	ILE	A	264	-14.910	-0.527	6.568	1.00	9.86	C
ATOM	2065	CG2	ILE	A	264	-15.057	1.676	5.551	1.00	12.03	C
ATOM	2066	CD1	ILE	A	264	-13.924	0.134	7.518	1.00	8.24	C
ATOM	2067	N	CYS	A	265	-16.146	1.431	2.817	1.00	18.20	N
ATOM	2068	CA	CYS	A	265	-16.409	2.706	2.184	1.00	21.00	C
ATOM	2069	C	CYS	A	265	-17.183	2.683	0.863	1.00	20.34	C
ATOM	2070	O	CYS	A	265	-17.164	3.648	0.104	1.00	18.15	O
ATOM	2071	CB	CYS	A	265	-15.131	3.508	2.016	1.00	18.44	C
ATOM	2072	SG	CYS	A	265	-15.717	5.147	1.584	1.00	24.42	S
ATOM	2073	N	GLU	A	266	-17.927	1.612	0.630	1.00	22.55	N
ATOM	2074	CA	GLU	A	266	-19.044	1.668	-0.305	1.00	25.71	C
ATOM	2075	C	GLU	A	266	-20.359	1.649	0.507	1.00	25.99	C
ATOM	2076	O	GLU	A	266	-21.401	2.132	0.031	1.00	24.57	O
ATOM	2077	CB	GLU	A	266	-18.982	0.486	-1.306	1.00	30.26	C
ATOM	2078	CG	GLU	A	266	-18.126	0.746	-2.589	1.00	33.40	C
ATOM	2079	CD	GLU	A	266	-18.430	-0.224	-3.755	1.00	33.38	C
ATOM	2080	OE1	GLU	A	266	-19.340	0.064	-4.569	1.00	34.65	O
ATOM	2081	OE2	GLU	A	266	-17.702	-1.226	-3.918	1.00	31.65	O1-
ATOM	2082	N	ASN	A	267	-20.231	1.304	1.792	1.00	24.11	N

ATOM	2083	CA	ASN	A	267	-21.367	1.133	2.693	1.00	24.75	C
ATOM	2084	C	ASN	A	267	-21.562	2.304	3.675	1.00	25.96	C
ATOM	2085	O	ASN	A	267	-22.551	2.367	4.425	1.00	24.17	O
ATOM	2086	CB	ASN	A	267	-21.201	-0.171	3.465	1.00	25.26	C
ATOM	2087	CG	ASN	A	267	-21.552	-1.388	2.630	1.00	26.30	C
ATOM	2088	ND2	ASN	A	267	-21.767	-2.527	3.293	1.00	23.38	N
ATOM	2089	OD1	ASN	A	267	-21.602	-1.314	1.394	1.00	28.38	O
ATOM	2090	N	GLN	A	268	-20.543	3.150	3.756	1.00	27.51	N
ATOM	2091	CA	GLN	A	268	-20.619	4.455	4.422	1.00	26.43	C
ATOM	2092	C	GLN	A	268	-21.731	4.680	5.469	1.00	25.52	C
ATOM	2093	O	GLN	A	268	-21.432	5.026	6.604	1.00	23.74	O
ATOM	2094	CB	GLN	A	268	-20.693	5.552	3.354	1.00	24.02	C
ATOM	2095	CG	GLN	A	268	-19.418	6.340	3.149	1.00	20.44	C
ATOM	2096	CD	GLN	A	268	-19.711	7.673	2.493	1.00	23.29	C
ATOM	2097	NE2	GLN	A	268	-18.762	8.607	2.568	1.00	22.63	N
ATOM	2098	OE1	GLN	A	268	-20.807	7.873	1.949	1.00	24.24	O
ATOM	2099	N	ASP	A	269	-22.993	4.648	5.054	1.00	25.35	N
ATOM	2100	CA	ASP	A	269	-24.064	5.203	5.882	1.00	26.24	C
ATOM	2101	C	ASP	A	269	-24.667	4.170	6.833	1.00	28.27	C
ATOM	2102	O	ASP	A	269	-25.243	4.533	7.873	1.00	29.26	O
ATOM	2103	CB	ASP	A	269	-25.180	5.803	5.009	1.00	27.91	C
ATOM	2104	CG	ASP	A	269	-25.889	4.753	4.153	1.00	29.64	C
ATOM	2105	OD1	ASP	A	269	-25.178	3.900	3.565	1.00	30.12	O
ATOM	2106	OD2	ASP	A	269	-27.151	4.754	4.103	1.00	29.00	O1-
ATOM	2107	N	SER	A	270	-24.536	2.886	6.501	1.00	27.48	N
ATOM	2108	CA	SER	A	270	-24.993	1.847	7.413	1.00	26.75	C
ATOM	2109	C	SER	A	270	-23.949	1.519	8.504	1.00	26.55	C
ATOM	2110	O	SER	A	270	-24.139	0.582	9.286	1.00	26.34	O
ATOM	2111	CB	SER	A	270	-25.359	0.589	6.623	1.00	28.19	C
ATOM	2112	OG	SER	A	270	-24.224	0.037	5.957	1.00	30.87	O
ATOM	2113	N	ILE	A	271	-22.895	2.329	8.614	1.00	25.27	N
ATOM	2114	CA	ILE	A	271	-21.741	1.967	9.420	1.00	24.11	C
ATOM	2115	C	ILE	A	271	-21.253	3.034	10.393	1.00	25.93	C
ATOM	2116	O	ILE	A	271	-21.228	2.784	11.593	1.00	27.16	O
ATOM	2117	CB	ILE	A	271	-20.578	1.539	8.535	1.00	25.05	C
ATOM	2118	CG1	ILE	A	271	-20.832	0.113	8.013	1.00	27.97	C
ATOM	2119	CG2	ILE	A	271	-19.278	1.600	9.305	1.00	24.66	C
ATOM	2120	CD1	ILE	A	271	-19.661	-0.523	7.232	1.00	23.58	C
ATOM	2121	N	SER	A	272	-20.749	4.167	9.908	1.00	27.76	N
ATOM	2122	CA	SER	A	272	-20.329	5.238	10.836	1.00	28.74	C
ATOM	2123	C	SER	A	272	-20.165	6.631	10.227	1.00	27.74	C
ATOM	2124	O	SER	A	272	-19.123	7.259	10.418	1.00	26.72	O
ATOM	2125	CB	SER	A	272	-19.018	4.837	11.530	1.00	30.16	C
ATOM	2126	OG	SER	A	272	-18.380	5.901	12.239	1.00	27.56	O
ATOM	2127	N	SER	A	273	-21.257	7.182	9.701	1.00	27.40	N
ATOM	2128	CA	SER	A	273	-21.236	8.458	8.970	1.00	26.73	C
ATOM	2129	C	SER	A	273	-19.842	9.066	8.643	1.00	25.15	C
ATOM	2130	O	SER	A	273	-19.475	9.204	7.468	1.00	20.82	O
ATOM	2131	CB	SER	A	273	-22.110	9.495	9.714	1.00	25.04	C
ATOM	2132	OG	SER	A	273	-21.417	10.725	9.872	1.00	24.31	O
ATOM	2133	N	LYS	A	274	-19.104	9.433	9.697	1.00	24.05	N
ATOM	2134	CA	LYS	A	274	-17.875	10.230	9.625	1.00	24.74	C
ATOM	2135	C	LYS	A	274	-16.764	9.750	8.705	1.00	22.58	C
ATOM	2136	O	LYS	A	274	-15.721	10.376	8.669	1.00	21.30	O
ATOM	2137	CB	LYS	A	274	-17.260	10.431	11.024	1.00	25.80	C
ATOM	2138	CG	LYS	A	274	-18.220	10.937	12.072	1.00	29.40	C
ATOM	2139	CD	LYS	A	274	-18.241	12.437	12.131	1.00	33.71	C
ATOM	2140	CE	LYS	A	274	-18.315	12.928	13.589	1.00	34.96	C
ATOM	2141	NZ	LYS	A	274	-17.356	14.082	13.815	1.00	34.17	N1+
ATOM	2142	N	LEU	A	275	-16.941	8.657	7.970	1.00	23.57	N
ATOM	2143	CA	LEU	A	275	-15.937	8.325	6.959	1.00	23.18	C
ATOM	2144	C	LEU	A	275	-16.330	8.834	5.585	1.00	22.54	C
ATOM	2145	O	LEU	A	275	-16.224	8.104	4.610	1.00	21.30	O
ATOM	2146	CB	LEU	A	275	-15.652	6.828	6.872	1.00	21.39	C
ATOM	2147	CG	LEU	A	275	-16.319	5.772	7.741	1.00	23.35	C
ATOM	2148	CD1	LEU	A	275	-17.580	5.221	7.072	1.00	23.95	C
ATOM	2149	CD2	LEU	A	275	-15.319	4.663	7.986	1.00	22.41	C
ATOM	2150	N	LYS	A	276	-16.828	10.073	5.514	1.00	25.95	N
ATOM	2151	CA	LYS	A	276	-16.886	10.830	4.247	1.00	26.73	C
ATOM	2152	C	LYS	A	276	-15.478	11.187	3.799	1.00	25.18	C
ATOM	2153	O	LYS	A	276	-14.865	10.434	3.052	1.00	25.22	O

ATOM	2154	CB	LYS	A	276	-17.710	12.129	4.402	1.00	29.83	C
ATOM	2155	CG	LYS	A	276	-17.702	12.805	5.801	1.00	30.45	C
ATOM	2156	CD	LYS	A	276	-16.755	14.005	5.874	1.00	30.09	C
ATOM	2157	CE	LYS	A	276	-16.974	14.966	4.712	1.00	31.30	C
ATOM	2158	NZ	LYS	A	276	-18.160	15.877	4.882	1.00	29.48	N1+
ATOM	2159	N	GLU	A	277	-14.899	12.172	4.482	1.00	25.27	N
ATOM	2160	CA	GLU	A	277	-13.552	12.674	4.211	1.00	26.60	C
ATOM	2161	C	GLU	A	277	-12.640	11.674	3.545	1.00	25.74	C
ATOM	2162	O	GLU	A	277	-12.369	11.752	2.348	1.00	25.45	O
ATOM	2163	CB	GLU	A	277	-12.899	13.131	5.511	1.00	26.87	C
ATOM	2164	CG	GLU	A	277	-13.175	14.597	5.841	1.00	31.72	C
ATOM	2165	CD	GLU	A	277	-12.559	15.583	4.830	1.00	30.45	C
ATOM	2166	OE1	GLU	A	277	-13.198	15.870	3.787	1.00	27.15	O
ATOM	2167	OE2	GLU	A	277	-11.473	16.133	5.136	1.00	30.36	O1-
ATOM	2168	N	CYS	A	278	-12.396	10.601	4.270	1.00	26.80	N
ATOM	2169	CA	CYS	A	278	-11.438	9.582	3.859	1.00	27.12	C
ATOM	2170	C	CYS	A	278	-12.143	8.579	2.975	1.00	23.38	C
ATOM	2171	O	CYS	A	278	-12.167	7.391	3.233	1.00	24.65	O
ATOM	2172	CB	CYS	A	278	-10.871	8.920	5.104	1.00	31.72	C
ATOM	2173	SG	CYS	A	278	-11.643	9.660	6.586	1.00	33.14	S
ATOM	2174	N	CYS	A	279	-12.574	9.086	1.842	1.00	21.92	N
ATOM	2175	CA	CYS	A	279	-13.315	8.339	0.856	1.00	20.79	C
ATOM	2176	C	CYS	A	279	-13.460	9.359	-0.255	1.00	21.27	C
ATOM	2177	O	CYS	A	279	-13.962	9.073	-1.350	1.00	22.78	O
ATOM	2178	CB	CYS	A	279	-14.661	7.929	1.439	1.00	19.99	C
ATOM	2179	SG	CYS	A	279	-14.510	6.478	2.522	1.00	21.12	S
ATOM	2180	N	GLU	A	280	-13.143	10.594	0.107	1.00	18.93	N
ATOM	2181	CA	GLU	A	280	-12.521	11.517	-0.799	1.00	19.87	C
ATOM	2182	C	GLU	A	280	-11.046	11.586	-0.419	1.00	21.37	C
ATOM	2183	O	GLU	A	280	-10.570	12.645	-0.028	1.00	22.62	O
ATOM	2184	CB	GLU	A	280	-13.152	12.896	-0.661	1.00	19.56	C
ATOM	2185	CG	GLU	A	280	-14.562	12.867	-0.153	1.00	22.77	C
ATOM	2186	CD	GLU	A	280	-15.551	12.438	-1.207	1.00	24.09	C
ATOM	2187	OE1	GLU	A	280	-15.638	11.225	-1.511	1.00	22.38	O
ATOM	2188	OE2	GLU	A	280	-16.260	13.325	-1.723	1.00	26.29	O1-
ATOM	2189	N	LYS	A	281	-10.362	10.437	-0.413	1.00	21.75	N
ATOM	2190	CA	LYS	A	281	-8.896	10.399	-0.281	1.00	22.31	C
ATOM	2191	C	LYS	A	281	-8.246	9.170	-0.941	1.00	23.66	C
ATOM	2192	O	LYS	A	281	-8.367	8.041	-0.431	1.00	25.63	O
ATOM	2193	CB	LYS	A	281	-8.474	10.459	1.188	1.00	19.47	C
ATOM	2194	CG	LYS	A	281	-8.113	11.852	1.682	1.00	21.76	C
ATOM	2195	CD	LYS	A	281	-8.906	12.198	2.952	1.00	25.79	C
ATOM	2196	CE	LYS	A	281	-8.282	13.351	3.761	1.00	27.84	C
ATOM	2197	NZ	LYS	A	281	-7.158	12.950	4.670	1.00	23.24	N1+
ATOM	2198	N	PRO	A	282	-7.451	9.396	-2.015	1.00	20.78	N
ATOM	2199	CA	PRO	A	282	-6.604	8.401	-2.683	1.00	19.52	C
ATOM	2200	C	PRO	A	282	-6.214	7.216	-1.818	1.00	19.87	C
ATOM	2201	O	PRO	A	282	-5.362	7.365	-0.939	1.00	19.82	O
ATOM	2202	CB	PRO	A	282	-5.376	9.210	-3.100	1.00	16.20	C
ATOM	2203	CG	PRO	A	282	-5.586	10.592	-2.507	1.00	15.71	C
ATOM	2204	CD	PRO	A	282	-7.052	10.740	-2.438	1.00	17.15	C
ATOM	2205	N	LEU	A	283	-6.642	6.027	-2.254	1.00	20.57	N
ATOM	2206	CA	LEU	A	283	-6.610	4.787	-1.461	1.00	20.98	C
ATOM	2207	C	LEU	A	283	-5.739	4.913	-0.252	1.00	20.62	C
ATOM	2208	O	LEU	A	283	-6.176	5.479	0.723	1.00	18.64	O
ATOM	2209	CB	LEU	A	283	-6.147	3.585	-2.288	1.00	22.23	C
ATOM	2210	CG	LEU	A	283	-6.130	2.292	-1.452	1.00	23.90	C
ATOM	2211	CD1	LEU	A	283	-7.217	1.299	-1.964	1.00	22.21	C
ATOM	2212	CD2	LEU	A	283	-4.730	1.673	-1.500	1.00	20.45	C
ATOM	2213	N	LEU	A	284	-4.471	4.536	-0.395	1.00	22.99	N
ATOM	2214	CA	LEU	A	284	-3.396	4.918	0.519	1.00	26.73	C
ATOM	2215	C	LEU	A	284	-3.912	5.607	1.803	1.00	28.33	C
ATOM	2216	O	LEU	A	284	-4.326	4.906	2.739	1.00	28.88	O
ATOM	2217	CB	LEU	A	284	-2.385	5.825	-0.225	1.00	28.31	C
ATOM	2218	CG	LEU	A	284	-1.765	5.434	-1.595	1.00	26.94	C
ATOM	2219	CD1	LEU	A	284	-1.303	6.690	-2.364	1.00	20.85	C
ATOM	2220	CD2	LEU	A	284	-0.594	4.469	-1.384	1.00	26.30	C
ATOM	2221	N	GLU	A	285	-4.223	6.906	1.683	1.00	28.79	N
ATOM	2222	CA	GLU	A	285	-4.614	7.759	2.825	1.00	28.34	C
ATOM	2223	C	GLU	A	285	-6.001	7.475	3.396	1.00	26.33	C
ATOM	2224	O	GLU	A	285	-6.293	7.857	4.516	1.00	28.65	O

```
ATOM   2225  CB  GLU A 285      -4.542   9.247   2.462  1.00 28.57           C
ATOM   2226  CG  GLU A 285      -3.144   9.754   2.142  1.00 29.88           C
ATOM   2227  CD  GLU A 285      -3.105  10.581   0.870  1.00 30.17           C
ATOM   2228  OE1 GLU A 285      -3.483  11.778   0.920  1.00 29.25           O
ATOM   2229  OE2 GLU A 285      -2.744  10.009  -0.185  1.00 28.46           O1-
ATOM   2230  N   LYS A 286      -6.880   6.870   2.617  1.00 26.40           N
ATOM   2231  CA  LYS A 286      -8.030   6.193   3.204  1.00 26.58           C
ATOM   2232  C   LYS A 286      -7.525   5.430   4.440  1.00 24.48           C
ATOM   2233  O   LYS A 286      -7.732   5.868   5.567  1.00 25.77           O
ATOM   2234  CB  LYS A 286      -8.650   5.179   2.218  1.00 27.97           C
ATOM   2235  CG  LYS A 286      -9.914   5.578   1.425  1.00 19.93           C
ATOM   2236  CD  LYS A 286     -10.135   4.498   0.364  1.00 19.31           C
ATOM   2237  CE  LYS A 286     -11.548   4.386  -0.099  1.00 16.08           C
ATOM   2238  NZ  LYS A 286     -12.013   5.632  -0.734  1.00 16.67           N1+
ATOM   2239  N   SER A 287      -6.716   4.406   4.244  1.00 19.71           N
ATOM   2240  CA  SER A 287      -6.403   3.545   5.359  1.00 19.25           C
ATOM   2241  C   SER A 287      -5.478   4.164   6.423  1.00 19.83           C
ATOM   2242  O   SER A 287      -4.917   3.451   7.252  1.00 19.56           O
ATOM   2243  CB  SER A 287      -5.832   2.230   4.832  1.00 22.16           C
ATOM   2244  OG  SER A 287      -6.709   1.646   3.874  1.00 24.16           O
ATOM   2245  N   HIS A 288      -5.335   5.486   6.427  1.00 20.41           N
ATOM   2246  CA  HIS A 288      -4.825   6.194   7.611  1.00 23.21           C
ATOM   2247  C   HIS A 288      -5.564   7.515   7.785  1.00 24.49           C
ATOM   2248  O   HIS A 288      -4.955   8.582   7.929  1.00 24.42           O
ATOM   2249  CB  HIS A 288      -3.315   6.446   7.531  1.00 23.44           C
ATOM   2250  CG  HIS A 288      -2.712   6.985   8.800  1.00 24.13           C
ATOM   2251  CD2 HIS A 288      -2.834   8.190   9.411  1.00 22.19           C
ATOM   2252  ND1 HIS A 288      -1.809   6.267   9.556  1.00 25.67           N
ATOM   2253  CE1 HIS A 288      -1.401   7.001  10.576  1.00 23.98           C
ATOM   2254  NE2 HIS A 288      -2.009   8.172  10.512  1.00 26.26           N
ATOM   2255  N   CYS A 289      -6.865   7.447   7.540  1.00 25.16           N
ATOM   2256  CA  CYS A 289      -7.819   8.301   8.209  1.00 26.57           C
ATOM   2257  C   CYS A 289      -8.701   7.377   9.055  1.00 25.76           C
ATOM   2258  O   CYS A 289      -9.060   7.710  10.192  1.00 26.10           O
ATOM   2259  CB  CYS A 289      -8.716   9.020   7.218  1.00 26.87           C
ATOM   2260  SG  CYS A 289     -10.336   9.115   8.027  1.00 35.83           S
ATOM   2261  N   ILE A 290      -9.231   6.354   8.381  1.00 23.01           N
ATOM   2262  CA  ILE A 290      -9.982   5.262   8.996  1.00 20.80           C
ATOM   2263  C   ILE A 290      -9.125   4.551  10.043  1.00 21.11           C
ATOM   2264  O   ILE A 290      -9.551   4.335  11.168  1.00 22.48           O
ATOM   2265  CB  ILE A 290     -10.432   4.241   7.920  1.00 17.33           C
ATOM   2266  CG1 ILE A 290     -11.770   4.670   7.312  1.00 16.61           C
ATOM   2267  CG2 ILE A 290     -10.558   2.866   8.514  1.00 19.50           C
ATOM   2268  CD1 ILE A 290     -11.867   4.462   5.808  1.00 11.81           C
ATOM   2269  N   ALA A 291      -7.920   4.156   9.665  1.00 20.66           N
ATOM   2270  CA  ALA A 291      -6.948   3.747  10.664  1.00 20.97           C
ATOM   2271  C   ALA A 291      -6.893   4.911  11.619  1.00 19.91           C
ATOM   2272  O   ALA A 291      -7.270   6.008  11.249  1.00 18.39           O
ATOM   2273  CB  ALA A 291      -5.583   3.518  10.024  1.00 21.17           C
ATOM   2274  N   GLU A 292      -6.302   4.720  12.784  1.00 21.16           N
ATOM   2275  CA  GLU A 292      -6.572   5.631  13.881  1.00 24.55           C
ATOM   2276  C   GLU A 292      -6.249   7.130  13.695  1.00 26.59           C
ATOM   2277  O   GLU A 292      -5.185   7.622  14.100  1.00 27.80           O
ATOM   2278  CB  GLU A 292      -5.962   5.076  15.170  1.00 24.02           C
ATOM   2279  CG  GLU A 292      -6.808   3.952  15.736  1.00 23.17           C
ATOM   2280  CD  GLU A 292      -6.260   3.354  17.010  1.00 25.80           C
ATOM   2281  OE1 GLU A 292      -6.050   4.132  17.989  1.00 23.17           O
ATOM   2282  OE2 GLU A 292      -6.112   2.098  17.040  1.00 20.76           O1-
ATOM   2283  N   VAL A 293      -7.121   7.800  12.940  1.00 26.75           N
ATOM   2284  CA  VAL A 293      -7.593   9.139  13.260  1.00 26.08           C
ATOM   2285  C   VAL A 293      -9.082   9.127  12.906  1.00 26.05           C
ATOM   2286  O   VAL A 293      -9.506   9.564  11.824  1.00 23.87           O
ATOM   2287  CB  VAL A 293      -6.824  10.236  12.489  1.00 26.52           C
ATOM   2288  CG1 VAL A 293      -7.586  11.572  12.540  1.00 23.04           C
ATOM   2289  CG2 VAL A 293      -5.434  10.417  13.116  1.00 25.28           C
ATOM   2290  N   GLU A 294      -9.845   8.465  13.774  1.00 26.28           N
ATOM   2291  CA  GLU A 294     -11.252   8.203  13.506  1.00 27.28           C
ATOM   2292  C   GLU A 294     -12.157   8.786  14.564  1.00 25.18           C
ATOM   2293  O   GLU A 294     -12.655   8.059  15.407  1.00 25.83           O
ATOM   2294  CB  GLU A 294     -11.509   6.688  13.403  1.00 29.38           C
ATOM   2295  CG  GLU A 294     -11.930   6.202  12.013  1.00 27.11           C
```

ATOM	2296	CD	GLU	A	294	-12.826	7.200	11.307	1.00	28.00	C
ATOM	2297	OE1	GLU	A	294	-12.301	8.229	10.798	1.00	24.31	O
ATOM	2298	OE2	GLU	A	294	-14.056	6.961	11.289	1.00	26.88	O1-
ATOM	2299	N	ASN	A	295	-12.381	10.093	14.525	1.00	23.72	N
ATOM	2300	CA	ASN	A	295	-13.248	10.720	15.510	1.00	21.83	C
ATOM	2301	C	ASN	A	295	-14.523	9.893	15.424	1.00	18.95	C
ATOM	2302	O	ASN	A	295	-14.920	9.519	14.325	1.00	16.49	O
ATOM	2303	CB	ASN	A	295	-13.476	12.217	15.175	1.00	23.98	C
ATOM	2304	CG	ASN	A	295	-13.935	13.068	16.404	1.00	27.75	C
ATOM	2305	ND2	ASN	A	295	-13.404	12.768	17.605	1.00	26.54	N
ATOM	2306	OD1	ASN	A	295	-14.706	14.027	16.242	1.00	27.01	O
ATOM	2307	N	ASP	A	296	-14.757	9.198	16.536	1.00	19.63	N
ATOM	2308	CA	ASP	A	296	-15.900	8.306	16.708	1.00	18.42	C
ATOM	2309	C	ASP	A	296	-17.117	9.146	16.474	1.00	18.35	C
ATOM	2310	O	ASP	A	296	-17.158	10.280	16.940	1.00	19.40	O
ATOM	2311	CB	ASP	A	296	-16.006	7.790	18.155	1.00	17.52	C
ATOM	2312	CG	ASP	A	296	-14.676	7.708	18.867	1.00	16.66	C
ATOM	2313	OD1	ASP	A	296	-14.270	8.699	19.506	1.00	16.57	O
ATOM	2314	OD2	ASP	A	296	-14.096	6.609	18.887	1.00	17.73	O1-
ATOM	2315	N	GLU	A	297	-18.180	8.541	15.963	1.00	20.63	N
ATOM	2316	CA	GLU	A	297	-19.492	9.152	16.137	1.00	22.06	C
ATOM	2317	C	GLU	A	297	-20.176	8.612	17.385	1.00	20.95	C
ATOM	2318	O	GLU	A	297	-21.015	7.728	17.316	1.00	17.62	O
ATOM	2319	CB	GLU	A	297	-20.393	8.974	14.910	1.00	22.28	C
ATOM	2320	CG	GLU	A	297	-21.083	10.290	14.487	1.00	19.78	C
ATOM	2321	CD	GLU	A	297	-22.552	10.114	14.097	1.00	17.46	C
ATOM	2322	OE1	GLU	A	297	-23.107	9.029	14.331	1.00	18.89	O
ATOM	2323	OE2	GLU	A	297	-23.177	11.082	13.618	1.00	17.10	O1-
ATOM	2324	N	MET	A	298	-19.640	9.016	18.530	1.00	21.96	N
ATOM	2325	CA	MET	A	298	-20.458	9.312	19.694	1.00	23.40	C
ATOM	2326	C	MET	A	298	-21.712	10.057	19.184	1.00	25.29	C
ATOM	2327	O	MET	A	298	-21.650	10.760	18.154	1.00	23.28	O
ATOM	2328	CB	MET	A	298	-19.659	10.192	20.679	1.00	23.66	C
ATOM	2329	CG	MET	A	298	-19.890	11.713	20.580	1.00	23.57	C
ATOM	2330	SD	MET	A	298	-21.327	12.337	21.577	1.00	29.35	S
ATOM	2331	CE	MET	A	298	-20.736	14.067	22.065	1.00	21.21	C
ATOM	2332	N	PRO	A	299	-22.855	9.926	19.902	1.00	25.70	N
ATOM	2333	CA	PRO	A	299	-22.933	9.341	21.242	1.00	23.52	C
ATOM	2334	C	PRO	A	299	-23.444	7.894	21.195	1.00	22.57	C
ATOM	2335	O	PRO	A	299	-22.991	7.044	21.985	1.00	18.81	O
ATOM	2336	CB	PRO	A	299	-23.889	10.297	21.982	1.00	24.67	C
ATOM	2337	CG	PRO	A	299	-24.523	11.190	20.892	1.00	23.48	C
ATOM	2338	CD	PRO	A	299	-24.126	10.590	19.566	1.00	26.37	C
ATOM	2339	N	ALA	A	300	-24.449	7.663	20.336	1.00	23.45	N
ATOM	2340	CA	ALA	A	300	-24.730	6.330	19.769	1.00	24.55	C
ATOM	2341	C	ALA	A	300	-26.034	6.174	18.960	1.00	23.16	C
ATOM	2342	O	ALA	A	300	-26.739	7.152	18.691	1.00	21.49	O
ATOM	2343	CB	ALA	A	300	-24.655	5.248	20.867	1.00	26.49	C
ATOM	2344	N	ASP	A	301	-26.398	4.908	18.733	1.00	24.12	N
ATOM	2345	CA	ASP	A	301	-27.360	4.437	17.718	1.00	25.32	C
ATOM	2346	C	ASP	A	301	-28.711	5.075	17.818	1.00	26.66	C
ATOM	2347	O	ASP	A	301	-29.504	4.991	16.890	1.00	27.53	O
ATOM	2348	CB	ASP	A	301	-27.548	2.919	17.890	1.00	26.16	C
ATOM	2349	CG	ASP	A	301	-28.362	2.254	16.767	1.00	27.64	C
ATOM	2350	OD1	ASP	A	301	-28.582	2.850	15.681	1.00	27.63	O
ATOM	2351	OD2	ASP	A	301	-28.674	1.051	16.947	1.00	26.67	O1-
ATOM	2352	N	LEU	A	302	-28.958	5.727	18.946	1.00	30.35	N
ATOM	2353	CA	LEU	A	302	-30.086	5.351	19.792	1.00	31.56	C
ATOM	2354	C	LEU	A	302	-30.537	6.488	20.686	1.00	33.72	C
ATOM	2355	O	LEU	A	302	-29.798	7.474	20.867	1.00	34.95	O
ATOM	2356	CB	LEU	A	302	-29.647	4.182	20.653	1.00	28.92	C
ATOM	2357	CG	LEU	A	302	-28.155	4.371	20.967	1.00	31.25	C
ATOM	2358	CD1	LEU	A	302	-27.961	5.472	22.020	1.00	31.44	C
ATOM	2359	CD2	LEU	A	302	-27.489	3.041	21.378	1.00	32.08	C
ATOM	2360	N	PRO	A	303	-31.584	6.224	21.488	1.00	35.90	N
ATOM	2361	CA	PRO	A	303	-31.430	6.284	22.954	1.00	38.93	C
ATOM	2362	C	PRO	A	303	-30.874	4.971	23.513	1.00	38.86	C
ATOM	2363	O	PRO	A	303	-29.892	4.975	24.269	1.00	38.96	O
ATOM	2364	CB	PRO	A	303	-32.853	6.564	23.459	1.00	38.70	C
ATOM	2365	CG	PRO	A	303	-33.766	6.592	22.142	1.00	38.82	C
ATOM	2366	CD	PRO	A	303	-32.961	5.875	21.101	1.00	36.51	C

```
ATOM   2367  N   SER A 304     -31.360   3.855  22.964  1.00 37.24           N
ATOM   2368  CA  SER A 304     -31.097   2.522  23.528  1.00 35.92           C
ATOM   2369  C   SER A 304     -31.120   2.445  25.034  1.00 32.14           C
ATOM   2370  O   SER A 304     -30.681   1.441  25.597  1.00 30.33           O
ATOM   2371  CB  SER A 304     -29.774   1.948  23.042  1.00 36.77           C
ATOM   2372  OG  SER A 304     -30.029   0.971  22.042  1.00 42.09           O
ATOM   2373  N   LEU A 305     -31.942   3.312  25.607  1.00 30.13           N
ATOM   2374  CA  LEU A 305     -32.018   3.481  27.031  1.00 30.37           C
ATOM   2375  C   LEU A 305     -30.913   2.663  27.730  1.00 31.82           C
ATOM   2376  O   LEU A 305     -29.749   3.000  27.555  1.00 35.56           O
ATOM   2377  CB  LEU A 305     -33.405   3.084  27.532  1.00 29.63           C
ATOM   2378  CG  LEU A 305     -34.657   3.305  26.681  1.00 28.08           C
ATOM   2379  CD1 LEU A 305     -35.835   3.366  27.647  1.00 28.23           C
ATOM   2380  CD2 LEU A 305     -34.583   4.576  25.848  1.00 26.17           C
ATOM   2381  N   ALA A 306     -31.218   1.418  28.097  1.00 28.81           N
ATOM   2382  CA  ALA A 306     -30.480   0.759  29.160  1.00 25.85           C
ATOM   2383  C   ALA A 306     -30.048  -0.635  28.744  1.00 24.00           C
ATOM   2384  O   ALA A 306     -30.699  -1.241  27.912  1.00 23.85           O
ATOM   2385  CB  ALA A 306     -31.353   0.688  30.406  1.00 28.20           C
ATOM   2386  N   ALA A 307     -29.050  -1.198  29.422  1.00 22.64           N
ATOM   2387  CA  ALA A 307     -28.519  -2.505  29.043  1.00 24.62           C
ATOM   2388  C   ALA A 307     -28.896  -3.578  30.063  1.00 25.94           C
ATOM   2389  O   ALA A 307     -28.053  -4.112  30.807  1.00 22.41           O
ATOM   2390  CB  ALA A 307     -26.990  -2.432  28.860  1.00 26.11           C
ATOM   2391  N   ASP A 308     -30.187  -3.895  30.092  1.00 28.91           N
ATOM   2392  CA  ASP A 308     -30.732  -4.831  31.078  1.00 27.05           C
ATOM   2393  C   ASP A 308     -31.350  -6.031  30.350  1.00 26.11           C
ATOM   2394  O   ASP A 308     -32.535  -6.372  30.516  1.00 24.92           O
ATOM   2395  CB  ASP A 308     -31.721  -4.099  32.001  1.00 25.32           C
ATOM   2396  CG  ASP A 308     -31.006  -3.359  33.154  1.00 25.24           C
ATOM   2397  OD1 ASP A 308     -29.958  -3.840  33.629  1.00 19.08           O
ATOM   2398  OD2 ASP A 308     -31.452  -2.267  33.552  1.00 27.30           O1-
ATOM   2399  N   PHE A 309     -30.575  -6.493  29.370  1.00 21.83           N
ATOM   2400  CA  PHE A 309     -30.779  -7.762  28.711  1.00 18.30           C
ATOM   2401  C   PHE A 309     -29.716  -8.657  29.238  1.00 15.33           C
ATOM   2402  O   PHE A 309     -29.757  -9.841  29.018  1.00 18.02           O
ATOM   2403  CB  PHE A 309     -30.554  -7.614  27.217  1.00 21.96           C
ATOM   2404  CG  PHE A 309     -31.727  -8.017  26.378  1.00 22.43           C
ATOM   2405  CD1 PHE A 309     -33.007  -8.013  26.902  1.00 21.17           C
ATOM   2406  CD2 PHE A 309     -31.532  -8.450  25.084  1.00 21.60           C
ATOM   2407  CE1 PHE A 309     -34.060  -8.440  26.163  1.00 22.58           C
ATOM   2408  CE2 PHE A 309     -32.577  -8.876  24.337  1.00 24.09           C
ATOM   2409  CZ  PHE A 309     -33.851  -8.876  24.874  1.00 26.92           C
ATOM   2410  N   VAL A 310     -28.619  -8.040  29.631  1.00 17.52           N
ATOM   2411  CA  VAL A 310     -27.491  -8.754  30.234  1.00 21.16           C
ATOM   2412  C   VAL A 310     -27.676  -8.866  31.760  1.00 22.19           C
ATOM   2413  O   VAL A 310     -26.735  -9.228  32.476  1.00 19.99           O
ATOM   2414  CB  VAL A 310     -26.093  -8.031  29.910  1.00 22.76           C
ATOM   2415  CG1 VAL A 310     -26.078  -7.481  28.455  1.00 16.58           C
ATOM   2416  CG2 VAL A 310     -25.796  -6.903  30.929  1.00 19.12           C
ATOM   2417  N   GLU A 311     -28.904  -8.615  32.231  1.00 24.86           N
ATOM   2418  CA  GLU A 311     -29.132  -8.190  33.620  1.00 25.85           C
ATOM   2419  C   GLU A 311     -30.462  -8.620  34.250  1.00 26.80           C
ATOM   2420  O   GLU A 311     -30.617  -9.786  34.614  1.00 26.88           O
ATOM   2421  CB  GLU A 311     -28.997  -6.672  33.724  1.00 24.55           C
ATOM   2422  CG  GLU A 311     -28.150  -6.231  34.893  1.00 24.81           C
ATOM   2423  CD  GLU A 311     -28.390  -7.071  36.097  1.00 22.49           C
ATOM   2424  OE1 GLU A 311     -29.486  -6.952  36.659  1.00 27.72           O
ATOM   2425  OE2 GLU A 311     -27.551  -7.932  36.415  1.00 21.47           O1-
ATOM   2426  N   SER A 312     -31.340  -7.646  34.518  1.00 27.11           N
ATOM   2427  CA  SER A 312     -32.668  -7.899  35.086  1.00 28.09           C
ATOM   2428  C   SER A 312     -33.508  -8.811  34.212  1.00 29.00           C
ATOM   2429  O   SER A 312     -34.603  -8.413  33.813  1.00 32.24           O
ATOM   2430  CB  SER A 312     -33.463  -6.594  35.244  1.00 25.60           C
ATOM   2431  OG  SER A 312     -32.912  -5.751  36.227  1.00 29.41           O
ATOM   2432  N   LYS A 313     -33.071 -10.047  34.005  1.00 27.40           N
ATOM   2433  CA  LYS A 313     -33.782 -10.965  33.129  1.00 27.14           C
ATOM   2434  C   LYS A 313     -33.672 -12.368  33.711  1.00 30.75           C
ATOM   2435  O   LYS A 313     -33.007 -12.566  34.740  1.00 31.32           O
ATOM   2436  CB  LYS A 313     -33.153 -10.936  31.738  1.00 21.94           C
ATOM   2437  CG  LYS A 313     -33.859 -11.786  30.716  1.00 17.30           C
```

```
ATOM   2438  CD  LYS A 313     -34.578 -10.916  29.723  1.00 15.90           C
ATOM   2439  CE  LYS A 313     -34.503 -11.512  28.346  1.00 12.19           C
ATOM   2440  NZ  LYS A 313     -33.124 -11.478  27.875  1.00 14.13           N1+
ATOM   2441  N   ASP A 314     -34.367 -13.332  33.102  1.00 33.04           N
ATOM   2442  CA  ASP A 314     -33.947 -14.726  33.210  1.00 34.53           C
ATOM   2443  C   ASP A 314     -33.696 -15.303  31.820  1.00 34.78           C
ATOM   2444  O   ASP A 314     -34.638 -15.649  31.091  1.00 34.31           O
ATOM   2445  CB  ASP A 314     -34.962 -15.587  33.974  1.00 34.53           C
ATOM   2446  CG  ASP A 314     -34.538 -17.065  34.066  1.00 36.92           C
ATOM   2447  OD1 ASP A 314     -33.379 -17.438  33.692  1.00 33.31           O
ATOM   2448  OD2 ASP A 314     -35.415 -17.878  34.458  1.00 41.34           O1-
ATOM   2449  N   VAL A 315     -32.463 -15.055  31.384  1.00 33.55           N
ATOM   2450  CA  VAL A 315     -31.752 -15.855  30.402  1.00 30.37           C
ATOM   2451  C   VAL A 315     -30.272 -15.815  30.829  1.00 28.87           C
ATOM   2452  O   VAL A 315     -29.493 -14.952  30.414  1.00 25.57           O
ATOM   2453  CB  VAL A 315     -31.929 -15.287  28.979  1.00 32.83           C
ATOM   2454  CG1 VAL A 315     -33.417 -15.436  28.512  1.00 29.71           C
ATOM   2455  CG2 VAL A 315     -31.453 -13.820  28.934  1.00 33.25           C
ATOM   2456  N   CYS A 316     -30.032 -16.517  31.931  1.00 29.88           N
ATOM   2457  CA  CYS A 316     -28.683 -16.794  32.423  1.00 27.73           C
ATOM   2458  C   CYS A 316     -27.953 -17.584  31.361  1.00 26.33           C
ATOM   2459  O   CYS A 316     -27.153 -17.013  30.620  1.00 26.76           O
ATOM   2460  CB  CYS A 316     -28.734 -17.628  33.688  1.00 27.85           C
ATOM   2461  SG  CYS A 316     -27.077 -18.069  34.276  1.00 31.37           S
ATOM   2462  N   LYS A 317     -28.313 -18.863  31.217  1.00 22.54           N
ATOM   2463  CA  LYS A 317     -27.900 -19.631  30.046  1.00 21.29           C
ATOM   2464  C   LYS A 317     -28.905 -19.504  28.912  1.00 22.46           C
ATOM   2465  O   LYS A 317     -28.510 -19.220  27.790  1.00 22.63           O
ATOM   2466  CB  LYS A 317     -27.693 -21.120  30.369  1.00 18.85           C
ATOM   2467  CG  LYS A 317     -26.247 -21.541  30.640  1.00 18.02           C
ATOM   2468  CD  LYS A 317     -26.179 -22.731  31.604  1.00 20.06           C
ATOM   2469  CE  LYS A 317     -24.770 -22.943  32.219  1.00 22.33           C
ATOM   2470  NZ  LYS A 317     -24.375 -24.401  32.437  1.00 22.00           N1+
ATOM   2471  N   ASN A 318     -30.201 -19.646  29.199  1.00 22.97           N
ATOM   2472  CA  ASN A 318     -31.215 -19.864  28.143  1.00 22.32           C
ATOM   2473  C   ASN A 318     -30.963 -19.181  26.798  1.00 24.26           C
ATOM   2474  O   ASN A 318     -31.447 -19.657  25.759  1.00 26.24           O
ATOM   2475  CB  ASN A 318     -32.607 -19.439  28.601  1.00 19.64           C
ATOM   2476  CG  ASN A 318     -33.659 -19.693  27.542  1.00 17.20           C
ATOM   2477  ND2 ASN A 318     -33.852 -18.753  26.630  1.00 17.37           N
ATOM   2478  OD1 ASN A 318     -34.234 -20.753  27.507  1.00 19.89           O
ATOM   2479  N   TYR A 319     -30.390 -17.978  26.856  1.00 23.97           N
ATOM   2480  CA  TYR A 319     -30.222 -17.095  25.694  1.00 24.24           C
ATOM   2481  C   TYR A 319     -28.722 -16.854  25.621  1.00 23.49           C
ATOM   2482  O   TYR A 319     -28.250 -15.857  25.088  1.00 22.66           O
ATOM   2483  CB  TYR A 319     -31.022 -15.804  25.948  1.00 23.01           C
ATOM   2484  CG  TYR A 319     -30.749 -14.587  25.094  1.00 22.30           C
ATOM   2485  CD1 TYR A 319     -31.440 -14.393  23.908  1.00 24.18           C
ATOM   2486  CD2 TYR A 319     -30.040 -13.502  25.616  1.00 20.42           C
ATOM   2487  CE1 TYR A 319     -31.453 -13.148  23.278  1.00 23.68           C
ATOM   2488  CE2 TYR A 319     -30.044 -12.265  24.993  1.00 17.71           C
ATOM   2489  CZ  TYR A 319     -30.751 -12.097  23.826  1.00 20.42           C
ATOM   2490  OH  TYR A 319     -30.732 -10.898  23.159  1.00 22.03           O
ATOM   2491  N   ALA A 320     -27.993 -17.889  25.995  1.00 21.15           N
ATOM   2492  CA  ALA A 320     -26.572 -17.798  26.227  1.00 23.56           C
ATOM   2493  C   ALA A 320     -26.032 -19.198  26.089  1.00 25.78           C
ATOM   2494  O   ALA A 320     -25.428 -19.751  27.004  1.00 25.36           O
ATOM   2495  CB  ALA A 320     -26.290 -17.267  27.618  1.00 24.04           C
ATOM   2496  N   GLU A 321     -26.398 -19.828  24.985  1.00 28.84           N
ATOM   2497  CA  GLU A 321     -25.690 -21.008  24.536  1.00 27.99           C
ATOM   2498  C   GLU A 321     -24.907 -20.746  23.257  1.00 25.24           C
ATOM   2499  O   GLU A 321     -24.235 -21.634  22.715  1.00 27.19           O
ATOM   2500  CB  GLU A 321     -26.658 -22.167  24.388  1.00 30.20           C
ATOM   2501  CG  GLU A 321     -26.266 -23.326  25.298  1.00 37.63           C
ATOM   2502  CD  GLU A 321     -25.831 -22.921  26.736  1.00 38.24           C
ATOM   2503  OE1 GLU A 321     -24.725 -22.329  26.915  1.00 36.30           O
ATOM   2504  OE2 GLU A 321     -26.509 -23.388  27.699  1.00 40.13           O1-
ATOM   2505  N   ALA A 322     -24.889 -19.483  22.857  1.00 20.08           N
ATOM   2506  CA  ALA A 322     -23.864 -19.005  21.965  1.00 17.91           C
ATOM   2507  C   ALA A 322     -22.817 -18.388  22.879  1.00 16.32           C
ATOM   2508  O   ALA A 322     -23.051 -17.332  23.441  1.00 17.37           O
```

ATOM	2509	CB	ALA	A	322	-24.437	-17.962	21.032	1.00	16.29	C
ATOM	2510	N	LYS	A	323	-21.746	-19.116	23.166	1.00	13.15	N
ATOM	2511	CA	LYS	A	323	-20.707	-18.569	24.019	1.00	13.43	C
ATOM	2512	C	LYS	A	323	-20.251	-17.244	23.408	1.00	15.07	C
ATOM	2513	O	LYS	A	323	-20.751	-16.180	23.773	1.00	14.91	O
ATOM	2514	CB	LYS	A	323	-19.537	-19.560	24.124	1.00	14.15	C
ATOM	2515	CG	LYS	A	323	-18.201	-18.956	24.571	1.00	15.59	C
ATOM	2516	CD	LYS	A	323	-17.235	-20.027	25.079	1.00	14.64	C
ATOM	2517	CE	LYS	A	323	-15.850	-19.935	24.450	1.00	14.18	C
ATOM	2518	NZ	LYS	A	323	-15.145	-18.696	24.813	1.00	15.36	N1+
ATOM	2519	N	ASP	A	324	-19.608	-17.372	22.252	1.00	17.96	N
ATOM	2520	CA	ASP	A	324	-18.847	-16.299	21.623	1.00	15.80	C
ATOM	2521	C	ASP	A	324	-19.718	-15.035	21.411	1.00	17.56	C
ATOM	2522	O	ASP	A	324	-19.304	-13.924	21.781	1.00	17.35	O
ATOM	2523	CB	ASP	A	324	-18.233	-16.790	20.275	1.00	15.91	C
ATOM	2524	CG	ASP	A	324	-17.787	-18.293	20.293	1.00	16.85	C
ATOM	2525	OD1	ASP	A	324	-16.749	-18.623	20.903	1.00	15.85	O
ATOM	2526	OD2	ASP	A	324	-18.420	-19.140	19.617	1.00	17.67	O1-
ATOM	2527	N	VAL	A	325	-20.930	-15.180	20.870	1.00	15.39	N
ATOM	2528	CA	VAL	A	325	-21.660	-13.979	20.467	1.00	18.29	C
ATOM	2529	C	VAL	A	325	-22.388	-13.305	21.613	1.00	20.30	C
ATOM	2530	O	VAL	A	325	-22.751	-12.137	21.509	1.00	20.95	O
ATOM	2531	CB	VAL	A	325	-22.693	-14.215	19.333	1.00	18.19	C
ATOM	2532	CG1	VAL	A	325	-22.039	-14.874	18.125	1.00	17.25	C
ATOM	2533	CG2	VAL	A	325	-23.875	-15.003	19.849	1.00	19.92	C
ATOM	2534	N	PHE	A	326	-22.608	-14.039	22.701	1.00	23.24	N
ATOM	2535	CA	PHE	A	326	-23.259	-13.497	23.893	1.00	22.88	C
ATOM	2536	C	PHE	A	326	-22.336	-12.431	24.476	1.00	23.49	C
ATOM	2537	O	PHE	A	326	-22.675	-11.242	24.548	1.00	20.07	O
ATOM	2538	CB	PHE	A	326	-23.474	-14.624	24.911	1.00	23.89	C
ATOM	2539	CG	PHE	A	326	-24.307	-14.229	26.092	1.00	25.04	C
ATOM	2540	CD1	PHE	A	326	-25.603	-13.781	25.923	1.00	25.47	C
ATOM	2541	CD2	PHE	A	326	-23.776	-14.256	27.367	1.00	24.57	C
ATOM	2542	CE1	PHE	A	326	-26.356	-13.354	27.016	1.00	26.31	C
ATOM	2543	CE2	PHE	A	326	-24.522	-13.830	28.453	1.00	25.69	C
ATOM	2544	CZ	PHE	A	326	-25.814	-13.375	28.277	1.00	24.50	C
ATOM	2545	N	LEU	A	327	-21.109	-12.854	24.753	1.00	24.46	N
ATOM	2546	CA	LEU	A	327	-20.056	-11.948	25.200	1.00	25.44	C
ATOM	2547	C	LEU	A	327	-19.866	-10.805	24.193	1.00	23.62	C
ATOM	2548	O	LEU	A	327	-19.782	-9.623	24.568	1.00	18.87	O
ATOM	2549	CB	LEU	A	327	-18.744	-12.727	25.357	1.00	25.68	C
ATOM	2550	CG	LEU	A	327	-18.569	-13.566	26.624	1.00	22.99	C
ATOM	2551	CD1	LEU	A	327	-18.534	-12.630	27.851	1.00	21.08	C
ATOM	2552	CD2	LEU	A	327	-19.688	-14.578	26.719	1.00	21.68	C
ATOM	2553	N	GLY	A	328	-19.861	-11.175	22.911	1.00	22.86	N
ATOM	2554	CA	GLY	A	328	-19.715	-10.197	21.850	1.00	19.96	C
ATOM	2555	C	GLY	A	328	-20.775	-9.121	21.937	1.00	18.31	C
ATOM	2556	O	GLY	A	328	-20.496	-7.961	21.651	1.00	14.99	O
ATOM	2557	N	MET	A	329	-21.988	-9.503	22.332	1.00	18.03	N
ATOM	2558	CA	MET	A	329	-23.095	-8.563	22.406	1.00	16.11	C
ATOM	2559	C	MET	A	329	-23.171	-7.903	23.772	1.00	16.92	C
ATOM	2560	O	MET	A	329	-23.768	-6.838	23.930	1.00	12.56	O
ATOM	2561	CB	MET	A	329	-24.412	-9.253	22.090	1.00	15.19	C
ATOM	2562	CG	MET	A	329	-25.567	-8.288	22.048	1.00	16.53	C
ATOM	2563	SD	MET	A	329	-26.910	-8.847	21.026	1.00	19.89	S
ATOM	2564	CE	MET	A	329	-27.585	-7.279	20.531	1.00	17.00	C
ATOM	2565	N	PHE	A	330	-22.516	-8.495	24.761	1.00	18.68	N
ATOM	2566	CA	PHE	A	330	-22.249	-7.713	25.953	1.00	20.67	C
ATOM	2567	C	PHE	A	330	-21.302	-6.630	25.467	1.00	20.74	C
ATOM	2568	O	PHE	A	330	-21.793	-5.643	24.911	1.00	18.89	O
ATOM	2569	CB	PHE	A	330	-21.628	-8.544	27.091	1.00	19.97	C
ATOM	2570	CG	PHE	A	330	-21.607	-7.824	28.417	1.00	17.23	C
ATOM	2571	CD1	PHE	A	330	-22.552	-6.854	28.710	1.00	16.05	C
ATOM	2572	CD2	PHE	A	330	-20.594	-8.041	29.323	1.00	16.93	C
ATOM	2573	CE1	PHE	A	330	-22.478	-6.118	29.854	1.00	13.30	C
ATOM	2574	CE2	PHE	A	330	-20.523	-7.294	30.483	1.00	15.90	C
ATOM	2575	CZ	PHE	A	330	-21.464	-6.336	30.738	1.00	14.36	C
ATOM	2576	N	LEU	A	331	-20.019	-7.003	25.336	1.00	21.76	N
ATOM	2577	CA	LEU	A	331	-18.930	-6.124	24.885	1.00	22.65	C
ATOM	2578	C	LEU	A	331	-19.431	-4.988	24.012	1.00	26.84	C
ATOM	2579	O	LEU	A	331	-19.549	-3.859	24.503	1.00	30.55	O

ATOM	2580	CB	LEU	A	331	-17.900	-6.919	24.093	1.00	21.01	C
ATOM	2581	CG	LEU	A	331	-16.415	-6.566	24.178	1.00	21.98	C
ATOM	2582	CD1	LEU	A	331	-16.125	-5.467	25.205	1.00	18.67	C
ATOM	2583	CD2	LEU	A	331	-15.674	-7.848	24.537	1.00	19.61	C
ATOM	2584	N	TYR	A	332	-19.930	-5.338	22.816	1.00	27.17	N
ATOM	2585	CA	TYR	A	332	-20.406	-4.377	21.794	1.00	24.12	C
ATOM	2586	C	TYR	A	332	-21.396	-3.374	22.322	1.00	22.23	C
ATOM	2587	O	TYR	A	332	-21.229	-2.187	22.105	1.00	22.76	O
ATOM	2588	CB	TYR	A	332	-21.021	-5.134	20.599	1.00	22.53	C
ATOM	2589	CG	TYR	A	332	-22.199	-4.494	19.884	1.00	16.65	C
ATOM	2590	CD1	TYR	A	332	-22.123	-3.225	19.365	1.00	17.03	C
ATOM	2591	CD2	TYR	A	332	-23.319	-5.242	19.579	1.00	15.91	C
ATOM	2592	CE1	TYR	A	332	-23.132	-2.727	18.564	1.00	15.87	C
ATOM	2593	CE2	TYR	A	332	-24.309	-4.760	18.779	1.00	12.78	C
ATOM	2594	CZ	TYR	A	332	-24.219	-3.503	18.270	1.00	12.74	C
ATOM	2595	OH	TYR	A	332	-25.211	-3.032	17.443	1.00	11.75	O
ATOM	2596	N	GLU	A	333	-22.462	-3.858	22.947	1.00	21.11	N
ATOM	2597	CA	GLU	A	333	-23.480	-2.982	23.516	1.00	19.69	C
ATOM	2598	C	GLU	A	333	-22.973	-2.225	24.753	1.00	19.94	C
ATOM	2599	O	GLU	A	333	-23.239	-1.039	24.920	1.00	21.44	O
ATOM	2600	CB	GLU	A	333	-24.722	-3.791	23.854	1.00	17.19	C
ATOM	2601	CG	GLU	A	333	-25.676	-3.903	22.700	1.00	13.27	C
ATOM	2602	CD	GLU	A	333	-26.462	-2.640	22.499	1.00	16.80	C
ATOM	2603	OE1	GLU	A	333	-26.673	-1.903	23.474	1.00	17.89	O
ATOM	2604	OE2	GLU	A	333	-26.870	-2.358	21.358	1.00	21.21	O1-
ATOM	2605	N	TYR	A	334	-22.108	-2.846	25.535	1.00	17.50	N
ATOM	2606	CA	TYR	A	334	-21.473	-2.102	26.591	1.00	16.87	C
ATOM	2607	C	TYR	A	334	-20.552	-1.074	25.929	1.00	17.20	C
ATOM	2608	O	TYR	A	334	-20.643	0.126	26.220	1.00	15.06	O
ATOM	2609	CB	TYR	A	334	-20.694	-3.049	27.511	1.00	19.62	C
ATOM	2610	CG	TYR	A	334	-20.693	-2.600	28.969	1.00	21.56	C
ATOM	2611	CD1	TYR	A	334	-20.828	-1.258	29.304	1.00	23.27	C
ATOM	2612	CD2	TYR	A	334	-20.546	-3.503	29.994	1.00	19.88	C
ATOM	2613	CE1	TYR	A	334	-20.808	-0.840	30.609	1.00	21.45	C
ATOM	2614	CE2	TYR	A	334	-20.523	-3.084	31.297	1.00	21.56	C
ATOM	2615	CZ	TYR	A	334	-20.643	-1.750	31.594	1.00	21.82	C
ATOM	2616	OH	TYR	A	334	-20.475	-1.288	32.873	1.00	24.16	O
ATOM	2617	N	ALA	A	335	-19.934	-1.510	24.833	1.00	16.03	N
ATOM	2618	CA	ALA	A	335	-18.944	-0.733	24.108	1.00	15.10	C
ATOM	2619	C	ALA	A	335	-19.581	0.539	23.577	1.00	16.13	C
ATOM	2620	O	ALA	A	335	-19.116	1.638	23.861	1.00	17.41	O
ATOM	2621	CB	ALA	A	335	-18.392	-1.555	22.966	1.00	13.10	C
ATOM	2622	N	ARG	A	336	-20.753	0.374	22.974	1.00	16.93	N
ATOM	2623	CA	ARG	A	336	-21.547	1.458	22.418	1.00	16.89	C
ATOM	2624	C	ARG	A	336	-21.658	2.712	23.290	1.00	18.08	C
ATOM	2625	O	ARG	A	336	-21.482	3.820	22.803	1.00	16.00	O
ATOM	2626	CB	ARG	A	336	-22.946	0.940	22.127	1.00	17.05	C
ATOM	2627	CG	ARG	A	336	-23.108	0.324	20.776	1.00	16.01	C
ATOM	2628	CD	ARG	A	336	-24.129	1.117	19.996	1.00	18.77	C
ATOM	2629	NE	ARG	A	336	-25.334	0.350	19.735	1.00	23.35	N
ATOM	2630	CZ	ARG	A	336	-25.554	-0.337	18.613	1.00	26.57	C
ATOM	2631	NH1	ARG	A	336	-24.595	-0.458	17.700	1.00	24.51	N1+
ATOM	2632	NH2	ARG	A	336	-26.743	-0.891	18.390	1.00	27.93	N
ATOM	2633	N	ARG	A	337	-22.052	2.543	24.548	1.00	21.47	N
ATOM	2634	CA	ARG	A	337	-22.449	3.689	25.383	1.00	24.30	C
ATOM	2635	C	ARG	A	337	-21.309	4.239	26.248	1.00	23.58	C
ATOM	2636	O	ARG	A	337	-21.539	4.923	27.280	1.00	21.32	O
ATOM	2637	CB	ARG	A	337	-23.657	3.342	26.285	1.00	27.68	C
ATOM	2638	CG	ARG	A	337	-24.089	1.856	26.376	1.00	29.06	C
ATOM	2639	CD	ARG	A	337	-25.460	1.731	27.076	1.00	29.77	C
ATOM	2640	NE	ARG	A	337	-26.418	0.886	26.353	1.00	28.59	N
ATOM	2641	CZ	ARG	A	337	-26.845	1.102	25.108	1.00	28.23	C
ATOM	2642	NH1	ARG	A	337	-26.324	2.083	24.382	1.00	27.06	N1+
ATOM	2643	NH2	ARG	A	337	-27.795	0.324	24.582	1.00	24.49	N
ATOM	2644	N	HIS	A	338	-20.082	3.889	25.864	1.00	21.05	N
ATOM	2645	CA	HIS	A	338	-18.922	4.164	26.703	1.00	18.37	C
ATOM	2646	C	HIS	A	338	-17.702	4.574	25.901	1.00	13.99	C
ATOM	2647	O	HIS	A	338	-16.611	4.073	26.118	1.00	11.40	O
ATOM	2648	CB	HIS	A	338	-18.610	2.954	27.576	1.00	19.06	C
ATOM	2649	CG	HIS	A	338	-19.551	2.791	28.728	1.00	21.75	C
ATOM	2650	CD2	HIS	A	338	-19.937	1.685	29.406	1.00	21.31	C

ATOM	2651	ND1	HIS	A	338	-20.238	3.847	29.288	1.00	22.04	N
ATOM	2652	CE1	HIS	A	338	-21.007	3.394	30.267	1.00	25.17	C
ATOM	2653	NE2	HIS	A	338	-20.844	2.087	30.358	1.00	21.03	N
ATOM	2654	N	PRO	A	339	-17.826	5.673	25.169	1.00	10.97	N
ATOM	2655	CA	PRO	A	339	-16.623	6.285	24.652	1.00	12.13	C
ATOM	2656	C	PRO	A	339	-15.808	6.864	25.780	1.00	14.82	C
ATOM	2657	O	PRO	A	339	-14.743	7.430	25.543	1.00	16.89	O
ATOM	2658	CB	PRO	A	339	-17.148	7.379	23.719	1.00	12.95	C
ATOM	2659	CG	PRO	A	339	-18.636	7.237	23.705	1.00	11.47	C
ATOM	2660	CD	PRO	A	339	-18.985	6.546	24.970	1.00	12.68	C
ATOM	2661	N	ASP	A	340	-16.335	6.799	26.998	1.00	16.39	N
ATOM	2662	CA	ASP	A	340	-15.676	7.456	28.120	1.00	17.93	C
ATOM	2663	C	ASP	A	340	-14.888	6.494	29.012	1.00	16.22	C
ATOM	2664	O	ASP	A	340	-14.163	6.926	29.905	1.00	13.33	O
ATOM	2665	CB	ASP	A	340	-16.689	8.324	28.916	1.00	23.67	C
ATOM	2666	CG	ASP	A	340	-17.750	7.512	29.671	1.00	28.01	C
ATOM	2667	OD1	ASP	A	340	-18.805	7.174	29.077	1.00	27.93	O
ATOM	2668	OD2	ASP	A	340	-17.630	7.403	30.917	1.00	31.19	O1-
ATOM	2669	N	TYR	A	341	-14.848	5.230	28.586	1.00	16.78	N
ATOM	2670	CA	TYR	A	341	-14.063	4.179	29.225	1.00	16.62	C
ATOM	2671	C	TYR	A	341	-13.182	3.548	28.151	1.00	18.04	C
ATOM	2672	O	TYR	A	341	-13.662	3.157	27.095	1.00	19.19	O
ATOM	2673	CB	TYR	A	341	-14.956	3.059	29.782	1.00	17.89	C
ATOM	2674	CG	TYR	A	341	-15.863	3.378	30.973	1.00	17.57	C
ATOM	2675	CD1	TYR	A	341	-15.606	4.437	31.821	1.00	16.92	C
ATOM	2676	CD2	TYR	A	341	-16.915	2.536	31.290	1.00	15.97	C
ATOM	2677	CE1	TYR	A	341	-16.361	4.638	32.959	1.00	20.41	C
ATOM	2678	CE2	TYR	A	341	-17.666	2.723	32.406	1.00	18.32	C
ATOM	2679	CZ	TYR	A	341	-17.393	3.772	33.260	1.00	20.96	C
ATOM	2680	OH	TYR	A	341	-18.120	3.905	34.452	1.00	18.90	O
ATOM	2681	N	SER	A	342	-11.972	3.194	28.546	1.00	19.93	N
ATOM	2682	CA	SER	A	342	-10.979	2.592	27.668	1.00	17.81	C
ATOM	2683	C	SER	A	342	-11.334	1.153	27.386	1.00	17.13	C
ATOM	2684	O	SER	A	342	-11.554	0.401	28.331	1.00	17.80	O
ATOM	2685	CB	SER	A	342	-9.643	2.595	28.389	1.00	19.87	C
ATOM	2686	OG	SER	A	342	-9.820	2.057	29.693	1.00	17.44	O
ATOM	2687	N	VAL	A	343	-11.037	0.712	26.168	1.00	16.67	N
ATOM	2688	CA	VAL	A	343	-11.195	-0.679	25.788	1.00	15.06	C
ATOM	2689	C	VAL	A	343	-10.570	-1.594	26.815	1.00	15.29	C
ATOM	2690	O	VAL	A	343	-11.284	-2.267	27.538	1.00	17.32	O
ATOM	2691	CB	VAL	A	343	-10.601	-0.964	24.386	1.00	17.69	C
ATOM	2692	CG1	VAL	A	343	-9.194	-0.425	24.270	1.00	17.11	C
ATOM	2693	CG2	VAL	A	343	-10.638	-2.460	24.091	1.00	19.44	C
ATOM	2694	N	VAL	A	344	-9.273	-1.446	27.044	1.00	14.96	N
ATOM	2695	CA	VAL	A	344	-8.571	-2.281	28.013	1.00	12.81	C
ATOM	2696	C	VAL	A	344	-9.315	-2.430	29.334	1.00	14.93	C
ATOM	2697	O	VAL	A	344	-9.358	-3.522	29.872	1.00	16.62	O
ATOM	2698	CB	VAL	A	344	-7.169	-1.751	28.262	1.00	9.56	C
ATOM	2699	CG1	VAL	A	344	-6.537	-2.427	29.439	1.00	11.59	C
ATOM	2700	CG2	VAL	A	344	-6.344	-1.992	27.048	1.00	15.07	C
ATOM	2701	N	LEU	A	345	-10.048	-1.400	29.763	1.00	16.26	N
ATOM	2702	CA	LEU	A	345	-10.925	-1.526	30.942	1.00	17.05	C
ATOM	2703	C	LEU	A	345	-12.105	-2.415	30.592	1.00	15.69	C
ATOM	2704	O	LEU	A	345	-12.294	-3.481	31.172	1.00	14.48	O
ATOM	2705	CB	LEU	A	345	-11.459	-0.159	31.392	1.00	18.45	C
ATOM	2706	CG	LEU	A	345	-11.962	0.060	32.830	1.00	18.68	C
ATOM	2707	CD1	LEU	A	345	-13.262	-0.629	33.058	1.00	17.13	C
ATOM	2708	CD2	LEU	A	345	-10.926	-0.419	33.841	1.00	21.35	C
ATOM	2709	N	LEU	A	346	-12.855	-1.992	29.588	1.00	14.21	N
ATOM	2710	CA	LEU	A	346	-14.109	-2.633	29.226	1.00	14.41	C
ATOM	2711	C	LEU	A	346	-13.900	-4.106	28.967	1.00	15.55	C
ATOM	2712	O	LEU	A	346	-14.832	-4.889	29.076	1.00	16.56	O
ATOM	2713	CB	LEU	A	346	-14.688	-1.981	27.970	1.00	14.22	C
ATOM	2714	CG	LEU	A	346	-16.004	-1.213	27.991	1.00	11.29	C
ATOM	2715	CD1	LEU	A	346	-16.066	-0.240	29.135	1.00	13.74	C
ATOM	2716	CD2	LEU	A	346	-16.105	-0.487	26.692	1.00	11.30	C
ATOM	2717	N	LEU	A	347	-12.680	-4.472	28.591	1.00	17.67	N
ATOM	2718	CA	LEU	A	347	-12.325	-5.868	28.316	1.00	18.46	C
ATOM	2719	C	LEU	A	347	-12.252	-6.655	29.618	1.00	18.38	C
ATOM	2720	O	LEU	A	347	-12.542	-7.844	29.626	1.00	18.38	O
ATOM	2721	CB	LEU	A	347	-10.977	-5.941	27.598	1.00	18.93	C

ATOM	2722	CG	LEU	A	347	-10.687	-7.030	26.566	1.00	18.83	C
ATOM	2723	CD1	LEU	A	347	-11.567	-8.246	26.778	1.00	17.43	C
ATOM	2724	CD2	LEU	A	347	-10.883	-6.449	25.188	1.00	17.31	C
ATOM	2725	N	ARG	A	348	-11.908	-5.966	30.710	1.00	18.37	N
ATOM	2726	CA	ARG	A	348	-12.082	-6.454	32.088	1.00	19.23	C
ATOM	2727	C	ARG	A	348	-13.543	-6.617	32.563	1.00	19.03	C
ATOM	2728	O	ARG	A	348	-13.821	-7.463	33.423	1.00	21.17	O
ATOM	2729	CB	ARG	A	348	-11.365	-5.526	33.060	1.00	17.92	C
ATOM	2730	CG	ARG	A	348	-9.954	-5.943	33.395	1.00	21.29	C
ATOM	2731	CD	ARG	A	348	-9.199	-4.790	34.054	1.00	22.78	C
ATOM	2732	NE	ARG	A	348	-7.997	-5.281	34.717	1.00	25.21	N
ATOM	2733	CZ	ARG	A	348	-7.661	-5.008	35.977	1.00	26.74	C
ATOM	2734	NH1	ARG	A	348	-8.245	-4.007	36.636	1.00	24.24	N1+
ATOM	2735	NH2	ARG	A	348	-6.631	-5.644	36.530	1.00	25.87	N
ATOM	2736	N	LEU	A	349	-14.437	-5.728	32.119	1.00	15.77	N
ATOM	2737	CA	LEU	A	349	-15.868	-5.894	32.346	1.00	11.96	C
ATOM	2738	C	LEU	A	349	-16.417	-7.004	31.453	1.00	14.75	C
ATOM	2739	O	LEU	A	349	-17.446	-7.619	31.748	1.00	16.02	O
ATOM	2740	CB	LEU	A	349	-16.599	-4.603	32.054	1.00	6.86	C
ATOM	2741	CG	LEU	A	349	-16.030	-3.328	32.671	1.00	12.17	C
ATOM	2742	CD1	LEU	A	349	-17.117	-2.246	32.667	1.00	14.15	C
ATOM	2743	CD2	LEU	A	349	-15.553	-3.568	34.090	1.00	10.52	C
ATOM	2744	N	ALA	A	350	-15.667	-7.344	30.416	1.00	15.21	N
ATOM	2745	CA	ALA	A	350	-16.061	-8.424	29.539	1.00	16.39	C
ATOM	2746	C	ALA	A	350	-15.410	-9.730	29.963	1.00	17.56	C
ATOM	2747	O	ALA	A	350	-15.616	-10.767	29.332	1.00	17.63	O
ATOM	2748	CB	ALA	A	350	-15.695	-8.084	28.116	1.00	17.35	C
ATOM	2749	N	LYS	A	351	-14.654	-9.688	31.057	1.00	19.16	N
ATOM	2750	CA	LYS	A	351	-14.267	-10.913	31.747	1.00	20.56	C
ATOM	2751	C	LYS	A	351	-14.763	-11.048	33.166	1.00	20.35	C
ATOM	2752	O	LYS	A	351	-14.523	-12.083	33.770	1.00	20.19	O
ATOM	2753	CB	LYS	A	351	-12.756	-11.122	31.726	1.00	23.48	C
ATOM	2754	CG	LYS	A	351	-12.300	-12.152	30.682	1.00	29.67	C
ATOM	2755	CD	LYS	A	351	-12.868	-11.815	29.274	1.00	33.17	C
ATOM	2756	CE	LYS	A	351	-11.936	-12.235	28.116	1.00	33.44	C
ATOM	2757	NZ	LYS	A	351	-12.007	-11.236	26.983	1.00	31.75	N1+
ATOM	2758	N	THR	A	352	-15.548	-10.079	33.651	1.00	22.45	N
ATOM	2759	CA	THR	A	352	-16.326	-10.254	34.892	1.00	25.02	C
ATOM	2760	C	THR	A	352	-17.850	-10.434	34.671	1.00	27.15	C
ATOM	2761	O	THR	A	352	-18.596	-10.768	35.590	1.00	28.15	O
ATOM	2762	CB	THR	A	352	-16.105	-9.093	35.854	1.00	25.24	C
ATOM	2763	CG2	THR	A	352	-16.302	-9.557	37.305	1.00	25.36	C
ATOM	2764	OG1	THR	A	352	-14.772	-8.583	35.690	1.00	27.53	O
ATOM	2765	N	TYR	A	353	-18.314	-10.227	33.443	1.00	28.56	N
ATOM	2766	CA	TYR	A	353	-19.436	-11.009	32.951	1.00	27.35	C
ATOM	2767	C	TYR	A	353	-18.988	-12.487	32.781	1.00	28.05	C
ATOM	2768	O	TYR	A	353	-19.141	-13.277	33.718	1.00	29.94	O
ATOM	2769	CB	TYR	A	353	-19.942	-10.427	31.629	1.00	25.44	C
ATOM	2770	CG	TYR	A	353	-21.447	-10.511	31.448	1.00	24.45	C
ATOM	2771	CD1	TYR	A	353	-22.327	-10.076	32.455	1.00	21.15	C
ATOM	2772	CD2	TYR	A	353	-21.991	-11.122	30.316	1.00	22.41	C
ATOM	2773	CE1	TYR	A	353	-23.705	-10.270	32.338	1.00	18.94	C
ATOM	2774	CE2	TYR	A	353	-23.364	-11.318	30.195	1.00	19.82	C
ATOM	2775	CZ	TYR	A	353	-24.208	-10.906	31.206	1.00	19.86	C
ATOM	2776	OH	TYR	A	353	-25.525	-11.262	31.124	1.00	18.86	O
ATOM	2777	N	GLU	A	354	-18.244	-12.794	31.714	1.00	25.58	N
ATOM	2778	CA	GLU	A	354	-17.941	-14.192	31.332	1.00	24.34	C
ATOM	2779	C	GLU	A	354	-17.618	-15.178	32.445	1.00	23.15	C
ATOM	2780	O	GLU	A	354	-17.902	-16.361	32.315	1.00	21.79	O
ATOM	2781	CB	GLU	A	354	-16.774	-14.261	30.346	1.00	25.14	C
ATOM	2782	CG	GLU	A	354	-16.485	-15.694	29.867	1.00	24.30	C
ATOM	2783	CD	GLU	A	354	-15.011	-16.006	29.672	1.00	23.93	C
ATOM	2784	OE1	GLU	A	354	-14.145	-15.338	30.288	1.00	22.57	O
ATOM	2785	OE2	GLU	A	354	-14.727	-16.952	28.905	1.00	22.72	O1-
ATOM	2786	N	THR	A	355	-16.746	-14.776	33.353	1.00	22.89	N
ATOM	2787	CA	THR	A	355	-16.424	-15.652	34.455	1.00	21.39	C
ATOM	2788	C	THR	A	355	-17.689	-15.821	35.259	1.00	23.76	C
ATOM	2789	O	THR	A	355	-18.292	-16.908	35.252	1.00	24.10	O
ATOM	2790	CB	THR	A	355	-15.414	-15.041	35.375	1.00	22.64	C
ATOM	2791	CG2	THR	A	355	-14.930	-16.101	36.358	1.00	23.56	C
ATOM	2792	OG1	THR	A	355	-14.333	-14.484	34.609	1.00	23.91	O

ATOM	2793	N	THR	A	356	-18.140	-14.704	35.852	1.00	23.40	N
ATOM	2794	CA	THR	A	356	-19.244	-14.695	36.812	1.00	23.16	C
ATOM	2795	C	THR	A	356	-20.470	-15.421	36.293	1.00	22.64	C
ATOM	2796	O	THR	A	356	-20.910	-16.389	36.901	1.00	24.94	O
ATOM	2797	CB	THR	A	356	-19.636	-13.257	37.216	1.00	24.04	C
ATOM	2798	CG2	THR	A	356	-21.004	-13.225	37.905	1.00	21.12	C
ATOM	2799	OG1	THR	A	356	-18.647	-12.740	38.121	1.00	28.79	O
ATOM	2800	N	LEU	A	357	-20.936	-15.060	35.105	1.00	19.87	N
ATOM	2801	CA	LEU	A	357	-22.092	-15.724	34.542	1.00	16.07	C
ATOM	2802	C	LEU	A	357	-21.893	-17.215	34.408	1.00	16.68	C
ATOM	2803	O	LEU	A	357	-22.844	-17.937	34.102	1.00	16.65	O
ATOM	2804	CB	LEU	A	357	-22.428	-15.140	33.189	1.00	13.80	C
ATOM	2805	CG	LEU	A	357	-23.662	-14.274	33.305	1.00	14.12	C
ATOM	2806	CD1	LEU	A	357	-23.278	-12.964	33.979	1.00	11.35	C
ATOM	2807	CD2	LEU	A	357	-24.275	-14.076	31.936	1.00	9.43	C
ATOM	2808	N	GLU	A	358	-20.639	-17.653	34.510	1.00	18.54	N
ATOM	2809	CA	GLU	A	358	-20.306	-19.077	34.509	1.00	22.18	C
ATOM	2810	C	GLU	A	358	-20.175	-19.565	35.932	1.00	23.29	C
ATOM	2811	O	GLU	A	358	-20.495	-20.722	36.210	1.00	25.03	O
ATOM	2812	CB	GLU	A	358	-19.004	-19.353	33.731	1.00	25.37	C
ATOM	2813	CG	GLU	A	358	-19.208	-20.016	32.329	1.00	29.81	C
ATOM	2814	CD	GLU	A	358	-19.717	-19.054	31.220	1.00	30.46	C
ATOM	2815	OE1	GLU	A	358	-18.859	-18.380	30.586	1.00	30.40	O
ATOM	2816	OE2	GLU	A	358	-20.946	-19.046	30.925	1.00	25.21	O1-
ATOM	2817	N	LYS	A	359	-19.818	-18.642	36.833	1.00	22.28	N
ATOM	2818	CA	LYS	A	359	-19.902	-18.823	38.294	1.00	21.07	C
ATOM	2819	C	LYS	A	359	-21.329	-19.097	38.762	1.00	21.42	C
ATOM	2820	O	LYS	A	359	-21.543	-19.518	39.903	1.00	21.98	O
ATOM	2821	CB	LYS	A	359	-19.409	-17.562	39.021	1.00	20.11	C
ATOM	2822	CG	LYS	A	359	-18.134	-17.700	39.848	1.00	22.37	C
ATOM	2823	CD	LYS	A	359	-17.703	-19.159	40.055	1.00	24.00	C
ATOM	2824	CE	LYS	A	359	-16.830	-19.349	41.307	1.00	24.54	C
ATOM	2825	NZ	LYS	A	359	-17.624	-19.380	42.606	1.00	21.49	N1+
ATOM	2826	N	CYS	A	360	-22.304	-18.705	37.946	1.00	22.87	N
ATOM	2827	CA	CYS	A	360	-23.709	-18.847	38.318	1.00	24.14	C
ATOM	2828	C	CYS	A	360	-24.710	-18.775	37.158	1.00	23.71	C
ATOM	2829	O	CYS	A	360	-25.613	-17.929	37.133	1.00	24.52	O
ATOM	2830	CB	CYS	A	360	-24.057	-17.808	39.374	1.00	24.78	C
ATOM	2831	SG	CYS	A	360	-22.748	-16.563	39.608	1.00	26.85	S
ATOM	2832	N	CYS	A	361	-24.405	-19.527	36.113	1.00	22.64	N
ATOM	2833	CA	CYS	A	361	-25.419	-20.138	35.292	1.00	22.37	C
ATOM	2834	C	CYS	A	361	-25.485	-21.605	35.702	1.00	23.29	C
ATOM	2835	O	CYS	A	361	-26.339	-22.359	35.231	1.00	23.57	O
ATOM	2836	CB	CYS	A	361	-25.025	-20.055	33.828	1.00	23.14	C
ATOM	2837	SG	CYS	A	361	-26.368	-19.332	32.880	1.00	26.83	S
ATOM	2838	N	ALA	A	362	-24.508	-22.030	36.497	1.00	23.59	N
ATOM	2839	CA	ALA	A	362	-24.434	-23.405	36.944	1.00	25.12	C
ATOM	2840	C	ALA	A	362	-25.368	-23.670	38.107	1.00	25.80	C
ATOM	2841	O	ALA	A	362	-26.545	-23.951	37.901	1.00	26.67	O
ATOM	2842	CB	ALA	A	362	-22.999	-23.748	37.342	1.00	27.30	C
ATOM	2843	N	ALA	A	363	-24.858	-23.502	39.326	1.00	23.91	N
ATOM	2844	CA	ALA	A	363	-25.445	-24.155	40.488	1.00	26.21	C
ATOM	2845	C	ALA	A	363	-26.359	-23.304	41.386	1.00	26.42	C
ATOM	2846	O	ALA	A	363	-26.234	-22.069	41.407	1.00	24.96	O
ATOM	2847	CB	ALA	A	363	-24.331	-24.804	41.343	1.00	25.36	C
ATOM	2848	N	ALA	A	364	-27.415	-23.948	41.912	1.00	28.14	N
ATOM	2849	CA	ALA	A	364	-28.010	-23.673	43.236	1.00	31.01	C
ATOM	2850	C	ALA	A	364	-28.676	-22.323	43.366	1.00	32.95	C
ATOM	2851	O	ALA	A	364	-28.424	-21.582	44.343	1.00	33.01	O
ATOM	2852	CB	ALA	A	364	-26.941	-23.792	44.302	1.00	32.09	C
ATOM	2853	N	ASP	A	365	-29.630	-22.080	42.470	1.00	33.49	N
ATOM	2854	CA	ASP	A	365	-30.065	-20.719	42.092	1.00	32.31	C
ATOM	2855	C	ASP	A	365	-30.308	-19.789	43.290	1.00	34.06	C
ATOM	2856	O	ASP	A	365	-30.266	-20.216	44.442	1.00	35.19	O
ATOM	2857	CB	ASP	A	365	-31.305	-20.839	41.165	1.00	30.17	C
ATOM	2858	CG	ASP	A	365	-32.215	-21.973	41.556	1.00	27.56	C
ATOM	2859	OD1	ASP	A	365	-32.811	-21.879	42.644	1.00	25.00	O
ATOM	2860	OD2	ASP	A	365	-32.379	-22.899	40.741	1.00	24.35	O1-
ATOM	2861	N	PRO	A	366	-30.401	-18.469	43.078	1.00	34.76	N
ATOM	2862	CA	PRO	A	366	-31.123	-17.957	41.922	1.00	34.85	C
ATOM	2863	C	PRO	A	366	-30.281	-16.923	41.199	1.00	35.81	C

```
ATOM   2864  O   PRO A 366     -29.071 -17.095  41.054  1.00 36.75           O
ATOM   2865  CB  PRO A 366     -32.402 -17.379  42.539  1.00 33.58           C
ATOM   2866  CG  PRO A 366     -31.998 -16.969  43.986  1.00 34.17           C
ATOM   2867  CD  PRO A 366     -30.585 -17.580  44.243  1.00 35.22           C
ATOM   2868  N   HIS A 367     -30.879 -15.760  40.952  1.00 35.99           N
ATOM   2869  CA  HIS A 367     -30.318 -14.767  40.058  1.00 32.68           C
ATOM   2870  C   HIS A 367     -29.370 -13.770  40.677  1.00 32.19           C
ATOM   2871  O   HIS A 367     -28.438 -13.300  40.019  1.00 32.60           O
ATOM   2872  CB  HIS A 367     -31.447 -14.017  39.383  1.00 30.02           C
ATOM   2873  CG  HIS A 367     -31.860 -14.638  38.108  1.00 25.99           C
ATOM   2874  CD2 HIS A 367     -32.718 -14.223  37.152  1.00 26.12           C
ATOM   2875  ND1 HIS A 367     -31.248 -15.773  37.620  1.00 25.25           N
ATOM   2876  CE1 HIS A 367     -31.706 -16.026  36.408  1.00 27.08           C
ATOM   2877  NE2 HIS A 367     -32.598 -15.099  36.097  1.00 29.00           N
ATOM   2878  N   GLU A 368     -29.538 -13.526  41.962  1.00 30.39           N
ATOM   2879  CA  GLU A 368     -28.843 -12.426  42.577  1.00 29.24           C
ATOM   2880  C   GLU A 368     -27.331 -12.453  42.287  1.00 28.13           C
ATOM   2881  O   GLU A 368     -26.729 -11.384  42.163  1.00 26.88           O
ATOM   2882  CB  GLU A 368     -29.136 -12.413  44.090  1.00 29.55           C
ATOM   2883  CG  GLU A 368     -30.634 -12.566  44.422  1.00 25.73           C
ATOM   2884  CD  GLU A 368     -31.265 -11.300  44.974  1.00 24.58           C
ATOM   2885  OE1 GLU A 368     -30.521 -10.338  45.266  1.00 25.16           O
ATOM   2886  OE2 GLU A 368     -32.498 -11.296  45.176  1.00 21.06           O1-
ATOM   2887  N   CYS A 369     -26.758 -13.638  42.021  1.00 26.26           N
ATOM   2888  CA  CYS A 369     -25.313 -13.757  41.759  1.00 24.90           C
ATOM   2889  C   CYS A 369     -24.874 -12.701  40.778  1.00 25.23           C
ATOM   2890  O   CYS A 369     -23.911 -12.003  41.032  1.00 25.76           O
ATOM   2891  CB  CYS A 369     -24.941 -15.093  41.136  1.00 25.85           C
ATOM   2892  SG  CYS A 369     -23.840 -14.850  39.683  1.00 28.33           S
ATOM   2893  N   TYR A 370     -25.488 -12.721  39.593  1.00 24.71           N
ATOM   2894  CA  TYR A 370     -25.245 -11.722  38.563  1.00 23.51           C
ATOM   2895  C   TYR A 370     -26.029 -10.420  38.757  1.00 24.62           C
ATOM   2896  O   TYR A 370     -25.953  -9.523  37.906  1.00 23.98           O
ATOM   2897  CB  TYR A 370     -25.537 -12.308  37.178  1.00 24.52           C
ATOM   2898  CG  TYR A 370     -26.989 -12.560  36.798  1.00 22.90           C
ATOM   2899  CD1 TYR A 370     -27.771 -11.529  36.277  1.00 24.94           C
ATOM   2900  CD2 TYR A 370     -27.445 -13.861  36.605  1.00 22.80           C
ATOM   2901  CE1 TYR A 370     -28.942 -11.789  35.548  1.00 23.26           C
ATOM   2902  CE2 TYR A 370     -28.605 -14.126  35.880  1.00 21.68           C
ATOM   2903  CZ  TYR A 370     -29.347 -13.085  35.357  1.00 22.31           C
ATOM   2904  OH  TYR A 370     -30.530 -13.345  34.705  1.00 19.63           O
ATOM   2905  N   ALA A 371     -26.806 -10.341  39.843  1.00 22.53           N
ATOM   2906  CA  ALA A 371     -27.517  -9.121  40.212  1.00 20.95           C
ATOM   2907  C   ALA A 371     -26.534  -8.002  40.483  1.00 21.22           C
ATOM   2908  O   ALA A 371     -25.767  -8.032  41.452  1.00 18.20           O
ATOM   2909  CB  ALA A 371     -28.392  -9.347  41.437  1.00 20.29           C
ATOM   2910  N   LYS A 372     -26.618  -6.979  39.644  1.00 23.07           N
ATOM   2911  CA  LYS A 372     -25.638  -5.897  39.648  1.00 26.70           C
ATOM   2912  C   LYS A 372     -24.221  -6.384  39.978  1.00 28.46           C
ATOM   2913  O   LYS A 372     -23.624  -5.993  41.000  1.00 26.84           O
ATOM   2914  CB  LYS A 372     -26.041  -4.787  40.619  1.00 25.91           C
ATOM   2915  CG  LYS A 372     -27.377  -4.140  40.291  1.00 25.48           C
ATOM   2916  CD  LYS A 372     -27.886  -3.362  41.489  1.00 21.87           C
ATOM   2917  CE  LYS A 372     -26.866  -2.361  41.922  1.00 18.47           C
ATOM   2918  NZ  LYS A 372     -26.407  -1.618  40.728  1.00 20.65           N1+
ATOM   2919  N   VAL A 373     -23.710  -7.262  39.109  1.00 27.77           N
ATOM   2920  CA  VAL A 373     -22.311  -7.224  38.739  1.00 24.92           C
ATOM   2921  C   VAL A 373     -21.958  -5.773  38.482  1.00 24.34           C
ATOM   2922  O   VAL A 373     -20.982  -5.276  39.007  1.00 23.37           O
ATOM   2923  CB  VAL A 373     -22.036  -8.008  37.459  1.00 25.87           C
ATOM   2924  CG1 VAL A 373     -21.594  -9.423  37.811  1.00 27.81           C
ATOM   2925  CG2 VAL A 373     -23.271  -8.017  36.564  1.00 25.54           C
ATOM   2926  N   PHE A 374     -22.878  -5.047  37.856  1.00 25.35           N
ATOM   2927  CA  PHE A 374     -22.738  -3.597  37.666  1.00 25.95           C
ATOM   2928  C   PHE A 374     -22.261  -2.928  38.950  1.00 26.20           C
ATOM   2929  O   PHE A 374     -21.593  -1.880  38.921  1.00 21.77           O
ATOM   2930  CB  PHE A 374     -24.083  -2.995  37.222  1.00 25.29           C
ATOM   2931  CG  PHE A 374     -24.338  -3.095  35.744  1.00 24.61           C
ATOM   2932  CD1 PHE A 374     -23.850  -4.157  35.010  1.00 25.83           C
ATOM   2933  CD2 PHE A 374     -25.029  -2.096  35.083  1.00 27.32           C
ATOM   2934  CE1 PHE A 374     -24.043  -4.218  33.648  1.00 27.69           C
```

```
ATOM   2935  CE2 PHE A 374     -25.224  -2.145  33.714  1.00 26.77           C
ATOM   2936  CZ  PHE A 374     -24.731  -3.208  32.999  1.00 27.96           C
ATOM   2937  N   ASP A 375     -22.562  -3.601  40.064  1.00 29.26           N
ATOM   2938  CA  ASP A 375     -22.212  -3.148  41.409  1.00 31.59           C
ATOM   2939  C   ASP A 375     -21.374  -4.155  42.225  1.00 29.82           C
ATOM   2940  O   ASP A 375     -21.614  -4.358  43.415  1.00 29.59           O
ATOM   2941  CB  ASP A 375     -23.477  -2.747  42.176  1.00 31.39           C
ATOM   2942  CG  ASP A 375     -23.954  -1.340  41.817  1.00 32.35           C
ATOM   2943  OD1 ASP A 375     -23.865  -0.935  40.627  1.00 30.78           O
ATOM   2944  OD2 ASP A 375     -24.511  -0.658  42.715  1.00 36.30           O1-
ATOM   2945  N   GLU A 376     -20.457  -4.836  41.539  1.00 28.50           N
ATOM   2946  CA  GLU A 376     -19.115  -5.035  42.053  1.00 26.94           C
ATOM   2947  C   GLU A 376     -18.043  -5.009  40.962  1.00 26.28           C
ATOM   2948  O   GLU A 376     -16.887  -5.333  41.245  1.00 28.07           O
ATOM   2949  CB  GLU A 376     -19.002  -6.334  42.848  1.00 29.11           C
ATOM   2950  CG  GLU A 376     -19.509  -7.591  42.165  1.00 29.86           C
ATOM   2951  CD  GLU A 376     -18.961  -7.787  40.769  1.00 28.06           C
ATOM   2952  OE1 GLU A 376     -17.736  -7.935  40.608  1.00 29.85           O
ATOM   2953  OE2 GLU A 376     -19.768  -7.812  39.828  1.00 27.54           O1-
ATOM   2954  N   PHE A 377     -18.435  -4.748  39.709  1.00 21.99           N
ATOM   2955  CA  PHE A 377     -17.479  -4.330  38.674  1.00 17.15           C
ATOM   2956  C   PHE A 377     -16.712  -3.219  39.354  1.00 17.60           C
ATOM   2957  O   PHE A 377     -15.582  -3.419  39.769  1.00 18.80           O
ATOM   2958  CB  PHE A 377     -18.158  -3.679  37.448  1.00 14.09           C
ATOM   2959  CG  PHE A 377     -18.727  -4.640  36.433  1.00  5.40           C
ATOM   2960  CD1 PHE A 377     -18.294  -5.932  36.335  1.00  3.20           C
ATOM   2961  CD2 PHE A 377     -19.749  -4.233  35.616  1.00  2.00           C
ATOM   2962  CE1 PHE A 377     -18.883  -6.791  35.456  1.00  2.00           C
ATOM   2963  CE2 PHE A 377     -20.325  -5.088  34.742  1.00  2.00           C
ATOM   2964  CZ  PHE A 377     -19.896  -6.362  34.666  1.00  2.00           C
ATOM   2965  N   LYS A 378     -17.484  -2.219  39.775  1.00 17.81           N
ATOM   2966  CA  LYS A 378     -17.005  -0.869  40.084  1.00 17.26           C
ATOM   2967  C   LYS A 378     -15.683  -0.699  40.831  1.00 15.51           C
ATOM   2968  O   LYS A 378     -15.089   0.362  40.777  1.00 14.50           O
ATOM   2969  CB  LYS A 378     -18.106  -0.082  40.813  1.00 17.88           C
ATOM   2970  CG  LYS A 378     -17.952   1.453  40.765  1.00 18.67           C
ATOM   2971  CD  LYS A 378     -19.143   2.216  41.408  1.00 16.76           C
ATOM   2972  CE  LYS A 378     -19.247   2.011  42.921  1.00 15.54           C
ATOM   2973  NZ  LYS A 378     -19.884   0.728  43.329  1.00  9.93           N1+
ATOM   2974  N   PRO A 379     -15.161  -1.749  41.468  1.00 15.24           N
ATOM   2975  CA  PRO A 379     -13.732  -1.558  41.696  1.00 17.61           C
ATOM   2976  C   PRO A 379     -12.965  -1.316  40.391  1.00 19.67           C
ATOM   2977  O   PRO A 379     -12.378  -0.234  40.207  1.00 20.74           O
ATOM   2978  CB  PRO A 379     -13.302  -2.852  42.408  1.00 17.69           C
ATOM   2979  CG  PRO A 379     -14.565  -3.698  42.537  1.00 15.99           C
ATOM   2980  CD  PRO A 379     -15.707  -2.740  42.404  1.00 15.75           C
ATOM   2981  N   LEU A 380     -13.078  -2.261  39.450  1.00 18.64           N
ATOM   2982  CA  LEU A 380     -12.334  -2.227  38.183  1.00 16.78           C
ATOM   2983  C   LEU A 380     -12.899  -1.233  37.170  1.00 16.37           C
ATOM   2984  O   LEU A 380     -13.224  -1.618  36.052  1.00 15.89           O
ATOM   2985  CB  LEU A 380     -12.274  -3.625  37.545  1.00 12.95           C
ATOM   2986  CG  LEU A 380     -13.247  -4.733  37.928  1.00 11.61           C
ATOM   2987  CD1 LEU A 380     -13.959  -5.227  36.703  1.00 10.69           C
ATOM   2988  CD2 LEU A 380     -12.504  -5.870  38.586  1.00 10.18           C
ATOM   2989  N   VAL A 381     -13.112   0.004  37.626  1.00 16.46           N
ATOM   2990  CA  VAL A 381     -13.610   1.151  36.844  1.00 16.90           C
ATOM   2991  C   VAL A 381     -13.242   2.411  37.648  1.00 17.54           C
ATOM   2992  O   VAL A 381     -13.279   3.526  37.143  1.00 18.24           O
ATOM   2993  CB  VAL A 381     -15.167   1.160  36.674  1.00 16.00           C
ATOM   2994  CG1 VAL A 381     -15.587   2.360  35.866  1.00 15.15           C
ATOM   2995  CG2 VAL A 381     -15.668  -0.096  35.995  1.00 15.14           C
ATOM   2996  N   GLU A 382     -12.936   2.228  38.926  1.00 19.02           N
ATOM   2997  CA  GLU A 382     -12.424   3.318  39.744  1.00 20.65           C
ATOM   2998  C   GLU A 382     -10.913   3.520  39.525  1.00 19.24           C
ATOM   2999  O   GLU A 382     -10.503   4.596  39.114  1.00 18.63           O
ATOM   3000  CB  GLU A 382     -12.740   3.080  41.245  1.00 24.33           C
ATOM   3001  CG  GLU A 382     -14.261   3.175  41.666  1.00 22.93           C
ATOM   3002  CD  GLU A 382     -14.712   4.589  42.012  1.00 23.13           C
ATOM   3003  OE1 GLU A 382     -13.975   5.273  42.751  1.00 26.43           O
ATOM   3004  OE2 GLU A 382     -15.776   5.038  41.527  1.00 21.00           O1-
ATOM   3005  N   GLU A 383     -10.115   2.456  39.602  1.00 20.27           N
```

ATOM	3006	CA	GLU	A	383	-8.643	2.609	39.553	1.00	20.93	C
ATOM	3007	C	GLU	A	383	-8.048	3.427	38.400	1.00	17.88	C
ATOM	3008	O	GLU	A	383	-7.189	4.253	38.640	1.00	18.41	O
ATOM	3009	CB	GLU	A	383	-7.916	1.246	39.643	1.00	24.93	C
ATOM	3010	CG	GLU	A	383	-6.405	1.261	39.272	1.00	22.82	C
ATOM	3011	CD	GLU	A	383	-5.485	1.591	40.440	1.00	22.48	C
ATOM	3012	OE1	GLU	A	383	-5.574	2.732	40.956	1.00	21.74	O
ATOM	3013	OE2	GLU	A	383	-4.621	0.737	40.781	1.00	19.05	O1-
ATOM	3014	N	PRO	A	384	-8.487	3.215	37.153	1.00	14.01	N
ATOM	3015	CA	PRO	A	384	-8.206	4.228	36.135	1.00	15.62	C
ATOM	3016	C	PRO	A	384	-8.687	5.643	36.491	1.00	18.77	C
ATOM	3017	O	PRO	A	384	-7.860	6.487	36.866	1.00	20.60	O
ATOM	3018	CB	PRO	A	384	-8.890	3.683	34.890	1.00	14.10	C
ATOM	3019	CG	PRO	A	384	-9.622	2.425	35.353	1.00	14.48	C
ATOM	3020	CD	PRO	A	384	-8.870	1.952	36.529	1.00	13.10	C
ATOM	3021	N	GLN	A	385	-10.005	5.890	36.426	1.00	19.75	N
ATOM	3022	CA	GLN	A	385	-10.623	7.176	36.806	1.00	19.44	C
ATOM	3023	C	GLN	A	385	-9.841	7.869	37.909	1.00	19.08	C
ATOM	3024	O	GLN	A	385	-9.943	9.075	38.105	1.00	19.58	O
ATOM	3025	CB	GLN	A	385	-12.068	6.967	37.300	1.00	18.93	C
ATOM	3026	CG	GLN	A	385	-13.135	7.054	36.222	1.00	22.91	C
ATOM	3027	CD	GLN	A	385	-14.452	7.633	36.729	1.00	27.37	C
ATOM	3028	NE2	GLN	A	385	-14.359	8.627	37.618	1.00	29.27	N
ATOM	3029	OE1	GLN	A	385	-15.538	7.269	36.248	1.00	23.96	O
ATOM	3030	N	ASN	A	386	-9.198	7.062	38.735	1.00	19.76	N
ATOM	3031	CA	ASN	A	386	-8.384	7.586	39.800	1.00	22.19	C
ATOM	3032	C	ASN	A	386	-6.935	7.815	39.379	1.00	20.96	C
ATOM	3033	O	ASN	A	386	-6.552	8.967	39.235	1.00	20.97	O
ATOM	3034	CB	ASN	A	386	-8.524	6.695	41.050	1.00	23.37	C
ATOM	3035	CG	ASN	A	386	-9.864	6.933	41.789	1.00	24.44	C
ATOM	3036	ND2	ASN	A	386	-10.081	8.176	42.227	1.00	19.73	N
ATOM	3037	OD1	ASN	A	386	-10.734	6.045	41.845	1.00	22.44	O
ATOM	3038	N	LEU	A	387	-6.223	6.749	38.991	1.00	20.99	N
ATOM	3039	CA	LEU	A	387	-4.777	6.761	38.639	1.00	19.03	C
ATOM	3040	C	LEU	A	387	-4.344	7.967	37.800	1.00	19.94	C
ATOM	3041	O	LEU	A	387	-3.326	8.605	38.088	1.00	15.85	O
ATOM	3042	CB	LEU	A	387	-4.387	5.459	37.888	1.00	16.60	C
ATOM	3043	CG	LEU	A	387	-2.952	5.202	37.368	1.00	14.35	C
ATOM	3044	CD1	LEU	A	387	-2.328	3.996	38.035	1.00	8.49	C
ATOM	3045	CD2	LEU	A	387	-2.974	4.987	35.868	1.00	12.25	C
ATOM	3046	N	ILE	A	388	-5.183	8.336	36.838	1.00	21.75	N
ATOM	3047	CA	ILE	A	388	-4.984	9.544	36.034	1.00	24.55	C
ATOM	3048	C	ILE	A	388	-5.094	10.847	36.880	1.00	26.81	C
ATOM	3049	O	ILE	A	388	-5.268	11.943	36.341	1.00	26.63	O
ATOM	3050	CB	ILE	A	388	-6.000	9.592	34.822	1.00	22.71	C
ATOM	3051	CG1	ILE	A	388	-6.237	8.196	34.243	1.00	18.19	C
ATOM	3052	CG2	ILE	A	388	-5.417	10.407	33.692	1.00	22.80	C
ATOM	3053	CD1	ILE	A	388	-4.998	7.574	33.621	1.00	16.41	C
ATOM	3054	N	LYS	A	389	-5.028	10.699	38.205	1.00	29.41	N
ATOM	3055	CA	LYS	A	389	-4.770	11.788	39.136	1.00	30.13	C
ATOM	3056	C	LYS	A	389	-3.251	11.818	39.376	1.00	30.26	C
ATOM	3057	O	LYS	A	389	-2.558	12.722	38.899	1.00	30.98	O
ATOM	3058	CB	LYS	A	389	-5.517	11.556	40.468	1.00	33.95	C
ATOM	3059	CG	LYS	A	389	-5.040	12.458	41.657	1.00	35.82	C
ATOM	3060	CD	LYS	A	389	-5.674	12.085	43.010	1.00	34.70	C
ATOM	3061	CE	LYS	A	389	-6.883	12.973	43.356	1.00	35.61	C
ATOM	3062	NZ	LYS	A	389	-8.212	12.441	42.868	1.00	33.54	N1+
ATOM	3063	N	GLN	A	390	-2.709	10.787	40.017	1.00	29.25	N
ATOM	3064	CA	GLN	A	390	-1.259	10.707	40.162	1.00	28.68	C
ATOM	3065	C	GLN	A	390	-0.642	10.117	38.874	1.00	27.03	C
ATOM	3066	O	GLN	A	390	0.333	9.360	38.911	1.00	26.91	O
ATOM	3067	CB	GLN	A	390	-0.882	9.887	41.406	1.00	29.73	C
ATOM	3068	CG	GLN	A	390	0.416	10.334	42.107	1.00	28.58	C
ATOM	3069	CD	GLN	A	390	0.193	11.454	43.108	1.00	29.76	C
ATOM	3070	NE2	GLN	A	390	-0.114	11.089	44.353	1.00	30.78	N
ATOM	3071	OE1	GLN	A	390	0.315	12.635	42.775	1.00	30.93	O
ATOM	3072	N	ASN	A	391	-1.358	10.288	37.772	1.00	24.04	N
ATOM	3073	CA	ASN	A	391	-0.718	10.582	36.515	1.00	22.15	C
ATOM	3074	C	ASN	A	391	-0.857	12.091	36.495	1.00	18.92	C
ATOM	3075	O	ASN	A	391	-0.065	12.773	37.132	1.00	18.87	O
ATOM	3076	CB	ASN	A	391	-1.487	9.959	35.334	1.00	28.55	C

ATOM	3077	CG	ASN	A	391	-0.680	8.877	34.571	1.00	33.87	C
ATOM	3078	ND2	ASN	A	391	0.535	8.555	35.050	1.00	36.03	N
ATOM	3079	OD1	ASN	A	391	-1.189	8.289	33.605	1.00	33.57	O
ATOM	3080	N	CYS	A	392	-2.048	12.556	36.135	1.00	16.20	N
ATOM	3081	CA	CYS	A	392	-2.222	13.923	35.670	1.00	13.67	C
ATOM	3082	C	CYS	A	392	-2.326	14.985	36.745	1.00	13.13	C
ATOM	3083	O	CYS	A	392	-2.971	16.008	36.549	1.00	15.19	O
ATOM	3084	CB	CYS	A	392	-3.420	14.004	34.730	1.00	11.64	C
ATOM	3085	SG	CYS	A	392	-3.165	13.048	33.210	1.00	12.44	S
ATOM	3086	N	GLU	A	393	-1.503	14.864	37.768	1.00	14.75	N
ATOM	3087	CA	GLU	A	393	-1.268	16.000	38.653	1.00	19.49	C
ATOM	3088	C	GLU	A	393	0.227	16.228	38.975	1.00	20.11	C
ATOM	3089	O	GLU	A	393	0.754	17.335	38.787	1.00	18.94	O
ATOM	3090	CB	GLU	A	393	-2.081	15.840	39.940	1.00	20.40	C
ATOM	3091	CG	GLU	A	393	-2.403	17.152	40.613	1.00	20.82	C
ATOM	3092	CD	GLU	A	393	-3.414	16.997	41.713	1.00	22.54	C
ATOM	3093	OE1	GLU	A	393	-3.048	16.456	42.785	1.00	24.39	O
ATOM	3094	OE2	GLU	A	393	-4.574	17.418	41.505	1.00	24.30	O1-
ATOM	3095	N	LEU	A	394	0.908	15.165	39.396	1.00	19.67	N
ATOM	3096	CA	LEU	A	394	2.353	15.160	39.518	1.00	21.83	C
ATOM	3097	C	LEU	A	394	2.947	15.795	38.259	1.00	24.26	C
ATOM	3098	O	LEU	A	394	3.959	16.503	38.286	1.00	26.00	O
ATOM	3099	CB	LEU	A	394	2.839	13.722	39.646	1.00	21.88	C
ATOM	3100	CG	LEU	A	394	4.062	13.487	40.531	1.00	23.92	C
ATOM	3101	CD1	LEU	A	394	5.242	14.340	40.026	1.00	25.67	C
ATOM	3102	CD2	LEU	A	394	3.724	13.789	41.997	1.00	21.11	C
ATOM	3103	N	PHE	A	395	2.277	15.598	37.142	1.00	25.37	N
ATOM	3104	CA	PHE	A	395	2.708	16.304	35.965	1.00	25.77	C
ATOM	3105	C	PHE	A	395	2.196	17.746	35.907	1.00	26.16	C
ATOM	3106	O	PHE	A	395	2.988	18.663	36.144	1.00	26.53	O
ATOM	3107	CB	PHE	A	395	2.361	15.469	34.744	1.00	25.66	C
ATOM	3108	CG	PHE	A	395	3.224	14.254	34.625	1.00	26.69	C
ATOM	3109	CD1	PHE	A	395	4.603	14.367	34.780	1.00	26.93	C
ATOM	3110	CD2	PHE	A	395	2.676	13.000	34.460	1.00	26.92	C
ATOM	3111	CE1	PHE	A	395	5.417	13.264	34.773	1.00	25.90	C
ATOM	3112	CE2	PHE	A	395	3.487	11.886	34.451	1.00	29.35	C
ATOM	3113	CZ	PHE	A	395	4.867	12.022	34.609	1.00	28.67	C
ATOM	3114	N	GLU	A	396	0.868	17.902	35.930	1.00	24.62	N
ATOM	3115	CA	GLU	A	396	0.191	19.186	35.745	1.00	22.66	C
ATOM	3116	C	GLU	A	396	0.982	20.346	36.329	1.00	24.98	C
ATOM	3117	O	GLU	A	396	1.905	20.842	35.684	1.00	25.79	O
ATOM	3118	CB	GLU	A	396	-1.209	19.129	36.363	1.00	21.41	C
ATOM	3119	CG	GLU	A	396	-2.062	20.342	36.075	1.00	18.86	C
ATOM	3120	CD	GLU	A	396	-3.511	20.135	36.448	1.00	19.66	C
ATOM	3121	OE1	GLU	A	396	-4.119	19.159	35.965	1.00	21.53	O
ATOM	3122	OE2	GLU	A	396	-4.056	20.951	37.210	1.00	16.14	O1-
ATOM	3123	N	GLN	A	397	0.686	20.717	37.577	1.00	28.23	N
ATOM	3124	CA	GLN	A	397	1.460	21.738	38.288	1.00	27.32	C
ATOM	3125	C	GLN	A	397	2.530	20.945	38.997	1.00	25.36	C
ATOM	3126	O	GLN	A	397	2.351	20.612	40.158	1.00	22.92	O
ATOM	3127	CB	GLN	A	397	0.595	22.527	39.326	1.00	28.36	C
ATOM	3128	CG	GLN	A	397	1.354	23.682	40.097	1.00	26.26	C
ATOM	3129	CD	GLN	A	397	0.487	24.528	41.064	1.00	25.26	C
ATOM	3130	NE2	GLN	A	397	-0.812	24.290	41.070	1.00	27.07	N
ATOM	3131	OE1	GLN	A	397	1.011	25.332	41.847	1.00	24.51	O
ATOM	3132	N	LEU	A	398	3.461	20.405	38.210	1.00	25.94	N
ATOM	3133	CA	LEU	A	398	4.677	19.792	38.751	1.00	26.57	C
ATOM	3134	C	LEU	A	398	5.577	19.106	37.699	1.00	24.32	C
ATOM	3135	O	LEU	A	398	6.460	18.340	38.071	1.00	22.18	O
ATOM	3136	CB	LEU	A	398	4.328	18.793	39.899	1.00	29.28	C
ATOM	3137	CG	LEU	A	398	4.839	18.900	41.365	1.00	29.60	C
ATOM	3138	CD1	LEU	A	398	4.218	20.072	42.135	1.00	29.70	C
ATOM	3139	CD2	LEU	A	398	4.513	17.604	42.113	1.00	27.39	C
ATOM	3140	N	GLY	A	399	5.407	19.409	36.409	1.00	24.36	N
ATOM	3141	CA	GLY	A	399	6.447	19.039	35.442	1.00	26.34	C
ATOM	3142	C	GLY	A	399	6.243	18.217	34.146	1.00	25.35	C
ATOM	3143	O	GLY	A	399	6.431	16.983	34.105	1.00	24.71	O
ATOM	3144	N	GLU	A	400	6.272	18.954	33.041	1.00	23.31	N
ATOM	3145	CA	GLU	A	400	6.309	18.381	31.700	1.00	21.85	C
ATOM	3146	C	GLU	A	400	7.499	17.394	31.494	1.00	21.88	C
ATOM	3147	O	GLU	A	400	7.351	16.186	31.628	1.00	20.25	O

ATOM	3148	CB	GLU	A	400	6.389	19.544	30.704	1.00	18.99	C
ATOM	3149	CG	GLU	A	400	5.051	20.164	30.301	1.00	18.80	C
ATOM	3150	CD	GLU	A	400	4.368	21.016	31.372	1.00	15.88	C
ATOM	3151	OE1	GLU	A	400	4.576	20.786	32.574	1.00	13.73	O
ATOM	3152	OE2	GLU	A	400	3.524	21.862	30.999	1.00	14.44	O1-
ATOM	3153	N	TYR	A	401	8.701	17.951	31.356	1.00	22.47	N
ATOM	3154	CA	TYR	A	401	9.926	17.216	31.020	1.00	20.38	C
ATOM	3155	C	TYR	A	401	10.935	17.239	32.181	1.00	19.52	C
ATOM	3156	O	TYR	A	401	12.064	16.790	32.033	1.00	18.53	O
ATOM	3157	CB	TYR	A	401	10.554	17.881	29.797	1.00	23.78	C
ATOM	3158	CG	TYR	A	401	11.448	16.997	28.969	1.00	25.15	C
ATOM	3159	CD1	TYR	A	401	12.748	16.752	29.363	1.00	25.90	C
ATOM	3160	CD2	TYR	A	401	11.060	16.579	27.706	1.00	25.00	C
ATOM	3161	CE1	TYR	A	401	13.641	16.158	28.525	1.00	27.01	C
ATOM	3162	CE2	TYR	A	401	11.948	15.979	26.863	1.00	26.20	C
ATOM	3163	CZ	TYR	A	401	13.250	15.790	27.270	1.00	26.58	C
ATOM	3164	OH	TYR	A	401	14.209	15.388	26.375	1.00	22.64	O
ATOM	3165	N	LYS	A	402	10.621	18.021	33.211	1.00	22.60	N
ATOM	3166	CA	LYS	A	402	11.307	17.936	34.503	1.00	22.71	C
ATOM	3167	C	LYS	A	402	10.981	16.574	35.103	1.00	24.22	C
ATOM	3168	O	LYS	A	402	11.543	15.565	34.676	1.00	25.37	O
ATOM	3169	CB	LYS	A	402	10.845	19.067	35.435	1.00	20.39	C
ATOM	3170	CG	LYS	A	402	11.323	18.957	36.876	1.00	17.50	C
ATOM	3171	CD	LYS	A	402	12.633	19.686	37.105	1.00	17.85	C
ATOM	3172	CE	LYS	A	402	13.838	18.750	37.135	1.00	17.83	C
ATOM	3173	NZ	LYS	A	402	15.068	19.486	37.556	1.00	19.64	N1+
ATOM	3174	N	PHE	A	403	9.954	16.495	35.941	1.00	25.18	N
ATOM	3175	CA	PHE	A	403	9.679	15.217	36.562	1.00	28.48	C
ATOM	3176	C	PHE	A	403	8.640	14.362	35.850	1.00	30.15	C
ATOM	3177	O	PHE	A	403	7.621	13.986	36.423	1.00	30.31	O
ATOM	3178	CB	PHE	A	403	9.370	15.398	38.045	1.00	28.85	C
ATOM	3179	CG	PHE	A	403	10.610	15.601	38.886	1.00	30.05	C
ATOM	3180	CD1	PHE	A	403	11.155	16.860	39.037	1.00	28.78	C
ATOM	3181	CD2	PHE	A	403	11.326	14.511	39.371	1.00	29.76	C
ATOM	3182	CE1	PHE	A	403	12.393	17.026	39.632	1.00	28.52	C
ATOM	3183	CE2	PHE	A	403	12.561	14.677	39.967	1.00	26.34	C
ATOM	3184	CZ	PHE	A	403	13.097	15.928	40.092	1.00	26.88	C
ATOM	3185	N	GLN	A	404	8.911	14.106	34.565	1.00	32.37	N
ATOM	3186	CA	GLN	A	404	8.532	12.852	33.887	1.00	30.49	C
ATOM	3187	C	GLN	A	404	9.506	11.781	34.350	1.00	29.27	C
ATOM	3188	O	GLN	A	404	9.194	10.601	34.304	1.00	29.71	O
ATOM	3189	CB	GLN	A	404	8.648	12.975	32.361	1.00	31.75	C
ATOM	3190	CG	GLN	A	404	10.038	12.639	31.796	1.00	30.57	C
ATOM	3191	CD	GLN	A	404	10.134	12.873	30.298	1.00	31.42	C
ATOM	3192	NE2	GLN	A	404	9.715	11.891	29.507	1.00	30.06	N
ATOM	3193	OE1	GLN	A	404	10.548	13.942	29.858	1.00	31.82	O
ATOM	3194	N	ASN	A	405	10.695	12.196	34.786	1.00	27.19	N
ATOM	3195	CA	ASN	A	405	11.561	11.290	35.527	1.00	24.01	C
ATOM	3196	C	ASN	A	405	11.159	11.181	37.030	1.00	21.70	C
ATOM	3197	O	ASN	A	405	11.983	11.211	37.946	1.00	15.16	O
ATOM	3198	CB	ASN	A	405	13.033	11.663	35.278	1.00	22.91	C
ATOM	3199	CG	ASN	A	405	13.611	12.577	36.314	1.00	19.47	C
ATOM	3200	ND2	ASN	A	405	13.058	13.770	36.427	1.00	19.73	N
ATOM	3201	OD1	ASN	A	405	14.666	12.283	36.866	1.00	17.07	O
ATOM	3202	N	ALA	A	406	9.852	10.999	37.231	1.00	19.67	N
ATOM	3203	CA	ALA	A	406	9.294	10.467	38.458	1.00	18.79	C
ATOM	3204	C	ALA	A	406	8.941	9.011	38.194	1.00	20.96	C
ATOM	3205	O	ALA	A	406	9.168	8.141	39.044	1.00	23.15	O
ATOM	3206	CB	ALA	A	406	8.048	11.228	38.841	1.00	15.73	C
ATOM	3207	N	LEU	A	407	8.393	8.731	37.013	1.00	19.76	N
ATOM	3208	CA	LEU	A	407	7.955	7.379	36.692	1.00	16.89	C
ATOM	3209	C	LEU	A	407	9.136	6.433	36.519	1.00	17.14	C
ATOM	3210	O	LEU	A	407	8.978	5.221	36.655	1.00	15.99	O
ATOM	3211	CB	LEU	A	407	7.086	7.393	35.444	1.00	16.01	C
ATOM	3212	CG	LEU	A	407	5.659	7.835	35.763	1.00	18.48	C
ATOM	3213	CD1	LEU	A	407	4.978	8.430	34.540	1.00	20.67	C
ATOM	3214	CD2	LEU	A	407	4.871	6.641	36.285	1.00	20.49	C
ATOM	3215	N	LEU	A	408	10.317	6.998	36.250	1.00	18.02	N
ATOM	3216	CA	LEU	A	408	11.617	6.307	36.411	1.00	18.24	C
ATOM	3217	C	LEU	A	408	11.824	5.869	37.872	1.00	18.70	C
ATOM	3218	O	LEU	A	408	11.811	4.687	38.190	1.00	17.33	O

ATOM	3219	CB	LEU	A	408	12.767	7.250	36.018	1.00	18.19	C
ATOM	3220	CG	LEU	A	408	13.659	7.027	34.797	1.00	16.19	C
ATOM	3221	CD1	LEU	A	408	14.511	8.259	34.575	1.00	11.96	C
ATOM	3222	CD2	LEU	A	408	14.539	5.807	34.995	1.00	16.05	C
ATOM	3223	N	VAL	A	409	11.989	6.846	38.759	1.00	20.54	N
ATOM	3224	CA	VAL	A	409	12.091	6.603	40.193	1.00	20.69	C
ATOM	3225	C	VAL	A	409	11.118	5.538	40.642	1.00	21.47	C
ATOM	3226	O	VAL	A	409	11.487	4.672	41.418	1.00	23.58	O
ATOM	3227	CB	VAL	A	409	11.797	7.867	41.012	1.00	19.59	C
ATOM	3228	CG1	VAL	A	409	11.897	7.549	42.518	1.00	19.23	C
ATOM	3229	CG2	VAL	A	409	12.753	8.975	40.613	1.00	17.46	C
ATOM	3230	N	ARG	A	410	9.879	5.610	40.163	1.00	21.93	N
ATOM	3231	CA	ARG	A	410	8.889	4.593	40.488	1.00	21.95	C
ATOM	3232	C	ARG	A	410	9.369	3.263	39.930	1.00	21.48	C
ATOM	3233	O	ARG	A	410	9.723	2.363	40.692	1.00	24.94	O
ATOM	3234	CB	ARG	A	410	7.506	4.939	39.904	1.00	22.67	C
ATOM	3235	CG	ARG	A	410	6.846	6.205	40.445	1.00	20.28	C
ATOM	3236	CD	ARG	A	410	5.357	6.220	40.114	1.00	20.80	C
ATOM	3237	NE	ARG	A	410	4.733	7.522	40.365	1.00	20.80	N
ATOM	3238	CZ	ARG	A	410	3.487	7.841	40.011	1.00	20.59	C
ATOM	3239	NH1	ARG	A	410	2.705	6.941	39.426	1.00	21.04	N1+
ATOM	3240	NH2	ARG	A	410	3.025	9.067	40.234	1.00	20.07	N
ATOM	3241	N	TYR	A	411	9.529	3.186	38.615	1.00	18.76	N
ATOM	3242	CA	TYR	A	411	9.792	1.907	37.974	1.00	17.50	C
ATOM	3243	C	TYR	A	411	11.169	1.356	38.330	1.00	17.38	C
ATOM	3244	O	TYR	A	411	11.289	0.240	38.823	1.00	15.90	O
ATOM	3245	CB	TYR	A	411	9.606	2.024	36.453	1.00	18.41	C
ATOM	3246	CG	TYR	A	411	8.151	1.974	36.064	1.00	17.19	C
ATOM	3247	CD1	TYR	A	411	7.215	2.657	36.815	1.00	15.09	C
ATOM	3248	CD2	TYR	A	411	7.684	1.060	35.137	1.00	18.38	C
ATOM	3249	CE1	TYR	A	411	5.885	2.431	36.681	1.00	11.66	C
ATOM	3250	CE2	TYR	A	411	6.325	0.829	34.998	1.00	15.51	C
ATOM	3251	CZ	TYR	A	411	5.436	1.536	35.776	1.00	12.43	C
ATOM	3252	OH	TYR	A	411	4.079	1.476	35.573	1.00	16.59	O
ATOM	3253	N	THR	A	412	12.186	2.195	38.234	1.00	19.91	N
ATOM	3254	CA	THR	A	412	13.541	1.791	38.601	1.00	24.09	C
ATOM	3255	C	THR	A	412	13.591	1.176	39.988	1.00	23.32	C
ATOM	3256	O	THR	A	412	14.583	0.526	40.342	1.00	22.82	O
ATOM	3257	CB	THR	A	412	14.510	2.978	38.590	1.00	26.90	C
ATOM	3258	CG2	THR	A	412	15.900	2.514	38.214	1.00	29.28	C
ATOM	3259	OG1	THR	A	412	14.063	3.952	37.634	1.00	31.00	O
ATOM	3260	N	LYS	A	413	12.580	1.492	40.802	1.00	21.28	N
ATOM	3261	CA	LYS	A	413	12.512	1.006	42.170	1.00	19.27	C
ATOM	3262	C	LYS	A	413	11.796	-0.322	42.158	1.00	19.80	C
ATOM	3263	O	LYS	A	413	12.418	-1.382	42.340	1.00	20.91	O
ATOM	3264	CB	LYS	A	413	11.768	1.999	43.068	1.00	16.46	C
ATOM	3265	CG	LYS	A	413	12.670	2.713	44.084	1.00	17.32	C
ATOM	3266	CD	LYS	A	413	11.899	3.127	45.323	1.00	18.80	C
ATOM	3267	CE	LYS	A	413	11.409	4.570	45.242	1.00	21.30	C
ATOM	3268	NZ	LYS	A	413	12.515	5.551	45.015	1.00	20.06	N1+
ATOM	3269	N	LYS	A	414	10.531	-0.282	41.752	1.00	18.33	N
ATOM	3270	CA	LYS	A	414	9.678	-1.445	41.909	1.00	16.94	C
ATOM	3271	C	LYS	A	414	10.083	-2.597	41.015	1.00	17.20	C
ATOM	3272	O	LYS	A	414	9.744	-3.742	41.285	1.00	17.03	O
ATOM	3273	CB	LYS	A	414	8.192	-1.098	41.704	1.00	17.27	C
ATOM	3274	CG	LYS	A	414	7.823	-0.088	40.645	1.00	12.68	C
ATOM	3275	CD	LYS	A	414	6.341	-0.276	40.311	1.00	14.55	C
ATOM	3276	CE	LYS	A	414	5.798	0.739	39.303	1.00	14.56	C
ATOM	3277	NZ	LYS	A	414	5.337	2.037	39.917	1.00	14.02	N1+
ATOM	3278	N	VAL	A	415	10.934	-2.302	40.043	1.00	17.01	N
ATOM	3279	CA	VAL	A	415	11.369	-3.287	39.070	1.00	16.75	C
ATOM	3280	C	VAL	A	415	12.780	-2.914	38.581	1.00	21.86	C
ATOM	3281	O	VAL	A	415	12.949	-2.362	37.490	1.00	23.99	O
ATOM	3282	CB	VAL	A	415	10.373	-3.353	37.897	1.00	12.19	C
ATOM	3283	CG1	VAL	A	415	10.301	-2.032	37.199	1.00	10.88	C
ATOM	3284	CG2	VAL	A	415	10.754	-4.432	36.947	1.00	9.96	C
ATOM	3285	N	PRO	A	416	13.817	-3.194	39.401	1.00	23.73	N
ATOM	3286	CA	PRO	A	416	15.121	-2.556	39.239	1.00	23.14	C
ATOM	3287	C	PRO	A	416	16.129	-3.485	38.583	1.00	22.45	C
ATOM	3288	O	PRO	A	416	17.310	-3.467	38.920	1.00	20.59	O
ATOM	3289	CB	PRO	A	416	15.513	-2.221	40.679	1.00	26.64	C

ATOM	3290	CG	PRO	A	416	14.755	-3.262	41.552	1.00	24.34	C
ATOM	3291	CD	PRO	A	416	13.777	-3.984	40.645	1.00	25.02	C
ATOM	3292	N	GLN	A	417	15.653	-4.338	37.686	1.00	24.78	N
ATOM	3293	CA	GLN	A	417	16.554	-5.178	36.894	1.00	25.39	C
ATOM	3294	C	GLN	A	417	16.386	-5.115	35.367	1.00	25.68	C
ATOM	3295	O	GLN	A	417	17.256	-5.608	34.646	1.00	28.59	O
ATOM	3296	CB	GLN	A	417	16.476	-6.640	37.343	1.00	22.92	C
ATOM	3297	CG	GLN	A	417	17.445	-6.998	38.437	1.00	16.76	C
ATOM	3298	CD	GLN	A	417	16.729	-7.293	39.727	1.00	19.80	C
ATOM	3299	NE2	GLN	A	417	17.337	-8.118	40.561	1.00	17.72	N
ATOM	3300	OE1	GLN	A	417	15.618	-6.796	39.965	1.00	20.09	O
ATOM	3301	N	VAL	A	418	15.284	-4.562	34.862	1.00	23.83	N
ATOM	3302	CA	VAL	A	418	15.206	-4.262	33.420	1.00	25.79	C
ATOM	3303	C	VAL	A	418	16.443	-3.398	33.023	1.00	25.27	C
ATOM	3304	O	VAL	A	418	17.075	-2.784	33.886	1.00	26.72	O
ATOM	3305	CB	VAL	A	418	13.872	-3.506	33.057	1.00	24.98	C
ATOM	3306	CG1	VAL	A	418	13.516	-3.697	31.597	1.00	21.82	C
ATOM	3307	CG2	VAL	A	418	12.720	-4.010	33.907	1.00	25.57	C
ATOM	3308	N	SER	A	419	16.865	-3.451	31.761	1.00	23.89	N
ATOM	3309	CA	SER	A	419	18.115	-2.791	31.338	1.00	24.81	C
ATOM	3310	C	SER	A	419	18.045	-1.274	31.448	1.00	23.00	C
ATOM	3311	O	SER	A	419	17.072	-0.685	31.022	1.00	23.69	O
ATOM	3312	CB	SER	A	419	18.449	-3.159	29.888	1.00	25.20	C
ATOM	3313	OG	SER	A	419	18.798	-4.528	29.762	1.00	28.67	O
ATOM	3314	N	THR	A	420	19.130	-0.623	31.854	1.00	24.58	N
ATOM	3315	CA	THR	A	420	19.075	0.829	32.039	1.00	24.29	C
ATOM	3316	C	THR	A	420	18.501	1.555	30.855	1.00	24.81	C
ATOM	3317	O	THR	A	420	17.600	2.367	31.013	1.00	26.08	O
ATOM	3318	CB	THR	A	420	20.425	1.453	32.348	1.00	21.78	C
ATOM	3319	CG2	THR	A	420	20.375	2.953	32.130	1.00	21.26	C
ATOM	3320	OG1	THR	A	420	20.763	1.193	33.715	1.00	24.19	O
ATOM	3321	N	PRO	A	421	18.946	1.222	29.642	1.00	25.02	N
ATOM	3322	CA	PRO	A	421	18.193	1.830	28.545	1.00	26.98	C
ATOM	3323	C	PRO	A	421	16.704	1.458	28.629	1.00	27.18	C
ATOM	3324	O	PRO	A	421	15.850	2.349	28.768	1.00	25.10	O
ATOM	3325	CB	PRO	A	421	18.867	1.272	27.282	1.00	27.79	C
ATOM	3326	CG	PRO	A	421	19.758	0.135	27.777	1.00	29.44	C
ATOM	3327	CD	PRO	A	421	20.152	0.531	29.167	1.00	25.03	C
ATOM	3328	N	THR	A	422	16.427	0.149	28.728	1.00	26.36	N
ATOM	3329	CA	THR	A	422	15.064	-0.393	28.605	1.00	24.58	C
ATOM	3330	C	THR	A	422	14.162	0.069	29.744	1.00	24.87	C
ATOM	3331	O	THR	A	422	13.011	-0.349	29.839	1.00	27.96	O
ATOM	3332	CB	THR	A	422	15.058	-1.947	28.560	1.00	21.16	C
ATOM	3333	CG2	THR	A	422	13.682	-2.465	28.189	1.00	21.32	C
ATOM	3334	OG1	THR	A	422	15.985	-2.411	27.570	1.00	20.86	O
ATOM	3335	N	LEU	A	423	14.745	0.778	30.703	1.00	25.19	N
ATOM	3336	CA	LEU	A	423	13.976	1.651	31.558	1.00	22.20	C
ATOM	3337	C	LEU	A	423	13.860	2.946	30.755	1.00	22.66	C
ATOM	3338	O	LEU	A	423	12.860	3.113	30.062	1.00	22.77	O
ATOM	3339	CB	LEU	A	423	14.689	1.889	32.891	1.00	23.24	C
ATOM	3340	CG	LEU	A	423	14.626	0.885	34.065	1.00	22.20	C
ATOM	3341	CD1	LEU	A	423	13.289	0.159	34.098	1.00	19.45	C
ATOM	3342	CD2	LEU	A	423	15.788	-0.093	33.971	1.00	18.12	C
ATOM	3343	N	VAL	A	424	14.969	3.678	30.592	1.00	22.40	N
ATOM	3344	CA	VAL	A	424	14.941	5.007	29.944	1.00	19.48	C
ATOM	3345	C	VAL	A	424	14.315	5.097	28.554	1.00	21.48	C
ATOM	3346	O	VAL	A	424	14.119	6.211	28.067	1.00	23.47	O
ATOM	3347	CB	VAL	A	424	16.337	5.707	29.809	1.00	17.06	C
ATOM	3348	CG1	VAL	A	424	16.141	7.204	29.867	1.00	9.48	C
ATOM	3349	CG2	VAL	A	424	17.336	5.264	30.890	1.00	14.26	C
ATOM	3350	N	GLU	A	425	13.977	3.977	27.908	1.00	20.71	N
ATOM	3351	CA	GLU	A	425	13.114	4.076	26.724	1.00	20.21	C
ATOM	3352	C	GLU	A	425	11.679	4.209	27.187	1.00	21.09	C
ATOM	3353	O	GLU	A	425	10.983	5.151	26.790	1.00	22.27	O
ATOM	3354	CB	GLU	A	425	13.231	2.868	25.790	1.00	19.48	C
ATOM	3355	CG	GLU	A	425	13.577	3.210	24.334	1.00	17.47	C
ATOM	3356	CD	GLU	A	425	12.582	4.152	23.653	1.00	17.36	C
ATOM	3357	OE1	GLU	A	425	12.703	5.386	23.801	1.00	16.77	O
ATOM	3358	OE2	GLU	A	425	11.746	3.674	22.860	1.00	20.99	O1-
ATOM	3359	N	VAL	A	426	11.277	3.332	28.108	1.00	20.45	N
ATOM	3360	CA	VAL	A	426	9.940	3.379	28.710	1.00	18.52	C

```
ATOM   3361  C   VAL A 426       9.831   4.594  29.631  1.00 17.11           C
ATOM   3362  O   VAL A 426       9.014   5.466  29.407  1.00 19.30           O
ATOM   3363  CB  VAL A 426       9.662   2.131  29.556  1.00 17.57           C
ATOM   3364  CG1 VAL A 426       8.194   1.980  29.795  1.00 17.28           C
ATOM   3365  CG2 VAL A 426      10.227   0.911  28.878  1.00 20.59           C
ATOM   3366  N   SER A 427      10.693   4.664  30.635  1.00 14.13           N
ATOM   3367  CA  SER A 427      10.598   5.670  31.676  1.00 14.17           C
ATOM   3368  C   SER A 427      10.109   7.012  31.164  1.00 13.54           C
ATOM   3369  O   SER A 427       9.024   7.432  31.522  1.00 11.17           O
ATOM   3370  CB  SER A 427      11.950   5.834  32.356  1.00 17.92           C
ATOM   3371  OG  SER A 427      12.541   4.586  32.668  1.00 14.61           O
ATOM   3372  N   ARG A 428      10.786   7.547  30.152  1.00 15.97           N
ATOM   3373  CA  ARG A 428      10.307   8.758  29.451  1.00 19.71           C
ATOM   3374  C   ARG A 428       9.104   8.551  28.504  1.00 19.67           C
ATOM   3375  O   ARG A 428       8.208   9.395  28.438  1.00 20.46           O
ATOM   3376  CB  ARG A 428      11.447   9.463  28.679  1.00 17.27           C
ATOM   3377  CG  ARG A 428      12.600   8.580  28.234  1.00 13.84           C
ATOM   3378  CD  ARG A 428      13.345   9.187  27.075  1.00 12.03           C
ATOM   3379  NE  ARG A 428      13.832  10.531  27.369  1.00 13.38           N
ATOM   3380  CZ  ARG A 428      13.437  11.648  26.750  1.00 12.65           C
ATOM   3381  NH1 ARG A 428      12.447  11.635  25.863  1.00  7.48           N1+
ATOM   3382  NH2 ARG A 428      14.028  12.800  27.040  1.00 10.10           N
ATOM   3383  N   ASN A 429       9.094   7.458  27.750  1.00 20.12           N
ATOM   3384  CA  ASN A 429       7.972   7.177  26.876  1.00 20.34           C
ATOM   3385  C   ASN A 429       6.612   7.140  27.579  1.00 20.43           C
ATOM   3386  O   ASN A 429       5.772   7.993  27.303  1.00 23.09           O
ATOM   3387  CB  ASN A 429       8.215   5.903  26.081  1.00 19.92           C
ATOM   3388  CG  ASN A 429       8.527   6.196  24.626  1.00 23.69           C
ATOM   3389  ND2 ASN A 429       9.313   7.253  24.394  1.00 24.44           N
ATOM   3390  OD1 ASN A 429       7.988   5.552  23.716  1.00 23.46           O
ATOM   3391  N   LEU A 430       6.418   6.233  28.542  1.00 18.98           N
ATOM   3392  CA  LEU A 430       5.201   6.222  29.366  1.00 13.10           C
ATOM   3393  C   LEU A 430       5.217   7.456  30.222  1.00 13.42           C
ATOM   3394  O   LEU A 430       4.270   7.738  30.921  1.00 14.99           O
ATOM   3395  CB  LEU A 430       5.120   4.968  30.239  1.00  7.34           C
ATOM   3396  CG  LEU A 430       5.092   4.992  31.769  1.00  2.61           C
ATOM   3397  CD1 LEU A 430       4.894   3.578  32.284  1.00  3.10           C
ATOM   3398  CD2 LEU A 430       6.381   5.518  32.313  1.00  2.00           C
ATOM   3399  N   GLY A 431       6.352   8.135  30.244  1.00 16.38           N
ATOM   3400  CA  GLY A 431       6.351   9.513  30.682  1.00 17.97           C
ATOM   3401  C   GLY A 431       5.371  10.263  29.810  1.00 17.99           C
ATOM   3402  O   GLY A 431       4.290  10.586  30.285  1.00 20.37           O
ATOM   3403  N   LYS A 432       5.621  10.252  28.499  1.00 16.66           N
ATOM   3404  CA  LYS A 432       4.947  11.127  27.524  1.00 15.91           C
ATOM   3405  C   LYS A 432       3.429  11.034  27.418  1.00 15.02           C
ATOM   3406  O   LYS A 432       2.850  11.519  26.453  1.00 11.29           O
ATOM   3407  CB  LYS A 432       5.525  10.900  26.135  1.00 16.80           C
ATOM   3408  CG  LYS A 432       7.003  11.220  25.997  1.00 18.19           C
ATOM   3409  CD  LYS A 432       7.359  11.418  24.526  1.00 19.26           C
ATOM   3410  CE  LYS A 432       8.406  10.405  24.082  1.00 22.95           C
ATOM   3411  NZ  LYS A 432       8.438  10.312  22.595  1.00 27.31           N1+
ATOM   3412  N   VAL A 433       2.802  10.333  28.356  1.00 16.94           N
ATOM   3413  CA  VAL A 433       1.353  10.328  28.507  1.00 15.68           C
ATOM   3414  C   VAL A 433       0.985  11.622  29.194  1.00 14.62           C
ATOM   3415  O   VAL A 433       0.140  12.354  28.707  1.00 18.83           O
ATOM   3416  CB  VAL A 433       0.878   9.124  29.363  1.00 16.06           C
ATOM   3417  CG1 VAL A 433      -0.188   9.552  30.376  1.00 15.00           C
ATOM   3418  CG2 VAL A 433       0.367   8.019  28.463  1.00 10.29           C
ATOM   3419  N   GLY A 434       1.701  11.967  30.251  1.00 13.40           N
ATOM   3420  CA  GLY A 434       1.392  13.190  30.969  1.00 17.13           C
ATOM   3421  C   GLY A 434       1.177  14.348  30.013  1.00 18.55           C
ATOM   3422  O   GLY A 434       0.108  14.946  29.981  1.00 17.84           O
ATOM   3423  N   SER A 435       2.091  14.472  29.063  1.00 20.65           N
ATOM   3424  CA  SER A 435       2.031  15.533  28.073  1.00 21.99           C
ATOM   3425  C   SER A 435       0.790  15.331  27.200  1.00 22.53           C
ATOM   3426  O   SER A 435      -0.251  15.929  27.471  1.00 22.40           O
ATOM   3427  CB  SER A 435       3.329  15.553  27.221  1.00 25.26           C
ATOM   3428  OG  SER A 435       4.523  15.746  28.007  1.00 22.37           O
ATOM   3429  N   LYS A 436       0.832  14.330  26.322  1.00 23.94           N
ATOM   3430  CA  LYS A 436      -0.157  14.181  25.250  1.00 25.08           C
ATOM   3431  C   LYS A 436      -1.528  13.626  25.661  1.00 24.18           C
```

```
ATOM   3432  O   LYS A 436      -2.216  13.046  24.827  1.00 26.29           O
ATOM   3433  CB  LYS A 436       0.410  13.295  24.120  1.00 27.26           C
ATOM   3434  CG  LYS A 436      -0.211  13.540  22.713  1.00 29.66           C
ATOM   3435  CD  LYS A 436      -0.836  12.276  22.091  1.00 28.08           C
ATOM   3436  CE  LYS A 436      -2.362  12.442  21.910  1.00 28.14           C
ATOM   3437  NZ  LYS A 436      -3.079  11.155  21.561  1.00 26.79           N1+
ATOM   3438  N   CYS A 437      -1.959  13.825  26.903  1.00 22.66           N
ATOM   3439  CA  CYS A 437      -3.299  13.371  27.287  1.00 22.80           C
ATOM   3440  C   CYS A 437      -3.940  14.098  28.461  1.00 22.95           C
ATOM   3441  O   CYS A 437      -5.118  14.394  28.420  1.00 23.92           O
ATOM   3442  CB  CYS A 437      -3.317  11.854  27.520  1.00 19.38           C
ATOM   3443  SG  CYS A 437      -3.502  10.914  25.959  1.00 24.98           S
ATOM   3444  N   CYS A 438      -3.170  14.449  29.484  1.00 23.70           N
ATOM   3445  CA  CYS A 438      -3.717  15.267  30.546  1.00 22.08           C
ATOM   3446  C   CYS A 438      -4.216  16.551  29.900  1.00 23.11           C
ATOM   3447  O   CYS A 438      -5.390  16.891  30.012  1.00 23.14           O
ATOM   3448  CB  CYS A 438      -2.650  15.582  31.595  1.00 19.90           C
ATOM   3449  SG  CYS A 438      -1.760  14.132  32.242  1.00 17.60           S
ATOM   3450  N   LYS A 439      -3.378  17.119  29.037  1.00 25.03           N
ATOM   3451  CA  LYS A 439      -3.676  18.377  28.346  1.00 25.75           C
ATOM   3452  C   LYS A 439      -4.672  18.082  27.239  1.00 25.01           C
ATOM   3453  O   LYS A 439      -4.405  18.336  26.059  1.00 22.62           O
ATOM   3454  CB  LYS A 439      -2.385  18.981  27.761  1.00 27.02           C
ATOM   3455  CG  LYS A 439      -2.044  20.364  28.297  1.00 26.58           C
ATOM   3456  CD  LYS A 439      -0.569  20.490  28.681  1.00 25.12           C
ATOM   3457  CE  LYS A 439      -0.261  21.909  29.205  1.00 26.40           C
ATOM   3458  NZ  LYS A 439       1.186  22.172  29.539  1.00 24.08           N1+
ATOM   3459  N   HIS A 440      -5.835  17.575  27.646  1.00 25.71           N
ATOM   3460  CA  HIS A 440      -6.781  16.952  26.733  1.00 25.96           C
ATOM   3461  C   HIS A 440      -8.119  16.494  27.332  1.00 26.52           C
ATOM   3462  O   HIS A 440      -8.967  16.005  26.602  1.00 27.11           O
ATOM   3463  CB  HIS A 440      -6.108  15.762  26.054  1.00 24.62           C
ATOM   3464  CG  HIS A 440      -7.055  14.877  25.323  1.00 20.53           C
ATOM   3465  CD2 HIS A 440      -7.449  13.606  25.559  1.00 20.18           C
ATOM   3466  ND1 HIS A 440      -7.787  15.313  24.247  1.00 21.98           N
ATOM   3467  CE1 HIS A 440      -8.575  14.341  23.830  1.00 22.57           C
ATOM   3468  NE2 HIS A 440      -8.386  13.291  24.607  1.00 20.14           N
ATOM   3469  N   PRO A 441      -8.400  16.840  28.595  1.00 28.45           N
ATOM   3470  CA  PRO A 441      -9.107  15.970  29.553  1.00 30.20           C
ATOM   3471  C   PRO A 441     -10.490  15.409  29.137  1.00 30.12           C
ATOM   3472  O   PRO A 441     -10.683  15.001  27.984  1.00 28.70           O
ATOM   3473  CB  PRO A 441      -9.202  16.838  30.811  1.00 32.36           C
ATOM   3474  CG  PRO A 441      -9.186  18.258  30.292  1.00 32.89           C
ATOM   3475  CD  PRO A 441      -8.282  18.229  29.090  1.00 30.04           C
ATOM   3476  N   GLU A 442     -11.410  15.285  30.101  1.00 29.30           N
ATOM   3477  CA  GLU A 442     -12.751  14.720  29.866  1.00 29.85           C
ATOM   3478  C   GLU A 442     -12.863  13.169  29.936  1.00 28.51           C
ATOM   3479  O   GLU A 442     -12.375  12.533  30.885  1.00 22.26           O
ATOM   3480  CB  GLU A 442     -13.324  15.201  28.507  1.00 29.58           C
ATOM   3481  CG  GLU A 442     -13.697  16.673  28.422  1.00 26.93           C
ATOM   3482  CD  GLU A 442     -12.757  17.475  27.541  1.00 25.31           C
ATOM   3483  OE1 GLU A 442     -11.572  17.123  27.442  1.00 20.53           O
ATOM   3484  OE2 GLU A 442     -13.192  18.505  26.974  1.00 28.11           O1-
ATOM   3485  N   ALA A 443     -13.822  12.669  29.147  1.00 27.70           N
ATOM   3486  CA  ALA A 443     -13.825  11.304  28.638  1.00 26.01           C
ATOM   3487  C   ALA A 443     -12.551  11.111  27.852  1.00 24.19           C
ATOM   3488  O   ALA A 443     -11.639  10.447  28.316  1.00 26.28           O
ATOM   3489  CB  ALA A 443     -15.016  11.089  27.708  1.00 26.77           C
ATOM   3490  N   LYS A 444     -12.411  11.892  26.788  1.00 22.05           N
ATOM   3491  CA  LYS A 444     -11.370  11.674  25.785  1.00 21.39           C
ATOM   3492  C   LYS A 444     -10.000  11.321  26.357  1.00 20.82           C
ATOM   3493  O   LYS A 444      -9.137  10.831  25.620  1.00 18.13           O
ATOM   3494  CB  LYS A 444     -11.248  12.911  24.894  1.00 22.35           C
ATOM   3495  CG  LYS A 444     -11.775  12.721  23.484  1.00 22.26           C
ATOM   3496  CD  LYS A 444     -13.306  12.807  23.457  1.00 20.62           C
ATOM   3497  CE  LYS A 444     -13.783  14.030  22.666  1.00 18.58           C
ATOM   3498  NZ  LYS A 444     -13.011  15.272  22.977  1.00 15.81           N1+
ATOM   3499  N   ARG A 445      -9.762  11.777  27.594  1.00 20.03           N
ATOM   3500  CA  ARG A 445      -8.614  11.392  28.429  1.00 18.49           C
ATOM   3501  C   ARG A 445      -8.397   9.892  28.555  1.00 19.53           C
ATOM   3502  O   ARG A 445      -7.514   9.343  27.914  1.00 21.22           O
```

```
ATOM   3503  CB  ARG A 445      -8.758  11.970  29.830  1.00 14.76           C
ATOM   3504  CG  ARG A 445      -7.643  11.580  30.764  1.00 10.97           C
ATOM   3505  CD  ARG A 445      -7.994  11.871  32.222  1.00 11.73           C
ATOM   3506  NE  ARG A 445      -8.114  13.288  32.629  1.00 13.19           N
ATOM   3507  CZ  ARG A 445      -7.458  14.336  32.122  1.00  9.58           C
ATOM   3508  NH1 ARG A 445      -6.945  14.323  30.899  1.00  7.80           N1+
ATOM   3509  NH2 ARG A 445      -7.497  15.482  32.775  1.00  6.22           N
ATOM   3510  N   MET A 446      -9.204   9.204  29.348  1.00 20.28           N
ATOM   3511  CA  MET A 446      -8.894   7.805  29.608  1.00 22.11           C
ATOM   3512  C   MET A 446      -8.796   6.892  28.368  1.00 21.20           C
ATOM   3513  O   MET A 446      -7.843   6.105  28.273  1.00 21.62           O
ATOM   3514  CB  MET A 446      -9.830   7.188  30.659  1.00 22.74           C
ATOM   3515  CG  MET A 446      -9.620   5.693  30.794  1.00 24.58           C
ATOM   3516  SD  MET A 446      -9.860   4.949  32.408  1.00 28.20           S
ATOM   3517  CE  MET A 446     -11.076   3.660  31.945  1.00 22.67           C
ATOM   3518  N   PRO A 447      -9.733   6.979  27.405  1.00 20.32           N
ATOM   3519  CA  PRO A 447      -9.574   6.042  26.294  1.00 22.10           C
ATOM   3520  C   PRO A 447      -8.133   6.079  25.772  1.00 23.45           C
ATOM   3521  O   PRO A 447      -7.541   5.014  25.563  1.00 25.75           O
ATOM   3522  CB  PRO A 447     -10.578   6.532  25.234  1.00 21.91           C
ATOM   3523  CG  PRO A 447     -11.561   7.311  25.970  1.00 20.33           C
ATOM   3524  CD  PRO A 447     -10.792   7.962  27.113  1.00 22.51           C
ATOM   3525  N   CYS A 448      -7.509   7.266  25.813  1.00 21.39           N
ATOM   3526  CA  CYS A 448      -6.156   7.436  25.279  1.00 19.05           C
ATOM   3527  C   CYS A 448      -5.183   6.767  26.222  1.00 18.85           C
ATOM   3528  O   CYS A 448      -4.317   6.025  25.775  1.00 21.92           O
ATOM   3529  CB  CYS A 448      -5.756   8.916  25.173  1.00 19.04           C
ATOM   3530  SG  CYS A 448      -4.591   9.295  26.505  1.00 20.87           S
ATOM   3531  N   ALA A 449      -5.386   6.971  27.527  1.00 17.31           N
ATOM   3532  CA  ALA A 449      -4.314   6.924  28.522  1.00 13.97           C
ATOM   3533  C   ALA A 449      -4.209   5.679  29.390  1.00 16.06           C
ATOM   3534  O   ALA A 449      -3.241   5.544  30.100  1.00 20.40           O
ATOM   3535  CB  ALA A 449      -4.376   8.142  29.409  1.00 10.09           C
ATOM   3536  N   GLU A 450      -5.188   4.780  29.374  1.00 17.26           N
ATOM   3537  CA  GLU A 450      -4.941   3.417  29.855  1.00 16.55           C
ATOM   3538  C   GLU A 450      -4.304   2.693  28.672  1.00 18.65           C
ATOM   3539  O   GLU A 450      -3.139   2.330  28.712  1.00 18.04           O
ATOM   3540  CB  GLU A 450      -6.245   2.725  30.241  1.00 17.28           C
ATOM   3541  CG  GLU A 450      -6.151   1.924  31.533  1.00 15.50           C
ATOM   3542  CD  GLU A 450      -7.098   0.716  31.585  1.00 18.82           C
ATOM   3543  OE1 GLU A 450      -8.213   0.768  31.027  1.00 17.57           O
ATOM   3544  OE2 GLU A 450      -6.726  -0.310  32.195  1.00 21.24           O1-
ATOM   3545  N   ASP A 451      -4.985   2.799  27.531  1.00 19.88           N
ATOM   3546  CA  ASP A 451      -4.495   2.392  26.213  1.00 18.66           C
ATOM   3547  C   ASP A 451      -3.055   2.711  25.815  1.00 18.58           C
ATOM   3548  O   ASP A 451      -2.341   1.810  25.397  1.00 20.61           O
ATOM   3549  CB  ASP A 451      -5.435   2.927  25.140  1.00 17.00           C
ATOM   3550  CG  ASP A 451      -6.675   2.088  25.009  1.00 19.38           C
ATOM   3551  OD1 ASP A 451      -6.556   0.869  25.262  1.00 16.86           O
ATOM   3552  OD2 ASP A 451      -7.762   2.634  24.690  1.00 19.70           O1-
ATOM   3553  N   TYR A 452      -2.658   3.981  25.827  1.00 16.13           N
ATOM   3554  CA  TYR A 452      -1.281   4.344  25.489  1.00 15.08           C
ATOM   3555  C   TYR A 452      -0.335   3.655  26.464  1.00 16.14           C
ATOM   3556  O   TYR A 452       0.481   2.837  26.070  1.00 17.95           O
ATOM   3557  CB  TYR A 452      -1.093   5.854  25.560  1.00 13.93           C
ATOM   3558  CG  TYR A 452       0.277   6.331  25.144  1.00 15.16           C
ATOM   3559  CD1 TYR A 452       1.381   6.207  25.995  1.00 13.94           C
ATOM   3560  CD2 TYR A 452       0.463   6.960  23.919  1.00 15.41           C
ATOM   3561  CE1 TYR A 452       2.634   6.698  25.624  1.00 13.09           C
ATOM   3562  CE2 TYR A 452       1.706   7.450  23.550  1.00 15.00           C
ATOM   3563  CZ  TYR A 452       2.779   7.318  24.401  1.00 12.67           C
ATOM   3564  OH  TYR A 452       3.973   7.861  24.029  1.00 11.49           O
ATOM   3565  N   LEU A 453      -0.498   3.931  27.751  1.00 16.23           N
ATOM   3566  CA  LEU A 453       0.196   3.200  28.794  1.00 13.79           C
ATOM   3567  C   LEU A 453       0.231   1.710  28.457  1.00 13.05           C
ATOM   3568  O   LEU A 453       1.301   1.125  28.413  1.00 15.56           O
ATOM   3569  CB  LEU A 453      -0.502   3.427  30.135  1.00 11.83           C
ATOM   3570  CG  LEU A 453       0.131   4.454  31.073  1.00 13.55           C
ATOM   3571  CD1 LEU A 453      -0.933   5.105  31.932  1.00 11.05           C
ATOM   3572  CD2 LEU A 453       1.178   3.782  31.944  1.00 10.99           C
ATOM   3573  N   SER A 454      -0.879   1.181  27.955  1.00 11.72           N
```

ATOM	3574	CA	SER	A	454	-0.980	-0.244	27.649	1.00	11.87	C
ATOM	3575	C	SER	A	454	-0.003	-0.699	26.557	1.00	12.13	C
ATOM	3576	O	SER	A	454	0.518	-1.825	26.598	1.00	8.69	O
ATOM	3577	CB	SER	A	454	-2.408	-0.605	27.220	1.00	14.00	C
ATOM	3578	OG	SER	A	454	-3.390	0.232	27.809	1.00	11.75	O
ATOM	3579	N	VAL	A	455	0.155	0.154	25.543	1.00	11.63	N
ATOM	3580	CA	VAL	A	455	1.107	-0.046	24.450	1.00	10.07	C
ATOM	3581	C	VAL	A	455	2.516	-0.105	25.015	1.00	11.00	C
ATOM	3582	O	VAL	A	455	3.288	-0.996	24.675	1.00	10.74	O
ATOM	3583	CB	VAL	A	455	1.075	1.128	23.425	1.00	11.18	C
ATOM	3584	CG1	VAL	A	455	2.127	0.913	22.352	1.00	10.50	C
ATOM	3585	CG2	VAL	A	455	-0.299	1.283	22.809	1.00	6.79	C
ATOM	3586	N	VAL	A	456	2.858	0.864	25.861	1.00	12.33	N
ATOM	3587	CA	VAL	A	456	4.227	1.008	26.324	1.00	15.30	C
ATOM	3588	C	VAL	A	456	4.669	-0.208	27.125	1.00	18.48	C
ATOM	3589	O	VAL	A	456	5.671	-0.858	26.797	1.00	21.87	O
ATOM	3590	CB	VAL	A	456	4.399	2.248	27.164	1.00	13.68	C
ATOM	3591	CG1	VAL	A	456	5.777	2.285	27.727	1.00	14.03	C
ATOM	3592	CG2	VAL	A	456	4.167	3.460	26.316	1.00	17.71	C
ATOM	3593	N	LEU	A	457	3.867	-0.585	28.110	1.00	18.16	N
ATOM	3594	CA	LEU	A	457	4.124	-1.806	28.837	1.00	17.51	C
ATOM	3595	C	LEU	A	457	4.406	-2.967	27.856	1.00	19.56	C
ATOM	3596	O	LEU	A	457	5.322	-3.758	28.077	1.00	23.05	O
ATOM	3597	CB	LEU	A	457	2.943	-2.096	29.782	1.00	14.10	C
ATOM	3598	CG	LEU	A	457	3.038	-1.457	31.183	1.00	10.11	C
ATOM	3599	CD1	LEU	A	457	3.844	-0.180	31.147	1.00	6.96	C
ATOM	3600	CD2	LEU	A	457	1.668	-1.183	31.723	1.00	6.91	C
ATOM	3601	N	ASN	A	458	3.820	-2.914	26.666	1.00	19.26	N
ATOM	3602	CA	ASN	A	458	4.044	-3.982	25.696	1.00	20.09	C
ATOM	3603	C	ASN	A	458	5.403	-3.848	25.054	1.00	20.20	C
ATOM	3604	O	ASN	A	458	6.144	-4.823	25.000	1.00	22.87	O
ATOM	3605	CB	ASN	A	458	2.967	-3.985	24.622	1.00	22.45	C
ATOM	3606	CG	ASN	A	458	3.165	-5.070	23.594	1.00	23.30	C
ATOM	3607	ND2	ASN	A	458	3.201	-4.665	22.348	1.00	27.57	N
ATOM	3608	OD1	ASN	A	458	3.122	-6.263	23.895	1.00	25.73	O
ATOM	3609	N	GLN	A	459	5.782	-2.627	24.683	1.00	18.71	N
ATOM	3610	CA	GLN	A	459	7.156	-2.341	24.284	1.00	16.79	C
ATOM	3611	C	GLN	A	459	8.104	-2.947	25.318	1.00	19.53	C
ATOM	3612	O	GLN	A	459	8.829	-3.897	25.000	1.00	20.10	O
ATOM	3613	CB	GLN	A	459	7.375	-0.850	24.212	1.00	16.06	C
ATOM	3614	CG	GLN	A	459	8.559	-0.451	23.411	1.00	19.00	C
ATOM	3615	CD	GLN	A	459	8.212	-0.265	21.970	1.00	18.86	C
ATOM	3616	NE2	GLN	A	459	7.618	0.865	21.647	1.00	18.61	N
ATOM	3617	OE1	GLN	A	459	8.431	-1.150	21.156	1.00	20.77	O
ATOM	3618	N	LEU	A	460	7.894	-2.591	26.591	1.00	19.18	N
ATOM	3619	CA	LEU	A	460	8.570	-3.248	27.725	1.00	17.82	C
ATOM	3620	C	LEU	A	460	8.632	-4.768	27.635	1.00	18.45	C
ATOM	3621	O	LEU	A	460	9.692	-5.338	27.877	1.00	21.46	O
ATOM	3622	CB	LEU	A	460	7.919	-2.860	29.063	1.00	15.63	C
ATOM	3623	CG	LEU	A	460	8.279	-3.617	30.355	1.00	14.04	C
ATOM	3624	CD1	LEU	A	460	9.785	-3.769	30.501	1.00	15.77	C
ATOM	3625	CD2	LEU	A	460	7.747	-2.867	31.555	1.00	12.12	C
ATOM	3626	N	CYS	A	461	7.513	-5.430	27.330	1.00	20.02	N
ATOM	3627	CA	CYS	A	461	7.470	-6.904	27.322	1.00	20.16	C
ATOM	3628	C	CYS	A	461	8.172	-7.527	26.121	1.00	21.18	C
ATOM	3629	O	CYS	A	461	8.929	-8.505	26.272	1.00	19.43	O
ATOM	3630	CB	CYS	A	461	6.028	-7.408	27.348	1.00	17.64	C
ATOM	3631	SG	CYS	A	461	5.081	-6.878	28.795	1.00	17.61	S
ATOM	3632	N	VAL	A	462	7.942	-6.920	24.950	1.00	21.46	N
ATOM	3633	CA	VAL	A	462	8.437	-7.401	23.660	1.00	22.27	C
ATOM	3634	C	VAL	A	462	9.942	-7.136	23.468	1.00	23.83	C
ATOM	3635	O	VAL	A	462	10.679	-7.973	22.924	1.00	22.80	O
ATOM	3636	CB	VAL	A	462	7.672	-6.738	22.517	1.00	20.37	C
ATOM	3637	CG1	VAL	A	462	6.179	-6.891	22.734	1.00	20.18	C
ATOM	3638	CG2	VAL	A	462	8.039	-5.304	22.420	1.00	17.82	C
ATOM	3639	N	LEU	A	463	10.358	-5.933	23.863	1.00	24.45	N
ATOM	3640	CA	LEU	A	463	11.749	-5.617	24.183	1.00	23.28	C
ATOM	3641	C	LEU	A	463	12.444	-6.794	24.843	1.00	23.88	C
ATOM	3642	O	LEU	A	463	13.285	-7.448	24.233	1.00	25.91	O
ATOM	3643	CB	LEU	A	463	11.800	-4.420	25.144	1.00	20.02	C
ATOM	3644	CG	LEU	A	463	12.855	-3.340	24.952	1.00	16.71	C

```
ATOM   3645  CD1 LEU A 463      13.665  -3.676  23.734  1.00 14.34           C
ATOM   3646  CD2 LEU A 463      12.196  -1.970  24.804  1.00 15.27           C
ATOM   3647  N   HIS A 464      12.115  -7.035  26.106  1.00 25.21           N
ATOM   3648  CA  HIS A 464      12.941  -7.887  26.951  1.00 27.10           C
ATOM   3649  C   HIS A 464      12.269  -9.220  27.310  1.00 25.73           C
ATOM   3650  O   HIS A 464      11.668  -9.364  28.380  1.00 26.56           O
ATOM   3651  CB  HIS A 464      13.392  -7.121  28.224  1.00 27.53           C
ATOM   3652  CG  HIS A 464      14.883  -7.158  28.474  1.00 26.94           C
ATOM   3653  CD2 HIS A 464      15.904  -7.678  27.738  1.00 21.05           C
ATOM   3654  ND1 HIS A 464      15.464  -6.665  29.625  1.00 24.27           N
ATOM   3655  CE1 HIS A 464      16.764  -6.892  29.600  1.00 20.67           C
ATOM   3656  NE2 HIS A 464      17.053  -7.502  28.464  1.00 17.48           N
ATOM   3657  N   GLU A 465      12.180 -10.082  26.305  1.00 23.46           N
ATOM   3658  CA  GLU A 465      12.280 -11.518  26.492  1.00 21.43           C
ATOM   3659  C   GLU A 465      13.764 -11.787  26.213  1.00 21.62           C
ATOM   3660  O   GLU A 465      14.509 -12.216  27.092  1.00 20.90           O
ATOM   3661  CB  GLU A 465      11.406 -12.258  25.458  1.00 23.62           C
ATOM   3662  CG  GLU A 465       9.867 -12.214  25.681  1.00 20.17           C
ATOM   3663  CD  GLU A 465       9.063 -12.794  24.503  1.00 18.19           C
ATOM   3664  OE1 GLU A 465       8.978 -12.111  23.465  1.00 15.65           O
ATOM   3665  OE2 GLU A 465       8.455 -13.888  24.626  1.00 15.39           O1-
ATOM   3666  N   LYS A 466      14.199 -11.367  25.024  1.00 22.56           N
ATOM   3667  CA  LYS A 466      15.611 -11.327  24.604  1.00 24.39           C
ATOM   3668  C   LYS A 466      16.664 -11.640  25.685  1.00 21.61           C
ATOM   3669  O   LYS A 466      17.404 -12.606  25.542  1.00 22.78           O
ATOM   3670  CB  LYS A 466      15.899  -9.967  23.918  1.00 26.20           C
ATOM   3671  CG  LYS A 466      17.381  -9.571  23.804  1.00 27.48           C
ATOM   3672  CD  LYS A 466      17.668  -8.239  24.520  1.00 26.14           C
ATOM   3673  CE  LYS A 466      19.123  -7.796  24.343  1.00 23.78           C
ATOM   3674  NZ  LYS A 466      20.084  -8.644  25.115  1.00 20.55           N1+
ATOM   3675  N   THR A 467      16.826 -10.776  26.681  1.00 19.96           N
ATOM   3676  CA  THR A 467      17.514 -11.183  27.914  1.00 22.51           C
ATOM   3677  C   THR A 467      16.397 -11.294  28.923  1.00 25.70           C
ATOM   3678  O   THR A 467      15.585 -10.359  29.041  1.00 29.10           O
ATOM   3679  CB  THR A 467      18.489 -10.112  28.487  1.00 16.74           C
ATOM   3680  CG2 THR A 467      19.829 -10.712  28.809  1.00 14.95           C
ATOM   3681  OG1 THR A 467      18.653  -9.056  27.550  1.00 14.94           O
ATOM   3682  N   PRO A 468      16.405 -12.344  29.752  1.00 25.15           N
ATOM   3683  CA  PRO A 468      15.248 -12.352  30.645  1.00 26.23           C
ATOM   3684  C   PRO A 468      15.654 -11.886  32.035  1.00 25.34           C
ATOM   3685  O   PRO A 468      16.667 -12.324  32.567  1.00 24.12           O
ATOM   3686  CB  PRO A 468      14.786 -13.809  30.609  1.00 27.92           C
ATOM   3687  CG  PRO A 468      15.621 -14.461  29.496  1.00 26.78           C
ATOM   3688  CD  PRO A 468      16.904 -13.702  29.528  1.00 23.56           C
ATOM   3689  N   VAL A 469      14.900 -10.945  32.589  1.00 26.09           N
ATOM   3690  CA  VAL A 469      15.269 -10.322  33.863  1.00 26.57           C
ATOM   3691  C   VAL A 469      14.259 -10.507  35.043  1.00 26.29           C
ATOM   3692  O   VAL A 469      14.272 -11.537  35.720  1.00 25.05           O
ATOM   3693  CB  VAL A 469      15.609  -8.808  33.643  1.00 25.96           C
ATOM   3694  CG1 VAL A 469      17.067  -8.663  33.262  1.00 23.63           C
ATOM   3695  CG2 VAL A 469      14.723  -8.211  32.541  1.00 22.41           C
ATOM   3696  N   SER A 470      13.398  -9.520  35.283  1.00 24.94           N
ATOM   3697  CA  SER A 470      12.584  -9.486  36.492  1.00 25.10           C
ATOM   3698  C   SER A 470      11.436 -10.470  36.378  1.00 26.22           C
ATOM   3699  O   SER A 470      10.438 -10.196  35.697  1.00 27.50           O
ATOM   3700  CB  SER A 470      12.021  -8.070  36.744  1.00 24.74           C
ATOM   3701  OG  SER A 470      11.640  -7.884  38.110  1.00 22.77           O
ATOM   3702  N   ASP A 471      11.516 -11.553  37.145  1.00 25.67           N
ATOM   3703  CA  ASP A 471      10.504 -12.596  37.091  1.00 25.01           C
ATOM   3704  C   ASP A 471       9.126 -11.976  37.245  1.00 26.17           C
ATOM   3705  O   ASP A 471       8.146 -12.438  36.640  1.00 27.11           O
ATOM   3706  CB  ASP A 471      10.765 -13.620  38.175  1.00 23.33           C
ATOM   3707  CG  ASP A 471      12.078 -14.320  37.978  1.00 25.27           C
ATOM   3708  OD1 ASP A 471      13.125 -13.757  38.359  1.00 29.79           O
ATOM   3709  OD2 ASP A 471      12.086 -15.384  37.335  1.00 29.40           O1-
ATOM   3710  N   ARG A 472       9.108 -10.793  37.853  1.00 25.75           N
ATOM   3711  CA  ARG A 472       7.884 -10.014  37.949  1.00 26.02           C
ATOM   3712  C   ARG A 472       7.420  -9.534  36.583  1.00 26.00           C
ATOM   3713  O   ARG A 472       6.279  -9.785  36.182  1.00 26.81           O
ATOM   3714  CB  ARG A 472       8.098  -8.836  38.897  1.00 25.19           C
ATOM   3715  CG  ARG A 472       8.527  -9.296  40.283  1.00 24.67           C
```

ATOM	3716	CD	ARG	A	472	7.413	-9.145	41.277	1.00 20.81	C
ATOM	3717	NE	ARG	A	472	7.918	-8.558	42.510	1.00 15.73	N
ATOM	3718	CZ	ARG	A	472	7.177	-7.901	43.390	1.00 15.03	C
ATOM	3719	NH1	ARG	A	472	5.946	-7.542	43.087	1.00 15.14	N1+
ATOM	3720	NH2	ARG	A	472	7.720	-7.494	44.523	1.00 16.85	N
ATOM	3721	N	VAL	A	473	8.323	-8.888	35.853	1.00 24.83	N
ATOM	3722	CA	VAL	A	473	8.029	-8.415	34.512	1.00 23.45	C
ATOM	3723	C	VAL	A	473	7.641	-9.624	33.669	1.00 22.85	C
ATOM	3724	O	VAL	A	473	6.743	-9.554	32.840	1.00 22.94	O
ATOM	3725	CB	VAL	A	473	9.259	-7.717	33.873	1.00 25.33	C
ATOM	3726	CG1	VAL	A	473	8.869	-7.093	32.530	1.00 23.20	C
ATOM	3727	CG2	VAL	A	473	9.845	-6.670	34.828	1.00 19.55	C
ATOM	3728	N	THR	A	474	8.260	-10.755	33.947	1.00 21.09	N
ATOM	3729	CA	THR	A	474	7.847	-11.976	33.298	1.00 24.95	C
ATOM	3730	C	THR	A	474	6.372	-12.199	33.594	1.00 25.61	C
ATOM	3731	O	THR	A	474	5.565	-12.398	32.675	1.00 27.28	O
ATOM	3732	CB	THR	A	474	8.647	-13.169	33.829	1.00 27.15	C
ATOM	3733	CG2	THR	A	474	8.638	-14.329	32.811	1.00 28.51	C
ATOM	3734	OG1	THR	A	474	9.997	-12.748	34.084	1.00 29.10	O
ATOM	3735	N	LYS	A	475	6.000	-12.011	34.856	1.00 26.00	N
ATOM	3736	CA	LYS	A	475	4.657	-12.344	35.320	1.00 24.30	C
ATOM	3737	C	LYS	A	475	3.533	-11.428	34.798	1.00 21.93	C
ATOM	3738	O	LYS	A	475	2.528	-11.951	34.305	1.00 20.33	O
ATOM	3739	CB	LYS	A	475	4.636	-12.427	36.858	1.00 25.66	C
ATOM	3740	CG	LYS	A	475	4.745	-13.872	37.436	1.00 27.40	C
ATOM	3741	CD	LYS	A	475	6.131	-14.541	37.206	1.00 29.53	C
ATOM	3742	CE	LYS	A	475	6.706	-15.275	38.468	1.00 27.50	C
ATOM	3743	NZ	LYS	A	475	7.235	-14.367	39.560	1.00 20.78	N1+
ATOM	3744	N	CYS	A	476	3.730	-10.098	34.805	1.00 18.81	N
ATOM	3745	CA	CYS	A	476	2.672	-9.129	34.411	1.00 14.74	C
ATOM	3746	C	CYS	A	476	2.446	-9.020	32.891	1.00 14.14	C
ATOM	3747	O	CYS	A	476	1.340	-8.728	32.413	1.00 10.01	O
ATOM	3748	CB	CYS	A	476	2.973	-7.735	34.969	1.00 14.71	C
ATOM	3749	SG	CYS	A	476	2.665	-7.476	36.756	1.00 17.94	S
ATOM	3750	N	CYS	A	477	3.502	-9.291	32.135	1.00 14.05	N
ATOM	3751	CA	CYS	A	477	3.383	-9.530	30.702	1.00 12.13	C
ATOM	3752	C	CYS	A	477	2.553	-10.798	30.380	1.00 11.06	C
ATOM	3753	O	CYS	A	477	1.395	-10.686	29.966	1.00 6.49	O
ATOM	3754	CB	CYS	A	477	4.794	-9.588	30.090	1.00 13.42	C
ATOM	3755	SG	CYS	A	477	5.744	-8.034	30.324	1.00 14.64	S
ATOM	3756	N	THR	A	478	3.070	-11.969	30.755	1.00 11.58	N
ATOM	3757	CA	THR	A	478	2.425	-13.263	30.481	1.00 14.89	C
ATOM	3758	C	THR	A	478	1.027	-13.474	31.033	1.00 15.80	C
ATOM	3759	O	THR	A	478	0.194	-14.100	30.369	1.00 16.44	O
ATOM	3760	CB	THR	A	478	3.241	-14.447	31.035	1.00 18.24	C
ATOM	3761	CG2	THR	A	478	3.479	-14.300	32.546	1.00 16.23	C
ATOM	3762	OG1	THR	A	478	2.514	-15.661	30.807	1.00 19.21	O
ATOM	3763	N	GLU	A	479	0.903	-13.261	32.343	1.00 15.82	N
ATOM	3764	CA	GLU	A	479	-0.286	-13.641	33.070	1.00 18.03	C
ATOM	3765	C	GLU	A	479	-1.524	-12.993	32.455	1.00 19.17	C
ATOM	3766	O	GLU	A	479	-2.474	-13.703	32.167	1.00 20.64	O
ATOM	3767	CB	GLU	A	479	-0.144	-13.319	34.572	1.00 20.48	C
ATOM	3768	CG	GLU	A	479	0.926	-14.185	35.346	1.00 25.02	C
ATOM	3769	CD	GLU	A	479	0.428	-15.556	35.910	1.00 26.36	C
ATOM	3770	OE1	GLU	A	479	-0.438	-15.569	36.819	1.00 27.61	O
ATOM	3771	OE2	GLU	A	479	1.014	-16.614	35.558	1.00 23.89	O1-
ATOM	3772	N	SER	A	480	-1.476	-11.697	32.129	1.00 21.19	N
ATOM	3773	CA	SER	A	480	-2.479	-11.102	31.229	1.00 23.00	C
ATOM	3774	C	SER	A	480	-2.032	-9.944	30.320	1.00 22.37	C
ATOM	3775	O	SER	A	480	-1.343	-9.022	30.768	1.00 23.67	O
ATOM	3776	CB	SER	A	480	-3.703	-10.633	32.019	1.00 25.73	C
ATOM	3777	OG	SER	A	480	-4.717	-10.096	31.159	1.00 24.90	O
ATOM	3778	N	LEU	A	481	-2.642	-9.900	29.133	1.00 18.32	N
ATOM	3779	CA	LEU	A	481	-2.721	-8.692	28.306	1.00 17.04	C
ATOM	3780	C	LEU	A	481	-3.581	-7.681	29.029	1.00 16.84	C
ATOM	3781	O	LEU	A	481	-3.162	-6.563	29.292	1.00 16.88	O
ATOM	3782	CB	LEU	A	481	-3.392	-8.992	26.950	1.00 12.89	C
ATOM	3783	CG	LEU	A	481	-3.438	-7.943	25.831	1.00 8.60	C
ATOM	3784	CD1	LEU	A	481	-2.107	-7.944	25.173	1.00 11.20	C
ATOM	3785	CD2	LEU	A	481	-4.510	-8.235	24.771	1.00 2.00	C
ATOM	3786	N	VAL	A	482	-4.791	-8.109	29.364	1.00 18.70	N

ATOM	3787	CA	VAL	A	482	-5.873	-7.205	29.741	1.00	17.38	C
ATOM	3788	C	VAL	A	482	-5.656	-6.631	31.146	1.00	15.41	C
ATOM	3789	O	VAL	A	482	-6.034	-5.490	31.420	1.00	12.39	O
ATOM	3790	CB	VAL	A	482	-7.235	-7.944	29.665	1.00	19.15	C
ATOM	3791	CG1	VAL	A	482	-8.371	-6.944	29.602	1.00	16.59	C
ATOM	3792	CG2	VAL	A	482	-7.262	-8.910	28.452	1.00	14.21	C
ATOM	3793	N	ASN	A	483	-4.887	-7.349	31.962	1.00	15.51	N
ATOM	3794	CA	ASN	A	483	-4.543	-6.871	33.313	1.00	19.45	C
ATOM	3795	C	ASN	A	483	-3.117	-6.282	33.448	1.00	18.86	C
ATOM	3796	O	ASN	A	483	-2.541	-6.286	34.527	1.00	18.09	O
ATOM	3797	CB	ASN	A	483	-4.744	-7.999	34.349	1.00	20.35	C
ATOM	3798	CG	ASN	A	483	-6.101	-8.689	34.212	1.00	23.29	C
ATOM	3799	ND2	ASN	A	483	-7.134	-8.065	34.770	1.00	23.14	N
ATOM	3800	OD1	ASN	A	483	-6.232	-9.728	33.544	1.00	20.79	O
ATOM	3801	N	ARG	A	484	-2.627	-5.632	32.400	1.00	19.94	N
ATOM	3802	CA	ARG	A	484	-1.235	-5.195	32.348	1.00	18.62	C
ATOM	3803	C	ARG	A	484	-1.091	-4.010	33.255	1.00	16.95	C
ATOM	3804	O	ARG	A	484	-0.397	-4.077	34.247	1.00	15.09	O
ATOM	3805	CB	ARG	A	484	-0.844	-4.773	30.920	1.00	22.74	C
ATOM	3806	CG	ARG	A	484	0.231	-5.615	30.212	1.00	18.99	C
ATOM	3807	CD	ARG	A	484	0.209	-5.384	28.687	1.00	16.43	C
ATOM	3808	NE	ARG	A	484	1.293	-6.102	28.008	1.00	17.41	N
ATOM	3809	CZ	ARG	A	484	1.157	-7.231	27.313	1.00	16.27	C
ATOM	3810	NH1	ARG	A	484	-0.025	-7.784	27.149	1.00	17.18	N1+
ATOM	3811	NH2	ARG	A	484	2.216	-7.812	26.767	1.00	20.22	N
ATOM	3812	N	ARG	A	485	-1.868	-2.973	32.992	1.00	17.31	N
ATOM	3813	CA	ARG	A	485	-1.641	-1.679	33.626	1.00	19.94	C
ATOM	3814	C	ARG	A	485	-2.259	-1.408	35.051	1.00	22.79	C
ATOM	3815	O	ARG	A	485	-1.991	-0.351	35.670	1.00	22.25	O
ATOM	3816	CB	ARG	A	485	-2.043	-0.583	32.637	1.00	16.29	C
ATOM	3817	CG	ARG	A	485	-1.603	0.799	33.039	1.00	16.70	C
ATOM	3818	CD	ARG	A	485	-2.801	1.708	33.276	1.00	15.57	C
ATOM	3819	NE	ARG	A	485	-3.930	1.020	33.887	1.00	12.17	N
ATOM	3820	CZ	ARG	A	485	-4.215	1.091	35.173	1.00	11.81	C
ATOM	3821	NH1	ARG	A	485	-3.411	1.751	35.978	1.00	15.13	N1+
ATOM	3822	NH2	ARG	A	485	-5.192	0.371	35.680	1.00	12.58	N
ATOM	3823	N	PRO	A	486	-3.067	-2.350	35.598	1.00	22.59	N
ATOM	3824	CA	PRO	A	486	-3.024	-2.488	37.067	1.00	23.53	C
ATOM	3825	C	PRO	A	486	-2.092	-3.558	37.639	1.00	21.48	C
ATOM	3826	O	PRO	A	486	-2.263	-3.997	38.779	1.00	18.78	O
ATOM	3827	CB	PRO	A	486	-4.489	-2.756	37.448	1.00	23.69	C
ATOM	3828	CG	PRO	A	486	-5.101	-3.264	36.224	1.00	19.85	C
ATOM	3829	CD	PRO	A	486	-4.446	-2.530	35.110	1.00	22.51	C
ATOM	3830	N	CYS	A	487	-1.118	-3.982	36.846	1.00	19.53	N
ATOM	3831	CA	CYS	A	487	-0.170	-4.986	37.304	1.00	19.20	C
ATOM	3832	C	CYS	A	487	1.135	-4.343	37.720	1.00	20.85	C
ATOM	3833	O	CYS	A	487	1.389	-4.179	38.908	1.00	24.17	O
ATOM	3834	CB	CYS	A	487	0.102	-6.004	36.223	1.00	15.12	C
ATOM	3835	SG	CYS	A	487	0.659	-7.543	36.947	1.00	12.46	S
ATOM	3836	N	PHE	A	488	1.882	-3.834	36.745	1.00	21.06	N
ATOM	3837	CA	PHE	A	488	3.057	-3.030	37.018	1.00	16.19	C
ATOM	3838	C	PHE	A	488	2.761	-2.078	38.133	1.00	16.14	C
ATOM	3839	O	PHE	A	488	3.334	-2.231	39.193	1.00	16.21	O
ATOM	3840	CB	PHE	A	488	3.487	-2.282	35.767	1.00	15.68	C
ATOM	3841	CG	PHE	A	488	4.434	-3.072	34.917	1.00	18.17	C
ATOM	3842	CD1	PHE	A	488	3.953	-4.049	34.066	1.00	14.32	C
ATOM	3843	CD2	PHE	A	488	5.800	-3.027	35.173	1.00	15.88	C
ATOM	3844	CE1	PHE	A	488	4.799	-4.961	33.530	1.00	13.05	C
ATOM	3845	CE2	PHE	A	488	6.638	-3.937	34.632	1.00	7.89	C
ATOM	3846	CZ	PHE	A	488	6.142	-4.907	33.821	1.00	11.30	C
ATOM	3847	N	SER	A	489	1.648	-1.356	37.989	1.00	16.65	N
ATOM	3848	CA	SER	A	489	1.124	-0.449	39.020	1.00	14.57	C
ATOM	3849	C	SER	A	489	1.020	-1.124	40.400	1.00	14.49	C
ATOM	3850	O	SER	A	489	1.604	-0.644	41.368	1.00	14.95	O
ATOM	3851	CB	SER	A	489	-0.261	0.084	38.606	1.00	13.40	C
ATOM	3852	OG	SER	A	489	-0.220	0.832	37.402	1.00	9.27	O
ATOM	3853	N	ALA	A	490	0.329	-2.264	40.471	1.00	14.36	N
ATOM	3854	CA	ALA	A	490	0.018	-2.922	41.746	1.00	14.42	C
ATOM	3855	C	ALA	A	490	1.174	-3.786	42.177	1.00	15.92	C
ATOM	3856	O	ALA	A	490	1.016	-4.996	42.395	1.00	16.03	O
ATOM	3857	CB	ALA	A	490	-1.233	-3.764	41.628	1.00	14.31	C

ATOM	3858	N	LEU	A	491	2.275	-3.111	42.483	1.00	16.50	N
ATOM	3859	CA	LEU	A	491	3.582	-3.730	42.630	1.00	17.68	C
ATOM	3860	C	LEU	A	491	4.264	-2.722	43.515	1.00	18.16	C
ATOM	3861	O	LEU	A	491	4.181	-1.532	43.235	1.00	19.45	O
ATOM	3862	CB	LEU	A	491	4.274	-3.755	41.260	1.00	18.42	C
ATOM	3863	CG	LEU	A	491	5.176	-4.844	40.673	1.00	19.23	C
ATOM	3864	CD1	LEU	A	491	4.410	-6.103	40.303	1.00	19.20	C
ATOM	3865	CD2	LEU	A	491	5.816	-4.257	39.436	1.00	21.21	C
ATOM	3866	N	GLU	A	492	4.742	-3.138	44.677	1.00	17.64	N
ATOM	3867	CA	GLU	A	492	5.441	-2.176	45.526	1.00	20.34	C
ATOM	3868	C	GLU	A	492	6.920	-2.316	45.191	1.00	20.14	C
ATOM	3869	O	GLU	A	492	7.255	-2.927	44.186	1.00	18.77	O
ATOM	3870	CB	GLU	A	492	5.143	-2.414	47.033	1.00	20.09	C
ATOM	3871	CG	GLU	A	492	3.620	-2.630	47.374	1.00	24.17	C
ATOM	3872	CD	GLU	A	492	2.836	-1.411	47.983	1.00	25.80	C
ATOM	3873	OE1	GLU	A	492	3.305	-0.784	48.959	1.00	27.22	O
ATOM	3874	OE2	GLU	A	492	1.648	-1.216	47.614	1.00	24.48	O1-
ATOM	3875	N	VAL	A	493	7.786	-1.623	45.924	1.00	21.53	N
ATOM	3876	CA	VAL	A	493	9.231	-1.795	45.779	1.00	20.93	C
ATOM	3877	C	VAL	A	493	9.635	-3.247	46.044	1.00	22.35	C
ATOM	3878	O	VAL	A	493	9.525	-3.722	47.170	1.00	24.31	O
ATOM	3879	CB	VAL	A	493	9.982	-0.910	46.767	1.00	18.51	C
ATOM	3880	CG1	VAL	A	493	11.487	-1.196	46.692	1.00	19.04	C
ATOM	3881	CG2	VAL	A	493	9.666	0.552	46.492	1.00	17.43	C
ATOM	3882	N	ASP	A	494	10.093	-3.951	45.016	1.00	24.29	N
ATOM	3883	CA	ASP	A	494	10.404	-5.382	45.148	1.00	25.27	C
ATOM	3884	C	ASP	A	494	11.691	-5.486	45.946	1.00	23.84	C
ATOM	3885	O	ASP	A	494	12.763	-5.666	45.375	1.00	22.03	O
ATOM	3886	CB	ASP	A	494	10.603	-6.067	43.764	1.00	25.69	C
ATOM	3887	CG	ASP	A	494	9.375	-5.952	42.826	1.00	21.60	C
ATOM	3888	OD1	ASP	A	494	8.405	-5.219	43.111	1.00	20.70	O
ATOM	3889	OD2	ASP	A	494	9.391	-6.593	41.768	1.00	16.55	O1-
ATOM	3890	N	GLU	A	495	11.558	-5.460	47.270	1.00	24.75	N
ATOM	3891	CA	GLU	A	495	12.703	-5.461	48.188	1.00	25.22	C
ATOM	3892	C	GLU	A	495	13.578	-6.701	48.020	1.00	25.09	C
ATOM	3893	O	GLU	A	495	14.671	-6.793	48.586	1.00	23.87	O
ATOM	3894	CB	GLU	A	495	12.229	-5.334	49.644	1.00	25.31	C
ATOM	3895	CG	GLU	A	495	11.358	-4.089	49.951	1.00	22.81	C
ATOM	3896	CD	GLU	A	495	9.976	-4.443	50.509	1.00	23.79	C
ATOM	3897	OE1	GLU	A	495	9.639	-5.650	50.564	1.00	20.96	O
ATOM	3898	OE2	GLU	A	495	9.223	-3.511	50.885	1.00	22.17	O1-
ATOM	3899	N	THR	A	496	13.107	-7.595	47.149	1.00	26.34	N
ATOM	3900	CA	THR	A	496	13.866	-8.694	46.535	1.00	23.22	C
ATOM	3901	C	THR	A	496	15.335	-8.416	46.231	1.00	22.62	C
ATOM	3902	O	THR	A	496	16.150	-9.331	46.277	1.00	22.94	O
ATOM	3903	CB	THR	A	496	13.187	-9.146	45.199	1.00	21.63	C
ATOM	3904	CG2	THR	A	496	12.732	-10.581	45.296	1.00	21.18	C
ATOM	3905	OG1	THR	A	496	12.060	-8.302	44.898	1.00	16.30	O
ATOM	3906	N	TYR	A	497	15.661	-7.150	45.975	1.00	22.20	N
ATOM	3907	CA	TYR	A	497	16.844	-6.768	45.215	1.00	23.14	C
ATOM	3908	C	TYR	A	497	18.148	-7.505	45.559	1.00	25.54	C
ATOM	3909	O	TYR	A	497	18.420	-8.588	45.015	1.00	24.48	O
ATOM	3910	CB	TYR	A	497	17.055	-5.256	45.330	1.00	23.83	C
ATOM	3911	CG	TYR	A	497	17.784	-4.607	44.155	1.00	25.67	C
ATOM	3912	CD1	TYR	A	497	17.903	-5.248	42.939	1.00	26.17	C
ATOM	3913	CD2	TYR	A	497	18.419	-3.381	44.302	1.00	24.70	C
ATOM	3914	CE1	TYR	A	497	18.656	-4.699	41.917	1.00	27.56	C
ATOM	3915	CE2	TYR	A	497	19.163	-2.831	43.293	1.00	24.84	C
ATOM	3916	CZ	TYR	A	497	19.298	-3.496	42.103	1.00	27.12	C
ATOM	3917	OH	TYR	A	497	20.187	-3.028	41.144	1.00	25.38	O
ATOM	3918	N	VAL	A	498	18.915	-6.947	46.504	1.00	26.48	N
ATOM	3919	CA	VAL	A	498	20.386	-7.050	46.515	1.00	25.76	C
ATOM	3920	C	VAL	A	498	20.815	-6.909	45.059	1.00	27.52	C
ATOM	3921	O	VAL	A	498	20.314	-6.011	44.377	1.00	26.89	O
ATOM	3922	CB	VAL	A	498	20.934	-8.371	47.162	1.00	25.41	C
ATOM	3923	CG1	VAL	A	498	22.373	-8.146	47.637	1.00	23.31	C
ATOM	3924	CG2	VAL	A	498	20.075	-8.802	48.361	1.00	25.94	C
ATOM	3925	N	PRO	A	499	21.622	-7.846	44.516	1.00	30.00	N
ATOM	3926	CA	PRO	A	499	22.892	-7.469	43.868	1.00	29.18	C
ATOM	3927	C	PRO	A	499	23.366	-6.065	44.192	1.00	29.84	C
ATOM	3928	O	PRO	A	499	24.553	-5.856	44.419	1.00	32.19	O

ATOM	3929	CB	PRO	A	499	22.589	-7.640	42.383	1.00	29.25	C
ATOM	3930	CG	PRO	A	499	21.521	-8.787	42.353	1.00	31.67	C
ATOM	3931	CD	PRO	A	499	21.046	-9.013	43.821	1.00	29.92	C
ATOM	3932	N	LYS	A	500	22.480	-5.092	44.014	1.00	29.43	N
ATOM	3933	CA	LYS	A	500	22.604	-3.779	44.641	1.00	28.77	C
ATOM	3934	C	LYS	A	500	23.964	-3.178	44.341	1.00	29.16	C
ATOM	3935	O	LYS	A	500	24.597	-2.585	45.213	1.00	31.34	O
ATOM	3936	CB	LYS	A	500	22.376	-3.880	46.153	1.00	25.37	C
ATOM	3937	CG	LYS	A	500	21.848	-2.597	46.770	1.00	23.83	C
ATOM	3938	CD	LYS	A	500	21.013	-2.876	48.001	1.00	24.99	C
ATOM	3939	CE	LYS	A	500	19.991	-3.991	47.712	1.00	29.69	C
ATOM	3940	NZ	LYS	A	500	18.606	-3.820	48.316	1.00	30.96	N1+
ATOM	3941	N	GLU	A	501	24.456	-3.494	43.144	1.00	28.36	N
ATOM	3942	CA	GLU	A	501	25.733	-3.020	42.626	1.00	27.10	C
ATOM	3943	C	GLU	A	501	26.219	-1.775	43.365	1.00	23.87	C
ATOM	3944	O	GLU	A	501	25.440	-0.854	43.611	1.00	24.20	O
ATOM	3945	CB	GLU	A	501	25.583	-2.722	41.120	1.00	28.81	C
ATOM	3946	CG	GLU	A	501	26.853	-2.919	40.279	1.00	27.95	C
ATOM	3947	CD	GLU	A	501	26.979	-4.325	39.717	1.00	26.77	C
ATOM	3948	OE1	GLU	A	501	25.944	-4.926	39.354	1.00	26.88	O
ATOM	3949	OE2	GLU	A	501	28.114	-4.842	39.661	1.00	27.69	O1-
ATOM	3950	N	PHE	A	502	27.466	-1.790	43.799	1.00	19.52	N
ATOM	3951	CA	PHE	A	502	28.068	-0.583	44.320	1.00	21.84	C
ATOM	3952	C	PHE	A	502	29.008	0.086	43.277	1.00	22.82	C
ATOM	3953	O	PHE	A	502	29.712	-0.598	42.551	1.00	24.52	O
ATOM	3954	CB	PHE	A	502	28.827	-0.921	45.617	1.00	22.57	C
ATOM	3955	CG	PHE	A	502	28.007	-0.747	46.897	1.00	24.13	C
ATOM	3956	CD1	PHE	A	502	26.662	-0.372	46.856	1.00	20.81	C
ATOM	3957	CD2	PHE	A	502	28.627	-0.839	48.149	1.00	22.46	C
ATOM	3958	CE1	PHE	A	502	25.976	-0.085	48.030	1.00	19.06	C
ATOM	3959	CE2	PHE	A	502	27.937	-0.544	49.329	1.00	18.64	C
ATOM	3960	CZ	PHE	A	502	26.621	-0.165	49.266	1.00	19.92	C
ATOM	3961	N	ASN	A	503	28.876	1.392	43.069	1.00	22.88	N
ATOM	3962	CA	ASN	A	503	30.017	2.214	42.668	1.00	24.10	C
ATOM	3963	C	ASN	A	503	30.509	2.157	41.188	1.00	25.87	C
ATOM	3964	O	ASN	A	503	31.690	2.401	40.905	1.00	24.39	O
ATOM	3965	CB	ASN	A	503	31.167	1.906	43.630	1.00	24.77	C
ATOM	3966	CG	ASN	A	503	30.765	2.064	45.094	1.00	25.30	C
ATOM	3967	ND2	ASN	A	503	31.270	1.181	45.961	1.00	25.44	N
ATOM	3968	OD1	ASN	A	503	30.024	2.978	45.442	1.00	26.75	O
ATOM	3969	N	ALA	A	504	29.564	2.196	40.254	1.00	28.88	N
ATOM	3970	CA	ALA	A	504	29.804	1.734	38.874	1.00	31.13	C
ATOM	3971	C	ALA	A	504	30.300	2.776	37.823	1.00	31.37	C
ATOM	3972	O	ALA	A	504	29.549	3.108	36.885	1.00	29.77	O
ATOM	3973	CB	ALA	A	504	28.522	1.003	38.351	1.00	29.20	C
ATOM	3974	N	GLU	A	505	31.615	3.064	37.847	1.00	31.12	N
ATOM	3975	CA	GLU	A	505	32.305	4.005	36.923	1.00	30.29	C
ATOM	3976	C	GLU	A	505	32.672	3.490	35.500	1.00	30.54	C
ATOM	3977	O	GLU	A	505	33.795	3.603	35.040	1.00	30.90	O
ATOM	3978	CB	GLU	A	505	33.555	4.579	37.623	1.00	29.84	C
ATOM	3979	CG	GLU	A	505	34.522	5.484	36.794	1.00	30.64	C
ATOM	3980	CD	GLU	A	505	33.845	6.636	36.067	1.00	30.76	C
ATOM	3981	OE1	GLU	A	505	32.604	6.713	36.126	1.00	33.58	O
ATOM	3982	OE2	GLU	A	505	34.546	7.445	35.405	1.00	27.70	O1-
ATOM	3983	N	THR	A	506	31.770	2.737	34.900	1.00	32.72	N
ATOM	3984	CA	THR	A	506	31.498	2.842	33.477	1.00	30.71	C
ATOM	3985	C	THR	A	506	30.144	3.518	33.584	1.00	30.02	C
ATOM	3986	O	THR	A	506	29.098	2.881	33.485	1.00	30.52	O
ATOM	3987	CB	THR	A	506	31.347	1.480	32.857	1.00	31.87	C
ATOM	3988	CG2	THR	A	506	30.724	1.603	31.495	1.00	32.08	C
ATOM	3989	OG1	THR	A	506	32.644	0.859	32.776	1.00	33.97	O
ATOM	3990	N	PHE	A	507	30.249	4.657	34.251	1.00	28.98	N
ATOM	3991	CA	PHE	A	507	29.169	5.342	34.917	1.00	26.92	C
ATOM	3992	C	PHE	A	507	29.075	6.625	34.106	1.00	25.90	C
ATOM	3993	O	PHE	A	507	28.730	6.586	32.917	1.00	22.64	O
ATOM	3994	CB	PHE	A	507	29.648	5.661	36.330	1.00	27.01	C
ATOM	3995	CG	PHE	A	507	28.600	6.192	37.248	1.00	28.40	C
ATOM	3996	CD1	PHE	A	507	27.527	6.923	36.778	1.00	26.26	C
ATOM	3997	CD2	PHE	A	507	28.789	6.093	38.622	1.00	28.34	C
ATOM	3998	CE1	PHE	A	507	26.673	7.551	37.666	1.00	28.02	C
ATOM	3999	CE2	PHE	A	507	27.934	6.718	39.511	1.00	26.64	C

ATOM	4000	CZ	PHE	A	507	26.882	7.446	39.035	1.00	26.34	C
ATOM	4001	N	THR	A	508	29.795	7.615	34.624	1.00	23.79	N
ATOM	4002	CA	THR	A	508	29.605	9.007	34.270	1.00	25.58	C
ATOM	4003	C	THR	A	508	30.579	9.372	33.153	1.00	24.27	C
ATOM	4004	O	THR	A	508	31.651	9.945	33.379	1.00	19.34	O
ATOM	4005	CB	THR	A	508	29.824	9.898	35.522	1.00	27.81	C
ATOM	4006	CG2	THR	A	508	29.006	11.184	35.427	1.00	27.64	C
ATOM	4007	OG1	THR	A	508	29.411	9.172	36.692	1.00	27.38	O
ATOM	4008	N	PHE	A	509	30.175	9.076	31.927	1.00	26.09	N
ATOM	4009	CA	PHE	A	509	31.089	9.209	30.800	1.00	29.27	C
ATOM	4010	C	PHE	A	509	31.562	10.649	30.558	1.00	29.37	C
ATOM	4011	O	PHE	A	509	32.470	10.857	29.742	1.00	29.22	O
ATOM	4012	CB	PHE	A	509	30.475	8.578	29.519	1.00	28.61	C
ATOM	4013	CG	PHE	A	509	29.409	9.410	28.848	1.00	30.39	C
ATOM	4014	CD1	PHE	A	509	29.751	10.520	28.076	1.00	31.31	C
ATOM	4015	CD2	PHE	A	509	28.107	8.935	28.764	1.00	31.82	C
ATOM	4016	CE1	PHE	A	509	28.831	11.131	27.223	1.00	29.90	C
ATOM	4017	CE2	PHE	A	509	27.170	9.543	27.906	1.00	31.41	C
ATOM	4018	CZ	PHE	A	509	27.541	10.639	27.136	1.00	31.04	C
ATOM	4019	N	HIS	A	510	31.160	11.554	31.466	1.00	28.52	N
ATOM	4020	CA	HIS	A	510	31.106	13.001	31.228	1.00	26.73	C
ATOM	4021	C	HIS	A	510	31.430	13.363	29.766	1.00	27.36	C
ATOM	4022	O	HIS	A	510	30.619	13.094	28.892	1.00	27.39	O
ATOM	4023	CB	HIS	A	510	32.021	13.738	32.212	1.00	24.61	C
ATOM	4024	CG	HIS	A	510	32.016	15.224	32.042	1.00	23.20	C
ATOM	4025	CD2	HIS	A	510	31.108	16.156	32.409	1.00	19.13	C
ATOM	4026	ND1	HIS	A	510	32.998	15.900	31.348	1.00	23.83	N
ATOM	4027	CE1	HIS	A	510	32.685	17.177	31.279	1.00	20.37	C
ATOM	4028	NE2	HIS	A	510	31.546	17.360	31.923	1.00	18.67	N
ATOM	4029	N	ALA	A	511	32.650	13.831	29.490	1.00	26.47	N
ATOM	4030	CA	ALA	A	511	33.110	14.040	28.119	1.00	26.06	C
ATOM	4031	C	ALA	A	511	32.517	15.279	27.427	1.00	28.02	C
ATOM	4032	O	ALA	A	511	32.011	15.182	26.314	1.00	30.20	O
ATOM	4033	CB	ALA	A	511	32.831	12.774	27.276	1.00	22.01	C
ATOM	4034	N	ASP	A	512	32.665	16.458	28.031	1.00	27.66	N
ATOM	4035	CA	ASP	A	512	31.940	17.650	27.558	1.00	26.35	C
ATOM	4036	C	ASP	A	512	30.605	17.315	26.859	1.00	25.46	C
ATOM	4037	O	ASP	A	512	30.404	17.662	25.687	1.00	22.46	O
ATOM	4038	CB	ASP	A	512	32.822	18.475	26.622	1.00	28.10	C
ATOM	4039	CG	ASP	A	512	32.789	19.984	26.933	1.00	30.49	C
ATOM	4040	OD1	ASP	A	512	32.547	20.373	28.103	1.00	27.50	O
ATOM	4041	OD2	ASP	A	512	33.113	20.783	26.011	1.00	31.67	O1-
ATOM	4042	N	ILE	A	513	29.781	16.505	27.545	1.00	24.03	N
ATOM	4043	CA	ILE	A	513	28.389	16.258	27.174	1.00	18.35	C
ATOM	4044	C	ILE	A	513	27.730	17.571	26.913	1.00	19.35	C
ATOM	4045	O	ILE	A	513	26.907	17.671	26.015	1.00	19.34	O
ATOM	4046	CB	ILE	A	513	27.557	15.616	28.278	1.00	16.43	C
ATOM	4047	CG1	ILE	A	513	28.316	14.488	28.959	1.00	16.18	C
ATOM	4048	CG2	ILE	A	513	26.270	15.102	27.670	1.00	18.39	C
ATOM	4049	CD1	ILE	A	513	27.473	13.550	29.809	1.00	14.73	C
ATOM	4050	N	CYS	A	514	27.917	18.494	27.857	1.00	19.00	N
ATOM	4051	CA	CYS	A	514	27.567	19.896	27.650	1.00	20.07	C
ATOM	4052	C	CYS	A	514	28.126	20.289	26.300	1.00	22.50	C
ATOM	4053	O	CYS	A	514	29.334	20.276	26.095	1.00	23.48	O
ATOM	4054	CB	CYS	A	514	28.217	20.768	28.708	1.00	17.67	C
ATOM	4055	SG	CYS	A	514	30.001	20.505	28.723	1.00	18.01	S
ATOM	4056	N	THR	A	515	27.228	20.406	25.335	1.00	25.46	N
ATOM	4057	CA	THR	A	515	27.580	20.748	23.970	1.00	26.86	C
ATOM	4058	C	THR	A	515	28.699	19.896	23.361	1.00	26.41	C
ATOM	4059	O	THR	A	515	29.493	20.391	22.563	1.00	28.04	O
ATOM	4060	CB	THR	A	515	27.885	22.263	23.827	1.00	28.89	C
ATOM	4061	CG2	THR	A	515	26.576	23.040	23.525	1.00	29.83	C
ATOM	4062	OG1	THR	A	515	28.464	22.767	25.042	1.00	32.17	O
ATOM	4063	N	LEU	A	516	28.791	18.639	23.804	1.00	22.97	N
ATOM	4064	CA	LEU	A	516	28.855	17.500	22.881	1.00	20.76	C
ATOM	4065	C	LEU	A	516	27.510	17.536	22.152	1.00	20.35	C
ATOM	4066	O	LEU	A	516	27.427	17.980	21.001	1.00	19.65	O
ATOM	4067	CB	LEU	A	516	28.905	16.167	23.656	1.00	18.70	C
ATOM	4068	CG	LEU	A	516	30.129	15.387	24.146	1.00	11.26	C
ATOM	4069	CD1	LEU	A	516	29.858	13.895	24.061	1.00	11.26	C
ATOM	4070	CD2	LEU	A	516	31.309	15.729	23.330	1.00	14.12	C

ATOM	4071	N	SER	A	517	26.489	17.504	23.015	1.00	21.27	N
ATOM	4072	CA	SER	A	517	25.113	17.099	22.728	1.00	18.57	C
ATOM	4073	C	SER	A	517	24.157	18.259	22.953	1.00	17.16	C
ATOM	4074	O	SER	A	517	24.307	18.999	23.915	1.00	14.00	O
ATOM	4075	CB	SER	A	517	24.713	15.963	23.680	1.00	20.93	C
ATOM	4076	OG	SER	A	517	24.982	16.296	25.045	1.00	16.97	O
ATOM	4077	N	GLU	A	518	23.085	18.293	22.169	1.00	18.23	N
ATOM	4078	CA	GLU	A	518	22.162	19.418	22.155	1.00	19.60	C
ATOM	4079	C	GLU	A	518	20.713	18.940	22.104	1.00	19.98	C
ATOM	4080	O	GLU	A	518	20.437	17.817	21.692	1.00	18.87	O
ATOM	4081	CB	GLU	A	518	22.443	20.318	20.947	1.00	22.90	C
ATOM	4082	CG	GLU	A	518	23.891	20.813	20.811	1.00	25.02	C
ATOM	4083	CD	GLU	A	518	23.986	22.237	20.281	1.00	22.60	C
ATOM	4084	OE1	GLU	A	518	23.615	22.475	19.110	1.00	20.70	O
ATOM	4085	OE2	GLU	A	518	24.388	23.126	21.061	1.00	23.36	O1-
ATOM	4086	N	LYS	A	519	19.797	19.884	22.314	1.00	23.54	N
ATOM	4087	CA	LYS	A	519	18.371	19.614	22.551	1.00	24.75	C
ATOM	4088	C	LYS	A	519	18.200	18.755	23.804	1.00	27.97	C
ATOM	4089	O	LYS	A	519	18.733	17.638	23.900	1.00	28.69	O
ATOM	4090	CB	LYS	A	519	17.732	18.927	21.346	1.00	21.56	C
ATOM	4091	CG	LYS	A	519	16.276	18.526	21.524	1.00	16.97	C
ATOM	4092	CD	LYS	A	519	15.692	18.116	20.174	1.00	17.59	C
ATOM	4093	CE	LYS	A	519	14.341	17.430	20.287	1.00	18.48	C
ATOM	4094	NZ	LYS	A	519	13.728	17.036	18.962	1.00	18.54	N1+
ATOM	4095	N	GLU	A	520	17.552	19.324	24.817	1.00	28.25	N
ATOM	4096	CA	GLU	A	520	17.420	18.641	26.099	1.00	27.40	C
ATOM	4097	C	GLU	A	520	16.947	17.204	25.945	1.00	29.51	C
ATOM	4098	O	GLU	A	520	17.010	16.423	26.886	1.00	31.44	O
ATOM	4099	CB	GLU	A	520	16.459	19.417	26.980	1.00	22.39	C
ATOM	4100	CG	GLU	A	520	16.902	20.824	27.137	1.00	17.69	C
ATOM	4101	CD	GLU	A	520	18.391	20.924	27.333	1.00	16.28	C
ATOM	4102	OE1	GLU	A	520	18.877	20.613	28.429	1.00	17.87	O
ATOM	4103	OE2	GLU	A	520	19.084	21.272	26.372	1.00	17.14	O1-
ATOM	4104	N	ARG	A	521	16.406	16.883	24.775	1.00	32.04	N
ATOM	4105	CA	ARG	A	521	16.024	15.522	24.475	1.00	33.58	C
ATOM	4106	C	ARG	A	521	17.287	14.687	24.495	1.00	33.32	C
ATOM	4107	O	ARG	A	521	17.341	13.664	25.185	1.00	35.16	O
ATOM	4108	CB	ARG	A	521	15.298	15.456	23.131	1.00	34.23	C
ATOM	4109	CG	ARG	A	521	13.950	16.232	23.131	1.00	35.49	C
ATOM	4110	CD	ARG	A	521	12.801	15.429	23.747	1.00	38.31	C
ATOM	4111	NE	ARG	A	521	12.787	14.036	23.296	1.00	39.41	N
ATOM	4112	CZ	ARG	A	521	12.362	13.650	22.094	1.00	40.68	C
ATOM	4113	NH1	ARG	A	521	11.733	14.513	21.308	1.00	43.68	N1+
ATOM	4114	NH2	ARG	A	521	12.479	12.384	21.712	1.00	40.15	N
ATOM	4115	N	GLN	A	522	18.369	15.302	24.032	1.00	30.52	N
ATOM	4116	CA	GLN	A	522	19.687	14.689	24.103	1.00	25.95	C
ATOM	4117	C	GLN	A	522	20.450	15.095	25.345	1.00	20.74	C
ATOM	4118	O	GLN	A	522	21.400	14.411	25.727	1.00	15.80	O
ATOM	4119	CB	GLN	A	522	20.515	15.047	22.867	1.00	26.11	C
ATOM	4120	CG	GLN	A	522	19.902	14.640	21.555	1.00	26.14	C
ATOM	4121	CD	GLN	A	522	20.623	15.273	20.397	1.00	28.67	C
ATOM	4122	NE2	GLN	A	522	19.963	15.346	19.241	1.00	26.87	N
ATOM	4123	OE1	GLN	A	522	21.777	15.681	20.533	1.00	27.66	O
ATOM	4124	N	ILE	A	523	20.145	16.264	25.897	1.00	18.75	N
ATOM	4125	CA	ILE	A	523	20.906	16.687	27.069	1.00	19.51	C
ATOM	4126	C	ILE	A	523	20.389	15.978	28.312	1.00	19.06	C
ATOM	4127	O	ILE	A	523	21.156	15.338	29.050	1.00	19.63	O
ATOM	4128	CB	ILE	A	523	20.862	18.206	27.321	1.00	17.88	C
ATOM	4129	CG1	ILE	A	523	20.994	18.975	26.004	1.00	21.59	C
ATOM	4130	CG2	ILE	A	523	21.993	18.578	28.263	1.00	18.05	C
ATOM	4131	CD1	ILE	A	523	21.840	20.265	26.080	1.00	21.79	C
ATOM	4132	N	LYS	A	524	19.076	16.059	28.512	1.00	16.03	N
ATOM	4133	CA	LYS	A	524	18.464	15.493	29.680	1.00	12.94	C
ATOM	4134	C	LYS	A	524	18.691	13.989	29.649	1.00	13.61	C
ATOM	4135	O	LYS	A	524	19.531	13.510	30.385	1.00	13.89	O
ATOM	4136	CB	LYS	A	524	16.986	15.858	29.752	1.00	10.56	C
ATOM	4137	CG	LYS	A	524	16.375	15.511	31.100	1.00	15.61	C
ATOM	4138	CD	LYS	A	524	15.266	16.457	31.565	1.00	13.48	C
ATOM	4139	CE	LYS	A	524	14.946	16.213	33.033	1.00	11.44	C
ATOM	4140	NZ	LYS	A	524	16.147	16.413	33.888	1.00	5.17	N1+
ATOM	4141	N	LYS	A	525	18.184	13.312	28.625	1.00	14.90	N

ATOM	4142	CA	LYS	A	525	18.299	11.851	28.506	1.00	16.22	C
ATOM	4143	C	LYS	A	525	19.550	11.230	29.180	1.00	17.48	C
ATOM	4144	O	LYS	A	525	19.457	10.186	29.837	1.00	16.17	O
ATOM	4145	CB	LYS	A	525	18.212	11.450	27.014	1.00	13.21	C
ATOM	4146	CG	LYS	A	525	18.854	10.133	26.631	1.00	10.66	C
ATOM	4147	CD	LYS	A	525	20.084	10.378	25.775	1.00	15.61	C
ATOM	4148	CE	LYS	A	525	20.840	9.078	25.401	1.00	17.64	C
ATOM	4149	NZ	LYS	A	525	21.001	8.890	23.909	1.00	14.96	N1+
ATOM	4150	N	GLN	A	526	20.707	11.881	29.040	1.00	18.82	N
ATOM	4151	CA	GLN	A	526	21.912	11.429	29.719	1.00	18.67	C
ATOM	4152	C	GLN	A	526	21.750	11.834	31.156	1.00	20.95	C
ATOM	4153	O	GLN	A	526	21.540	10.964	32.010	1.00	21.14	O
ATOM	4154	CB	GLN	A	526	23.178	12.073	29.151	1.00	18.08	C
ATOM	4155	CG	GLN	A	526	23.862	11.294	28.030	1.00	17.91	C
ATOM	4156	CD	GLN	A	526	23.946	9.804	28.298	1.00	20.28	C
ATOM	4157	NE2	GLN	A	526	23.552	9.007	27.320	1.00	22.11	N
ATOM	4158	OE1	GLN	A	526	24.427	9.376	29.339	1.00	22.06	O
ATOM	4159	N	THR	A	527	21.660	13.145	31.398	1.00	21.86	N
ATOM	4160	CA	THR	A	527	21.391	13.654	32.749	1.00	24.95	C
ATOM	4161	C	THR	A	527	20.378	12.766	33.490	1.00	25.85	C
ATOM	4162	O	THR	A	527	20.595	12.424	34.646	1.00	27.72	O
ATOM	4163	CB	THR	A	527	20.843	15.103	32.733	1.00	25.26	C
ATOM	4164	CG2	THR	A	527	21.009	15.752	34.105	1.00	23.79	C
ATOM	4165	OG1	THR	A	527	21.544	15.880	31.752	1.00	24.22	O
ATOM	4166	N	ALA	A	528	19.375	12.254	32.771	1.00	27.33	N
ATOM	4167	CA	ALA	A	528	18.426	11.281	33.333	1.00	26.81	C
ATOM	4168	C	ALA	A	528	18.996	9.857	33.369	1.00	25.61	C
ATOM	4169	O	ALA	A	528	18.965	9.204	34.412	1.00	25.23	O
ATOM	4170	CB	ALA	A	528	17.100	11.306	32.562	1.00	25.49	C
ATOM	4171	N	LEU	A	529	19.654	9.438	32.296	1.00	24.25	N
ATOM	4172	CA	LEU	A	529	20.315	8.139	32.279	1.00	25.05	C
ATOM	4173	C	LEU	A	529	21.267	7.996	33.493	1.00	25.41	C
ATOM	4174	O	LEU	A	529	21.762	6.891	33.797	1.00	25.16	O
ATOM	4175	CB	LEU	A	529	21.086	7.959	30.955	1.00	25.66	C
ATOM	4176	CG	LEU	A	529	20.727	6.802	30.000	1.00	24.04	C
ATOM	4177	CD1	LEU	A	529	20.160	7.352	28.710	1.00	19.96	C
ATOM	4178	CD2	LEU	A	529	21.952	5.961	29.702	1.00	21.68	C
ATOM	4179	N	VAL	A	530	21.523	9.116	34.175	1.00	24.26	N
ATOM	4180	CA	VAL	A	530	22.246	9.102	35.440	1.00	22.74	C
ATOM	4181	C	VAL	A	530	21.369	8.479	36.518	1.00	23.56	C
ATOM	4182	O	VAL	A	530	21.867	7.708	37.360	1.00	22.76	O
ATOM	4183	CB	VAL	A	530	22.618	10.525	35.896	1.00	23.03	C
ATOM	4184	CG1	VAL	A	530	23.610	10.471	37.036	1.00	24.30	C
ATOM	4185	CG2	VAL	A	530	23.200	11.305	34.754	1.00	22.73	C
ATOM	4186	N	GLU	A	531	20.076	8.831	36.502	1.00	22.46	N
ATOM	4187	CA	GLU	A	531	19.139	8.472	37.575	1.00	23.18	C
ATOM	4188	C	GLU	A	531	19.096	7.000	37.982	1.00	24.30	C
ATOM	4189	O	GLU	A	531	19.552	6.668	39.074	1.00	28.02	O
ATOM	4190	CB	GLU	A	531	17.728	8.959	37.262	1.00	22.28	C
ATOM	4191	CG	GLU	A	531	16.791	8.825	38.456	1.00	21.12	C
ATOM	4192	CD	GLU	A	531	16.177	10.137	38.917	1.00	19.64	C
ATOM	4193	OE1	GLU	A	531	16.850	10.917	39.623	1.00	18.65	O
ATOM	4194	OE2	GLU	A	531	14.977	10.337	38.671	1.00	18.88	O1-
ATOM	4195	N	LEU	A	532	18.603	6.115	37.114	1.00	23.63	N
ATOM	4196	CA	LEU	A	532	18.693	4.662	37.362	1.00	24.00	C
ATOM	4197	C	LEU	A	532	19.972	4.240	38.078	1.00	24.04	C
ATOM	4198	O	LEU	A	532	19.907	3.770	39.211	1.00	26.15	O
ATOM	4199	CB	LEU	A	532	18.604	3.854	36.071	1.00	22.16	C
ATOM	4200	CG	LEU	A	532	17.991	4.619	34.926	1.00	23.10	C
ATOM	4201	CD1	LEU	A	532	19.075	5.386	34.246	1.00	28.26	C
ATOM	4202	CD2	LEU	A	532	17.348	3.681	33.973	1.00	27.82	C
ATOM	4203	N	VAL	A	533	21.131	4.440	37.442	1.00	22.79	N
ATOM	4204	CA	VAL	A	533	22.397	3.925	37.974	1.00	19.76	C
ATOM	4205	C	VAL	A	533	22.905	4.700	39.179	1.00	18.89	C
ATOM	4206	O	VAL	A	533	23.874	4.302	39.806	1.00	19.08	O
ATOM	4207	CB	VAL	A	533	23.521	3.852	36.889	1.00	18.59	C
ATOM	4208	CG1	VAL	A	533	23.308	2.624	35.996	1.00	15.07	C
ATOM	4209	CG2	VAL	A	533	23.560	5.142	36.045	1.00	20.41	C
ATOM	4210	N	LYS	A	534	22.243	5.792	39.530	1.00	19.45	N
ATOM	4211	CA	LYS	A	534	22.478	6.365	40.845	1.00	21.27	C
ATOM	4212	C	LYS	A	534	21.526	5.757	41.868	1.00	21.02	C

ATOM	4213	O	LYS	A	534	21.531	6.167	43.023	1.00	20.41	O
ATOM	4214	CB	LYS	A	534	22.357	7.898	40.818	1.00	22.77	C
ATOM	4215	CG	LYS	A	534	20.941	8.444	40.696	1.00	23.98	C
ATOM	4216	CD	LYS	A	534	20.290	8.641	42.054	1.00	28.34	C
ATOM	4217	CE	LYS	A	534	18.928	7.988	42.095	1.00	26.73	C
ATOM	4218	NZ	LYS	A	534	18.037	8.601	41.075	1.00	25.87	N1+
ATOM	4219	N	HIS	A	535	20.627	4.879	41.418	1.00	21.35	N
ATOM	4220	CA	HIS	A	535	19.932	3.950	42.323	1.00	20.99	C
ATOM	4221	C	HIS	A	535	19.602	2.550	41.769	1.00	22.37	C
ATOM	4222	O	HIS	A	535	18.524	2.290	41.214	1.00	22.53	O
ATOM	4223	CB	HIS	A	535	18.722	4.628	42.999	1.00	20.28	C
ATOM	4224	CG	HIS	A	535	17.424	4.489	42.272	1.00	16.66	C
ATOM	4225	CD2	HIS	A	535	17.111	4.603	40.961	1.00	19.33	C
ATOM	4226	ND1	HIS	A	535	16.227	4.401	42.941	1.00	14.54	N
ATOM	4227	CE1	HIS	A	535	15.235	4.488	42.081	1.00	19.05	C
ATOM	4228	NE2	HIS	A	535	15.743	4.611	40.868	1.00	19.23	N
ATOM	4229	N	LYS	A	536	20.476	1.632	42.174	1.00	22.18	N
ATOM	4230	CA	LYS	A	536	20.778	0.332	41.553	1.00	23.44	C
ATOM	4231	C	LYS	A	536	22.184	0.035	42.107	1.00	26.46	C
ATOM	4232	O	LYS	A	536	22.757	-1.031	41.833	1.00	24.60	O
ATOM	4233	CB	LYS	A	536	20.884	0.419	40.023	1.00	18.83	C
ATOM	4234	CG	LYS	A	536	19.572	0.375	39.276	1.00	14.87	C
ATOM	4235	CD	LYS	A	536	19.778	0.539	37.773	1.00	12.15	C
ATOM	4236	CE	LYS	A	536	20.178	-0.774	37.107	1.00	12.18	C
ATOM	4237	NZ	LYS	A	536	19.620	-0.875	35.727	1.00	11.32	N1+
ATOM	4238	N	PRO	A	537	22.840	1.084	42.670	1.00	28.07	N
ATOM	4239	CA	PRO	A	537	23.264	1.257	44.063	1.00	27.56	C
ATOM	4240	C	PRO	A	537	22.139	1.885	44.911	1.00	26.87	C
ATOM	4241	O	PRO	A	537	21.305	2.611	44.393	1.00	26.05	O
ATOM	4242	CB	PRO	A	537	24.475	2.181	43.918	1.00	26.82	C
ATOM	4243	CG	PRO	A	537	24.086	3.095	42.877	1.00	29.18	C
ATOM	4244	CD	PRO	A	537	23.285	2.244	41.868	1.00	30.49	C
ATOM	4245	N	LYS	A	538	22.119	1.587	46.205	1.00	28.13	N
ATOM	4246	CA	LYS	A	538	21.009	1.933	47.109	1.00	27.98	C
ATOM	4247	C	LYS	A	538	19.630	2.015	46.460	1.00	26.40	C
ATOM	4248	O	LYS	A	538	19.218	3.072	45.974	1.00	24.43	O
ATOM	4249	CB	LYS	A	538	21.297	3.229	47.883	1.00	29.62	C
ATOM	4250	CG	LYS	A	538	21.027	3.139	49.423	1.00	25.31	C
ATOM	4251	CD	LYS	A	538	22.012	2.197	50.131	1.00	24.14	C
ATOM	4252	CE	LYS	A	538	21.945	2.359	51.647	1.00	23.68	C
ATOM	4253	NZ	LYS	A	538	23.277	2.474	52.317	1.00	18.75	N1+
ATOM	4254	N	ALA	A	539	18.869	0.938	46.625	1.00	24.41	N
ATOM	4255	CA	ALA	A	539	17.672	0.728	45.840	1.00	24.62	C
ATOM	4256	C	ALA	A	539	16.793	1.958	45.958	1.00	23.58	C
ATOM	4257	O	ALA	A	539	16.534	2.635	44.963	1.00	22.67	O
ATOM	4258	CB	ALA	A	539	16.943	-0.511	46.325	1.00	25.28	C
ATOM	4259	N	THR	A	540	16.559	2.380	47.195	1.00	23.77	N
ATOM	4260	CA	THR	A	540	15.772	3.585	47.440	1.00	23.68	C
ATOM	4261	C	THR	A	540	16.133	4.351	48.713	1.00	25.34	C
ATOM	4262	O	THR	A	540	16.602	3.772	49.709	1.00	23.64	O
ATOM	4263	CB	THR	A	540	14.275	3.272	47.461	1.00	21.40	C
ATOM	4264	CG2	THR	A	540	13.857	2.656	48.804	1.00	20.40	C
ATOM	4265	OG1	THR	A	540	13.546	4.479	47.220	1.00	20.73	O
ATOM	4266	N	LYS	A	541	15.942	5.666	48.654	1.00	26.45	N
ATOM	4267	CA	LYS	A	541	16.156	6.528	49.810	1.00	28.39	C
ATOM	4268	C	LYS	A	541	15.191	7.698	49.794	1.00	27.80	C
ATOM	4269	O	LYS	A	541	14.459	7.884	48.830	1.00	29.39	O
ATOM	4270	CB	LYS	A	541	17.608	7.027	49.847	1.00	29.26	C
ATOM	4271	CG	LYS	A	541	18.614	5.982	50.350	1.00	25.06	C
ATOM	4272	CD	LYS	A	541	18.582	5.886	51.869	1.00	20.48	C
ATOM	4273	CE	LYS	A	541	19.335	7.030	52.505	1.00	17.85	C
ATOM	4274	NZ	LYS	A	541	20.734	7.139	52.022	1.00	16.81	N1+
ATOM	4275	N	GLU	A	542	15.016	8.318	50.952	1.00	29.97	N
ATOM	4276	CA	GLU	A	542	14.101	9.453	51.086	1.00	31.00	C
ATOM	4277	C	GLU	A	542	14.840	10.660	50.478	1.00	30.24	C
ATOM	4278	O	GLU	A	542	15.814	10.456	49.756	1.00	29.44	O
ATOM	4279	CB	GLU	A	542	13.755	9.669	52.582	1.00	29.69	C
ATOM	4280	CG	GLU	A	542	12.602	10.630	52.878	1.00	26.76	C
ATOM	4281	CD	GLU	A	542	11.289	10.221	52.240	1.00	26.79	C
ATOM	4282	OE1	GLU	A	542	11.093	9.010	51.995	1.00	25.61	O
ATOM	4283	OE2	GLU	A	542	10.425	11.112	52.035	1.00	25.89	O1-

ATOM	4284	N	GLN	A	543	14.538	11.862	50.981	1.00	30.95	N
ATOM	4285	CA	GLN	A	543	15.020	13.157	50.451	1.00	29.95	C
ATOM	4286	C	GLN	A	543	16.307	13.073	49.606	1.00	29.03	C
ATOM	4287	O	GLN	A	543	16.294	13.347	48.389	1.00	25.49	O
ATOM	4288	CB	GLN	A	543	15.208	14.161	51.624	1.00	26.87	C
ATOM	4289	CG	GLN	A	543	16.249	13.747	52.684	1.00	21.98	C
ATOM	4290	CD	GLN	A	543	15.650	13.140	53.962	1.00	21.18	C
ATOM	4291	NE2	GLN	A	543	16.336	13.335	55.077	1.00	20.31	N
ATOM	4292	OE1	GLN	A	543	14.613	12.479	53.939	1.00	20.60	O
ATOM	4293	N	LEU	A	544	17.307	12.444	50.225	1.00	26.45	N
ATOM	4294	CA	LEU	A	544	18.653	12.279	49.694	1.00	24.19	C
ATOM	4295	C	LEU	A	544	18.793	12.637	48.228	1.00	22.92	C
ATOM	4296	O	LEU	A	544	19.185	13.758	47.923	1.00	23.86	O
ATOM	4297	CB	LEU	A	544	19.140	10.841	49.914	1.00	21.09	C
ATOM	4298	CG	LEU	A	544	19.186	10.233	51.311	1.00	16.44	C
ATOM	4299	CD1	LEU	A	544	19.940	11.158	52.243	1.00	16.15	C
ATOM	4300	CD2	LEU	A	544	17.789	9.967	51.819	1.00	16.66	C
ATOM	4301	N	LYS	A	545	18.194	11.817	47.362	1.00	20.06	N
ATOM	4302	CA	LYS	A	545	18.749	11.587	46.040	1.00	20.52	C
ATOM	4303	C	LYS	A	545	18.614	12.701	45.002	1.00	22.77	C
ATOM	4304	O	LYS	A	545	18.889	12.471	43.832	1.00	25.57	O
ATOM	4305	CB	LYS	A	545	18.248	10.254	45.461	1.00	18.18	C
ATOM	4306	CG	LYS	A	545	16.821	10.244	44.916	1.00	17.26	C
ATOM	4307	CD	LYS	A	545	15.906	9.289	45.696	1.00	12.74	C
ATOM	4308	CE	LYS	A	545	16.474	7.882	45.800	1.00	8.79	C
ATOM	4309	NZ	LYS	A	545	17.238	7.691	47.056	1.00	6.18	N1+
ATOM	4310	N	ALA	A	546	18.410	13.938	45.439	1.00	23.50	N
ATOM	4311	CA	ALA	A	546	18.556	15.071	44.535	1.00	22.32	C
ATOM	4312	C	ALA	A	546	19.741	15.954	44.903	1.00	22.35	C
ATOM	4313	O	ALA	A	546	20.086	16.854	44.156	1.00	23.42	O
ATOM	4314	CB	ALA	A	546	17.277	15.899	44.490	1.00	23.51	C
ATOM	4315	N	VAL	A	547	20.379	15.694	46.036	1.00	22.24	N
ATOM	4316	CA	VAL	A	547	21.717	16.229	46.279	1.00	23.87	C
ATOM	4317	C	VAL	A	547	22.677	15.472	45.360	1.00	23.89	C
ATOM	4318	O	VAL	A	547	23.877	15.782	45.287	1.00	23.60	O
ATOM	4319	CB	VAL	A	547	22.170	16.040	47.768	1.00	25.64	C
ATOM	4320	CG1	VAL	A	547	22.400	14.566	48.059	1.00	25.82	C
ATOM	4321	CG2	VAL	A	547	23.449	16.861	48.073	1.00	24.16	C
ATOM	4322	N	MET	A	548	22.132	14.475	44.661	1.00	22.84	N
ATOM	4323	CA	MET	A	548	22.841	13.763	43.593	1.00	20.52	C
ATOM	4324	C	MET	A	548	22.779	14.480	42.239	1.00	17.26	C
ATOM	4325	O	MET	A	548	23.748	15.082	41.805	1.00	17.45	O
ATOM	4326	CB	MET	A	548	22.268	12.353	43.445	1.00	21.64	C
ATOM	4327	CG	MET	A	548	22.122	11.605	44.756	1.00	18.22	C
ATOM	4328	SD	MET	A	548	23.630	10.850	45.228	1.00	14.88	S
ATOM	4329	CE	MET	A	548	24.207	12.056	46.438	1.00	16.13	C
ATOM	4330	N	ASP	A	549	21.618	14.485	41.609	1.00	13.78	N
ATOM	4331	CA	ASP	A	549	21.502	15.018	40.264	1.00	16.01	C
ATOM	4332	C	ASP	A	549	21.636	16.528	40.156	1.00	18.51	C
ATOM	4333	O	ASP	A	549	21.000	17.147	39.300	1.00	20.40	O
ATOM	4334	CB	ASP	A	549	20.174	14.604	39.649	1.00	14.29	C
ATOM	4335	CG	ASP	A	549	20.323	13.496	38.677	1.00	14.70	C
ATOM	4336	OD1	ASP	A	549	21.204	12.641	38.891	1.00	11.87	O
ATOM	4337	OD2	ASP	A	549	19.566	13.495	37.687	1.00	17.11	O1-
ATOM	4338	N	ASP	A	550	22.330	17.136	41.102	1.00	19.81	N
ATOM	4339	CA	ASP	A	550	22.847	18.486	40.917	1.00	25.24	C
ATOM	4340	C	ASP	A	550	24.372	18.383	41.093	1.00	27.84	C
ATOM	4341	O	ASP	A	550	25.143	18.785	40.213	1.00	29.39	O
ATOM	4342	CB	ASP	A	550	22.214	19.460	41.935	1.00	24.68	C
ATOM	4343	CG	ASP	A	550	20.805	19.913	41.527	1.00	25.32	C
ATOM	4344	OD1	ASP	A	550	20.168	19.239	40.694	1.00	26.62	O
ATOM	4345	OD2	ASP	A	550	20.334	20.971	42.003	1.00	23.45	O1-
ATOM	4346	N	PHE	A	551	24.778	17.554	42.046	1.00	29.10	N
ATOM	4347	CA	PHE	A	551	26.143	17.056	42.061	1.00	29.37	C
ATOM	4348	C	PHE	A	551	26.481	16.269	40.798	1.00	28.36	C
ATOM	4349	O	PHE	A	551	27.633	15.963	40.538	1.00	29.93	O
ATOM	4350	CB	PHE	A	551	26.361	16.227	43.324	1.00	30.56	C
ATOM	4351	CG	PHE	A	551	26.670	17.064	44.509	1.00	31.41	C
ATOM	4352	CD1	PHE	A	551	26.260	18.398	44.526	1.00	31.60	C
ATOM	4353	CD2	PHE	A	551	27.588	16.637	45.447	1.00	32.87	C
ATOM	4354	CE1	PHE	A	551	26.774	19.299	45.427	1.00	31.67	C

ATOM	4355	CE2	PHE	A	551	28.120	17.542	46.375	1.00	34.18	C
ATOM	4356	CZ	PHE	A	551	27.712	18.878	46.357	1.00	33.28	C
ATOM	4357	N	ALA	A	552	25.470	16.021	39.976	1.00	27.01	N
ATOM	4358	CA	ALA	A	552	25.676	15.608	38.605	1.00	25.37	C
ATOM	4359	C	ALA	A	552	24.735	16.431	37.719	1.00	25.73	C
ATOM	4360	O	ALA	A	552	23.980	15.910	36.886	1.00	24.39	O
ATOM	4361	CB	ALA	A	552	25.417	14.134	38.461	1.00	26.18	C
ATOM	4362	N	ALA	A	553	24.769	17.732	37.975	1.00	24.63	N
ATOM	4363	CA	ALA	A	553	24.274	18.746	37.068	1.00	22.98	C
ATOM	4364	C	ALA	A	553	25.328	19.824	37.002	1.00	25.17	C
ATOM	4365	O	ALA	A	553	25.138	20.792	36.306	1.00	26.82	O
ATOM	4366	CB	ALA	A	553	23.003	19.333	37.572	1.00	23.98	C
ATOM	4367	N	PHE	A	554	26.340	19.752	37.866	1.00	27.85	N
ATOM	4368	CA	PHE	A	554	27.586	20.505	37.668	1.00	28.64	C
ATOM	4369	C	PHE	A	554	28.412	19.808	36.596	1.00	25.54	C
ATOM	4370	O	PHE	A	554	29.324	20.398	36.026	1.00	24.77	O
ATOM	4371	CB	PHE	A	554	28.405	20.583	38.968	1.00	32.92	C
ATOM	4372	CG	PHE	A	554	28.806	21.997	39.366	1.00	37.67	C
ATOM	4373	CD1	PHE	A	554	28.374	23.110	38.618	1.00	39.00	C
ATOM	4374	CD2	PHE	A	554	29.534	22.220	40.544	1.00	37.65	C
ATOM	4375	CE1	PHE	A	554	28.648	24.436	39.051	1.00	39.21	C
ATOM	4376	CE2	PHE	A	554	29.818	23.532	40.984	1.00	40.34	C
ATOM	4377	CZ	PHE	A	554	29.369	24.647	40.233	1.00	39.54	C
ATOM	4378	N	VAL	A	555	28.081	18.543	36.352	1.00	23.93	N
ATOM	4379	CA	VAL	A	555	28.467	17.800	35.147	1.00	22.21	C
ATOM	4380	C	VAL	A	555	28.557	18.670	33.893	1.00	20.12	C
ATOM	4381	O	VAL	A	555	29.305	18.360	32.975	1.00	18.10	O
ATOM	4382	CB	VAL	A	555	27.484	16.602	34.897	1.00	21.30	C
ATOM	4383	CG1	VAL	A	555	26.074	17.094	34.820	1.00	22.10	C
ATOM	4384	CG2	VAL	A	555	27.815	15.870	33.632	1.00	22.52	C
ATOM	4385	N	GLU	A	556	27.829	19.772	33.855	1.00	20.36	N
ATOM	4386	CA	GLU	A	556	28.107	20.753	32.822	1.00	22.51	C
ATOM	4387	C	GLU	A	556	29.129	21.755	33.324	1.00	22.13	C
ATOM	4388	O	GLU	A	556	30.316	21.503	33.183	1.00	21.46	O
ATOM	4389	CB	GLU	A	556	26.827	21.440	32.333	1.00	24.42	C
ATOM	4390	CG	GLU	A	556	26.014	20.591	31.348	1.00	25.09	C
ATOM	4391	CD	GLU	A	556	25.397	19.340	32.002	1.00	29.89	C
ATOM	4392	OE1	GLU	A	556	26.157	18.391	32.313	1.00	28.92	O
ATOM	4393	OE2	GLU	A	556	24.150	19.300	32.192	1.00	30.78	O1-
ATOM	4394	N	LYS	A	557	28.693	22.721	34.130	1.00	23.25	N
ATOM	4395	CA	LYS	A	557	29.514	23.885	34.484	1.00	26.16	C
ATOM	4396	C	LYS	A	557	30.893	23.667	35.189	1.00	28.55	C
ATOM	4397	O	LYS	A	557	31.455	24.625	35.736	1.00	32.54	O
ATOM	4398	CB	LYS	A	557	28.688	24.876	35.319	1.00	25.61	C
ATOM	4399	CG	LYS	A	557	27.221	24.986	34.979	1.00	25.26	C
ATOM	4400	CD	LYS	A	557	26.383	25.468	36.212	1.00	26.93	C
ATOM	4401	CE	LYS	A	557	24.868	25.275	35.938	1.00	29.30	C
ATOM	4402	NZ	LYS	A	557	23.869	26.075	36.726	1.00	26.93	N1+
ATOM	4403	N	CYS	A	558	31.439	22.448	35.204	1.00	28.73	N
ATOM	4404	CA	CYS	A	558	32.899	22.266	35.342	1.00	26.69	C
ATOM	4405	C	CYS	A	558	33.460	21.735	34.016	1.00	26.16	C
ATOM	4406	O	CYS	A	558	34.575	21.193	33.962	1.00	25.82	O
ATOM	4407	CB	CYS	A	558	33.247	21.290	36.484	1.00	27.88	C
ATOM	4408	SG	CYS	A	558	34.126	19.745	35.984	1.00	25.90	S
ATOM	4409	N	CYS	A	559	32.702	21.924	32.939	1.00	24.28	N
ATOM	4410	CA	CYS	A	559	33.067	21.378	31.638	1.00	24.57	C
ATOM	4411	C	CYS	A	559	33.527	22.453	30.635	1.00	25.31	C
ATOM	4412	O	CYS	A	559	32.955	22.594	29.534	1.00	22.80	O
ATOM	4413	CB	CYS	A	559	31.882	20.610	31.057	1.00	24.33	C
ATOM	4414	SG	CYS	A	559	30.664	21.671	30.225	1.00	18.67	S
ATOM	4415	N	LYS	A	560	34.588	23.170	30.993	1.00	24.30	N
ATOM	4416	CA	LYS	A	560	35.196	24.124	30.082	1.00	24.61	C
ATOM	4417	C	LYS	A	560	36.487	24.703	30.634	1.00	24.70	C
ATOM	4418	O	LYS	A	560	36.873	24.417	31.765	1.00	22.58	O
ATOM	4419	CB	LYS	A	560	34.215	25.242	29.754	1.00	25.54	C
ATOM	4420	CG	LYS	A	560	33.656	25.152	28.339	1.00	26.31	C
ATOM	4421	CD	LYS	A	560	32.511	26.109	28.108	1.00	24.00	C
ATOM	4422	CE	LYS	A	560	31.495	25.480	27.188	1.00	25.24	C
ATOM	4423	NZ	LYS	A	560	31.446	24.014	27.391	1.00	26.72	N1+
ATOM	4424	N	ALA	A	561	37.126	25.555	29.841	1.00	24.25	N
ATOM	4425	CA	ALA	A	561	38.507	25.915	30.078	1.00	24.71	C

ATOM	4426	C	ALA	A	561	39.300	24.620	30.175	1.00	26.65	C
ATOM	4427	O	ALA	A	561	39.333	23.999	31.218	1.00	27.41	O
ATOM	4428	CB	ALA	A	561	38.622	26.707	31.359	1.00	25.16	C
ATOM	4429	N	ASP	A	562	39.642	24.065	29.016	1.00	30.60	N
ATOM	4430	CA	ASP	A	562	40.525	22.892	28.929	1.00	30.34	C
ATOM	4431	C	ASP	A	562	39.961	21.653	29.616	1.00	31.09	C
ATOM	4432	O	ASP	A	562	40.593	21.132	30.562	1.00	31.08	O
ATOM	4433	CB	ASP	A	562	41.905	23.198	29.516	1.00	28.16	C
ATOM	4434	CG	ASP	A	562	42.952	22.216	29.054	1.00	25.04	C
ATOM	4435	OD1	ASP	A	562	42.717	21.514	28.053	1.00	24.20	O
ATOM	4436	OD2	ASP	A	562	44.000	22.117	29.707	1.00	28.51	O1-
ATOM	4437	N	ASP	A	563	38.979	21.040	28.935	1.00	30.71	N
ATOM	4438	CA	ASP	A	563	38.059	20.068	29.538	1.00	28.76	C
ATOM	4439	C	ASP	A	563	38.485	18.610	29.373	1.00	28.00	C
ATOM	4440	O	ASP	A	563	39.612	18.256	29.693	1.00	28.65	O
ATOM	4441	CB	ASP	A	563	36.615	20.261	29.028	1.00	30.30	C
ATOM	4442	CG	ASP	A	563	36.508	21.125	27.746	1.00	33.01	C
ATOM	4443	OD1	ASP	A	563	37.154	22.206	27.640	1.00	32.70	O
ATOM	4444	OD2	ASP	A	563	35.634	20.803	26.908	1.00	31.15	O1-
ATOM	4445	N	LYS	A	564	37.563	17.749	28.966	1.00	29.53	N
ATOM	4446	CA	LYS	A	564	37.756	16.283	28.945	1.00	31.53	C
ATOM	4447	C	LYS	A	564	38.549	15.598	30.061	1.00	32.06	C
ATOM	4448	O	LYS	A	564	38.498	14.370	30.188	1.00	32.29	O
ATOM	4449	CB	LYS	A	564	38.296	15.821	27.591	1.00	30.45	C
ATOM	4450	CG	LYS	A	564	37.233	15.744	26.497	1.00	31.07	C
ATOM	4451	CD	LYS	A	564	36.111	14.775	26.849	1.00	30.53	C
ATOM	4452	CE	LYS	A	564	35.347	14.318	25.598	1.00	32.26	C
ATOM	4453	NZ	LYS	A	564	35.355	12.798	25.474	1.00	34.40	N1+
ATOM	4454	N	GLU	A	565	39.050	16.383	31.006	1.00	34.20	N
ATOM	4455	CA	GLU	A	565	39.522	15.830	32.269	1.00	33.71	C
ATOM	4456	C	GLU	A	565	39.123	16.707	33.477	1.00	33.57	C
ATOM	4457	O	GLU	A	565	39.325	16.298	34.620	1.00	36.71	O
ATOM	4458	CB	GLU	A	565	41.038	15.547	32.203	1.00	31.00	C
ATOM	4459	CG	GLU	A	565	41.948	16.433	33.029	1.00	28.71	C
ATOM	4460	CD	GLU	A	565	41.705	17.906	32.810	1.00	28.14	C
ATOM	4461	OE1	GLU	A	565	41.476	18.343	31.664	1.00	27.94	O
ATOM	4462	OE2	GLU	A	565	41.605	18.611	33.826	1.00	31.46	O1-
ATOM	4463	N	THR	A	566	38.550	17.891	33.230	1.00	31.43	N
ATOM	4464	CA	THR	A	566	37.891	18.648	34.290	1.00	30.06	C
ATOM	4465	C	THR	A	566	36.888	17.740	34.978	1.00	31.89	C
ATOM	4466	O	THR	A	566	37.183	17.117	36.017	1.00	33.59	O
ATOM	4467	CB	THR	A	566	37.091	19.837	33.754	1.00	27.34	C
ATOM	4468	CG2	THR	A	566	37.562	21.118	34.385	1.00	29.75	C
ATOM	4469	OG1	THR	A	566	37.226	19.915	32.329	1.00	28.29	O
ATOM	4470	N	CYS	A	567	35.700	17.644	34.390	1.00	32.18	N
ATOM	4471	CA	CYS	A	567	34.654	16.825	34.979	1.00	31.39	C
ATOM	4472	C	CYS	A	567	34.889	15.378	34.594	1.00	29.93	C
ATOM	4473	O	CYS	A	567	34.352	14.909	33.590	1.00	29.42	O
ATOM	4474	CB	CYS	A	567	33.262	17.276	34.513	1.00	29.68	C
ATOM	4475	SG	CYS	A	567	33.055	19.080	34.407	1.00	25.19	S
ATOM	4476	N	PHE	A	568	35.686	14.688	35.405	1.00	28.61	N
ATOM	4477	CA	PHE	A	568	35.928	13.255	35.250	1.00	28.31	C
ATOM	4478	C	PHE	A	568	36.751	12.792	36.449	1.00	29.41	C
ATOM	4479	O	PHE	A	568	36.664	11.642	36.860	1.00	31.51	O
ATOM	4480	CB	PHE	A	568	36.729	12.998	33.976	1.00	25.95	C
ATOM	4481	CG	PHE	A	568	36.206	11.876	33.139	1.00	21.28	C
ATOM	4482	CD1	PHE	A	568	35.062	12.035	32.394	1.00	20.57	C
ATOM	4483	CD2	PHE	A	568	36.903	10.698	33.038	1.00	20.14	C
ATOM	4484	CE1	PHE	A	568	34.622	11.051	31.574	1.00	17.15	C
ATOM	4485	CE2	PHE	A	568	36.471	9.710	32.217	1.00	17.57	C
ATOM	4486	CZ	PHE	A	568	35.321	9.888	31.481	1.00	19.73	C
ATOM	4487	N	ALA	A	569	37.683	13.636	36.877	1.00	28.87	N
ATOM	4488	CA	ALA	A	569	38.383	13.420	38.134	1.00	28.65	C
ATOM	4489	C	ALA	A	569	37.468	13.939	39.220	1.00	28.03	C
ATOM	4490	O	ALA	A	569	37.356	13.378	40.309	1.00	26.78	O
ATOM	4491	CB	ALA	A	569	39.710	14.213	38.138	1.00	30.00	C
ATOM	4492	N	GLU	A	570	36.832	15.057	38.898	1.00	30.62	N
ATOM	4493	CA	GLU	A	570	36.151	15.878	39.882	1.00	30.63	C
ATOM	4494	C	GLU	A	570	34.945	15.123	40.390	1.00	30.05	C
ATOM	4495	O	GLU	A	570	35.001	14.560	41.483	1.00	29.90	O
ATOM	4496	CB	GLU	A	570	35.749	17.210	39.258	1.00	28.89	C

ATOM	4497	CG	GLU	A	570	36.936	18.084	38.893	1.00	27.66	C
ATOM	4498	CD	GLU	A	570	36.512	19.417	38.301	1.00	29.60	C
ATOM	4499	OE1	GLU	A	570	35.295	19.714	38.265	1.00	27.40	O
ATOM	4500	OE2	GLU	A	570	37.407	20.200	37.922	1.00	31.42	O1-
ATOM	4501	N	GLU	A	571	33.921	15.009	39.544	1.00	28.64	N
ATOM	4502	CA	GLU	A	571	32.793	14.109	39.781	1.00	29.73	C
ATOM	4503	C	GLU	A	571	33.172	12.828	40.555	1.00	29.42	C
ATOM	4504	O	GLU	A	571	32.703	12.600	41.666	1.00	26.88	O
ATOM	4505	CB	GLU	A	571	32.153	13.698	38.444	1.00	31.90	C
ATOM	4506	CG	GLU	A	571	31.887	14.842	37.444	1.00	33.12	C
ATOM	4507	CD	GLU	A	571	30.475	15.396	37.551	1.00	31.21	C
ATOM	4508	OE1	GLU	A	571	30.276	16.300	38.392	1.00	29.20	O
ATOM	4509	OE2	GLU	A	571	29.584	14.930	36.791	1.00	28.63	O1-
ATOM	4510	N	GLY	A	572	34.072	12.032	39.986	1.00	29.49	N
ATOM	4511	CA	GLY	A	572	34.301	10.677	40.478	1.00	28.71	C
ATOM	4512	C	GLY	A	572	34.559	10.612	41.965	1.00	25.97	C
ATOM	4513	O	GLY	A	572	34.133	9.670	42.649	1.00	25.03	O
ATOM	4514	N	LYS	A	573	35.292	11.596	42.463	1.00	25.00	N
ATOM	4515	CA	LYS	A	573	35.292	11.839	43.886	1.00	26.40	C
ATOM	4516	C	LYS	A	573	33.907	12.403	44.227	1.00	27.40	C
ATOM	4517	O	LYS	A	573	33.053	11.637	44.652	1.00	28.80	O
ATOM	4518	CB	LYS	A	573	36.422	12.802	44.278	1.00	25.67	C
ATOM	4519	CG	LYS	A	573	37.835	12.284	43.924	1.00	24.04	C
ATOM	4520	CD	LYS	A	573	38.725	12.132	45.169	1.00	21.49	C
ATOM	4521	CE	LYS	A	573	39.633	13.331	45.408	1.00	17.20	C
ATOM	4522	NZ	LYS	A	573	40.867	12.848	46.062	1.00	15.20	N1+
ATOM	4523	N	LYS	A	574	33.581	13.580	43.695	1.00	26.75	N
ATOM	4524	CA	LYS	A	574	32.400	14.324	44.131	1.00	25.51	C
ATOM	4525	C	LYS	A	574	30.989	13.898	43.651	1.00	25.42	C
ATOM	4526	O	LYS	A	574	30.011	14.569	44.005	1.00	25.17	O
ATOM	4527	CB	LYS	A	574	32.612	15.817	43.872	1.00	27.14	C
ATOM	4528	CG	LYS	A	574	32.851	16.209	42.416	1.00	28.72	C
ATOM	4529	CD	LYS	A	574	32.816	17.739	42.193	1.00	28.75	C
ATOM	4530	CE	LYS	A	574	31.382	18.254	41.916	1.00	28.20	C
ATOM	4531	NZ	LYS	A	574	31.384	19.599	41.254	1.00	28.78	N1+
ATOM	4532	N	LEU	A	575	30.860	12.683	43.105	1.00	23.44	N
ATOM	4533	CA	LEU	A	575	29.559	12.086	42.759	1.00	25.31	C
ATOM	4534	C	LEU	A	575	29.409	10.605	43.176	1.00	27.47	C
ATOM	4535	O	LEU	A	575	28.300	10.045	43.137	1.00	26.81	O
ATOM	4536	CB	LEU	A	575	29.301	12.205	41.264	1.00	24.20	C
ATOM	4537	CG	LEU	A	575	28.778	10.958	40.541	1.00	24.99	C
ATOM	4538	CD1	LEU	A	575	27.265	11.091	40.260	1.00	22.96	C
ATOM	4539	CD2	LEU	A	575	29.587	10.746	39.253	1.00	23.54	C
ATOM	4540	N	VAL	A	576	30.545	9.953	43.443	1.00	30.48	N
ATOM	4541	CA	VAL	A	576	30.594	8.583	43.980	1.00	28.40	C
ATOM	4542	C	VAL	A	576	30.933	8.698	45.451	1.00	26.50	C
ATOM	4543	O	VAL	A	576	30.389	7.969	46.264	1.00	26.24	O
ATOM	4544	CB	VAL	A	576	31.703	7.709	43.313	1.00	29.72	C
ATOM	4545	CG1	VAL	A	576	31.613	6.269	43.830	1.00	31.31	C
ATOM	4546	CG2	VAL	A	576	31.568	7.728	41.788	1.00	28.27	C
ATOM	4547	N	ALA	A	577	31.839	9.614	45.783	1.00	26.23	N
ATOM	4548	CA	ALA	A	577	32.042	10.018	47.171	1.00	27.55	C
ATOM	4549	C	ALA	A	577	30.723	10.465	47.754	1.00	27.60	C
ATOM	4550	O	ALA	A	577	30.498	10.341	48.959	1.00	29.06	O
ATOM	4551	CB	ALA	A	577	33.057	11.163	47.280	1.00	24.38	C
ATOM	4552	N	ALA	A	578	29.896	11.084	46.921	1.00	26.48	N
ATOM	4553	CA	ALA	A	578	28.663	11.639	47.421	1.00	28.56	C
ATOM	4554	C	ALA	A	578	27.604	10.545	47.504	1.00	30.37	C
ATOM	4555	O	ALA	A	578	26.515	10.788	48.040	1.00	32.62	O
ATOM	4556	CB	ALA	A	578	28.198	12.775	46.522	1.00	27.95	C
ATOM	4557	N	SER	A	579	27.969	9.336	47.045	1.00	30.43	N
ATOM	4558	CA	SER	A	579	27.041	8.214	46.794	1.00	28.38	C
ATOM	4559	C	SER	A	579	26.717	7.338	48.012	1.00	28.20	C
ATOM	4560	O	SER	A	579	27.423	7.379	49.026	1.00	27.16	O
ATOM	4561	CB	SER	A	579	27.600	7.308	45.678	1.00	26.10	C
ATOM	4562	OG	SER	A	579	27.127	7.695	44.407	1.00	21.67	O
ATOM	4563	N	GLN	A	580	25.858	6.349	47.756	1.00	28.20	N
ATOM	4564	CA	GLN	A	580	25.294	5.456	48.775	1.00	30.20	C
ATOM	4565	C	GLN	A	580	25.251	5.794	50.275	1.00	29.79	C
ATOM	4566	O	GLN	A	580	24.240	6.301	50.763	1.00	29.46	O
ATOM	4567	CB	GLN	A	580	25.887	4.059	48.652	1.00	31.82	C

```
ATOM   4568  CG  GLN A 580      24.809   3.035  48.450  1.00 35.01           C
ATOM   4569  CD  GLN A 580      24.605   2.755  46.984  1.00 39.20           C
ATOM   4570  NE2 GLN A 580      24.214   3.795  46.222  1.00 39.46           N
ATOM   4571  OE1 GLN A 580      24.873   1.642  46.513  1.00 37.19           O
ATOM   4572  N   ALA A 581      26.147   5.153  51.022  1.00 28.09           N
ATOM   4573  CA  ALA A 581      25.904   4.949  52.433  1.00 28.48           C
ATOM   4574  C   ALA A 581      26.749   5.880  53.305  1.00 31.33           C
ATOM   4575  O   ALA A 581      27.878   5.542  53.689  1.00 33.68           O
ATOM   4576  CB  ALA A 581      26.139   3.517  52.786  1.00 27.41           C
ATOM   4577  N   ALA A 582      26.292   7.133  53.354  1.00 32.13           N
ATOM   4578  CA  ALA A 582      26.690   8.140  54.330  1.00 30.12           C
ATOM   4579  C   ALA A 582      25.992   9.430  53.865  1.00 32.98           C
ATOM   4580  O   ALA A 582      25.336  10.117  54.686  1.00 34.50           O
ATOM   4581  CB  ALA A 582      28.212   8.325  54.362  1.00 23.03           C
ATOM   4582  N   LEU A 583      26.076   9.711  52.548  1.00 32.99           N
ATOM   4583  CA  LEU A 583      25.497  10.927  51.932  1.00 32.69           C
ATOM   4584  C   LEU A 583      24.530  10.469  50.841  1.00 33.35           C
ATOM   4585  O   LEU A 583      23.484  11.124  50.604  1.00 31.85           O
ATOM   4586  CB  LEU A 583      26.588  11.815  51.276  1.00 28.59           C
ATOM   4587  CG  LEU A 583      27.733  12.582  51.993  1.00 26.24           C
ATOM   4588  CD1 LEU A 583      27.189  13.656  52.930  1.00 24.68           C
ATOM   4589  CD2 LEU A 583      28.651  11.626  52.741  1.00 22.20           C
HETATM 4590  C   UNK     0      -8.743   1.026  14.253  0.00  0.00       .001 A
HETATM 4591  C   UNK     0      -9.951   1.717  14.711  0.00  0.00       .029 A
HETATM 4592  C   UNK     0      -7.512  -0.949  14.907  0.00  0.00       .033 A
HETATM 4593  C   UNK     0      -6.608  -0.707  13.898  0.00  0.00       .039 A
HETATM 4594  C   UNK     0      -8.588  -0.088  15.098  0.00  0.00       .043 A
HETATM 4595  C   UNK     0      -7.817   1.264  13.235  0.00  0.00       .050 A
HETATM 4596  C   UNK     0      -6.759   0.395  13.059  0.00  0.00       .066 A
HETATM 4597  C   UNK     0     -10.542   2.972  14.124  0.00  0.00       .070 C
HETATM 4598  N   UNK     0     -11.768   2.489  12.060  0.00  0.00       .078 N
HETATM 4599  C   UNK     0     -10.433   1.018  15.751  0.00  0.00       .096 A
HETATM 4600  H   UNK     0      -9.778  -0.716  16.700  0.00  0.00       .165 H
HETATM 4601  H   UNK     0      -6.103   1.337  11.466  0.00  0.00       .217 H
HETATM 4602  C   UNK     0     -11.908   2.656  13.513  0.00  0.00       .271 C
HETATM 4603  H   UNK     0     -10.808   2.603  11.774  0.00  0.00       .312 H
HETATM 4604  H   UNK     0     -12.130   1.595  11.763  0.00  0.00       .312 H
HETATM 4605  O   UNK     0      -5.857   0.620  12.066  0.00  0.00       .361 O
HETATM 4606  N   UNK     0      -9.632  -0.063  15.998  0.00  0.00       .365 N
CONECT 4590 4595 4591 4594
CONECT 4591 4590 4597 4599
CONECT 4592 4594 4593
CONECT 4593 4592 4596
CONECT 4594 4592 4590 4606
CONECT 4595 4590 4596
CONECT 4596 4593 4595 4605
CONECT 4597 4591 4602
CONECT 4598 4603 4604 4602
CONECT 4599 4591 4606
CONECT 4600 4606
CONECT 4601 4605
CONECT 4602 4597 4598
CONECT 4603 4598
CONECT 4604 4598
CONECT 4605 4596 4601
CONECT 4606 4594 4599 4600
END
```

ALB_TRP.pdb

```
CRYST1   58.860   88.280   60.680  90.00 101.85  90.00 P 1 21 1      0
ATOM      1  N   LYS A   4      -4.155 -21.388 -11.190  1.00 29.11           N
ATOM      2  CA  LYS A   4      -3.964 -19.918 -11.113  1.00 27.16           C
ATOM      3  C   LYS A   4      -2.692 -19.737 -10.315  1.00 26.91           C
ATOM      4  O   LYS A   4      -2.108 -20.724  -9.881  1.00 25.06           O
ATOM      5  CB  LYS A   4      -5.146 -19.257 -10.398  1.00 25.62           C
ATOM      6  CG  LYS A   4      -6.503 -19.517 -11.051  1.00 24.88           C
ATOM      7  CD  LYS A   4      -7.331 -18.233 -11.175  1.00 26.34           C
ATOM      8  CE  LYS A   4      -8.649 -18.441 -11.958  1.00 27.70           C
ATOM      9  NZ  LYS A   4      -9.717 -19.227 -11.215  1.00 25.48           N1+
ATOM     10  N   SER A   5      -2.168 -18.518 -10.290  1.00 28.23           N
ATOM     11  CA  SER A   5      -0.939 -18.247  -9.551  1.00 30.21           C
ATOM     12  C   SER A   5      -1.207 -17.300  -8.380  1.00 30.60           C
ATOM     13  O   SER A   5      -2.299 -16.719  -8.247  1.00 28.63           O
ATOM     14  CB  SER A   5       0.155 -17.652 -10.476  1.00 30.07           C
ATOM     15  OG  SER A   5       0.110 -16.225 -10.543  1.00 25.84           O
ATOM     16  N   GLU A   6      -0.206 -17.159  -7.526  1.00 29.67           N
ATOM     17  CA  GLU A   6      -0.250 -16.146  -6.511  1.00 28.73           C
ATOM     18  C   GLU A   6       0.506 -14.913  -6.949  1.00 25.98           C
ATOM     19  O   GLU A   6      -0.094 -13.854  -7.065  1.00 25.99           O
ATOM     20  CB  GLU A   6       0.289 -16.706  -5.196  1.00 32.20           C
ATOM     21  CG  GLU A   6      -0.730 -17.561  -4.457  1.00 34.86           C
ATOM     22  CD  GLU A   6      -2.158 -17.229  -4.877  1.00 37.50           C
ATOM     23  OE1 GLU A   6      -2.669 -16.146  -4.502  1.00 36.49           O
ATOM     24  OE2 GLU A   6      -2.746 -18.025  -5.641  1.00 41.69           O1-
ATOM     25  N   VAL A   7       1.732 -15.105  -7.428  1.00 26.46           N
ATOM     26  CA  VAL A   7       2.660 -13.992  -7.658  1.00 28.01           C
ATOM     27  C   VAL A   7       2.019 -12.877  -8.472  1.00 29.09           C
ATOM     28  O   VAL A   7       1.728 -11.784  -7.951  1.00 28.54           O
ATOM     29  CB  VAL A   7       3.929 -14.439  -8.413  1.00 27.74           C
ATOM     30  CG1 VAL A   7       4.947 -13.333  -8.405  1.00 29.26           C
ATOM     31  CG2 VAL A   7       4.524 -15.668  -7.773  1.00 31.03           C
ATOM     32  N   ALA A   8       1.681 -13.202  -9.716  1.00 29.77           N
ATOM     33  CA  ALA A   8       1.215 -12.182 -10.646  1.00 29.95           C
ATOM     34  C   ALA A   8      -0.133 -11.672 -10.189  1.00 29.29           C
ATOM     35  O   ALA A   8      -0.491 -10.525 -10.473  1.00 30.27           O
ATOM     36  CB  ALA A   8       1.117 -12.736 -12.073  1.00 29.88           C
ATOM     37  N   HIS A   9      -0.866 -12.500  -9.452  1.00 28.46           N
ATOM     38  CA  HIS A   9      -2.215 -12.121  -9.052  1.00 28.28           C
ATOM     39  C   HIS A   9      -2.121 -10.972  -8.070  1.00 28.15           C
ATOM     40  O   HIS A   9      -2.926 -10.045  -8.124  1.00 28.25           O
ATOM     41  CB  HIS A   9      -2.961 -13.275  -8.387  1.00 26.15           C
ATOM     42  CG  HIS A   9      -4.260 -12.866  -7.776  1.00 23.88           C
ATOM     43  CD2 HIS A   9      -5.086 -11.821  -8.052  1.00 22.55           C
ATOM     44  ND1 HIS A   9      -4.868 -13.577  -6.766  1.00 22.06           N
ATOM     45  CE1 HIS A   9      -6.009 -12.994  -6.443  1.00 23.71           C
ATOM     46  NE2 HIS A   9      -6.162 -11.927  -7.212  1.00 25.62           N
ATOM     47  N   ARG A  10      -1.181 -11.070  -7.134  1.00 26.61           N
ATOM     48  CA  ARG A  10      -0.934  -9.977  -6.211  1.00 26.64           C
ATOM     49  C   ARG A  10      -0.481  -8.776  -7.042  1.00 26.73           C
ATOM     50  O   ARG A  10      -1.080  -7.701  -6.952  1.00 26.46           O
ATOM     51  CB  ARG A  10       0.123 -10.384  -5.179  1.00 25.45           C
ATOM     52  CG  ARG A  10      -0.411 -11.233  -3.973  1.00 26.28           C
ATOM     53  CD  ARG A  10      -1.808 -11.885  -4.176  1.00 22.90           C
ATOM     54  NE  ARG A  10      -2.565 -11.999  -2.920  1.00 22.44           N
ATOM     55  CZ  ARG A  10      -3.843 -12.387  -2.815  1.00 23.29           C
ATOM     56  NH1 ARG A  10      -4.491 -12.934  -3.836  1.00 20.37           N1+
ATOM     57  NH2 ARG A  10      -4.460 -12.295  -1.650  1.00 22.05           N
ATOM     58  N   PHE A  11       0.332  -9.061  -8.061  1.00 27.91           N
ATOM     59  CA  PHE A  11       0.735  -8.053  -9.045  1.00 25.84           C
ATOM     60  C   PHE A  11      -0.452  -7.383  -9.727  1.00 24.74           C
ATOM     61  O   PHE A  11      -0.444  -6.168  -9.938  1.00 25.29           O
ATOM     62  CB  PHE A  11       1.652  -8.664 -10.105  1.00 24.40           C
ATOM     63  CG  PHE A  11       2.851  -7.809 -10.430  1.00 27.14           C
ATOM     64  CD1 PHE A  11       2.762  -6.785 -11.368  1.00 26.91           C
ATOM     65  CD2 PHE A  11       4.042  -7.965  -9.728  1.00 26.00           C
ATOM     66  CE1 PHE A  11       3.829  -5.934 -11.585  1.00 27.01           C
ATOM     67  CE2 PHE A  11       5.115  -7.111  -9.945  1.00 25.82           C
ATOM     68  CZ  PHE A  11       5.009  -6.095 -10.871  1.00 25.28           C
```

```
ATOM     69  N   LYS A  12      -1.530  -8.136  -9.903  1.00 23.91           N
ATOM     70  CA  LYS A  12      -2.692  -7.668 -10.651  1.00 22.95           C
ATOM     71  C   LYS A  12      -3.685  -6.841  -9.819  1.00 22.94           C
ATOM     72  O   LYS A  12      -4.825  -6.624 -10.255  1.00 22.98           O
ATOM     73  CB  LYS A  12      -3.449  -8.860 -11.267  1.00 23.41           C
ATOM     74  CG  LYS A  12      -2.859  -9.489 -12.523  1.00 18.48           C
ATOM     75  CD  LYS A  12      -3.793 -10.579 -13.069  1.00 15.47           C
ATOM     76  CE  LYS A  12      -3.024 -11.641 -13.836  1.00 13.47           C
ATOM     77  NZ  LYS A  12      -2.824 -11.308 -15.273  1.00 12.20           N1+
ATOM     78  N   ASP A  13      -3.313  -6.444  -8.606  1.00 22.30           N
ATOM     79  CA  ASP A  13      -4.140  -5.462  -7.897  1.00 22.25           C
ATOM     80  C   ASP A  13      -3.358  -4.266  -7.363  1.00 20.77           C
ATOM     81  O   ASP A  13      -3.612  -3.114  -7.758  1.00 18.06           O
ATOM     82  CB  ASP A  13      -4.929  -6.102  -6.748  1.00 27.23           C
ATOM     83  CG  ASP A  13      -4.846  -7.609  -6.734  1.00 30.68           C
ATOM     84  OD1 ASP A  13      -3.712  -8.130  -6.758  1.00 31.81           O
ATOM     85  OD2 ASP A  13      -5.905  -8.262  -6.569  1.00 31.63           O1-
ATOM     86  N   LEU A  14      -2.360  -4.555  -6.527  1.00 19.90           N
ATOM     87  CA  LEU A  14      -1.567  -3.530  -5.840  1.00 19.12           C
ATOM     88  C   LEU A  14      -0.542  -2.906  -6.802  1.00 21.59           C
ATOM     89  O   LEU A  14       0.414  -2.214  -6.388  1.00 20.70           O
ATOM     90  CB  LEU A  14      -0.867  -4.154  -4.625  1.00 16.73           C
ATOM     91  CG  LEU A  14       0.256  -5.186  -4.824  1.00 15.13           C
ATOM     92  CD1 LEU A  14       1.447  -4.781  -3.981  1.00 11.65           C
ATOM     93  CD2 LEU A  14      -0.189  -6.568  -4.441  1.00 11.37           C
ATOM     94  N   GLY A  15      -0.829  -3.065  -8.092  1.00 21.89           N
ATOM     95  CA  GLY A  15       0.087  -2.683  -9.140  1.00 23.39           C
ATOM     96  C   GLY A  15       1.491  -3.220  -8.965  1.00 24.62           C
ATOM     97  O   GLY A  15       1.700  -4.264  -8.353  1.00 23.01           O
ATOM     98  N   GLU A  16       2.457  -2.395  -9.367  1.00 26.22           N
ATOM     99  CA  GLU A  16       3.837  -2.809  -9.504  1.00 25.91           C
ATOM    100  C   GLU A  16       4.725  -2.038  -8.535  1.00 27.19           C
ATOM    101  O   GLU A  16       5.421  -2.680  -7.760  1.00 26.80           O
ATOM    102  CB  GLU A  16       4.282  -2.641 -10.966  1.00 27.05           C
ATOM    103  CG  GLU A  16       5.730  -2.204 -11.175  1.00 28.89           C
ATOM    104  CD  GLU A  16       6.302  -2.613 -12.537  1.00 29.95           C
ATOM    105  OE1 GLU A  16       5.637  -2.398 -13.596  1.00 28.27           O
ATOM    106  OE2 GLU A  16       7.444  -3.137 -12.531  1.00 28.17           O1-
ATOM    107  N   GLU A  17       4.596  -0.699  -8.451  1.00 30.38           N
ATOM    108  CA  GLU A  17       5.444   0.099  -7.520  1.00 30.91           C
ATOM    109  C   GLU A  17       5.306  -0.525  -6.158  1.00 32.10           C
ATOM    110  O   GLU A  17       6.187  -1.268  -5.730  1.00 34.11           O
ATOM    111  CB  GLU A  17       5.044   1.589  -7.404  1.00 28.23           C
ATOM    112  CG  GLU A  17       6.165   2.451  -6.706  1.00 30.17           C
ATOM    113  CD  GLU A  17       5.688   3.762  -5.967  1.00 32.12           C
ATOM    114  OE1 GLU A  17       5.082   4.666  -6.598  1.00 30.67           O
ATOM    115  OE2 GLU A  17       6.027   3.939  -4.760  1.00 31.49           O1-
ATOM    116  N   ASN A  18       4.054  -0.563  -5.720  1.00 33.09           N
ATOM    117  CA  ASN A  18       3.696  -1.121  -4.420  1.00 32.50           C
ATOM    118  C   ASN A  18       3.913  -2.668  -4.325  1.00 31.11           C
ATOM    119  O   ASN A  18       3.947  -3.231  -3.212  1.00 30.55           O
ATOM    120  CB  ASN A  18       2.249  -0.690  -4.057  1.00 31.08           C
ATOM    121  CG  ASN A  18       2.086   0.851  -3.988  1.00 28.14           C
ATOM    122  ND2 ASN A  18       0.918   1.344  -4.361  1.00 28.12           N
ATOM    123  OD1 ASN A  18       3.018   1.572  -3.648  1.00 24.14           O
ATOM    124  N   PHE A  19       4.287  -3.307  -5.437  1.00 27.36           N
ATOM    125  CA  PHE A  19       4.761  -4.691  -5.361  1.00 25.30           C
ATOM    126  C   PHE A  19       6.240  -4.636  -5.071  1.00 25.50           C
ATOM    127  O   PHE A  19       6.703  -5.188  -4.078  1.00 26.44           O
ATOM    128  CB  PHE A  19       4.513  -5.455  -6.667  1.00 24.34           C
ATOM    129  CG  PHE A  19       4.448  -6.959  -6.504  1.00 21.73           C
ATOM    130  CD1 PHE A  19       5.594  -7.720  -6.488  1.00 21.65           C
ATOM    131  CD2 PHE A  19       3.236  -7.616  -6.507  1.00 21.52           C
ATOM    132  CE1 PHE A  19       5.532  -9.106  -6.486  1.00 18.58           C
ATOM    133  CE2 PHE A  19       3.175  -8.997  -6.508  1.00 20.40           C
ATOM    134  CZ  PHE A  19       4.328  -9.738  -6.499  1.00 18.31           C
ATOM    135  N   LYS A  20       6.957  -3.831  -5.839  1.00 24.36           N
ATOM    136  CA  LYS A  20       8.340  -3.545  -5.501  1.00 26.07           C
ATOM    137  C   LYS A  20       8.335  -3.055  -4.053  1.00 26.27           C
ATOM    138  O   LYS A  20       9.090  -3.567  -3.221  1.00 25.42           O
ATOM    139  CB  LYS A  20       8.921  -2.458  -6.432  1.00 27.79           C
```

ATOM	140	CG	LYS	A	20	9.948	-2.952	-7.490	1.00	26.76	C
ATOM	141	CD	LYS	A	20	9.952	-2.041	-8.743	1.00	22.31	C
ATOM	142	CE	LYS	A	20	10.898	-2.548	-9.824	1.00	20.10	C
ATOM	143	NZ	LYS	A	20	12.337	-2.566	-9.387	1.00	19.71	N1+
ATOM	144	N	ALA	A	21	7.287	-2.301	-3.711	1.00	26.57	N
ATOM	145	CA	ALA	A	21	7.264	-1.504	-2.480	1.00	25.54	C
ATOM	146	C	ALA	A	21	7.045	-2.381	-1.289	1.00	23.24	C
ATOM	147	O	ALA	A	21	7.661	-2.178	-0.261	1.00	24.58	O
ATOM	148	CB	ALA	A	21	6.188	-0.411	-2.539	1.00	24.10	C
ATOM	149	N	LEU	A	22	6.251	-3.422	-1.444	1.00	22.80	N
ATOM	150	CA	LEU	A	22	6.151	-4.371	-0.353	1.00	23.66	C
ATOM	151	C	LEU	A	22	7.414	-5.226	-0.300	1.00	22.00	C
ATOM	152	O	LEU	A	22	8.277	-4.987	0.543	1.00	22.37	O
ATOM	153	CB	LEU	A	22	4.892	-5.242	-0.489	1.00	25.44	C
ATOM	154	CG	LEU	A	22	3.648	-4.849	0.324	1.00	23.88	C
ATOM	155	CD1	LEU	A	22	4.053	-4.132	1.586	1.00	21.55	C
ATOM	156	CD2	LEU	A	22	2.740	-3.964	-0.500	1.00	23.25	C
ATOM	157	N	VAL	A	23	7.649	-5.965	-1.382	1.00	19.37	N
ATOM	158	CA	VAL	A	23	8.590	-7.075	-1.410	1.00	16.76	C
ATOM	159	C	VAL	A	23	10.018	-6.698	-0.945	1.00	18.40	C
ATOM	160	O	VAL	A	23	10.734	-7.519	-0.372	1.00	17.50	O
ATOM	161	CB	VAL	A	23	8.579	-7.698	-2.823	1.00	15.62	C
ATOM	162	CG1	VAL	A	23	9.429	-8.950	-2.878	1.00	13.90	C
ATOM	163	CG2	VAL	A	23	7.157	-8.040	-3.211	1.00	9.89	C
ATOM	164	N	LEU	A	24	10.336	-5.412	-0.999	1.00	20.80	N
ATOM	165	CA	LEU	A	24	11.503	-4.879	-0.319	1.00	21.23	C
ATOM	166	C	LEU	A	24	11.278	-4.949	1.182	1.00	22.45	C
ATOM	167	O	LEU	A	24	11.885	-5.812	1.826	1.00	25.32	O
ATOM	168	CB	LEU	A	24	11.767	-3.434	-0.729	1.00	23.94	C
ATOM	169	CG	LEU	A	24	13.205	-2.910	-0.666	1.00	25.01	C
ATOM	170	CD1	LEU	A	24	13.154	-1.397	-0.414	1.00	24.47	C
ATOM	171	CD2	LEU	A	24	14.017	-3.618	0.417	1.00	24.82	C
ATOM	172	N	ILE	A	25	10.410	-4.100	1.756	1.00	21.80	N
ATOM	173	CA	ILE	A	25	10.241	-4.122	3.238	1.00	22.89	C
ATOM	174	C	ILE	A	25	10.125	-5.571	3.747	1.00	23.14	C
ATOM	175	O	ILE	A	25	10.935	-5.997	4.564	1.00	20.48	O
ATOM	176	CB	ILE	A	25	8.993	-3.319	3.785	1.00	19.69	C
ATOM	177	CG1	ILE	A	25	7.714	-3.751	3.081	1.00	18.78	C
ATOM	178	CG2	ILE	A	25	9.220	-1.841	3.691	1.00	14.48	C
ATOM	179	CD1	ILE	A	25	6.793	-2.619	2.745	1.00	19.12	C
ATOM	180	N	ALA	A	26	9.289	-6.364	3.071	1.00	23.61	N
ATOM	181	CA	ALA	A	26	9.182	-7.799	3.320	1.00	24.15	C
ATOM	182	C	ALA	A	26	10.563	-8.405	3.520	1.00	24.53	C
ATOM	183	O	ALA	A	26	10.954	-8.689	4.649	1.00	24.25	O
ATOM	184	CB	ALA	A	26	8.475	-8.487	2.149	1.00	23.75	C
ATOM	185	N	PHE	A	27	11.367	-8.391	2.458	1.00	25.34	N
ATOM	186	CA	PHE	A	27	12.700	-8.999	2.472	1.00	23.54	C
ATOM	187	C	PHE	A	27	13.657	-8.301	3.432	1.00	21.84	C
ATOM	188	O	PHE	A	27	14.755	-8.811	3.685	1.00	21.92	O
ATOM	189	CB	PHE	A	27	13.312	-8.985	1.067	1.00	22.66	C
ATOM	190	CG	PHE	A	27	12.781	-10.058	0.157	1.00	21.80	C
ATOM	191	CD1	PHE	A	27	12.742	-11.375	0.562	1.00	22.31	C
ATOM	192	CD2	PHE	A	27	12.383	-9.753	-1.130	1.00	19.87	C
ATOM	193	CE1	PHE	A	27	12.309	-12.365	-0.311	1.00	23.11	C
ATOM	194	CE2	PHE	A	27	11.955	-10.733	-1.999	1.00	17.81	C
ATOM	195	CZ	PHE	A	27	11.914	-12.034	-1.595	1.00	20.05	C
ATOM	196	N	ALA	A	28	13.258	-7.145	3.956	1.00	19.72	N
ATOM	197	CA	ALA	A	28	14.162	-6.345	4.775	1.00	20.10	C
ATOM	198	C	ALA	A	28	13.699	-5.958	6.201	1.00	21.53	C
ATOM	199	O	ALA	A	28	14.345	-5.141	6.856	1.00	21.18	O
ATOM	200	CB	ALA	A	28	14.589	-5.111	4.002	1.00	19.50	C
ATOM	201	N	GLN	A	29	12.557	-6.484	6.644	1.00	22.14	N
ATOM	202	CA	GLN	A	29	12.293	-6.695	8.068	1.00	19.68	C
ATOM	203	C	GLN	A	29	12.975	-8.025	8.333	1.00	22.08	C
ATOM	204	O	GLN	A	29	13.984	-8.084	9.029	1.00	21.67	O
ATOM	205	CB	GLN	A	29	10.785	-6.840	8.336	1.00	15.49	C
ATOM	206	CG	GLN	A	29	9.953	-5.582	8.068	1.00	16.39	C
ATOM	207	CD	GLN	A	29	8.445	-5.805	8.202	1.00	17.09	C
ATOM	208	NE2	GLN	A	29	7.699	-4.724	8.411	1.00	12.64	N
ATOM	209	OE1	GLN	A	29	7.959	-6.934	8.092	1.00	19.84	O
ATOM	210	N	TYR	A	30	12.622	-8.974	7.462	1.00	24.94	N

ATOM	211	CA	TYR	A	30	13.071	-10.369	7.483	1.00	23.92	C
ATOM	212	C	TYR	A	30	14.537	-10.604	7.852	1.00	24.37	C
ATOM	213	O	TYR	A	30	14.813	-11.198	8.911	1.00	26.54	O
ATOM	214	CB	TYR	A	30	12.785	-11.008	6.129	1.00	22.66	C
ATOM	215	CG	TYR	A	30	11.456	-11.702	6.058	1.00	24.59	C
ATOM	216	CD1	TYR	A	30	11.236	-12.867	6.775	1.00	26.93	C
ATOM	217	CD2	TYR	A	30	10.448	-11.251	5.218	1.00	25.02	C
ATOM	218	CE1	TYR	A	30	10.052	-13.584	6.651	1.00	27.16	C
ATOM	219	CE2	TYR	A	30	9.253	-11.953	5.082	1.00	26.54	C
ATOM	220	CZ	TYR	A	30	9.068	-13.128	5.798	1.00	27.87	C
ATOM	221	OH	TYR	A	30	7.931	-13.888	5.629	1.00	28.25	O
ATOM	222	N	LEU	A	31	15.469	-10.188	6.982	1.00	19.65	N
ATOM	223	CA	LEU	A	31	16.893	-10.427	7.227	1.00	16.82	C
ATOM	224	C	LEU	A	31	17.698	-9.262	7.838	1.00	16.70	C
ATOM	225	O	LEU	A	31	18.814	-9.467	8.332	1.00	16.93	O
ATOM	226	CB	LEU	A	31	17.569	-10.920	5.949	1.00	18.70	C
ATOM	227	CG	LEU	A	31	17.564	-12.423	5.628	1.00	18.34	C
ATOM	228	CD1	LEU	A	31	18.751	-12.798	4.728	1.00	16.70	C
ATOM	229	CD2	LEU	A	31	17.629	-13.188	6.913	1.00	17.85	C
ATOM	230	N	GLN	A	32	17.196	-8.040	7.703	1.00	12.99	N
ATOM	231	CA	GLN	A	32	17.563	-6.922	8.579	1.00	15.39	C
ATOM	232	C	GLN	A	32	19.015	-6.620	9.024	1.00	16.50	C
ATOM	233	O	GLN	A	32	19.233	-5.706	9.832	1.00	16.68	O
ATOM	234	CB	GLN	A	32	16.647	-6.914	9.830	1.00	17.32	C
ATOM	235	CG	GLN	A	32	16.825	-8.056	10.882	1.00	18.77	C
ATOM	236	CD	GLN	A	32	16.438	-7.610	12.302	1.00	19.47	C
ATOM	237	NE2	GLN	A	32	17.197	-8.054	13.301	1.00	11.37	N
ATOM	238	OE1	GLN	A	32	15.564	-6.759	12.468	1.00	21.08	O
ATOM	239	N	GLN	A	33	20.018	-7.295	8.474	1.00	17.10	N
ATOM	240	CA	GLN	A	33	21.399	-6.839	8.685	1.00	18.29	C
ATOM	241	C	GLN	A	33	22.190	-6.783	7.353	1.00	19.96	C
ATOM	242	O	GLN	A	33	22.912	-5.816	7.095	1.00	20.35	O
ATOM	243	CB	GLN	A	33	22.119	-7.705	9.752	1.00	16.98	C
ATOM	244	CG	GLN	A	33	21.184	-8.265	10.879	1.00	21.56	C
ATOM	245	CD	GLN	A	33	21.643	-7.989	12.350	1.00	23.80	C
ATOM	246	NE2	GLN	A	33	22.932	-7.685	12.533	1.00	26.50	N
ATOM	247	OE1	GLN	A	33	20.834	-8.040	13.295	1.00	19.44	O
ATOM	248	N	CYS	A	34	21.943	-7.745	6.458	1.00	20.58	N
ATOM	249	CA	CYS	A	34	22.517	-7.742	5.113	1.00	18.89	C
ATOM	250	C	CYS	A	34	22.254	-6.421	4.403	1.00	20.26	C
ATOM	251	O	CYS	A	34	21.094	-5.970	4.313	1.00	18.49	O
ATOM	252	CB	CYS	A	34	21.906	-8.857	4.277	1.00	17.31	C
ATOM	253	SG	CYS	A	34	22.155	-10.502	4.964	1.00	28.19	S
ATOM	254	N	PRO	A	35	23.309	-5.836	3.795	1.00	19.86	N
ATOM	255	CA	PRO	A	35	23.265	-4.593	3.019	1.00	16.08	C
ATOM	256	C	PRO	A	35	22.189	-4.451	1.981	1.00	13.85	C
ATOM	257	O	PRO	A	35	21.368	-5.324	1.779	1.00	11.96	O
ATOM	258	CB	PRO	A	35	24.648	-4.512	2.420	1.00	14.75	C
ATOM	259	CG	PRO	A	35	25.491	-4.991	3.508	1.00	21.60	C
ATOM	260	CD	PRO	A	35	24.692	-6.116	4.215	1.00	21.49	C
ATOM	261	N	PHE	A	36	22.110	-3.257	1.430	1.00	16.85	N
ATOM	262	CA	PHE	A	36	20.988	-2.926	0.602	1.00	17.73	C
ATOM	263	C	PHE	A	36	20.944	-3.782	-0.646	1.00	20.98	C
ATOM	264	O	PHE	A	36	20.009	-4.576	-0.789	1.00	24.39	O
ATOM	265	CB	PHE	A	36	20.979	-1.443	0.233	1.00	18.53	C
ATOM	266	CG	PHE	A	36	19.674	-0.996	-0.359	1.00	19.96	C
ATOM	267	CD1	PHE	A	36	18.482	-1.376	0.223	1.00	18.68	C
ATOM	268	CD2	PHE	A	36	19.632	-0.366	-1.579	1.00	20.99	C
ATOM	269	CE1	PHE	A	36	17.308	-1.164	-0.391	1.00	17.22	C
ATOM	270	CE2	PHE	A	36	18.443	-0.149	-2.195	1.00	19.46	C
ATOM	271	CZ	PHE	A	36	17.282	-0.559	-1.600	1.00	19.20	C
ATOM	272	N	GLU	A	37	21.990	-3.721	-1.482	1.00	21.10	N
ATOM	273	CA	GLU	A	37	21.895	-4.190	-2.874	1.00	20.88	C
ATOM	274	C	GLU	A	37	21.529	-5.668	-3.024	1.00	20.33	C
ATOM	275	O	GLU	A	37	20.863	-6.060	-3.979	1.00	18.29	O
ATOM	276	CB	GLU	A	37	23.192	-3.914	-3.617	1.00	22.94	C
ATOM	277	CG	GLU	A	37	23.564	-2.453	-3.675	1.00	24.70	C
ATOM	278	CD	GLU	A	37	24.725	-2.103	-2.759	1.00	27.37	C
ATOM	279	OE1	GLU	A	37	25.368	-3.042	-2.215	1.00	26.97	O
ATOM	280	OE2	GLU	A	37	24.966	-0.880	-2.567	1.00	25.74	O1-
ATOM	281	N	ASP	A	38	22.115	-6.488	-2.161	1.00	20.03	N

```
ATOM    282  CA  ASP A  38      21.747  -7.891  -1.979  1.00 18.25           C
ATOM    283  C   ASP A  38      20.224  -8.028  -1.946  1.00 20.19           C
ATOM    284  O   ASP A  38      19.615  -8.679  -2.800  1.00 17.87           O
ATOM    285  CB  ASP A  38      22.348  -8.378  -0.663  1.00 16.94           C
ATOM    286  CG  ASP A  38      23.796  -7.902  -0.463  1.00 18.58           C
ATOM    287  OD1 ASP A  38      24.047  -6.683  -0.383  1.00 14.38           O
ATOM    288  OD2 ASP A  38      24.696  -8.758  -0.380  1.00 22.12           O1-
ATOM    289  N   HIS A  39      19.612  -7.271  -1.038  1.00 23.52           N
ATOM    290  CA  HIS A  39      18.160  -7.214  -0.882  1.00 22.04           C
ATOM    291  C   HIS A  39      17.507  -6.768  -2.173  1.00 20.54           C
ATOM    292  O   HIS A  39      16.482  -7.318  -2.558  1.00 21.75           O
ATOM    293  CB  HIS A  39      17.765  -6.228   0.235  1.00 22.52           C
ATOM    294  CG  HIS A  39      17.962  -6.765   1.616  1.00 21.90           C
ATOM    295  CD2 HIS A  39      17.572  -7.930   2.183  1.00 22.42           C
ATOM    296  ND1 HIS A  39      18.606  -6.054   2.609  1.00 23.78           N
ATOM    297  CE1 HIS A  39      18.596  -6.759   3.729  1.00 21.46           C
ATOM    298  NE2 HIS A  39      17.980  -7.900   3.492  1.00 22.35           N
ATOM    299  N   VAL A  40      18.052  -5.725  -2.797  1.00 17.74           N
ATOM    300  CA  VAL A  40      17.394  -5.125  -3.959  1.00 17.68           C
ATOM    301  C   VAL A  40      17.509  -5.956  -5.264  1.00 21.04           C
ATOM    302  O   VAL A  40      16.509  -6.136  -5.967  1.00 22.22           O
ATOM    303  CB  VAL A  40      17.863  -3.667  -4.225  1.00 15.09           C
ATOM    304  CG1 VAL A  40      16.681  -2.748  -4.220  1.00 15.66           C
ATOM    305  CG2 VAL A  40      18.816  -3.204  -3.185  1.00 12.61           C
ATOM    306  N   LYS A  41      18.664  -6.575  -5.518  1.00 21.82           N
ATOM    307  CA  LYS A  41      18.789  -7.519  -6.630  1.00 21.46           C
ATOM    308  C   LYS A  41      17.644  -8.486  -6.523  1.00 21.01           C
ATOM    309  O   LYS A  41      16.769  -8.480  -7.376  1.00 23.65           O
ATOM    310  CB  LYS A  41      20.108  -8.290  -6.546  1.00 25.68           C
ATOM    311  CG  LYS A  41      20.620  -8.833  -7.888  1.00 27.49           C
ATOM    312  CD  LYS A  41      22.165  -8.736  -8.020  1.00 23.95           C
ATOM    313  CE  LYS A  41      22.573  -8.660  -9.504  1.00 21.47           C
ATOM    314  NZ  LYS A  41      23.966  -8.207  -9.722  1.00 16.15           N1+
ATOM    315  N   LEU A  42      17.495  -9.053  -5.324  1.00 21.14           N
ATOM    316  CA  LEU A  42      16.451 -10.045  -5.020  1.00 20.21           C
ATOM    317  C   LEU A  42      15.021  -9.526  -5.193  1.00 18.80           C
ATOM    318  O   LEU A  42      14.119 -10.252  -5.621  1.00 14.06           O
ATOM    319  CB  LEU A  42      16.653 -10.582  -3.604  1.00 20.68           C
ATOM    320  CG  LEU A  42      18.057 -11.154  -3.410  1.00 23.93           C
ATOM    321  CD1 LEU A  42      18.337 -11.363  -1.954  1.00 26.48           C
ATOM    322  CD2 LEU A  42      18.195 -12.468  -4.155  1.00 27.94           C
ATOM    323  N   VAL A  43      14.828  -8.248  -4.920  1.00 20.91           N
ATOM    324  CA  VAL A  43      13.557  -7.646  -5.243  1.00 25.09           C
ATOM    325  C   VAL A  43      13.455  -7.618  -6.756  1.00 24.80           C
ATOM    326  O   VAL A  43      12.655  -8.371  -7.319  1.00 24.61           O
ATOM    327  CB  VAL A  43      13.424  -6.220  -4.671  1.00 29.14           C
ATOM    328  CG1 VAL A  43      12.078  -5.628  -5.086  1.00 29.20           C
ATOM    329  CG2 VAL A  43      13.555  -6.252  -3.122  1.00 29.89           C
ATOM    330  N   ASN A  44      14.422  -6.952  -7.393  1.00 24.28           N
ATOM    331  CA  ASN A  44      14.472  -6.794  -8.849  1.00 24.67           C
ATOM    332  C   ASN A  44      13.983  -8.073  -9.527  1.00 25.13           C
ATOM    333  O   ASN A  44      13.160  -8.021 -10.443  1.00 27.80           O
ATOM    334  CB  ASN A  44      15.908  -6.461  -9.325  1.00 25.32           C
ATOM    335  CG  ASN A  44      16.357  -5.040  -8.947  1.00 29.48           C
ATOM    336  ND2 ASN A  44      15.803  -4.049  -9.631  1.00 31.83           N
ATOM    337  OD1 ASN A  44      17.192  -4.838  -8.052  1.00 30.17           O
ATOM    338  N   GLU A  45      14.326  -9.217  -8.942  1.00 24.11           N
ATOM    339  CA  GLU A  45      14.019 -10.503  -9.553  1.00 25.12           C
ATOM    340  C   GLU A  45      12.569 -10.992  -9.356  1.00 26.68           C
ATOM    341  O   GLU A  45      11.931 -11.464 -10.315  1.00 26.81           O
ATOM    342  CB  GLU A  45      15.015 -11.547  -9.043  1.00 22.72           C
ATOM    343  CG  GLU A  45      16.351 -11.537  -9.773  1.00 16.42           C
ATOM    344  CD  GLU A  45      17.486 -11.808  -8.848  1.00 12.41           C
ATOM    345  OE1 GLU A  45      17.454 -12.854  -8.182  1.00  8.42           O
ATOM    346  OE2 GLU A  45      18.353 -10.928  -8.705  1.00 12.61           O1-
ATOM    347  N   VAL A  46      12.105 -10.980  -8.100  1.00 28.78           N
ATOM    348  CA  VAL A  46      10.677 -11.141  -7.758  1.00 27.45           C
ATOM    349  C   VAL A  46       9.849 -10.270  -8.670  1.00 25.30           C
ATOM    350  O   VAL A  46       8.937 -10.754  -9.344  1.00 24.88           O
ATOM    351  CB  VAL A  46      10.352 -10.637  -6.332  1.00 28.88           C
ATOM    352  CG1 VAL A  46       8.928 -11.039  -5.958  1.00 28.73           C
```

ATOM	353	CG2	VAL	A	46	11.381	-11.152	-5.327	1.00	30.37	C
ATOM	354	N	THR	A	47	10.115	-8.966	-8.588	1.00	22.75	N
ATOM	355	CA	THR	A	47	9.506	-7.984	-9.460	1.00	21.85	C
ATOM	356	C	THR	A	47	9.418	-8.500	-10.909	1.00	24.34	C
ATOM	357	O	THR	A	47	8.328	-8.429	-11.517	1.00	23.89	O
ATOM	358	CB	THR	A	47	10.293	-6.653	-9.427	1.00	20.39	C
ATOM	359	CG2	THR	A	47	9.430	-5.527	-9.894	1.00	22.88	C
ATOM	360	OG1	THR	A	47	10.696	-6.351	-8.090	1.00	18.42	O
ATOM	361	N	GLU	A	48	10.502	-9.131	-11.402	1.00	24.26	N
ATOM	362	CA	GLU	A	48	10.540	-9.677	-12.778	1.00	25.92	C
ATOM	363	C	GLU	A	48	9.432	-10.732	-13.024	1.00	26.47	C
ATOM	364	O	GLU	A	48	8.254	-10.386	-13.267	1.00	21.91	O
ATOM	365	CB	GLU	A	48	11.920	-10.285	-13.101	1.00	25.59	C
ATOM	366	CG	GLU	A	48	12.994	-9.275	-13.584	1.00	34.29	C
ATOM	367	CD	GLU	A	48	13.681	-9.665	-14.925	1.00	37.71	C
ATOM	368	OE1	GLU	A	48	14.662	-10.459	-14.916	1.00	37.81	O
ATOM	369	OE2	GLU	A	48	13.290	-9.109	-15.986	1.00	39.96	O1-
ATOM	370	N	PHE	A	49	9.865	-11.982	-13.195	1.00	26.44	N
ATOM	371	CA	PHE	A	49	9.041	-13.162	-12.909	1.00	26.68	C
ATOM	372	C	PHE	A	49	7.528	-12.915	-12.813	1.00	27.60	C
ATOM	373	O	PHE	A	49	6.732	-13.630	-13.444	1.00	27.93	O
ATOM	374	CB	PHE	A	49	9.534	-13.796	-11.611	1.00	23.99	C
ATOM	375	CG	PHE	A	49	9.256	-15.241	-11.510	1.00	22.41	C
ATOM	376	CD1	PHE	A	49	8.042	-15.760	-11.905	1.00	25.30	C
ATOM	377	CD2	PHE	A	49	10.177	-16.079	-10.948	1.00	23.15	C
ATOM	378	CE1	PHE	A	49	7.754	-17.099	-11.725	1.00	26.44	C
ATOM	379	CE2	PHE	A	49	9.893	-17.410	-10.766	1.00	26.07	C
ATOM	380	CZ	PHE	A	49	8.682	-17.917	-11.151	1.00	23.64	C
ATOM	381	N	ALA	A	50	7.147	-12.051	-11.869	1.00	28.68	N
ATOM	382	CA	ALA	A	50	5.757	-11.693	-11.611	1.00	26.29	C
ATOM	383	C	ALA	A	50	5.093	-11.370	-12.939	1.00	26.64	C
ATOM	384	O	ALA	A	50	4.068	-11.963	-13.302	1.00	25.34	O
ATOM	385	CB	ALA	A	50	5.717	-10.487	-10.704	1.00	22.98	C
ATOM	386	N	LYS	A	51	5.773	-10.515	-13.703	1.00	26.55	N
ATOM	387	CA	LYS	A	51	5.334	-10.065	-15.024	1.00	24.67	C
ATOM	388	C	LYS	A	51	5.138	-11.225	-15.983	1.00	23.19	C
ATOM	389	O	LYS	A	51	4.298	-11.140	-16.871	1.00	22.00	O
ATOM	390	CB	LYS	A	51	6.366	-9.099	-15.636	1.00	25.82	C
ATOM	391	CG	LYS	A	51	6.392	-7.692	-15.057	1.00	20.83	C
ATOM	392	CD	LYS	A	51	7.753	-7.062	-15.237	1.00	15.07	C
ATOM	393	CE	LYS	A	51	7.799	-5.778	-14.475	1.00	17.03	C
ATOM	394	NZ	LYS	A	51	7.346	-5.991	-13.051	1.00	19.85	N1+
ATOM	395	N	THR	A	52	5.942	-12.276	-15.844	1.00	20.21	N
ATOM	396	CA	THR	A	52	5.860	-13.406	-16.750	1.00	20.84	C
ATOM	397	C	THR	A	52	4.763	-14.438	-16.407	1.00	23.73	C
ATOM	398	O	THR	A	52	4.255	-15.121	-17.310	1.00	25.44	O
ATOM	399	CB	THR	A	52	7.233	-14.084	-16.902	1.00	18.84	C
ATOM	400	CG2	THR	A	52	7.234	-15.510	-16.319	1.00	15.42	C
ATOM	401	OG1	THR	A	52	7.585	-14.107	-18.292	1.00	18.38	O
ATOM	402	N	CYS	A	53	4.305	-14.468	-15.154	1.00	24.15	N
ATOM	403	CA	CYS	A	53	3.020	-15.113	-14.852	1.00	23.39	C
ATOM	404	C	CYS	A	53	1.841	-14.134	-15.054	1.00	21.30	C
ATOM	405	O	CYS	A	53	0.704	-14.552	-15.268	1.00	21.30	O
ATOM	406	CB	CYS	A	53	3.000	-15.710	-13.428	1.00	24.03	C
ATOM	407	SG	CYS	A	53	4.336	-16.896	-13.023	1.00	22.23	S
ATOM	408	N	VAL	A	54	2.135	-12.843	-15.077	1.00	19.83	N
ATOM	409	CA	VAL	A	54	1.222	-11.856	-15.647	1.00	23.85	C
ATOM	410	C	VAL	A	54	0.895	-12.233	-17.099	1.00	24.27	C
ATOM	411	O	VAL	A	54	-0.282	-12.282	-17.504	1.00	24.50	O
ATOM	412	CB	VAL	A	54	1.865	-10.426	-15.619	1.00	26.97	C
ATOM	413	CG1	VAL	A	54	1.309	-9.538	-16.746	1.00	26.12	C
ATOM	414	CG2	VAL	A	54	1.638	-9.771	-14.250	1.00	30.36	C
ATOM	415	N	ALA	A	55	1.952	-12.457	-17.881	1.00	22.66	N
ATOM	416	CA	ALA	A	55	1.845	-12.830	-19.291	1.00	20.65	C
ATOM	417	C	ALA	A	55	0.971	-14.066	-19.412	1.00	21.32	C
ATOM	418	O	ALA	A	55	0.002	-14.111	-20.189	1.00	20.09	O
ATOM	419	CB	ALA	A	55	3.235	-13.120	-19.850	1.00	18.03	C
ATOM	420	N	ASP	A	56	1.236	-14.994	-18.498	1.00	22.03	N
ATOM	421	CA	ASP	A	56	0.765	-16.364	-18.591	1.00	20.78	C
ATOM	422	C	ASP	A	56	0.823	-16.974	-17.200	1.00	17.21	C
ATOM	423	O	ASP	A	56	1.892	-17.299	-16.698	1.00	13.60	O

```
ATOM    424  CB  ASP A  56       1.667 -17.168 -19.555  1.00 21.11           C
ATOM    425  CG  ASP A  56       0.882 -17.885 -20.641  1.00 20.85           C
ATOM    426  OD1 ASP A  56      -0.136 -17.326 -21.131  1.00 18.50           O
ATOM    427  OD2 ASP A  56       1.283 -19.021 -20.979  1.00 19.93           O1-
ATOM    428  N   GLU A  57      -0.326 -17.055 -16.554  1.00 17.39           N
ATOM    429  CA  GLU A  57      -0.486 -17.952 -15.418  1.00 19.41           C
ATOM    430  C   GLU A  57       0.281 -19.246 -15.683  1.00 19.69           C
ATOM    431  O   GLU A  57       1.357 -19.437 -15.115  1.00 18.89           O
ATOM    432  CB  GLU A  57      -1.968 -18.291 -15.198  1.00 21.01           C
ATOM    433  CG  GLU A  57      -2.926 -17.181 -15.550  1.00 18.07           C
ATOM    434  CD  GLU A  57      -3.521 -16.521 -14.336  1.00 21.55           C
ATOM    435  OE1 GLU A  57      -2.782 -15.874 -13.551  1.00 18.91           O
ATOM    436  OE2 GLU A  57      -4.756 -16.620 -14.200  1.00 23.50           O1-
ATOM    437  N   SER A  58      -0.138 -19.944 -16.752  1.00 20.54           N
ATOM    438  CA  SER A  58       0.233 -21.343 -17.074  1.00 18.77           C
ATOM    439  C   SER A  58       1.734 -21.685 -17.200  1.00 16.17           C
ATOM    440  O   SER A  58       2.107 -22.848 -17.305  1.00 12.41           O
ATOM    441  CB  SER A  58      -0.468 -21.771 -18.367  1.00 19.91           C
ATOM    442  OG  SER A  58      -1.586 -20.943 -18.670  1.00 22.34           O
ATOM    443  N   ALA A  59       2.580 -20.666 -17.191  1.00 16.30           N
ATOM    444  CA  ALA A  59       4.000 -20.840 -17.363  1.00 19.02           C
ATOM    445  C   ALA A  59       4.585 -21.590 -16.177  1.00 21.95           C
ATOM    446  O   ALA A  59       4.252 -21.262 -15.033  1.00 23.97           O
ATOM    447  CB  ALA A  59       4.640 -19.489 -17.474  1.00 22.09           C
ATOM    448  N   GLU A  60       5.602 -22.419 -16.441  1.00 22.59           N
ATOM    449  CA  GLU A  60       6.267 -23.242 -15.417  1.00 22.93           C
ATOM    450  C   GLU A  60       6.511 -22.498 -14.113  1.00 24.76           C
ATOM    451  O   GLU A  60       6.901 -21.338 -14.122  1.00 28.75           O
ATOM    452  CB  GLU A  60       7.611 -23.778 -15.934  1.00 19.81           C
ATOM    453  CG  GLU A  60       8.417 -24.596 -14.918  1.00 16.82           C
ATOM    454  CD  GLU A  60       9.732 -25.160 -15.474  1.00 17.21           C
ATOM    455  OE1 GLU A  60      10.733 -24.414 -15.489  1.00 14.77           O
ATOM    456  OE2 GLU A  60       9.794 -26.376 -15.792  1.00 16.15           O1-
ATOM    457  N   ASN A  61       6.248 -23.175 -13.002  1.00 25.15           N
ATOM    458  CA  ASN A  61       6.546 -22.712 -11.638  1.00 26.26           C
ATOM    459  C   ASN A  61       5.873 -21.448 -11.121  1.00 26.29           C
ATOM    460  O   ASN A  61       6.007 -21.124  -9.945  1.00 26.16           O
ATOM    461  CB  ASN A  61       8.054 -22.619 -11.376  1.00 25.05           C
ATOM    462  CG  ASN A  61       8.730 -21.612 -12.252  1.00 25.32           C
ATOM    463  ND2 ASN A  61       8.313 -20.363 -12.155  1.00 21.35           N
ATOM    464  OD1 ASN A  61       9.503 -21.985 -13.129  1.00 26.74           O
ATOM    465  N   CYS A  62       5.005 -20.840 -11.915  1.00 27.22           N
ATOM    466  CA  CYS A  62       4.054 -19.901 -11.338  1.00 27.20           C
ATOM    467  C   CYS A  62       3.419 -20.547 -10.106  1.00 27.53           C
ATOM    468  O   CYS A  62       3.560 -20.010  -9.012  1.00 27.96           O
ATOM    469  CB  CYS A  62       3.001 -19.538 -12.379  1.00 27.41           C
ATOM    470  SG  CYS A  62       3.731 -18.673 -13.817  1.00 27.27           S
ATOM    471  N   ASP A  63       3.166 -21.853 -10.247  1.00 29.32           N
ATOM    472  CA  ASP A  63       2.519 -22.729  -9.259  1.00 29.88           C
ATOM    473  C   ASP A  63       3.127 -22.814  -7.853  1.00 31.82           C
ATOM    474  O   ASP A  63       2.702 -23.660  -7.046  1.00 30.96           O
ATOM    475  CB  ASP A  63       2.437 -24.142  -9.824  1.00 29.57           C
ATOM    476  CG  ASP A  63       1.214 -24.355 -10.687  1.00 31.17           C
ATOM    477  OD1 ASP A  63       1.082 -23.702 -11.758  1.00 32.77           O
ATOM    478  OD2 ASP A  63       0.381 -25.200 -10.295  1.00 32.47           O1-
ATOM    479  N   LYS A  64       4.183 -22.033  -7.600  1.00 33.23           N
ATOM    480  CA  LYS A  64       4.695 -21.785  -6.244  1.00 30.18           C
ATOM    481  C   LYS A  64       3.561 -21.200  -5.372  1.00 29.80           C
ATOM    482  O   LYS A  64       2.803 -20.316  -5.817  1.00 28.83           O
ATOM    483  CB  LYS A  64       5.864 -20.782  -6.308  1.00 27.37           C
ATOM    484  CG  LYS A  64       7.257 -21.397  -6.361  1.00 25.95           C
ATOM    485  CD  LYS A  64       7.352 -22.527  -7.386  1.00 27.90           C
ATOM    486  CE  LYS A  64       8.191 -23.714  -6.889  1.00 28.98           C
ATOM    487  NZ  LYS A  64       9.640 -23.392  -6.604  1.00 27.67           N1+
ATOM    488  N   SER A  65       3.474 -21.647  -4.120  1.00 28.75           N
ATOM    489  CA  SER A  65       2.650 -20.934  -3.136  1.00 27.25           C
ATOM    490  C   SER A  65       3.419 -19.725  -2.546  1.00 25.40           C
ATOM    491  O   SER A  65       4.196 -19.869  -1.583  1.00 21.94           O
ATOM    492  CB  SER A  65       2.188 -21.897  -2.019  1.00 28.94           C
ATOM    493  OG  SER A  65       0.824 -22.307  -2.198  1.00 26.21           O
ATOM    494  N   LEU A  66       3.329 -18.597  -3.256  1.00 21.69           N
```

ATOM	495	CA	LEU	A	66	3.971	-17.330	-2.891	1.00	18.29	C
ATOM	496	C	LEU	A	66	5.229	-17.507	-2.069	1.00	20.23	C
ATOM	497	O	LEU	A	66	6.283	-17.683	-2.652	1.00	23.75	O
ATOM	498	CB	LEU	A	66	2.982	-16.401	-2.180	1.00	12.64	C
ATOM	499	CG	LEU	A	66	3.160	-14.880	-2.253	1.00	5.07	C
ATOM	500	CD1	LEU	A	66	4.592	-14.537	-2.380	1.00	2.40	C
ATOM	501	CD2	LEU	A	66	2.384	-14.296	-3.399	1.00	2.17	C
ATOM	502	N	HIS	A	67	5.083	-17.646	-0.749	1.00	23.72	N
ATOM	503	CA	HIS	A	67	6.208	-17.826	0.192	1.00	25.77	C
ATOM	504	C	HIS	A	67	7.266	-18.712	-0.428	1.00	27.03	C
ATOM	505	O	HIS	A	67	8.407	-18.292	-0.612	1.00	28.17	O
ATOM	506	CB	HIS	A	67	5.757	-18.505	1.505	1.00	27.30	C
ATOM	507	CG	HIS	A	67	5.053	-17.596	2.474	1.00	28.03	C
ATOM	508	CD2	HIS	A	67	5.372	-16.363	2.940	1.00	25.15	C
ATOM	509	ND1	HIS	A	67	3.955	-18.009	3.206	1.00	25.30	N
ATOM	510	CE1	HIS	A	67	3.645	-17.076	4.091	1.00	24.34	C
ATOM	511	NE2	HIS	A	67	4.488	-16.070	3.947	1.00	23.45	N
ATOM	512	N	THR	A	68	6.833	-19.877	-0.895	1.00	28.20	N
ATOM	513	CA	THR	A	68	7.749	-20.853	-1.467	1.00	31.25	C
ATOM	514	C	THR	A	68	8.754	-20.271	-2.461	1.00	32.00	C
ATOM	515	O	THR	A	68	9.905	-20.750	-2.549	1.00	31.36	O
ATOM	516	CB	THR	A	68	6.986	-21.972	-2.142	1.00	32.39	C
ATOM	517	CG2	THR	A	68	7.624	-23.327	-1.793	1.00	31.68	C
ATOM	518	OG1	THR	A	68	5.617	-21.933	-1.697	1.00	35.75	O
ATOM	519	N	LEU	A	69	8.353	-19.227	-3.185	1.00	31.87	N
ATOM	520	CA	LEU	A	69	9.340	-18.500	-3.974	1.00	33.65	C
ATOM	521	C	LEU	A	69	10.314	-17.880	-2.982	1.00	32.74	C
ATOM	522	O	LEU	A	69	11.288	-18.548	-2.631	1.00	32.06	O
ATOM	523	CB	LEU	A	69	8.705	-17.444	-4.905	1.00	34.67	C
ATOM	524	CG	LEU	A	69	9.426	-17.171	-6.259	1.00	34.79	C
ATOM	525	CD1	LEU	A	69	10.395	-15.967	-6.148	1.00	32.65	C
ATOM	526	CD2	LEU	A	69	10.174	-18.427	-6.738	1.00	31.64	C
ATOM	527	N	PHE	A	70	9.943	-16.747	-2.374	1.00	30.96	N
ATOM	528	CA	PHE	A	70	10.788	-16.080	-1.365	1.00	30.54	C
ATOM	529	C	PHE	A	70	11.691	-17.099	-0.646	1.00	29.74	C
ATOM	530	O	PHE	A	70	12.906	-17.097	-0.807	1.00	26.41	O
ATOM	531	CB	PHE	A	70	9.932	-15.387	-0.288	1.00	30.25	C
ATOM	532	CG	PHE	A	70	9.227	-14.125	-0.737	1.00	28.46	C
ATOM	533	CD1	PHE	A	70	8.468	-14.109	-1.899	1.00	26.39	C
ATOM	534	CD2	PHE	A	70	9.075	-13.072	0.168	1.00	24.40	C
ATOM	535	CE1	PHE	A	70	7.554	-13.084	-2.130	1.00	27.08	C
ATOM	536	CE2	PHE	A	70	8.165	-12.058	-0.054	1.00	21.72	C
ATOM	537	CZ	PHE	A	70	7.399	-12.062	-1.196	1.00	25.72	C
ATOM	538	N	GLY	A	71	11.043	-18.122	-0.104	1.00	31.24	N
ATOM	539	CA	GLY	A	71	11.712	-19.075	0.764	1.00	31.89	C
ATOM	540	C	GLY	A	71	12.873	-19.697	0.044	1.00	31.60	C
ATOM	541	O	GLY	A	71	14.004	-19.640	0.536	1.00	31.27	O
ATOM	542	N	ASP	A	72	12.614	-19.987	-1.227	1.00	33.37	N
ATOM	543	CA	ASP	A	72	13.555	-20.627	-2.154	1.00	33.34	C
ATOM	544	C	ASP	A	72	14.353	-19.663	-3.083	1.00	32.34	C
ATOM	545	O	ASP	A	72	15.121	-20.113	-3.946	1.00	30.19	O
ATOM	546	CB	ASP	A	72	12.767	-21.662	-2.979	1.00	33.35	C
ATOM	547	CG	ASP	A	72	12.165	-22.762	-2.099	1.00	31.88	C
ATOM	548	OD1	ASP	A	72	12.805	-23.100	-1.077	1.00	30.99	O
ATOM	549	OD2	ASP	A	72	11.059	-23.269	-2.406	1.00	29.43	O1-
ATOM	550	N	LYS	A	73	14.183	-18.352	-2.870	1.00	31.11	N
ATOM	551	CA	LYS	A	73	14.958	-17.288	-3.538	1.00	27.64	C
ATOM	552	C	LYS	A	73	15.199	-16.145	-2.533	1.00	26.15	C
ATOM	553	O	LYS	A	73	14.585	-15.081	-2.619	1.00	23.20	O
ATOM	554	CB	LYS	A	73	14.187	-16.738	-4.757	1.00	28.01	C
ATOM	555	CG	LYS	A	73	14.083	-17.691	-5.962	1.00	25.72	C
ATOM	556	CD	LYS	A	73	14.918	-17.203	-7.143	1.00	24.18	C
ATOM	557	CE	LYS	A	73	14.754	-15.712	-7.383	1.00	23.68	C
ATOM	558	NZ	LYS	A	73	16.079	-15.048	-7.529	1.00	22.67	N1+
ATOM	559	N	LEU	A	74	16.031	-16.423	-1.533	1.00	27.33	N
ATOM	560	CA	LEU	A	74	16.166	-15.602	-0.312	1.00	26.12	C
ATOM	561	C	LEU	A	74	17.355	-16.178	0.438	1.00	23.46	C
ATOM	562	O	LEU	A	74	18.398	-15.545	0.518	1.00	21.59	O
ATOM	563	CB	LEU	A	74	14.922	-15.728	0.593	1.00	26.77	C
ATOM	564	CG	LEU	A	74	14.153	-14.538	1.199	1.00	25.70	C
ATOM	565	CD1	LEU	A	74	12.799	-15.077	1.659	1.00	26.20	C

ATOM	566	CD2	LEU	A	74	14.879	-13.871	2.381	1.00	22.43	C
ATOM	567	N	CYS	A	75	17.225	-17.448	0.826	1.00	24.52	N
ATOM	568	CA	CYS	A	75	18.313	-18.240	1.395	1.00	26.88	C
ATOM	569	C	CYS	A	75	19.554	-18.212	0.475	1.00	28.33	C
ATOM	570	O	CYS	A	75	20.714	-18.148	0.939	1.00	26.66	O
ATOM	571	CB	CYS	A	75	17.830	-19.698	1.633	1.00	26.77	C
ATOM	572	SG	CYS	A	75	17.685	-20.118	3.425	1.00	27.16	S
ATOM	573	N	THR	A	76	19.283	-18.088	-0.822	1.00	30.35	N
ATOM	574	CA	THR	A	76	20.287	-18.225	-1.866	1.00	30.59	C
ATOM	575	C	THR	A	76	21.471	-17.261	-1.658	1.00	30.26	C
ATOM	576	O	THR	A	76	22.293	-17.437	-0.739	1.00	29.82	O
ATOM	577	CB	THR	A	76	19.623	-18.024	-3.284	1.00	32.29	C
ATOM	578	CG2	THR	A	76	20.023	-19.164	-4.228	1.00	32.43	C
ATOM	579	OG1	THR	A	76	18.179	-17.994	-3.171	1.00	31.05	O
ATOM	580	N	VAL	A	77	21.493	-16.179	-2.427	1.00	31.21	N
ATOM	581	CA	VAL	A	77	22.661	-15.299	-2.455	1.00	30.79	C
ATOM	582	C	VAL	A	77	22.468	-14.103	-1.514	1.00	32.91	C
ATOM	583	O	VAL	A	77	23.392	-13.312	-1.272	1.00	33.22	O
ATOM	584	CB	VAL	A	77	22.944	-14.813	-3.893	1.00	28.48	C
ATOM	585	CG1	VAL	A	77	22.227	-13.486	-4.176	1.00	25.60	C
ATOM	586	CG2	VAL	A	77	24.439	-14.718	-4.118	1.00	24.59	C
ATOM	587	N	ALA	A	78	21.257	-13.954	-0.987	1.00	33.04	N
ATOM	588	CA	ALA	A	78	21.110	-13.066	0.134	1.00	33.55	C
ATOM	589	C	ALA	A	78	22.005	-13.706	1.199	1.00	35.33	C
ATOM	590	O	ALA	A	78	23.151	-13.260	1.362	1.00	36.95	O
ATOM	591	CB	ALA	A	78	19.660	-13.008	0.576	1.00	32.27	C
ATOM	592	N	THR	A	79	21.683	-14.963	1.521	1.00	34.35	N
ATOM	593	CA	THR	A	79	22.100	-15.570	2.779	1.00	33.26	C
ATOM	594	C	THR	A	79	23.555	-16.077	2.835	1.00	32.95	C
ATOM	595	O	THR	A	79	24.250	-15.840	3.828	1.00	29.56	O
ATOM	596	CB	THR	A	79	21.140	-16.723	3.185	1.00	34.83	C
ATOM	597	CG2	THR	A	79	21.198	-16.977	4.704	1.00	35.95	C
ATOM	598	OG1	THR	A	79	19.791	-16.383	2.832	1.00	32.22	O
ATOM	599	N	LEU	A	80	24.027	-16.777	1.798	1.00	32.03	N
ATOM	600	CA	LEU	A	80	25.417	-17.297	1.808	1.00	28.26	C
ATOM	601	C	LEU	A	80	26.496	-16.225	1.630	1.00	27.39	C
ATOM	602	O	LEU	A	80	27.663	-16.471	1.914	1.00	27.30	O
ATOM	603	CB	LEU	A	80	25.609	-18.388	0.750	1.00	27.14	C
ATOM	604	CG	LEU	A	80	26.202	-18.027	-0.613	1.00	23.17	C
ATOM	605	CD1	LEU	A	80	27.191	-19.114	-1.025	1.00	23.29	C
ATOM	606	CD2	LEU	A	80	25.084	-17.888	-1.649	1.00	22.37	C
ATOM	607	N	ARG	A	81	26.117	-15.063	1.102	1.00	28.16	N
ATOM	608	CA	ARG	A	81	26.911	-13.843	1.282	1.00	28.08	C
ATOM	609	C	ARG	A	81	26.719	-13.293	2.706	1.00	29.33	C
ATOM	610	O	ARG	A	81	27.463	-12.415	3.169	1.00	28.86	O
ATOM	611	CB	ARG	A	81	26.477	-12.780	0.281	1.00	25.69	C
ATOM	612	CG	ARG	A	81	27.446	-11.627	0.186	1.00	24.75	C
ATOM	613	CD	ARG	A	81	27.337	-10.950	-1.152	1.00	25.26	C
ATOM	614	NE	ARG	A	81	27.236	-11.935	-2.211	1.00	28.70	N
ATOM	615	CZ	ARG	A	81	28.043	-12.988	-2.337	1.00	32.72	C
ATOM	616	NH1	ARG	A	81	29.145	-13.080	-1.599	1.00	31.48	N1+
ATOM	617	NH2	ARG	A	81	27.766	-13.933	-3.239	1.00	35.72	N
ATOM	618	N	GLU	A	82	25.660	-13.760	3.361	1.00	29.80	N
ATOM	619	CA	GLU	A	82	25.385	-13.405	4.745	1.00	30.13	C
ATOM	620	C	GLU	A	82	25.702	-14.591	5.709	1.00	28.95	C
ATOM	621	O	GLU	A	82	24.836	-15.378	6.068	1.00	25.40	O
ATOM	622	CB	GLU	A	82	23.930	-12.881	4.802	1.00	29.79	C
ATOM	623	CG	GLU	A	82	22.913	-13.604	5.673	1.00	28.95	C
ATOM	624	CD	GLU	A	82	22.631	-12.867	6.965	1.00	28.32	C
ATOM	625	OE1	GLU	A	82	23.152	-11.733	7.119	1.00	27.70	O
ATOM	626	OE2	GLU	A	82	21.883	-13.416	7.814	1.00	23.91	O1-
ATOM	627	N	THR	A	83	26.989	-14.795	5.996	1.00	29.92	N
ATOM	628	CA	THR	A	83	27.418	-16.000	6.718	1.00	32.38	C
ATOM	629	C	THR	A	83	28.141	-15.685	8.052	1.00	35.54	C
ATOM	630	O	THR	A	83	29.381	-15.602	8.095	1.00	37.17	O
ATOM	631	CB	THR	A	83	28.363	-16.937	5.826	1.00	29.73	C
ATOM	632	CG2	THR	A	83	27.562	-17.986	5.086	1.00	28.72	C
ATOM	633	OG1	THR	A	83	29.107	-16.168	4.865	1.00	28.72	O
ATOM	634	N	TYR	A	84	27.418	-15.611	9.171	1.00	36.72	N
ATOM	635	CA	TYR	A	84	28.134	-15.232	10.392	1.00	36.41	C
ATOM	636	C	TYR	A	84	27.787	-15.855	11.752	1.00	37.17	C

ATOM	637	O	TYR	A	84	28.696	-16.145	12.553	1.00	37.81	O
ATOM	638	CB	TYR	A	84	28.155	-13.719	10.504	1.00	32.67	C
ATOM	639	CG	TYR	A	84	29.547	-13.173	10.612	1.00	27.81	C
ATOM	640	CD1	TYR	A	84	30.321	-13.442	11.728	1.00	26.38	C
ATOM	641	CD2	TYR	A	84	30.000	-12.224	9.714	1.00	27.01	C
ATOM	642	CE1	TYR	A	84	31.467	-12.758	11.964	1.00	24.78	C
ATOM	643	CE2	TYR	A	84	31.147	-11.539	9.938	1.00	24.86	C
ATOM	644	CZ	TYR	A	84	31.865	-11.794	11.072	1.00	25.15	C
ATOM	645	OH	TYR	A	84	32.909	-10.976	11.395	1.00	27.73	O
ATOM	646	N	GLY	A	85	26.498	-15.954	12.073	1.00	37.02	N
ATOM	647	CA	GLY	A	85	26.123	-16.752	13.222	1.00	36.20	C
ATOM	648	C	GLY	A	85	25.033	-16.151	14.070	1.00	35.50	C
ATOM	649	O	GLY	A	85	25.291	-15.646	15.165	1.00	36.73	O
ATOM	650	N	GLU	A	86	23.816	-16.174	13.539	1.00	34.43	N
ATOM	651	CA	GLU	A	86	22.602	-16.106	14.366	1.00	31.00	C
ATOM	652	C	GLU	A	86	21.465	-16.802	13.613	1.00	28.33	C
ATOM	653	O	GLU	A	86	20.837	-17.735	14.124	1.00	28.62	O
ATOM	654	CB	GLU	A	86	22.220	-14.655	14.676	1.00	29.50	C
ATOM	655	CG	GLU	A	86	21.084	-14.522	15.646	1.00	25.84	C
ATOM	656	CD	GLU	A	86	21.097	-15.620	16.687	1.00	26.43	C
ATOM	657	OE1	GLU	A	86	21.992	-15.623	17.568	1.00	22.42	O
ATOM	658	OE2	GLU	A	86	20.223	-16.512	16.584	1.00	27.71	O1-
ATOM	659	N	MET	A	87	21.326	-16.474	12.340	1.00	24.32	N
ATOM	660	CA	MET	A	87	20.476	-17.275	11.494	1.00	24.03	C
ATOM	661	C	MET	A	87	21.175	-18.576	11.102	1.00	23.82	C
ATOM	662	O	MET	A	87	21.884	-18.629	10.092	1.00	22.28	O
ATOM	663	CB	MET	A	87	20.062	-16.483	10.266	1.00	22.69	C
ATOM	664	CG	MET	A	87	19.281	-15.254	10.625	1.00	21.49	C
ATOM	665	SD	MET	A	87	17.968	-15.060	9.479	1.00	25.30	S
ATOM	666	CE	MET	A	87	17.164	-13.558	10.089	1.00	25.45	C
ATOM	667	N	ALA	A	88	21.100	-19.558	12.007	1.00	24.16	N
ATOM	668	CA	ALA	A	88	21.551	-20.939	11.769	1.00	23.40	C
ATOM	669	C	ALA	A	88	20.674	-21.671	10.750	1.00	22.19	C
ATOM	670	O	ALA	A	88	20.034	-22.659	11.074	1.00	19.67	O
ATOM	671	CB	ALA	A	88	21.562	-21.707	13.092	1.00	23.53	C
ATOM	672	N	ASP	A	89	20.914	-21.344	9.489	1.00	24.31	N
ATOM	673	CA	ASP	A	89	19.989	-21.540	8.373	1.00	26.76	C
ATOM	674	C	ASP	A	89	18.712	-22.355	8.559	1.00	27.24	C
ATOM	675	O	ASP	A	89	18.747	-23.479	9.048	1.00	28.36	O
ATOM	676	CB	ASP	A	89	20.745	-22.081	7.155	1.00	29.44	C
ATOM	677	CG	ASP	A	89	20.619	-21.162	5.954	1.00	33.40	C
ATOM	678	OD1	ASP	A	89	21.358	-20.136	5.934	1.00	34.86	O
ATOM	679	OD2	ASP	A	89	19.716	-21.401	5.103	1.00	29.72	O1-
ATOM	680	N	CYS	A	90	17.732	-21.971	7.752	1.00	27.38	N
ATOM	681	CA	CYS	A	90	16.343	-22.307	8.008	1.00	26.95	C
ATOM	682	C	CYS	A	90	15.605	-22.522	6.664	1.00	26.81	C
ATOM	683	O	CYS	A	90	14.461	-22.077	6.485	1.00	28.42	O
ATOM	684	CB	CYS	A	90	15.719	-21.154	8.799	1.00	27.53	C
ATOM	685	SG	CYS	A	90	14.094	-21.429	9.556	1.00	25.39	S
ATOM	686	N	CYS	A	91	16.248	-23.192	5.708	1.00	24.62	N
ATOM	687	CA	CYS	A	91	15.559	-23.491	4.457	1.00	22.81	C
ATOM	688	C	CYS	A	91	15.315	-24.990	4.337	1.00	21.96	C
ATOM	689	O	CYS	A	91	14.563	-25.413	3.449	1.00	19.33	O
ATOM	690	CB	CYS	A	91	16.330	-22.934	3.255	1.00	24.46	C
ATOM	691	SG	CYS	A	91	17.922	-22.131	3.674	1.00	26.72	S
ATOM	692	N	ALA	A	92	15.547	-25.641	5.480	1.00	20.50	N
ATOM	693	CA	ALA	A	92	15.453	-27.100	5.702	1.00	21.22	C
ATOM	694	C	ALA	A	92	14.711	-27.984	4.682	1.00	24.74	C
ATOM	695	O	ALA	A	92	15.053	-27.983	3.494	1.00	24.03	O
ATOM	696	CB	ALA	A	92	14.900	-27.358	7.109	1.00	19.84	C
ATOM	697	N	LYS	A	93	13.819	-28.862	5.165	1.00	29.36	N
ATOM	698	CA	LYS	A	93	13.030	-29.749	4.272	1.00	32.05	C
ATOM	699	C	LYS	A	93	11.829	-28.986	3.713	1.00	32.90	C
ATOM	700	O	LYS	A	93	10.689	-29.355	4.021	1.00	32.86	O
ATOM	701	CB	LYS	A	93	12.505	-30.991	5.016	1.00	31.93	C
ATOM	702	CG	LYS	A	93	13.547	-31.775	5.787	1.00	33.86	C
ATOM	703	CD	LYS	A	93	13.897	-33.068	5.060	1.00	41.47	C
ATOM	704	CE	LYS	A	93	13.058	-34.327	5.515	1.00	43.89	C
ATOM	705	NZ	LYS	A	93	13.324	-35.551	4.649	1.00	41.62	N1+
ATOM	706	N	GLN	A	94	12.106	-27.847	3.058	1.00	34.28	N
ATOM	707	CA	GLN	A	94	11.143	-27.119	2.211	1.00	33.24	C

ATOM	708	C	GLN	A	94	9.733	-27.305	2.726	1.00	34.19	C
ATOM	709	O	GLN	A	94	9.042	-28.292	2.385	1.00	33.84	O
ATOM	710	CB	GLN	A	94	11.233	-27.588	0.762	1.00	31.80	C
ATOM	711	CG	GLN	A	94	10.790	-26.549	-0.236	1.00	34.94	C
ATOM	712	CD	GLN	A	94	11.426	-26.732	-1.637	1.00	38.30	C
ATOM	713	NE2	GLN	A	94	12.641	-26.177	-1.815	1.00	40.46	N
ATOM	714	OE1	GLN	A	94	10.825	-27.321	-2.563	1.00	32.88	O
ATOM	715	N	GLU	A	95	9.332	-26.345	3.554	1.00	32.90	N
ATOM	716	CA	GLU	A	95	8.335	-26.553	4.596	1.00	29.83	C
ATOM	717	C	GLU	A	95	7.742	-25.193	4.953	1.00	27.35	C
ATOM	718	O	GLU	A	95	8.463	-24.205	5.067	1.00	27.18	O
ATOM	719	CB	GLU	A	95	8.999	-27.179	5.835	1.00	29.20	C
ATOM	720	CG	GLU	A	95	10.428	-26.672	6.091	1.00	31.06	C
ATOM	721	CD	GLU	A	95	11.023	-27.126	7.434	1.00	31.73	C
ATOM	722	OE1	GLU	A	95	10.421	-28.009	8.097	1.00	33.07	O
ATOM	723	OE2	GLU	A	95	12.075	-26.563	7.821	1.00	28.41	O1-
ATOM	724	N	PRO	A	96	6.413	-25.117	5.082	1.00	26.21	N
ATOM	725	CA	PRO	A	96	5.812	-24.684	6.341	1.00	24.52	C
ATOM	726	C	PRO	A	96	6.808	-24.184	7.407	1.00	25.14	C
ATOM	727	O	PRO	A	96	7.050	-22.970	7.514	1.00	25.18	O
ATOM	728	CB	PRO	A	96	5.052	-25.927	6.769	1.00	25.77	C
ATOM	729	CG	PRO	A	96	4.695	-26.630	5.390	1.00	25.29	C
ATOM	730	CD	PRO	A	96	5.461	-25.931	4.308	1.00	22.87	C
ATOM	731	N	GLU	A	97	7.471	-25.114	8.099	1.00	25.87	N
ATOM	732	CA	GLU	A	97	8.490	-24.774	9.105	1.00	24.65	C
ATOM	733	C	GLU	A	97	9.746	-24.064	8.538	1.00	24.75	C
ATOM	734	O	GLU	A	97	10.841	-24.176	9.090	1.00	25.54	O
ATOM	735	CB	GLU	A	97	8.900	-26.035	9.903	1.00	22.10	C
ATOM	736	CG	GLU	A	97	9.205	-25.768	11.382	1.00	20.67	C
ATOM	737	CD	GLU	A	97	9.771	-26.971	12.146	1.00	21.89	C
ATOM	738	OE1	GLU	A	97	9.336	-28.118	11.890	1.00	20.02	O
ATOM	739	OE2	GLU	A	97	10.584	-26.752	13.080	1.00	21.31	O1-
ATOM	740	N	ARG	A	98	9.603	-23.344	7.433	1.00	25.09	N
ATOM	741	CA	ARG	A	98	10.673	-22.458	7.010	1.00	25.81	C
ATOM	742	C	ARG	A	98	10.317	-21.131	7.623	1.00	25.39	C
ATOM	743	O	ARG	A	98	10.978	-20.710	8.581	1.00	24.60	O
ATOM	744	CB	ARG	A	98	10.787	-22.358	5.466	1.00	28.52	C
ATOM	745	CG	ARG	A	98	10.015	-21.212	4.761	1.00	29.53	C
ATOM	746	CD	ARG	A	98	9.578	-21.625	3.364	1.00	32.14	C
ATOM	747	NE	ARG	A	98	8.122	-21.618	3.201	1.00	35.46	N
ATOM	748	CZ	ARG	A	98	7.473	-22.269	2.232	1.00	36.58	C
ATOM	749	NH1	ARG	A	98	8.153	-23.041	1.390	1.00	34.88	N1+
ATOM	750	NH2	ARG	A	98	6.140	-22.181	2.122	1.00	35.51	N
ATOM	751	N	ASN	A	99	9.118	-20.641	7.289	1.00	24.15	N
ATOM	752	CA	ASN	A	99	8.828	-19.225	7.492	1.00	23.31	C
ATOM	753	C	ASN	A	99	9.235	-18.901	8.910	1.00	22.87	C
ATOM	754	O	ASN	A	99	10.205	-18.165	9.103	1.00	20.69	O
ATOM	755	CB	ASN	A	99	7.347	-18.902	7.287	1.00	22.49	C
ATOM	756	CG	ASN	A	99	7.134	-17.493	6.738	1.00	24.38	C
ATOM	757	ND2	ASN	A	99	7.943	-16.540	7.206	1.00	21.20	N
ATOM	758	OD1	ASN	A	99	6.321	-17.287	5.821	1.00	28.44	O
ATOM	759	N	GLU	A	100	8.818	-19.821	9.785	1.00	21.65	N
ATOM	760	CA	GLU	A	100	8.975	-19.687	11.215	1.00	20.63	C
ATOM	761	C	GLU	A	100	10.448	-19.660	11.578	1.00	22.84	C
ATOM	762	O	GLU	A	100	11.049	-18.584	11.656	1.00	23.88	O
ATOM	763	CB	GLU	A	100	8.255	-20.822	11.950	1.00	16.75	C
ATOM	764	CG	GLU	A	100	6.754	-20.854	11.705	1.00	18.79	C
ATOM	765	CD	GLU	A	100	6.355	-21.788	10.556	1.00	24.05	C
ATOM	766	OE1	GLU	A	100	6.735	-22.979	10.603	1.00	23.63	O
ATOM	767	OE2	GLU	A	100	5.629	-21.357	9.622	1.00	26.06	O1-
ATOM	768	N	CYS	A	101	11.073	-20.826	11.570	1.00	26.26	N
ATOM	769	CA	CYS	A	101	12.183	-21.078	12.477	1.00	29.17	C
ATOM	770	C	CYS	A	101	13.170	-19.962	12.458	1.00	28.71	C
ATOM	771	O	CYS	A	101	13.708	-19.631	13.507	1.00	29.61	O
ATOM	772	CB	CYS	A	101	12.927	-22.363	12.140	1.00	31.73	C
ATOM	773	SG	CYS	A	101	14.563	-21.967	11.457	1.00	33.00	S
ATOM	774	N	PHE	A	102	13.459	-19.419	11.274	1.00	29.99	N
ATOM	775	CA	PHE	A	102	14.339	-18.251	11.208	1.00	30.99	C
ATOM	776	C	PHE	A	102	13.937	-17.084	10.309	1.00	30.01	C
ATOM	777	O	PHE	A	102	14.198	-15.929	10.646	1.00	31.09	O
ATOM	778	CB	PHE	A	102	15.794	-18.669	10.927	1.00	29.15	C

ATOM	779	CG	PHE	A	102	16.643	-18.701	12.165	1.00	31.69	C
ATOM	780	CD1	PHE	A	102	16.206	-18.063	13.325	1.00	31.15	C
ATOM	781	CD2	PHE	A	102	17.771	-19.508	12.236	1.00	33.88	C
ATOM	782	CE1	PHE	A	102	16.858	-18.244	14.537	1.00	33.52	C
ATOM	783	CE2	PHE	A	102	18.441	-19.698	13.451	1.00	34.28	C
ATOM	784	CZ	PHE	A	102	17.979	-19.066	14.605	1.00	35.49	C
ATOM	785	N	LEU	A	103	13.308	-17.360	9.174	1.00	29.36	N
ATOM	786	CA	LEU	A	103	13.003	-16.279	8.237	1.00	26.14	C
ATOM	787	C	LEU	A	103	11.972	-15.389	8.900	1.00	25.93	C
ATOM	788	O	LEU	A	103	12.147	-14.171	8.921	1.00	25.78	O
ATOM	789	CB	LEU	A	103	12.461	-16.815	6.906	1.00	19.74	C
ATOM	790	CG	LEU	A	103	13.228	-17.942	6.214	1.00	12.38	C
ATOM	791	CD1	LEU	A	103	12.638	-18.120	4.854	1.00	9.21	C
ATOM	792	CD2	LEU	A	103	14.710	-17.659	6.156	1.00	6.56	C
ATOM	793	N	GLN	A	104	11.085	-16.014	9.683	1.00	25.30	N
ATOM	794	CA	GLN	A	104	10.052	-15.283	10.414	1.00	25.11	C
ATOM	795	C	GLN	A	104	10.454	-14.979	11.849	1.00	25.04	C
ATOM	796	O	GLN	A	104	9.587	-14.855	12.719	1.00	24.08	O
ATOM	797	CB	GLN	A	104	8.742	-16.057	10.419	1.00	24.18	C
ATOM	798	CG	GLN	A	104	7.502	-15.185	10.263	1.00	26.71	C
ATOM	799	CD	GLN	A	104	6.727	-14.995	11.555	1.00	27.66	C
ATOM	800	NE2	GLN	A	104	5.873	-13.976	11.579	1.00	27.08	N
ATOM	801	OE1	GLN	A	104	6.884	-15.758	12.520	1.00	26.58	O
ATOM	802	N	HIS	A	105	11.771	-14.903	12.102	1.00	27.00	N
ATOM	803	CA	HIS	A	105	12.310	-14.502	13.416	1.00	24.47	C
ATOM	804	C	HIS	A	105	13.366	-13.402	13.432	1.00	21.96	C
ATOM	805	O	HIS	A	105	14.561	-13.626	13.636	1.00	18.43	O
ATOM	806	CB	HIS	A	105	12.745	-15.726	14.239	1.00	22.38	C
ATOM	807	CG	HIS	A	105	11.579	-16.471	14.785	1.00	21.89	C
ATOM	808	CD2	HIS	A	105	10.375	-16.007	15.196	1.00	21.60	C
ATOM	809	ND1	HIS	A	105	11.379	-17.806	14.479	1.00	20.64	N
ATOM	810	CE1	HIS	A	105	10.095	-18.100	14.625	1.00	22.23	C
ATOM	811	NE2	HIS	A	105	9.467	-17.022	15.048	1.00	23.35	N
ATOM	812	N	LYS	A	106	12.917	-12.304	12.860	1.00	19.95	N
ATOM	813	CA	LYS	A	106	12.919	-11.020	13.530	1.00	19.97	C
ATOM	814	C	LYS	A	106	13.352	-11.055	14.998	1.00	20.41	C
ATOM	815	O	LYS	A	106	12.569	-11.484	15.844	1.00	19.74	O
ATOM	816	CB	LYS	A	106	11.509	-10.434	13.441	1.00	18.97	C
ATOM	817	CG	LYS	A	106	10.855	-10.624	12.077	1.00	14.34	C
ATOM	818	CD	LYS	A	106	9.593	-9.822	11.956	1.00	12.34	C
ATOM	819	CE	LYS	A	106	8.917	-10.071	10.618	1.00	15.93	C
ATOM	820	NZ	LYS	A	106	7.629	-9.296	10.461	1.00	19.28	N1+
ATOM	821	N	ASP	A	107	14.647	-10.804	15.235	1.00	20.45	N
ATOM	822	CA	ASP	A	107	15.129	-10.023	16.386	1.00	19.31	C
ATOM	823	C	ASP	A	107	14.254	-8.775	16.470	1.00	20.32	C
ATOM	824	O	ASP	A	107	13.725	-8.318	15.464	1.00	18.89	O
ATOM	825	CB	ASP	A	107	16.582	-9.589	16.168	1.00	18.38	C
ATOM	826	CG	ASP	A	107	17.324	-9.293	17.468	1.00	20.19	C
ATOM	827	OD1	ASP	A	107	16.972	-8.328	18.186	1.00	20.89	O
ATOM	828	OD2	ASP	A	107	18.343	-9.964	17.709	1.00	20.24	O1-
ATOM	829	N	ASP	A	108	14.062	-8.248	17.673	1.00	22.88	N
ATOM	830	CA	ASP	A	108	12.971	-7.310	17.867	1.00	24.38	C
ATOM	831	C	ASP	A	108	13.419	-5.855	17.972	1.00	28.19	C
ATOM	832	O	ASP	A	108	13.842	-5.278	16.979	1.00	34.68	O
ATOM	833	CB	ASP	A	108	12.119	-7.724	19.066	1.00	21.46	C
ATOM	834	CG	ASP	A	108	10.665	-7.323	18.893	1.00	21.67	C
ATOM	835	OD1	ASP	A	108	10.407	-6.411	18.098	1.00	20.61	O
ATOM	836	OD2	ASP	A	108	9.792	-7.901	19.583	1.00	24.35	O1-
ATOM	837	N	ASN	A	109	13.182	-5.218	19.110	1.00	28.47	N
ATOM	838	CA	ASN	A	109	13.326	-3.763	19.264	1.00	28.03	C
ATOM	839	C	ASN	A	109	14.728	-3.298	18.833	1.00	28.47	C
ATOM	840	O	ASN	A	109	15.003	-3.159	17.631	1.00	29.63	O
ATOM	841	CB	ASN	A	109	13.100	-3.463	20.742	1.00	30.55	C
ATOM	842	CG	ASN	A	109	12.170	-2.307	20.987	1.00	30.28	C
ATOM	843	ND2	ASN	A	109	11.029	-2.303	20.315	1.00	34.20	N
ATOM	844	OD1	ASN	A	109	12.411	-1.514	21.893	1.00	28.96	O
ATOM	845	N	PRO	A	110	15.632	-3.006	19.797	1.00	27.35	N
ATOM	846	CA	PRO	A	110	16.964	-3.597	19.834	1.00	27.24	C
ATOM	847	C	PRO	A	110	17.241	-4.929	19.113	1.00	29.36	C
ATOM	848	O	PRO	A	110	17.484	-6.001	19.740	1.00	29.54	O
ATOM	849	CB	PRO	A	110	17.284	-3.559	21.317	1.00	25.13	C

ATOM	850	CG	PRO	A	110	16.640	-2.142	21.702	1.00	25.75	C
ATOM	851	CD	PRO	A	110	15.683	-1.751	20.558	1.00	24.19	C
ATOM	852	N	ASN	A	111	17.144	-4.835	17.781	1.00	27.97	N
ATOM	853	CA	ASN	A	111	18.078	-5.469	16.846	1.00	24.32	C
ATOM	854	C	ASN	A	111	19.394	-4.725	16.826	1.00	22.19	C
ATOM	855	O	ASN	A	111	19.509	-3.710	17.495	1.00	19.34	O
ATOM	856	CB	ASN	A	111	17.515	-5.455	15.440	1.00	21.73	C
ATOM	857	CG	ASN	A	111	16.979	-4.106	15.050	1.00	19.05	C
ATOM	858	ND2	ASN	A	111	17.263	-3.075	15.851	1.00	14.84	N
ATOM	859	OD1	ASN	A	111	16.299	-3.992	14.035	1.00	22.36	O
ATOM	860	N	LEU	A	112	20.147	-4.978	15.759	1.00	24.65	N
ATOM	861	CA	LEU	A	112	21.611	-4.907	15.800	1.00	26.73	C
ATOM	862	C	LEU	A	112	22.172	-3.568	15.277	1.00	26.67	C
ATOM	863	O	LEU	A	112	21.839	-2.531	15.854	1.00	23.61	O
ATOM	864	CB	LEU	A	112	22.185	-6.148	15.089	1.00	27.52	C
ATOM	865	CG	LEU	A	112	23.017	-7.089	15.972	1.00	31.09	C
ATOM	866	CD1	LEU	A	112	22.946	-8.557	15.481	1.00	26.90	C
ATOM	867	CD2	LEU	A	112	24.458	-6.546	16.003	1.00	29.65	C
ATOM	868	N	PRO	A	113	23.112	-3.565	14.288	1.00	29.08	N
ATOM	869	CA	PRO	A	113	23.734	-2.245	14.101	1.00	31.20	C
ATOM	870	C	PRO	A	113	22.679	-1.238	13.619	1.00	33.56	C
ATOM	871	O	PRO	A	113	21.661	-1.636	12.981	1.00	31.23	O
ATOM	872	CB	PRO	A	113	24.818	-2.486	13.030	1.00	29.19	C
ATOM	873	CG	PRO	A	113	24.576	-3.877	12.496	1.00	28.17	C
ATOM	874	CD	PRO	A	113	23.241	-4.343	13.047	1.00	29.64	C
ATOM	875	N	ARG	A	114	22.928	0.050	13.928	1.00	33.99	N
ATOM	876	CA	ARG	A	114	21.881	1.077	13.890	1.00	32.55	C
ATOM	877	C	ARG	A	114	22.292	2.540	13.957	1.00	32.21	C
ATOM	878	O	ARG	A	114	21.575	3.391	13.430	1.00	31.11	O
ATOM	879	CB	ARG	A	114	20.905	0.809	15.017	1.00	33.18	C
ATOM	880	CG	ARG	A	114	19.953	-0.281	14.661	1.00	36.64	C
ATOM	881	CD	ARG	A	114	19.020	-0.467	15.750	1.00	39.87	C
ATOM	882	NE	ARG	A	114	18.517	0.826	16.159	1.00	43.15	N
ATOM	883	CZ	ARG	A	114	17.756	0.977	17.222	1.00	47.64	C
ATOM	884	NH1	ARG	A	114	17.324	-0.119	17.852	1.00	51.30	N1+
ATOM	885	NH2	ARG	A	114	17.373	2.192	17.608	1.00	50.22	N
ATOM	886	N	LEU	A	115	23.284	2.864	14.788	1.00	33.02	N
ATOM	887	CA	LEU	A	115	23.491	4.243	15.202	1.00	33.18	C
ATOM	888	C	LEU	A	115	23.305	5.255	14.059	1.00	34.71	C
ATOM	889	O	LEU	A	115	22.386	6.065	14.145	1.00	35.24	O
ATOM	890	CB	LEU	A	115	24.860	4.423	15.860	1.00	32.34	C
ATOM	891	CG	LEU	A	115	25.037	5.442	17.017	1.00	31.68	C
ATOM	892	CD1	LEU	A	115	26.534	5.671	17.168	1.00	29.02	C
ATOM	893	CD2	LEU	A	115	24.324	6.786	16.764	1.00	26.36	C
ATOM	894	N	VAL	A	116	24.007	5.019	12.919	1.00	35.67	N
ATOM	895	CA	VAL	A	116	24.061	5.935	11.756	1.00	36.09	C
ATOM	896	C	VAL	A	116	22.692	6.478	11.298	1.00	37.34	C
ATOM	897	O	VAL	A	116	21.657	6.293	11.959	1.00	39.54	O
ATOM	898	CB	VAL	A	116	24.705	5.248	10.482	1.00	34.24	C
ATOM	899	CG1	VAL	A	116	26.241	4.937	10.734	1.00	33.93	C
ATOM	900	CG2	VAL	A	116	23.974	3.954	10.123	1.00	30.72	C
ATOM	901	N	ARG	A	117	22.732	7.203	10.185	1.00	37.72	N
ATOM	902	CA	ARG	A	117	21.486	7.644	9.511	1.00	40.29	C
ATOM	903	C	ARG	A	117	21.917	8.611	8.413	1.00	39.62	C
ATOM	904	O	ARG	A	117	23.104	8.596	8.037	1.00	41.35	O
ATOM	905	CB	ARG	A	117	20.547	8.386	10.490	1.00	42.16	C
ATOM	906	CG	ARG	A	117	19.116	7.763	10.656	1.00	46.39	C
ATOM	907	CD	ARG	A	117	18.704	7.899	12.134	1.00	51.79	C
ATOM	908	NE	ARG	A	117	19.092	9.229	12.637	1.00	56.27	N
ATOM	909	CZ	ARG	A	117	18.282	10.024	13.353	1.00	57.56	C
ATOM	910	NH1	ARG	A	117	18.086	11.271	12.915	1.00	57.16	N1+
ATOM	911	NH2	ARG	A	117	18.065	9.782	14.657	1.00	55.96	N
ATOM	912	N	PRO	A	118	20.982	9.415	7.863	1.00	37.83	N
ATOM	913	CA	PRO	A	118	21.179	10.738	7.262	1.00	38.11	C
ATOM	914	C	PRO	A	118	21.640	11.818	8.236	1.00	36.72	C
ATOM	915	O	PRO	A	118	21.590	11.611	9.463	1.00	38.18	O
ATOM	916	CB	PRO	A	118	19.774	11.075	6.712	1.00	38.53	C
ATOM	917	CG	PRO	A	118	18.892	10.088	7.386	1.00	38.63	C
ATOM	918	CD	PRO	A	118	19.697	8.870	7.443	1.00	37.42	C
ATOM	919	N	GLU	A	119	21.806	13.037	7.693	1.00	34.77	N
ATOM	920	CA	GLU	A	119	22.310	14.182	8.455	1.00	33.84	C

ATOM	921	C	GLU	A	119	22.333	15.588	7.791	1.00	31.31	C
ATOM	922	O	GLU	A	119	21.800	15.762	6.680	1.00	27.96	O
ATOM	923	CB	GLU	A	119	23.684	13.859	9.039	1.00	34.16	C
ATOM	924	CG	GLU	A	119	23.650	13.731	10.573	1.00	29.57	C
ATOM	925	CD	GLU	A	119	23.678	15.084	11.263	1.00	27.59	C
ATOM	926	OE1	GLU	A	119	24.804	15.594	11.417	1.00	25.30	O
ATOM	927	OE2	GLU	A	119	22.602	15.581	11.689	1.00	21.70	O1-
ATOM	928	N	VAL	A	120	23.119	16.516	8.368	1.00	29.09	N
ATOM	929	CA	VAL	A	120	22.754	17.938	8.468	1.00	27.70	C
ATOM	930	C	VAL	A	120	21.995	18.387	7.264	1.00	27.84	C
ATOM	931	O	VAL	A	120	20.779	18.514	7.318	1.00	29.90	O
ATOM	932	CB	VAL	A	120	23.973	18.925	8.622	1.00	27.28	C
ATOM	933	CG1	VAL	A	120	23.965	19.580	10.005	1.00	25.95	C
ATOM	934	CG2	VAL	A	120	25.309	18.221	8.353	1.00	27.55	C
ATOM	935	N	ASP	A	121	22.688	18.398	6.131	1.00	28.02	N
ATOM	936	CA	ASP	A	121	22.234	19.108	4.946	1.00	26.82	C
ATOM	937	C	ASP	A	121	21.939	18.197	3.749	1.00	25.21	C
ATOM	938	O	ASP	A	121	21.035	18.483	2.953	1.00	23.24	O
ATOM	939	CB	ASP	A	121	23.247	20.212	4.560	1.00	25.99	C
ATOM	940	CG	ASP	A	121	24.676	19.899	5.019	1.00	26.35	C
ATOM	941	OD1	ASP	A	121	25.271	18.950	4.459	1.00	27.73	O
ATOM	942	OD2	ASP	A	121	25.210	20.583	5.923	1.00	21.53	O1-
ATOM	943	N	VAL	A	122	22.591	17.041	3.682	1.00	23.49	N
ATOM	944	CA	VAL	A	122	22.769	16.383	2.386	1.00	25.51	C
ATOM	945	C	VAL	A	122	21.644	15.468	1.927	1.00	26.41	C
ATOM	946	O	VAL	A	122	21.527	15.180	0.732	1.00	25.53	O
ATOM	947	CB	VAL	A	122	24.124	15.616	2.299	1.00	25.87	C
ATOM	948	CG1	VAL	A	122	24.962	15.857	3.561	1.00	27.67	C
ATOM	949	CG2	VAL	A	122	23.896	14.114	2.031	1.00	25.34	C
ATOM	950	N	MET	A	123	20.814	15.019	2.867	1.00	29.60	N
ATOM	951	CA	MET	A	123	19.863	13.932	2.601	1.00	30.15	C
ATOM	952	C	MET	A	123	18.835	14.384	1.578	1.00	32.67	C
ATOM	953	O	MET	A	123	18.100	13.549	1.034	1.00	33.77	O
ATOM	954	CB	MET	A	123	19.148	13.469	3.889	1.00	27.54	C
ATOM	955	CG	MET	A	123	17.910	14.282	4.295	1.00	25.66	C
ATOM	956	SD	MET	A	123	18.202	15.511	5.615	1.00	22.74	S
ATOM	957	CE	MET	A	123	16.615	16.318	5.641	1.00	22.28	C
ATOM	958	N	CYS	A	124	18.756	15.707	1.361	1.00	32.18	N
ATOM	959	CA	CYS	A	124	17.895	16.302	0.328	1.00	29.82	C
ATOM	960	C	CYS	A	124	18.423	15.917	-1.060	1.00	27.70	C
ATOM	961	O	CYS	A	124	19.457	16.417	-1.503	1.00	27.67	O
ATOM	962	CB	CYS	A	124	17.879	17.819	0.455	1.00	27.21	C
ATOM	963	SG	CYS	A	124	17.768	18.608	-1.174	1.00	27.90	S
ATOM	964	N	ALA	A	126	16.357	14.257	-3.975	1.00	22.43	N
ATOM	965	CA	ALA	A	126	15.531	13.862	-5.118	1.00	26.10	C
ATOM	966	C	ALA	A	126	16.012	14.663	-6.335	1.00	27.83	C
ATOM	967	O	ALA	A	126	15.423	15.699	-6.716	1.00	28.31	O
ATOM	968	CB	ALA	A	126	14.054	14.116	-4.848	1.00	27.88	C
ATOM	969	N	PHE	A	127	17.206	14.284	-6.793	1.00	27.19	N
ATOM	970	CA	PHE	A	127	17.877	14.923	-7.916	1.00	25.57	C
ATOM	971	C	PHE	A	127	18.309	13.827	-8.896	1.00	25.18	C
ATOM	972	O	PHE	A	127	17.915	13.833	-10.062	1.00	24.40	O
ATOM	973	CB	PHE	A	127	19.107	15.705	-7.422	1.00	25.50	C
ATOM	974	CG	PHE	A	127	18.819	16.683	-6.298	1.00	24.31	C
ATOM	975	CD1	PHE	A	127	17.616	17.361	-6.224	1.00	23.10	C
ATOM	976	CD2	PHE	A	127	19.761	16.911	-5.307	1.00	24.98	C
ATOM	977	CE1	PHE	A	127	17.356	18.229	-5.169	1.00	24.24	C
ATOM	978	CE2	PHE	A	127	19.507	17.792	-4.260	1.00	23.55	C
ATOM	979	CZ	PHE	A	127	18.304	18.442	-4.188	1.00	22.96	C
ATOM	980	N	HIS	A	128	19.093	12.872	-8.395	1.00	23.97	N
ATOM	981	CA	HIS	A	128	19.603	11.748	-9.190	1.00	25.06	C
ATOM	982	C	HIS	A	128	19.666	10.507	-8.299	1.00	25.79	C
ATOM	983	O	HIS	A	128	20.736	9.885	-8.155	1.00	24.46	O
ATOM	984	CB	HIS	A	128	21.006	12.060	-9.762	1.00	26.48	C
ATOM	985	CG	HIS	A	128	21.630	10.926	-10.528	1.00	26.14	C
ATOM	986	CD2	HIS	A	128	21.599	10.603	-11.842	1.00	26.98	C
ATOM	987	ND1	HIS	A	128	22.404	9.955	-9.919	1.00	25.60	N
ATOM	988	CE1	HIS	A	128	22.814	9.086	-10.824	1.00	25.56	C
ATOM	989	NE2	HIS	A	128	22.341	9.456	-11.999	1.00	26.11	N
ATOM	990	N	ASP	A	129	18.631	10.376	-7.473	1.00	25.20	N
ATOM	991	CA	ASP	A	129	18.171	9.095	-6.944	1.00	23.48	C

ATOM	992	C	ASP	A	129	17.157	9.345	-5.843	1.00	24.69	C
ATOM	993	O	ASP	A	129	17.123	10.429	-5.245	1.00	22.84	O
ATOM	994	CB	ASP	A	129	19.312	8.207	-6.401	1.00	22.35	C
ATOM	995	CG	ASP	A	129	20.159	8.879	-5.310	1.00	19.90	C
ATOM	996	OD1	ASP	A	129	19.796	9.960	-4.801	1.00	16.62	O
ATOM	997	OD2	ASP	A	129	21.216	8.304	-4.963	1.00	18.41	O1-
ATOM	998	N	GLU	A	131	16.294	6.107	-4.845	1.00	26.76	N
ATOM	999	CA	GLU	A	131	16.936	4.947	-4.238	1.00	26.85	C
ATOM	1000	C	GLU	A	131	17.501	5.427	-2.883	1.00	26.10	C
ATOM	1001	O	GLU	A	131	17.117	4.893	-1.861	1.00	25.45	O
ATOM	1002	CB	GLU	A	131	18.035	4.384	-5.180	1.00	25.89	C
ATOM	1003	CG	GLU	A	131	17.551	3.445	-6.329	1.00	21.04	C
ATOM	1004	CD	GLU	A	131	16.340	3.971	-7.147	1.00	22.14	C
ATOM	1005	OE1	GLU	A	131	16.533	4.847	-8.019	1.00	19.96	O
ATOM	1006	OE2	GLU	A	131	15.222	3.409	-7.018	1.00	19.19	O1-
ATOM	1007	N	GLU	A	132	18.176	6.583	-2.862	1.00	27.14	N
ATOM	1008	CA	GLU	A	132	18.411	7.302	-1.593	1.00	27.48	C
ATOM	1009	C	GLU	A	132	17.082	7.842	-1.037	1.00	28.11	C
ATOM	1010	O	GLU	A	132	16.106	7.962	-1.754	1.00	24.38	O
ATOM	1011	CB	GLU	A	132	19.392	8.487	-1.758	1.00	26.09	C
ATOM	1012	CG	GLU	A	132	20.903	8.155	-1.682	1.00	25.28	C
ATOM	1013	CD	GLU	A	132	21.435	7.896	-0.264	1.00	21.57	C
ATOM	1014	OE1	GLU	A	132	21.255	6.778	0.262	1.00	19.27	O
ATOM	1015	OE2	GLU	A	132	22.139	8.767	0.272	1.00	15.77	O1-
ATOM	1016	N	THR	A	133	17.137	8.383	0.170	1.00	30.85	N
ATOM	1017	CA	THR	A	133	15.943	8.621	0.974	1.00	33.25	C
ATOM	1018	C	THR	A	133	14.702	7.774	0.628	1.00	34.75	C
ATOM	1019	O	THR	A	133	14.606	6.619	1.090	1.00	36.23	O
ATOM	1020	CB	THR	A	133	15.615	10.107	0.979	1.00	34.23	C
ATOM	1021	CG2	THR	A	133	16.767	10.868	1.680	1.00	36.08	C
ATOM	1022	OG1	THR	A	133	15.497	10.586	-0.370	1.00	35.21	O
ATOM	1023	N	PHE	A	134	13.798	8.301	-0.209	1.00	34.14	N
ATOM	1024	CA	PHE	A	134	12.474	7.689	-0.444	1.00	32.44	C
ATOM	1025	C	PHE	A	134	12.485	6.162	-0.360	1.00	32.71	C
ATOM	1026	O	PHE	A	134	11.952	5.604	0.584	1.00	35.71	O
ATOM	1027	CB	PHE	A	134	11.886	8.175	-1.787	1.00	30.94	C
ATOM	1028	CG	PHE	A	134	11.252	7.086	-2.630	1.00	29.00	C
ATOM	1029	CD1	PHE	A	134	10.070	6.474	-2.245	1.00	30.88	C
ATOM	1030	CD2	PHE	A	134	11.853	6.672	-3.805	1.00	28.45	C
ATOM	1031	CE1	PHE	A	134	9.508	5.473	-3.015	1.00	29.95	C
ATOM	1032	CE2	PHE	A	134	11.302	5.674	-4.585	1.00	28.90	C
ATOM	1033	CZ	PHE	A	134	10.132	5.074	-4.191	1.00	31.63	C
ATOM	1034	N	LEU	A	135	13.227	5.508	-1.242	1.00	32.49	N
ATOM	1035	CA	LEU	A	135	13.400	4.061	-1.193	1.00	32.55	C
ATOM	1036	C	LEU	A	135	14.020	3.666	0.150	1.00	32.40	C
ATOM	1037	O	LEU	A	135	13.384	2.984	0.946	1.00	33.86	O
ATOM	1038	CB	LEU	A	135	14.295	3.623	-2.362	1.00	34.15	C
ATOM	1039	CG	LEU	A	135	14.898	2.211	-2.532	1.00	37.12	C
ATOM	1040	CD1	LEU	A	135	14.673	1.676	-3.994	1.00	35.21	C
ATOM	1041	CD2	LEU	A	135	16.396	2.278	-2.232	1.00	33.42	C
ATOM	1042	N	LYS	A	136	15.169	4.258	0.477	1.00	32.96	N
ATOM	1043	CA	LYS	A	136	15.967	3.890	1.662	1.00	31.27	C
ATOM	1044	C	LYS	A	136	15.382	4.437	2.974	1.00	30.10	C
ATOM	1045	O	LYS	A	136	16.095	4.623	3.975	1.00	27.30	O
ATOM	1046	CB	LYS	A	136	17.429	4.370	1.501	1.00	30.80	C
ATOM	1047	CG	LYS	A	136	18.317	3.488	0.613	1.00	25.87	C
ATOM	1048	CD	LYS	A	136	19.724	4.099	0.430	1.00	25.64	C
ATOM	1049	CE	LYS	A	136	20.846	3.309	1.182	1.00	21.64	C
ATOM	1050	NZ	LYS	A	136	22.219	3.921	1.063	1.00	11.74	N1+
ATOM	1051	N	LYS	A	137	14.073	4.642	2.986	1.00	28.98	N
ATOM	1052	CA	LYS	A	137	13.340	4.610	4.251	1.00	27.29	C
ATOM	1053	C	LYS	A	137	12.719	3.231	4.562	1.00	25.01	C
ATOM	1054	O	LYS	A	137	11.769	3.148	5.343	1.00	19.88	O
ATOM	1055	CB	LYS	A	137	12.267	5.703	4.278	1.00	27.89	C
ATOM	1056	CG	LYS	A	137	11.043	5.458	3.424	1.00	25.43	C
ATOM	1057	CD	LYS	A	137	10.013	6.549	3.657	1.00	24.61	C
ATOM	1058	CE	LYS	A	137	9.532	6.581	5.121	1.00	25.80	C
ATOM	1059	NZ	LYS	A	137	10.478	7.205	6.080	1.00	22.10	N1+
ATOM	1060	N	TYR	A	138	13.261	2.170	3.944	1.00	23.60	N
ATOM	1061	CA	TYR	A	138	12.987	0.797	4.367	1.00	23.57	C
ATOM	1062	C	TYR	A	138	14.201	-0.014	4.898	1.00	25.14	C

```
ATOM   1063  O   TYR A 138      14.157  -0.518   6.019  1.00 26.51           O
ATOM   1064  CB  TYR A 138      12.287   0.013   3.262  1.00 23.90           C
ATOM   1065  CG  TYR A 138      11.095   0.708   2.626  1.00 23.12           C
ATOM   1066  CD1 TYR A 138      10.272   1.532   3.375  1.00 21.31           C
ATOM   1067  CD2 TYR A 138      10.950   0.725   1.238  1.00 20.04           C
ATOM   1068  CE1 TYR A 138       9.396   2.388   2.764  1.00 21.87           C
ATOM   1069  CE2 TYR A 138      10.096   1.584   0.627  1.00 18.89           C
ATOM   1070  CZ  TYR A 138       9.347   2.451   1.385  1.00 19.37           C
ATOM   1071  OH  TYR A 138       8.863   3.599   0.796  1.00 16.36           O
ATOM   1072  N   LEU A 139      15.348  -0.004   4.229  1.00 26.31           N
ATOM   1073  CA  LEU A 139      16.544  -0.492   4.932  1.00 28.30           C
ATOM   1074  C   LEU A 139      16.903   0.423   6.137  1.00 30.40           C
ATOM   1075  O   LEU A 139      18.050   0.459   6.603  1.00 33.77           O
ATOM   1076  CB  LEU A 139      17.764  -0.643   3.987  1.00 23.98           C
ATOM   1077  CG  LEU A 139      18.987  -1.356   4.621  1.00 21.06           C
ATOM   1078  CD1 LEU A 139      19.161  -2.769   4.075  1.00 15.84           C
ATOM   1079  CD2 LEU A 139      20.249  -0.513   4.429  1.00 18.03           C
ATOM   1080  N   TYR A 140      15.914   1.125   6.676  1.00 28.28           N
ATOM   1081  CA  TYR A 140      16.160   2.032   7.782  1.00 27.48           C
ATOM   1082  C   TYR A 140      14.903   2.160   8.640  1.00 26.31           C
ATOM   1083  O   TYR A 140      14.644   1.316   9.484  1.00 25.70           O
ATOM   1084  CB  TYR A 140      16.555   3.370   7.196  1.00 29.37           C
ATOM   1085  CG  TYR A 140      18.029   3.568   7.070  1.00 32.07           C
ATOM   1086  CD1 TYR A 140      18.907   2.754   7.774  1.00 32.26           C
ATOM   1087  CD2 TYR A 140      18.511   4.785   6.615  1.00 35.07           C
ATOM   1088  CE1 TYR A 140      20.189   3.177   8.085  1.00 31.31           C
ATOM   1089  CE2 TYR A 140      19.788   5.220   6.938  1.00 34.45           C
ATOM   1090  CZ  TYR A 140      20.604   4.429   7.691  1.00 30.17           C
ATOM   1091  OH  TYR A 140      21.706   5.009   8.252  1.00 30.52           O
ATOM   1092  N   GLU A 141      13.993   3.007   8.184  1.00 27.43           N
ATOM   1093  CA  GLU A 141      12.635   3.080   8.702  1.00 26.66           C
ATOM   1094  C   GLU A 141      11.830   1.793   8.474  1.00 25.94           C
ATOM   1095  O   GLU A 141      10.589   1.841   8.394  1.00 24.42           O
ATOM   1096  CB  GLU A 141      11.920   4.269   8.058  1.00 27.66           C
ATOM   1097  CG  GLU A 141      12.753   5.571   8.070  1.00 28.85           C
ATOM   1098  CD  GLU A 141      12.126   6.665   8.930  1.00 30.04           C
ATOM   1099  OE1 GLU A 141      12.059   6.463  10.169  1.00 28.05           O
ATOM   1100  OE2 GLU A 141      11.684   7.706   8.368  1.00 27.92           O1-
ATOM   1101  N   ILE A 142      12.543   0.668   8.301  1.00 25.02           N
ATOM   1102  CA  ILE A 142      11.959  -0.670   8.411  1.00 23.92           C
ATOM   1103  C   ILE A 142      12.891  -1.879   8.483  1.00 22.66           C
ATOM   1104  O   ILE A 142      12.422  -3.019   8.460  1.00 21.14           O
ATOM   1105  CB  ILE A 142      10.920  -0.960   7.307  1.00 25.56           C
ATOM   1106  CG1 ILE A 142       9.839  -1.855   7.893  1.00 31.26           C
ATOM   1107  CG2 ILE A 142      11.533  -1.747   6.136  1.00 26.16           C
ATOM   1108  CD1 ILE A 142       9.808  -1.835   9.391  1.00 32.34           C
ATOM   1109  N   ALA A 143      14.192  -1.655   8.574  1.00 21.53           N
ATOM   1110  CA  ALA A 143      15.080  -2.739   8.995  1.00 21.28           C
ATOM   1111  C   ALA A 143      15.259  -2.555  10.491  1.00 21.40           C
ATOM   1112  O   ALA A 143      15.077  -3.478  11.295  1.00 20.55           O
ATOM   1113  CB  ALA A 143      16.437  -2.637   8.279  1.00 20.32           C
ATOM   1114  N   ARG A 144      15.496  -1.300  10.850  1.00 21.00           N
ATOM   1115  CA  ARG A 144      15.726  -0.906  12.216  1.00 21.33           C
ATOM   1116  C   ARG A 144      14.498  -1.163  13.053  1.00 21.04           C
ATOM   1117  O   ARG A 144      13.397  -0.738  12.694  1.00 20.32           O
ATOM   1118  CB  ARG A 144      16.052   0.577  12.271  1.00 21.01           C
ATOM   1119  CG  ARG A 144      17.510   0.863  12.247  1.00 17.71           C
ATOM   1120  CD  ARG A 144      18.023   1.041  10.857  1.00 17.22           C
ATOM   1121  NE  ARG A 144      18.645   2.342  10.630  1.00 18.81           N
ATOM   1122  CZ  ARG A 144      19.496   2.950  11.447  1.00 17.78           C
ATOM   1123  NH1 ARG A 144      19.398   2.791  12.749  1.00 22.32           N1+
ATOM   1124  NH2 ARG A 144      20.194   3.978  11.001  1.00 19.08           N
ATOM   1125  N   ARG A 145      14.686  -1.933  14.117  1.00 22.67           N
ATOM   1126  CA  ARG A 145      13.838  -1.888  15.297  1.00 22.80           C
ATOM   1127  C   ARG A 145      12.341  -1.995  14.978  1.00 23.74           C
ATOM   1128  O   ARG A 145      11.492  -1.697  15.827  1.00 24.29           O
ATOM   1129  CB  ARG A 145      14.150  -0.595  16.060  1.00 23.74           C
ATOM   1130  CG  ARG A 145      13.284   0.605  15.663  1.00 24.56           C
ATOM   1131  CD  ARG A 145      14.041   1.882  15.206  1.00 23.54           C
ATOM   1132  NE  ARG A 145      13.036   2.868  14.811  1.00 23.80           N
ATOM   1133  CZ  ARG A 145      13.214   3.859  13.954  1.00 24.48           C
```

```
ATOM   1134  NH1 ARG A 145      14.272   4.656  14.069  1.00 24.11           N1+
ATOM   1135  NH2 ARG A 145      12.154   4.288  13.278  1.00 24.06           N
ATOM   1136  N   HIS A 146      12.021  -2.380  13.739  1.00 23.24           N
ATOM   1137  CA  HIS A 146      10.633  -2.505  13.276  1.00 21.90           C
ATOM   1138  C   HIS A 146      10.413  -3.719  12.380  1.00 19.01           C
ATOM   1139  O   HIS A 146       9.649  -3.649  11.433  1.00 15.22           O
ATOM   1140  CB  HIS A 146      10.196  -1.244  12.519  1.00 21.14           C
ATOM   1141  CG  HIS A 146      10.226  -0.013  13.357  1.00 23.07           C
ATOM   1142  CD2 HIS A 146      10.883   1.158  13.170  1.00 24.02           C
ATOM   1143  ND1 HIS A 146       9.778   0.000  14.658  1.00 21.23           N
ATOM   1144  CE1 HIS A 146      10.178   1.100  15.251  1.00 24.29           C
ATOM   1145  NE2 HIS A 146      10.844   1.831  14.373  1.00 24.47           N
ATOM   1146  N   PRO A 147      10.896  -4.885  12.783  1.00 18.32           N
ATOM   1147  CA  PRO A 147      10.624  -5.945  11.843  1.00 19.37           C
ATOM   1148  C   PRO A 147       9.128  -6.280  11.753  1.00 24.48           C
ATOM   1149  O   PRO A 147       8.718  -6.993  10.833  1.00 27.95           O
ATOM   1150  CB  PRO A 147      11.461  -7.097  12.365  1.00 17.35           C
ATOM   1151  CG  PRO A 147      12.544  -6.430  13.108  1.00 17.68           C
ATOM   1152  CD  PRO A 147      11.866  -5.332  13.806  1.00 18.03           C
ATOM   1153  N   TYR A 148       8.294  -5.713  12.629  1.00 24.82           N
ATOM   1154  CA  TYR A 148       6.868  -6.045  12.564  1.00 25.01           C
ATOM   1155  C   TYR A 148       6.009  -4.990  11.846  1.00 26.88           C
ATOM   1156  O   TYR A 148       5.048  -5.353  11.167  1.00 29.08           O
ATOM   1157  CB  TYR A 148       6.326  -6.357  13.960  1.00 21.84           C
ATOM   1158  CG  TYR A 148       6.986  -7.573  14.586  1.00 20.84           C
ATOM   1159  CD1 TYR A 148       8.272  -7.502  15.093  1.00 21.27           C
ATOM   1160  CD2 TYR A 148       6.368  -8.812  14.576  1.00 21.92           C
ATOM   1161  CE1 TYR A 148       8.926  -8.629  15.558  1.00 18.20           C
ATOM   1162  CE2 TYR A 148       7.019  -9.947  15.036  1.00 19.43           C
ATOM   1163  CZ  TYR A 148       8.298  -9.843  15.521  1.00 17.54           C
ATOM   1164  OH  TYR A 148       8.966 -10.954  15.973  1.00 19.26           O
ATOM   1165  N   PHE A 149       6.519  -3.755  11.776  1.00 26.54           N
ATOM   1166  CA  PHE A 149       5.862  -2.630  11.080  1.00 23.95           C
ATOM   1167  C   PHE A 149       4.992  -3.027   9.893  1.00 23.50           C
ATOM   1168  O   PHE A 149       5.444  -3.643   8.915  1.00 20.73           O
ATOM   1169  CB  PHE A 149       6.892  -1.578  10.614  1.00 19.95           C
ATOM   1170  CG  PHE A 149       6.368  -0.157  10.584  1.00 18.95           C
ATOM   1171  CD1 PHE A 149       5.244   0.189   9.840  1.00 21.19           C
ATOM   1172  CD2 PHE A 149       7.044   0.853  11.240  1.00 17.97           C
ATOM   1173  CE1 PHE A 149       4.814   1.514   9.748  1.00 17.03           C
ATOM   1174  CE2 PHE A 149       6.613   2.177  11.152  1.00 17.64           C
ATOM   1175  CZ  PHE A 149       5.501   2.501  10.402  1.00 13.77           C
ATOM   1176  N   ALA A 151       3.646  -2.885   6.915  1.00 16.85           N
ATOM   1177  CA  ALA A 151       3.539  -2.791   5.463  1.00 14.78           C
ATOM   1178  C   ALA A 151       2.681  -1.614   4.972  1.00 15.60           C
ATOM   1179  O   ALA A 151       3.223  -0.518   4.831  1.00 17.98           O
ATOM   1180  CB  ALA A 151       3.038  -4.104   4.884  1.00 13.97           C
ATOM   1181  N   PRO A 152       1.346  -1.776   4.762  1.00 13.15           N
ATOM   1182  CA  PRO A 152       0.722  -0.739   3.932  1.00  9.23           C
ATOM   1183  C   PRO A 152       0.901   0.647   4.494  1.00  9.42           C
ATOM   1184  O   PRO A 152       1.203   1.559   3.742  1.00 12.31           O
ATOM   1185  CB  PRO A 152      -0.746  -1.137   3.863  1.00  3.96           C
ATOM   1186  CG  PRO A 152      -0.950  -1.981   4.978  1.00  9.07           C
ATOM   1187  CD  PRO A 152       0.329  -2.722   5.244  1.00 12.15           C
ATOM   1188  N   GLU A 153       1.031   0.730   5.816  1.00 11.50           N
ATOM   1189  CA  GLU A 153       1.234   2.006   6.514  1.00  9.86           C
ATOM   1190  C   GLU A 153       2.605   2.638   6.305  1.00 10.68           C
ATOM   1191  O   GLU A 153       2.752   3.857   6.337  1.00  9.78           O
ATOM   1192  CB  GLU A 153       0.971   1.828   7.995  1.00  5.51           C
ATOM   1193  CG  GLU A 153      -0.476   2.003   8.348  1.00  7.11           C
ATOM   1194  CD  GLU A 153      -0.969   3.409   8.114  1.00  7.65           C
ATOM   1195  OE1 GLU A 153      -0.136   4.313   8.022  1.00  5.80           O
ATOM   1196  OE2 GLU A 153      -2.194   3.611   8.020  1.00 11.12           O1-
ATOM   1197  N   LEU A 154       3.597   1.806   6.027  1.00 13.56           N
ATOM   1198  CA  LEU A 154       4.861   2.287   5.484  1.00 17.58           C
ATOM   1199  C   LEU A 154       4.729   2.816   4.045  1.00 19.77           C
ATOM   1200  O   LEU A 154       5.245   3.881   3.731  1.00 19.71           O
ATOM   1201  CB  LEU A 154       5.914   1.189   5.541  1.00 14.47           C
ATOM   1202  CG  LEU A 154       7.016   1.644   6.467  1.00 16.08           C
ATOM   1203  CD1 LEU A 154       8.162   0.670   6.454  1.00 16.69           C
ATOM   1204  CD2 LEU A 154       7.461   3.019   6.022  1.00 19.40           C
```

```
ATOM   1205  N   LEU A 155       3.982   2.112   3.201  1.00 20.63           N
ATOM   1206  CA  LEU A 155       3.669   2.622   1.872  1.00 22.58           C
ATOM   1207  C   LEU A 155       2.916   3.949   2.012  1.00 25.56           C
ATOM   1208  O   LEU A 155       3.099   4.850   1.190  1.00 27.00           O
ATOM   1209  CB  LEU A 155       2.801   1.636   1.075  1.00 19.65           C
ATOM   1210  CG  LEU A 155       3.080   0.133   1.019  1.00 17.19           C
ATOM   1211  CD1 LEU A 155       3.069  -0.273  -0.422  1.00 18.14           C
ATOM   1212  CD2 LEU A 155       4.396  -0.259   1.647  1.00 17.17           C
ATOM   1213  N   PHE A 156       2.120   4.101   3.079  1.00 26.64           N
ATOM   1214  CA  PHE A 156       1.490   5.403   3.375  1.00 26.26           C
ATOM   1215  C   PHE A 156       2.460   6.391   4.033  1.00 24.10           C
ATOM   1216  O   PHE A 156       2.373   7.601   3.814  1.00 23.49           O
ATOM   1217  CB  PHE A 156       0.240   5.268   4.260  1.00 23.92           C
ATOM   1218  CG  PHE A 156      -0.286   6.590   4.749  1.00 21.57           C
ATOM   1219  CD1 PHE A 156      -0.843   7.500   3.865  1.00 20.83           C
ATOM   1220  CD2 PHE A 156      -0.113   6.977   6.066  1.00 20.34           C
ATOM   1221  CE1 PHE A 156      -1.209   8.766   4.286  1.00 16.36           C
ATOM   1222  CE2 PHE A 156      -0.481   8.251   6.492  1.00 17.60           C
ATOM   1223  CZ  PHE A 156      -1.029   9.140   5.594  1.00 16.53           C
ATOM   1224  N   PHE A 157       3.415   5.874   4.791  1.00 21.35           N
ATOM   1225  CA  PHE A 157       4.441   6.724   5.339  1.00 21.42           C
ATOM   1226  C   PHE A 157       5.657   6.859   4.427  1.00 22.33           C
ATOM   1227  O   PHE A 157       6.712   7.303   4.879  1.00 23.73           O
ATOM   1228  CB  PHE A 157       4.863   6.195   6.700  1.00 21.49           C
ATOM   1229  CG  PHE A 157       4.238   6.920   7.846  1.00 17.83           C
ATOM   1230  CD1 PHE A 157       3.628   8.139   7.652  1.00 17.24           C
ATOM   1231  CD2 PHE A 157       4.376   6.444   9.120  1.00 19.41           C
ATOM   1232  CE1 PHE A 157       3.197   8.872   8.688  1.00 14.49           C
ATOM   1233  CE2 PHE A 157       3.948   7.170  10.159  1.00 18.81           C
ATOM   1234  CZ  PHE A 157       3.362   8.398   9.940  1.00 20.97           C
ATOM   1235  N   ALA A 158       5.524   6.405   3.175  1.00 23.41           N
ATOM   1236  CA  ALA A 158       6.590   6.492   2.160  1.00 21.51           C
ATOM   1237  C   ALA A 158       6.383   7.773   1.393  1.00 22.27           C
ATOM   1238  O   ALA A 158       7.251   8.647   1.377  1.00 22.61           O
ATOM   1239  CB  ALA A 158       6.526   5.321   1.199  1.00 16.28           C
ATOM   1240  N   LYS A 159       5.185   7.930   0.840  1.00 22.44           N
ATOM   1241  CA  LYS A 159       4.829   9.184   0.200  1.00 21.86           C
ATOM   1242  C   LYS A 159       5.146  10.345   1.144  1.00 23.62           C
ATOM   1243  O   LYS A 159       5.899  11.218   0.761  1.00 25.81           O
ATOM   1244  CB  LYS A 159       3.358   9.187  -0.202  1.00 19.48           C
ATOM   1245  CG  LYS A 159       3.096   8.664  -1.584  1.00 18.68           C
ATOM   1246  CD  LYS A 159       4.087   7.579  -1.993  1.00 19.63           C
ATOM   1247  CE  LYS A 159       3.781   7.040  -3.387  1.00 20.86           C
ATOM   1248  NZ  LYS A 159       4.795   6.042  -3.824  1.00 23.26           N1+
ATOM   1249  N   ARG A 160       4.850  10.195   2.436  1.00 23.94           N
ATOM   1250  CA  ARG A 160       5.123  11.255   3.413  1.00 25.27           C
ATOM   1251  C   ARG A 160       6.546  11.157   3.957  1.00 26.63           C
ATOM   1252  O   ARG A 160       6.750  11.043   5.183  1.00 23.87           O
ATOM   1253  CB  ARG A 160       4.135  11.184   4.583  1.00 28.93           C
ATOM   1254  CG  ARG A 160       2.655  11.179   4.194  1.00 27.51           C
ATOM   1255  CD  ARG A 160       2.030  12.549   4.347  1.00 25.69           C
ATOM   1256  NE  ARG A 160       0.589  12.455   4.562  1.00 24.09           N
ATOM   1257  CZ  ARG A 160       0.025  12.102   5.714  1.00 22.36           C
ATOM   1258  NH1 ARG A 160       0.772  11.875   6.797  1.00 20.48           N1+
ATOM   1259  NH2 ARG A 160      -1.292  11.998   5.786  1.00 19.93           N
ATOM   1260  N   TYR A 161       7.468  10.909   3.020  1.00 26.96           N
ATOM   1261  CA  TYR A 161       8.917  11.151   3.154  1.00 25.36           C
ATOM   1262  C   TYR A 161       9.285  11.672   1.781  1.00 25.00           C
ATOM   1263  O   TYR A 161       9.938  12.717   1.649  1.00 26.93           O
ATOM   1264  CB  TYR A 161       9.666   9.839   3.404  1.00 22.15           C
ATOM   1265  CG  TYR A 161      11.146   9.948   3.736  1.00 19.16           C
ATOM   1266  CD1 TYR A 161      11.649  10.985   4.526  1.00 21.34           C
ATOM   1267  CD2 TYR A 161      12.035   8.967   3.311  1.00 17.23           C
ATOM   1268  CE1 TYR A 161      13.021  11.033   4.875  1.00 18.50           C
ATOM   1269  CE2 TYR A 161      13.376   9.007   3.641  1.00 15.97           C
ATOM   1270  CZ  TYR A 161      13.870  10.035   4.411  1.00 16.75           C
ATOM   1271  OH  TYR A 161      15.220  10.080   4.642  1.00 14.91           O
ATOM   1272  N   LYS A 162       8.684  11.030   0.776  1.00 24.44           N
ATOM   1273  CA  LYS A 162       8.762  11.445  -0.629  1.00 20.54           C
ATOM   1274  C   LYS A 162       8.217  12.847  -0.779  1.00 18.06           C
ATOM   1275  O   LYS A 162       8.972  13.809  -0.711  1.00 18.82           O
```

ATOM	1276	CB	LYS	A	162	7.963	10.477	-1.508	1.00	19.05	C
ATOM	1277	CG	LYS	A	162	8.158	10.649	-3.005	1.00	18.72	C
ATOM	1278	CD	LYS	A	162	7.086	11.530	-3.600	1.00	15.41	C
ATOM	1279	CE	LYS	A	162	6.906	11.243	-5.053	1.00	13.03	C
ATOM	1280	NZ	LYS	A	162	6.676	9.809	-5.269	1.00	14.01	N1+
ATOM	1281	N	ALA	A	163	6.893	12.947	-0.846	1.00	17.36	N
ATOM	1282	CA	ALA	A	163	6.153	14.205	-0.954	1.00	17.24	C
ATOM	1283	C	ALA	A	163	6.795	15.380	-0.258	1.00	17.12	C
ATOM	1284	O	ALA	A	163	6.690	16.493	-0.748	1.00	19.51	O
ATOM	1285	CB	ALA	A	163	4.736	14.030	-0.439	1.00	14.73	C
ATOM	1286	N	ALA	A	164	7.419	15.144	0.894	1.00	16.39	N
ATOM	1287	CA	ALA	A	164	8.122	16.201	1.603	1.00	17.36	C
ATOM	1288	C	ALA	A	164	9.253	16.693	0.709	1.00	19.77	C
ATOM	1289	O	ALA	A	164	9.142	17.759	0.114	1.00	20.30	O
ATOM	1290	CB	ALA	A	164	8.670	15.686	2.930	1.00	15.34	C
ATOM	1291	N	PHE	A	165	10.238	15.827	0.466	1.00	21.94	N
ATOM	1292	CA	PHE	A	165	11.379	16.135	-0.393	1.00	20.00	C
ATOM	1293	C	PHE	A	165	10.864	16.794	-1.664	1.00	19.97	C
ATOM	1294	O	PHE	A	165	11.217	17.951	-1.933	1.00	19.72	O
ATOM	1295	CB	PHE	A	165	12.108	14.842	-0.765	1.00	24.10	C
ATOM	1296	CG	PHE	A	165	13.261	14.491	0.139	1.00	26.13	C
ATOM	1297	CD1	PHE	A	165	13.084	14.331	1.495	1.00	26.42	C
ATOM	1298	CD2	PHE	A	165	14.505	14.200	-0.396	1.00	27.17	C
ATOM	1299	CE1	PHE	A	165	14.128	13.891	2.287	1.00	24.89	C
ATOM	1300	CE2	PHE	A	165	15.536	13.758	0.397	1.00	22.89	C
ATOM	1301	CZ	PHE	A	165	15.350	13.609	1.730	1.00	22.00	C
ATOM	1302	N	THR	A	166	9.807	16.176	-2.209	1.00	18.51	N
ATOM	1303	CA	THR	A	166	9.151	16.502	-3.488	1.00	17.70	C
ATOM	1304	C	THR	A	166	8.432	17.857	-3.457	1.00	20.02	C
ATOM	1305	O	THR	A	166	7.358	18.038	-4.048	1.00	21.09	O
ATOM	1306	CB	THR	A	166	8.105	15.409	-3.838	1.00	18.48	C
ATOM	1307	CG2	THR	A	166	7.511	15.601	-5.242	1.00	15.89	C
ATOM	1308	OG1	THR	A	166	8.715	14.114	-3.735	1.00	18.39	O
ATOM	1309	N	GLU	A	167	8.978	18.778	-2.677	1.00	20.35	N
ATOM	1310	CA	GLU	A	167	8.511	20.145	-2.670	1.00	20.28	C
ATOM	1311	C	GLU	A	167	9.603	21.010	-2.066	1.00	18.79	C
ATOM	1312	O	GLU	A	167	10.110	21.924	-2.699	1.00	19.11	O
ATOM	1313	CB	GLU	A	167	7.243	20.228	-1.846	1.00	20.88	C
ATOM	1314	CG	GLU	A	167	6.759	21.612	-1.587	1.00	26.70	C
ATOM	1315	CD	GLU	A	167	5.856	21.656	-0.377	1.00	29.94	C
ATOM	1316	OE1	GLU	A	167	4.740	21.074	-0.432	1.00	32.74	O
ATOM	1317	OE2	GLU	A	167	6.300	22.200	0.658	1.00	32.31	O1-
ATOM	1318	N	CYS	A	168	10.066	20.636	-0.895	1.00	19.65	N
ATOM	1319	CA	CYS	A	168	10.978	21.490	-0.205	1.00	22.23	C
ATOM	1320	C	CYS	A	168	12.291	20.781	-0.033	1.00	25.31	C
ATOM	1321	O	CYS	A	168	12.626	20.370	1.077	1.00	29.59	O
ATOM	1322	CB	CYS	A	168	10.395	21.919	1.147	1.00	22.76	C
ATOM	1323	SG	CYS	A	168	11.603	22.432	2.429	1.00	26.02	S
ATOM	1324	N	CYS	A	169	13.009	20.566	-1.136	1.00	24.80	N
ATOM	1325	CA	CYS	A	169	14.459	20.798	-1.101	1.00	27.27	C
ATOM	1326	C	CYS	A	169	14.569	22.293	-1.412	1.00	26.49	C
ATOM	1327	O	CYS	A	169	15.379	23.015	-0.802	1.00	26.20	O
ATOM	1328	CB	CYS	A	169	15.190	19.988	-2.194	1.00	30.98	C
ATOM	1329	SG	CYS	A	169	15.841	18.333	-1.718	1.00	33.25	S
ATOM	1330	N	GLN	A	170	13.492	22.748	-2.059	1.00	26.22	N
ATOM	1331	CA	GLN	A	170	13.353	23.983	-2.844	1.00	25.86	C
ATOM	1332	C	GLN	A	170	13.998	25.203	-2.207	1.00	26.18	C
ATOM	1333	O	GLN	A	170	15.202	25.251	-2.033	1.00	30.02	O
ATOM	1334	CB	GLN	A	170	11.842	24.269	-3.085	1.00	25.67	C
ATOM	1335	CG	GLN	A	170	11.397	24.518	-4.531	1.00	21.68	C
ATOM	1336	CD	GLN	A	170	11.373	23.257	-5.393	1.00	20.70	C
ATOM	1337	NE2	GLN	A	170	12.540	22.814	-5.816	1.00	20.61	N
ATOM	1338	OE1	GLN	A	170	10.321	22.737	-5.727	1.00	16.98	O
ATOM	1339	N	ALA	A	171	13.192	26.161	-1.785	1.00	25.17	N
ATOM	1340	CA	ALA	A	171	13.718	27.493	-1.587	1.00	22.37	C
ATOM	1341	C	ALA	A	171	14.440	27.547	-0.279	1.00	23.13	C
ATOM	1342	O	ALA	A	171	15.176	28.489	-0.017	1.00	25.63	O
ATOM	1343	CB	ALA	A	171	12.619	28.501	-1.608	1.00	24.39	C
ATOM	1344	N	ALA	A	172	14.170	26.578	0.583	1.00	22.80	N
ATOM	1345	CA	ALA	A	172	14.686	26.649	1.935	1.00	23.27	C
ATOM	1346	C	ALA	A	172	15.967	25.845	2.129	1.00	23.80	C

```
ATOM   1347  O   ALA A 172      16.315  25.519   3.270  1.00 21.53           O
ATOM   1348  CB  ALA A 172      13.634  26.207   2.915  1.00 21.51           C
ATOM   1349  N   ASP A 173      16.684  25.569   1.031  1.00 25.42           N
ATOM   1350  CA  ASP A 173      17.901  24.751   1.088  1.00 28.45           C
ATOM   1351  C   ASP A 173      17.612  23.425   1.779  1.00 30.18           C
ATOM   1352  O   ASP A 173      18.403  22.987   2.608  1.00 31.67           O
ATOM   1353  CB  ASP A 173      18.973  25.462   1.906  1.00 29.65           C
ATOM   1354  CG  ASP A 173      19.953  26.208   1.057  1.00 30.99           C
ATOM   1355  OD1 ASP A 173      19.492  27.029   0.227  1.00 32.55           O
ATOM   1356  OD2 ASP A 173      21.176  25.999   1.265  1.00 32.46           O1-
ATOM   1357  N   LYS A 174      16.377  22.957   1.652  1.00 30.45           N
ATOM   1358  CA  LYS A 174      15.795  21.965   2.549  1.00 29.08           C
ATOM   1359  C   LYS A 174      15.766  22.368   4.036  1.00 28.75           C
ATOM   1360  O   LYS A 174      15.050  21.761   4.840  1.00 29.82           O
ATOM   1361  CB  LYS A 174      16.501  20.618   2.369  1.00 29.28           C
ATOM   1362  CG  LYS A 174      17.744  20.415   3.213  1.00 28.90           C
ATOM   1363  CD  LYS A 174      17.508  19.425   4.326  1.00 28.34           C
ATOM   1364  CE  LYS A 174      18.696  19.385   5.263  1.00 28.62           C
ATOM   1365  NZ  LYS A 174      19.405  18.085   5.065  1.00 31.78           N1+
ATOM   1366  N   ALA A 175      16.507  23.402   4.401  1.00 26.56           N
ATOM   1367  CA  ALA A 175      16.781  23.678   5.795  1.00 27.02           C
ATOM   1368  C   ALA A 175      15.532  23.529   6.661  1.00 27.16           C
ATOM   1369  O   ALA A 175      15.266  22.457   7.181  1.00 27.67           O
ATOM   1370  CB  ALA A 175      17.359  25.092   5.929  1.00 29.25           C
ATOM   1371  N   ALA A 176      14.672  24.542   6.602  1.00 26.48           N
ATOM   1372  CA  ALA A 176      13.614  24.748   7.567  1.00 20.96           C
ATOM   1373  C   ALA A 176      12.325  24.254   6.976  1.00 21.35           C
ATOM   1374  O   ALA A 176      11.676  23.432   7.557  1.00 22.19           O
ATOM   1375  CB  ALA A 176      13.506  26.217   7.899  1.00 20.23           C
ATOM   1376  N   CYS A 177      11.973  24.711   5.784  1.00 22.81           N
ATOM   1377  CA  CYS A 177      10.706  24.305   5.168  1.00 25.45           C
ATOM   1378  C   CYS A 177      10.548  22.808   4.974  1.00 25.72           C
ATOM   1379  O   CYS A 177       9.532  22.387   4.413  1.00 25.67           O
ATOM   1380  CB  CYS A 177      10.498  24.980   3.800  1.00 28.47           C
ATOM   1381  SG  CYS A 177      11.590  24.464   2.414  1.00 27.41           S
ATOM   1382  N   LEU A 178      11.645  22.068   5.169  1.00 25.62           N
ATOM   1383  CA  LEU A 178      11.655  20.608   5.049  1.00 25.90           C
ATOM   1384  C   LEU A 178      11.677  19.970   6.433  1.00 27.85           C
ATOM   1385  O   LEU A 178      10.620  19.599   6.972  1.00 31.37           O
ATOM   1386  CB  LEU A 178      12.880  20.117   4.255  1.00 23.18           C
ATOM   1387  CG  LEU A 178      12.918  18.765   3.515  1.00 22.71           C
ATOM   1388  CD1 LEU A 178      14.366  18.315   3.373  1.00 15.42           C
ATOM   1389  CD2 LEU A 178      12.073  17.684   4.217  1.00 19.52           C
ATOM   1390  N   LEU A 179      12.858  19.897   7.046  1.00 24.44           N
ATOM   1391  CA  LEU A 179      13.079  18.960   8.143  1.00 23.00           C
ATOM   1392  C   LEU A 179      12.215  19.038   9.449  1.00 23.84           C
ATOM   1393  O   LEU A 179      12.326  18.165  10.315  1.00 22.67           O
ATOM   1394  CB  LEU A 179      14.588  18.940   8.460  1.00 21.35           C
ATOM   1395  CG  LEU A 179      15.248  19.579   9.697  1.00 23.42           C
ATOM   1396  CD1 LEU A 179      16.763  19.387   9.589  1.00 22.06           C
ATOM   1397  CD2 LEU A 179      14.898  21.062   9.845  1.00 18.81           C
ATOM   1398  N   PRO A 180      11.301  20.026   9.578  1.00 24.90           N
ATOM   1399  CA  PRO A 180      10.275  19.957  10.637  1.00 25.32           C
ATOM   1400  C   PRO A 180       9.020  19.164  10.271  1.00 25.47           C
ATOM   1401  O   PRO A 180       8.078  19.034  11.074  1.00 24.17           O
ATOM   1402  CB  PRO A 180       9.956  21.425  10.914  1.00 25.04           C
ATOM   1403  CG  PRO A 180      10.217  22.072   9.597  1.00 22.93           C
ATOM   1404  CD  PRO A 180      11.523  21.427   9.210  1.00 24.99           C
ATOM   1405  N   LYS A 181       9.018  18.641   9.054  1.00 24.23           N
ATOM   1406  CA  LYS A 181       8.168  17.507   8.701  1.00 23.92           C
ATOM   1407  C   LYS A 181       9.027  16.222   8.798  1.00 22.82           C
ATOM   1408  O   LYS A 181       8.584  15.135   8.454  1.00 20.58           O
ATOM   1409  CB  LYS A 181       7.615  17.711   7.286  1.00 25.23           C
ATOM   1410  CG  LYS A 181       7.840  19.139   6.771  1.00 23.39           C
ATOM   1411  CD  LYS A 181       7.528  19.289   5.312  1.00 21.94           C
ATOM   1412  CE  LYS A 181       6.048  19.541   5.120  1.00 22.34           C
ATOM   1413  NZ  LYS A 181       5.471  20.266   6.296  1.00 24.22           N1+
ATOM   1414  N   LEU A 182      10.231  16.372   9.354  1.00 21.40           N
ATOM   1415  CA  LEU A 182      11.159  15.277   9.612  1.00 18.92           C
ATOM   1416  C   LEU A 182      11.513  15.303  11.107  1.00 18.56           C
ATOM   1417  O   LEU A 182      12.482  14.685  11.539  1.00 16.25           O
```

ATOM	1418	CB	LEU	A	182	12.431	15.475	8.777	1.00	19.22	C
ATOM	1419	CG	LEU	A	182	12.869	14.556	7.621	1.00	20.67	C
ATOM	1420	CD1	LEU	A	182	12.740	13.115	8.064	1.00	22.41	C
ATOM	1421	CD2	LEU	A	182	12.070	14.796	6.342	1.00	16.92	C
ATOM	1422	N	ASP	A	183	10.857	16.186	11.849	1.00	19.20	N
ATOM	1423	CA	ASP	A	183	10.839	16.085	13.305	1.00	21.07	C
ATOM	1424	C	ASP	A	183	9.608	15.238	13.640	1.00	21.27	C
ATOM	1425	O	ASP	A	183	9.693	14.015	13.759	1.00	20.65	O
ATOM	1426	CB	ASP	A	183	10.731	17.485	13.960	1.00	22.49	C
ATOM	1427	CG	ASP	A	183	12.064	18.279	13.924	1.00	24.78	C
ATOM	1428	OD1	ASP	A	183	12.922	18.040	14.805	1.00	22.96	O
ATOM	1429	OD2	ASP	A	183	12.261	19.129	13.018	1.00	24.01	O1-
ATOM	1430	N	GLU	A	184	8.443	15.856	13.510	1.00	20.83	N
ATOM	1431	CA	GLU	A	184	7.190	15.210	13.853	1.00	20.58	C
ATOM	1432	C	GLU	A	184	7.081	13.767	13.329	1.00	19.58	C
ATOM	1433	O	GLU	A	184	7.119	12.822	14.118	1.00	18.60	O
ATOM	1434	CB	GLU	A	184	6.021	16.067	13.356	1.00	21.76	C
ATOM	1435	CG	GLU	A	184	4.698	15.723	13.997	1.00	25.58	C
ATOM	1436	CD	GLU	A	184	3.531	15.959	13.071	1.00	27.71	C
ATOM	1437	OE1	GLU	A	184	3.257	17.145	12.760	1.00	26.41	O
ATOM	1438	OE2	GLU	A	184	2.892	14.954	12.659	1.00	29.24	O1-
ATOM	1439	N	LEU	A	185	7.114	13.580	12.015	1.00	19.28	N
ATOM	1440	CA	LEU	A	185	6.872	12.254	11.438	1.00	21.28	C
ATOM	1441	C	LEU	A	185	8.027	11.229	11.664	1.00	21.48	C
ATOM	1442	O	LEU	A	185	8.321	10.422	10.797	1.00	20.44	O
ATOM	1443	CB	LEU	A	185	6.549	12.387	9.930	1.00	21.87	C
ATOM	1444	CG	LEU	A	185	5.217	13.009	9.440	1.00	24.75	C
ATOM	1445	CD1	LEU	A	185	5.400	14.429	8.858	1.00	21.49	C
ATOM	1446	CD2	LEU	A	185	4.605	12.100	8.376	1.00	22.23	C
ATOM	1447	N	ARG	A	186	8.728	11.323	12.789	1.00	21.60	N
ATOM	1448	CA	ARG	A	186	9.692	10.304	13.191	1.00	20.65	C
ATOM	1449	C	ARG	A	186	9.175	9.608	14.448	1.00	23.63	C
ATOM	1450	O	ARG	A	186	9.353	8.393	14.626	1.00	24.05	O
ATOM	1451	CB	ARG	A	186	11.036	10.941	13.514	1.00	21.81	C
ATOM	1452	CG	ARG	A	186	11.149	11.524	14.926	1.00	20.40	C
ATOM	1453	CD	ARG	A	186	12.527	11.273	15.525	1.00	22.73	C
ATOM	1454	NE	ARG	A	186	12.739	9.855	15.758	1.00	24.25	N
ATOM	1455	CZ	ARG	A	186	13.468	9.358	16.750	1.00	26.77	C
ATOM	1456	NH1	ARG	A	186	14.043	10.164	17.638	1.00	26.94	N1+
ATOM	1457	NH2	ARG	A	186	13.561	8.042	16.896	1.00	28.22	N
ATOM	1458	N	ASP	A	187	8.589	10.399	15.349	1.00	23.17	N
ATOM	1459	CA	ASP	A	187	7.844	9.858	16.463	1.00	20.77	C
ATOM	1460	C	ASP	A	187	6.644	9.094	15.937	1.00	18.87	C
ATOM	1461	O	ASP	A	187	6.412	7.993	16.382	1.00	18.84	O
ATOM	1462	CB	ASP	A	187	7.385	10.972	17.388	1.00	23.77	C
ATOM	1463	CG	ASP	A	187	8.523	11.556	18.208	1.00	26.43	C
ATOM	1464	OD1	ASP	A	187	8.918	10.937	19.241	1.00	27.86	O
ATOM	1465	OD2	ASP	A	187	8.981	12.669	17.847	1.00	26.15	O1-
ATOM	1466	N	GLU	A	188	6.000	9.594	14.880	1.00	18.08	N
ATOM	1467	CA	GLU	A	188	4.861	8.894	14.247	1.00	18.47	C
ATOM	1468	C	GLU	A	188	5.280	7.641	13.475	1.00	18.83	C
ATOM	1469	O	GLU	A	188	4.440	6.970	12.890	1.00	18.98	O
ATOM	1470	CB	GLU	A	188	4.074	9.840	13.299	1.00	16.29	C
ATOM	1471	CG	GLU	A	188	2.648	9.362	12.934	1.00	12.67	C
ATOM	1472	CD	GLU	A	188	1.770	10.425	12.231	1.00	16.08	C
ATOM	1473	OE1	GLU	A	188	2.195	11.591	12.086	1.00	16.21	O
ATOM	1474	OE2	GLU	A	188	0.607	10.109	11.882	1.00	14.17	O1-
ATOM	1475	N	GLY	A	189	6.587	7.418	13.355	1.00	20.49	N
ATOM	1476	CA	GLY	A	189	7.098	6.195	12.753	1.00	19.35	C
ATOM	1477	C	GLY	A	189	7.221	5.173	13.857	1.00	18.26	C
ATOM	1478	O	GLY	A	189	6.532	4.161	13.867	1.00	18.56	O
ATOM	1479	N	LYS	A	190	7.964	5.536	14.886	1.00	20.08	N
ATOM	1480	CA	LYS	A	190	7.903	4.816	16.152	1.00	22.77	C
ATOM	1481	C	LYS	A	190	6.463	4.574	16.647	1.00	22.46	C
ATOM	1482	O	LYS	A	190	6.120	3.434	16.940	1.00	24.59	O
ATOM	1483	CB	LYS	A	190	8.710	5.555	17.232	1.00	24.21	C
ATOM	1484	CG	LYS	A	190	10.186	5.699	16.889	1.00	27.27	C
ATOM	1485	CD	LYS	A	190	11.034	5.974	18.115	1.00	31.27	C
ATOM	1486	CE	LYS	A	190	10.959	7.431	18.550	1.00	32.12	C
ATOM	1487	NZ	LYS	A	190	12.010	7.723	19.570	1.00	31.95	N1+
ATOM	1488	N	ALA	A	191	5.594	5.589	16.605	1.00	20.34	N

```
ATOM   1489  CA  ALA A 191       4.280   5.495  17.239  1.00 17.99           C
ATOM   1490  C   ALA A 191       3.575   4.269  16.726  1.00 17.52           C
ATOM   1491  O   ALA A 191       3.591   3.250  17.391  1.00 21.21           O
ATOM   1492  CB  ALA A 191       3.442   6.712  16.971  1.00 17.43           C
ATOM   1493  N   SER A 192       3.200   4.269  15.458  1.00 16.48           N
ATOM   1494  CA  SER A 192       2.445   3.151  14.932  1.00 15.33           C
ATOM   1495  C   SER A 192       3.246   1.855  14.918  1.00 17.13           C
ATOM   1496  O   SER A 192       2.673   0.771  15.078  1.00 18.89           O
ATOM   1497  CB  SER A 192       1.943   3.446  13.538  1.00 14.90           C
ATOM   1498  OG  SER A 192       1.128   2.380  13.116  1.00 19.59           O
ATOM   1499  N   SER A 193       4.567   1.946  14.777  1.00 13.75           N
ATOM   1500  CA  SER A 193       5.377   0.738  14.665  1.00 11.93           C
ATOM   1501  C   SER A 193       5.408   0.064  15.996  1.00 10.26           C
ATOM   1502  O   SER A 193       6.136  -0.909  16.173  1.00  8.48           O
ATOM   1503  CB  SER A 193       6.802   1.089  14.258  1.00 15.39           C
ATOM   1504  OG  SER A 193       7.526   1.680  15.322  1.00 15.80           O
ATOM   1505  N   ALA A 194       4.916   0.833  16.965  1.00 13.13           N
ATOM   1506  CA  ALA A 194       4.875   0.494  18.384  1.00 14.96           C
ATOM   1507  C   ALA A 194       3.436   0.431  18.859  1.00 12.28           C
ATOM   1508  O   ALA A 194       3.117  -0.360  19.733  1.00 16.44           O
ATOM   1509  CB  ALA A 194       5.647   1.543  19.205  1.00 14.37           C
ATOM   1510  N   LYS A 195       2.560   1.162  18.186  1.00  8.87           N
ATOM   1511  CA  LYS A 195       1.129   1.081  18.415  1.00 10.05           C
ATOM   1512  C   LYS A 195       0.656  -0.181  17.755  1.00  9.99           C
ATOM   1513  O   LYS A 195      -0.535  -0.421  17.682  1.00 12.22           O
ATOM   1514  CB  LYS A 195       0.390   2.267  17.767  1.00 12.99           C
ATOM   1515  CG  LYS A 195       0.478   3.588  18.531  1.00 15.37           C
ATOM   1516  CD  LYS A 195       0.179   4.787  17.664  1.00 16.04           C
ATOM   1517  CE  LYS A 195      -1.184   5.396  17.995  1.00 19.24           C
ATOM   1518  NZ  LYS A 195      -1.397   6.691  17.246  1.00 20.94           N1+
ATOM   1519  N   GLN A 196       1.586  -0.882  17.112  1.00 13.42           N
ATOM   1520  CA  GLN A 196       1.302  -2.120  16.370  1.00 13.91           C
ATOM   1521  C   GLN A 196       1.790  -3.377  17.084  1.00 13.37           C
ATOM   1522  O   GLN A 196       0.998  -4.282  17.270  1.00 15.65           O
ATOM   1523  CB  GLN A 196       1.923  -2.071  14.971  1.00 13.77           C
ATOM   1524  CG  GLN A 196       1.763  -3.343  14.183  1.00 15.75           C
ATOM   1525  CD  GLN A 196       0.630  -3.266  13.192  1.00 20.39           C
ATOM   1526  NE2 GLN A 196      -0.021  -2.104  13.132  1.00 21.97           N
ATOM   1527  OE1 GLN A 196       0.344  -4.228  12.470  1.00 19.31           O
ATOM   1528  N   ARG A 197       3.063  -3.437  17.495  1.00 10.81           N
ATOM   1529  CA  ARG A 197       3.666  -4.683  17.989  1.00 10.56           C
ATOM   1530  C   ARG A 197       2.685  -5.310  18.950  1.00 12.83           C
ATOM   1531  O   ARG A 197       2.476  -6.508  18.910  1.00 14.23           O
ATOM   1532  CB  ARG A 197       5.014  -4.404  18.679  1.00 11.17           C
ATOM   1533  CG  ARG A 197       5.808  -5.620  19.258  1.00 11.07           C
ATOM   1534  CD  ARG A 197       5.853  -6.866  18.353  1.00  8.47           C
ATOM   1535  NE  ARG A 197       6.520  -8.013  18.986  1.00  6.63           N
ATOM   1536  CZ  ARG A 197       5.931  -9.185  19.260  1.00  8.70           C
ATOM   1537  NH1 ARG A 197       4.618  -9.319  19.201  1.00  7.16           N1+
ATOM   1538  NH2 ARG A 197       6.645 -10.211  19.706  1.00  7.19           N
ATOM   1539  N   LEU A 198       1.906  -4.441  19.595  1.00 14.31           N
ATOM   1540  CA  LEU A 198       0.664  -4.783  20.286  1.00 15.62           C
ATOM   1541  C   LEU A 198      -0.305  -5.651  19.460  1.00 18.97           C
ATOM   1542  O   LEU A 198      -0.561  -6.800  19.835  1.00 22.05           O
ATOM   1543  CB  LEU A 198      -0.047  -3.501  20.714  1.00 12.42           C
ATOM   1544  CG  LEU A 198      -0.455  -3.324  22.174  1.00 13.86           C
ATOM   1545  CD1 LEU A 198      -1.936  -2.970  22.232  1.00 13.35           C
ATOM   1546  CD2 LEU A 198      -0.162  -4.573  22.987  1.00 10.76           C
ATOM   1547  N   LYS A 199      -0.925  -5.076  18.423  1.00 18.33           N
ATOM   1548  CA  LYS A 199      -1.898  -5.785  17.578  1.00 18.04           C
ATOM   1549  C   LYS A 199      -1.443  -7.160  17.098  1.00 19.50           C
ATOM   1550  O   LYS A 199      -2.262  -7.986  16.724  1.00 20.23           O
ATOM   1551  CB  LYS A 199      -2.249  -4.949  16.355  1.00 18.92           C
ATOM   1552  CG  LYS A 199      -3.608  -4.261  16.389  1.00 18.14           C
ATOM   1553  CD  LYS A 199      -3.598  -2.944  15.594  1.00 13.47           C
ATOM   1554  CE  LYS A 199      -2.700  -1.907  16.227  1.00  8.90           C
ATOM   1555  NZ  LYS A 199      -2.448  -2.218  17.659  1.00  9.83           N1+
ATOM   1556  N   CYS A 200      -0.140  -7.375  17.001  1.00 21.92           N
ATOM   1557  CA  CYS A 200       0.372  -8.717  16.755  1.00 23.15           C
ATOM   1558  C   CYS A 200       0.459  -9.453  18.071  1.00 25.39           C
ATOM   1559  O   CYS A 200       0.080 -10.614  18.142  1.00 29.62           O
```

```
ATOM   1560  CB  CYS A 200       1.762  -8.682  16.118  1.00 22.40           C
ATOM   1561  SG  CYS A 200       1.793  -7.924  14.469  1.00 21.69           S
ATOM   1562  N   ALA A 201       0.949  -8.778  19.114  1.00 26.47           N
ATOM   1563  CA  ALA A 201       1.139  -9.381  20.440  1.00 23.93           C
ATOM   1564  C   ALA A 201      -0.165  -9.943  20.993  1.00 21.85           C
ATOM   1565  O   ALA A 201      -0.208 -11.107  21.360  1.00 20.07           O
ATOM   1566  CB  ALA A 201       1.740  -8.371  21.408  1.00 24.67           C
ATOM   1567  N   SER A 202      -1.245  -9.173  20.919  1.00 20.21           N
ATOM   1568  CA  SER A 202      -2.584  -9.760  20.944  1.00 22.62           C
ATOM   1569  C   SER A 202      -2.608 -11.018  20.056  1.00 23.67           C
ATOM   1570  O   SER A 202      -2.798 -12.128  20.544  1.00 24.94           O
ATOM   1571  CB  SER A 202      -3.644  -8.779  20.400  1.00 23.40           C
ATOM   1572  OG  SER A 202      -3.857  -7.642  21.221  1.00 23.09           O
ATOM   1573  N   LEU A 203      -2.326 -10.847  18.765  1.00 24.01           N
ATOM   1574  CA  LEU A 203      -2.674 -11.850  17.762  1.00 22.30           C
ATOM   1575  C   LEU A 203      -2.159 -13.281  17.940  1.00 20.69           C
ATOM   1576  O   LEU A 203      -2.961 -14.201  17.903  1.00 20.01           O
ATOM   1577  CB  LEU A 203      -2.320 -11.341  16.368  1.00 21.07           C
ATOM   1578  CG  LEU A 203      -3.523 -11.255  15.429  1.00 20.68           C
ATOM   1579  CD1 LEU A 203      -4.289 -12.558  15.445  1.00 22.29           C
ATOM   1580  CD2 LEU A 203      -4.410 -10.140  15.868  1.00 20.27           C
ATOM   1581  N   GLN A 204      -0.843 -13.487  17.919  1.00 20.66           N
ATOM   1582  CA  GLN A 204      -0.277 -14.834  18.099  1.00 23.10           C
ATOM   1583  C   GLN A 204       1.062 -14.932  18.874  1.00 24.84           C
ATOM   1584  O   GLN A 204       1.816 -15.917  18.736  1.00 25.14           O
ATOM   1585  CB  GLN A 204      -0.135 -15.567  16.762  1.00 22.96           C
ATOM   1586  CG  GLN A 204      -0.633 -14.814  15.556  1.00 24.32           C
ATOM   1587  CD  GLN A 204       0.297 -14.952  14.367  1.00 23.07           C
ATOM   1588  NE2 GLN A 204       0.408 -13.888  13.570  1.00 22.46           N
ATOM   1589  OE1 GLN A 204       0.920 -15.990  14.175  1.00 21.96           O
ATOM   1590  N   LYS A 205       1.345 -13.922  19.695  1.00 21.97           N
ATOM   1591  CA  LYS A 205       2.050 -14.152  20.952  1.00 18.40           C
ATOM   1592  C   LYS A 205       0.998 -14.276  22.063  1.00 14.76           C
ATOM   1593  O   LYS A 205       1.311 -14.599  23.192  1.00 13.45           O
ATOM   1594  CB  LYS A 205       3.020 -13.007  21.232  1.00 19.14           C
ATOM   1595  CG  LYS A 205       2.841 -12.327  22.558  1.00 18.87           C
ATOM   1596  CD  LYS A 205       3.969 -12.710  23.485  1.00 21.97           C
ATOM   1597  CE  LYS A 205       5.126 -11.720  23.351  1.00 23.57           C
ATOM   1598  NZ  LYS A 205       6.410 -12.330  23.789  1.00 23.75           N1+
ATOM   1599  N   PHE A 206      -0.253 -14.040  21.707  1.00 12.24           N
ATOM   1600  CA  PHE A 206      -1.395 -14.291  22.578  1.00 13.28           C
ATOM   1601  C   PHE A 206      -2.440 -14.872  21.656  1.00 13.87           C
ATOM   1602  O   PHE A 206      -2.389 -14.638  20.459  1.00 15.15           O
ATOM   1603  CB  PHE A 206      -1.945 -12.972  23.110  1.00 14.11           C
ATOM   1604  CG  PHE A 206      -1.995 -12.870  24.594  1.00  8.89           C
ATOM   1605  CD1 PHE A 206      -0.961 -12.280  25.264  1.00  7.13           C
ATOM   1606  CD2 PHE A 206      -3.173 -13.071  25.251  1.00  6.33           C
ATOM   1607  CE1 PHE A 206      -1.102 -11.880  26.508  1.00  7.41           C
ATOM   1608  CE2 PHE A 206      -3.317 -12.670  26.507  1.00  8.37           C
ATOM   1609  CZ  PHE A 206      -2.278 -12.066  27.139  1.00  9.60           C
ATOM   1610  N   GLY A 207      -3.502 -15.416  22.230  1.00 14.43           N
ATOM   1611  CA  GLY A 207      -4.553 -16.003  21.425  1.00 14.40           C
ATOM   1612  C   GLY A 207      -5.095 -15.066  20.366  1.00 15.46           C
ATOM   1613  O   GLY A 207      -5.411 -13.904  20.628  1.00 16.20           O
ATOM   1614  N   GLU A 208      -5.330 -15.625  19.192  1.00 15.63           N
ATOM   1615  CA  GLU A 208      -5.998 -14.904  18.125  1.00 18.59           C
ATOM   1616  C   GLU A 208      -7.446 -14.492  18.494  1.00 19.71           C
ATOM   1617  O   GLU A 208      -7.997 -13.540  17.917  1.00 21.56           O
ATOM   1618  CB  GLU A 208      -5.971 -15.761  16.845  1.00 18.16           C
ATOM   1619  CG  GLU A 208      -7.133 -16.747  16.691  1.00 18.76           C
ATOM   1620  CD  GLU A 208      -8.245 -16.193  15.814  1.00 19.03           C
ATOM   1621  OE1 GLU A 208      -8.448 -14.963  15.793  1.00 18.38           O
ATOM   1622  OE2 GLU A 208      -8.860 -16.979  15.069  1.00 22.62           O1-
ATOM   1623  N   ARG A 209      -8.083 -15.253  19.389  1.00 18.66           N
ATOM   1624  CA  ARG A 209      -9.477 -15.021  19.769  1.00 17.70           C
ATOM   1625  C   ARG A 209      -9.626 -13.656  20.423  1.00 18.80           C
ATOM   1626  O   ARG A 209     -10.580 -12.930  20.170  1.00 17.41           O
ATOM   1627  CB  ARG A 209      -9.939 -16.095  20.751  1.00 17.78           C
ATOM   1628  CG  ARG A 209     -11.325 -16.650  20.479  1.00 22.53           C
ATOM   1629  CD  ARG A 209     -12.149 -15.748  19.536  1.00 23.84           C
ATOM   1630  NE  ARG A 209     -12.523 -14.506  20.204  1.00 25.57           N
```

```
ATOM   1631  CZ  ARG A 209     -13.644 -13.825  19.989  1.00 25.17           C
ATOM   1632  NH1 ARG A 209     -14.470 -14.180  19.011  1.00 27.58           N1+
ATOM   1633  NH2 ARG A 209     -13.900 -12.739  20.714  1.00 24.46           N
ATOM   1634  N   ALA A 210      -8.623 -13.292  21.210  1.00 22.42           N
ATOM   1635  CA  ALA A 210      -8.644 -12.078  21.995  1.00 25.29           C
ATOM   1636  C   ALA A 210      -8.974 -10.918  21.071  1.00 27.14           C
ATOM   1637  O   ALA A 210     -10.042 -10.313  21.208  1.00 29.89           O
ATOM   1638  CB  ALA A 210      -7.289 -11.867  22.666  1.00 24.38           C
ATOM   1639  N   PHE A 211      -8.173 -10.760  20.012  1.00 26.77           N
ATOM   1640  CA  PHE A 211      -8.289  -9.629  19.081  1.00 23.38           C
ATOM   1641  C   PHE A 211      -9.721  -9.257  18.741  1.00 21.80           C
ATOM   1642  O   PHE A 211     -10.079  -8.090  18.838  1.00 18.92           O
ATOM   1643  CB  PHE A 211      -7.543  -9.926  17.788  1.00 25.16           C
ATOM   1644  CG  PHE A 211      -7.077  -8.695  17.057  1.00 26.16           C
ATOM   1645  CD1 PHE A 211      -6.020  -7.951  17.542  1.00 25.15           C
ATOM   1646  CD2 PHE A 211      -7.562  -8.404  15.786  1.00 25.67           C
ATOM   1647  CE1 PHE A 211      -5.440  -6.965  16.771  1.00 23.64           C
ATOM   1648  CE2 PHE A 211      -6.994  -7.414  15.017  1.00 23.27           C
ATOM   1649  CZ  PHE A 211      -5.925  -6.698  15.511  1.00 25.63           C
ATOM   1650  N   LYS A 212     -10.536 -10.244  18.363  1.00 20.64           N
ATOM   1651  CA  LYS A 212     -11.955 -10.003  18.096  1.00 20.36           C
ATOM   1652  C   LYS A 212     -12.497  -9.141  19.218  1.00 22.63           C
ATOM   1653  O   LYS A 212     -12.772  -7.964  19.004  1.00 24.04           O
ATOM   1654  CB  LYS A 212     -12.748 -11.308  18.044  1.00 19.99           C
ATOM   1655  CG  LYS A 212     -13.261 -11.723  16.651  1.00 22.19           C
ATOM   1656  CD  LYS A 212     -12.319 -12.755  15.969  1.00 17.63           C
ATOM   1657  CE  LYS A 212     -13.061 -13.725  15.077  1.00 14.45           C
ATOM   1658  NZ  LYS A 212     -14.268 -13.106  14.457  1.00 14.02           N1+
ATOM   1659  N   ALA A 213     -12.384  -9.642  20.446  1.00 22.31           N
ATOM   1660  CA  ALA A 213     -12.827  -8.900  21.614  1.00 21.34           C
ATOM   1661  C   ALA A 213     -12.149  -7.537  21.705  1.00 21.10           C
ATOM   1662  O   ALA A 213     -12.763  -6.552  22.098  1.00 23.45           O
ATOM   1663  CB  ALA A 213     -12.569  -9.696  22.871  1.00 20.73           C
ATOM   1664  N   TRP A 214     -10.922  -7.437  21.240  1.00 19.46           N
ATOM   1665  CA  TRP A 214     -10.271  -6.142  21.224  1.00 19.15           C
ATOM   1666  C   TRP A 214     -11.048  -5.224  20.314  1.00 18.85           C
ATOM   1667  O   TRP A 214     -11.516  -4.175  20.734  1.00 20.49           O
ATOM   1668  CB  TRP A 214      -8.870  -6.296  20.686  1.00 22.36           C
ATOM   1669  CG  TRP A 214      -7.861  -5.482  21.350  1.00 24.56           C
ATOM   1670  CD1 TRP A 214      -6.926  -5.942  22.246  1.00 26.33           C
ATOM   1671  CD2 TRP A 214      -7.377  -4.211  20.888  1.00 25.12           C
ATOM   1672  CE2 TRP A 214      -6.120  -4.005  21.484  1.00 28.85           C
ATOM   1673  CE3 TRP A 214      -7.863  -3.267  19.975  1.00 21.79           C
ATOM   1674  NE1 TRP A 214      -5.867  -5.073  22.305  1.00 31.06           N
ATOM   1675  CZ2 TRP A 214      -5.341  -2.887  21.189  1.00 28.30           C
ATOM   1676  CZ3 TRP A 214      -7.100  -2.178  19.689  1.00 18.16           C
ATOM   1677  CH2 TRP A 214      -5.852  -1.991  20.287  1.00 25.36           C
ATOM   1678  N   ALA A 215     -11.279  -5.677  19.092  1.00 18.46           N
ATOM   1679  CA  ALA A 215     -11.815  -4.819  18.056  1.00 18.34           C
ATOM   1680  C   ALA A 215     -13.346  -4.685  18.072  1.00 19.69           C
ATOM   1681  O   ALA A 215     -13.853  -3.636  17.695  1.00 19.89           O
ATOM   1682  CB  ALA A 215     -11.337  -5.295  16.705  1.00 17.27           C
ATOM   1683  N   VAL A 216     -14.074  -5.711  18.532  1.00 19.07           N
ATOM   1684  CA  VAL A 216     -15.533  -5.613  18.768  1.00 18.57           C
ATOM   1685  C   VAL A 216     -15.801  -4.635  19.911  1.00 16.42           C
ATOM   1686  O   VAL A 216     -16.854  -4.013  19.985  1.00 16.02           O
ATOM   1687  CB  VAL A 216     -16.199  -6.979  19.159  1.00 18.60           C
ATOM   1688  CG1 VAL A 216     -17.658  -6.980  18.765  1.00 18.91           C
ATOM   1689  CG2 VAL A 216     -15.530  -8.129  18.488  1.00 18.58           C
ATOM   1690  N   ALA A 217     -14.860  -4.555  20.837  1.00 15.89           N
ATOM   1691  CA  ALA A 217     -14.811  -3.444  21.767  1.00 17.11           C
ATOM   1692  C   ALA A 217     -14.553  -2.125  21.050  1.00 17.79           C
ATOM   1693  O   ALA A 217     -15.155  -1.114  21.413  1.00 20.39           O
ATOM   1694  CB  ALA A 217     -13.734  -3.679  22.827  1.00 17.06           C
ATOM   1695  N   ARG A 218     -13.664  -2.114  20.050  1.00 16.98           N
ATOM   1696  CA  ARG A 218     -13.190  -0.845  19.494  1.00 14.92           C
ATOM   1697  C   ARG A 218     -13.902  -0.275  18.310  1.00 13.69           C
ATOM   1698  O   ARG A 218     -14.093   0.920  18.241  1.00 17.63           O
ATOM   1699  CB  ARG A 218     -11.717  -0.886  19.162  1.00 16.75           C
ATOM   1700  CG  ARG A 218     -11.031   0.429  19.482  1.00 20.34           C
ATOM   1701  CD  ARG A 218      -9.529   0.272  19.350  1.00 28.41           C
```

ATOM	1702	NE	ARG	A	218	-8.742	0.955	20.381	1.00	31.43	N
ATOM	1703	CZ	ARG	A	218	-7.410	0.999	20.357	1.00	31.18	C
ATOM	1704	NH1	ARG	A	218	-6.781	0.627	19.249	1.00	33.54	N1+
ATOM	1705	NH2	ARG	A	218	-6.709	1.463	21.392	1.00	28.29	N
ATOM	1706	N	LEU	A	219	-14.188	-1.074	17.304	1.00	13.67	N
ATOM	1707	CA	LEU	A	219	-14.863	-0.525	16.134	1.00	13.57	C
ATOM	1708	C	LEU	A	219	-16.279	-0.119	16.523	1.00	13.16	C
ATOM	1709	O	LEU	A	219	-16.875	0.721	15.861	1.00	11.91	O
ATOM	1710	CB	LEU	A	219	-14.861	-1.538	14.977	1.00	12.34	C
ATOM	1711	CG	LEU	A	219	-13.507	-2.219	14.718	1.00	11.08	C
ATOM	1712	CD1	LEU	A	219	-13.701	-3.486	13.935	1.00	12.13	C
ATOM	1713	CD2	LEU	A	219	-12.585	-1.277	13.980	1.00	10.31	C
ATOM	1714	N	SER	A	220	-16.711	-0.572	17.704	1.00	13.60	N
ATOM	1715	CA	SER	A	220	-18.039	-0.287	18.266	1.00	13.96	C
ATOM	1716	C	SER	A	220	-18.006	0.971	19.116	1.00	13.33	C
ATOM	1717	O	SER	A	220	-19.027	1.613	19.324	1.00	12.54	O
ATOM	1718	CB	SER	A	220	-18.527	-1.462	19.127	1.00	15.85	C
ATOM	1719	OG	SER	A	220	-19.168	-2.479	18.357	1.00	16.62	O
ATOM	1720	N	GLN	A	221	-16.798	1.360	19.507	1.00	15.03	N
ATOM	1721	CA	GLN	A	221	-16.498	2.618	20.203	1.00	14.21	C
ATOM	1722	C	GLN	A	221	-16.470	3.821	19.238	1.00	15.97	C
ATOM	1723	O	GLN	A	221	-16.314	4.959	19.669	1.00	17.34	O
ATOM	1724	CB	GLN	A	221	-15.127	2.473	20.843	1.00	13.10	C
ATOM	1725	CG	GLN	A	221	-14.878	3.266	22.062	1.00	20.06	C
ATOM	1726	CD	GLN	A	221	-14.119	2.455	23.085	1.00	24.79	C
ATOM	1727	NE2	GLN	A	221	-14.716	2.285	24.265	1.00	27.31	N
ATOM	1728	OE1	GLN	A	221	-13.066	1.884	22.784	1.00	24.46	O
ATOM	1729	N	ARG	A	222	-16.391	3.530	17.937	1.00	16.06	N
ATOM	1730	CA	ARG	A	222	-16.293	4.545	16.884	1.00	14.67	C
ATOM	1731	C	ARG	A	222	-17.453	4.450	15.874	1.00	14.79	C
ATOM	1732	O	ARG	A	222	-17.942	5.455	15.360	1.00	16.58	O
ATOM	1733	CB	ARG	A	222	-14.951	4.423	16.141	1.00	13.32	C
ATOM	1734	CG	ARG	A	222	-13.933	3.526	16.803	1.00	10.78	C
ATOM	1735	CD	ARG	A	222	-12.562	4.147	16.820	1.00	14.99	C
ATOM	1736	NE	ARG	A	222	-11.738	3.584	17.887	1.00	16.71	N
ATOM	1737	CZ	ARG	A	222	-10.937	4.294	18.683	1.00	17.32	C
ATOM	1738	NH1	ARG	A	222	-10.806	5.607	18.507	1.00	16.06	N1+
ATOM	1739	NH2	ARG	A	222	-10.252	3.687	19.654	1.00	12.90	N
ATOM	1740	N	PHE	A	223	-17.957	3.250	15.647	1.00	14.56	N
ATOM	1741	CA	PHE	A	223	-19.111	3.084	14.792	1.00	12.35	C
ATOM	1742	C	PHE	A	223	-20.298	2.602	15.626	1.00	13.17	C
ATOM	1743	O	PHE	A	223	-20.916	1.601	15.272	1.00	12.50	O
ATOM	1744	CB	PHE	A	223	-18.820	2.042	13.706	1.00	15.57	C
ATOM	1745	CG	PHE	A	223	-17.648	2.373	12.788	1.00	18.20	C
ATOM	1746	CD1	PHE	A	223	-16.762	3.398	13.060	1.00	18.12	C
ATOM	1747	CD2	PHE	A	223	-17.389	1.571	11.689	1.00	19.72	C
ATOM	1748	CE1	PHE	A	223	-15.642	3.594	12.261	1.00	20.71	C
ATOM	1749	CE2	PHE	A	223	-16.274	1.765	10.896	1.00	18.61	C
ATOM	1750	CZ	PHE	A	223	-15.401	2.767	11.182	1.00	18.31	C
ATOM	1751	N	PRO	A	224	-20.667	3.323	16.717	1.00	14.94	N
ATOM	1752	CA	PRO	A	224	-21.635	2.860	17.714	1.00	14.17	C
ATOM	1753	C	PRO	A	224	-23.096	2.979	17.275	1.00	15.44	C
ATOM	1754	O	PRO	A	224	-23.942	3.488	18.004	1.00	16.00	O
ATOM	1755	CB	PRO	A	224	-21.344	3.757	18.900	1.00	13.07	C
ATOM	1756	CG	PRO	A	224	-21.074	5.010	18.284	1.00	14.26	C
ATOM	1757	CD	PRO	A	224	-20.226	4.679	17.073	1.00	17.21	C
ATOM	1758	N	LYS	A	225	-23.347	2.630	16.026	1.00	15.99	N
ATOM	1759	CA	LYS	A	225	-24.668	2.300	15.555	1.00	15.34	C
ATOM	1760	C	LYS	A	225	-24.515	1.109	14.603	1.00	20.08	C
ATOM	1761	O	LYS	A	225	-25.481	0.396	14.332	1.00	23.63	O
ATOM	1762	CB	LYS	A	225	-25.285	3.490	14.839	1.00	10.48	C
ATOM	1763	CG	LYS	A	225	-24.358	4.174	13.887	1.00	10.33	C
ATOM	1764	CD	LYS	A	225	-24.354	5.652	14.130	1.00	9.50	C
ATOM	1765	CE	LYS	A	225	-25.685	6.266	13.772	1.00	11.24	C
ATOM	1766	NZ	LYS	A	225	-25.779	7.709	14.196	1.00	13.92	N1+
ATOM	1767	N	ALA	A	226	-23.282	0.839	14.171	1.00	21.60	N
ATOM	1768	CA	ALA	A	226	-22.963	-0.387	13.446	1.00	22.74	C
ATOM	1769	C	ALA	A	226	-23.686	-1.533	14.133	1.00	23.96	C
ATOM	1770	O	ALA	A	226	-23.520	-1.710	15.331	1.00	28.06	O
ATOM	1771	CB	ALA	A	226	-21.474	-0.630	13.497	1.00	21.05	C
ATOM	1772	N	GLU	A	227	-24.595	-2.212	13.446	1.00	23.06	N

ATOM	1773	CA	GLU	A	227	-25.267	-3.340	14.079	1.00	20.89	C
ATOM	1774	C	GLU	A	227	-24.243	-4.476	14.307	1.00	18.38	C
ATOM	1775	O	GLU	A	227	-23.142	-4.455	13.748	1.00	11.59	O
ATOM	1776	CB	GLU	A	227	-26.507	-3.810	13.258	1.00	22.63	C
ATOM	1777	CG	GLU	A	227	-27.720	-2.794	13.205	1.00	26.40	C
ATOM	1778	CD	GLU	A	227	-28.944	-3.104	14.146	1.00	29.49	C
ATOM	1779	OE1	GLU	A	227	-28.945	-4.106	14.913	1.00	29.48	O
ATOM	1780	OE2	GLU	A	227	-29.925	-2.315	14.120	1.00	26.69	O1-
ATOM	1781	N	PHE	A	228	-24.524	-5.295	15.321	1.00	19.59	N
ATOM	1782	CA	PHE	A	228	-23.714	-6.468	15.669	1.00	21.16	C
ATOM	1783	C	PHE	A	228	-23.418	-7.380	14.467	1.00	22.82	C
ATOM	1784	O	PHE	A	228	-22.343	-8.002	14.393	1.00	22.75	O
ATOM	1785	CB	PHE	A	228	-24.392	-7.285	16.798	1.00	19.31	C
ATOM	1786	CG	PHE	A	228	-23.470	-8.270	17.481	1.00	15.55	C
ATOM	1787	CD1	PHE	A	228	-22.401	-7.826	18.231	1.00	11.82	C
ATOM	1788	CD2	PHE	A	228	-23.598	-9.636	17.254	1.00	13.74	C
ATOM	1789	CE1	PHE	A	228	-21.482	-8.709	18.727	1.00	11.35	C
ATOM	1790	CE2	PHE	A	228	-22.675	-10.526	17.751	1.00	10.57	C
ATOM	1791	CZ	PHE	A	228	-21.620	-10.067	18.485	1.00	10.53	C
ATOM	1792	N	ALA	A	229	-24.362	-7.472	13.532	1.00	22.31	N
ATOM	1793	CA	ALA	A	229	-24.033	-7.999	12.215	1.00	21.94	C
ATOM	1794	C	ALA	A	229	-22.699	-7.383	11.737	1.00	22.28	C
ATOM	1795	O	ALA	A	229	-21.645	-7.988	11.955	1.00	22.58	O
ATOM	1796	CB	ALA	A	229	-25.157	-7.698	11.238	1.00	18.80	C
ATOM	1797	N	GLU	A	230	-22.718	-6.071	11.494	1.00	23.17	N
ATOM	1798	CA	GLU	A	230	-21.634	-5.373	10.779	1.00	22.57	C
ATOM	1799	C	GLU	A	230	-20.289	-5.093	11.487	1.00	21.60	C
ATOM	1800	O	GLU	A	230	-19.291	-4.855	10.813	1.00	20.95	O
ATOM	1801	CB	GLU	A	230	-22.147	-4.061	10.165	1.00	19.48	C
ATOM	1802	CG	GLU	A	230	-21.896	-3.975	8.671	1.00	17.15	C
ATOM	1803	CD	GLU	A	230	-22.865	-4.813	7.850	1.00	17.55	C
ATOM	1804	OE1	GLU	A	230	-23.995	-4.329	7.598	1.00	17.87	O
ATOM	1805	OE2	GLU	A	230	-22.488	-5.926	7.409	1.00	15.08	O1-
ATOM	1806	N	VAL	A	231	-20.231	-5.073	12.814	1.00	20.69	N
ATOM	1807	CA	VAL	A	231	-18.917	-5.007	13.435	1.00	16.38	C
ATOM	1808	C	VAL	A	231	-18.287	-6.364	13.136	1.00	20.32	C
ATOM	1809	O	VAL	A	231	-17.101	-6.442	12.828	1.00	21.55	O
ATOM	1810	CB	VAL	A	231	-18.978	-4.758	14.964	1.00	12.08	C
ATOM	1811	CG1	VAL	A	231	-17.812	-5.401	15.645	1.00	11.97	C
ATOM	1812	CG2	VAL	A	231	-18.937	-3.285	15.262	1.00	11.69	C
ATOM	1813	N	SER	A	232	-19.108	-7.408	13.049	1.00	22.30	N
ATOM	1814	CA	SER	A	232	-18.566	-8.749	12.844	1.00	25.21	C
ATOM	1815	C	SER	A	232	-18.383	-9.125	11.373	1.00	26.83	C
ATOM	1816	O	SER	A	232	-18.337	-10.306	11.009	1.00	28.08	O
ATOM	1817	CB	SER	A	232	-19.408	-9.788	13.586	1.00	25.39	C
ATOM	1818	OG	SER	A	232	-18.793	-10.070	14.841	1.00	28.11	O
ATOM	1819	N	LYS	A	233	-18.260	-8.096	10.536	1.00	26.97	N
ATOM	1820	CA	LYS	A	233	-17.661	-8.228	9.225	1.00	23.19	C
ATOM	1821	C	LYS	A	233	-16.769	-7.018	9.066	1.00	20.48	C
ATOM	1822	O	LYS	A	233	-16.735	-6.421	8.012	1.00	21.98	O
ATOM	1823	CB	LYS	A	233	-18.733	-8.254	8.133	1.00	25.56	C
ATOM	1824	CG	LYS	A	233	-18.198	-8.687	6.754	1.00	29.24	C
ATOM	1825	CD	LYS	A	233	-19.299	-9.170	5.786	1.00	28.84	C
ATOM	1826	CE	LYS	A	233	-18.745	-10.207	4.785	1.00	29.50	C
ATOM	1827	NZ	LYS	A	233	-19.637	-10.474	3.618	1.00	28.23	N1+
ATOM	1828	N	LEU	A	234	-16.191	-6.564	10.174	1.00	19.32	N
ATOM	1829	CA	LEU	A	234	-15.119	-5.570	10.155	1.00	19.26	C
ATOM	1830	C	LEU	A	234	-13.971	-6.022	11.075	1.00	20.45	C
ATOM	1831	O	LEU	A	234	-12.798	-5.927	10.710	1.00	20.91	O
ATOM	1832	CB	LEU	A	234	-15.623	-4.191	10.598	1.00	18.55	C
ATOM	1833	CG	LEU	A	234	-16.945	-3.619	10.054	1.00	20.65	C
ATOM	1834	CD1	LEU	A	234	-17.229	-2.274	10.718	1.00	20.73	C
ATOM	1835	CD2	LEU	A	234	-16.900	-3.452	8.536	1.00	18.64	C
ATOM	1836	N	VAL	A	235	-14.308	-6.626	12.214	1.00	19.59	N
ATOM	1837	CA	VAL	A	235	-13.301	-7.227	13.089	1.00	16.45	C
ATOM	1838	C	VAL	A	235	-12.927	-8.677	12.704	1.00	16.23	C
ATOM	1839	O	VAL	A	235	-11.848	-9.153	13.054	1.00	17.61	O
ATOM	1840	CB	VAL	A	235	-13.756	-7.164	14.556	1.00	14.01	C
ATOM	1841	CG1	VAL	A	235	-15.208	-7.513	14.660	1.00	14.30	C
ATOM	1842	CG2	VAL	A	235	-12.943	-8.102	15.421	1.00	14.29	C
ATOM	1843	N	THR	A	236	-13.808	-9.361	11.976	1.00	13.39	N

ATOM	1844	CA	THR	A	236	-13.512	-10.659	11.367	1.00	10.14	C
ATOM	1845	C	THR	A	236	-12.673	-10.444	10.102	1.00	12.00	C
ATOM	1846	O	THR	A	236	-11.962	-11.342	9.665	1.00	10.48	O
ATOM	1847	CB	THR	A	236	-14.803	-11.359	10.949	1.00	9.09	C
ATOM	1848	CG2	THR	A	236	-14.960	-12.671	11.657	1.00	10.99	C
ATOM	1849	OG1	THR	A	236	-15.914	-10.525	11.281	1.00	5.10	O
ATOM	1850	N	ASP	A	237	-12.708	-9.207	9.592	1.00	13.66	N
ATOM	1851	CA	ASP	A	237	-12.113	-8.807	8.320	1.00	12.69	C
ATOM	1852	C	ASP	A	237	-10.804	-8.086	8.567	1.00	16.23	C
ATOM	1853	O	ASP	A	237	-10.160	-7.609	7.622	1.00	17.55	O
ATOM	1854	CB	ASP	A	237	-13.052	-7.857	7.581	1.00	9.81	C
ATOM	1855	CG	ASP	A	237	-14.195	-8.580	6.879	1.00	15.79	C
ATOM	1856	OD1	ASP	A	237	-14.151	-9.824	6.788	1.00	20.11	O
ATOM	1857	OD2	ASP	A	237	-15.114	-7.914	6.350	1.00	14.61	O1-
ATOM	1858	N	LEU	A	238	-10.504	-7.875	9.849	1.00	17.84	N
ATOM	1859	CA	LEU	A	238	-9.376	-7.046	10.284	1.00	17.70	C
ATOM	1860	C	LEU	A	238	-8.366	-7.946	10.952	1.00	17.11	C
ATOM	1861	O	LEU	A	238	-7.171	-7.656	10.954	1.00	18.24	O
ATOM	1862	CB	LEU	A	238	-9.830	-6.016	11.310	1.00	18.48	C
ATOM	1863	CG	LEU	A	238	-9.252	-4.614	11.261	1.00	15.02	C
ATOM	1864	CD1	LEU	A	238	-10.258	-3.748	10.568	1.00	13.60	C
ATOM	1865	CD2	LEU	A	238	-8.989	-4.102	12.650	1.00	11.12	C
ATOM	1866	N	THR	A	239	-8.868	-8.928	11.684	1.00	15.12	N
ATOM	1867	CA	THR	A	239	-8.031	-10.025	12.104	1.00	15.74	C
ATOM	1868	C	THR	A	239	-7.229	-10.453	10.851	1.00	16.69	C
ATOM	1869	O	THR	A	239	-6.120	-9.942	10.661	1.00	18.36	O
ATOM	1870	CB	THR	A	239	-8.899	-11.180	12.745	1.00	15.86	C
ATOM	1871	CG2	THR	A	239	-8.061	-12.423	13.008	1.00	12.44	C
ATOM	1872	OG1	THR	A	239	-9.476	-10.724	13.988	1.00	7.33	O
ATOM	1873	N	LYS	A	240	-7.899	-11.064	9.873	1.00	15.75	N
ATOM	1874	CA	LYS	A	240	-7.262	-11.521	8.617	1.00	14.50	C
ATOM	1875	C	LYS	A	240	-6.100	-10.649	8.114	1.00	12.60	C
ATOM	1876	O	LYS	A	240	-5.042	-11.164	7.764	1.00	12.12	O
ATOM	1877	CB	LYS	A	240	-8.314	-11.608	7.513	1.00	13.34	C
ATOM	1878	CG	LYS	A	240	-8.390	-12.935	6.845	1.00	16.59	C
ATOM	1879	CD	LYS	A	240	-9.351	-12.874	5.664	1.00	23.14	C
ATOM	1880	CE	LYS	A	240	-8.943	-13.846	4.544	1.00	26.65	C
ATOM	1881	NZ	LYS	A	240	-7.629	-13.487	3.874	1.00	31.21	N1+
ATOM	1882	N	VAL	A	241	-6.361	-9.349	7.966	1.00	12.72	N
ATOM	1883	CA	VAL	A	241	-5.359	-8.348	7.614	1.00	9.66	C
ATOM	1884	C	VAL	A	241	-4.266	-8.481	8.636	1.00	11.94	C
ATOM	1885	O	VAL	A	241	-3.289	-9.155	8.386	1.00	14.55	O
ATOM	1886	CB	VAL	A	241	-5.909	-6.900	7.705	1.00	8.96	C
ATOM	1887	CG1	VAL	A	241	-4.808	-5.903	7.469	1.00	6.95	C
ATOM	1888	CG2	VAL	A	241	-7.030	-6.681	6.719	1.00	7.52	C
ATOM	1889	N	HIS	A	242	-4.541	-8.050	9.858	1.00	11.85	N
ATOM	1890	CA	HIS	A	242	-3.490	-7.939	10.863	1.00	17.50	C
ATOM	1891	C	HIS	A	242	-2.715	-9.236	11.110	1.00	19.56	C
ATOM	1892	O	HIS	A	242	-1.490	-9.212	11.321	1.00	19.10	O
ATOM	1893	CB	HIS	A	242	-4.071	-7.433	12.179	1.00	19.40	C
ATOM	1894	CG	HIS	A	242	-4.033	-5.949	12.311	1.00	17.51	C
ATOM	1895	CD2	HIS	A	242	-3.023	-5.113	12.638	1.00	17.18	C
ATOM	1896	ND1	HIS	A	242	-5.063	-5.146	11.870	1.00	14.59	N
ATOM	1897	CE1	HIS	A	242	-4.680	-3.885	11.896	1.00	14.07	C
ATOM	1898	NE2	HIS	A	242	-3.445	-3.842	12.360	1.00	20.83	N
ATOM	1899	N	THR	A	243	-3.419	-10.364	11.043	1.00	20.29	N
ATOM	1900	CA	THR	A	243	-2.791	-11.653	11.247	1.00	21.66	C
ATOM	1901	C	THR	A	243	-1.638	-11.802	10.259	1.00	22.36	C
ATOM	1902	O	THR	A	243	-0.494	-11.979	10.667	1.00	20.98	O
ATOM	1903	CB	THR	A	243	-3.803	-12.808	11.058	1.00	22.04	C
ATOM	1904	CG2	THR	A	243	-4.560	-13.061	12.338	1.00	19.78	C
ATOM	1905	OG1	THR	A	243	-4.743	-12.465	10.030	1.00	22.04	O
ATOM	1906	N	GLU	A	244	-1.923	-11.503	8.994	1.00	24.92	N
ATOM	1907	CA	GLU	A	244	-0.963	-11.634	7.884	1.00	24.81	C
ATOM	1908	C	GLU	A	244	0.269	-10.751	8.073	1.00	24.55	C
ATOM	1909	O	GLU	A	244	1.365	-11.289	8.316	1.00	25.76	O
ATOM	1910	CB	GLU	A	244	-1.654	-11.320	6.540	1.00	21.04	C
ATOM	1911	CG	GLU	A	244	-2.417	-12.526	5.981	1.00	20.42	C
ATOM	1912	CD	GLU	A	244	-3.749	-12.186	5.369	1.00	16.71	C
ATOM	1913	OE1	GLU	A	244	-3.861	-11.097	4.794	1.00	19.88	O
ATOM	1914	OE2	GLU	A	244	-4.670	-13.029	5.410	1.00	15.80	O1-

ATOM	1915	N	CYS	A	245	0.061	-9.426	8.150	1.00	23.02	N
ATOM	1916	CA	CYS	A	245	1.148	-8.466	8.429	1.00	21.48	C
ATOM	1917	C	CYS	A	245	1.936	-8.829	9.702	1.00	21.49	C
ATOM	1918	O	CYS	A	245	2.965	-8.215	10.000	1.00	20.53	O
ATOM	1919	CB	CYS	A	245	0.604	-7.039	8.577	1.00	20.81	C
ATOM	1920	SG	CYS	A	245	0.340	-6.068	7.058	1.00	19.72	S
ATOM	1921	N	CYS	A	246	1.434	-9.803	10.458	1.00	20.66	N
ATOM	1922	CA	CYS	A	246	2.133	-10.288	11.635	1.00	22.93	C
ATOM	1923	C	CYS	A	246	2.810	-11.634	11.342	1.00	23.54	C
ATOM	1924	O	CYS	A	246	4.001	-11.829	11.613	1.00	23.49	O
ATOM	1925	CB	CYS	A	246	1.150	-10.431	12.828	1.00	23.38	C
ATOM	1926	SG	CYS	A	246	0.340	-8.909	13.476	1.00	21.26	S
ATOM	1927	N	HIS	A	247	2.054	-12.531	10.717	1.00	26.98	N
ATOM	1928	CA	HIS	A	247	2.461	-13.924	10.463	1.00	28.41	C
ATOM	1929	C	HIS	A	247	3.551	-14.018	9.371	1.00	27.16	C
ATOM	1930	O	HIS	A	247	3.999	-15.107	8.996	1.00	24.90	O
ATOM	1931	CB	HIS	A	247	1.221	-14.734	10.034	1.00	30.64	C
ATOM	1932	CG	HIS	A	247	0.887	-15.871	10.953	1.00	33.09	C
ATOM	1933	CD2	HIS	A	247	1.675	-16.666	11.718	1.00	31.19	C
ATOM	1934	ND1	HIS	A	247	-0.397	-16.372	11.077	1.00	32.64	N
ATOM	1935	CE1	HIS	A	247	-0.383	-17.423	11.881	1.00	32.18	C
ATOM	1936	NE2	HIS	A	247	0.859	-17.616	12.285	1.00	34.75	N
ATOM	1937	N	GLY	A	248	3.882	-12.875	8.788	1.00	26.19	N
ATOM	1938	CA	GLY	A	248	5.010	-12.822	7.893	1.00	24.62	C
ATOM	1939	C	GLY	A	248	4.560	-12.235	6.584	1.00	24.54	C
ATOM	1940	O	GLY	A	248	5.066	-11.200	6.176	1.00	25.55	O
ATOM	1941	N	ASP	A	249	3.553	-12.847	5.966	1.00	23.88	N
ATOM	1942	CA	ASP	A	249	3.154	-12.470	4.619	1.00	20.47	C
ATOM	1943	C	ASP	A	249	2.317	-11.215	4.621	1.00	20.76	C
ATOM	1944	O	ASP	A	249	1.132	-11.257	4.957	1.00	21.26	O
ATOM	1945	CB	ASP	A	249	2.408	-13.612	3.903	1.00	20.40	C
ATOM	1946	CG	ASP	A	249	1.351	-14.282	4.764	1.00	18.24	C
ATOM	1947	OD1	ASP	A	249	1.703	-15.199	5.537	1.00	19.57	O
ATOM	1948	OD2	ASP	A	249	0.152	-14.040	4.523	1.00	17.76	O1-
ATOM	1949	N	LEU	A	250	3.003	-10.087	4.423	1.00	20.08	N
ATOM	1950	CA	LEU	A	250	2.373	-8.789	4.180	1.00	20.75	C
ATOM	1951	C	LEU	A	250	1.422	-8.841	2.956	1.00	23.97	C
ATOM	1952	O	LEU	A	250	0.246	-9.180	3.084	1.00	24.23	O
ATOM	1953	CB	LEU	A	250	3.427	-7.715	3.892	1.00	18.42	C
ATOM	1954	CG	LEU	A	250	4.765	-7.394	4.537	1.00	17.52	C
ATOM	1955	CD1	LEU	A	250	5.333	-8.472	5.381	1.00	20.69	C
ATOM	1956	CD2	LEU	A	250	5.689	-7.106	3.393	1.00	20.41	C
ATOM	1957	N	LEU	A	251	1.964	-8.473	1.788	1.00	24.70	N
ATOM	1958	CA	LEU	A	251	1.273	-8.329	0.499	1.00	21.99	C
ATOM	1959	C	LEU	A	251	-0.227	-8.608	0.403	1.00	22.01	C
ATOM	1960	O	LEU	A	251	-0.983	-7.834	-0.179	1.00	19.96	O
ATOM	1961	CB	LEU	A	251	2.016	-9.161	-0.540	1.00	22.73	C
ATOM	1962	CG	LEU	A	251	3.503	-8.825	-0.674	1.00	21.62	C
ATOM	1963	CD1	LEU	A	251	4.302	-9.609	0.354	1.00	22.59	C
ATOM	1964	CD2	LEU	A	251	3.972	-9.145	-2.085	1.00	20.79	C
ATOM	1965	N	GLU	A	252	-0.633	-9.779	0.856	1.00	23.52	N
ATOM	1966	CA	GLU	A	252	-2.053	-10.078	0.994	1.00	24.95	C
ATOM	1967	C	GLU	A	252	-2.699	-8.956	1.840	1.00	25.20	C
ATOM	1968	O	GLU	A	252	-3.583	-8.247	1.364	1.00	26.85	O
ATOM	1969	CB	GLU	A	252	-2.225	-11.463	1.648	1.00	23.50	C
ATOM	1970	CG	GLU	A	252	-1.702	-12.659	0.793	1.00	23.59	C
ATOM	1971	CD	GLU	A	252	-0.211	-12.968	0.966	1.00	22.46	C
ATOM	1972	OE1	GLU	A	252	0.548	-12.064	1.372	1.00	21.42	O
ATOM	1973	OE2	GLU	A	252	0.212	-14.112	0.667	1.00	20.54	O1-
ATOM	1974	N	CYS	A	253	-2.113	-8.683	3.002	1.00	23.06	N
ATOM	1975	CA	CYS	A	253	-2.418	-7.509	3.825	1.00	21.89	C
ATOM	1976	C	CYS	A	253	-2.879	-6.277	3.017	1.00	22.51	C
ATOM	1977	O	CYS	A	253	-4.077	-5.982	2.928	1.00	18.60	O
ATOM	1978	CB	CYS	A	253	-1.171	-7.153	4.662	1.00	20.32	C
ATOM	1979	SG	CYS	A	253	-1.466	-6.653	6.385	1.00	15.33	S
ATOM	1980	N	ALA	A	254	-1.921	-5.638	2.345	1.00	22.49	N
ATOM	1981	CA	ALA	A	254	-2.105	-4.310	1.755	1.00	22.36	C
ATOM	1982	C	ALA	A	254	-3.233	-4.310	0.727	1.00	22.41	C
ATOM	1983	O	ALA	A	254	-3.812	-3.264	0.430	1.00	20.73	O
ATOM	1984	CB	ALA	A	254	-0.785	-3.836	1.104	1.00	20.82	C
ATOM	1985	N	ASP	A	255	-3.433	-5.475	0.108	1.00	23.61	N

```
ATOM   1986  CA  ASP A 255      -4.448  -5.684  -0.918  1.00 23.62           C
ATOM   1987  C   ASP A 255      -5.725  -5.744  -0.112  1.00 24.73           C
ATOM   1988  O   ASP A 255      -6.638  -4.941  -0.353  1.00 25.80           O
ATOM   1989  CB  ASP A 255      -4.205  -7.026  -1.648  1.00 24.09           C
ATOM   1990  CG  ASP A 255      -5.128  -7.234  -2.858  1.00 26.69           C
ATOM   1991  OD1 ASP A 255      -5.894  -6.309  -3.200  1.00 28.50           O
ATOM   1992  OD2 ASP A 255      -5.063  -8.310  -3.507  1.00 25.31           O1-
ATOM   1993  N   ASP A 256      -5.645  -6.478   1.009  1.00 24.05           N
ATOM   1994  CA  ASP A 256      -6.778  -6.758   1.893  1.00 21.53           C
ATOM   1995  C   ASP A 256      -7.408  -5.507   2.499  1.00 22.11           C
ATOM   1996  O   ASP A 256      -8.603  -5.319   2.331  1.00 21.08           O
ATOM   1997  CB  ASP A 256      -6.375  -7.720   3.009  1.00 19.32           C
ATOM   1998  CG  ASP A 256      -6.474  -9.182   2.594  1.00 21.17           C
ATOM   1999  OD1 ASP A 256      -7.390  -9.548   1.814  1.00 17.48           O
ATOM   2000  OD2 ASP A 256      -5.650  -9.984   3.094  1.00 19.56           O1-
ATOM   2001  N   ARG A 257      -6.624  -4.584   3.069  1.00 22.19           N
ATOM   2002  CA  ARG A 257      -7.214  -3.327   3.566  1.00 22.80           C
ATOM   2003  C   ARG A 257      -7.861  -2.446   2.497  1.00 23.33           C
ATOM   2004  O   ARG A 257      -8.179  -1.277   2.756  1.00 24.22           O
ATOM   2005  CB  ARG A 257      -6.212  -2.474   4.344  1.00 23.48           C
ATOM   2006  CG  ARG A 257      -6.912  -1.398   5.220  1.00 26.53           C
ATOM   2007  CD  ARG A 257      -6.367  -1.389   6.660  1.00 32.02           C
ATOM   2008  NE  ARG A 257      -7.343  -1.126   7.730  1.00 29.83           N
ATOM   2009  CZ  ARG A 257      -6.970  -0.900   8.990  1.00 29.33           C
ATOM   2010  NH1 ARG A 257      -5.677  -0.815   9.258  1.00 31.19           N1+
ATOM   2011  NH2 ARG A 257      -7.857  -0.700   9.970  1.00 27.88           N
ATOM   2012  N   ALA A 258      -8.016  -2.989   1.288  1.00 23.60           N
ATOM   2013  CA  ALA A 258      -8.843  -2.381   0.247  1.00 19.06           C
ATOM   2014  C   ALA A 258      -9.853  -3.397  -0.273  1.00 17.21           C
ATOM   2015  O   ALA A 258     -11.020  -3.095  -0.345  1.00 16.88           O
ATOM   2016  CB  ALA A 258      -7.989  -1.870  -0.849  1.00 17.23           C
ATOM   2017  N   ASP A 259      -9.464  -4.662  -0.292  1.00 18.75           N
ATOM   2018  CA  ASP A 259     -10.430  -5.768  -0.205  1.00 21.08           C
ATOM   2019  C   ASP A 259     -11.253  -5.577   1.090  1.00 19.96           C
ATOM   2020  O   ASP A 259     -12.159  -6.365   1.380  1.00 20.42           O
ATOM   2021  CB  ASP A 259      -9.690  -7.150  -0.180  1.00 24.22           C
ATOM   2022  CG  ASP A 259     -10.169  -8.150  -1.288  1.00 21.26           C
ATOM   2023  OD1 ASP A 259     -11.075  -7.821  -2.076  1.00 21.43           O
ATOM   2024  OD2 ASP A 259      -9.634  -9.279  -1.364  1.00 17.32           O1-
ATOM   2025  N   LEU A 260     -10.848  -4.617   1.924  1.00 18.38           N
ATOM   2026  CA  LEU A 260     -11.732  -4.087   2.948  1.00 19.05           C
ATOM   2027  C   LEU A 260     -12.198  -2.692   2.561  1.00 20.78           C
ATOM   2028  O   LEU A 260     -13.257  -2.581   1.950  1.00 22.27           O
ATOM   2029  CB  LEU A 260     -11.087  -4.076   4.334  1.00 18.98           C
ATOM   2030  CG  LEU A 260     -11.986  -4.337   5.564  1.00 15.64           C
ATOM   2031  CD1 LEU A 260     -11.913  -3.163   6.525  1.00 14.71           C
ATOM   2032  CD2 LEU A 260     -13.418  -4.561   5.153  1.00 12.68           C
ATOM   2033  N   ALA A 261     -11.398  -1.651   2.801  1.00 19.14           N
ATOM   2034  CA  ALA A 261     -11.884  -0.274   2.617  1.00 20.29           C
ATOM   2035  C   ALA A 261     -12.836  -0.069   1.445  1.00 20.76           C
ATOM   2036  O   ALA A 261     -13.748   0.727   1.551  1.00 23.76           O
ATOM   2037  CB  ALA A 261     -10.738   0.703   2.506  1.00 20.41           C
ATOM   2038  N   LYS A 262     -12.715  -0.870   0.390  1.00 21.67           N
ATOM   2039  CA  LYS A 262     -13.724  -0.914  -0.678  1.00 22.13           C
ATOM   2040  C   LYS A 262     -14.858  -1.897  -0.338  1.00 22.91           C
ATOM   2041  O   LYS A 262     -15.231  -2.735  -1.178  1.00 24.14           O
ATOM   2042  CB  LYS A 262     -13.079  -1.319  -2.018  1.00 21.14           C
ATOM   2043  CG  LYS A 262     -12.745  -0.182  -2.958  1.00 20.45           C
ATOM   2044  CD  LYS A 262     -13.871   0.032  -3.965  1.00 22.63           C
ATOM   2045  CE  LYS A 262     -13.416   0.915  -5.138  1.00 22.50           C
ATOM   2046  NZ  LYS A 262     -13.721   0.318  -6.475  1.00 18.96           N1+
ATOM   2047  N   TYR A 263     -15.279  -1.881   0.933  1.00 20.93           N
ATOM   2048  CA  TYR A 263     -16.547  -2.447   1.416  1.00 15.95           C
ATOM   2049  C   TYR A 263     -17.010  -1.449   2.439  1.00 14.10           C
ATOM   2050  O   TYR A 263     -17.949  -0.720   2.178  1.00 16.17           O
ATOM   2051  CB  TYR A 263     -16.349  -3.803   2.096  1.00 16.93           C
ATOM   2052  CG  TYR A 263     -17.327  -4.107   3.212  1.00 14.41           C
ATOM   2053  CD1 TYR A 263     -18.595  -4.556   2.931  1.00 17.78           C
ATOM   2054  CD2 TYR A 263     -17.055  -3.743   4.505  1.00 15.15           C
ATOM   2055  CE1 TYR A 263     -19.577  -4.581   3.889  1.00 17.72           C
ATOM   2056  CE2 TYR A 263     -18.025  -3.763   5.472  1.00 16.35           C
```

```
ATOM   2057  CZ  TYR A 263     -19.294  -4.157   5.153  1.00 17.14           C
ATOM   2058  OH  TYR A 263     -20.326  -3.914   6.025  1.00 18.82           O
ATOM   2059  N   ILE A 264     -16.205  -1.275   3.488  1.00 13.60           N
ATOM   2060  CA  ILE A 264     -16.396  -0.224   4.502  1.00 13.72           C
ATOM   2061  C   ILE A 264     -16.916   0.992   3.801  1.00 14.67           C
ATOM   2062  O   ILE A 264     -18.082   1.333   3.943  1.00 16.24           O
ATOM   2063  CB  ILE A 264     -15.058   0.192   5.230  1.00 11.33           C
ATOM   2064  CG1 ILE A 264     -14.910  -0.527   6.568  1.00  9.86           C
ATOM   2065  CG2 ILE A 264     -15.057   1.676   5.551  1.00 12.03           C
ATOM   2066  CD1 ILE A 264     -13.924   0.134   7.518  1.00  8.24           C
ATOM   2067  N   CYS A 265     -16.146   1.431   2.817  1.00 18.20           N
ATOM   2068  CA  CYS A 265     -16.409   2.706   2.184  1.00 21.00           C
ATOM   2069  C   CYS A 265     -17.183   2.683   0.863  1.00 20.34           C
ATOM   2070  O   CYS A 265     -17.164   3.648   0.104  1.00 18.15           O
ATOM   2071  CB  CYS A 265     -15.131   3.508   2.016  1.00 18.44           C
ATOM   2072  SG  CYS A 265     -15.717   5.147   1.584  1.00 24.42           S
ATOM   2073  N   GLU A 266     -17.927   1.612   0.630  1.00 22.55           N
ATOM   2074  CA  GLU A 266     -19.044   1.668  -0.305  1.00 25.71           C
ATOM   2075  C   GLU A 266     -20.359   1.649   0.507  1.00 25.99           C
ATOM   2076  O   GLU A 266     -21.401   2.132   0.031  1.00 24.57           O
ATOM   2077  CB  GLU A 266     -18.982   0.486  -1.306  1.00 30.26           C
ATOM   2078  CG  GLU A 266     -18.126   0.746  -2.589  1.00 33.40           C
ATOM   2079  CD  GLU A 266     -18.430  -0.224  -3.755  1.00 33.38           C
ATOM   2080  OE1 GLU A 266     -19.340   0.064  -4.569  1.00 34.65           O
ATOM   2081  OE2 GLU A 266     -17.702  -1.226  -3.918  1.00 31.65           O1-
ATOM   2082  N   ASN A 267     -20.231   1.304   1.792  1.00 24.11           N
ATOM   2083  CA  ASN A 267     -21.367   1.133   2.693  1.00 24.75           C
ATOM   2084  C   ASN A 267     -21.562   2.304   3.675  1.00 25.96           C
ATOM   2085  O   ASN A 267     -22.551   2.367   4.425  1.00 24.17           O
ATOM   2086  CB  ASN A 267     -21.201  -0.171   3.465  1.00 25.26           C
ATOM   2087  CG  ASN A 267     -21.552  -1.388   2.630  1.00 26.30           C
ATOM   2088  ND2 ASN A 267     -21.767  -2.527   3.293  1.00 23.38           N
ATOM   2089  OD1 ASN A 267     -21.602  -1.314   1.394  1.00 28.38           O
ATOM   2090  N   GLN A 268     -20.543   3.150   3.756  1.00 27.51           N
ATOM   2091  CA  GLN A 268     -20.619   4.455   4.422  1.00 26.43           C
ATOM   2092  C   GLN A 268     -21.731   4.680   5.469  1.00 25.52           C
ATOM   2093  O   GLN A 268     -21.432   5.026   6.604  1.00 23.74           O
ATOM   2094  CB  GLN A 268     -20.693   5.552   3.354  1.00 24.02           C
ATOM   2095  CG  GLN A 268     -19.418   6.340   3.149  1.00 20.44           C
ATOM   2096  CD  GLN A 268     -19.711   7.673   2.493  1.00 23.29           C
ATOM   2097  NE2 GLN A 268     -18.762   8.607   2.568  1.00 22.63           N
ATOM   2098  OE1 GLN A 268     -20.807   7.873   1.949  1.00 24.24           O
ATOM   2099  N   ASP A 269     -22.993   4.648   5.054  1.00 25.35           N
ATOM   2100  CA  ASP A 269     -24.064   5.203   5.882  1.00 26.24           C
ATOM   2101  C   ASP A 269     -24.667   4.170   6.833  1.00 28.27           C
ATOM   2102  O   ASP A 269     -25.243   4.533   7.873  1.00 29.26           O
ATOM   2103  CB  ASP A 269     -25.180   5.803   5.009  1.00 27.91           C
ATOM   2104  CG  ASP A 269     -25.889   4.753   4.153  1.00 29.64           C
ATOM   2105  OD1 ASP A 269     -25.178   3.900   3.565  1.00 30.12           O
ATOM   2106  OD2 ASP A 269     -27.151   4.754   4.103  1.00 29.00           O1-
ATOM   2107  N   SER A 270     -24.536   2.886   6.501  1.00 27.48           N
ATOM   2108  CA  SER A 270     -24.993   1.847   7.413  1.00 26.75           C
ATOM   2109  C   SER A 270     -23.949   1.519   8.504  1.00 26.55           C
ATOM   2110  O   SER A 270     -24.139   0.582   9.286  1.00 26.34           O
ATOM   2111  CB  SER A 270     -25.359   0.589   6.623  1.00 28.19           C
ATOM   2112  OG  SER A 270     -24.224   0.037   5.957  1.00 30.87           O
ATOM   2113  N   ILE A 271     -22.895   2.329   8.614  1.00 25.27           N
ATOM   2114  CA  ILE A 271     -21.741   1.967   9.420  1.00 24.11           C
ATOM   2115  C   ILE A 271     -21.253   3.034  10.393  1.00 25.93           C
ATOM   2116  O   ILE A 271     -21.228   2.784  11.593  1.00 27.16           O
ATOM   2117  CB  ILE A 271     -20.578   1.539   8.535  1.00 25.05           C
ATOM   2118  CG1 ILE A 271     -20.832   0.113   8.013  1.00 27.97           C
ATOM   2119  CG2 ILE A 271     -19.278   1.600   9.305  1.00 24.66           C
ATOM   2120  CD1 ILE A 271     -19.661  -0.523   7.232  1.00 23.58           C
ATOM   2121  N   SER A 272     -20.749   4.167   9.908  1.00 27.76           N
ATOM   2122  CA  SER A 272     -20.329   5.238  10.836  1.00 28.74           C
ATOM   2123  C   SER A 272     -20.165   6.631  10.227  1.00 27.74           C
ATOM   2124  O   SER A 272     -19.123   7.259  10.418  1.00 26.72           O
ATOM   2125  CB  SER A 272     -19.018   4.837  11.530  1.00 30.16           C
ATOM   2126  OG  SER A 272     -18.380   5.901  12.239  1.00 27.56           O
ATOM   2127  N   SER A 273     -21.257   7.182   9.701  1.00 27.40           N
```

ATOM	2128	CA	SER	A	273	-21.236	8.458	8.970	1.00	26.73	C
ATOM	2129	C	SER	A	273	-19.842	9.066	8.643	1.00	25.15	C
ATOM	2130	O	SER	A	273	-19.475	9.204	7.468	1.00	20.82	O
ATOM	2131	CB	SER	A	273	-22.110	9.495	9.714	1.00	25.04	C
ATOM	2132	OG	SER	A	273	-21.417	10.725	9.872	1.00	24.31	O
ATOM	2133	N	LYS	A	274	-19.104	9.433	9.697	1.00	24.05	N
ATOM	2134	CA	LYS	A	274	-17.875	10.230	9.625	1.00	24.74	C
ATOM	2135	C	LYS	A	274	-16.764	9.750	8.705	1.00	22.58	C
ATOM	2136	O	LYS	A	274	-15.721	10.376	8.669	1.00	21.30	O
ATOM	2137	CB	LYS	A	274	-17.260	10.431	11.024	1.00	25.80	C
ATOM	2138	CG	LYS	A	274	-18.220	10.937	12.072	1.00	29.40	C
ATOM	2139	CD	LYS	A	274	-18.241	12.437	12.131	1.00	33.71	C
ATOM	2140	CE	LYS	A	274	-18.315	12.928	13.589	1.00	34.96	C
ATOM	2141	NZ	LYS	A	274	-17.356	14.082	13.815	1.00	34.17	N1+
ATOM	2142	N	LEU	A	275	-16.941	8.657	7.970	1.00	23.57	N
ATOM	2143	CA	LEU	A	275	-15.937	8.325	6.959	1.00	23.18	C
ATOM	2144	C	LEU	A	275	-16.330	8.834	5.585	1.00	22.54	C
ATOM	2145	O	LEU	A	275	-16.224	8.104	4.610	1.00	21.30	O
ATOM	2146	CB	LEU	A	275	-15.652	6.828	6.872	1.00	21.39	C
ATOM	2147	CG	LEU	A	275	-16.319	5.772	7.741	1.00	23.35	C
ATOM	2148	CD1	LEU	A	275	-17.580	5.221	7.072	1.00	23.95	C
ATOM	2149	CD2	LEU	A	275	-15.319	4.663	7.986	1.00	22.41	C
ATOM	2150	N	LYS	A	276	-16.828	10.073	5.514	1.00	25.95	N
ATOM	2151	CA	LYS	A	276	-16.886	10.830	4.247	1.00	26.73	C
ATOM	2152	C	LYS	A	276	-15.478	11.187	3.799	1.00	25.18	C
ATOM	2153	O	LYS	A	276	-14.865	10.434	3.052	1.00	25.22	O
ATOM	2154	CB	LYS	A	276	-17.710	12.129	4.402	1.00	29.83	C
ATOM	2155	CG	LYS	A	276	-17.702	12.805	5.801	1.00	30.45	C
ATOM	2156	CD	LYS	A	276	-16.755	14.005	5.874	1.00	30.09	C
ATOM	2157	CE	LYS	A	276	-16.974	14.966	4.712	1.00	31.30	C
ATOM	2158	NZ	LYS	A	276	-18.160	15.877	4.882	1.00	29.48	N1+
ATOM	2159	N	GLU	A	277	-14.899	12.172	4.482	1.00	25.27	N
ATOM	2160	CA	GLU	A	277	-13.552	12.674	4.211	1.00	26.60	C
ATOM	2161	C	GLU	A	277	-12.640	11.674	3.545	1.00	25.74	C
ATOM	2162	O	GLU	A	277	-12.369	11.752	2.348	1.00	25.45	O
ATOM	2163	CB	GLU	A	277	-12.899	13.131	5.511	1.00	26.87	C
ATOM	2164	CG	GLU	A	277	-13.175	14.597	5.841	1.00	31.72	C
ATOM	2165	CD	GLU	A	277	-12.559	15.583	4.830	1.00	30.45	C
ATOM	2166	OE1	GLU	A	277	-13.198	15.870	3.787	1.00	27.15	O
ATOM	2167	OE2	GLU	A	277	-11.473	16.133	5.136	1.00	30.36	O1-
ATOM	2168	N	CYS	A	278	-12.396	10.601	4.270	1.00	26.80	N
ATOM	2169	CA	CYS	A	278	-11.438	9.582	3.859	1.00	27.12	C
ATOM	2170	C	CYS	A	278	-12.143	8.579	2.975	1.00	23.38	C
ATOM	2171	O	CYS	A	278	-12.167	7.391	3.233	1.00	24.65	O
ATOM	2172	CB	CYS	A	278	-10.871	8.920	5.104	1.00	31.72	C
ATOM	2173	SG	CYS	A	278	-11.643	9.660	6.586	1.00	33.14	S
ATOM	2174	N	CYS	A	279	-12.574	9.086	1.842	1.00	21.92	N
ATOM	2175	CA	CYS	A	279	-13.315	8.339	0.856	1.00	20.79	C
ATOM	2176	C	CYS	A	279	-13.460	9.359	-0.255	1.00	21.27	C
ATOM	2177	O	CYS	A	279	-13.962	9.073	-1.350	1.00	22.78	O
ATOM	2178	CB	CYS	A	279	-14.661	7.929	1.439	1.00	19.99	C
ATOM	2179	SG	CYS	A	279	-14.510	6.478	2.522	1.00	21.12	S
ATOM	2180	N	GLU	A	280	-13.143	10.594	0.107	1.00	18.93	N
ATOM	2181	CA	GLU	A	280	-12.521	11.517	-0.799	1.00	19.87	C
ATOM	2182	C	GLU	A	280	-11.046	11.586	-0.419	1.00	21.37	C
ATOM	2183	O	GLU	A	280	-10.570	12.645	-0.028	1.00	22.62	O
ATOM	2184	CB	GLU	A	280	-13.152	12.896	-0.661	1.00	19.56	C
ATOM	2185	CG	GLU	A	280	-14.562	12.867	-0.153	1.00	22.77	C
ATOM	2186	CD	GLU	A	280	-15.551	12.438	-1.207	1.00	24.09	C
ATOM	2187	OE1	GLU	A	280	-15.638	11.225	-1.511	1.00	22.38	O
ATOM	2188	OE2	GLU	A	280	-16.260	13.325	-1.723	1.00	26.29	O1-
ATOM	2189	N	LYS	A	281	-10.362	10.437	-0.413	1.00	21.75	N
ATOM	2190	CA	LYS	A	281	-8.896	10.399	-0.281	1.00	22.31	C
ATOM	2191	C	LYS	A	281	-8.246	9.170	-0.941	1.00	23.66	C
ATOM	2192	O	LYS	A	281	-8.367	8.041	-0.431	1.00	25.63	O
ATOM	2193	CB	LYS	A	281	-8.474	10.459	1.188	1.00	19.47	C
ATOM	2194	CG	LYS	A	281	-8.113	11.852	1.682	1.00	21.76	C
ATOM	2195	CD	LYS	A	281	-8.906	12.198	2.952	1.00	25.79	C
ATOM	2196	CE	LYS	A	281	-8.282	13.351	3.761	1.00	27.84	C
ATOM	2197	NZ	LYS	A	281	-7.158	12.950	4.670	1.00	23.24	N1+
ATOM	2198	N	PRO	A	282	-7.451	9.396	-2.015	1.00	20.78	N

ATOM	2199	CA	PRO	A	282	-6.604	8.401	-2.683	1.00	19.52	C
ATOM	2200	C	PRO	A	282	-6.214	7.216	-1.818	1.00	19.87	C
ATOM	2201	O	PRO	A	282	-5.362	7.365	-0.939	1.00	19.82	O
ATOM	2202	CB	PRO	A	282	-5.376	9.210	-3.100	1.00	16.20	C
ATOM	2203	CG	PRO	A	282	-5.586	10.592	-2.507	1.00	15.71	C
ATOM	2204	CD	PRO	A	282	-7.052	10.740	-2.438	1.00	17.15	C
ATOM	2205	N	LEU	A	283	-6.642	6.027	-2.254	1.00	20.57	N
ATOM	2206	CA	LEU	A	283	-6.610	4.787	-1.461	1.00	20.98	C
ATOM	2207	C	LEU	A	283	-5.739	4.913	-0.252	1.00	20.62	C
ATOM	2208	O	LEU	A	283	-6.176	5.479	0.723	1.00	18.64	O
ATOM	2209	CB	LEU	A	283	-6.147	3.585	-2.288	1.00	22.23	C
ATOM	2210	CG	LEU	A	283	-6.130	2.292	-1.452	1.00	23.90	C
ATOM	2211	CD1	LEU	A	283	-7.217	1.299	-1.964	1.00	22.21	C
ATOM	2212	CD2	LEU	A	283	-4.730	1.673	-1.500	1.00	20.45	C
ATOM	2213	N	LEU	A	284	-4.471	4.536	-0.395	1.00	22.99	N
ATOM	2214	CA	LEU	A	284	-3.396	4.918	0.519	1.00	26.73	C
ATOM	2215	C	LEU	A	284	-3.912	5.607	1.803	1.00	28.33	C
ATOM	2216	O	LEU	A	284	-4.326	4.906	2.739	1.00	28.88	O
ATOM	2217	CB	LEU	A	284	-2.385	5.825	-0.225	1.00	28.31	C
ATOM	2218	CG	LEU	A	284	-1.765	5.434	-1.595	1.00	26.94	C
ATOM	2219	CD1	LEU	A	284	-1.303	6.690	-2.364	1.00	20.85	C
ATOM	2220	CD2	LEU	A	284	-0.594	4.469	-1.384	1.00	26.30	C
ATOM	2221	N	GLU	A	285	-4.223	6.906	1.683	1.00	28.79	N
ATOM	2222	CA	GLU	A	285	-4.614	7.759	2.825	1.00	28.34	C
ATOM	2223	C	GLU	A	285	-6.001	7.475	3.396	1.00	26.33	C
ATOM	2224	O	GLU	A	285	-6.293	7.857	4.516	1.00	28.65	O
ATOM	2225	CB	GLU	A	285	-4.542	9.247	2.462	1.00	28.57	C
ATOM	2226	CG	GLU	A	285	-3.144	9.754	2.142	1.00	29.88	C
ATOM	2227	CD	GLU	A	285	-3.105	10.581	0.870	1.00	30.17	C
ATOM	2228	OE1	GLU	A	285	-3.483	11.778	0.920	1.00	29.25	O
ATOM	2229	OE2	GLU	A	285	-2.744	10.009	-0.185	1.00	28.46	O1-
ATOM	2230	N	LYS	A	286	-6.880	6.870	2.617	1.00	26.40	N
ATOM	2231	CA	LYS	A	286	-8.030	6.193	3.204	1.00	26.58	C
ATOM	2232	C	LYS	A	286	-7.525	5.430	4.440	1.00	24.48	C
ATOM	2233	O	LYS	A	286	-7.732	5.868	5.567	1.00	25.77	O
ATOM	2234	CB	LYS	A	286	-8.650	5.179	2.218	1.00	27.97	C
ATOM	2235	CG	LYS	A	286	-9.914	5.578	1.425	1.00	19.93	C
ATOM	2236	CD	LYS	A	286	-10.135	4.498	0.364	1.00	19.31	C
ATOM	2237	CE	LYS	A	286	-11.548	4.386	-0.099	1.00	16.08	C
ATOM	2238	NZ	LYS	A	286	-12.013	5.632	-0.734	1.00	16.67	N1+
ATOM	2239	N	SER	A	287	-6.716	4.406	4.244	1.00	19.71	N
ATOM	2240	CA	SER	A	287	-6.403	3.545	5.359	1.00	19.25	C
ATOM	2241	C	SER	A	287	-5.478	4.164	6.423	1.00	19.83	C
ATOM	2242	O	SER	A	287	-4.917	3.451	7.252	1.00	19.56	O
ATOM	2243	CB	SER	A	287	-5.832	2.230	4.832	1.00	22.16	C
ATOM	2244	OG	SER	A	287	-6.709	1.646	3.874	1.00	24.16	O
ATOM	2245	N	HIS	A	288	-5.335	5.486	6.427	1.00	20.41	N
ATOM	2246	CA	HIS	A	288	-4.825	6.194	7.611	1.00	23.21	C
ATOM	2247	C	HIS	A	288	-5.564	7.515	7.785	1.00	24.49	C
ATOM	2248	O	HIS	A	288	-4.955	8.582	7.929	1.00	24.42	O
ATOM	2249	CB	HIS	A	288	-3.315	6.446	7.531	1.00	23.44	C
ATOM	2250	CG	HIS	A	288	-2.712	6.985	8.800	1.00	24.13	C
ATOM	2251	CD2	HIS	A	288	-2.834	8.190	9.411	1.00	22.19	C
ATOM	2252	ND1	HIS	A	288	-1.809	6.267	9.556	1.00	25.67	N
ATOM	2253	CE1	HIS	A	288	-1.401	7.001	10.576	1.00	23.98	C
ATOM	2254	NE2	HIS	A	288	-2.009	8.172	10.512	1.00	26.26	N
ATOM	2255	N	CYS	A	289	-6.865	7.447	7.540	1.00	25.16	N
ATOM	2256	CA	CYS	A	289	-7.819	8.301	8.209	1.00	26.57	C
ATOM	2257	C	CYS	A	289	-8.701	7.377	9.055	1.00	25.76	C
ATOM	2258	O	CYS	A	289	-9.060	7.710	10.192	1.00	26.10	O
ATOM	2259	CB	CYS	A	289	-8.716	9.020	7.218	1.00	26.87	C
ATOM	2260	SG	CYS	A	289	-10.336	9.115	8.027	1.00	35.83	S
ATOM	2261	N	ILE	A	290	-9.231	6.354	8.381	1.00	23.01	N
ATOM	2262	CA	ILE	A	290	-9.982	5.262	8.996	1.00	20.80	C
ATOM	2263	C	ILE	A	290	-9.125	4.551	10.043	1.00	21.11	C
ATOM	2264	O	ILE	A	290	-9.551	4.335	11.168	1.00	22.48	O
ATOM	2265	CB	ILE	A	290	-10.432	4.241	7.920	1.00	17.33	C
ATOM	2266	CG1	ILE	A	290	-11.770	4.670	7.312	1.00	16.61	C
ATOM	2267	CG2	ILE	A	290	-10.558	2.866	8.514	1.00	19.50	C
ATOM	2268	CD1	ILE	A	290	-11.867	4.462	5.808	1.00	11.81	C
ATOM	2269	N	ALA	A	291	-7.920	4.156	9.665	1.00	20.66	N

ATOM	2270	CA	ALA	A	291	-6.948	3.747	10.664	1.00	20.97	C
ATOM	2271	C	ALA	A	291	-6.893	4.911	11.619	1.00	19.91	C
ATOM	2272	O	ALA	A	291	-7.270	6.008	11.249	1.00	18.39	O
ATOM	2273	CB	ALA	A	291	-5.583	3.518	10.024	1.00	21.17	C
ATOM	2274	N	GLU	A	292	-6.302	4.720	12.784	1.00	21.16	N
ATOM	2275	CA	GLU	A	292	-6.572	5.631	13.881	1.00	24.55	C
ATOM	2276	C	GLU	A	292	-6.249	7.130	13.695	1.00	26.59	C
ATOM	2277	O	GLU	A	292	-5.185	7.622	14.100	1.00	27.80	O
ATOM	2278	CB	GLU	A	292	-5.962	5.076	15.170	1.00	24.02	C
ATOM	2279	CG	GLU	A	292	-6.808	3.952	15.736	1.00	23.17	C
ATOM	2280	CD	GLU	A	292	-6.260	3.354	17.010	1.00	25.80	C
ATOM	2281	OE1	GLU	A	292	-6.050	4.132	17.989	1.00	23.17	O
ATOM	2282	OE2	GLU	A	292	-6.112	2.098	17.040	1.00	20.76	O1-
ATOM	2283	N	VAL	A	293	-7.121	7.800	12.940	1.00	26.75	N
ATOM	2284	CA	VAL	A	293	-7.593	9.139	13.260	1.00	26.08	C
ATOM	2285	C	VAL	A	293	-9.082	9.127	12.906	1.00	26.05	C
ATOM	2286	O	VAL	A	293	-9.506	9.564	11.824	1.00	23.87	O
ATOM	2287	CB	VAL	A	293	-6.824	10.236	12.489	1.00	26.52	C
ATOM	2288	CG1	VAL	A	293	-7.586	11.572	12.540	1.00	23.04	C
ATOM	2289	CG2	VAL	A	293	-5.434	10.417	13.116	1.00	25.28	C
ATOM	2290	N	GLU	A	294	-9.845	8.465	13.774	1.00	26.28	N
ATOM	2291	CA	GLU	A	294	-11.252	8.203	13.506	1.00	27.28	C
ATOM	2292	C	GLU	A	294	-12.157	8.786	14.564	1.00	25.18	C
ATOM	2293	O	GLU	A	294	-12.655	8.059	15.407	1.00	25.83	O
ATOM	2294	CB	GLU	A	294	-11.509	6.688	13.403	1.00	29.38	C
ATOM	2295	CG	GLU	A	294	-11.930	6.202	12.013	1.00	27.11	C
ATOM	2296	CD	GLU	A	294	-12.826	7.200	11.307	1.00	28.00	C
ATOM	2297	OE1	GLU	A	294	-12.301	8.229	10.798	1.00	24.31	O
ATOM	2298	OE2	GLU	A	294	-14.056	6.961	11.289	1.00	26.88	O1-
ATOM	2299	N	ASN	A	295	-12.381	10.093	14.525	1.00	23.72	N
ATOM	2300	CA	ASN	A	295	-13.248	10.720	15.510	1.00	21.83	C
ATOM	2301	C	ASN	A	295	-14.523	9.893	15.424	1.00	18.95	C
ATOM	2302	O	ASN	A	295	-14.920	9.519	14.325	1.00	16.49	O
ATOM	2303	CB	ASN	A	295	-13.476	12.217	15.175	1.00	23.98	C
ATOM	2304	CG	ASN	A	295	-13.935	13.068	16.404	1.00	27.75	C
ATOM	2305	ND2	ASN	A	295	-13.404	12.768	17.605	1.00	26.54	N
ATOM	2306	OD1	ASN	A	295	-14.706	14.027	16.242	1.00	27.01	O
ATOM	2307	N	ASP	A	296	-14.757	9.198	16.536	1.00	19.63	N
ATOM	2308	CA	ASP	A	296	-15.900	8.306	16.708	1.00	18.42	C
ATOM	2309	C	ASP	A	296	-17.117	9.146	16.474	1.00	18.35	C
ATOM	2310	O	ASP	A	296	-17.158	10.280	16.940	1.00	19.40	O
ATOM	2311	CB	ASP	A	296	-16.006	7.790	18.155	1.00	17.52	C
ATOM	2312	CG	ASP	A	296	-14.676	7.708	18.867	1.00	16.66	C
ATOM	2313	OD1	ASP	A	296	-14.270	8.699	19.506	1.00	16.57	O
ATOM	2314	OD2	ASP	A	296	-14.096	6.609	18.887	1.00	17.73	O1-
ATOM	2315	N	GLU	A	297	-18.180	8.541	15.963	1.00	20.63	N
ATOM	2316	CA	GLU	A	297	-19.492	9.152	16.137	1.00	22.06	C
ATOM	2317	C	GLU	A	297	-20.176	8.612	17.385	1.00	20.95	C
ATOM	2318	O	GLU	A	297	-21.015	7.728	17.316	1.00	17.62	O
ATOM	2319	CB	GLU	A	297	-20.393	8.974	14.910	1.00	22.28	C
ATOM	2320	CG	GLU	A	297	-21.083	10.290	14.487	1.00	19.78	C
ATOM	2321	CD	GLU	A	297	-22.552	10.114	14.097	1.00	17.46	C
ATOM	2322	OE1	GLU	A	297	-23.107	9.029	14.331	1.00	18.89	O
ATOM	2323	OE2	GLU	A	297	-23.177	11.082	13.618	1.00	17.10	O1-
ATOM	2324	N	MET	A	298	-19.640	9.016	18.530	1.00	21.96	N
ATOM	2325	CA	MET	A	298	-20.458	9.312	19.694	1.00	23.40	C
ATOM	2326	C	MET	A	298	-21.712	10.057	19.184	1.00	25.29	C
ATOM	2327	O	MET	A	298	-21.650	10.760	18.154	1.00	23.28	O
ATOM	2328	CB	MET	A	298	-19.659	10.192	20.679	1.00	23.66	C
ATOM	2329	CG	MET	A	298	-19.890	11.713	20.580	1.00	23.57	C
ATOM	2330	SD	MET	A	298	-21.327	12.337	21.577	1.00	29.35	S
ATOM	2331	CE	MET	A	298	-20.736	14.067	22.065	1.00	21.21	C
ATOM	2332	N	PRO	A	299	-22.855	9.926	19.902	1.00	25.70	N
ATOM	2333	CA	PRO	A	299	-22.933	9.341	21.242	1.00	23.52	C
ATOM	2334	C	PRO	A	299	-23.444	7.894	21.195	1.00	22.57	C
ATOM	2335	O	PRO	A	299	-22.991	7.044	21.985	1.00	18.81	O
ATOM	2336	CB	PRO	A	299	-23.889	10.297	21.982	1.00	24.67	C
ATOM	2337	CG	PRO	A	299	-24.523	11.190	20.892	1.00	23.48	C
ATOM	2338	CD	PRO	A	299	-24.126	10.590	19.566	1.00	26.37	C
ATOM	2339	N	ALA	A	300	-24.449	7.663	20.336	1.00	23.45	N
ATOM	2340	CA	ALA	A	300	-24.730	6.330	19.769	1.00	24.55	C

ATOM	2341	C	ALA	A	300	-26.034	6.174	18.960	1.00	23.16	C
ATOM	2342	O	ALA	A	300	-26.739	7.152	18.691	1.00	21.49	O
ATOM	2343	CB	ALA	A	300	-24.655	5.248	20.867	1.00	26.49	C
ATOM	2344	N	ASP	A	301	-26.398	4.908	18.733	1.00	24.12	N
ATOM	2345	CA	ASP	A	301	-27.360	4.437	17.718	1.00	25.32	C
ATOM	2346	C	ASP	A	301	-28.711	5.075	17.818	1.00	26.66	C
ATOM	2347	O	ASP	A	301	-29.504	4.991	16.890	1.00	27.53	O
ATOM	2348	CB	ASP	A	301	-27.548	2.919	17.890	1.00	26.16	C
ATOM	2349	CG	ASP	A	301	-28.362	2.254	16.767	1.00	27.64	C
ATOM	2350	OD1	ASP	A	301	-28.582	2.850	15.681	1.00	27.63	O
ATOM	2351	OD2	ASP	A	301	-28.674	1.051	16.947	1.00	26.67	O1-
ATOM	2352	N	LEU	A	302	-28.958	5.727	18.946	1.00	30.35	N
ATOM	2353	CA	LEU	A	302	-30.086	5.351	19.792	1.00	31.56	C
ATOM	2354	C	LEU	A	302	-30.537	6.488	20.686	1.00	33.72	C
ATOM	2355	O	LEU	A	302	-29.798	7.474	20.867	1.00	34.95	O
ATOM	2356	CB	LEU	A	302	-29.647	4.182	20.653	1.00	28.92	C
ATOM	2357	CG	LEU	A	302	-28.155	4.371	20.967	1.00	31.25	C
ATOM	2358	CD1	LEU	A	302	-27.961	5.472	22.020	1.00	31.44	C
ATOM	2359	CD2	LEU	A	302	-27.489	3.041	21.378	1.00	32.08	C
ATOM	2360	N	PRO	A	303	-31.584	6.224	21.488	1.00	35.90	N
ATOM	2361	CA	PRO	A	303	-31.430	6.284	22.954	1.00	38.93	C
ATOM	2362	C	PRO	A	303	-30.874	4.971	23.513	1.00	38.86	C
ATOM	2363	O	PRO	A	303	-29.892	4.975	24.269	1.00	38.96	O
ATOM	2364	CB	PRO	A	303	-32.853	6.564	23.459	1.00	38.70	C
ATOM	2365	CG	PRO	A	303	-33.766	6.592	22.142	1.00	38.82	C
ATOM	2366	CD	PRO	A	303	-32.961	5.875	21.101	1.00	36.51	C
ATOM	2367	N	SER	A	304	-31.360	3.855	22.964	1.00	37.24	N
ATOM	2368	CA	SER	A	304	-31.097	2.522	23.528	1.00	35.92	C
ATOM	2369	C	SER	A	304	-31.120	2.445	25.034	1.00	32.14	C
ATOM	2370	O	SER	A	304	-30.681	1.441	25.597	1.00	30.33	O
ATOM	2371	CB	SER	A	304	-29.774	1.948	23.042	1.00	36.77	C
ATOM	2372	OG	SER	A	304	-30.029	0.971	22.042	1.00	42.09	O
ATOM	2373	N	LEU	A	305	-31.942	3.312	25.607	1.00	30.13	N
ATOM	2374	CA	LEU	A	305	-32.018	3.481	27.031	1.00	30.37	C
ATOM	2375	C	LEU	A	305	-30.913	2.663	27.730	1.00	31.82	C
ATOM	2376	O	LEU	A	305	-29.749	3.000	27.555	1.00	35.56	O
ATOM	2377	CB	LEU	A	305	-33.405	3.084	27.532	1.00	29.63	C
ATOM	2378	CG	LEU	A	305	-34.657	3.305	26.681	1.00	28.08	C
ATOM	2379	CD1	LEU	A	305	-35.835	3.366	27.647	1.00	28.23	C
ATOM	2380	CD2	LEU	A	305	-34.583	4.576	25.848	1.00	26.17	C
ATOM	2381	N	ALA	A	306	-31.218	1.418	28.097	1.00	28.81	N
ATOM	2382	CA	ALA	A	306	-30.480	0.759	29.160	1.00	25.85	C
ATOM	2383	C	ALA	A	306	-30.048	-0.635	28.744	1.00	24.00	C
ATOM	2384	O	ALA	A	306	-30.699	-1.241	27.912	1.00	23.85	O
ATOM	2385	CB	ALA	A	306	-31.353	0.688	30.406	1.00	28.20	C
ATOM	2386	N	ALA	A	307	-29.050	-1.198	29.422	1.00	22.64	N
ATOM	2387	CA	ALA	A	307	-28.519	-2.505	29.043	1.00	24.62	C
ATOM	2388	C	ALA	A	307	-28.896	-3.578	30.063	1.00	25.94	C
ATOM	2389	O	ALA	A	307	-28.053	-4.112	30.807	1.00	22.41	O
ATOM	2390	CB	ALA	A	307	-26.990	-2.432	28.860	1.00	26.11	C
ATOM	2391	N	ASP	A	308	-30.187	-3.895	30.092	1.00	28.91	N
ATOM	2392	CA	ASP	A	308	-30.732	-4.831	31.078	1.00	27.05	C
ATOM	2393	C	ASP	A	308	-31.350	-6.031	30.350	1.00	26.11	C
ATOM	2394	O	ASP	A	308	-32.535	-6.372	30.516	1.00	24.92	O
ATOM	2395	CB	ASP	A	308	-31.721	-4.099	32.001	1.00	25.32	C
ATOM	2396	CG	ASP	A	308	-31.006	-3.359	33.154	1.00	25.24	C
ATOM	2397	OD1	ASP	A	308	-29.958	-3.840	33.629	1.00	19.08	O
ATOM	2398	OD2	ASP	A	308	-31.452	-2.267	33.552	1.00	27.30	O1-
ATOM	2399	N	PHE	A	309	-30.575	-6.493	29.370	1.00	21.83	N
ATOM	2400	CA	PHE	A	309	-30.779	-7.762	28.711	1.00	18.30	C
ATOM	2401	C	PHE	A	309	-29.716	-8.657	29.238	1.00	15.33	C
ATOM	2402	O	PHE	A	309	-29.757	-9.841	29.018	1.00	18.02	O
ATOM	2403	CB	PHE	A	309	-30.554	-7.614	27.217	1.00	21.96	C
ATOM	2404	CG	PHE	A	309	-31.727	-8.017	26.378	1.00	22.43	C
ATOM	2405	CD1	PHE	A	309	-33.007	-8.013	26.902	1.00	21.17	C
ATOM	2406	CD2	PHE	A	309	-31.532	-8.450	25.084	1.00	21.60	C
ATOM	2407	CE1	PHE	A	309	-34.060	-8.440	26.163	1.00	22.58	C
ATOM	2408	CE2	PHE	A	309	-32.577	-8.876	24.337	1.00	24.09	C
ATOM	2409	CZ	PHE	A	309	-33.851	-8.876	24.874	1.00	26.92	C
ATOM	2410	N	VAL	A	310	-28.619	-8.040	29.631	1.00	17.52	N
ATOM	2411	CA	VAL	A	310	-27.491	-8.754	30.234	1.00	21.16	C

ATOM	2412	C	VAL	A	310	-27.676	-8.866	31.760	1.00	22.19	C
ATOM	2413	O	VAL	A	310	-26.735	-9.228	32.476	1.00	19.99	O
ATOM	2414	CB	VAL	A	310	-26.093	-8.031	29.910	1.00	22.76	C
ATOM	2415	CG1	VAL	A	310	-26.078	-7.481	28.455	1.00	16.58	C
ATOM	2416	CG2	VAL	A	310	-25.796	-6.903	30.929	1.00	19.12	C
ATOM	2417	N	GLU	A	311	-28.904	-8.615	32.231	1.00	24.86	N
ATOM	2418	CA	GLU	A	311	-29.132	-8.190	33.620	1.00	25.85	C
ATOM	2419	C	GLU	A	311	-30.462	-8.620	34.250	1.00	26.80	C
ATOM	2420	O	GLU	A	311	-30.617	-9.786	34.614	1.00	26.88	O
ATOM	2421	CB	GLU	A	311	-28.997	-6.672	33.724	1.00	24.55	C
ATOM	2422	CG	GLU	A	311	-28.150	-6.231	34.893	1.00	24.81	C
ATOM	2423	CD	GLU	A	311	-28.390	-7.071	36.097	1.00	22.49	C
ATOM	2424	OE1	GLU	A	311	-29.486	-6.952	36.659	1.00	27.72	O
ATOM	2425	OE2	GLU	A	311	-27.551	-7.932	36.415	1.00	21.47	O1-
ATOM	2426	N	SER	A	312	-31.340	-7.646	34.518	1.00	27.11	N
ATOM	2427	CA	SER	A	312	-32.668	-7.899	35.086	1.00	28.09	C
ATOM	2428	C	SER	A	312	-33.508	-8.811	34.212	1.00	29.00	C
ATOM	2429	O	SER	A	312	-34.603	-8.413	33.813	1.00	32.24	O
ATOM	2430	CB	SER	A	312	-33.463	-6.594	35.244	1.00	25.60	C
ATOM	2431	OG	SER	A	312	-32.912	-5.751	36.227	1.00	29.41	O
ATOM	2432	N	LYS	A	313	-33.071	-10.047	34.005	1.00	27.40	N
ATOM	2433	CA	LYS	A	313	-33.782	-10.965	33.129	1.00	27.14	C
ATOM	2434	C	LYS	A	313	-33.672	-12.368	33.711	1.00	30.75	C
ATOM	2435	O	LYS	A	313	-33.007	-12.566	34.740	1.00	31.32	O
ATOM	2436	CB	LYS	A	313	-33.153	-10.936	31.738	1.00	21.94	C
ATOM	2437	CG	LYS	A	313	-33.859	-11.786	30.716	1.00	17.30	C
ATOM	2438	CD	LYS	A	313	-34.578	-10.916	29.723	1.00	15.90	C
ATOM	2439	CE	LYS	A	313	-34.503	-11.512	28.346	1.00	12.19	C
ATOM	2440	NZ	LYS	A	313	-33.124	-11.478	27.875	1.00	14.13	N1+
ATOM	2441	N	ASP	A	314	-34.367	-13.332	33.102	1.00	33.04	N
ATOM	2442	CA	ASP	A	314	-33.947	-14.726	33.210	1.00	34.53	C
ATOM	2443	C	ASP	A	314	-33.696	-15.303	31.820	1.00	34.78	C
ATOM	2444	O	ASP	A	314	-34.638	-15.649	31.091	1.00	34.31	O
ATOM	2445	CB	ASP	A	314	-34.962	-15.587	33.974	1.00	34.53	C
ATOM	2446	CG	ASP	A	314	-34.538	-17.065	34.066	1.00	36.92	C
ATOM	2447	OD1	ASP	A	314	-33.379	-17.438	33.692	1.00	33.31	O
ATOM	2448	OD2	ASP	A	314	-35.415	-17.878	34.458	1.00	41.34	O1-
ATOM	2449	N	VAL	A	315	-32.463	-15.055	31.384	1.00	33.55	N
ATOM	2450	CA	VAL	A	315	-31.752	-15.855	30.402	1.00	30.37	C
ATOM	2451	C	VAL	A	315	-30.272	-15.815	30.829	1.00	28.87	C
ATOM	2452	O	VAL	A	315	-29.493	-14.952	30.414	1.00	25.57	O
ATOM	2453	CB	VAL	A	315	-31.929	-15.287	28.979	1.00	32.83	C
ATOM	2454	CG1	VAL	A	315	-33.417	-15.436	28.512	1.00	29.71	C
ATOM	2455	CG2	VAL	A	315	-31.453	-13.820	28.934	1.00	33.25	C
ATOM	2456	N	CYS	A	316	-30.032	-16.517	31.931	1.00	29.88	N
ATOM	2457	CA	CYS	A	316	-28.683	-16.794	32.423	1.00	27.73	C
ATOM	2458	C	CYS	A	316	-27.953	-17.584	31.361	1.00	26.33	C
ATOM	2459	O	CYS	A	316	-27.153	-17.013	30.620	1.00	26.76	O
ATOM	2460	CB	CYS	A	316	-28.734	-17.628	33.688	1.00	27.85	C
ATOM	2461	SG	CYS	A	316	-27.077	-18.069	34.276	1.00	31.37	S
ATOM	2462	N	LYS	A	317	-28.313	-18.863	31.217	1.00	22.54	N
ATOM	2463	CA	LYS	A	317	-27.900	-19.631	30.046	1.00	21.29	C
ATOM	2464	C	LYS	A	317	-28.905	-19.504	28.912	1.00	22.46	C
ATOM	2465	O	LYS	A	317	-28.510	-19.220	27.790	1.00	22.63	O
ATOM	2466	CB	LYS	A	317	-27.693	-21.120	30.369	1.00	18.85	C
ATOM	2467	CG	LYS	A	317	-26.247	-21.541	30.640	1.00	18.02	C
ATOM	2468	CD	LYS	A	317	-26.179	-22.731	31.604	1.00	20.06	C
ATOM	2469	CE	LYS	A	317	-24.770	-22.943	32.219	1.00	22.33	C
ATOM	2470	NZ	LYS	A	317	-24.375	-24.401	32.437	1.00	22.00	N1+
ATOM	2471	N	ASN	A	318	-30.201	-19.646	29.199	1.00	22.97	N
ATOM	2472	CA	ASN	A	318	-31.215	-19.864	28.143	1.00	22.32	C
ATOM	2473	C	ASN	A	318	-30.963	-19.181	26.798	1.00	24.26	C
ATOM	2474	O	ASN	A	318	-31.447	-19.657	25.759	1.00	26.24	O
ATOM	2475	CB	ASN	A	318	-32.607	-19.439	28.601	1.00	19.64	C
ATOM	2476	CG	ASN	A	318	-33.659	-19.693	27.542	1.00	17.20	C
ATOM	2477	ND2	ASN	A	318	-33.852	-18.753	26.630	1.00	17.37	N
ATOM	2478	OD1	ASN	A	318	-34.234	-20.753	27.507	1.00	19.89	O
ATOM	2479	N	TYR	A	319	-30.390	-17.978	26.856	1.00	23.97	N
ATOM	2480	CA	TYR	A	319	-30.222	-17.095	25.694	1.00	24.24	C
ATOM	2481	C	TYR	A	319	-28.722	-16.854	25.621	1.00	23.49	C
ATOM	2482	O	TYR	A	319	-28.250	-15.857	25.088	1.00	22.66	O

ATOM	2483	CB	TYR	A	319	-31.022	-15.804	25.948	1.00	23.01	C
ATOM	2484	CG	TYR	A	319	-30.749	-14.587	25.094	1.00	22.30	C
ATOM	2485	CD1	TYR	A	319	-31.440	-14.393	23.908	1.00	24.18	C
ATOM	2486	CD2	TYR	A	319	-30.040	-13.502	25.616	1.00	20.42	C
ATOM	2487	CE1	TYR	A	319	-31.453	-13.148	23.278	1.00	23.68	C
ATOM	2488	CE2	TYR	A	319	-30.044	-12.265	24.993	1.00	17.71	C
ATOM	2489	CZ	TYR	A	319	-30.751	-12.097	23.826	1.00	20.42	C
ATOM	2490	OH	TYR	A	319	-30.732	-10.898	23.159	1.00	22.03	O
ATOM	2491	N	ALA	A	320	-27.993	-17.889	25.995	1.00	21.15	N
ATOM	2492	CA	ALA	A	320	-26.572	-17.798	26.227	1.00	23.56	C
ATOM	2493	C	ALA	A	320	-26.032	-19.198	26.089	1.00	25.78	C
ATOM	2494	O	ALA	A	320	-25.428	-19.751	27.004	1.00	25.36	O
ATOM	2495	CB	ALA	A	320	-26.290	-17.267	27.618	1.00	24.04	C
ATOM	2496	N	GLU	A	321	-26.398	-19.828	24.985	1.00	28.84	N
ATOM	2497	CA	GLU	A	321	-25.690	-21.008	24.536	1.00	27.99	C
ATOM	2498	C	GLU	A	321	-24.907	-20.746	23.257	1.00	25.24	C
ATOM	2499	O	GLU	A	321	-24.235	-21.634	22.715	1.00	27.19	O
ATOM	2500	CB	GLU	A	321	-26.658	-22.167	24.388	1.00	30.20	C
ATOM	2501	CG	GLU	A	321	-26.266	-23.326	25.298	1.00	37.63	C
ATOM	2502	CD	GLU	A	321	-25.831	-22.921	26.736	1.00	38.24	C
ATOM	2503	OE1	GLU	A	321	-24.725	-22.329	26.915	1.00	36.30	O
ATOM	2504	OE2	GLU	A	321	-26.509	-23.388	27.699	1.00	40.13	O1-
ATOM	2505	N	ALA	A	322	-24.889	-19.483	22.857	1.00	20.08	N
ATOM	2506	CA	ALA	A	322	-23.864	-19.005	21.965	1.00	17.91	C
ATOM	2507	C	ALA	A	322	-22.817	-18.388	22.879	1.00	16.32	C
ATOM	2508	O	ALA	A	322	-23.051	-17.332	23.441	1.00	17.37	O
ATOM	2509	CB	ALA	A	322	-24.437	-17.962	21.032	1.00	16.29	C
ATOM	2510	N	LYS	A	323	-21.746	-19.116	23.166	1.00	13.15	N
ATOM	2511	CA	LYS	A	323	-20.707	-18.569	24.019	1.00	13.43	C
ATOM	2512	C	LYS	A	323	-20.251	-17.244	23.408	1.00	15.07	C
ATOM	2513	O	LYS	A	323	-20.751	-16.180	23.773	1.00	14.91	O
ATOM	2514	CB	LYS	A	323	-19.537	-19.560	24.124	1.00	14.15	C
ATOM	2515	CG	LYS	A	323	-18.201	-18.956	24.571	1.00	15.59	C
ATOM	2516	CD	LYS	A	323	-17.235	-20.027	25.079	1.00	14.64	C
ATOM	2517	CE	LYS	A	323	-15.850	-19.935	24.450	1.00	14.18	C
ATOM	2518	NZ	LYS	A	323	-15.145	-18.696	24.813	1.00	15.36	N1+
ATOM	2519	N	ASP	A	324	-19.608	-17.372	22.252	1.00	17.96	N
ATOM	2520	CA	ASP	A	324	-18.847	-16.299	21.623	1.00	15.80	C
ATOM	2521	C	ASP	A	324	-19.718	-15.035	21.411	1.00	17.56	C
ATOM	2522	O	ASP	A	324	-19.304	-13.924	21.781	1.00	17.35	O
ATOM	2523	CB	ASP	A	324	-18.233	-16.790	20.275	1.00	15.91	C
ATOM	2524	CG	ASP	A	324	-17.787	-18.293	20.293	1.00	16.85	C
ATOM	2525	OD1	ASP	A	324	-16.749	-18.623	20.903	1.00	15.85	O
ATOM	2526	OD2	ASP	A	324	-18.420	-19.140	19.617	1.00	17.67	O1-
ATOM	2527	N	VAL	A	325	-20.930	-15.180	20.870	1.00	15.39	N
ATOM	2528	CA	VAL	A	325	-21.660	-13.979	20.467	1.00	18.29	C
ATOM	2529	C	VAL	A	325	-22.388	-13.305	21.613	1.00	20.30	C
ATOM	2530	O	VAL	A	325	-22.751	-12.137	21.509	1.00	20.95	O
ATOM	2531	CB	VAL	A	325	-22.693	-14.215	19.333	1.00	18.19	C
ATOM	2532	CG1	VAL	A	325	-22.039	-14.874	18.125	1.00	17.25	C
ATOM	2533	CG2	VAL	A	325	-23.875	-15.003	19.849	1.00	19.92	C
ATOM	2534	N	PHE	A	326	-22.608	-14.039	22.701	1.00	23.24	N
ATOM	2535	CA	PHE	A	326	-23.259	-13.497	23.893	1.00	22.88	C
ATOM	2536	C	PHE	A	326	-22.336	-12.431	24.476	1.00	23.49	C
ATOM	2537	O	PHE	A	326	-22.675	-11.242	24.548	1.00	20.07	O
ATOM	2538	CB	PHE	A	326	-23.474	-14.624	24.911	1.00	23.89	C
ATOM	2539	CG	PHE	A	326	-24.307	-14.229	26.092	1.00	25.04	C
ATOM	2540	CD1	PHE	A	326	-25.603	-13.781	25.923	1.00	25.47	C
ATOM	2541	CD2	PHE	A	326	-23.776	-14.256	27.367	1.00	24.57	C
ATOM	2542	CE1	PHE	A	326	-26.356	-13.354	27.016	1.00	26.31	C
ATOM	2543	CE2	PHE	A	326	-24.522	-13.830	28.453	1.00	25.69	C
ATOM	2544	CZ	PHE	A	326	-25.814	-13.375	28.277	1.00	24.50	C
ATOM	2545	N	LEU	A	327	-21.109	-12.854	24.753	1.00	24.46	N
ATOM	2546	CA	LEU	A	327	-20.056	-11.948	25.200	1.00	25.44	C
ATOM	2547	C	LEU	A	327	-19.866	-10.805	24.193	1.00	23.62	C
ATOM	2548	O	LEU	A	327	-19.782	-9.623	24.568	1.00	18.87	O
ATOM	2549	CB	LEU	A	327	-18.744	-12.727	25.357	1.00	25.68	C
ATOM	2550	CG	LEU	A	327	-18.569	-13.566	26.624	1.00	22.99	C
ATOM	2551	CD1	LEU	A	327	-18.534	-12.630	27.851	1.00	21.08	C
ATOM	2552	CD2	LEU	A	327	-19.688	-14.578	26.719	1.00	21.68	C
ATOM	2553	N	GLY	A	328	-19.861	-11.175	22.911	1.00	22.86	N

```
ATOM   2554  CA  GLY A 328     -19.715 -10.197  21.850  1.00 19.96           C
ATOM   2555  C   GLY A 328     -20.775  -9.121  21.937  1.00 18.31           C
ATOM   2556  O   GLY A 328     -20.496  -7.961  21.651  1.00 14.99           O
ATOM   2557  N   MET A 329     -21.988  -9.503  22.332  1.00 18.03           N
ATOM   2558  CA  MET A 329     -23.095  -8.563  22.406  1.00 16.11           C
ATOM   2559  C   MET A 329     -23.171  -7.903  23.772  1.00 16.92           C
ATOM   2560  O   MET A 329     -23.768  -6.838  23.930  1.00 12.56           O
ATOM   2561  CB  MET A 329     -24.412  -9.253  22.090  1.00 15.19           C
ATOM   2562  CG  MET A 329     -25.567  -8.288  22.048  1.00 16.53           C
ATOM   2563  SD  MET A 329     -26.910  -8.847  21.026  1.00 19.89           S
ATOM   2564  CE  MET A 329     -27.585  -7.279  20.531  1.00 17.00           C
ATOM   2565  N   PHE A 330     -22.516  -8.495  24.761  1.00 18.68           N
ATOM   2566  CA  PHE A 330     -22.249  -7.713  25.953  1.00 20.67           C
ATOM   2567  C   PHE A 330     -21.302  -6.630  25.467  1.00 20.74           C
ATOM   2568  O   PHE A 330     -21.793  -5.643  24.911  1.00 18.89           O
ATOM   2569  CB  PHE A 330     -21.628  -8.544  27.091  1.00 19.97           C
ATOM   2570  CG  PHE A 330     -21.607  -7.824  28.417  1.00 17.23           C
ATOM   2571  CD1 PHE A 330     -22.552  -6.854  28.710  1.00 16.05           C
ATOM   2572  CD2 PHE A 330     -20.594  -8.041  29.323  1.00 16.93           C
ATOM   2573  CE1 PHE A 330     -22.478  -6.118  29.854  1.00 13.30           C
ATOM   2574  CE2 PHE A 330     -20.523  -7.294  30.483  1.00 15.90           C
ATOM   2575  CZ  PHE A 330     -21.464  -6.336  30.738  1.00 14.36           C
ATOM   2576  N   LEU A 331     -20.019  -7.003  25.336  1.00 21.76           N
ATOM   2577  CA  LEU A 331     -18.930  -6.124  24.885  1.00 22.65           C
ATOM   2578  C   LEU A 331     -19.431  -4.988  24.012  1.00 26.84           C
ATOM   2579  O   LEU A 331     -19.549  -3.859  24.503  1.00 30.55           O
ATOM   2580  CB  LEU A 331     -17.900  -6.919  24.093  1.00 21.01           C
ATOM   2581  CG  LEU A 331     -16.415  -6.566  24.178  1.00 21.98           C
ATOM   2582  CD1 LEU A 331     -16.125  -5.467  25.205  1.00 18.67           C
ATOM   2583  CD2 LEU A 331     -15.674  -7.848  24.537  1.00 19.61           C
ATOM   2584  N   TYR A 332     -19.930  -5.338  22.816  1.00 27.17           N
ATOM   2585  CA  TYR A 332     -20.406  -4.377  21.794  1.00 24.12           C
ATOM   2586  C   TYR A 332     -21.396  -3.374  22.322  1.00 22.23           C
ATOM   2587  O   TYR A 332     -21.229  -2.187  22.105  1.00 22.76           O
ATOM   2588  CB  TYR A 332     -21.021  -5.134  20.599  1.00 22.53           C
ATOM   2589  CG  TYR A 332     -22.199  -4.494  19.884  1.00 16.65           C
ATOM   2590  CD1 TYR A 332     -22.123  -3.225  19.365  1.00 17.03           C
ATOM   2591  CD2 TYR A 332     -23.319  -5.242  19.579  1.00 15.91           C
ATOM   2592  CE1 TYR A 332     -23.132  -2.727  18.564  1.00 15.87           C
ATOM   2593  CE2 TYR A 332     -24.309  -4.760  18.779  1.00 12.78           C
ATOM   2594  CZ  TYR A 332     -24.219  -3.503  18.270  1.00 12.74           C
ATOM   2595  OH  TYR A 332     -25.211  -3.032  17.443  1.00 11.75           O
ATOM   2596  N   GLU A 333     -22.462  -3.858  22.947  1.00 21.11           N
ATOM   2597  CA  GLU A 333     -23.480  -2.982  23.516  1.00 19.69           C
ATOM   2598  C   GLU A 333     -22.973  -2.225  24.753  1.00 19.94           C
ATOM   2599  O   GLU A 333     -23.239  -1.039  24.920  1.00 21.44           O
ATOM   2600  CB  GLU A 333     -24.722  -3.791  23.854  1.00 17.19           C
ATOM   2601  CG  GLU A 333     -25.676  -3.903  22.700  1.00 13.27           C
ATOM   2602  CD  GLU A 333     -26.462  -2.640  22.499  1.00 16.80           C
ATOM   2603  OE1 GLU A 333     -26.673  -1.903  23.474  1.00 17.89           O
ATOM   2604  OE2 GLU A 333     -26.870  -2.358  21.358  1.00 21.21           O1-
ATOM   2605  N   TYR A 334     -22.108  -2.846  25.535  1.00 17.50           N
ATOM   2606  CA  TYR A 334     -21.473  -2.102  26.591  1.00 16.87           C
ATOM   2607  C   TYR A 334     -20.552  -1.074  25.929  1.00 17.20           C
ATOM   2608  O   TYR A 334     -20.643   0.126  26.220  1.00 15.06           O
ATOM   2609  CB  TYR A 334     -20.694  -3.049  27.511  1.00 19.62           C
ATOM   2610  CG  TYR A 334     -20.693  -2.600  28.969  1.00 21.56           C
ATOM   2611  CD1 TYR A 334     -20.828  -1.258  29.304  1.00 23.27           C
ATOM   2612  CD2 TYR A 334     -20.546  -3.503  29.994  1.00 19.88           C
ATOM   2613  CE1 TYR A 334     -20.808  -0.840  30.609  1.00 21.45           C
ATOM   2614  CE2 TYR A 334     -20.523  -3.084  31.297  1.00 21.56           C
ATOM   2615  CZ  TYR A 334     -20.643  -1.750  31.594  1.00 21.82           C
ATOM   2616  OH  TYR A 334     -20.475  -1.288  32.873  1.00 24.16           O
ATOM   2617  N   ALA A 335     -19.934  -1.510  24.833  1.00 16.03           N
ATOM   2618  CA  ALA A 335     -18.944  -0.733  24.108  1.00 15.10           C
ATOM   2619  C   ALA A 335     -19.581   0.539  23.577  1.00 16.13           C
ATOM   2620  O   ALA A 335     -19.116   1.638  23.861  1.00 17.41           O
ATOM   2621  CB  ALA A 335     -18.392  -1.555  22.966  1.00 13.10           C
ATOM   2622  N   ARG A 336     -20.753   0.374  22.974  1.00 16.93           N
ATOM   2623  CA  ARG A 336     -21.547   1.458  22.418  1.00 16.89           C
ATOM   2624  C   ARG A 336     -21.658   2.712  23.290  1.00 18.08           C
```

ATOM	2625	O	ARG	A	336	-21.482	3.820	22.803	1.00	16.00	O
ATOM	2626	CB	ARG	A	336	-22.946	0.940	22.127	1.00	17.05	C
ATOM	2627	CG	ARG	A	336	-23.108	0.324	20.776	1.00	16.01	C
ATOM	2628	CD	ARG	A	336	-24.129	1.117	19.996	1.00	18.77	C
ATOM	2629	NE	ARG	A	336	-25.334	0.350	19.735	1.00	23.35	N
ATOM	2630	CZ	ARG	A	336	-25.554	-0.337	18.613	1.00	26.57	C
ATOM	2631	NH1	ARG	A	336	-24.595	-0.458	17.700	1.00	24.51	N1+
ATOM	2632	NH2	ARG	A	336	-26.743	-0.891	18.390	1.00	27.93	N
ATOM	2633	N	ARG	A	337	-22.052	2.543	24.548	1.00	21.47	N
ATOM	2634	CA	ARG	A	337	-22.449	3.689	25.383	1.00	24.30	C
ATOM	2635	C	ARG	A	337	-21.309	4.239	26.248	1.00	23.58	C
ATOM	2636	O	ARG	A	337	-21.539	4.923	27.280	1.00	21.32	O
ATOM	2637	CB	ARG	A	337	-23.657	3.342	26.285	1.00	27.68	C
ATOM	2638	CG	ARG	A	337	-24.089	1.856	26.376	1.00	29.06	C
ATOM	2639	CD	ARG	A	337	-25.460	1.731	27.076	1.00	29.77	C
ATOM	2640	NE	ARG	A	337	-26.418	0.886	26.353	1.00	28.59	N
ATOM	2641	CZ	ARG	A	337	-26.845	1.102	25.108	1.00	28.23	C
ATOM	2642	NH1	ARG	A	337	-26.324	2.083	24.382	1.00	27.06	N1+
ATOM	2643	NH2	ARG	A	337	-27.795	0.324	24.582	1.00	24.49	N
ATOM	2644	N	HIS	A	338	-20.082	3.889	25.864	1.00	21.05	N
ATOM	2645	CA	HIS	A	338	-18.922	4.164	26.703	1.00	18.37	C
ATOM	2646	C	HIS	A	338	-17.702	4.574	25.901	1.00	13.99	C
ATOM	2647	O	HIS	A	338	-16.611	4.073	26.118	1.00	11.40	O
ATOM	2648	CB	HIS	A	338	-18.610	2.954	27.576	1.00	19.06	C
ATOM	2649	CG	HIS	A	338	-19.551	2.791	28.728	1.00	21.75	C
ATOM	2650	CD2	HIS	A	338	-19.937	1.685	29.406	1.00	21.31	C
ATOM	2651	ND1	HIS	A	338	-20.238	3.847	29.288	1.00	22.04	N
ATOM	2652	CE1	HIS	A	338	-21.007	3.394	30.267	1.00	25.17	C
ATOM	2653	NE2	HIS	A	338	-20.844	2.087	30.358	1.00	21.03	N
ATOM	2654	N	PRO	A	339	-17.826	5.673	25.169	1.00	10.97	N
ATOM	2655	CA	PRO	A	339	-16.623	6.285	24.652	1.00	12.13	C
ATOM	2656	C	PRO	A	339	-15.808	6.864	25.780	1.00	14.82	C
ATOM	2657	O	PRO	A	339	-14.743	7.430	25.543	1.00	16.89	O
ATOM	2658	CB	PRO	A	339	-17.148	7.379	23.719	1.00	12.95	C
ATOM	2659	CG	PRO	A	339	-18.636	7.237	23.705	1.00	11.47	C
ATOM	2660	CD	PRO	A	339	-18.985	6.546	24.970	1.00	12.68	C
ATOM	2661	N	ASP	A	340	-16.335	6.799	26.998	1.00	16.39	N
ATOM	2662	CA	ASP	A	340	-15.676	7.456	28.120	1.00	17.93	C
ATOM	2663	C	ASP	A	340	-14.888	6.494	29.012	1.00	16.22	C
ATOM	2664	O	ASP	A	340	-14.163	6.926	29.905	1.00	13.33	O
ATOM	2665	CB	ASP	A	340	-16.689	8.324	28.916	1.00	23.67	C
ATOM	2666	CG	ASP	A	340	-17.750	7.512	29.671	1.00	28.01	C
ATOM	2667	OD1	ASP	A	340	-18.805	7.174	29.077	1.00	27.93	O
ATOM	2668	OD2	ASP	A	340	-17.630	7.403	30.917	1.00	31.19	O1-
ATOM	2669	N	TYR	A	341	-14.848	5.230	28.586	1.00	16.78	N
ATOM	2670	CA	TYR	A	341	-14.063	4.179	29.225	1.00	16.62	C
ATOM	2671	C	TYR	A	341	-13.182	3.548	28.151	1.00	18.04	C
ATOM	2672	O	TYR	A	341	-13.662	3.157	27.095	1.00	19.19	O
ATOM	2673	CB	TYR	A	341	-14.956	3.059	29.782	1.00	17.89	C
ATOM	2674	CG	TYR	A	341	-15.863	3.378	30.973	1.00	17.57	C
ATOM	2675	CD1	TYR	A	341	-15.606	4.437	31.821	1.00	16.92	C
ATOM	2676	CD2	TYR	A	341	-16.915	2.536	31.290	1.00	15.97	C
ATOM	2677	CE1	TYR	A	341	-16.361	4.638	32.959	1.00	20.41	C
ATOM	2678	CE2	TYR	A	341	-17.666	2.723	32.406	1.00	18.32	C
ATOM	2679	CZ	TYR	A	341	-17.393	3.772	33.260	1.00	20.96	C
ATOM	2680	OH	TYR	A	341	-18.120	3.905	34.452	1.00	18.90	O
ATOM	2681	N	SER	A	342	-11.972	3.194	28.546	1.00	19.93	N
ATOM	2682	CA	SER	A	342	-10.979	2.592	27.668	1.00	17.81	C
ATOM	2683	C	SER	A	342	-11.334	1.153	27.386	1.00	17.13	C
ATOM	2684	O	SER	A	342	-11.554	0.401	28.331	1.00	17.80	O
ATOM	2685	CB	SER	A	342	-9.643	2.595	28.389	1.00	19.87	C
ATOM	2686	OG	SER	A	342	-9.820	2.057	29.693	1.00	17.44	O
ATOM	2687	N	VAL	A	343	-11.037	0.712	26.168	1.00	16.67	N
ATOM	2688	CA	VAL	A	343	-11.195	-0.679	25.788	1.00	15.06	C
ATOM	2689	C	VAL	A	343	-10.570	-1.594	26.815	1.00	15.29	C
ATOM	2690	O	VAL	A	343	-11.284	-2.267	27.538	1.00	17.32	O
ATOM	2691	CB	VAL	A	343	-10.601	-0.964	24.386	1.00	17.69	C
ATOM	2692	CG1	VAL	A	343	-9.194	-0.425	24.270	1.00	17.11	C
ATOM	2693	CG2	VAL	A	343	-10.638	-2.460	24.091	1.00	19.44	C
ATOM	2694	N	VAL	A	344	-9.273	-1.446	27.044	1.00	14.96	N
ATOM	2695	CA	VAL	A	344	-8.571	-2.281	28.013	1.00	12.81	C

```
ATOM   2696  C   VAL A 344      -9.315  -2.430  29.334  1.00 14.93           C
ATOM   2697  O   VAL A 344      -9.358  -3.522  29.872  1.00 16.62           O
ATOM   2698  CB  VAL A 344      -7.169  -1.751  28.262  1.00  9.56           C
ATOM   2699  CG1 VAL A 344      -6.537  -2.427  29.439  1.00 11.59           C
ATOM   2700  CG2 VAL A 344      -6.344  -1.992  27.048  1.00 15.07           C
ATOM   2701  N   LEU A 345     -10.048  -1.400  29.763  1.00 16.26           N
ATOM   2702  CA  LEU A 345     -10.925  -1.526  30.942  1.00 17.05           C
ATOM   2703  C   LEU A 345     -12.105  -2.415  30.592  1.00 15.69           C
ATOM   2704  O   LEU A 345     -12.294  -3.481  31.172  1.00 14.48           O
ATOM   2705  CB  LEU A 345     -11.459  -0.159  31.392  1.00 18.45           C
ATOM   2706  CG  LEU A 345     -11.962   0.060  32.830  1.00 18.68           C
ATOM   2707  CD1 LEU A 345     -13.262  -0.629  33.058  1.00 17.13           C
ATOM   2708  CD2 LEU A 345     -10.926  -0.419  33.841  1.00 21.35           C
ATOM   2709  N   LEU A 346     -12.855  -1.992  29.588  1.00 14.21           N
ATOM   2710  CA  LEU A 346     -14.109  -2.633  29.226  1.00 14.41           C
ATOM   2711  C   LEU A 346     -13.900  -4.106  28.967  1.00 15.55           C
ATOM   2712  O   LEU A 346     -14.832  -4.889  29.076  1.00 16.56           O
ATOM   2713  CB  LEU A 346     -14.688  -1.981  27.970  1.00 14.22           C
ATOM   2714  CG  LEU A 346     -16.004  -1.213  27.991  1.00 11.29           C
ATOM   2715  CD1 LEU A 346     -16.066  -0.240  29.135  1.00 13.74           C
ATOM   2716  CD2 LEU A 346     -16.105  -0.487  26.692  1.00 11.30           C
ATOM   2717  N   LEU A 347     -12.680  -4.472  28.591  1.00 17.67           N
ATOM   2718  CA  LEU A 347     -12.325  -5.868  28.316  1.00 18.46           C
ATOM   2719  C   LEU A 347     -12.252  -6.655  29.618  1.00 18.38           C
ATOM   2720  O   LEU A 347     -12.542  -7.844  29.626  1.00 18.38           O
ATOM   2721  CB  LEU A 347     -10.977  -5.941  27.598  1.00 18.93           C
ATOM   2722  CG  LEU A 347     -10.687  -7.030  26.566  1.00 18.83           C
ATOM   2723  CD1 LEU A 347     -11.567  -8.246  26.778  1.00 17.43           C
ATOM   2724  CD2 LEU A 347     -10.883  -6.449  25.188  1.00 17.31           C
ATOM   2725  N   ARG A 348     -11.908  -5.966  30.710  1.00 18.37           N
ATOM   2726  CA  ARG A 348     -12.082  -6.454  32.088  1.00 19.23           C
ATOM   2727  C   ARG A 348     -13.543  -6.617  32.563  1.00 19.03           C
ATOM   2728  O   ARG A 348     -13.821  -7.463  33.423  1.00 21.17           O
ATOM   2729  CB  ARG A 348     -11.365  -5.526  33.060  1.00 17.92           C
ATOM   2730  CG  ARG A 348      -9.954  -5.943  33.395  1.00 21.29           C
ATOM   2731  CD  ARG A 348      -9.199  -4.790  34.054  1.00 22.78           C
ATOM   2732  NE  ARG A 348      -7.997  -5.281  34.717  1.00 25.21           N
ATOM   2733  CZ  ARG A 348      -7.661  -5.008  35.977  1.00 26.74           C
ATOM   2734  NH1 ARG A 348      -8.245  -4.007  36.636  1.00 24.24           N1+
ATOM   2735  NH2 ARG A 348      -6.631  -5.644  36.530  1.00 25.87           N
ATOM   2736  N   LEU A 349     -14.437  -5.728  32.119  1.00 15.77           N
ATOM   2737  CA  LEU A 349     -15.868  -5.894  32.346  1.00 11.96           C
ATOM   2738  C   LEU A 349     -16.417  -7.004  31.453  1.00 14.75           C
ATOM   2739  O   LEU A 349     -17.446  -7.619  31.748  1.00 16.02           O
ATOM   2740  CB  LEU A 349     -16.599  -4.603  32.054  1.00  6.86           C
ATOM   2741  CG  LEU A 349     -16.030  -3.328  32.671  1.00 12.17           C
ATOM   2742  CD1 LEU A 349     -17.117  -2.246  32.667  1.00 14.15           C
ATOM   2743  CD2 LEU A 349     -15.553  -3.568  34.090  1.00 10.52           C
ATOM   2744  N   ALA A 350     -15.667  -7.344  30.416  1.00 15.21           N
ATOM   2745  CA  ALA A 350     -16.061  -8.424  29.539  1.00 16.39           C
ATOM   2746  C   ALA A 350     -15.410  -9.730  29.963  1.00 17.56           C
ATOM   2747  O   ALA A 350     -15.616 -10.767  29.332  1.00 17.63           O
ATOM   2748  CB  ALA A 350     -15.695  -8.084  28.116  1.00 17.35           C
ATOM   2749  N   LYS A 351     -14.654  -9.688  31.057  1.00 19.16           N
ATOM   2750  CA  LYS A 351     -14.267 -10.913  31.747  1.00 20.56           C
ATOM   2751  C   LYS A 351     -14.763 -11.048  33.166  1.00 20.35           C
ATOM   2752  O   LYS A 351     -14.523 -12.083  33.770  1.00 20.19           O
ATOM   2753  CB  LYS A 351     -12.756 -11.122  31.726  1.00 23.48           C
ATOM   2754  CG  LYS A 351     -12.300 -12.152  30.682  1.00 29.67           C
ATOM   2755  CD  LYS A 351     -12.868 -11.815  29.274  1.00 33.17           C
ATOM   2756  CE  LYS A 351     -11.936 -12.235  28.116  1.00 33.44           C
ATOM   2757  NZ  LYS A 351     -12.007 -11.236  26.983  1.00 31.75           N1+
ATOM   2758  N   THR A 352     -15.548 -10.079  33.651  1.00 22.45           N
ATOM   2759  CA  THR A 352     -16.326 -10.254  34.892  1.00 25.02           C
ATOM   2760  C   THR A 352     -17.850 -10.434  34.671  1.00 27.15           C
ATOM   2761  O   THR A 352     -18.596 -10.768  35.590  1.00 28.15           O
ATOM   2762  CB  THR A 352     -16.105  -9.093  35.854  1.00 25.24           C
ATOM   2763  CG2 THR A 352     -16.302  -9.557  37.305  1.00 25.36           C
ATOM   2764  OG1 THR A 352     -14.772  -8.583  35.690  1.00 27.53           O
ATOM   2765  N   TYR A 353     -18.314 -10.227  33.443  1.00 28.56           N
ATOM   2766  CA  TYR A 353     -19.436 -11.009  32.951  1.00 27.35           C
```

ATOM	2767	C	TYR	A	353	-18.988	-12.487	32.781	1.00	28.05	C
ATOM	2768	O	TYR	A	353	-19.141	-13.277	33.718	1.00	29.94	O
ATOM	2769	CB	TYR	A	353	-19.942	-10.427	31.629	1.00	25.44	C
ATOM	2770	CG	TYR	A	353	-21.447	-10.511	31.448	1.00	24.45	C
ATOM	2771	CD1	TYR	A	353	-22.327	-10.076	32.455	1.00	21.15	C
ATOM	2772	CD2	TYR	A	353	-21.991	-11.122	30.316	1.00	22.41	C
ATOM	2773	CE1	TYR	A	353	-23.705	-10.270	32.338	1.00	18.94	C
ATOM	2774	CE2	TYR	A	353	-23.364	-11.318	30.195	1.00	19.82	C
ATOM	2775	CZ	TYR	A	353	-24.208	-10.906	31.206	1.00	19.86	C
ATOM	2776	OH	TYR	A	353	-25.525	-11.262	31.124	1.00	18.86	O
ATOM	2777	N	GLU	A	354	-18.244	-12.794	31.714	1.00	25.58	N
ATOM	2778	CA	GLU	A	354	-17.941	-14.192	31.332	1.00	24.34	C
ATOM	2779	C	GLU	A	354	-17.618	-15.178	32.445	1.00	23.15	C
ATOM	2780	O	GLU	A	354	-17.902	-16.361	32.315	1.00	21.79	O
ATOM	2781	CB	GLU	A	354	-16.774	-14.261	30.346	1.00	25.14	C
ATOM	2782	CG	GLU	A	354	-16.485	-15.694	29.867	1.00	24.30	C
ATOM	2783	CD	GLU	A	354	-15.011	-16.006	29.672	1.00	23.93	C
ATOM	2784	OE1	GLU	A	354	-14.145	-15.338	30.288	1.00	22.57	O
ATOM	2785	OE2	GLU	A	354	-14.727	-16.952	28.905	1.00	22.72	O1-
ATOM	2786	N	THR	A	355	-16.746	-14.776	33.353	1.00	22.89	N
ATOM	2787	CA	THR	A	355	-16.424	-15.652	34.455	1.00	21.39	C
ATOM	2788	C	THR	A	355	-17.689	-15.821	35.259	1.00	23.76	C
ATOM	2789	O	THR	A	355	-18.292	-16.908	35.252	1.00	24.10	O
ATOM	2790	CB	THR	A	355	-15.414	-15.041	35.375	1.00	22.64	C
ATOM	2791	CG2	THR	A	355	-14.930	-16.101	36.358	1.00	23.56	C
ATOM	2792	OG1	THR	A	355	-14.333	-14.484	34.609	1.00	23.91	O
ATOM	2793	N	THR	A	356	-18.140	-14.704	35.852	1.00	23.40	N
ATOM	2794	CA	THR	A	356	-19.244	-14.695	36.812	1.00	23.16	C
ATOM	2795	C	THR	A	356	-20.470	-15.421	36.293	1.00	22.64	C
ATOM	2796	O	THR	A	356	-20.910	-16.389	36.901	1.00	24.94	O
ATOM	2797	CB	THR	A	356	-19.636	-13.257	37.216	1.00	24.04	C
ATOM	2798	CG2	THR	A	356	-21.004	-13.225	37.905	1.00	21.12	C
ATOM	2799	OG1	THR	A	356	-18.647	-12.740	38.121	1.00	28.79	O
ATOM	2800	N	LEU	A	357	-20.936	-15.060	35.105	1.00	19.87	N
ATOM	2801	CA	LEU	A	357	-22.092	-15.724	34.542	1.00	16.07	C
ATOM	2802	C	LEU	A	357	-21.893	-17.215	34.408	1.00	16.68	C
ATOM	2803	O	LEU	A	357	-22.844	-17.937	34.102	1.00	16.65	O
ATOM	2804	CB	LEU	A	357	-22.428	-15.140	33.189	1.00	13.80	C
ATOM	2805	CG	LEU	A	357	-23.662	-14.274	33.305	1.00	14.12	C
ATOM	2806	CD1	LEU	A	357	-23.278	-12.964	33.979	1.00	11.35	C
ATOM	2807	CD2	LEU	A	357	-24.275	-14.076	31.936	1.00	9.43	C
ATOM	2808	N	GLU	A	358	-20.639	-17.653	34.510	1.00	18.54	N
ATOM	2809	CA	GLU	A	358	-20.306	-19.077	34.509	1.00	22.18	C
ATOM	2810	C	GLU	A	358	-20.175	-19.565	35.932	1.00	23.29	C
ATOM	2811	O	GLU	A	358	-20.495	-20.722	36.210	1.00	25.03	O
ATOM	2812	CB	GLU	A	358	-19.004	-19.353	33.731	1.00	25.37	C
ATOM	2813	CG	GLU	A	358	-19.208	-20.016	32.329	1.00	29.81	C
ATOM	2814	CD	GLU	A	358	-19.717	-19.054	31.220	1.00	30.46	C
ATOM	2815	OE1	GLU	A	358	-18.859	-18.380	30.586	1.00	30.40	O
ATOM	2816	OE2	GLU	A	358	-20.946	-19.046	30.925	1.00	25.21	O1-
ATOM	2817	N	LYS	A	359	-19.818	-18.642	36.833	1.00	22.28	N
ATOM	2818	CA	LYS	A	359	-19.902	-18.823	38.294	1.00	21.07	C
ATOM	2819	C	LYS	A	359	-21.329	-19.097	38.762	1.00	21.42	C
ATOM	2820	O	LYS	A	359	-21.543	-19.518	39.903	1.00	21.98	O
ATOM	2821	CB	LYS	A	359	-19.409	-17.562	39.021	1.00	20.11	C
ATOM	2822	CG	LYS	A	359	-18.134	-17.700	39.848	1.00	22.37	C
ATOM	2823	CD	LYS	A	359	-17.703	-19.159	40.055	1.00	24.00	C
ATOM	2824	CE	LYS	A	359	-16.830	-19.349	41.307	1.00	24.54	C
ATOM	2825	NZ	LYS	A	359	-17.624	-19.380	42.606	1.00	21.49	N1+
ATOM	2826	N	CYS	A	360	-22.304	-18.705	37.946	1.00	22.87	N
ATOM	2827	CA	CYS	A	360	-23.709	-18.847	38.318	1.00	24.14	C
ATOM	2828	C	CYS	A	360	-24.710	-18.775	37.158	1.00	23.71	C
ATOM	2829	O	CYS	A	360	-25.613	-17.929	37.133	1.00	24.52	O
ATOM	2830	CB	CYS	A	360	-24.057	-17.808	39.374	1.00	24.78	C
ATOM	2831	SG	CYS	A	360	-22.748	-16.563	39.608	1.00	26.85	S
ATOM	2832	N	CYS	A	361	-24.405	-19.527	36.113	1.00	22.64	N
ATOM	2833	CA	CYS	A	361	-25.419	-20.138	35.292	1.00	22.37	C
ATOM	2834	C	CYS	A	361	-25.485	-21.605	35.702	1.00	23.29	C
ATOM	2835	O	CYS	A	361	-26.339	-22.359	35.231	1.00	23.57	O
ATOM	2836	CB	CYS	A	361	-25.025	-20.055	33.828	1.00	23.14	C
ATOM	2837	SG	CYS	A	361	-26.368	-19.332	32.880	1.00	26.83	S

ATOM	2838	N	ALA	A	362	-24.508	-22.030	36.497	1.00	23.59	N
ATOM	2839	CA	ALA	A	362	-24.434	-23.405	36.944	1.00	25.12	C
ATOM	2840	C	ALA	A	362	-25.368	-23.670	38.107	1.00	25.80	C
ATOM	2841	O	ALA	A	362	-26.545	-23.951	37.901	1.00	26.67	O
ATOM	2842	CB	ALA	A	362	-22.999	-23.748	37.342	1.00	27.30	C
ATOM	2843	N	ALA	A	363	-24.858	-23.502	39.326	1.00	23.91	N
ATOM	2844	CA	ALA	A	363	-25.445	-24.155	40.488	1.00	26.21	C
ATOM	2845	C	ALA	A	363	-26.359	-23.304	41.386	1.00	26.42	C
ATOM	2846	O	ALA	A	363	-26.234	-22.069	41.407	1.00	24.96	O
ATOM	2847	CB	ALA	A	363	-24.331	-24.804	41.343	1.00	25.36	C
ATOM	2848	N	ALA	A	364	-27.415	-23.948	41.912	1.00	28.14	N
ATOM	2849	CA	ALA	A	364	-28.010	-23.673	43.236	1.00	31.01	C
ATOM	2850	C	ALA	A	364	-28.676	-22.323	43.366	1.00	32.95	C
ATOM	2851	O	ALA	A	364	-28.424	-21.582	44.343	1.00	33.01	O
ATOM	2852	CB	ALA	A	364	-26.941	-23.792	44.302	1.00	32.09	C
ATOM	2853	N	ASP	A	365	-29.630	-22.080	42.470	1.00	33.49	N
ATOM	2854	CA	ASP	A	365	-30.065	-20.719	42.092	1.00	32.31	C
ATOM	2855	C	ASP	A	365	-30.308	-19.789	43.290	1.00	34.06	C
ATOM	2856	O	ASP	A	365	-30.266	-20.216	44.442	1.00	35.19	O
ATOM	2857	CB	ASP	A	365	-31.305	-20.839	41.165	1.00	30.17	C
ATOM	2858	CG	ASP	A	365	-32.215	-21.973	41.556	1.00	27.56	C
ATOM	2859	OD1	ASP	A	365	-32.811	-21.879	42.644	1.00	25.00	O
ATOM	2860	OD2	ASP	A	365	-32.379	-22.899	40.741	1.00	24.35	O1-
ATOM	2861	N	PRO	A	366	-30.401	-18.469	43.078	1.00	34.76	N
ATOM	2862	CA	PRO	A	366	-31.123	-17.957	41.922	1.00	34.85	C
ATOM	2863	C	PRO	A	366	-30.281	-16.923	41.199	1.00	35.81	C
ATOM	2864	O	PRO	A	366	-29.071	-17.095	41.054	1.00	36.75	O
ATOM	2865	CB	PRO	A	366	-32.402	-17.379	42.539	1.00	33.58	C
ATOM	2866	CG	PRO	A	366	-31.998	-16.969	43.986	1.00	34.17	C
ATOM	2867	CD	PRO	A	366	-30.585	-17.580	44.243	1.00	35.22	C
ATOM	2868	N	HIS	A	367	-30.879	-15.760	40.952	1.00	35.99	N
ATOM	2869	CA	HIS	A	367	-30.318	-14.767	40.058	1.00	32.68	C
ATOM	2870	C	HIS	A	367	-29.370	-13.770	40.677	1.00	32.19	C
ATOM	2871	O	HIS	A	367	-28.438	-13.300	40.019	1.00	32.60	O
ATOM	2872	CB	HIS	A	367	-31.447	-14.017	39.383	1.00	30.02	C
ATOM	2873	CG	HIS	A	367	-31.860	-14.638	38.108	1.00	25.99	C
ATOM	2874	CD2	HIS	A	367	-32.718	-14.223	37.152	1.00	26.12	C
ATOM	2875	ND1	HIS	A	367	-31.248	-15.773	37.620	1.00	25.25	N
ATOM	2876	CE1	HIS	A	367	-31.706	-16.026	36.408	1.00	27.08	C
ATOM	2877	NE2	HIS	A	367	-32.598	-15.099	36.097	1.00	29.00	N
ATOM	2878	N	GLU	A	368	-29.538	-13.526	41.962	1.00	30.39	N
ATOM	2879	CA	GLU	A	368	-28.843	-12.426	42.577	1.00	29.24	C
ATOM	2880	C	GLU	A	368	-27.331	-12.453	42.287	1.00	28.13	C
ATOM	2881	O	GLU	A	368	-26.729	-11.384	42.163	1.00	26.88	O
ATOM	2882	CB	GLU	A	368	-29.136	-12.413	44.090	1.00	29.55	C
ATOM	2883	CG	GLU	A	368	-30.634	-12.566	44.422	1.00	25.73	C
ATOM	2884	CD	GLU	A	368	-31.265	-11.300	44.974	1.00	24.58	C
ATOM	2885	OE1	GLU	A	368	-30.521	-10.338	45.266	1.00	25.16	O
ATOM	2886	OE2	GLU	A	368	-32.498	-11.296	45.176	1.00	21.06	O1-
ATOM	2887	N	CYS	A	369	-26.758	-13.638	42.021	1.00	26.26	N
ATOM	2888	CA	CYS	A	369	-25.313	-13.757	41.759	1.00	24.90	C
ATOM	2889	C	CYS	A	369	-24.874	-12.701	40.778	1.00	25.23	C
ATOM	2890	O	CYS	A	369	-23.911	-12.003	41.032	1.00	25.76	O
ATOM	2891	CB	CYS	A	369	-24.941	-15.093	41.136	1.00	25.85	C
ATOM	2892	SG	CYS	A	369	-23.840	-14.850	39.683	1.00	28.33	S
ATOM	2893	N	TYR	A	370	-25.488	-12.721	39.593	1.00	24.71	N
ATOM	2894	CA	TYR	A	370	-25.245	-11.722	38.563	1.00	23.51	C
ATOM	2895	C	TYR	A	370	-26.029	-10.420	38.757	1.00	24.62	C
ATOM	2896	O	TYR	A	370	-25.953	-9.523	37.906	1.00	23.98	O
ATOM	2897	CB	TYR	A	370	-25.537	-12.308	37.178	1.00	24.52	C
ATOM	2898	CG	TYR	A	370	-26.989	-12.560	36.798	1.00	22.90	C
ATOM	2899	CD1	TYR	A	370	-27.771	-11.529	36.277	1.00	24.94	C
ATOM	2900	CD2	TYR	A	370	-27.445	-13.861	36.605	1.00	22.80	C
ATOM	2901	CE1	TYR	A	370	-28.942	-11.789	35.548	1.00	23.26	C
ATOM	2902	CE2	TYR	A	370	-28.605	-14.126	35.880	1.00	21.68	C
ATOM	2903	CZ	TYR	A	370	-29.347	-13.085	35.357	1.00	22.31	C
ATOM	2904	OH	TYR	A	370	-30.530	-13.345	34.705	1.00	19.63	O
ATOM	2905	N	ALA	A	371	-26.806	-10.341	39.843	1.00	22.53	N
ATOM	2906	CA	ALA	A	371	-27.517	-9.121	40.212	1.00	20.95	C
ATOM	2907	C	ALA	A	371	-26.534	-8.002	40.483	1.00	21.22	C
ATOM	2908	O	ALA	A	371	-25.767	-8.032	41.452	1.00	18.20	O

ATOM	2909	CB	ALA	A	371	-28.392	-9.347	41.437	1.00	20.29	C
ATOM	2910	N	LYS	A	372	-26.618	-6.979	39.644	1.00	23.07	N
ATOM	2911	CA	LYS	A	372	-25.638	-5.897	39.648	1.00	26.70	C
ATOM	2912	C	LYS	A	372	-24.221	-6.384	39.978	1.00	28.46	C
ATOM	2913	O	LYS	A	372	-23.624	-5.993	41.000	1.00	26.84	O
ATOM	2914	CB	LYS	A	372	-26.041	-4.787	40.619	1.00	25.91	C
ATOM	2915	CG	LYS	A	372	-27.377	-4.140	40.291	1.00	25.48	C
ATOM	2916	CD	LYS	A	372	-27.886	-3.362	41.489	1.00	21.87	C
ATOM	2917	CE	LYS	A	372	-26.866	-2.361	41.922	1.00	18.47	C
ATOM	2918	NZ	LYS	A	372	-26.407	-1.618	40.728	1.00	20.65	N1+
ATOM	2919	N	VAL	A	373	-23.710	-7.262	39.109	1.00	27.77	N
ATOM	2920	CA	VAL	A	373	-22.311	-7.224	38.739	1.00	24.92	C
ATOM	2921	C	VAL	A	373	-21.958	-5.773	38.482	1.00	24.34	C
ATOM	2922	O	VAL	A	373	-20.982	-5.276	39.007	1.00	23.37	O
ATOM	2923	CB	VAL	A	373	-22.036	-8.008	37.459	1.00	25.87	C
ATOM	2924	CG1	VAL	A	373	-21.594	-9.423	37.811	1.00	27.81	C
ATOM	2925	CG2	VAL	A	373	-23.271	-8.017	36.564	1.00	25.54	C
ATOM	2926	N	PHE	A	374	-22.878	-5.047	37.856	1.00	25.35	N
ATOM	2927	CA	PHE	A	374	-22.738	-3.597	37.666	1.00	25.95	C
ATOM	2928	C	PHE	A	374	-22.261	-2.928	38.950	1.00	26.20	C
ATOM	2929	O	PHE	A	374	-21.593	-1.880	38.921	1.00	21.77	O
ATOM	2930	CB	PHE	A	374	-24.083	-2.995	37.222	1.00	25.29	C
ATOM	2931	CG	PHE	A	374	-24.338	-3.095	35.744	1.00	24.61	C
ATOM	2932	CD1	PHE	A	374	-23.850	-4.157	35.010	1.00	25.83	C
ATOM	2933	CD2	PHE	A	374	-25.029	-2.096	35.083	1.00	27.32	C
ATOM	2934	CE1	PHE	A	374	-24.043	-4.218	33.648	1.00	27.69	C
ATOM	2935	CE2	PHE	A	374	-25.224	-2.145	33.714	1.00	26.77	C
ATOM	2936	CZ	PHE	A	374	-24.731	-3.208	32.999	1.00	27.96	C
ATOM	2937	N	ASP	A	375	-22.562	-3.601	40.064	1.00	29.26	N
ATOM	2938	CA	ASP	A	375	-22.212	-3.148	41.409	1.00	31.59	C
ATOM	2939	C	ASP	A	375	-21.374	-4.155	42.225	1.00	29.82	C
ATOM	2940	O	ASP	A	375	-21.614	-4.358	43.415	1.00	29.59	O
ATOM	2941	CB	ASP	A	375	-23.477	-2.747	42.176	1.00	31.39	C
ATOM	2942	CG	ASP	A	375	-23.954	-1.340	41.817	1.00	32.35	C
ATOM	2943	OD1	ASP	A	375	-23.865	-0.935	40.627	1.00	30.78	O
ATOM	2944	OD2	ASP	A	375	-24.511	-0.658	42.715	1.00	36.30	O1-
ATOM	2945	N	GLU	A	376	-20.457	-4.836	41.539	1.00	28.50	N
ATOM	2946	CA	GLU	A	376	-19.115	-5.035	42.053	1.00	26.94	C
ATOM	2947	C	GLU	A	376	-18.043	-5.009	40.962	1.00	26.28	C
ATOM	2948	O	GLU	A	376	-16.887	-5.333	41.245	1.00	28.07	O
ATOM	2949	CB	GLU	A	376	-19.002	-6.334	42.848	1.00	29.11	C
ATOM	2950	CG	GLU	A	376	-19.509	-7.591	42.165	1.00	29.86	C
ATOM	2951	CD	GLU	A	376	-18.961	-7.787	40.769	1.00	28.06	C
ATOM	2952	OE1	GLU	A	376	-17.736	-7.935	40.608	1.00	29.85	O
ATOM	2953	OE2	GLU	A	376	-19.768	-7.812	39.828	1.00	27.54	O1-
ATOM	2954	N	PHE	A	377	-18.435	-4.748	39.709	1.00	21.99	N
ATOM	2955	CA	PHE	A	377	-17.479	-4.330	38.674	1.00	17.15	C
ATOM	2956	C	PHE	A	377	-16.712	-3.219	39.354	1.00	17.60	C
ATOM	2957	O	PHE	A	377	-15.582	-3.419	39.769	1.00	18.80	O
ATOM	2958	CB	PHE	A	377	-18.158	-3.679	37.448	1.00	14.09	C
ATOM	2959	CG	PHE	A	377	-18.727	-4.640	36.433	1.00	5.40	C
ATOM	2960	CD1	PHE	A	377	-18.294	-5.932	36.335	1.00	3.20	C
ATOM	2961	CD2	PHE	A	377	-19.749	-4.233	35.616	1.00	2.00	C
ATOM	2962	CE1	PHE	A	377	-18.883	-6.791	35.456	1.00	2.00	C
ATOM	2963	CE2	PHE	A	377	-20.325	-5.088	34.742	1.00	2.00	C
ATOM	2964	CZ	PHE	A	377	-19.896	-6.362	34.666	1.00	2.00	C
ATOM	2965	N	LYS	A	378	-17.484	-2.219	39.775	1.00	17.81	N
ATOM	2966	CA	LYS	A	378	-17.005	-0.869	40.084	1.00	17.26	C
ATOM	2967	C	LYS	A	378	-15.683	-0.699	40.831	1.00	15.51	C
ATOM	2968	O	LYS	A	378	-15.089	0.362	40.777	1.00	14.50	O
ATOM	2969	CB	LYS	A	378	-18.106	-0.082	40.813	1.00	17.88	C
ATOM	2970	CG	LYS	A	378	-17.952	1.453	40.765	1.00	18.67	C
ATOM	2971	CD	LYS	A	378	-19.143	2.216	41.408	1.00	16.76	C
ATOM	2972	CE	LYS	A	378	-19.247	2.011	42.921	1.00	15.54	C
ATOM	2973	NZ	LYS	A	378	-19.884	0.728	43.329	1.00	9.93	N1+
ATOM	2974	N	PRO	A	379	-15.161	-1.749	41.468	1.00	15.24	N
ATOM	2975	CA	PRO	A	379	-13.732	-1.558	41.696	1.00	17.61	C
ATOM	2976	C	PRO	A	379	-12.965	-1.316	40.391	1.00	19.67	C
ATOM	2977	O	PRO	A	379	-12.378	-0.234	40.207	1.00	20.74	O
ATOM	2978	CB	PRO	A	379	-13.302	-2.852	42.408	1.00	17.69	C
ATOM	2979	CG	PRO	A	379	-14.565	-3.698	42.537	1.00	15.99	C

ATOM	2980	CD	PRO	A	379	-15.707	-2.740	42.404	1.00	15.75	C
ATOM	2981	N	LEU	A	380	-13.078	-2.261	39.450	1.00	18.64	N
ATOM	2982	CA	LEU	A	380	-12.334	-2.227	38.183	1.00	16.78	C
ATOM	2983	C	LEU	A	380	-12.899	-1.233	37.170	1.00	16.37	C
ATOM	2984	O	LEU	A	380	-13.224	-1.618	36.052	1.00	15.89	O
ATOM	2985	CB	LEU	A	380	-12.274	-3.625	37.545	1.00	12.95	C
ATOM	2986	CG	LEU	A	380	-13.247	-4.733	37.928	1.00	11.61	C
ATOM	2987	CD1	LEU	A	380	-13.959	-5.227	36.703	1.00	10.69	C
ATOM	2988	CD2	LEU	A	380	-12.504	-5.870	38.586	1.00	10.18	C
ATOM	2989	N	VAL	A	381	-13.112	0.004	37.626	1.00	16.46	N
ATOM	2990	CA	VAL	A	381	-13.610	1.151	36.844	1.00	16.90	C
ATOM	2991	C	VAL	A	381	-13.242	2.411	37.648	1.00	17.54	C
ATOM	2992	O	VAL	A	381	-13.279	3.526	37.143	1.00	18.24	O
ATOM	2993	CB	VAL	A	381	-15.167	1.160	36.674	1.00	16.00	C
ATOM	2994	CG1	VAL	A	381	-15.587	2.360	35.866	1.00	15.15	C
ATOM	2995	CG2	VAL	A	381	-15.668	-0.096	35.995	1.00	15.14	C
ATOM	2996	N	GLU	A	382	-12.936	2.228	38.926	1.00	19.02	N
ATOM	2997	CA	GLU	A	382	-12.424	3.318	39.744	1.00	20.65	C
ATOM	2998	C	GLU	A	382	-10.913	3.520	39.525	1.00	19.24	C
ATOM	2999	O	GLU	A	382	-10.503	4.596	39.114	1.00	18.63	O
ATOM	3000	CB	GLU	A	382	-12.740	3.080	41.245	1.00	24.33	C
ATOM	3001	CG	GLU	A	382	-14.261	3.175	41.666	1.00	22.93	C
ATOM	3002	CD	GLU	A	382	-14.712	4.589	42.012	1.00	23.13	C
ATOM	3003	OE1	GLU	A	382	-13.975	5.273	42.751	1.00	26.43	O
ATOM	3004	OE2	GLU	A	382	-15.776	5.038	41.527	1.00	21.00	O1-
ATOM	3005	N	GLU	A	383	-10.115	2.456	39.602	1.00	20.27	N
ATOM	3006	CA	GLU	A	383	-8.643	2.609	39.553	1.00	20.93	C
ATOM	3007	C	GLU	A	383	-8.048	3.427	38.400	1.00	17.88	C
ATOM	3008	O	GLU	A	383	-7.189	4.253	38.640	1.00	18.41	O
ATOM	3009	CB	GLU	A	383	-7.916	1.246	39.643	1.00	24.93	C
ATOM	3010	CG	GLU	A	383	-6.405	1.261	39.272	1.00	22.82	C
ATOM	3011	CD	GLU	A	383	-5.485	1.591	40.440	1.00	22.48	C
ATOM	3012	OE1	GLU	A	383	-5.574	2.732	40.956	1.00	21.74	O
ATOM	3013	OE2	GLU	A	383	-4.621	0.737	40.781	1.00	19.05	O1-
ATOM	3014	N	PRO	A	384	-8.487	3.215	37.153	1.00	14.01	N
ATOM	3015	CA	PRO	A	384	-8.206	4.228	36.135	1.00	15.62	C
ATOM	3016	C	PRO	A	384	-8.687	5.643	36.491	1.00	18.77	C
ATOM	3017	O	PRO	A	384	-7.860	6.487	36.866	1.00	20.60	O
ATOM	3018	CB	PRO	A	384	-8.890	3.683	34.890	1.00	14.10	C
ATOM	3019	CG	PRO	A	384	-9.622	2.425	35.353	1.00	14.48	C
ATOM	3020	CD	PRO	A	384	-8.870	1.952	36.529	1.00	13.10	C
ATOM	3021	N	GLN	A	385	-10.005	5.890	36.426	1.00	19.75	N
ATOM	3022	CA	GLN	A	385	-10.623	7.176	36.806	1.00	19.44	C
ATOM	3023	C	GLN	A	385	-9.841	7.869	37.909	1.00	19.08	C
ATOM	3024	O	GLN	A	385	-9.943	9.075	38.105	1.00	19.58	O
ATOM	3025	CB	GLN	A	385	-12.068	6.967	37.300	1.00	18.93	C
ATOM	3026	CG	GLN	A	385	-13.135	7.054	36.222	1.00	22.91	C
ATOM	3027	CD	GLN	A	385	-14.452	7.633	36.729	1.00	27.37	C
ATOM	3028	NE2	GLN	A	385	-14.359	8.627	37.618	1.00	29.27	N
ATOM	3029	OE1	GLN	A	385	-15.538	7.269	36.248	1.00	23.96	O
ATOM	3030	N	ASN	A	386	-9.198	7.062	38.735	1.00	19.76	N
ATOM	3031	CA	ASN	A	386	-8.384	7.586	39.800	1.00	22.19	C
ATOM	3032	C	ASN	A	386	-6.935	7.815	39.379	1.00	20.96	C
ATOM	3033	O	ASN	A	386	-6.552	8.967	39.235	1.00	20.97	O
ATOM	3034	CB	ASN	A	386	-8.524	6.695	41.050	1.00	23.37	C
ATOM	3035	CG	ASN	A	386	-9.864	6.933	41.789	1.00	24.44	C
ATOM	3036	ND2	ASN	A	386	-10.081	8.176	42.227	1.00	19.73	N
ATOM	3037	OD1	ASN	A	386	-10.734	6.045	41.845	1.00	22.44	O
ATOM	3038	N	LEU	A	387	-6.223	6.749	38.991	1.00	20.99	N
ATOM	3039	CA	LEU	A	387	-4.777	6.761	38.639	1.00	19.03	C
ATOM	3040	C	LEU	A	387	-4.344	7.967	37.800	1.00	19.94	C
ATOM	3041	O	LEU	A	387	-3.326	8.605	38.088	1.00	15.85	O
ATOM	3042	CB	LEU	A	387	-4.387	5.459	37.888	1.00	16.60	C
ATOM	3043	CG	LEU	A	387	-2.952	5.202	37.368	1.00	14.35	C
ATOM	3044	CD1	LEU	A	387	-2.328	3.996	38.035	1.00	8.49	C
ATOM	3045	CD2	LEU	A	387	-2.974	4.987	35.868	1.00	12.25	C
ATOM	3046	N	ILE	A	388	-5.183	8.336	36.838	1.00	21.75	N
ATOM	3047	CA	ILE	A	388	-4.984	9.544	36.034	1.00	24.55	C
ATOM	3048	C	ILE	A	388	-5.094	10.847	36.880	1.00	26.81	C
ATOM	3049	O	ILE	A	388	-5.268	11.943	36.341	1.00	26.63	O
ATOM	3050	CB	ILE	A	388	-6.000	9.592	34.822	1.00	22.71	C

ATOM	3051	CG1	ILE	A	388	-6.237	8.196	34.243	1.00	18.19	C
ATOM	3052	CG2	ILE	A	388	-5.417	10.407	33.692	1.00	22.80	C
ATOM	3053	CD1	ILE	A	388	-4.998	7.574	33.621	1.00	16.41	C
ATOM	3054	N	LYS	A	389	-5.028	10.699	38.205	1.00	29.41	N
ATOM	3055	CA	LYS	A	389	-4.770	11.788	39.136	1.00	30.13	C
ATOM	3056	C	LYS	A	389	-3.251	11.818	39.376	1.00	30.26	C
ATOM	3057	O	LYS	A	389	-2.558	12.722	38.899	1.00	30.98	O
ATOM	3058	CB	LYS	A	389	-5.517	11.556	40.468	1.00	33.95	C
ATOM	3059	CG	LYS	A	389	-5.040	12.458	41.657	1.00	35.82	C
ATOM	3060	CD	LYS	A	389	-5.674	12.085	43.010	1.00	34.70	C
ATOM	3061	CE	LYS	A	389	-6.883	12.973	43.356	1.00	35.61	C
ATOM	3062	NZ	LYS	A	389	-8.212	12.441	42.868	1.00	33.54	N1+
ATOM	3063	N	GLN	A	390	-2.709	10.787	40.017	1.00	29.25	N
ATOM	3064	CA	GLN	A	390	-1.259	10.707	40.162	1.00	28.68	C
ATOM	3065	C	GLN	A	390	-0.642	10.117	38.874	1.00	27.03	C
ATOM	3066	O	GLN	A	390	0.333	9.360	38.911	1.00	26.91	O
ATOM	3067	CB	GLN	A	390	-0.882	9.887	41.406	1.00	29.73	C
ATOM	3068	CG	GLN	A	390	0.416	10.334	42.107	1.00	28.58	C
ATOM	3069	CD	GLN	A	390	0.193	11.454	43.108	1.00	29.76	C
ATOM	3070	NE2	GLN	A	390	-0.114	11.089	44.353	1.00	30.78	N
ATOM	3071	OE1	GLN	A	390	0.315	12.635	42.775	1.00	30.93	O
ATOM	3072	N	ASN	A	391	-1.358	10.288	37.772	1.00	24.04	N
ATOM	3073	CA	ASN	A	391	-0.718	10.582	36.515	1.00	22.15	C
ATOM	3074	C	ASN	A	391	-0.857	12.091	36.495	1.00	18.92	C
ATOM	3075	O	ASN	A	391	-0.065	12.773	37.132	1.00	18.87	O
ATOM	3076	CB	ASN	A	391	-1.487	9.959	35.334	1.00	28.55	C
ATOM	3077	CG	ASN	A	391	-0.680	8.877	34.571	1.00	33.87	C
ATOM	3078	ND2	ASN	A	391	0.535	8.555	35.050	1.00	36.03	N
ATOM	3079	OD1	ASN	A	391	-1.189	8.289	33.605	1.00	33.57	O
ATOM	3080	N	CYS	A	392	-2.048	12.556	36.135	1.00	16.20	N
ATOM	3081	CA	CYS	A	392	-2.222	13.923	35.670	1.00	13.67	C
ATOM	3082	C	CYS	A	392	-2.326	14.985	36.745	1.00	13.13	C
ATOM	3083	O	CYS	A	392	-2.971	16.008	36.549	1.00	15.19	O
ATOM	3084	CB	CYS	A	392	-3.420	14.004	34.730	1.00	11.64	C
ATOM	3085	SG	CYS	A	392	-3.165	13.048	33.210	1.00	12.44	S
ATOM	3086	N	GLU	A	393	-1.503	14.864	37.768	1.00	14.75	N
ATOM	3087	CA	GLU	A	393	-1.268	16.000	38.653	1.00	19.49	C
ATOM	3088	C	GLU	A	393	0.227	16.228	38.975	1.00	20.11	C
ATOM	3089	O	GLU	A	393	0.754	17.335	38.787	1.00	18.94	O
ATOM	3090	CB	GLU	A	393	-2.081	15.840	39.940	1.00	20.40	C
ATOM	3091	CG	GLU	A	393	-2.403	17.152	40.613	1.00	20.82	C
ATOM	3092	CD	GLU	A	393	-3.414	16.997	41.713	1.00	22.54	C
ATOM	3093	OE1	GLU	A	393	-3.048	16.456	42.785	1.00	24.39	O
ATOM	3094	OE2	GLU	A	393	-4.574	17.418	41.505	1.00	24.30	O1-
ATOM	3095	N	LEU	A	394	0.908	15.165	39.396	1.00	19.67	N
ATOM	3096	CA	LEU	A	394	2.353	15.160	39.518	1.00	21.83	C
ATOM	3097	C	LEU	A	394	2.947	15.795	38.259	1.00	24.26	C
ATOM	3098	O	LEU	A	394	3.959	16.503	38.286	1.00	26.00	O
ATOM	3099	CB	LEU	A	394	2.839	13.722	39.646	1.00	21.88	C
ATOM	3100	CG	LEU	A	394	4.062	13.487	40.531	1.00	23.92	C
ATOM	3101	CD1	LEU	A	394	5.242	14.340	40.026	1.00	25.67	C
ATOM	3102	CD2	LEU	A	394	3.724	13.789	41.997	1.00	21.11	C
ATOM	3103	N	PHE	A	395	2.277	15.598	37.142	1.00	25.37	N
ATOM	3104	CA	PHE	A	395	2.708	16.304	35.965	1.00	25.77	C
ATOM	3105	C	PHE	A	395	2.196	17.746	35.907	1.00	26.16	C
ATOM	3106	O	PHE	A	395	2.988	18.663	36.144	1.00	26.53	O
ATOM	3107	CB	PHE	A	395	2.361	15.469	34.744	1.00	25.66	C
ATOM	3108	CG	PHE	A	395	3.224	14.254	34.625	1.00	26.69	C
ATOM	3109	CD1	PHE	A	395	4.603	14.367	34.780	1.00	26.93	C
ATOM	3110	CD2	PHE	A	395	2.676	13.000	34.460	1.00	26.92	C
ATOM	3111	CE1	PHE	A	395	5.417	13.264	34.773	1.00	25.90	C
ATOM	3112	CE2	PHE	A	395	3.487	11.886	34.451	1.00	29.35	C
ATOM	3113	CZ	PHE	A	395	4.867	12.022	34.609	1.00	28.67	C
ATOM	3114	N	GLU	A	396	0.868	17.902	35.930	1.00	24.62	N
ATOM	3115	CA	GLU	A	396	0.191	19.186	35.745	1.00	22.66	C
ATOM	3116	C	GLU	A	396	0.982	20.346	36.329	1.00	24.98	C
ATOM	3117	O	GLU	A	396	1.905	20.842	35.684	1.00	25.79	O
ATOM	3118	CB	GLU	A	396	-1.209	19.129	36.363	1.00	21.41	C
ATOM	3119	CG	GLU	A	396	-2.062	20.342	36.075	1.00	18.86	C
ATOM	3120	CD	GLU	A	396	-3.511	20.135	36.448	1.00	19.66	C
ATOM	3121	OE1	GLU	A	396	-4.119	19.159	35.965	1.00	21.53	O

```
ATOM   3122  OE2 GLU A 396      -4.056  20.951  37.210  1.00 16.14           O1-
ATOM   3123  N   GLN A 397       0.686  20.717  37.577  1.00 28.23           N
ATOM   3124  CA  GLN A 397       1.460  21.738  38.288  1.00 27.32           C
ATOM   3125  C   GLN A 397       2.530  20.945  38.997  1.00 25.36           C
ATOM   3126  O   GLN A 397       2.351  20.612  40.158  1.00 22.92           O
ATOM   3127  CB  GLN A 397       0.595  22.527  39.326  1.00 28.36           C
ATOM   3128  CG  GLN A 397       1.354  23.682  40.097  1.00 26.26           C
ATOM   3129  CD  GLN A 397       0.487  24.528  41.064  1.00 25.26           C
ATOM   3130  NE2 GLN A 397      -0.812  24.290  41.070  1.00 27.07           N
ATOM   3131  OE1 GLN A 397       1.011  25.332  41.847  1.00 24.51           O
ATOM   3132  N   LEU A 398       3.461  20.405  38.210  1.00 25.94           N
ATOM   3133  CA  LEU A 398       4.677  19.792  38.751  1.00 26.57           C
ATOM   3134  C   LEU A 398       5.577  19.106  37.699  1.00 24.32           C
ATOM   3135  O   LEU A 398       6.460  18.340  38.071  1.00 22.18           O
ATOM   3136  CB  LEU A 398       4.328  18.793  39.899  1.00 29.28           C
ATOM   3137  CG  LEU A 398       4.839  18.900  41.365  1.00 29.60           C
ATOM   3138  CD1 LEU A 398       4.218  20.072  42.135  1.00 29.70           C
ATOM   3139  CD2 LEU A 398       4.513  17.604  42.113  1.00 27.39           C
ATOM   3140  N   GLY A 399       5.407  19.409  36.409  1.00 24.36           N
ATOM   3141  CA  GLY A 399       6.447  19.039  35.442  1.00 26.34           C
ATOM   3142  C   GLY A 399       6.243  18.217  34.146  1.00 25.35           C
ATOM   3143  O   GLY A 399       6.431  16.983  34.105  1.00 24.71           O
ATOM   3144  N   GLU A 400       6.272  18.954  33.041  1.00 23.31           N
ATOM   3145  CA  GLU A 400       6.309  18.381  31.700  1.00 21.85           C
ATOM   3146  C   GLU A 400       7.499  17.394  31.494  1.00 21.88           C
ATOM   3147  O   GLU A 400       7.351  16.186  31.628  1.00 20.25           O
ATOM   3148  CB  GLU A 400       6.389  19.544  30.704  1.00 18.99           C
ATOM   3149  CG  GLU A 400       5.051  20.164  30.301  1.00 18.80           C
ATOM   3150  CD  GLU A 400       4.368  21.016  31.372  1.00 15.88           C
ATOM   3151  OE1 GLU A 400       4.576  20.786  32.574  1.00 13.73           O
ATOM   3152  OE2 GLU A 400       3.524  21.862  30.999  1.00 14.44           O1-
ATOM   3153  N   TYR A 401       8.701  17.951  31.356  1.00 22.47           N
ATOM   3154  CA  TYR A 401       9.926  17.216  31.020  1.00 20.38           C
ATOM   3155  C   TYR A 401      10.935  17.239  32.181  1.00 19.52           C
ATOM   3156  O   TYR A 401      12.064  16.790  32.033  1.00 18.53           O
ATOM   3157  CB  TYR A 401      10.554  17.881  29.797  1.00 23.78           C
ATOM   3158  CG  TYR A 401      11.448  16.997  28.969  1.00 25.15           C
ATOM   3159  CD1 TYR A 401      12.748  16.752  29.363  1.00 25.90           C
ATOM   3160  CD2 TYR A 401      11.060  16.579  27.706  1.00 25.00           C
ATOM   3161  CE1 TYR A 401      13.641  16.158  28.525  1.00 27.01           C
ATOM   3162  CE2 TYR A 401      11.948  15.979  26.863  1.00 26.20           C
ATOM   3163  CZ  TYR A 401      13.250  15.790  27.270  1.00 26.58           C
ATOM   3164  OH  TYR A 401      14.209  15.388  26.375  1.00 22.64           O
ATOM   3165  N   LYS A 402      10.621  18.021  33.211  1.00 22.60           N
ATOM   3166  CA  LYS A 402      11.307  17.936  34.503  1.00 22.71           C
ATOM   3167  C   LYS A 402      10.981  16.574  35.103  1.00 24.22           C
ATOM   3168  O   LYS A 402      11.543  15.565  34.676  1.00 25.37           O
ATOM   3169  CB  LYS A 402      10.845  19.067  35.435  1.00 20.39           C
ATOM   3170  CG  LYS A 402      11.323  18.957  36.876  1.00 17.50           C
ATOM   3171  CD  LYS A 402      12.633  19.686  37.105  1.00 17.85           C
ATOM   3172  CE  LYS A 402      13.838  18.750  37.135  1.00 17.83           C
ATOM   3173  NZ  LYS A 402      15.068  19.486  37.556  1.00 19.64           N1+
ATOM   3174  N   PHE A 403       9.954  16.495  35.941  1.00 25.18           N
ATOM   3175  CA  PHE A 403       9.679  15.217  36.562  1.00 28.48           C
ATOM   3176  C   PHE A 403       8.640  14.362  35.850  1.00 30.15           C
ATOM   3177  O   PHE A 403       7.621  13.986  36.423  1.00 30.31           O
ATOM   3178  CB  PHE A 403       9.370  15.398  38.045  1.00 28.85           C
ATOM   3179  CG  PHE A 403      10.610  15.601  38.886  1.00 30.05           C
ATOM   3180  CD1 PHE A 403      11.155  16.860  39.037  1.00 28.78           C
ATOM   3181  CD2 PHE A 403      11.326  14.511  39.371  1.00 29.76           C
ATOM   3182  CE1 PHE A 403      12.393  17.026  39.632  1.00 28.52           C
ATOM   3183  CE2 PHE A 403      12.561  14.677  39.967  1.00 26.34           C
ATOM   3184  CZ  PHE A 403      13.097  15.928  40.092  1.00 26.88           C
ATOM   3185  N   GLN A 404       8.911  14.106  34.565  1.00 32.37           N
ATOM   3186  CA  GLN A 404       8.532  12.852  33.887  1.00 30.49           C
ATOM   3187  C   GLN A 404       9.506  11.781  34.350  1.00 29.27           C
ATOM   3188  O   GLN A 404       9.194  10.601  34.304  1.00 29.71           O
ATOM   3189  CB  GLN A 404       8.648  12.975  32.361  1.00 31.75           C
ATOM   3190  CG  GLN A 404      10.038  12.639  31.796  1.00 30.57           C
ATOM   3191  CD  GLN A 404      10.134  12.873  30.298  1.00 31.42           C
ATOM   3192  NE2 GLN A 404       9.715  11.891  29.507  1.00 30.06           N
```

ATOM	3193	OE1	GLN	A	404	10.548	13.942	29.858	1.00	31.82	O
ATOM	3194	N	ASN	A	405	10.695	12.196	34.786	1.00	27.19	N
ATOM	3195	CA	ASN	A	405	11.561	11.290	35.527	1.00	24.01	C
ATOM	3196	C	ASN	A	405	11.159	11.181	37.030	1.00	21.70	C
ATOM	3197	O	ASN	A	405	11.983	11.211	37.946	1.00	15.16	O
ATOM	3198	CB	ASN	A	405	13.033	11.663	35.278	1.00	22.91	C
ATOM	3199	CG	ASN	A	405	13.611	12.577	36.314	1.00	19.47	C
ATOM	3200	ND2	ASN	A	405	13.058	13.770	36.427	1.00	19.73	N
ATOM	3201	OD1	ASN	A	405	14.666	12.283	36.866	1.00	17.07	O
ATOM	3202	N	ALA	A	406	9.852	10.999	37.231	1.00	19.67	N
ATOM	3203	CA	ALA	A	406	9.294	10.467	38.458	1.00	18.79	C
ATOM	3204	C	ALA	A	406	8.941	9.011	38.194	1.00	20.96	C
ATOM	3205	O	ALA	A	406	9.168	8.141	39.044	1.00	23.15	O
ATOM	3206	CB	ALA	A	406	8.048	11.228	38.841	1.00	15.73	C
ATOM	3207	N	LEU	A	407	8.393	8.731	37.013	1.00	19.76	N
ATOM	3208	CA	LEU	A	407	7.955	7.379	36.692	1.00	16.89	C
ATOM	3209	C	LEU	A	407	9.136	6.433	36.519	1.00	17.14	C
ATOM	3210	O	LEU	A	407	8.978	5.221	36.655	1.00	15.99	O
ATOM	3211	CB	LEU	A	407	7.086	7.393	35.444	1.00	16.01	C
ATOM	3212	CG	LEU	A	407	5.659	7.835	35.763	1.00	18.48	C
ATOM	3213	CD1	LEU	A	407	4.978	8.430	34.540	1.00	20.67	C
ATOM	3214	CD2	LEU	A	407	4.871	6.641	36.285	1.00	20.49	C
ATOM	3215	N	LEU	A	408	10.317	6.998	36.250	1.00	18.02	N
ATOM	3216	CA	LEU	A	408	11.617	6.307	36.411	1.00	18.24	C
ATOM	3217	C	LEU	A	408	11.824	5.869	37.872	1.00	18.70	C
ATOM	3218	O	LEU	A	408	11.811	4.687	38.190	1.00	17.33	O
ATOM	3219	CB	LEU	A	408	12.767	7.250	36.018	1.00	18.19	C
ATOM	3220	CG	LEU	A	408	13.659	7.027	34.797	1.00	16.19	C
ATOM	3221	CD1	LEU	A	408	14.511	8.259	34.575	1.00	11.96	C
ATOM	3222	CD2	LEU	A	408	14.539	5.807	34.995	1.00	16.05	C
ATOM	3223	N	VAL	A	409	11.989	6.846	38.759	1.00	20.54	N
ATOM	3224	CA	VAL	A	409	12.091	6.603	40.193	1.00	20.69	C
ATOM	3225	C	VAL	A	409	11.118	5.538	40.642	1.00	21.47	C
ATOM	3226	O	VAL	A	409	11.487	4.672	41.418	1.00	23.58	O
ATOM	3227	CB	VAL	A	409	11.797	7.867	41.012	1.00	19.59	C
ATOM	3228	CG1	VAL	A	409	11.897	7.549	42.518	1.00	19.23	C
ATOM	3229	CG2	VAL	A	409	12.753	8.975	40.613	1.00	17.46	C
ATOM	3230	N	ARG	A	410	9.879	5.610	40.163	1.00	21.93	N
ATOM	3231	CA	ARG	A	410	8.889	4.593	40.488	1.00	21.95	C
ATOM	3232	C	ARG	A	410	9.369	3.263	39.930	1.00	21.48	C
ATOM	3233	O	ARG	A	410	9.723	2.363	40.692	1.00	24.94	O
ATOM	3234	CB	ARG	A	410	7.506	4.939	39.904	1.00	22.67	C
ATOM	3235	CG	ARG	A	410	6.846	6.205	40.445	1.00	20.28	C
ATOM	3236	CD	ARG	A	410	5.357	6.220	40.114	1.00	20.80	C
ATOM	3237	NE	ARG	A	410	4.733	7.522	40.365	1.00	20.80	N
ATOM	3238	CZ	ARG	A	410	3.487	7.841	40.011	1.00	20.59	C
ATOM	3239	NH1	ARG	A	410	2.705	6.941	39.426	1.00	21.04	N1+
ATOM	3240	NH2	ARG	A	410	3.025	9.067	40.234	1.00	20.07	N
ATOM	3241	N	TYR	A	411	9.529	3.186	38.615	1.00	18.76	N
ATOM	3242	CA	TYR	A	411	9.792	1.907	37.974	1.00	17.50	C
ATOM	3243	C	TYR	A	411	11.169	1.356	38.330	1.00	17.38	C
ATOM	3244	O	TYR	A	411	11.289	0.240	38.823	1.00	15.90	O
ATOM	3245	CB	TYR	A	411	9.606	2.024	36.453	1.00	18.41	C
ATOM	3246	CG	TYR	A	411	8.151	1.974	36.064	1.00	17.19	C
ATOM	3247	CD1	TYR	A	411	7.215	2.657	36.815	1.00	15.09	C
ATOM	3248	CD2	TYR	A	411	7.684	1.060	35.137	1.00	18.38	C
ATOM	3249	CE1	TYR	A	411	5.885	2.431	36.681	1.00	11.66	C
ATOM	3250	CE2	TYR	A	411	6.325	0.829	34.998	1.00	15.51	C
ATOM	3251	CZ	TYR	A	411	5.436	1.536	35.776	1.00	12.43	C
ATOM	3252	OH	TYR	A	411	4.079	1.476	35.573	1.00	16.59	O
ATOM	3253	N	THR	A	412	12.186	2.195	38.234	1.00	19.91	N
ATOM	3254	CA	THR	A	412	13.541	1.791	38.601	1.00	24.09	C
ATOM	3255	C	THR	A	412	13.591	1.176	39.988	1.00	23.32	C
ATOM	3256	O	THR	A	412	14.583	0.526	40.342	1.00	22.82	O
ATOM	3257	CB	THR	A	412	14.510	2.978	38.590	1.00	26.90	C
ATOM	3258	CG2	THR	A	412	15.900	2.514	38.214	1.00	29.28	C
ATOM	3259	OG1	THR	A	412	14.063	3.952	37.634	1.00	31.00	O
ATOM	3260	N	LYS	A	413	12.580	1.492	40.802	1.00	21.28	N
ATOM	3261	CA	LYS	A	413	12.512	1.006	42.170	1.00	19.27	C
ATOM	3262	C	LYS	A	413	11.796	-0.322	42.158	1.00	19.80	C
ATOM	3263	O	LYS	A	413	12.418	-1.382	42.340	1.00	20.91	O

ATOM	3264	CB	LYS	A	413	11.768	1.999	43.068	1.00	16.46	C
ATOM	3265	CG	LYS	A	413	12.670	2.713	44.084	1.00	17.32	C
ATOM	3266	CD	LYS	A	413	11.899	3.127	45.323	1.00	18.80	C
ATOM	3267	CE	LYS	A	413	11.409	4.570	45.242	1.00	21.30	C
ATOM	3268	NZ	LYS	A	413	12.515	5.551	45.015	1.00	20.06	N1+
ATOM	3269	N	LYS	A	414	10.531	-0.282	41.752	1.00	18.33	N
ATOM	3270	CA	LYS	A	414	9.678	-1.445	41.909	1.00	16.94	C
ATOM	3271	C	LYS	A	414	10.083	-2.597	41.015	1.00	17.20	C
ATOM	3272	O	LYS	A	414	9.744	-3.742	41.285	1.00	17.03	O
ATOM	3273	CB	LYS	A	414	8.192	-1.098	41.704	1.00	17.27	C
ATOM	3274	CG	LYS	A	414	7.823	-0.088	40.645	1.00	12.68	C
ATOM	3275	CD	LYS	A	414	6.341	-0.276	40.311	1.00	14.55	C
ATOM	3276	CE	LYS	A	414	5.798	0.739	39.303	1.00	14.56	C
ATOM	3277	NZ	LYS	A	414	5.337	2.037	39.917	1.00	14.02	N1+
ATOM	3278	N	VAL	A	415	10.934	-2.302	40.043	1.00	17.01	N
ATOM	3279	CA	VAL	A	415	11.369	-3.287	39.070	1.00	16.75	C
ATOM	3280	C	VAL	A	415	12.780	-2.914	38.581	1.00	21.86	C
ATOM	3281	O	VAL	A	415	12.949	-2.362	37.490	1.00	23.99	O
ATOM	3282	CB	VAL	A	415	10.373	-3.353	37.897	1.00	12.19	C
ATOM	3283	CG1	VAL	A	415	10.301	-2.032	37.199	1.00	10.88	C
ATOM	3284	CG2	VAL	A	415	10.754	-4.432	36.947	1.00	9.96	C
ATOM	3285	N	PRO	A	416	13.817	-3.194	39.401	1.00	23.73	N
ATOM	3286	CA	PRO	A	416	15.121	-2.556	39.239	1.00	23.14	C
ATOM	3287	C	PRO	A	416	16.129	-3.485	38.583	1.00	22.45	C
ATOM	3288	O	PRO	A	416	17.310	-3.467	38.920	1.00	20.59	O
ATOM	3289	CB	PRO	A	416	15.513	-2.221	40.679	1.00	26.64	C
ATOM	3290	CG	PRO	A	416	14.755	-3.262	41.552	1.00	24.34	C
ATOM	3291	CD	PRO	A	416	13.777	-3.984	40.645	1.00	25.02	C
ATOM	3292	N	GLN	A	417	15.653	-4.338	37.686	1.00	24.78	N
ATOM	3293	CA	GLN	A	417	16.554	-5.178	36.894	1.00	25.39	C
ATOM	3294	C	GLN	A	417	16.386	-5.115	35.367	1.00	25.68	C
ATOM	3295	O	GLN	A	417	17.256	-5.608	34.646	1.00	28.59	O
ATOM	3296	CB	GLN	A	417	16.476	-6.640	37.343	1.00	22.92	C
ATOM	3297	CG	GLN	A	417	17.445	-6.998	38.437	1.00	16.76	C
ATOM	3298	CD	GLN	A	417	16.729	-7.293	39.727	1.00	19.80	C
ATOM	3299	NE2	GLN	A	417	17.337	-8.118	40.561	1.00	17.72	N
ATOM	3300	OE1	GLN	A	417	15.618	-6.796	39.965	1.00	20.09	O
ATOM	3301	N	VAL	A	418	15.284	-4.562	34.862	1.00	23.83	N
ATOM	3302	CA	VAL	A	418	15.206	-4.262	33.420	1.00	25.79	C
ATOM	3303	C	VAL	A	418	16.443	-3.398	33.023	1.00	25.27	C
ATOM	3304	O	VAL	A	418	17.075	-2.784	33.886	1.00	26.72	O
ATOM	3305	CB	VAL	A	418	13.872	-3.506	33.057	1.00	24.98	C
ATOM	3306	CG1	VAL	A	418	13.516	-3.697	31.597	1.00	21.82	C
ATOM	3307	CG2	VAL	A	418	12.720	-4.010	33.907	1.00	25.57	C
ATOM	3308	N	SER	A	419	16.865	-3.451	31.761	1.00	23.89	N
ATOM	3309	CA	SER	A	419	18.115	-2.791	31.338	1.00	24.81	C
ATOM	3310	C	SER	A	419	18.045	-1.274	31.448	1.00	23.00	C
ATOM	3311	O	SER	A	419	17.072	-0.685	31.022	1.00	23.69	O
ATOM	3312	CB	SER	A	419	18.449	-3.159	29.888	1.00	25.20	C
ATOM	3313	OG	SER	A	419	18.798	-4.528	29.762	1.00	28.67	O
ATOM	3314	N	THR	A	420	19.130	-0.623	31.854	1.00	24.58	N
ATOM	3315	CA	THR	A	420	19.075	0.829	32.039	1.00	24.29	C
ATOM	3316	C	THR	A	420	18.501	1.555	30.855	1.00	24.81	C
ATOM	3317	O	THR	A	420	17.600	2.367	31.013	1.00	26.08	O
ATOM	3318	CB	THR	A	420	20.425	1.453	32.348	1.00	21.78	C
ATOM	3319	CG2	THR	A	420	20.375	2.953	32.130	1.00	21.26	C
ATOM	3320	OG1	THR	A	420	20.763	1.193	33.715	1.00	24.19	O
ATOM	3321	N	PRO	A	421	18.946	1.222	29.642	1.00	25.02	N
ATOM	3322	CA	PRO	A	421	18.193	1.830	28.545	1.00	26.98	C
ATOM	3323	C	PRO	A	421	16.704	1.458	28.629	1.00	27.18	C
ATOM	3324	O	PRO	A	421	15.850	2.349	28.768	1.00	25.10	O
ATOM	3325	CB	PRO	A	421	18.867	1.272	27.282	1.00	27.79	C
ATOM	3326	CG	PRO	A	421	19.758	0.135	27.777	1.00	29.44	C
ATOM	3327	CD	PRO	A	421	20.152	0.531	29.167	1.00	25.03	C
ATOM	3328	N	THR	A	422	16.427	0.149	28.728	1.00	26.36	N
ATOM	3329	CA	THR	A	422	15.064	-0.393	28.605	1.00	24.58	C
ATOM	3330	C	THR	A	422	14.162	0.069	29.744	1.00	24.87	C
ATOM	3331	O	THR	A	422	13.011	-0.349	29.839	1.00	27.96	O
ATOM	3332	CB	THR	A	422	15.058	-1.947	28.560	1.00	21.16	C
ATOM	3333	CG2	THR	A	422	13.682	-2.465	28.189	1.00	21.32	C
ATOM	3334	OG1	THR	A	422	15.985	-2.411	27.570	1.00	20.86	O

```
ATOM   3335  N   LEU A 423      14.745   0.778  30.703  1.00 25.19           N
ATOM   3336  CA  LEU A 423      13.976   1.651  31.558  1.00 22.20           C
ATOM   3337  C   LEU A 423      13.860   2.946  30.755  1.00 22.66           C
ATOM   3338  O   LEU A 423      12.860   3.113  30.062  1.00 22.77           O
ATOM   3339  CB  LEU A 423      14.689   1.889  32.891  1.00 23.24           C
ATOM   3340  CG  LEU A 423      14.626   0.885  34.065  1.00 22.20           C
ATOM   3341  CD1 LEU A 423      13.289   0.159  34.098  1.00 19.45           C
ATOM   3342  CD2 LEU A 423      15.788  -0.093  33.971  1.00 18.12           C
ATOM   3343  N   VAL A 424      14.969   3.678  30.592  1.00 22.40           N
ATOM   3344  CA  VAL A 424      14.941   5.007  29.944  1.00 19.48           C
ATOM   3345  C   VAL A 424      14.315   5.097  28.554  1.00 21.48           C
ATOM   3346  O   VAL A 424      14.119   6.211  28.067  1.00 23.47           O
ATOM   3347  CB  VAL A 424      16.337   5.707  29.809  1.00 17.06           C
ATOM   3348  CG1 VAL A 424      16.141   7.204  29.867  1.00  9.48           C
ATOM   3349  CG2 VAL A 424      17.336   5.264  30.890  1.00 14.26           C
ATOM   3350  N   GLU A 425      13.977   3.977  27.908  1.00 20.71           N
ATOM   3351  CA  GLU A 425      13.114   4.076  26.724  1.00 20.21           C
ATOM   3352  C   GLU A 425      11.679   4.209  27.187  1.00 21.09           C
ATOM   3353  O   GLU A 425      10.983   5.151  26.790  1.00 22.27           O
ATOM   3354  CB  GLU A 425      13.231   2.868  25.790  1.00 19.48           C
ATOM   3355  CG  GLU A 425      13.577   3.210  24.334  1.00 17.47           C
ATOM   3356  CD  GLU A 425      12.582   4.152  23.653  1.00 17.36           C
ATOM   3357  OE1 GLU A 425      12.703   5.386  23.801  1.00 16.77           O
ATOM   3358  OE2 GLU A 425      11.746   3.674  22.860  1.00 20.99           O1-
ATOM   3359  N   VAL A 426      11.277   3.332  28.108  1.00 20.45           N
ATOM   3360  CA  VAL A 426       9.940   3.379  28.710  1.00 18.52           C
ATOM   3361  C   VAL A 426       9.831   4.594  29.631  1.00 17.11           C
ATOM   3362  O   VAL A 426       9.014   5.466  29.407  1.00 19.30           O
ATOM   3363  CB  VAL A 426       9.662   2.131  29.556  1.00 17.57           C
ATOM   3364  CG1 VAL A 426       8.194   1.980  29.795  1.00 17.28           C
ATOM   3365  CG2 VAL A 426      10.227   0.911  28.878  1.00 20.59           C
ATOM   3366  N   SER A 427      10.693   4.664  30.635  1.00 14.13           N
ATOM   3367  CA  SER A 427      10.598   5.670  31.676  1.00 14.17           C
ATOM   3368  C   SER A 427      10.109   7.012  31.164  1.00 13.54           C
ATOM   3369  O   SER A 427       9.024   7.432  31.522  1.00 11.17           O
ATOM   3370  CB  SER A 427      11.950   5.834  32.356  1.00 17.92           C
ATOM   3371  OG  SER A 427      12.541   4.586  32.668  1.00 14.61           O
ATOM   3372  N   ARG A 428      10.786   7.547  30.152  1.00 15.97           N
ATOM   3373  CA  ARG A 428      10.307   8.758  29.451  1.00 19.71           C
ATOM   3374  C   ARG A 428       9.104   8.551  28.504  1.00 19.67           C
ATOM   3375  O   ARG A 428       8.208   9.395  28.438  1.00 20.46           O
ATOM   3376  CB  ARG A 428      11.447   9.463  28.679  1.00 17.27           C
ATOM   3377  CG  ARG A 428      12.600   8.580  28.234  1.00 13.84           C
ATOM   3378  CD  ARG A 428      13.345   9.187  27.075  1.00 12.03           C
ATOM   3379  NE  ARG A 428      13.832  10.531  27.369  1.00 13.38           N
ATOM   3380  CZ  ARG A 428      13.437  11.648  26.750  1.00 12.65           C
ATOM   3381  NH1 ARG A 428      12.447  11.635  25.863  1.00  7.48           N1+
ATOM   3382  NH2 ARG A 428      14.028  12.800  27.040  1.00 10.10           N
ATOM   3383  N   ASN A 429       9.094   7.458  27.750  1.00 20.12           N
ATOM   3384  CA  ASN A 429       7.972   7.177  26.876  1.00 20.34           C
ATOM   3385  C   ASN A 429       6.612   7.140  27.579  1.00 20.43           C
ATOM   3386  O   ASN A 429       5.772   7.993  27.303  1.00 23.09           O
ATOM   3387  CB  ASN A 429       8.215   5.903  26.081  1.00 19.92           C
ATOM   3388  CG  ASN A 429       8.527   6.196  24.626  1.00 23.69           C
ATOM   3389  ND2 ASN A 429       9.313   7.253  24.394  1.00 24.44           N
ATOM   3390  OD1 ASN A 429       7.988   5.552  23.716  1.00 23.46           O
ATOM   3391  N   LEU A 430       6.418   6.233  28.542  1.00 18.98           N
ATOM   3392  CA  LEU A 430       5.201   6.222  29.366  1.00 13.10           C
ATOM   3393  C   LEU A 430       5.217   7.456  30.222  1.00 13.42           C
ATOM   3394  O   LEU A 430       4.270   7.738  30.921  1.00 14.99           O
ATOM   3395  CB  LEU A 430       5.120   4.968  30.239  1.00  7.34           C
ATOM   3396  CG  LEU A 430       5.092   4.992  31.769  1.00  2.61           C
ATOM   3397  CD1 LEU A 430       4.894   3.578  32.284  1.00  3.10           C
ATOM   3398  CD2 LEU A 430       6.381   5.518  32.313  1.00  2.00           C
ATOM   3399  N   GLY A 431       6.352   8.135  30.244  1.00 16.38           N
ATOM   3400  CA  GLY A 431       6.351   9.513  30.682  1.00 17.97           C
ATOM   3401  C   GLY A 431       5.371  10.263  29.810  1.00 17.99           C
ATOM   3402  O   GLY A 431       4.290  10.586  30.285  1.00 20.37           O
ATOM   3403  N   LYS A 432       5.621  10.252  28.499  1.00 16.66           N
ATOM   3404  CA  LYS A 432       4.947  11.127  27.524  1.00 15.91           C
ATOM   3405  C   LYS A 432       3.429  11.034  27.418  1.00 15.02           C
```

ATOM	3406	O	LYS	A	432	2.850	11.519	26.453	1.00	11.29	O
ATOM	3407	CB	LYS	A	432	5.525	10.900	26.135	1.00	16.80	C
ATOM	3408	CG	LYS	A	432	7.003	11.220	25.997	1.00	18.19	C
ATOM	3409	CD	LYS	A	432	7.359	11.418	24.526	1.00	19.26	C
ATOM	3410	CE	LYS	A	432	8.406	10.405	24.082	1.00	22.95	C
ATOM	3411	NZ	LYS	A	432	8.438	10.312	22.595	1.00	27.31	N1+
ATOM	3412	N	VAL	A	433	2.802	10.333	28.356	1.00	16.94	N
ATOM	3413	CA	VAL	A	433	1.353	10.328	28.507	1.00	15.68	C
ATOM	3414	C	VAL	A	433	0.985	11.622	29.194	1.00	14.62	C
ATOM	3415	O	VAL	A	433	0.140	12.354	28.707	1.00	18.83	O
ATOM	3416	CB	VAL	A	433	0.878	9.124	29.363	1.00	16.06	C
ATOM	3417	CG1	VAL	A	433	-0.188	9.552	30.376	1.00	15.00	C
ATOM	3418	CG2	VAL	A	433	0.367	8.019	28.463	1.00	10.29	C
ATOM	3419	N	GLY	A	434	1.701	11.967	30.251	1.00	13.40	N
ATOM	3420	CA	GLY	A	434	1.392	13.190	30.969	1.00	17.13	C
ATOM	3421	C	GLY	A	434	1.177	14.348	30.013	1.00	18.55	C
ATOM	3422	O	GLY	A	434	0.108	14.946	29.981	1.00	17.84	O
ATOM	3423	N	SER	A	435	2.091	14.472	29.063	1.00	20.65	N
ATOM	3424	CA	SER	A	435	2.031	15.533	28.073	1.00	21.99	C
ATOM	3425	C	SER	A	435	0.790	15.331	27.200	1.00	22.53	C
ATOM	3426	O	SER	A	435	-0.251	15.929	27.471	1.00	22.40	O
ATOM	3427	CB	SER	A	435	3.329	15.553	27.221	1.00	25.26	C
ATOM	3428	OG	SER	A	435	4.523	15.746	28.007	1.00	22.37	O
ATOM	3429	N	LYS	A	436	0.832	14.330	26.322	1.00	23.94	N
ATOM	3430	CA	LYS	A	436	-0.157	14.181	25.250	1.00	25.08	C
ATOM	3431	C	LYS	A	436	-1.528	13.626	25.661	1.00	24.18	C
ATOM	3432	O	LYS	A	436	-2.216	13.046	24.827	1.00	26.29	O
ATOM	3433	CB	LYS	A	436	0.410	13.295	24.120	1.00	27.26	C
ATOM	3434	CG	LYS	A	436	-0.211	13.540	22.713	1.00	29.66	C
ATOM	3435	CD	LYS	A	436	-0.836	12.276	22.091	1.00	28.08	C
ATOM	3436	CE	LYS	A	436	-2.362	12.442	21.910	1.00	28.14	C
ATOM	3437	NZ	LYS	A	436	-3.079	11.155	21.561	1.00	26.79	N1+
ATOM	3438	N	CYS	A	437	-1.959	13.825	26.903	1.00	22.66	N
ATOM	3439	CA	CYS	A	437	-3.299	13.371	27.287	1.00	22.80	C
ATOM	3440	C	CYS	A	437	-3.940	14.098	28.461	1.00	22.95	C
ATOM	3441	O	CYS	A	437	-5.118	14.394	28.420	1.00	23.92	O
ATOM	3442	CB	CYS	A	437	-3.317	11.854	27.520	1.00	19.38	C
ATOM	3443	SG	CYS	A	437	-3.502	10.914	25.959	1.00	24.98	S
ATOM	3444	N	CYS	A	438	-3.170	14.449	29.484	1.00	23.70	N
ATOM	3445	CA	CYS	A	438	-3.717	15.267	30.546	1.00	22.08	C
ATOM	3446	C	CYS	A	438	-4.216	16.551	29.900	1.00	23.11	C
ATOM	3447	O	CYS	A	438	-5.390	16.891	30.012	1.00	23.14	O
ATOM	3448	CB	CYS	A	438	-2.650	15.582	31.595	1.00	19.90	C
ATOM	3449	SG	CYS	A	438	-1.760	14.132	32.242	1.00	17.60	S
ATOM	3450	N	LYS	A	439	-3.378	17.119	29.037	1.00	25.03	N
ATOM	3451	CA	LYS	A	439	-3.676	18.377	28.346	1.00	25.75	C
ATOM	3452	C	LYS	A	439	-4.672	18.082	27.239	1.00	25.01	C
ATOM	3453	O	LYS	A	439	-4.405	18.336	26.059	1.00	22.62	O
ATOM	3454	CB	LYS	A	439	-2.385	18.981	27.761	1.00	27.02	C
ATOM	3455	CG	LYS	A	439	-2.044	20.364	28.297	1.00	26.58	C
ATOM	3456	CD	LYS	A	439	-0.569	20.490	28.681	1.00	25.12	C
ATOM	3457	CE	LYS	A	439	-0.261	21.909	29.205	1.00	26.40	C
ATOM	3458	NZ	LYS	A	439	1.186	22.172	29.539	1.00	24.08	N1+
ATOM	3459	N	HIS	A	440	-5.835	17.575	27.646	1.00	25.71	N
ATOM	3460	CA	HIS	A	440	-6.781	16.952	26.733	1.00	25.96	C
ATOM	3461	C	HIS	A	440	-8.119	16.494	27.332	1.00	26.52	C
ATOM	3462	O	HIS	A	440	-8.967	16.005	26.602	1.00	27.11	O
ATOM	3463	CB	HIS	A	440	-6.108	15.762	26.054	1.00	24.62	C
ATOM	3464	CG	HIS	A	440	-7.055	14.877	25.323	1.00	20.53	C
ATOM	3465	CD2	HIS	A	440	-7.449	13.606	25.559	1.00	20.18	C
ATOM	3466	ND1	HIS	A	440	-7.787	15.313	24.247	1.00	21.98	N
ATOM	3467	CE1	HIS	A	440	-8.575	14.341	23.830	1.00	22.57	C
ATOM	3468	NE2	HIS	A	440	-8.386	13.291	24.607	1.00	20.14	N
ATOM	3469	N	PRO	A	441	-8.400	16.840	28.595	1.00	28.45	N
ATOM	3470	CA	PRO	A	441	-9.107	15.970	29.553	1.00	30.20	C
ATOM	3471	C	PRO	A	441	-10.490	15.409	29.137	1.00	30.12	C
ATOM	3472	O	PRO	A	441	-10.683	15.001	27.984	1.00	28.70	O
ATOM	3473	CB	PRO	A	441	-9.202	16.838	30.811	1.00	32.36	C
ATOM	3474	CG	PRO	A	441	-9.186	18.258	30.292	1.00	32.89	C
ATOM	3475	CD	PRO	A	441	-8.282	18.229	29.090	1.00	30.04	C
ATOM	3476	N	GLU	A	442	-11.410	15.285	30.101	1.00	29.30	N

ATOM	3477	CA	GLU	A	442	-12.751	14.720	29.866	1.00	29.85	C
ATOM	3478	C	GLU	A	442	-12.863	13.169	29.936	1.00	28.51	C
ATOM	3479	O	GLU	A	442	-12.375	12.533	30.885	1.00	22.26	O
ATOM	3480	CB	GLU	A	442	-13.324	15.201	28.507	1.00	29.58	C
ATOM	3481	CG	GLU	A	442	-13.697	16.673	28.422	1.00	26.93	C
ATOM	3482	CD	GLU	A	442	-12.757	17.475	27.541	1.00	25.31	C
ATOM	3483	OE1	GLU	A	442	-11.572	17.123	27.442	1.00	20.53	O
ATOM	3484	OE2	GLU	A	442	-13.192	18.505	26.974	1.00	28.11	O1-
ATOM	3485	N	ALA	A	443	-13.822	12.669	29.147	1.00	27.70	N
ATOM	3486	CA	ALA	A	443	-13.825	11.304	28.638	1.00	26.01	C
ATOM	3487	C	ALA	A	443	-12.551	11.111	27.852	1.00	24.19	C
ATOM	3488	O	ALA	A	443	-11.639	10.447	28.316	1.00	26.28	O
ATOM	3489	CB	ALA	A	443	-15.016	11.089	27.708	1.00	26.77	C
ATOM	3490	N	LYS	A	444	-12.411	11.892	26.788	1.00	22.05	N
ATOM	3491	CA	LYS	A	444	-11.370	11.674	25.785	1.00	21.39	C
ATOM	3492	C	LYS	A	444	-10.000	11.321	26.357	1.00	20.82	C
ATOM	3493	O	LYS	A	444	-9.137	10.831	25.620	1.00	18.13	O
ATOM	3494	CB	LYS	A	444	-11.248	12.911	24.894	1.00	22.35	C
ATOM	3495	CG	LYS	A	444	-11.775	12.721	23.484	1.00	22.26	C
ATOM	3496	CD	LYS	A	444	-13.306	12.807	23.457	1.00	20.62	C
ATOM	3497	CE	LYS	A	444	-13.783	14.030	22.666	1.00	18.58	C
ATOM	3498	NZ	LYS	A	444	-13.011	15.272	22.977	1.00	15.81	N1+
ATOM	3499	N	ARG	A	445	-9.762	11.777	27.594	1.00	20.03	N
ATOM	3500	CA	ARG	A	445	-8.614	11.392	28.429	1.00	18.49	C
ATOM	3501	C	ARG	A	445	-8.397	9.892	28.555	1.00	19.53	C
ATOM	3502	O	ARG	A	445	-7.514	9.343	27.914	1.00	21.22	O
ATOM	3503	CB	ARG	A	445	-8.758	11.970	29.830	1.00	14.76	C
ATOM	3504	CG	ARG	A	445	-7.643	11.580	30.764	1.00	10.97	C
ATOM	3505	CD	ARG	A	445	-7.994	11.871	32.222	1.00	11.73	C
ATOM	3506	NE	ARG	A	445	-8.114	13.288	32.629	1.00	13.19	N
ATOM	3507	CZ	ARG	A	445	-7.458	14.336	32.122	1.00	9.58	C
ATOM	3508	NH1	ARG	A	445	-6.945	14.323	30.899	1.00	7.80	N1+
ATOM	3509	NH2	ARG	A	445	-7.497	15.482	32.775	1.00	6.22	N
ATOM	3510	N	MET	A	446	-9.204	9.204	29.348	1.00	20.28	N
ATOM	3511	CA	MET	A	446	-8.894	7.805	29.608	1.00	22.11	C
ATOM	3512	C	MET	A	446	-8.796	6.892	28.368	1.00	21.20	C
ATOM	3513	O	MET	A	446	-7.843	6.105	28.273	1.00	21.62	O
ATOM	3514	CB	MET	A	446	-9.830	7.188	30.659	1.00	22.74	C
ATOM	3515	CG	MET	A	446	-9.620	5.693	30.794	1.00	24.58	C
ATOM	3516	SD	MET	A	446	-9.860	4.949	32.408	1.00	28.20	S
ATOM	3517	CE	MET	A	446	-11.076	3.660	31.945	1.00	22.67	C
ATOM	3518	N	PRO	A	447	-9.733	6.979	27.405	1.00	20.32	N
ATOM	3519	CA	PRO	A	447	-9.574	6.042	26.294	1.00	22.10	C
ATOM	3520	C	PRO	A	447	-8.133	6.079	25.772	1.00	23.45	C
ATOM	3521	O	PRO	A	447	-7.541	5.014	25.563	1.00	25.75	O
ATOM	3522	CB	PRO	A	447	-10.578	6.532	25.234	1.00	21.91	C
ATOM	3523	CG	PRO	A	447	-11.561	7.311	25.970	1.00	20.33	C
ATOM	3524	CD	PRO	A	447	-10.792	7.962	27.113	1.00	22.51	C
ATOM	3525	N	CYS	A	448	-7.509	7.266	25.813	1.00	21.39	N
ATOM	3526	CA	CYS	A	448	-6.156	7.436	25.279	1.00	19.05	C
ATOM	3527	C	CYS	A	448	-5.183	6.767	26.222	1.00	18.85	C
ATOM	3528	O	CYS	A	448	-4.317	6.025	25.775	1.00	21.92	O
ATOM	3529	CB	CYS	A	448	-5.756	8.916	25.173	1.00	19.04	C
ATOM	3530	SG	CYS	A	448	-4.591	9.295	26.505	1.00	20.87	S
ATOM	3531	N	ALA	A	449	-5.386	6.971	27.527	1.00	17.31	N
ATOM	3532	CA	ALA	A	449	-4.314	6.924	28.522	1.00	13.97	C
ATOM	3533	C	ALA	A	449	-4.209	5.679	29.390	1.00	16.06	C
ATOM	3534	O	ALA	A	449	-3.241	5.544	30.100	1.00	20.40	O
ATOM	3535	CB	ALA	A	449	-4.376	8.142	29.409	1.00	10.09	C
ATOM	3536	N	GLU	A	450	-5.188	4.780	29.374	1.00	17.26	N
ATOM	3537	CA	GLU	A	450	-4.941	3.417	29.855	1.00	16.55	C
ATOM	3538	C	GLU	A	450	-4.304	2.693	28.672	1.00	18.65	C
ATOM	3539	O	GLU	A	450	-3.139	2.330	28.712	1.00	18.04	O
ATOM	3540	CB	GLU	A	450	-6.245	2.725	30.241	1.00	17.28	C
ATOM	3541	CG	GLU	A	450	-6.151	1.924	31.533	1.00	15.50	C
ATOM	3542	CD	GLU	A	450	-7.098	0.716	31.585	1.00	18.82	C
ATOM	3543	OE1	GLU	A	450	-8.213	0.768	31.027	1.00	17.57	O
ATOM	3544	OE2	GLU	A	450	-6.726	-0.310	32.195	1.00	21.24	O1-
ATOM	3545	N	ASP	A	451	-4.985	2.799	27.531	1.00	19.88	N
ATOM	3546	CA	ASP	A	451	-4.495	2.392	26.213	1.00	18.66	C
ATOM	3547	C	ASP	A	451	-3.055	2.711	25.815	1.00	18.58	C

ATOM	3548	O	ASP	A	451	-2.341	1.810	25.397	1.00	20.61	O
ATOM	3549	CB	ASP	A	451	-5.435	2.927	25.140	1.00	17.00	C
ATOM	3550	CG	ASP	A	451	-6.675	2.088	25.009	1.00	19.38	C
ATOM	3551	OD1	ASP	A	451	-6.556	0.869	25.262	1.00	16.86	O
ATOM	3552	OD2	ASP	A	451	-7.762	2.634	24.690	1.00	19.70	O1-
ATOM	3553	N	TYR	A	452	-2.658	3.981	25.827	1.00	16.13	N
ATOM	3554	CA	TYR	A	452	-1.281	4.344	25.489	1.00	15.08	C
ATOM	3555	C	TYR	A	452	-0.335	3.655	26.464	1.00	16.14	C
ATOM	3556	O	TYR	A	452	0.481	2.837	26.070	1.00	17.95	O
ATOM	3557	CB	TYR	A	452	-1.093	5.854	25.560	1.00	13.93	C
ATOM	3558	CG	TYR	A	452	0.277	6.331	25.144	1.00	15.16	C
ATOM	3559	CD1	TYR	A	452	1.381	6.207	25.995	1.00	13.94	C
ATOM	3560	CD2	TYR	A	452	0.463	6.960	23.919	1.00	15.41	C
ATOM	3561	CE1	TYR	A	452	2.634	6.698	25.624	1.00	13.09	C
ATOM	3562	CE2	TYR	A	452	1.706	7.450	23.550	1.00	15.00	C
ATOM	3563	CZ	TYR	A	452	2.779	7.318	24.401	1.00	12.67	C
ATOM	3564	OH	TYR	A	452	3.973	7.861	24.029	1.00	11.49	O
ATOM	3565	N	LEU	A	453	-0.498	3.931	27.751	1.00	16.23	N
ATOM	3566	CA	LEU	A	453	0.196	3.200	28.794	1.00	13.79	C
ATOM	3567	C	LEU	A	453	0.231	1.710	28.457	1.00	13.05	C
ATOM	3568	O	LEU	A	453	1.301	1.125	28.413	1.00	15.56	O
ATOM	3569	CB	LEU	A	453	-0.502	3.427	30.135	1.00	11.83	C
ATOM	3570	CG	LEU	A	453	0.131	4.454	31.073	1.00	13.55	C
ATOM	3571	CD1	LEU	A	453	-0.933	5.105	31.932	1.00	11.05	C
ATOM	3572	CD2	LEU	A	453	1.178	3.782	31.944	1.00	10.99	C
ATOM	3573	N	SER	A	454	-0.879	1.181	27.955	1.00	11.72	N
ATOM	3574	CA	SER	A	454	-0.980	-0.244	27.649	1.00	11.87	C
ATOM	3575	C	SER	A	454	-0.003	-0.699	26.557	1.00	12.13	C
ATOM	3576	O	SER	A	454	0.518	-1.825	26.598	1.00	8.69	O
ATOM	3577	CB	SER	A	454	-2.408	-0.605	27.220	1.00	14.00	C
ATOM	3578	OG	SER	A	454	-3.390	0.232	27.809	1.00	11.75	O
ATOM	3579	N	VAL	A	455	0.155	0.154	25.543	1.00	11.63	N
ATOM	3580	CA	VAL	A	455	1.107	-0.046	24.450	1.00	10.07	C
ATOM	3581	C	VAL	A	455	2.516	-0.105	25.015	1.00	11.00	C
ATOM	3582	O	VAL	A	455	3.288	-0.996	24.675	1.00	10.74	O
ATOM	3583	CB	VAL	A	455	1.075	1.128	23.425	1.00	11.18	C
ATOM	3584	CG1	VAL	A	455	2.127	0.913	22.352	1.00	10.50	C
ATOM	3585	CG2	VAL	A	455	-0.299	1.283	22.809	1.00	6.79	C
ATOM	3586	N	VAL	A	456	2.858	0.864	25.861	1.00	12.33	N
ATOM	3587	CA	VAL	A	456	4.227	1.008	26.324	1.00	15.30	C
ATOM	3588	C	VAL	A	456	4.669	-0.208	27.125	1.00	18.48	C
ATOM	3589	O	VAL	A	456	5.671	-0.858	26.797	1.00	21.87	O
ATOM	3590	CB	VAL	A	456	4.399	2.248	27.164	1.00	13.68	C
ATOM	3591	CG1	VAL	A	456	5.777	2.285	27.727	1.00	14.03	C
ATOM	3592	CG2	VAL	A	456	4.167	3.460	26.316	1.00	17.71	C
ATOM	3593	N	LEU	A	457	3.867	-0.585	28.110	1.00	18.16	N
ATOM	3594	CA	LEU	A	457	4.124	-1.806	28.837	1.00	17.51	C
ATOM	3595	C	LEU	A	457	4.406	-2.967	27.856	1.00	19.56	C
ATOM	3596	O	LEU	A	457	5.322	-3.758	28.077	1.00	23.05	O
ATOM	3597	CB	LEU	A	457	2.943	-2.096	29.782	1.00	14.10	C
ATOM	3598	CG	LEU	A	457	3.038	-1.457	31.183	1.00	10.11	C
ATOM	3599	CD1	LEU	A	457	3.844	-0.180	31.147	1.00	6.96	C
ATOM	3600	CD2	LEU	A	457	1.668	-1.183	31.723	1.00	6.91	C
ATOM	3601	N	ASN	A	458	3.820	-2.914	26.666	1.00	19.26	N
ATOM	3602	CA	ASN	A	458	4.044	-3.982	25.696	1.00	20.09	C
ATOM	3603	C	ASN	A	458	5.403	-3.848	25.054	1.00	20.20	C
ATOM	3604	O	ASN	A	458	6.144	-4.823	25.000	1.00	22.87	O
ATOM	3605	CB	ASN	A	458	2.967	-3.985	24.622	1.00	22.45	C
ATOM	3606	CG	ASN	A	458	3.165	-5.070	23.594	1.00	23.30	C
ATOM	3607	ND2	ASN	A	458	3.201	-4.665	22.348	1.00	27.57	N
ATOM	3608	OD1	ASN	A	458	3.122	-6.263	23.895	1.00	25.73	O
ATOM	3609	N	GLN	A	459	5.782	-2.627	24.683	1.00	18.71	N
ATOM	3610	CA	GLN	A	459	7.156	-2.341	24.284	1.00	16.79	C
ATOM	3611	C	GLN	A	459	8.104	-2.947	25.318	1.00	19.53	C
ATOM	3612	O	GLN	A	459	8.829	-3.897	25.000	1.00	20.10	O
ATOM	3613	CB	GLN	A	459	7.375	-0.850	24.212	1.00	16.06	C
ATOM	3614	CG	GLN	A	459	8.559	-0.451	23.411	1.00	19.00	C
ATOM	3615	CD	GLN	A	459	8.212	-0.265	21.970	1.00	18.86	C
ATOM	3616	NE2	GLN	A	459	7.618	0.865	21.647	1.00	18.61	N
ATOM	3617	OE1	GLN	A	459	8.431	-1.150	21.156	1.00	20.77	O
ATOM	3618	N	LEU	A	460	7.894	-2.591	26.591	1.00	19.18	N

ATOM	3619	CA	LEU	A	460	8.570	-3.248	27.725	1.00	17.82	C
ATOM	3620	C	LEU	A	460	8.632	-4.768	27.635	1.00	18.45	C
ATOM	3621	O	LEU	A	460	9.692	-5.338	27.877	1.00	21.46	O
ATOM	3622	CB	LEU	A	460	7.919	-2.860	29.063	1.00	15.63	C
ATOM	3623	CG	LEU	A	460	8.279	-3.617	30.355	1.00	14.04	C
ATOM	3624	CD1	LEU	A	460	9.785	-3.769	30.501	1.00	15.77	C
ATOM	3625	CD2	LEU	A	460	7.747	-2.867	31.555	1.00	12.12	C
ATOM	3626	N	CYS	A	461	7.513	-5.430	27.330	1.00	20.02	N
ATOM	3627	CA	CYS	A	461	7.470	-6.904	27.322	1.00	20.16	C
ATOM	3628	C	CYS	A	461	8.172	-7.527	26.121	1.00	21.18	C
ATOM	3629	O	CYS	A	461	8.929	-8.505	26.272	1.00	19.43	O
ATOM	3630	CB	CYS	A	461	6.028	-7.408	27.348	1.00	17.64	C
ATOM	3631	SG	CYS	A	461	5.081	-6.878	28.795	1.00	17.61	S
ATOM	3632	N	VAL	A	462	7.942	-6.920	24.950	1.00	21.46	N
ATOM	3633	CA	VAL	A	462	8.437	-7.401	23.660	1.00	22.27	C
ATOM	3634	C	VAL	A	462	9.942	-7.136	23.468	1.00	23.83	C
ATOM	3635	O	VAL	A	462	10.679	-7.973	22.924	1.00	22.80	O
ATOM	3636	CB	VAL	A	462	7.672	-6.738	22.517	1.00	20.37	C
ATOM	3637	CG1	VAL	A	462	6.179	-6.891	22.734	1.00	20.18	C
ATOM	3638	CG2	VAL	A	462	8.039	-5.304	22.420	1.00	17.82	C
ATOM	3639	N	LEU	A	463	10.358	-5.933	23.863	1.00	24.45	N
ATOM	3640	CA	LEU	A	463	11.749	-5.617	24.183	1.00	23.28	C
ATOM	3641	C	LEU	A	463	12.444	-6.794	24.843	1.00	23.88	C
ATOM	3642	O	LEU	A	463	13.285	-7.448	24.233	1.00	25.91	O
ATOM	3643	CB	LEU	A	463	11.800	-4.420	25.144	1.00	20.02	C
ATOM	3644	CG	LEU	A	463	12.855	-3.340	24.952	1.00	16.71	C
ATOM	3645	CD1	LEU	A	463	13.665	-3.676	23.734	1.00	14.34	C
ATOM	3646	CD2	LEU	A	463	12.196	-1.970	24.804	1.00	15.27	C
ATOM	3647	N	HIS	A	464	12.115	-7.035	26.106	1.00	25.21	N
ATOM	3648	CA	HIS	A	464	12.941	-7.887	26.951	1.00	27.10	C
ATOM	3649	C	HIS	A	464	12.269	-9.220	27.310	1.00	25.73	C
ATOM	3650	O	HIS	A	464	11.668	-9.364	28.380	1.00	26.56	O
ATOM	3651	CB	HIS	A	464	13.392	-7.121	28.224	1.00	27.53	C
ATOM	3652	CG	HIS	A	464	14.883	-7.158	28.474	1.00	26.94	C
ATOM	3653	CD2	HIS	A	464	15.904	-7.678	27.738	1.00	21.05	C
ATOM	3654	ND1	HIS	A	464	15.464	-6.665	29.625	1.00	24.27	N
ATOM	3655	CE1	HIS	A	464	16.764	-6.892	29.600	1.00	20.67	C
ATOM	3656	NE2	HIS	A	464	17.053	-7.502	28.464	1.00	17.48	N
ATOM	3657	N	GLU	A	465	12.180	-10.082	26.305	1.00	23.46	N
ATOM	3658	CA	GLU	A	465	12.280	-11.518	26.492	1.00	21.43	C
ATOM	3659	C	GLU	A	465	13.764	-11.787	26.213	1.00	21.62	C
ATOM	3660	O	GLU	A	465	14.509	-12.216	27.092	1.00	20.90	O
ATOM	3661	CB	GLU	A	465	11.406	-12.258	25.458	1.00	23.62	C
ATOM	3662	CG	GLU	A	465	9.867	-12.214	25.681	1.00	20.17	C
ATOM	3663	CD	GLU	A	465	9.063	-12.794	24.503	1.00	18.19	C
ATOM	3664	OE1	GLU	A	465	8.978	-12.111	23.465	1.00	15.65	O
ATOM	3665	OE2	GLU	A	465	8.455	-13.888	24.626	1.00	15.39	O1-
ATOM	3666	N	LYS	A	466	14.199	-11.367	25.024	1.00	22.56	N
ATOM	3667	CA	LYS	A	466	15.611	-11.327	24.604	1.00	24.39	C
ATOM	3668	C	LYS	A	466	16.664	-11.640	25.685	1.00	21.61	C
ATOM	3669	O	LYS	A	466	17.404	-12.606	25.542	1.00	22.78	O
ATOM	3670	CB	LYS	A	466	15.899	-9.967	23.918	1.00	26.20	C
ATOM	3671	CG	LYS	A	466	17.381	-9.571	23.804	1.00	27.48	C
ATOM	3672	CD	LYS	A	466	17.668	-8.239	24.520	1.00	26.14	C
ATOM	3673	CE	LYS	A	466	19.123	-7.796	24.343	1.00	23.78	C
ATOM	3674	NZ	LYS	A	466	20.084	-8.644	25.115	1.00	20.55	N1+
ATOM	3675	N	THR	A	467	16.826	-10.776	26.681	1.00	19.96	N
ATOM	3676	CA	THR	A	467	17.514	-11.183	27.914	1.00	22.51	C
ATOM	3677	C	THR	A	467	16.397	-11.294	28.923	1.00	25.70	C
ATOM	3678	O	THR	A	467	15.585	-10.359	29.041	1.00	29.10	O
ATOM	3679	CB	THR	A	467	18.489	-10.112	28.487	1.00	16.74	C
ATOM	3680	CG2	THR	A	467	19.829	-10.712	28.809	1.00	14.95	C
ATOM	3681	OG1	THR	A	467	18.653	-9.056	27.550	1.00	14.94	O
ATOM	3682	N	PRO	A	468	16.405	-12.344	29.752	1.00	25.15	N
ATOM	3683	CA	PRO	A	468	15.248	-12.352	30.645	1.00	26.23	C
ATOM	3684	C	PRO	A	468	15.654	-11.886	32.035	1.00	25.34	C
ATOM	3685	O	PRO	A	468	16.667	-12.324	32.567	1.00	24.12	O
ATOM	3686	CB	PRO	A	468	14.786	-13.809	30.609	1.00	27.92	C
ATOM	3687	CG	PRO	A	468	15.621	-14.461	29.496	1.00	26.78	C
ATOM	3688	CD	PRO	A	468	16.904	-13.702	29.528	1.00	23.56	C
ATOM	3689	N	VAL	A	469	14.900	-10.945	32.589	1.00	26.09	N

```
ATOM   3690  CA  VAL A 469      15.269 -10.322  33.863  1.00 26.57           C
ATOM   3691  C   VAL A 469      14.259 -10.507  35.043  1.00 26.29           C
ATOM   3692  O   VAL A 469      14.272 -11.537  35.720  1.00 25.05           O
ATOM   3693  CB  VAL A 469      15.609  -8.808  33.643  1.00 25.96           C
ATOM   3694  CG1 VAL A 469      17.067  -8.663  33.262  1.00 23.63           C
ATOM   3695  CG2 VAL A 469      14.723  -8.211  32.541  1.00 22.41           C
ATOM   3696  N   SER A 470      13.398  -9.520  35.283  1.00 24.94           N
ATOM   3697  CA  SER A 470      12.584  -9.486  36.492  1.00 25.10           C
ATOM   3698  C   SER A 470      11.436 -10.470  36.378  1.00 26.22           C
ATOM   3699  O   SER A 470      10.438 -10.196  35.697  1.00 27.50           O
ATOM   3700  CB  SER A 470      12.021  -8.070  36.744  1.00 24.74           C
ATOM   3701  OG  SER A 470      11.640  -7.884  38.110  1.00 22.77           O
ATOM   3702  N   ASP A 471      11.516 -11.553  37.145  1.00 25.67           N
ATOM   3703  CA  ASP A 471      10.504 -12.596  37.091  1.00 25.01           C
ATOM   3704  C   ASP A 471       9.126 -11.976  37.245  1.00 26.17           C
ATOM   3705  O   ASP A 471       8.146 -12.438  36.640  1.00 27.11           O
ATOM   3706  CB  ASP A 471      10.765 -13.620  38.175  1.00 23.33           C
ATOM   3707  CG  ASP A 471      12.078 -14.320  37.978  1.00 25.27           C
ATOM   3708  OD1 ASP A 471      13.125 -13.757  38.359  1.00 29.79           O
ATOM   3709  OD2 ASP A 471      12.086 -15.384  37.335  1.00 29.40           O1-
ATOM   3710  N   ARG A 472       9.108 -10.793  37.853  1.00 25.75           N
ATOM   3711  CA  ARG A 472       7.884 -10.014  37.949  1.00 26.02           C
ATOM   3712  C   ARG A 472       7.420  -9.534  36.583  1.00 26.00           C
ATOM   3713  O   ARG A 472       6.279  -9.785  36.182  1.00 26.81           O
ATOM   3714  CB  ARG A 472       8.098  -8.836  38.897  1.00 25.19           C
ATOM   3715  CG  ARG A 472       8.527  -9.296  40.283  1.00 24.67           C
ATOM   3716  CD  ARG A 472       7.413  -9.145  41.277  1.00 20.81           C
ATOM   3717  NE  ARG A 472       7.918  -8.558  42.510  1.00 15.73           N
ATOM   3718  CZ  ARG A 472       7.177  -7.901  43.390  1.00 15.03           C
ATOM   3719  NH1 ARG A 472       5.946  -7.542  43.087  1.00 15.14           N1+
ATOM   3720  NH2 ARG A 472       7.720  -7.494  44.523  1.00 16.85           N
ATOM   3721  N   VAL A 473       8.323  -8.888  35.853  1.00 24.83           N
ATOM   3722  CA  VAL A 473       8.029  -8.415  34.512  1.00 23.45           C
ATOM   3723  C   VAL A 473       7.641  -9.624  33.669  1.00 22.85           C
ATOM   3724  O   VAL A 473       6.743  -9.554  32.840  1.00 22.94           O
ATOM   3725  CB  VAL A 473       9.259  -7.717  33.873  1.00 25.33           C
ATOM   3726  CG1 VAL A 473       8.869  -7.093  32.530  1.00 23.20           C
ATOM   3727  CG2 VAL A 473       9.845  -6.670  34.828  1.00 19.55           C
ATOM   3728  N   THR A 474       8.260 -10.755  33.947  1.00 21.09           N
ATOM   3729  CA  THR A 474       7.847 -11.976  33.298  1.00 24.95           C
ATOM   3730  C   THR A 474       6.372 -12.199  33.594  1.00 25.61           C
ATOM   3731  O   THR A 474       5.565 -12.398  32.675  1.00 27.28           O
ATOM   3732  CB  THR A 474       8.647 -13.169  33.829  1.00 27.15           C
ATOM   3733  CG2 THR A 474       8.638 -14.329  32.811  1.00 28.51           C
ATOM   3734  OG1 THR A 474       9.997 -12.748  34.084  1.00 29.10           O
ATOM   3735  N   LYS A 475       6.000 -12.011  34.856  1.00 26.00           N
ATOM   3736  CA  LYS A 475       4.657 -12.344  35.320  1.00 24.30           C
ATOM   3737  C   LYS A 475       3.533 -11.428  34.798  1.00 21.93           C
ATOM   3738  O   LYS A 475       2.528 -11.951  34.305  1.00 20.33           O
ATOM   3739  CB  LYS A 475       4.636 -12.427  36.858  1.00 25.66           C
ATOM   3740  CG  LYS A 475       4.745 -13.872  37.436  1.00 27.40           C
ATOM   3741  CD  LYS A 475       6.131 -14.541  37.206  1.00 29.53           C
ATOM   3742  CE  LYS A 475       6.706 -15.275  38.468  1.00 27.50           C
ATOM   3743  NZ  LYS A 475       7.235 -14.367  39.560  1.00 20.78           N1+
ATOM   3744  N   CYS A 476       3.730 -10.098  34.805  1.00 18.81           N
ATOM   3745  CA  CYS A 476       2.672  -9.129  34.411  1.00 14.74           C
ATOM   3746  C   CYS A 476       2.446  -9.020  32.891  1.00 14.14           C
ATOM   3747  O   CYS A 476       1.340  -8.728  32.413  1.00 10.01           O
ATOM   3748  CB  CYS A 476       2.973  -7.735  34.969  1.00 14.71           C
ATOM   3749  SG  CYS A 476       2.665  -7.476  36.756  1.00 17.94           S
ATOM   3750  N   CYS A 477       3.502  -9.291  32.135  1.00 14.05           N
ATOM   3751  CA  CYS A 477       3.383  -9.530  30.702  1.00 12.13           C
ATOM   3752  C   CYS A 477       2.553 -10.798  30.380  1.00 11.06           C
ATOM   3753  O   CYS A 477       1.395 -10.686  29.966  1.00  6.49           O
ATOM   3754  CB  CYS A 477       4.794  -9.588  30.090  1.00 13.42           C
ATOM   3755  SG  CYS A 477       5.744  -8.034  30.324  1.00 14.64           S
ATOM   3756  N   THR A 478       3.070 -11.969  30.755  1.00 11.58           N
ATOM   3757  CA  THR A 478       2.425 -13.263  30.481  1.00 14.89           C
ATOM   3758  C   THR A 478       1.027 -13.474  31.033  1.00 15.80           C
ATOM   3759  O   THR A 478       0.194 -14.100  30.369  1.00 16.44           O
ATOM   3760  CB  THR A 478       3.241 -14.447  31.035  1.00 18.24           C
```

ATOM	3761	CG2	THR	A	478	3.479	-14.300	32.546	1.00 16.23	C
ATOM	3762	OG1	THR	A	478	2.514	-15.661	30.807	1.00 19.21	O
ATOM	3763	N	GLU	A	479	0.903	-13.261	32.343	1.00 15.82	N
ATOM	3764	CA	GLU	A	479	-0.286	-13.641	33.070	1.00 18.03	C
ATOM	3765	C	GLU	A	479	-1.524	-12.993	32.455	1.00 19.17	C
ATOM	3766	O	GLU	A	479	-2.474	-13.703	32.167	1.00 20.64	O
ATOM	3767	CB	GLU	A	479	-0.144	-13.319	34.572	1.00 20.48	C
ATOM	3768	CG	GLU	A	479	0.926	-14.185	35.346	1.00 25.02	C
ATOM	3769	CD	GLU	A	479	0.428	-15.556	35.910	1.00 26.36	C
ATOM	3770	OE1	GLU	A	479	-0.438	-15.569	36.819	1.00 27.61	O
ATOM	3771	OE2	GLU	A	479	1.014	-16.614	35.558	1.00 23.89	O1-
ATOM	3772	N	SER	A	480	-1.476	-11.697	32.129	1.00 21.19	N
ATOM	3773	CA	SER	A	480	-2.479	-11.102	31.229	1.00 23.00	C
ATOM	3774	C	SER	A	480	-2.032	-9.944	30.320	1.00 22.37	C
ATOM	3775	O	SER	A	480	-1.343	-9.022	30.768	1.00 23.67	O
ATOM	3776	CB	SER	A	480	-3.703	-10.633	32.019	1.00 25.73	C
ATOM	3777	OG	SER	A	480	-4.717	-10.096	31.159	1.00 24.90	O
ATOM	3778	N	LEU	A	481	-2.642	-9.900	29.133	1.00 18.32	N
ATOM	3779	CA	LEU	A	481	-2.721	-8.692	28.306	1.00 17.04	C
ATOM	3780	C	LEU	A	481	-3.581	-7.681	29.029	1.00 16.84	C
ATOM	3781	O	LEU	A	481	-3.162	-6.563	29.292	1.00 16.88	O
ATOM	3782	CB	LEU	A	481	-3.392	-8.992	26.950	1.00 12.89	C
ATOM	3783	CG	LEU	A	481	-3.438	-7.943	25.831	1.00 8.60	C
ATOM	3784	CD1	LEU	A	481	-2.107	-7.944	25.173	1.00 11.20	C
ATOM	3785	CD2	LEU	A	481	-4.510	-8.235	24.771	1.00 2.00	C
ATOM	3786	N	VAL	A	482	-4.791	-8.109	29.364	1.00 18.70	N
ATOM	3787	CA	VAL	A	482	-5.873	-7.205	29.741	1.00 17.38	C
ATOM	3788	C	VAL	A	482	-5.656	-6.631	31.146	1.00 15.41	C
ATOM	3789	O	VAL	A	482	-6.034	-5.490	31.420	1.00 12.39	O
ATOM	3790	CB	VAL	A	482	-7.235	-7.944	29.665	1.00 19.15	C
ATOM	3791	CG1	VAL	A	482	-8.371	-6.944	29.602	1.00 16.59	C
ATOM	3792	CG2	VAL	A	482	-7.262	-8.910	28.452	1.00 14.21	C
ATOM	3793	N	ASN	A	483	-4.887	-7.349	31.962	1.00 15.51	N
ATOM	3794	CA	ASN	A	483	-4.543	-6.871	33.313	1.00 19.45	C
ATOM	3795	C	ASN	A	483	-3.117	-6.282	33.448	1.00 18.86	C
ATOM	3796	O	ASN	A	483	-2.541	-6.286	34.527	1.00 18.09	O
ATOM	3797	CB	ASN	A	483	-4.744	-7.999	34.349	1.00 20.35	C
ATOM	3798	CG	ASN	A	483	-6.101	-8.689	34.212	1.00 23.29	C
ATOM	3799	ND2	ASN	A	483	-7.134	-8.065	34.770	1.00 23.14	N
ATOM	3800	OD1	ASN	A	483	-6.232	-9.728	33.544	1.00 20.79	O
ATOM	3801	N	ARG	A	484	-2.627	-5.632	32.400	1.00 19.94	N
ATOM	3802	CA	ARG	A	484	-1.235	-5.195	32.348	1.00 18.62	C
ATOM	3803	C	ARG	A	484	-1.091	-4.010	33.255	1.00 16.95	C
ATOM	3804	O	ARG	A	484	-0.397	-4.077	34.247	1.00 15.09	O
ATOM	3805	CB	ARG	A	484	-0.844	-4.773	30.920	1.00 22.74	C
ATOM	3806	CG	ARG	A	484	0.231	-5.615	30.212	1.00 18.99	C
ATOM	3807	CD	ARG	A	484	0.209	-5.384	28.687	1.00 16.43	C
ATOM	3808	NE	ARG	A	484	1.293	-6.102	28.008	1.00 17.41	N
ATOM	3809	CZ	ARG	A	484	1.157	-7.231	27.313	1.00 16.27	C
ATOM	3810	NH1	ARG	A	484	-0.025	-7.784	27.149	1.00 17.18	N1+
ATOM	3811	NH2	ARG	A	484	2.216	-7.812	26.767	1.00 20.22	N
ATOM	3812	N	ARG	A	485	-1.868	-2.973	32.992	1.00 17.31	N
ATOM	3813	CA	ARG	A	485	-1.641	-1.679	33.626	1.00 19.94	C
ATOM	3814	C	ARG	A	485	-2.259	-1.408	35.051	1.00 22.79	C
ATOM	3815	O	ARG	A	485	-1.991	-0.351	35.670	1.00 22.25	O
ATOM	3816	CB	ARG	A	485	-2.043	-0.583	32.637	1.00 16.29	C
ATOM	3817	CG	ARG	A	485	-1.603	0.799	33.039	1.00 16.70	C
ATOM	3818	CD	ARG	A	485	-2.801	1.708	33.276	1.00 15.57	C
ATOM	3819	NE	ARG	A	485	-3.930	1.020	33.887	1.00 12.17	N
ATOM	3820	CZ	ARG	A	485	-4.215	1.091	35.173	1.00 11.81	C
ATOM	3821	NH1	ARG	A	485	-3.411	1.751	35.978	1.00 15.13	N1+
ATOM	3822	NH2	ARG	A	485	-5.192	0.371	35.680	1.00 12.58	N
ATOM	3823	N	PRO	A	486	-3.067	-2.350	35.598	1.00 22.59	N
ATOM	3824	CA	PRO	A	486	-3.024	-2.488	37.067	1.00 23.53	C
ATOM	3825	C	PRO	A	486	-2.092	-3.558	37.639	1.00 21.48	C
ATOM	3826	O	PRO	A	486	-2.263	-3.997	38.779	1.00 18.78	O
ATOM	3827	CB	PRO	A	486	-4.489	-2.756	37.448	1.00 23.69	C
ATOM	3828	CG	PRO	A	486	-5.101	-3.264	36.224	1.00 19.85	C
ATOM	3829	CD	PRO	A	486	-4.446	-2.530	35.110	1.00 22.51	C
ATOM	3830	N	CYS	A	487	-1.118	-3.982	36.846	1.00 19.53	N
ATOM	3831	CA	CYS	A	487	-0.170	-4.986	37.304	1.00 19.20	C

ATOM	3832	C	CYS	A	487	1.135	-4.343	37.720	1.00	20.85	C
ATOM	3833	O	CYS	A	487	1.389	-4.179	38.908	1.00	24.17	O
ATOM	3834	CB	CYS	A	487	0.102	-6.004	36.223	1.00	15.12	C
ATOM	3835	SG	CYS	A	487	0.659	-7.543	36.947	1.00	12.46	S
ATOM	3836	N	PHE	A	488	1.882	-3.834	36.745	1.00	21.06	N
ATOM	3837	CA	PHE	A	488	3.057	-3.030	37.018	1.00	16.19	C
ATOM	3838	C	PHE	A	488	2.761	-2.078	38.133	1.00	16.14	C
ATOM	3839	O	PHE	A	488	3.334	-2.231	39.193	1.00	16.21	O
ATOM	3840	CB	PHE	A	488	3.487	-2.282	35.767	1.00	15.68	C
ATOM	3841	CG	PHE	A	488	4.434	-3.072	34.917	1.00	18.17	C
ATOM	3842	CD1	PHE	A	488	3.953	-4.049	34.066	1.00	14.32	C
ATOM	3843	CD2	PHE	A	488	5.800	-3.027	35.173	1.00	15.88	C
ATOM	3844	CE1	PHE	A	488	4.799	-4.961	33.530	1.00	13.05	C
ATOM	3845	CE2	PHE	A	488	6.638	-3.937	34.632	1.00	7.89	C
ATOM	3846	CZ	PHE	A	488	6.142	-4.907	33.821	1.00	11.30	C
ATOM	3847	N	SER	A	489	1.648	-1.356	37.989	1.00	16.65	N
ATOM	3848	CA	SER	A	489	1.124	-0.449	39.020	1.00	14.57	C
ATOM	3849	C	SER	A	489	1.020	-1.124	40.400	1.00	14.49	C
ATOM	3850	O	SER	A	489	1.604	-0.644	41.368	1.00	14.95	O
ATOM	3851	CB	SER	A	489	-0.261	0.084	38.606	1.00	13.40	C
ATOM	3852	OG	SER	A	489	-0.220	0.832	37.402	1.00	9.27	O
ATOM	3853	N	ALA	A	490	0.329	-2.264	40.471	1.00	14.36	N
ATOM	3854	CA	ALA	A	490	0.018	-2.922	41.746	1.00	14.42	C
ATOM	3855	C	ALA	A	490	1.174	-3.786	42.177	1.00	15.92	C
ATOM	3856	O	ALA	A	490	1.016	-4.996	42.395	1.00	16.03	O
ATOM	3857	CB	ALA	A	490	-1.233	-3.764	41.628	1.00	14.31	C
ATOM	3858	N	LEU	A	491	2.275	-3.111	42.483	1.00	16.50	N
ATOM	3859	CA	LEU	A	491	3.582	-3.730	42.630	1.00	17.68	C
ATOM	3860	C	LEU	A	491	4.264	-2.722	43.515	1.00	18.16	C
ATOM	3861	O	LEU	A	491	4.181	-1.532	43.235	1.00	19.45	O
ATOM	3862	CB	LEU	A	491	4.274	-3.755	41.260	1.00	18.42	C
ATOM	3863	CG	LEU	A	491	5.176	-4.844	40.673	1.00	19.23	C
ATOM	3864	CD1	LEU	A	491	4.410	-6.103	40.303	1.00	19.20	C
ATOM	3865	CD2	LEU	A	491	5.816	-4.257	39.436	1.00	21.21	C
ATOM	3866	N	GLU	A	492	4.742	-3.138	44.677	1.00	17.64	N
ATOM	3867	CA	GLU	A	492	5.441	-2.176	45.526	1.00	20.34	C
ATOM	3868	C	GLU	A	492	6.920	-2.316	45.191	1.00	20.14	C
ATOM	3869	O	GLU	A	492	7.255	-2.927	44.186	1.00	18.77	O
ATOM	3870	CB	GLU	A	492	5.143	-2.414	47.033	1.00	20.09	C
ATOM	3871	CG	GLU	A	492	3.620	-2.630	47.374	1.00	24.17	C
ATOM	3872	CD	GLU	A	492	2.836	-1.411	47.983	1.00	25.80	C
ATOM	3873	OE1	GLU	A	492	3.305	-0.784	48.959	1.00	27.22	O
ATOM	3874	OE2	GLU	A	492	1.648	-1.216	47.614	1.00	24.48	O1-
ATOM	3875	N	VAL	A	493	7.786	-1.623	45.924	1.00	21.53	N
ATOM	3876	CA	VAL	A	493	9.231	-1.795	45.779	1.00	20.93	C
ATOM	3877	C	VAL	A	493	9.635	-3.247	46.044	1.00	22.35	C
ATOM	3878	O	VAL	A	493	9.525	-3.722	47.170	1.00	24.31	O
ATOM	3879	CB	VAL	A	493	9.982	-0.910	46.767	1.00	18.51	C
ATOM	3880	CG1	VAL	A	493	11.487	-1.196	46.692	1.00	19.04	C
ATOM	3881	CG2	VAL	A	493	9.666	0.552	46.492	1.00	17.43	C
ATOM	3882	N	ASP	A	494	10.093	-3.951	45.016	1.00	24.29	N
ATOM	3883	CA	ASP	A	494	10.404	-5.382	45.148	1.00	25.27	C
ATOM	3884	C	ASP	A	494	11.691	-5.486	45.946	1.00	23.84	C
ATOM	3885	O	ASP	A	494	12.763	-5.666	45.375	1.00	22.03	O
ATOM	3886	CB	ASP	A	494	10.603	-6.067	43.764	1.00	25.69	C
ATOM	3887	CG	ASP	A	494	9.375	-5.952	42.826	1.00	21.60	C
ATOM	3888	OD1	ASP	A	494	8.405	-5.219	43.111	1.00	20.70	O
ATOM	3889	OD2	ASP	A	494	9.391	-6.593	41.768	1.00	16.55	O1-
ATOM	3890	N	GLU	A	495	11.558	-5.460	47.270	1.00	24.75	N
ATOM	3891	CA	GLU	A	495	12.703	-5.461	48.188	1.00	25.22	C
ATOM	3892	C	GLU	A	495	13.578	-6.701	48.020	1.00	25.09	C
ATOM	3893	O	GLU	A	495	14.671	-6.793	48.586	1.00	23.87	O
ATOM	3894	CB	GLU	A	495	12.229	-5.334	49.644	1.00	25.31	C
ATOM	3895	CG	GLU	A	495	11.358	-4.089	49.951	1.00	22.81	C
ATOM	3896	CD	GLU	A	495	9.976	-4.443	50.509	1.00	23.79	C
ATOM	3897	OE1	GLU	A	495	9.639	-5.650	50.564	1.00	20.96	O
ATOM	3898	OE2	GLU	A	495	9.223	-3.511	50.885	1.00	22.17	O1-
ATOM	3899	N	THR	A	496	13.107	-7.595	47.149	1.00	26.34	N
ATOM	3900	CA	THR	A	496	13.866	-8.694	46.535	1.00	23.22	C
ATOM	3901	C	THR	A	496	15.335	-8.416	46.231	1.00	22.62	C
ATOM	3902	O	THR	A	496	16.150	-9.331	46.277	1.00	22.94	O

```
ATOM   3903  CB  THR A 496      13.187  -9.146  45.199  1.00 21.63           C
ATOM   3904  CG2 THR A 496      12.732 -10.581  45.296  1.00 21.18           C
ATOM   3905  OG1 THR A 496      12.060  -8.302  44.898  1.00 16.30           O
ATOM   3906  N   TYR A 497      15.661  -7.150  45.975  1.00 22.20           N
ATOM   3907  CA  TYR A 497      16.844  -6.768  45.215  1.00 23.14           C
ATOM   3908  C   TYR A 497      18.148  -7.505  45.559  1.00 25.54           C
ATOM   3909  O   TYR A 497      18.420  -8.588  45.015  1.00 24.48           O
ATOM   3910  CB  TYR A 497      17.055  -5.256  45.330  1.00 23.83           C
ATOM   3911  CG  TYR A 497      17.784  -4.607  44.155  1.00 25.67           C
ATOM   3912  CD1 TYR A 497      17.903  -5.248  42.939  1.00 26.17           C
ATOM   3913  CD2 TYR A 497      18.419  -3.381  44.302  1.00 24.70           C
ATOM   3914  CE1 TYR A 497      18.656  -4.699  41.917  1.00 27.56           C
ATOM   3915  CE2 TYR A 497      19.163  -2.831  43.293  1.00 24.84           C
ATOM   3916  CZ  TYR A 497      19.298  -3.496  42.103  1.00 27.12           C
ATOM   3917  OH  TYR A 497      20.187  -3.028  41.144  1.00 25.38           O
ATOM   3918  N   VAL A 498      18.915  -6.947  46.504  1.00 26.48           N
ATOM   3919  CA  VAL A 498      20.386  -7.050  46.515  1.00 25.76           C
ATOM   3920  C   VAL A 498      20.815  -6.909  45.059  1.00 27.52           C
ATOM   3921  O   VAL A 498      20.314  -6.011  44.377  1.00 26.89           O
ATOM   3922  CB  VAL A 498      20.934  -8.371  47.162  1.00 25.41           C
ATOM   3923  CG1 VAL A 498      22.373  -8.146  47.637  1.00 23.31           C
ATOM   3924  CG2 VAL A 498      20.075  -8.802  48.361  1.00 25.94           C
ATOM   3925  N   PRO A 499      21.622  -7.846  44.516  1.00 30.00           N
ATOM   3926  CA  PRO A 499      22.892  -7.469  43.868  1.00 29.18           C
ATOM   3927  C   PRO A 499      23.366  -6.065  44.192  1.00 29.84           C
ATOM   3928  O   PRO A 499      24.553  -5.856  44.419  1.00 32.19           O
ATOM   3929  CB  PRO A 499      22.589  -7.640  42.383  1.00 29.25           C
ATOM   3930  CG  PRO A 499      21.521  -8.787  42.353  1.00 31.67           C
ATOM   3931  CD  PRO A 499      21.046  -9.013  43.821  1.00 29.92           C
ATOM   3932  N   LYS A 500      22.480  -5.092  44.014  1.00 29.43           N
ATOM   3933  CA  LYS A 500      22.604  -3.779  44.641  1.00 28.77           C
ATOM   3934  C   LYS A 500      23.964  -3.178  44.341  1.00 29.16           C
ATOM   3935  O   LYS A 500      24.597  -2.585  45.213  1.00 31.34           O
ATOM   3936  CB  LYS A 500      22.376  -3.880  46.153  1.00 25.37           C
ATOM   3937  CG  LYS A 500      21.848  -2.597  46.770  1.00 23.83           C
ATOM   3938  CD  LYS A 500      21.013  -2.876  48.001  1.00 24.99           C
ATOM   3939  CE  LYS A 500      19.991  -3.991  47.712  1.00 29.69           C
ATOM   3940  NZ  LYS A 500      18.606  -3.820  48.316  1.00 30.96           N1+
ATOM   3941  N   GLU A 501      24.456  -3.494  43.144  1.00 28.36           N
ATOM   3942  CA  GLU A 501      25.733  -3.020  42.626  1.00 27.10           C
ATOM   3943  C   GLU A 501      26.219  -1.775  43.365  1.00 23.87           C
ATOM   3944  O   GLU A 501      25.440  -0.854  43.611  1.00 24.20           O
ATOM   3945  CB  GLU A 501      25.583  -2.722  41.120  1.00 28.81           C
ATOM   3946  CG  GLU A 501      26.853  -2.919  40.279  1.00 27.95           C
ATOM   3947  CD  GLU A 501      26.979  -4.325  39.717  1.00 26.77           C
ATOM   3948  OE1 GLU A 501      25.944  -4.926  39.354  1.00 26.88           O
ATOM   3949  OE2 GLU A 501      28.114  -4.842  39.661  1.00 27.69           O1-
ATOM   3950  N   PHE A 502      27.466  -1.790  43.799  1.00 19.52           N
ATOM   3951  CA  PHE A 502      28.068  -0.583  44.320  1.00 21.84           C
ATOM   3952  C   PHE A 502      29.008   0.086  43.277  1.00 22.82           C
ATOM   3953  O   PHE A 502      29.712  -0.598  42.551  1.00 24.52           O
ATOM   3954  CB  PHE A 502      28.827  -0.921  45.617  1.00 22.57           C
ATOM   3955  CG  PHE A 502      28.007  -0.747  46.897  1.00 24.13           C
ATOM   3956  CD1 PHE A 502      26.662  -0.372  46.856  1.00 20.81           C
ATOM   3957  CD2 PHE A 502      28.627  -0.839  48.149  1.00 22.46           C
ATOM   3958  CE1 PHE A 502      25.976  -0.085  48.030  1.00 19.06           C
ATOM   3959  CE2 PHE A 502      27.937  -0.544  49.329  1.00 18.64           C
ATOM   3960  CZ  PHE A 502      26.621  -0.165  49.266  1.00 19.92           C
ATOM   3961  N   ASN A 503      28.876   1.392  43.069  1.00 22.88           N
ATOM   3962  CA  ASN A 503      30.017   2.214  42.668  1.00 24.10           C
ATOM   3963  C   ASN A 503      30.509   2.157  41.188  1.00 25.87           C
ATOM   3964  O   ASN A 503      31.690   2.401  40.905  1.00 24.39           O
ATOM   3965  CB  ASN A 503      31.167   1.906  43.630  1.00 24.77           C
ATOM   3966  CG  ASN A 503      30.765   2.064  45.094  1.00 25.30           C
ATOM   3967  ND2 ASN A 503      31.270   1.181  45.961  1.00 25.44           N
ATOM   3968  OD1 ASN A 503      30.024   2.978  45.442  1.00 26.75           O
ATOM   3969  N   ALA A 504      29.564   2.196  40.254  1.00 28.88           N
ATOM   3970  CA  ALA A 504      29.804   1.734  38.874  1.00 31.13           C
ATOM   3971  C   ALA A 504      30.300   2.776  37.823  1.00 31.37           C
ATOM   3972  O   ALA A 504      29.549   3.108  36.885  1.00 29.77           O
ATOM   3973  CB  ALA A 504      28.522   1.003  38.351  1.00 29.20           C
```

ATOM	3974	N	GLU	A	505	31.615	3.064	37.847	1.00	31.12	N
ATOM	3975	CA	GLU	A	505	32.305	4.005	36.923	1.00	30.29	C
ATOM	3976	C	GLU	A	505	32.672	3.490	35.500	1.00	30.54	C
ATOM	3977	O	GLU	A	505	33.795	3.603	35.040	1.00	30.90	O
ATOM	3978	CB	GLU	A	505	33.555	4.579	37.623	1.00	29.84	C
ATOM	3979	CG	GLU	A	505	34.522	5.484	36.794	1.00	30.64	C
ATOM	3980	CD	GLU	A	505	33.845	6.636	36.067	1.00	30.76	C
ATOM	3981	OE1	GLU	A	505	32.604	6.713	36.126	1.00	33.58	O
ATOM	3982	OE2	GLU	A	505	34.546	7.445	35.405	1.00	27.70	O1-
ATOM	3983	N	THR	A	506	31.770	2.737	34.900	1.00	32.72	N
ATOM	3984	CA	THR	A	506	31.498	2.842	33.477	1.00	30.71	C
ATOM	3985	C	THR	A	506	30.144	3.518	33.584	1.00	30.02	C
ATOM	3986	O	THR	A	506	29.098	2.881	33.485	1.00	30.52	O
ATOM	3987	CB	THR	A	506	31.347	1.480	32.857	1.00	31.87	C
ATOM	3988	CG2	THR	A	506	30.724	1.603	31.495	1.00	32.08	C
ATOM	3989	OG1	THR	A	506	32.644	0.859	32.776	1.00	33.97	O
ATOM	3990	N	PHE	A	507	30.249	4.657	34.251	1.00	28.98	N
ATOM	3991	CA	PHE	A	507	29.169	5.342	34.917	1.00	26.92	C
ATOM	3992	C	PHE	A	507	29.075	6.625	34.106	1.00	25.90	C
ATOM	3993	O	PHE	A	507	28.730	6.586	32.917	1.00	22.64	O
ATOM	3994	CB	PHE	A	507	29.648	5.661	36.330	1.00	27.01	C
ATOM	3995	CG	PHE	A	507	28.600	6.192	37.248	1.00	28.40	C
ATOM	3996	CD1	PHE	A	507	27.527	6.923	36.778	1.00	26.26	C
ATOM	3997	CD2	PHE	A	507	28.789	6.093	38.622	1.00	28.34	C
ATOM	3998	CE1	PHE	A	507	26.673	7.551	37.666	1.00	28.02	C
ATOM	3999	CE2	PHE	A	507	27.934	6.718	39.511	1.00	26.64	C
ATOM	4000	CZ	PHE	A	507	26.882	7.446	39.035	1.00	26.34	C
ATOM	4001	N	THR	A	508	29.795	7.615	34.624	1.00	23.79	N
ATOM	4002	CA	THR	A	508	29.605	9.007	34.270	1.00	25.58	C
ATOM	4003	C	THR	A	508	30.579	9.372	33.153	1.00	24.27	C
ATOM	4004	O	THR	A	508	31.651	9.945	33.379	1.00	19.34	O
ATOM	4005	CB	THR	A	508	29.824	9.898	35.522	1.00	27.81	C
ATOM	4006	CG2	THR	A	508	29.006	11.184	35.427	1.00	27.64	C
ATOM	4007	OG1	THR	A	508	29.411	9.172	36.692	1.00	27.38	O
ATOM	4008	N	PHE	A	509	30.175	9.076	31.927	1.00	26.09	N
ATOM	4009	CA	PHE	A	509	31.089	9.209	30.800	1.00	29.27	C
ATOM	4010	C	PHE	A	509	31.562	10.649	30.558	1.00	29.37	C
ATOM	4011	O	PHE	A	509	32.470	10.857	29.742	1.00	29.22	O
ATOM	4012	CB	PHE	A	509	30.475	8.578	29.519	1.00	28.61	C
ATOM	4013	CG	PHE	A	509	29.409	9.410	28.848	1.00	30.39	C
ATOM	4014	CD1	PHE	A	509	29.751	10.520	28.076	1.00	31.31	C
ATOM	4015	CD2	PHE	A	509	28.107	8.935	28.764	1.00	31.82	C
ATOM	4016	CE1	PHE	A	509	28.831	11.131	27.223	1.00	29.90	C
ATOM	4017	CE2	PHE	A	509	27.170	9.543	27.906	1.00	31.41	C
ATOM	4018	CZ	PHE	A	509	27.541	10.639	27.136	1.00	31.04	C
ATOM	4019	N	HIS	A	510	31.160	11.554	31.466	1.00	28.52	N
ATOM	4020	CA	HIS	A	510	31.106	13.001	31.228	1.00	26.73	C
ATOM	4021	C	HIS	A	510	31.430	13.363	29.766	1.00	27.36	C
ATOM	4022	O	HIS	A	510	30.619	13.094	28.892	1.00	27.39	O
ATOM	4023	CB	HIS	A	510	32.021	13.738	32.212	1.00	24.61	C
ATOM	4024	CG	HIS	A	510	32.016	15.224	32.042	1.00	23.20	C
ATOM	4025	CD2	HIS	A	510	31.108	16.156	32.409	1.00	19.13	C
ATOM	4026	ND1	HIS	A	510	32.998	15.900	31.348	1.00	23.83	N
ATOM	4027	CE1	HIS	A	510	32.685	17.177	31.279	1.00	20.37	C
ATOM	4028	NE2	HIS	A	510	31.546	17.360	31.923	1.00	18.67	N
ATOM	4029	N	ALA	A	511	32.650	13.831	29.490	1.00	26.47	N
ATOM	4030	CA	ALA	A	511	33.110	14.040	28.119	1.00	26.06	C
ATOM	4031	C	ALA	A	511	32.517	15.279	27.427	1.00	28.02	C
ATOM	4032	O	ALA	A	511	32.011	15.182	26.314	1.00	30.20	O
ATOM	4033	CB	ALA	A	511	32.831	12.774	27.276	1.00	22.01	C
ATOM	4034	N	ASP	A	512	32.665	16.458	28.031	1.00	27.66	N
ATOM	4035	CA	ASP	A	512	31.940	17.650	27.558	1.00	26.35	C
ATOM	4036	C	ASP	A	512	30.605	17.315	26.859	1.00	25.46	C
ATOM	4037	O	ASP	A	512	30.404	17.662	25.687	1.00	22.46	O
ATOM	4038	CB	ASP	A	512	32.822	18.475	26.622	1.00	28.10	C
ATOM	4039	CG	ASP	A	512	32.789	19.984	26.933	1.00	30.49	C
ATOM	4040	OD1	ASP	A	512	32.547	20.373	28.103	1.00	27.50	O
ATOM	4041	OD2	ASP	A	512	33.113	20.783	26.011	1.00	31.67	O1-
ATOM	4042	N	ILE	A	513	29.781	16.505	27.545	1.00	24.03	N
ATOM	4043	CA	ILE	A	513	28.389	16.258	27.174	1.00	18.35	C
ATOM	4044	C	ILE	A	513	27.730	17.571	26.913	1.00	19.35	C

ATOM	4045	O	ILE	A	513	26.907	17.671	26.015	1.00	19.34	O
ATOM	4046	CB	ILE	A	513	27.557	15.616	28.278	1.00	16.43	C
ATOM	4047	CG1	ILE	A	513	28.316	14.488	28.959	1.00	16.18	C
ATOM	4048	CG2	ILE	A	513	26.270	15.102	27.670	1.00	18.39	C
ATOM	4049	CD1	ILE	A	513	27.473	13.550	29.809	1.00	14.73	C
ATOM	4050	N	CYS	A	514	27.917	18.494	27.857	1.00	19.00	N
ATOM	4051	CA	CYS	A	514	27.567	19.896	27.650	1.00	20.07	C
ATOM	4052	C	CYS	A	514	28.126	20.289	26.300	1.00	22.50	C
ATOM	4053	O	CYS	A	514	29.334	20.276	26.095	1.00	23.48	O
ATOM	4054	CB	CYS	A	514	28.217	20.768	28.708	1.00	17.67	C
ATOM	4055	SG	CYS	A	514	30.001	20.505	28.723	1.00	18.01	S
ATOM	4056	N	THR	A	515	27.228	20.406	25.335	1.00	25.46	N
ATOM	4057	CA	THR	A	515	27.580	20.748	23.970	1.00	26.86	C
ATOM	4058	C	THR	A	515	28.699	19.896	23.361	1.00	26.41	C
ATOM	4059	O	THR	A	515	29.493	20.391	22.563	1.00	28.04	O
ATOM	4060	CB	THR	A	515	27.885	22.263	23.827	1.00	28.89	C
ATOM	4061	CG2	THR	A	515	26.576	23.040	23.525	1.00	29.83	C
ATOM	4062	OG1	THR	A	515	28.464	22.767	25.042	1.00	32.17	O
ATOM	4063	N	LEU	A	516	28.791	18.639	23.804	1.00	22.97	N
ATOM	4064	CA	LEU	A	516	28.855	17.500	22.881	1.00	20.76	C
ATOM	4065	C	LEU	A	516	27.510	17.536	22.152	1.00	20.35	C
ATOM	4066	O	LEU	A	516	27.427	17.980	21.001	1.00	19.65	O
ATOM	4067	CB	LEU	A	516	28.905	16.167	23.656	1.00	18.70	C
ATOM	4068	CG	LEU	A	516	30.129	15.387	24.146	1.00	11.26	C
ATOM	4069	CD1	LEU	A	516	29.858	13.895	24.061	1.00	11.26	C
ATOM	4070	CD2	LEU	A	516	31.309	15.729	23.330	1.00	14.12	C
ATOM	4071	N	SER	A	517	26.489	17.504	23.015	1.00	21.27	N
ATOM	4072	CA	SER	A	517	25.113	17.099	22.728	1.00	18.57	C
ATOM	4073	C	SER	A	517	24.157	18.259	22.953	1.00	17.16	C
ATOM	4074	O	SER	A	517	24.307	18.999	23.915	1.00	14.00	O
ATOM	4075	CB	SER	A	517	24.713	15.963	23.680	1.00	20.93	C
ATOM	4076	OG	SER	A	517	24.982	16.296	25.045	1.00	16.97	O
ATOM	4077	N	GLU	A	518	23.085	18.293	22.169	1.00	18.23	N
ATOM	4078	CA	GLU	A	518	22.162	19.418	22.155	1.00	19.60	C
ATOM	4079	C	GLU	A	518	20.713	18.940	22.104	1.00	19.98	C
ATOM	4080	O	GLU	A	518	20.437	17.817	21.692	1.00	18.87	O
ATOM	4081	CB	GLU	A	518	22.443	20.318	20.947	1.00	22.90	C
ATOM	4082	CG	GLU	A	518	23.891	20.813	20.811	1.00	25.02	C
ATOM	4083	CD	GLU	A	518	23.986	22.237	20.281	1.00	22.60	C
ATOM	4084	OE1	GLU	A	518	23.615	22.475	19.110	1.00	20.70	O
ATOM	4085	OE2	GLU	A	518	24.388	23.126	21.061	1.00	23.36	O1-
ATOM	4086	N	LYS	A	519	19.797	19.884	22.314	1.00	23.54	N
ATOM	4087	CA	LYS	A	519	18.371	19.614	22.551	1.00	24.75	C
ATOM	4088	C	LYS	A	519	18.200	18.755	23.804	1.00	27.97	C
ATOM	4089	O	LYS	A	519	18.733	17.638	23.900	1.00	28.69	O
ATOM	4090	CB	LYS	A	519	17.732	18.927	21.346	1.00	21.56	C
ATOM	4091	CG	LYS	A	519	16.276	18.526	21.524	1.00	16.97	C
ATOM	4092	CD	LYS	A	519	15.692	18.116	20.174	1.00	17.59	C
ATOM	4093	CE	LYS	A	519	14.341	17.430	20.287	1.00	18.48	C
ATOM	4094	NZ	LYS	A	519	13.728	17.036	18.962	1.00	18.54	N1+
ATOM	4095	N	GLU	A	520	17.552	19.324	24.817	1.00	28.25	N
ATOM	4096	CA	GLU	A	520	17.420	18.641	26.099	1.00	27.40	C
ATOM	4097	C	GLU	A	520	16.947	17.204	25.945	1.00	29.51	C
ATOM	4098	O	GLU	A	520	17.010	16.423	26.886	1.00	31.44	O
ATOM	4099	CB	GLU	A	520	16.459	19.417	26.980	1.00	22.39	C
ATOM	4100	CG	GLU	A	520	16.902	20.824	27.137	1.00	17.69	C
ATOM	4101	CD	GLU	A	520	18.391	20.924	27.333	1.00	16.28	C
ATOM	4102	OE1	GLU	A	520	18.877	20.613	28.429	1.00	17.87	O
ATOM	4103	OE2	GLU	A	520	19.084	21.272	26.372	1.00	17.14	O1-
ATOM	4104	N	ARG	A	521	16.406	16.883	24.775	1.00	32.04	N
ATOM	4105	CA	ARG	A	521	16.024	15.522	24.475	1.00	33.58	C
ATOM	4106	C	ARG	A	521	17.287	14.687	24.495	1.00	33.32	C
ATOM	4107	O	ARG	A	521	17.341	13.664	25.185	1.00	35.16	O
ATOM	4108	CB	ARG	A	521	15.298	15.456	23.131	1.00	34.23	C
ATOM	4109	CG	ARG	A	521	13.950	16.232	23.131	1.00	35.49	C
ATOM	4110	CD	ARG	A	521	12.801	15.429	23.747	1.00	38.31	C
ATOM	4111	NE	ARG	A	521	12.787	14.036	23.296	1.00	39.41	N
ATOM	4112	CZ	ARG	A	521	12.362	13.650	22.094	1.00	40.68	C
ATOM	4113	NH1	ARG	A	521	11.733	14.513	21.308	1.00	43.68	N1+
ATOM	4114	NH2	ARG	A	521	12.479	12.384	21.712	1.00	40.15	N
ATOM	4115	N	GLN	A	522	18.369	15.302	24.032	1.00	30.52	N

ATOM	4116	CA	GLN	A	522	19.687	14.689	24.103	1.00	25.95	C
ATOM	4117	C	GLN	A	522	20.450	15.095	25.345	1.00	20.74	C
ATOM	4118	O	GLN	A	522	21.400	14.411	25.727	1.00	15.80	O
ATOM	4119	CB	GLN	A	522	20.515	15.047	22.867	1.00	26.11	C
ATOM	4120	CG	GLN	A	522	19.902	14.640	21.555	1.00	26.14	C
ATOM	4121	CD	GLN	A	522	20.623	15.273	20.397	1.00	28.67	C
ATOM	4122	NE2	GLN	A	522	19.963	15.346	19.241	1.00	26.87	N
ATOM	4123	OE1	GLN	A	522	21.777	15.681	20.533	1.00	27.66	O
ATOM	4124	N	ILE	A	523	20.145	16.264	25.897	1.00	18.75	N
ATOM	4125	CA	ILE	A	523	20.906	16.687	27.069	1.00	19.51	C
ATOM	4126	C	ILE	A	523	20.389	15.978	28.312	1.00	19.06	C
ATOM	4127	O	ILE	A	523	21.156	15.338	29.050	1.00	19.63	O
ATOM	4128	CB	ILE	A	523	20.862	18.206	27.321	1.00	17.88	C
ATOM	4129	CG1	ILE	A	523	20.994	18.975	26.004	1.00	21.59	C
ATOM	4130	CG2	ILE	A	523	21.993	18.578	28.263	1.00	18.05	C
ATOM	4131	CD1	ILE	A	523	21.840	20.265	26.080	1.00	21.79	C
ATOM	4132	N	LYS	A	524	19.076	16.059	28.512	1.00	16.03	N
ATOM	4133	CA	LYS	A	524	18.464	15.493	29.680	1.00	12.94	C
ATOM	4134	C	LYS	A	524	18.691	13.989	29.649	1.00	13.61	C
ATOM	4135	O	LYS	A	524	19.531	13.510	30.385	1.00	13.89	O
ATOM	4136	CB	LYS	A	524	16.986	15.858	29.752	1.00	10.56	C
ATOM	4137	CG	LYS	A	524	16.375	15.511	31.100	1.00	15.61	C
ATOM	4138	CD	LYS	A	524	15.266	16.457	31.565	1.00	13.48	C
ATOM	4139	CE	LYS	A	524	14.946	16.213	33.033	1.00	11.44	C
ATOM	4140	NZ	LYS	A	524	16.147	16.413	33.888	1.00	5.17	N1+
ATOM	4141	N	LYS	A	525	18.184	13.312	28.625	1.00	14.90	N
ATOM	4142	CA	LYS	A	525	18.299	11.851	28.506	1.00	16.22	C
ATOM	4143	C	LYS	A	525	19.550	11.230	29.180	1.00	17.48	C
ATOM	4144	O	LYS	A	525	19.457	10.186	29.837	1.00	16.17	O
ATOM	4145	CB	LYS	A	525	18.212	11.450	27.014	1.00	13.21	C
ATOM	4146	CG	LYS	A	525	18.854	10.133	26.631	1.00	10.66	C
ATOM	4147	CD	LYS	A	525	20.084	10.378	25.775	1.00	15.61	C
ATOM	4148	CE	LYS	A	525	20.840	9.078	25.401	1.00	17.64	C
ATOM	4149	NZ	LYS	A	525	21.001	8.890	23.909	1.00	14.96	N1+
ATOM	4150	N	GLN	A	526	20.707	11.881	29.040	1.00	18.82	N
ATOM	4151	CA	GLN	A	526	21.912	11.429	29.719	1.00	18.67	C
ATOM	4152	C	GLN	A	526	21.750	11.834	31.156	1.00	20.95	C
ATOM	4153	O	GLN	A	526	21.540	10.964	32.010	1.00	21.14	O
ATOM	4154	CB	GLN	A	526	23.178	12.073	29.151	1.00	18.08	C
ATOM	4155	CG	GLN	A	526	23.862	11.294	28.030	1.00	17.91	C
ATOM	4156	CD	GLN	A	526	23.946	9.804	28.298	1.00	20.28	C
ATOM	4157	NE2	GLN	A	526	23.552	9.007	27.320	1.00	22.11	N
ATOM	4158	OE1	GLN	A	526	24.427	9.376	29.339	1.00	22.06	O
ATOM	4159	N	THR	A	527	21.660	13.145	31.398	1.00	21.86	N
ATOM	4160	CA	THR	A	527	21.391	13.654	32.749	1.00	24.95	C
ATOM	4161	C	THR	A	527	20.378	12.766	33.490	1.00	25.85	C
ATOM	4162	O	THR	A	527	20.595	12.424	34.646	1.00	27.72	O
ATOM	4163	CB	THR	A	527	20.843	15.103	32.733	1.00	25.26	C
ATOM	4164	CG2	THR	A	527	21.009	15.752	34.105	1.00	23.79	C
ATOM	4165	OG1	THR	A	527	21.544	15.880	31.752	1.00	24.22	O
ATOM	4166	N	ALA	A	528	19.375	12.254	32.771	1.00	27.33	N
ATOM	4167	CA	ALA	A	528	18.426	11.281	33.333	1.00	26.81	C
ATOM	4168	C	ALA	A	528	18.996	9.857	33.369	1.00	25.61	C
ATOM	4169	O	ALA	A	528	18.965	9.204	34.412	1.00	25.23	O
ATOM	4170	CB	ALA	A	528	17.100	11.306	32.562	1.00	25.49	C
ATOM	4171	N	LEU	A	529	19.654	9.438	32.296	1.00	24.25	N
ATOM	4172	CA	LEU	A	529	20.315	8.139	32.279	1.00	25.05	C
ATOM	4173	C	LEU	A	529	21.267	7.996	33.493	1.00	25.41	C
ATOM	4174	O	LEU	A	529	21.762	6.891	33.797	1.00	25.16	O
ATOM	4175	CB	LEU	A	529	21.086	7.959	30.955	1.00	25.66	C
ATOM	4176	CG	LEU	A	529	20.727	6.802	30.000	1.00	24.04	C
ATOM	4177	CD1	LEU	A	529	20.160	7.352	28.710	1.00	19.96	C
ATOM	4178	CD2	LEU	A	529	21.952	5.961	29.702	1.00	21.68	C
ATOM	4179	N	VAL	A	530	21.523	9.116	34.175	1.00	24.26	N
ATOM	4180	CA	VAL	A	530	22.246	9.102	35.440	1.00	22.74	C
ATOM	4181	C	VAL	A	530	21.369	8.479	36.518	1.00	23.56	C
ATOM	4182	O	VAL	A	530	21.867	7.708	37.360	1.00	22.76	O
ATOM	4183	CB	VAL	A	530	22.618	10.525	35.896	1.00	23.03	C
ATOM	4184	CG1	VAL	A	530	23.610	10.471	37.036	1.00	24.30	C
ATOM	4185	CG2	VAL	A	530	23.200	11.305	34.754	1.00	22.73	C
ATOM	4186	N	GLU	A	531	20.076	8.831	36.502	1.00	22.46	N

ATOM	4187	CA	GLU	A	531	19.139	8.472	37.575	1.00	23.18	C
ATOM	4188	C	GLU	A	531	19.096	7.000	37.982	1.00	24.30	C
ATOM	4189	O	GLU	A	531	19.552	6.668	39.074	1.00	28.02	O
ATOM	4190	CB	GLU	A	531	17.728	8.959	37.262	1.00	22.28	C
ATOM	4191	CG	GLU	A	531	16.791	8.825	38.456	1.00	21.12	C
ATOM	4192	CD	GLU	A	531	16.177	10.137	38.917	1.00	19.64	C
ATOM	4193	OE1	GLU	A	531	16.850	10.917	39.623	1.00	18.65	O
ATOM	4194	OE2	GLU	A	531	14.977	10.337	38.671	1.00	18.88	O1-
ATOM	4195	N	LEU	A	532	18.603	6.115	37.114	1.00	23.63	N
ATOM	4196	CA	LEU	A	532	18.693	4.662	37.362	1.00	24.00	C
ATOM	4197	C	LEU	A	532	19.972	4.240	38.078	1.00	24.04	C
ATOM	4198	O	LEU	A	532	19.907	3.770	39.211	1.00	26.15	O
ATOM	4199	CB	LEU	A	532	18.604	3.854	36.071	1.00	22.16	C
ATOM	4200	CG	LEU	A	532	17.991	4.619	34.926	1.00	23.10	C
ATOM	4201	CD1	LEU	A	532	19.075	5.386	34.246	1.00	28.26	C
ATOM	4202	CD2	LEU	A	532	17.348	3.681	33.973	1.00	27.82	C
ATOM	4203	N	VAL	A	533	21.131	4.440	37.442	1.00	22.79	N
ATOM	4204	CA	VAL	A	533	22.397	3.925	37.974	1.00	19.76	C
ATOM	4205	C	VAL	A	533	22.905	4.700	39.179	1.00	18.89	C
ATOM	4206	O	VAL	A	533	23.874	4.302	39.806	1.00	19.08	O
ATOM	4207	CB	VAL	A	533	23.521	3.852	36.889	1.00	18.59	C
ATOM	4208	CG1	VAL	A	533	23.308	2.624	35.996	1.00	15.07	C
ATOM	4209	CG2	VAL	A	533	23.560	5.142	36.045	1.00	20.41	C
ATOM	4210	N	LYS	A	534	22.243	5.792	39.530	1.00	19.45	N
ATOM	4211	CA	LYS	A	534	22.478	6.365	40.845	1.00	21.27	C
ATOM	4212	C	LYS	A	534	21.526	5.757	41.868	1.00	21.02	C
ATOM	4213	O	LYS	A	534	21.531	6.167	43.023	1.00	20.41	O
ATOM	4214	CB	LYS	A	534	22.357	7.898	40.818	1.00	22.77	C
ATOM	4215	CG	LYS	A	534	20.941	8.444	40.696	1.00	23.98	C
ATOM	4216	CD	LYS	A	534	20.290	8.641	42.054	1.00	28.34	C
ATOM	4217	CE	LYS	A	534	18.928	7.988	42.095	1.00	26.73	C
ATOM	4218	NZ	LYS	A	534	18.037	8.601	41.075	1.00	25.87	N1+
ATOM	4219	N	HIS	A	535	20.627	4.879	41.418	1.00	21.35	N
ATOM	4220	CA	HIS	A	535	19.932	3.950	42.323	1.00	20.99	C
ATOM	4221	C	HIS	A	535	19.602	2.550	41.769	1.00	22.37	C
ATOM	4222	O	HIS	A	535	18.524	2.290	41.214	1.00	22.53	O
ATOM	4223	CB	HIS	A	535	18.722	4.628	42.999	1.00	20.28	C
ATOM	4224	CG	HIS	A	535	17.424	4.489	42.272	1.00	16.66	C
ATOM	4225	CD2	HIS	A	535	17.111	4.603	40.961	1.00	19.33	C
ATOM	4226	ND1	HIS	A	535	16.227	4.401	42.941	1.00	14.54	N
ATOM	4227	CE1	HIS	A	535	15.235	4.488	42.081	1.00	19.05	C
ATOM	4228	NE2	HIS	A	535	15.743	4.611	40.868	1.00	19.23	N
ATOM	4229	N	LYS	A	536	20.476	1.632	42.174	1.00	22.18	N
ATOM	4230	CA	LYS	A	536	20.778	0.332	41.553	1.00	23.44	C
ATOM	4231	C	LYS	A	536	22.184	0.035	42.107	1.00	26.46	C
ATOM	4232	O	LYS	A	536	22.757	-1.031	41.833	1.00	24.60	O
ATOM	4233	CB	LYS	A	536	20.884	0.419	40.023	1.00	18.83	C
ATOM	4234	CG	LYS	A	536	19.572	0.375	39.276	1.00	14.87	C
ATOM	4235	CD	LYS	A	536	19.778	0.539	37.773	1.00	12.15	C
ATOM	4236	CE	LYS	A	536	20.178	-0.774	37.107	1.00	12.18	C
ATOM	4237	NZ	LYS	A	536	19.620	-0.875	35.727	1.00	11.32	N1+
ATOM	4238	N	PRO	A	537	22.840	1.084	42.670	1.00	28.07	N
ATOM	4239	CA	PRO	A	537	23.264	1.257	44.063	1.00	27.56	C
ATOM	4240	C	PRO	A	537	22.139	1.885	44.911	1.00	26.87	C
ATOM	4241	O	PRO	A	537	21.305	2.611	44.393	1.00	26.05	O
ATOM	4242	CB	PRO	A	537	24.475	2.181	43.918	1.00	26.82	C
ATOM	4243	CG	PRO	A	537	24.086	3.095	42.877	1.00	29.18	C
ATOM	4244	CD	PRO	A	537	23.285	2.244	41.868	1.00	30.49	C
ATOM	4245	N	LYS	A	538	22.119	1.587	46.205	1.00	28.13	N
ATOM	4246	CA	LYS	A	538	21.009	1.933	47.109	1.00	27.98	C
ATOM	4247	C	LYS	A	538	19.630	2.015	46.460	1.00	26.40	C
ATOM	4248	O	LYS	A	538	19.218	3.072	45.974	1.00	24.43	O
ATOM	4249	CB	LYS	A	538	21.297	3.229	47.883	1.00	29.62	C
ATOM	4250	CG	LYS	A	538	21.027	3.139	49.423	1.00	25.31	C
ATOM	4251	CD	LYS	A	538	22.012	2.197	50.131	1.00	24.14	C
ATOM	4252	CE	LYS	A	538	21.945	2.359	51.647	1.00	23.68	C
ATOM	4253	NZ	LYS	A	538	23.277	2.474	52.317	1.00	18.75	N1+
ATOM	4254	N	ALA	A	539	18.869	0.938	46.625	1.00	24.41	N
ATOM	4255	CA	ALA	A	539	17.672	0.728	45.840	1.00	24.62	C
ATOM	4256	C	ALA	A	539	16.793	1.958	45.958	1.00	23.58	C
ATOM	4257	O	ALA	A	539	16.534	2.635	44.963	1.00	22.67	O

ATOM	4258	CB	ALA	A	539	16.943	-0.511	46.325	1.00	25.28	C
ATOM	4259	N	THR	A	540	16.559	2.380	47.195	1.00	23.77	N
ATOM	4260	CA	THR	A	540	15.772	3.585	47.440	1.00	23.68	C
ATOM	4261	C	THR	A	540	16.133	4.351	48.713	1.00	25.34	C
ATOM	4262	O	THR	A	540	16.602	3.772	49.709	1.00	23.64	O
ATOM	4263	CB	THR	A	540	14.275	3.272	47.461	1.00	21.40	C
ATOM	4264	CG2	THR	A	540	13.857	2.656	48.804	1.00	20.40	C
ATOM	4265	OG1	THR	A	540	13.546	4.479	47.220	1.00	20.73	O
ATOM	4266	N	LYS	A	541	15.942	5.666	48.654	1.00	26.45	N
ATOM	4267	CA	LYS	A	541	16.156	6.528	49.810	1.00	28.39	C
ATOM	4268	C	LYS	A	541	15.191	7.698	49.794	1.00	27.80	C
ATOM	4269	O	LYS	A	541	14.459	7.884	48.830	1.00	29.39	O
ATOM	4270	CB	LYS	A	541	17.608	7.027	49.847	1.00	29.26	C
ATOM	4271	CG	LYS	A	541	18.614	5.982	50.350	1.00	25.06	C
ATOM	4272	CD	LYS	A	541	18.582	5.886	51.869	1.00	20.48	C
ATOM	4273	CE	LYS	A	541	19.335	7.030	52.505	1.00	17.85	C
ATOM	4274	NZ	LYS	A	541	20.734	7.139	52.022	1.00	16.81	N1+
ATOM	4275	N	GLU	A	542	15.016	8.318	50.952	1.00	29.97	N
ATOM	4276	CA	GLU	A	542	14.101	9.453	51.086	1.00	31.00	C
ATOM	4277	C	GLU	A	542	14.840	10.660	50.478	1.00	30.24	C
ATOM	4278	O	GLU	A	542	15.814	10.456	49.756	1.00	29.44	O
ATOM	4279	CB	GLU	A	542	13.755	9.669	52.582	1.00	29.69	C
ATOM	4280	CG	GLU	A	542	12.602	10.630	52.878	1.00	26.76	C
ATOM	4281	CD	GLU	A	542	11.289	10.221	52.240	1.00	26.79	C
ATOM	4282	OE1	GLU	A	542	11.093	9.010	51.995	1.00	25.61	O
ATOM	4283	OE2	GLU	A	542	10.425	11.112	52.035	1.00	25.89	O1-
ATOM	4284	N	GLN	A	543	14.538	11.862	50.981	1.00	30.95	N
ATOM	4285	CA	GLN	A	543	15.020	13.157	50.451	1.00	29.95	C
ATOM	4286	C	GLN	A	543	16.307	13.073	49.606	1.00	29.03	C
ATOM	4287	O	GLN	A	543	16.294	13.347	48.389	1.00	25.49	O
ATOM	4288	CB	GLN	A	543	15.208	14.161	51.624	1.00	26.87	C
ATOM	4289	CG	GLN	A	543	16.249	13.747	52.684	1.00	21.98	C
ATOM	4290	CD	GLN	A	543	15.650	13.140	53.962	1.00	21.18	C
ATOM	4291	NE2	GLN	A	543	16.336	13.335	55.077	1.00	20.31	N
ATOM	4292	OE1	GLN	A	543	14.613	12.479	53.939	1.00	20.60	O
ATOM	4293	N	LEU	A	544	17.307	12.444	50.225	1.00	26.45	N
ATOM	4294	CA	LEU	A	544	18.653	12.279	49.694	1.00	24.19	C
ATOM	4295	C	LEU	A	544	18.793	12.637	48.228	1.00	22.92	C
ATOM	4296	O	LEU	A	544	19.185	13.758	47.923	1.00	23.86	O
ATOM	4297	CB	LEU	A	544	19.140	10.841	49.914	1.00	21.09	C
ATOM	4298	CG	LEU	A	544	19.186	10.233	51.311	1.00	16.44	C
ATOM	4299	CD1	LEU	A	544	19.940	11.158	52.243	1.00	16.15	C
ATOM	4300	CD2	LEU	A	544	17.789	9.967	51.819	1.00	16.66	C
ATOM	4301	N	LYS	A	545	18.194	11.817	47.362	1.00	20.06	N
ATOM	4302	CA	LYS	A	545	18.749	11.587	46.040	1.00	20.52	C
ATOM	4303	C	LYS	A	545	18.614	12.701	45.002	1.00	22.77	C
ATOM	4304	O	LYS	A	545	18.889	12.471	43.832	1.00	25.57	O
ATOM	4305	CB	LYS	A	545	18.248	10.254	45.461	1.00	18.18	C
ATOM	4306	CG	LYS	A	545	16.821	10.244	44.916	1.00	17.26	C
ATOM	4307	CD	LYS	A	545	15.906	9.289	45.696	1.00	12.74	C
ATOM	4308	CE	LYS	A	545	16.474	7.882	45.800	1.00	8.79	C
ATOM	4309	NZ	LYS	A	545	17.238	7.691	47.056	1.00	6.18	N1+
ATOM	4310	N	ALA	A	546	18.410	13.938	45.439	1.00	23.50	N
ATOM	4311	CA	ALA	A	546	18.556	15.071	44.535	1.00	22.32	C
ATOM	4312	C	ALA	A	546	19.741	15.954	44.903	1.00	22.35	C
ATOM	4313	O	ALA	A	546	20.086	16.854	44.156	1.00	23.42	O
ATOM	4314	CB	ALA	A	546	17.277	15.899	44.490	1.00	23.51	C
ATOM	4315	N	VAL	A	547	20.379	15.694	46.036	1.00	22.24	N
ATOM	4316	CA	VAL	A	547	21.717	16.229	46.279	1.00	23.87	C
ATOM	4317	C	VAL	A	547	22.677	15.472	45.360	1.00	23.89	C
ATOM	4318	O	VAL	A	547	23.877	15.782	45.287	1.00	23.60	O
ATOM	4319	CB	VAL	A	547	22.170	16.040	47.768	1.00	25.64	C
ATOM	4320	CG1	VAL	A	547	22.400	14.566	48.059	1.00	25.82	C
ATOM	4321	CG2	VAL	A	547	23.449	16.861	48.073	1.00	24.16	C
ATOM	4322	N	MET	A	548	22.132	14.475	44.661	1.00	22.84	N
ATOM	4323	CA	MET	A	548	22.841	13.763	43.593	1.00	20.52	C
ATOM	4324	C	MET	A	548	22.779	14.480	42.239	1.00	17.26	C
ATOM	4325	O	MET	A	548	23.748	15.082	41.805	1.00	17.45	O
ATOM	4326	CB	MET	A	548	22.268	12.353	43.445	1.00	21.64	C
ATOM	4327	CG	MET	A	548	22.122	11.605	44.756	1.00	18.22	C
ATOM	4328	SD	MET	A	548	23.630	10.850	45.228	1.00	14.88	S

ATOM	4329	CE	MET	A	548	24.207	12.056	46.438	1.00	16.13	C
ATOM	4330	N	ASP	A	549	21.618	14.485	41.609	1.00	13.78	N
ATOM	4331	CA	ASP	A	549	21.502	15.018	40.264	1.00	16.01	C
ATOM	4332	C	ASP	A	549	21.636	16.528	40.156	1.00	18.51	C
ATOM	4333	O	ASP	A	549	21.000	17.147	39.300	1.00	20.40	O
ATOM	4334	CB	ASP	A	549	20.174	14.604	39.649	1.00	14.29	C
ATOM	4335	CG	ASP	A	549	20.323	13.496	38.677	1.00	14.70	C
ATOM	4336	OD1	ASP	A	549	21.204	12.641	38.891	1.00	11.87	O
ATOM	4337	OD2	ASP	A	549	19.566	13.495	37.687	1.00	17.11	O1-
ATOM	4338	N	ASP	A	550	22.330	17.136	41.102	1.00	19.81	N
ATOM	4339	CA	ASP	A	550	22.847	18.486	40.917	1.00	25.24	C
ATOM	4340	C	ASP	A	550	24.372	18.383	41.093	1.00	27.84	C
ATOM	4341	O	ASP	A	550	25.143	18.785	40.213	1.00	29.39	O
ATOM	4342	CB	ASP	A	550	22.214	19.460	41.935	1.00	24.68	C
ATOM	4343	CG	ASP	A	550	20.805	19.913	41.527	1.00	25.32	C
ATOM	4344	OD1	ASP	A	550	20.168	19.239	40.694	1.00	26.62	O
ATOM	4345	OD2	ASP	A	550	20.334	20.971	42.003	1.00	23.45	O1-
ATOM	4346	N	PHE	A	551	24.778	17.554	42.046	1.00	29.10	N
ATOM	4347	CA	PHE	A	551	26.143	17.056	42.061	1.00	29.37	C
ATOM	4348	C	PHE	A	551	26.481	16.269	40.798	1.00	28.36	C
ATOM	4349	O	PHE	A	551	27.633	15.963	40.538	1.00	29.93	O
ATOM	4350	CB	PHE	A	551	26.361	16.227	43.324	1.00	30.56	C
ATOM	4351	CG	PHE	A	551	26.670	17.064	44.509	1.00	31.41	C
ATOM	4352	CD1	PHE	A	551	26.260	18.398	44.526	1.00	31.60	C
ATOM	4353	CD2	PHE	A	551	27.588	16.637	45.447	1.00	32.87	C
ATOM	4354	CE1	PHE	A	551	26.774	19.299	45.427	1.00	31.67	C
ATOM	4355	CE2	PHE	A	551	28.120	17.542	46.375	1.00	34.18	C
ATOM	4356	CZ	PHE	A	551	27.712	18.878	46.357	1.00	33.28	C
ATOM	4357	N	ALA	A	552	25.470	16.021	39.976	1.00	27.01	N
ATOM	4358	CA	ALA	A	552	25.676	15.608	38.605	1.00	25.37	C
ATOM	4359	C	ALA	A	552	24.735	16.431	37.719	1.00	25.73	C
ATOM	4360	O	ALA	A	552	23.980	15.910	36.886	1.00	24.39	O
ATOM	4361	CB	ALA	A	552	25.417	14.134	38.461	1.00	26.18	C
ATOM	4362	N	ALA	A	553	24.769	17.732	37.975	1.00	24.63	N
ATOM	4363	CA	ALA	A	553	24.274	18.746	37.068	1.00	22.98	C
ATOM	4364	C	ALA	A	553	25.328	19.824	37.002	1.00	25.17	C
ATOM	4365	O	ALA	A	553	25.138	20.792	36.306	1.00	26.82	O
ATOM	4366	CB	ALA	A	553	23.003	19.333	37.572	1.00	23.98	C
ATOM	4367	N	PHE	A	554	26.340	19.752	37.866	1.00	27.85	N
ATOM	4368	CA	PHE	A	554	27.586	20.505	37.668	1.00	28.64	C
ATOM	4369	C	PHE	A	554	28.412	19.808	36.596	1.00	25.54	C
ATOM	4370	O	PHE	A	554	29.324	20.398	36.026	1.00	24.77	O
ATOM	4371	CB	PHE	A	554	28.405	20.583	38.968	1.00	32.92	C
ATOM	4372	CG	PHE	A	554	28.806	21.997	39.366	1.00	37.67	C
ATOM	4373	CD1	PHE	A	554	28.374	23.110	38.618	1.00	39.00	C
ATOM	4374	CD2	PHE	A	554	29.534	22.220	40.544	1.00	37.65	C
ATOM	4375	CE1	PHE	A	554	28.648	24.436	39.051	1.00	39.21	C
ATOM	4376	CE2	PHE	A	554	29.818	23.532	40.984	1.00	40.34	C
ATOM	4377	CZ	PHE	A	554	29.369	24.647	40.233	1.00	39.54	C
ATOM	4378	N	VAL	A	555	28.081	18.543	36.352	1.00	23.93	N
ATOM	4379	CA	VAL	A	555	28.467	17.800	35.147	1.00	22.21	C
ATOM	4380	C	VAL	A	555	28.557	18.670	33.893	1.00	20.12	C
ATOM	4381	O	VAL	A	555	29.305	18.360	32.975	1.00	18.10	O
ATOM	4382	CB	VAL	A	555	27.484	16.602	34.897	1.00	21.30	C
ATOM	4383	CG1	VAL	A	555	26.074	17.094	34.820	1.00	22.10	C
ATOM	4384	CG2	VAL	A	555	27.815	15.870	33.632	1.00	22.52	C
ATOM	4385	N	GLU	A	556	27.829	19.772	33.855	1.00	20.36	N
ATOM	4386	CA	GLU	A	556	28.107	20.753	32.822	1.00	22.51	C
ATOM	4387	C	GLU	A	556	29.129	21.755	33.324	1.00	22.13	C
ATOM	4388	O	GLU	A	556	30.316	21.503	33.183	1.00	21.46	O
ATOM	4389	CB	GLU	A	556	26.827	21.440	32.333	1.00	24.42	C
ATOM	4390	CG	GLU	A	556	26.014	20.591	31.348	1.00	25.09	C
ATOM	4391	CD	GLU	A	556	25.397	19.340	32.002	1.00	29.89	C
ATOM	4392	OE1	GLU	A	556	26.157	18.391	32.313	1.00	28.92	O
ATOM	4393	OE2	GLU	A	556	24.150	19.300	32.192	1.00	30.78	O1-
ATOM	4394	N	LYS	A	557	28.693	22.721	34.130	1.00	23.25	N
ATOM	4395	CA	LYS	A	557	29.514	23.885	34.484	1.00	26.16	C
ATOM	4396	C	LYS	A	557	30.893	23.667	35.189	1.00	28.55	C
ATOM	4397	O	LYS	A	557	31.455	24.625	35.736	1.00	32.54	O
ATOM	4398	CB	LYS	A	557	28.688	24.876	35.319	1.00	25.61	C
ATOM	4399	CG	LYS	A	557	27.221	24.986	34.979	1.00	25.26	C

ATOM	4400	CD	LYS	A	557	26.383	25.468	36.212	1.00	26.93	C
ATOM	4401	CE	LYS	A	557	24.868	25.275	35.938	1.00	29.30	C
ATOM	4402	NZ	LYS	A	557	23.869	26.075	36.726	1.00	26.93	N1+
ATOM	4403	N	CYS	A	558	31.439	22.448	35.204	1.00	28.73	N
ATOM	4404	CA	CYS	A	558	32.899	22.266	35.342	1.00	26.69	C
ATOM	4405	C	CYS	A	558	33.460	21.735	34.016	1.00	26.16	C
ATOM	4406	O	CYS	A	558	34.575	21.193	33.962	1.00	25.82	O
ATOM	4407	CB	CYS	A	558	33.247	21.290	36.484	1.00	27.88	C
ATOM	4408	SG	CYS	A	558	34.126	19.745	35.984	1.00	25.90	S
ATOM	4409	N	CYS	A	559	32.702	21.924	32.939	1.00	24.28	N
ATOM	4410	CA	CYS	A	559	33.067	21.378	31.638	1.00	24.57	C
ATOM	4411	C	CYS	A	559	33.527	22.453	30.635	1.00	25.31	C
ATOM	4412	O	CYS	A	559	32.955	22.594	29.534	1.00	22.80	O
ATOM	4413	CB	CYS	A	559	31.882	20.610	31.057	1.00	24.33	C
ATOM	4414	SG	CYS	A	559	30.664	21.671	30.225	1.00	18.67	S
ATOM	4415	N	LYS	A	560	34.588	23.170	30.993	1.00	24.30	N
ATOM	4416	CA	LYS	A	560	35.196	24.124	30.082	1.00	24.61	C
ATOM	4417	C	LYS	A	560	36.487	24.703	30.634	1.00	24.70	C
ATOM	4418	O	LYS	A	560	36.873	24.417	31.765	1.00	22.58	O
ATOM	4419	CB	LYS	A	560	34.215	25.242	29.754	1.00	25.54	C
ATOM	4420	CG	LYS	A	560	33.656	25.152	28.339	1.00	26.31	C
ATOM	4421	CD	LYS	A	560	32.511	26.109	28.108	1.00	24.00	C
ATOM	4422	CE	LYS	A	560	31.495	25.480	27.188	1.00	25.24	C
ATOM	4423	NZ	LYS	A	560	31.446	24.014	27.391	1.00	26.72	N1+
ATOM	4424	N	ALA	A	561	37.126	25.555	29.841	1.00	24.25	N
ATOM	4425	CA	ALA	A	561	38.507	25.915	30.078	1.00	24.71	C
ATOM	4426	C	ALA	A	561	39.300	24.620	30.175	1.00	26.65	C
ATOM	4427	O	ALA	A	561	39.333	23.999	31.218	1.00	27.41	O
ATOM	4428	CB	ALA	A	561	38.622	26.707	31.359	1.00	25.16	C
ATOM	4429	N	ASP	A	562	39.642	24.065	29.016	1.00	30.60	N
ATOM	4430	CA	ASP	A	562	40.525	22.892	28.929	1.00	30.34	C
ATOM	4431	C	ASP	A	562	39.961	21.653	29.616	1.00	31.09	C
ATOM	4432	O	ASP	A	562	40.593	21.132	30.562	1.00	31.08	O
ATOM	4433	CB	ASP	A	562	41.905	23.198	29.516	1.00	28.16	C
ATOM	4434	CG	ASP	A	562	42.952	22.216	29.054	1.00	25.04	C
ATOM	4435	OD1	ASP	A	562	42.717	21.514	28.053	1.00	24.20	O
ATOM	4436	OD2	ASP	A	562	44.000	22.117	29.707	1.00	28.51	O1-
ATOM	4437	N	ASP	A	563	38.979	21.040	28.935	1.00	30.71	N
ATOM	4438	CA	ASP	A	563	38.059	20.068	29.538	1.00	28.76	C
ATOM	4439	C	ASP	A	563	38.485	18.610	29.373	1.00	28.00	C
ATOM	4440	O	ASP	A	563	39.612	18.256	29.693	1.00	28.65	O
ATOM	4441	CB	ASP	A	563	36.615	20.261	29.028	1.00	30.30	C
ATOM	4442	CG	ASP	A	563	36.508	21.125	27.746	1.00	33.01	C
ATOM	4443	OD1	ASP	A	563	37.154	22.206	27.640	1.00	32.70	O
ATOM	4444	OD2	ASP	A	563	35.634	20.803	26.908	1.00	31.15	O1-
ATOM	4445	N	LYS	A	564	37.563	17.749	28.966	1.00	29.53	N
ATOM	4446	CA	LYS	A	564	37.756	16.283	28.945	1.00	31.53	C
ATOM	4447	C	LYS	A	564	38.549	15.598	30.061	1.00	32.06	C
ATOM	4448	O	LYS	A	564	38.498	14.370	30.188	1.00	32.29	O
ATOM	4449	CB	LYS	A	564	38.296	15.821	27.591	1.00	30.45	C
ATOM	4450	CG	LYS	A	564	37.233	15.744	26.497	1.00	31.07	C
ATOM	4451	CD	LYS	A	564	36.111	14.775	26.849	1.00	30.53	C
ATOM	4452	CE	LYS	A	564	35.347	14.318	25.598	1.00	32.26	C
ATOM	4453	NZ	LYS	A	564	35.355	12.798	25.474	1.00	34.40	N1+
ATOM	4454	N	GLU	A	565	39.050	16.383	31.006	1.00	34.20	N
ATOM	4455	CA	GLU	A	565	39.522	15.830	32.269	1.00	33.71	C
ATOM	4456	C	GLU	A	565	39.123	16.707	33.477	1.00	33.57	C
ATOM	4457	O	GLU	A	565	39.325	16.298	34.620	1.00	36.71	O
ATOM	4458	CB	GLU	A	565	41.038	15.547	32.203	1.00	31.00	C
ATOM	4459	CG	GLU	A	565	41.948	16.433	33.029	1.00	28.71	C
ATOM	4460	CD	GLU	A	565	41.705	17.906	32.810	1.00	28.14	C
ATOM	4461	OE1	GLU	A	565	41.476	18.343	31.664	1.00	27.94	O
ATOM	4462	OE2	GLU	A	565	41.605	18.611	33.826	1.00	31.46	O1-
ATOM	4463	N	THR	A	566	38.550	17.891	33.230	1.00	31.43	N
ATOM	4464	CA	THR	A	566	37.891	18.648	34.290	1.00	30.06	C
ATOM	4465	C	THR	A	566	36.888	17.740	34.978	1.00	31.89	C
ATOM	4466	O	THR	A	566	37.183	17.117	36.017	1.00	33.59	O
ATOM	4467	CB	THR	A	566	37.091	19.837	33.754	1.00	27.34	C
ATOM	4468	CG2	THR	A	566	37.562	21.118	34.385	1.00	29.75	C
ATOM	4469	OG1	THR	A	566	37.226	19.915	32.329	1.00	28.29	O
ATOM	4470	N	CYS	A	567	35.700	17.644	34.390	1.00	32.18	N

ATOM	4471	CA	CYS	A	567	34.654	16.825	34.979	1.00	31.39	C
ATOM	4472	C	CYS	A	567	34.889	15.378	34.594	1.00	29.93	C
ATOM	4473	O	CYS	A	567	34.352	14.909	33.590	1.00	29.42	O
ATOM	4474	CB	CYS	A	567	33.262	17.276	34.513	1.00	29.68	C
ATOM	4475	SG	CYS	A	567	33.055	19.080	34.407	1.00	25.19	S
ATOM	4476	N	PHE	A	568	35.686	14.688	35.405	1.00	28.61	N
ATOM	4477	CA	PHE	A	568	35.928	13.255	35.250	1.00	28.31	C
ATOM	4478	C	PHE	A	568	36.751	12.792	36.449	1.00	29.41	C
ATOM	4479	O	PHE	A	568	36.664	11.642	36.860	1.00	31.51	O
ATOM	4480	CB	PHE	A	568	36.729	12.998	33.976	1.00	25.95	C
ATOM	4481	CG	PHE	A	568	36.206	11.876	33.139	1.00	21.28	C
ATOM	4482	CD1	PHE	A	568	35.062	12.035	32.394	1.00	20.57	C
ATOM	4483	CD2	PHE	A	568	36.903	10.698	33.038	1.00	20.14	C
ATOM	4484	CE1	PHE	A	568	34.622	11.051	31.574	1.00	17.15	C
ATOM	4485	CE2	PHE	A	568	36.471	9.710	32.217	1.00	17.57	C
ATOM	4486	CZ	PHE	A	568	35.321	9.888	31.481	1.00	19.73	C
ATOM	4487	N	ALA	A	569	37.683	13.636	36.877	1.00	28.87	N
ATOM	4488	CA	ALA	A	569	38.383	13.420	38.134	1.00	28.65	C
ATOM	4489	C	ALA	A	569	37.468	13.939	39.220	1.00	28.03	C
ATOM	4490	O	ALA	A	569	37.356	13.378	40.309	1.00	26.78	O
ATOM	4491	CB	ALA	A	569	39.710	14.213	38.138	1.00	30.00	C
ATOM	4492	N	GLU	A	570	36.832	15.057	38.898	1.00	30.62	N
ATOM	4493	CA	GLU	A	570	36.151	15.878	39.882	1.00	30.63	C
ATOM	4494	C	GLU	A	570	34.945	15.123	40.390	1.00	30.05	C
ATOM	4495	O	GLU	A	570	35.001	14.560	41.483	1.00	29.90	O
ATOM	4496	CB	GLU	A	570	35.749	17.210	39.258	1.00	28.89	C
ATOM	4497	CG	GLU	A	570	36.936	18.084	38.893	1.00	27.66	C
ATOM	4498	CD	GLU	A	570	36.512	19.417	38.301	1.00	29.60	C
ATOM	4499	OE1	GLU	A	570	35.295	19.714	38.265	1.00	27.40	O
ATOM	4500	OE2	GLU	A	570	37.407	20.200	37.922	1.00	31.42	O1-
ATOM	4501	N	GLU	A	571	33.921	15.009	39.544	1.00	28.64	N
ATOM	4502	CA	GLU	A	571	32.793	14.109	39.781	1.00	29.73	C
ATOM	4503	C	GLU	A	571	33.172	12.828	40.555	1.00	29.42	C
ATOM	4504	O	GLU	A	571	32.703	12.600	41.666	1.00	26.88	O
ATOM	4505	CB	GLU	A	571	32.153	13.698	38.444	1.00	31.90	C
ATOM	4506	CG	GLU	A	571	31.887	14.842	37.444	1.00	33.12	C
ATOM	4507	CD	GLU	A	571	30.475	15.396	37.551	1.00	31.21	C
ATOM	4508	OE1	GLU	A	571	30.276	16.300	38.392	1.00	29.20	O
ATOM	4509	OE2	GLU	A	571	29.584	14.930	36.791	1.00	28.63	O1-
ATOM	4510	N	GLY	A	572	34.072	12.032	39.986	1.00	29.49	N
ATOM	4511	CA	GLY	A	572	34.301	10.677	40.478	1.00	28.71	C
ATOM	4512	C	GLY	A	572	34.559	10.612	41.965	1.00	25.97	C
ATOM	4513	O	GLY	A	572	34.133	9.670	42.649	1.00	25.03	O
ATOM	4514	N	LYS	A	573	35.292	11.596	42.463	1.00	25.00	N
ATOM	4515	CA	LYS	A	573	35.292	11.839	43.886	1.00	26.40	C
ATOM	4516	C	LYS	A	573	33.907	12.403	44.227	1.00	27.40	C
ATOM	4517	O	LYS	A	573	33.053	11.637	44.652	1.00	28.80	O
ATOM	4518	CB	LYS	A	573	36.422	12.802	44.278	1.00	25.67	C
ATOM	4519	CG	LYS	A	573	37.835	12.284	43.924	1.00	24.04	C
ATOM	4520	CD	LYS	A	573	38.725	12.132	45.169	1.00	21.49	C
ATOM	4521	CE	LYS	A	573	39.633	13.331	45.408	1.00	17.20	C
ATOM	4522	NZ	LYS	A	573	40.867	12.848	46.062	1.00	15.20	N1+
ATOM	4523	N	LYS	A	574	33.581	13.580	43.695	1.00	26.75	N
ATOM	4524	CA	LYS	A	574	32.400	14.324	44.131	1.00	25.51	C
ATOM	4525	C	LYS	A	574	30.989	13.898	43.651	1.00	25.42	C
ATOM	4526	O	LYS	A	574	30.011	14.569	44.005	1.00	25.17	O
ATOM	4527	CB	LYS	A	574	32.612	15.817	43.872	1.00	27.14	C
ATOM	4528	CG	LYS	A	574	32.851	16.209	42.416	1.00	28.72	C
ATOM	4529	CD	LYS	A	574	32.816	17.739	42.193	1.00	28.75	C
ATOM	4530	CE	LYS	A	574	31.382	18.254	41.916	1.00	28.20	C
ATOM	4531	NZ	LYS	A	574	31.384	19.599	41.254	1.00	28.78	N1+
ATOM	4532	N	LEU	A	575	30.860	12.683	43.105	1.00	23.44	N
ATOM	4533	CA	LEU	A	575	29.559	12.086	42.759	1.00	25.31	C
ATOM	4534	C	LEU	A	575	29.409	10.605	43.176	1.00	27.47	C
ATOM	4535	O	LEU	A	575	28.300	10.045	43.137	1.00	26.81	O
ATOM	4536	CB	LEU	A	575	29.301	12.205	41.264	1.00	24.20	C
ATOM	4537	CG	LEU	A	575	28.778	10.958	40.541	1.00	24.99	C
ATOM	4538	CD1	LEU	A	575	27.265	11.091	40.260	1.00	22.96	C
ATOM	4539	CD2	LEU	A	575	29.587	10.746	39.253	1.00	23.54	C
ATOM	4540	N	VAL	A	576	30.545	9.953	43.443	1.00	30.48	N
ATOM	4541	CA	VAL	A	576	30.594	8.583	43.980	1.00	28.40	C

```
ATOM   4542  C   VAL A 576      30.933   8.698  45.451  1.00 26.50           C
ATOM   4543  O   VAL A 576      30.389   7.969  46.264  1.00 26.24           O
ATOM   4544  CB  VAL A 576      31.703   7.709  43.313  1.00 29.72           C
ATOM   4545  CG1 VAL A 576      31.613   6.269  43.830  1.00 31.31           C
ATOM   4546  CG2 VAL A 576      31.568   7.728  41.788  1.00 28.27           C
ATOM   4547  N   ALA A 577      31.839   9.614  45.783  1.00 26.23           N
ATOM   4548  CA  ALA A 577      32.042  10.018  47.171  1.00 27.55           C
ATOM   4549  C   ALA A 577      30.723  10.465  47.754  1.00 27.60           C
ATOM   4550  O   ALA A 577      30.498  10.341  48.959  1.00 29.06           O
ATOM   4551  CB  ALA A 577      33.057  11.163  47.280  1.00 24.38           C
ATOM   4552  N   ALA A 578      29.896  11.084  46.921  1.00 26.48           N
ATOM   4553  CA  ALA A 578      28.663  11.639  47.421  1.00 28.56           C
ATOM   4554  C   ALA A 578      27.604  10.545  47.504  1.00 30.37           C
ATOM   4555  O   ALA A 578      26.515  10.788  48.040  1.00 32.62           O
ATOM   4556  CB  ALA A 578      28.198  12.775  46.522  1.00 27.95           C
ATOM   4557  N   SER A 579      27.969   9.336  47.045  1.00 30.43           N
ATOM   4558  CA  SER A 579      27.041   8.214  46.794  1.00 28.38           C
ATOM   4559  C   SER A 579      26.717   7.338  48.012  1.00 28.20           C
ATOM   4560  O   SER A 579      27.423   7.379  49.026  1.00 27.16           O
ATOM   4561  CB  SER A 579      27.600   7.308  45.678  1.00 26.10           C
ATOM   4562  OG  SER A 579      27.127   7.695  44.407  1.00 21.67           O
ATOM   4563  N   GLN A 580      25.858   6.349  47.756  1.00 28.20           N
ATOM   4564  CA  GLN A 580      25.294   5.456  48.775  1.00 30.20           C
ATOM   4565  C   GLN A 580      25.251   5.794  50.275  1.00 29.79           C
ATOM   4566  O   GLN A 580      24.240   6.301  50.763  1.00 29.46           O
ATOM   4567  CB  GLN A 580      25.887   4.059  48.652  1.00 31.82           C
ATOM   4568  CG  GLN A 580      24.809   3.035  48.450  1.00 35.01           C
ATOM   4569  CD  GLN A 580      24.605   2.755  46.984  1.00 39.20           C
ATOM   4570  NE2 GLN A 580      24.214   3.795  46.222  1.00 39.46           N
ATOM   4571  OE1 GLN A 580      24.873   1.642  46.513  1.00 37.19           O
ATOM   4572  N   ALA A 581      26.147   5.153  51.022  1.00 28.09           N
ATOM   4573  CA  ALA A 581      25.904   4.949  52.433  1.00 28.48           C
ATOM   4574  C   ALA A 581      26.749   5.880  53.305  1.00 31.33           C
ATOM   4575  O   ALA A 581      27.878   5.542  53.689  1.00 33.68           O
ATOM   4576  CB  ALA A 581      26.139   3.517  52.786  1.00 27.41           C
ATOM   4577  N   ALA A 582      26.292   7.133  53.354  1.00 32.13           N
ATOM   4578  CA  ALA A 582      26.690   8.140  54.330  1.00 30.12           C
ATOM   4579  C   ALA A 582      25.992   9.430  53.865  1.00 32.98           C
ATOM   4580  O   ALA A 582      25.336  10.117  54.686  1.00 34.50           O
ATOM   4581  CB  ALA A 582      28.212   8.325  54.362  1.00 23.03           C
ATOM   4582  N   LEU A 583      26.076   9.711  52.548  1.00 32.99           N
ATOM   4583  CA  LEU A 583      25.497  10.927  51.932  1.00 32.69           C
ATOM   4584  C   LEU A 583      24.530  10.469  50.841  1.00 33.35           C
ATOM   4585  O   LEU A 583      23.484  11.124  50.604  1.00 31.85           O
ATOM   4586  CB  LEU A 583      26.588  11.815  51.276  1.00 28.59           C
ATOM   4587  CG  LEU A 583      27.733  12.582  51.993  1.00 26.24           C
ATOM   4588  CD1 LEU A 583      27.189  13.656  52.930  1.00 24.68           C
ATOM   4589  CD2 LEU A 583      28.651  11.626  52.741  1.00 22.20           C
HETATM 4590  C   UNK     0      -9.036  -2.832  16.564  0.00  0.00       .001 A
HETATM 4591  C   UNK     0      -8.764  -0.784  15.364  0.00  0.00       .002 A
HETATM 4592  C   UNK     0      -7.781  -3.248  16.143  0.00  0.00       .002 A
HETATM 4593  C   UNK     0      -9.530  -1.618  16.181  0.00  0.00       .014 A
HETATM 4594  C   UNK     0      -8.977   0.540  14.776  0.00  0.00       .028 A
HETATM 4595  C   UNK     0      -7.011  -2.444  15.332  0.00  0.00       .030 A
HETATM 4596  C   UNK     0      -7.493  -1.203  14.930  0.00  0.00       .042 A
HETATM 4597  C   UNK     0     -10.196   1.410  14.944  0.00  0.00       .087 C
HETATM 4598  N   UNK     0      -9.986   3.761  14.293  0.00  0.00       .093 N
HETATM 4599  C   UNK     0      -7.878   0.834  14.064  0.00  0.00       .096 A
HETATM 4600  H   UNK     0      -6.112  -0.213  13.713  0.00  0.00       .165 H
HETATM 4601  H   UNK     0     -11.058   2.325  11.268  0.00  0.00       .167 H
HETATM 4602  C   UNK     0     -11.650   2.380  13.186  0.00  0.00       .174 C
HETATM 4603  H   UNK     0      -9.014   3.946  14.093  0.00  0.00       .314 H
HETATM 4604  H   UNK     0     -10.525   4.400  13.728  0.00  0.00       .314 H
HETATM 4605  C   UNK     0     -10.269   2.410  13.791  0.00  0.00       .318 C
HETATM 4606  N   UNK     0      -6.980  -0.196  14.144  0.00  0.00       .365 N
HETATM 4607  O   UNK     0     -12.618   2.247  13.897  0.00  0.00       .648 O
HETATM 4608  O   UNK     0     -11.804   2.501  11.858  0.00  0.00       .775 O
CONECT 4590 4592 4593
CONECT 4591 4593 4594 4596
CONECT 4592 4590 4595
CONECT 4593 4591 4590
```

```
CONECT 4594 4591 4599 4597
CONECT 4595 4592 4596
CONECT 4596 4591 4595 4606
CONECT 4597 4594 4605
CONECT 4598 4603 4604 4605
CONECT 4599 4594 4606
CONECT 4600 4606
CONECT 4601 4608
CONECT 4602 4607 4608 4605
CONECT 4603 4598
CONECT 4604 4598
CONECT 4605 4598 4597 4602
CONECT 4606 4599 4596 4600
CONECT 4607 4602
CONECT 4608 4602 4601
END
```
